# Symptom Persistence Relates to Volume and Asymmetry of the Limbic System after Mild Traumatic Brain Injury

**DOI:** 10.3390/jcm13175154

**Published:** 2024-08-30

**Authors:** Cheryl Vanier, Priya Santhanam, Nicholas Rochester, Lauren Carter, Mike Lim, Amir Kilani, Shivani Venkatesh, Sherwin Azad, Thomas Knoblauch, Tapasya Surti, Colin Brown, Justin Roy Sanchez, Leon Ma, Shaunaq Parikh, Leo Germin, Enrico Fazzini, Travis H. Snyder

**Affiliations:** 1 Imgen Research Group, Las Vegas, NV 89118, USA; psan.nicoe@gmail.com (P.S.); travishsnyder@gmail.com (T.H.S.); 2 College of Osteopathic Medicine, Touro University Nevada, Henderson, NV 89014, USA; 3 College of Medicine, Central Michigan University, Midland, MI 48859, USA; 4 Department of Radiology, Sunrise Health Graduate Medical Education Consortium, Las Vegas, NV 89128, USA; 5 Department of Interdisciplinary Health Sciences, University of Nevada, Las Vegas, NV 89557, USA; 6 Department of Neurology, University of Texas Health Science Center, Houston, TX 78701-2982, USA; 7 Department of Anesthesiology, Loyola University Medical Center, Maywood, IL 60153, USA; 8 Department of Family Medicine, University of Pittsburgh, Pittsburgh, PA 15260, USA; 9 Clinical Neurology Specialists, Las Vegas, NV 89147, USA; 10 Department of Radiology, HCA Healthcare, Mountain View Hospital, Las Vegas, NV 89166, USA; 11 SimonMed Imaging, Las Vegas, NV 89121, USA

**Keywords:** brain concussion, diagnostic imaging, laterality, magnetic resonance imaging, mild traumatic brain injury

## Abstract

**Background**: Persistent symptoms have been reported in up to 50% of the 27 million people with mild traumatic brain injuries (mTBI) every year. MRI findings are currently limited by low diagnostic and prognostic sensitivities, constraining the value of imaging in the stratification of patients following mTBI. Limbic system structures are promising brain regions in offering prognostic factors for symptom persistence following mTBI. The objective of this study was to associate volume and symmetry of limbic system structures with the presence and persistence of common symptoms in patients with mTBI. **Methods:** This study focused on 524 adults (aged 18–82), 58% female, with 82% injured in motor vehicle accidents and 28% reporting loss of consciousness (LOC). Magnetic resonance imaging (MRI) data included a sagittal 3D T1-weighted sequence with 1.2 mm slice thickness, with voxel sizes of 0.93 mm × 0.93 mm × 1.2 mm, obtained a median of 156 days after injury. Symptom diagnosis and persistence were collected retrospectively from patient medical records. Intracranial volume-adjusted regional volumes per side utilizing automated volumetric analysis (NeuroQuant^®^) were used to calculate total volume, laterality index, and side-independent asymmetry. Covariates included age, sex, LOC, and days from injury. Limbic volumetrics did not relate to symptom presentation, except the (-) association between headache presence and thalamus volume (adjusted odds ratio = 0.51, 95% confidence interval = 0.32, 0.85). Headache, balance problems, anxiety, and depression persistence was (-) associated with thalamus volume (hazard ratio (HR) 1.25 to 1.94). Longer persistence of balance problems was associated with (-) lateral orbitofrontal cortex volume (HR = 1.33) and (+) asymmetry of the hippocampus (HR = 0.27). Persistence of cognitive deficits was associated with (+) asymmetry in the caudal anterior cingulate (HR = 0.67). Depression persistence was associated with (+) asymmetry in the isthmus of the cingulate gyrus (HR = 5.39). Persistence of anxiety was associated with (-) volume of the parahippocampal gyrus (HR = 1.67), orbitofrontal cortex (HR > 1.97), and right-biased laterality of the entorhinal cortex (HR = 0.52). **Conclusions:** Relative volume and asymmetry of the limbic system structures in patients with mTBI are associated with the persistence of symptoms, particularly anxiety. The conclusions of this study are limited by the absence of a reference group with no mTBI.

## 1. Introduction

Every year, 1.6–3.8 million individuals in the United States suffer a mild traumatic brain injury (mTBI) or concussion, with most cases resulting from motor vehicle accidents, motorcycle crashes, interactions with firearms, and falls [1,2,3]. Diagnosis of mTBI may employ several clinical criteria during initial and subsequent evaluations, but there is general agreement that Glasgow Coma Scale (GCS) scores between 13 and 15 after sustaining an injury involving physical forces that affect the brain are criteria for mTBI diagnosis [4]. In the acute phase immediately after mTBI, patients often experience symptoms such as headache, fatigue, irritability, cognitive dysfunction, and depression [5,6]. While for some people symptoms resolve quickly, it is relatively common for symptoms to become chronic, persisting three months or longer post-injury.

Based on the International Classification of Diseases, 10th revision (ICD-10) guidelines [7], long-term sequelae of mTBI can be diagnosed as “post-concussive syndrome” (PCS) when three of the following eight symptoms persist for at least three months after the date of injury: headache, dizziness, fatigue, irritability, insomnia, difficulty concentrating, memory difficulties, or intolerance to stress, emotion, or alcohol. Early reports suggesting that prolonged symptoms occurred after mTBI in less than 10% of patients were likely significant underestimations [4], as more recent estimates with larger patient cohorts and pooled analyses suggest much higher values [5,8]. The major public health and quality of life consequences of prolonged symptoms after mTBI were illustrated in a TRACK-TBI cohort study [9]. Three-quarters of the study population reported at least one PCS symptom <1 year post-injury. Patients also reported significantly reduced satisfaction with life scores at 6 and 12 months after injury, and one-third of study participants failed to return to full functional status after 6 months. Mild TBIs can therefore significantly reduce quality of life in the months and years after an injury [5,6,8,10].

Biomarkers indicating risk for prolonged symptoms post-mTBI can help physicians proactively manage and mitigate the more serious consequences of long-term symptom persistence after mTBI. However, the groups based on a PCS diagnosis group patients who have different sets of symptoms with potentially unrelated etiology in the brain, contributing to uncertainty in prognosing and treating long-term sequelae from mTBI. A symptom-based approach relative to regions of interest (ROI) in the brain that are prone to injury or that mechanistically influence symptoms may offer superior diagnostic and prognostic indicators in the imaging of patients with mTBI.

Many of the persistent symptoms after mTBI are in the cognitive or psychiatric domains [7], which are closely related to functionality of the limbic system. Although mapping complex cognitive and emotional behaviors to specific brain structures is often challenging, the limbic system is generally understood to include brain structures closely associated with learning, memory, and emotion. The structures of the limbic system are located primarily in the medial temporal lobe but with closely affiliated components located in the diencephalon and midbrain. The specific brain structures associated with the limbic system have not been defined in a way that has been universally accepted [11]. In addition to the amygdala, hippocampus, and parahippocampal gyrus, there is strong evidence that understanding limbic system function requires inclusion of additional nearby structures and surrounding cortices, including the cingulate [12] and the orbitofrontal cortex [13] as well as the thalamus and hypothalamus [14,15]. For this study, structures were included if they were segmented in NeuroQuant^®^ 3.0 software and they were identified as being associated with the limbic system in textbooks or the primary literature (e.g., [14,16]).

The ROI in the limbic system are vulnerable to biomechanical injury (reviewed in [17]) by virtue of their extensive connectivity with other structures, position near the midbrain, and anatomical structure [18]. For example, the hippocampus is a biomarker for mTBI because it is vulnerable to hypoxia and ischemia, has a predilection for select neurotransmitters that can create conditions for ongoing neuronal excitation, and is prone to shearing injuries because it has a thin base and adjacent fluid [19]. Connections between the hippocampus and entorhinal cortex may be affected by injury to the hippocampus [17,20]. Damage affecting connectivity and microstructure may in time result in volumetric changes by triggering neuroinflammation, particularly during the chronic phase of injury. However, volumetric and functional differences between mTBI subjects and controls have been reported as soon as two weeks after injury in the hippocampus [21].

Due to technological advances, automated volumetric analysis has been increasingly incorporated into clinical practice and has become a focus of research in many neurological conditions, including mTBI. Although laterality has been recognized as an important concept in neuroradiology, side-independent asymmetry has not, even though there is cause to suspect it may be important based on the existing understanding of functional brain structure. Examples include Leh et al., who offered a mechanistic explanation for general interhemispheric differences in structural damage following mTBI due to higher left hemispheric density of axon branching [22], and Derakhshan, who reviewed a model of lateralized brain activation in the context of rehabilitation that highlighted the importance of side-based dominance and connectivity through the corpus callosum for highly lateralized tasks [23]. An analysis of left and right sides separately cannot shed light on the importance (or lack thereof) of asymmetry or laterality. This is not a new insight, as an “asymmetry index”,—which is a variant of a published laterality index for fMRI—is available in, for example, NeuroQuant^®^ commercial software (Cortechs.AI). In this study, in addition to a laterality index, we introduce a new metric to differentiate between laterality and side-independent asymmetry.

The effects of volumetric laterality or asymmetry following mTBI require more study. Observed asymmetries may be induced by injury or they may pre-exist the injury. It is currently unclear whether the few reported relationships between right–left hemisphere volumetric differences and symptoms are rooted in reduced functionality; however, previous studies report that in addition to total volume, laterality (the relative allocation of volume between hemispheres) of cortical matter and white matter can differ by TBI severity and is correlated with unfavorable outcomes [24]. Whether unfavorable outcomes are due to a greater impact of injury, affecting both structural and functional asymmetry, can only be ascertained by direct functional evidence.

Prior work associating imaging biomarkers with symptoms after mTBI strongly suggest that patients diagnosed with mTBI are not a monolithic group; it seems there are sub-groups that vary in their patterns of symptom appearance and persistence [8]. The current study builds on the previous literature by characterizing neuroimaging findings in limbic structures relative to symptom persistence and, secondarily, symptom presentation, within a large cohort of patients with mTBI. The hypothesis was that regional volume and asymmetry in the limbic system would be associated with symptom-specific outcomes, such as slower rates of improvement or greater likelihood of presentation. The main findings suggest that limbic volumetrics are not associated with symptom presentation. However, small volume and greater asymmetry in certain limbic regions of interest, such as the thalamus, may indicate patients who suffer persistent headache, balance deficits, cognitive problems, fatigue, and neuropsychiatric symptoms—particularly anxiety.

## 2. Materials and Methods

This retrospective cohort study included adult patients (>18 years of age) diagnosed with mTBI by a board-certified neurologist specializing in head trauma based on standard DSM-V criteria [25]. The patients, all of whom were in litigation, include a consecutive series who visited one of two neurologists in private practice between 2011 and 2019 and were scanned under suspicion of compromised brain integrity after diagnosis of mTBI. Exclusion criteria included incomplete records, canceled/incomplete magnetic resonance imaging (MRI), >3 years between the date of injury and consultation, history of cerebrovascular accident, demyelinating disease, chronic epilepsy, previous head trauma, brain tumors, previous brain surgery, and imaging findings of cavernoma or cerebral vascular malformation, as previously defined [26]. Patients with brain contusions or evidence of intracranial hemorrhage, including traumatic microhemorrhage, were excluded. This study was conducted in accordance with the Declaration of Helsinki and determined exempt by Touro University Nevada’s Institutional Review Board (IRB4-5-17D, 15 March 2017).

During the initial neurology exam preceding the MRI, the presence or absence of seven common mTBI symptoms were noted, including headache, balance problems, overall cognitive deficits, fatigue, anxiety, depression, and emotional lability. Headaches and fatigue were based on participant self-reports. Balance problems were diagnosed by the neurologist and often confirmed by a physical test such as those reviewed in [27]. Cognitive deficits were identified based on standard cognitive testing [28] and assessment by the neurologist. Since no standard testing protocol was used during the neurology consultation, ”cognitive deficits” in this study were broadly defined to include issues with attention, learning and memory, frontal executive functions, and language and communication. Anxiety, depression, and emotional lability were diagnosed using standard DSM-V diagnostic criteria. The presence or absence of each symptom during irregularly spaced consultations with the neurologists was noted during a retrospective review of the medical chart. For analysis, the time elapsed between date of injury and each office visit was noted, and clinical symptoms were reported as sustained, improved, or resolved. Symptoms were considered sustained if they were approximately the same or worse, based on the neurologist’s assessment and self-report. Improved or resolved symptoms were combined into a single category.

All imaging was performed using either a Siemens Magnetom Verio 3T Scanner or an MR Signa HDxt system (G.E. Healthcare, Milwaukee, WI, USA) equipped with an 8-channel head coil. Sagittal 3D T1-weighted sequence was acquired with a 1.2 mm slice thickness with voxel sizes of 0.93 mm × 0.93 mm × 1.2 mm (TE 2.208 ms, TR 5.396 ms). All MRI images were reviewed by two blinded board-certified neuroradiologists, with any discrepancies resolved by consensus. Volumetric analysis and T1 images were reviewed for motion artifacts and segmentation errors, with subsequent exclusion of images whose integrity appeared to be compromised. The T1 images were processed with NeuroQuant^®^ version 3.0 for volumetric analysis. NeuroQuant^®^ may be more sensitive than radiological inspection alone, particularly in cases of mild injury where structural changes are less obvious [29,30].

**Figure 1:**
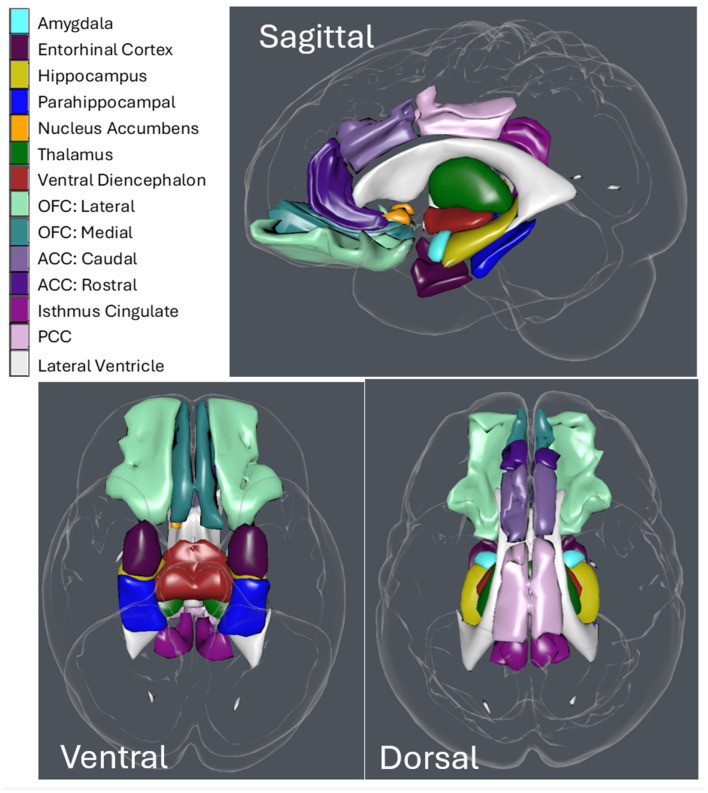
Color-coded limbic ROI (based on OASIS-TRT-20 joint fusion Atlas in Scaleable Brain Atlas) in three views, with lateral ventricle for context. Scaleable Brain Atlas (https://scalablebrainatlas.incf.org/ (accessed on 7 July 2024)).

Based on NeuroQuant^®^ segmentation, the volume of right and left sides of the following limbic system regions of interest were identified: amygdala, parahippocampal gyrus, hippocampus, entorhinal cortex, anterior cingulate cortex (ACC) divided into rostral (rACC) and caudal (cACC) sub-sections, posterior cingulate cortex (PCC), orbitofrontal cortex (OFC) divided into lateral (lOFC) and medial (mOFC) portions, the isthmus of the cingulate gyrus, the ventral diencephalon, nucleus accumbens, and thalamus (Figure 1). The ventral diencephalon ROI provided by NeuroQuant^®^ included several structures that could not be segmented individually: the hypothalamus, mammillary body, subthalamic nuclei, substantia nigra, red nucleus, lateral and medial geniculate nuclei, along with white matter corresponding to the zona incerta cerebral peduncle, lenticular fasciculus, and medial lemniscus [31].

The right and left sides for each region of interest (ROI) were first adjusted for total intracranial volume (TIV) using linear regression to minimize sex differences related to systematic size variation [32]. To summarize and separately analyze the overall volume relative to asymmetry between right and left volume, three metrics were calculated:

Total volume = Vright + Vleft(1)

Laterality index (LI) = (Vright − Vleft)/(Vright + Vleft)(2)

Asymmetry or Side-independent laterality index (siLI) = |LI-median(LI)|(3)

where V = TIV-adjusted volume [33]. The range of the resulting LI was [−1 (left-biased), 1 (right-biased)]. The siLI range was [0 (median symmetry for the ROI), 1 (maximum asymmetry, with all volume on one side)]. Volumes were analyzed as z-scores, and the LI and si-LI were multiplied by 10 for analysis. Sex, age, loss of consciousness (LOC), and time between the injury and image acquisition were covariates that had the potential to influence or mediate symptom presentation or longevity. Age was included in the model as a dichotomous variable (<40 and >40). The date of injury (DOI) to date of scan (DOS) was log_10_ transformed. The de-identified volumetric, symptom, and covariate datasets are available from Figshare (see below).

Demographic comparisons between LOC groups were based on Fisher’s exact tests, Mann–Whitney tests, or *t*-tests. Relationships among the LI and siLI from each ROI were based on Kendall’s correlations, whereas Pearson’s correlations were used for total volume z-scores. For symptom co-occurrence, groups of more than 10 participants (defined by presence and absence of symptoms), or which had expected size >10 based on probability of symptom combination, were analyzed. Headache was excluded from the analysis of symptom co-occurrence because it was present in 96% of patients.

Odds ratios (OR) and hazard ratios (HR) are biased when they are estimated with models that do not include appropriate covariates, or that incorporate unnecessary covariates [34]. The best subset of covariates to include in the logistic regression models for symptom presence was chosen based on a Least Absolute Shrinkage and Selection Operator (LASSO) with 10-fold cross-validation for model selection and with associated shrinkage estimators [34] using the glmnet [35] and Selective Inference R packages. Two-way interactions between covariates and limbic volumetric values were explored using nested models and included in the LASSO regression if *p* < 0.05 (log-likelihood test). Events per variable (EPV, number of ”events” in data/number of regression coefficients) had a range of 18 (for emotional lability) to 101 (headache), sufficient to avoid overfitting [36].

For the analysis of association between longevity of a symptom and limbic volumetric values, 3 of 7 symptoms had EPV < 7, so LASSO was not used [34]. Instead, baseline hazard functions associated with each symptom for data divided by sex, age (<40 or >40), and LOC at time of injury were first estimated as described below. For the final models, adjusted hazard ratios included LOC and the interaction between LOC and the ROI metric for all models, whereas age was included as a covariate only for the analysis of cognitive symptom persistence.

An association between volume, LI, or siLI and symptom persistence was estimated in the semi-parametric Cox proportional hazards model with interval-censoring and right-censoring as described in [26]. Analyses were conducted in R 4.3.0 software (R Core Team. 2023 with the “icenReg” package [37]. The Benjamini–Hochberg *p*-value correction procedure was applied to each table of 91 (13 ROI by 7 symptoms) *p*-values to inform interpretation [38]. Unadjusted relationships between symptoms and ROI volume, LI, or siLI were visualized in Kaplan–Meier plots based on z-score cut-offs (volume) or quantiles (LI and siLI).

## 3. Results

**Figure 2:**
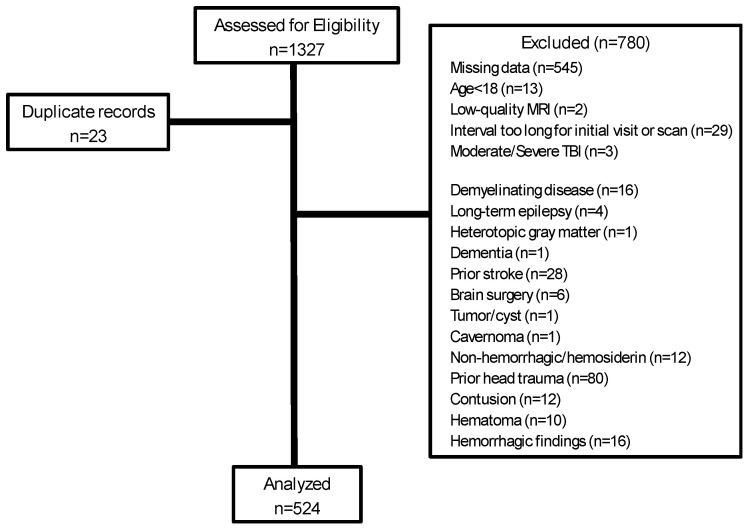
Flow diagram of records assessed for eligibility and exclusions.

Exclusions are detailed in Figure 2. Most exclusions were due to missing data, most commonly due to no MRI or no segmentation of MRI. Some patient records had significant missing details about injury, diagnosis, or other critical information that did not allow assessment for inclusion. The 524 participants were between the ages of 18 and 82, with a mean age of 42, and 302 (58%) were female (Table 1). Most (82%) of the participants were injured in motor vehicle accidents, and 108 (21%) had been diagnosed with post-traumatic stress disorder (PTSD). A median of 69 (range: 0 to 827) days passed between injury to first medical examination, and 156 (range: 11 to 1024) days from injury to scan. The 149 participants who lost consciousness at time of injury represented 28% of the total. The subset of participants with LOC were on average three years younger, more likely to be male (35% in no LOC group, 52% in LOC group), more likely to be injured by a fall, and more likely to have PTSD (27% compared to 18%) compared to participants who did not lose consciousness (Table 1; *p* < 0.05).

With the exceptions of headaches and emotional lability, prevalence of symptoms was higher and improvement or resolution rates lower in participants who reported LOC (Table 2). Symptoms co-occurred non-randomly in participants (Table S1). For example, balance problems and cognitive deficits occurred together without any other symptoms more often than expected by chance (86 expected, 125 observed). Headache (10 expected, 53 observed), balance problems (25 expected, 44 observed), and cognitive deficits (34 expected, 55 observed) occurred alone more than expected, and there were more participants with certain combinations of 4–6 symptoms than expected by chance (e.g., 5 with balance, cognitive, fatigue, anxiety, depression, and emotional lability were expected by chance, whereas 36 were observed). There were fewer participants with combinations of 2–3 symptoms than expected by chance, particularly balance problems and cognitive deficits in combinations with anxiety (45 expected, 10 observed), depression (36 expected, 3 observed), and fatigue (11 expected, 25 observed).

A summary of the right- and left-side volumes after TIV adjustment is presented in Table S2. The mean TIV in study participants was 1493 (standard deviation (SD) = 151 cm^3^; Table S3). Participants who reported LOC had larger average brain volume (1537 (155) cm^3^) than those who did not (1475 (146) cm^3^; *p* < 0.001), possibly due to the greater representation of males in the group with LOC. TIV-adjusted volumes and laterality indices were similar for those with and without LOC (Table S3), and there was no evidence for sex differences in the TIV-adjusted ROI. The median LI for the rostral ACC (LI = 0.24), caudal ACC (LI = 0.33), and medial OFC (LI = 0.26) but not the lateral OFC (LI = −0.05) were right-biased, whereas the other ROI did not indicate strongly biased laterality (Table S3). The TIV-adjusted volumes, LI and siLI, were not moderately or highly correlated among limbic brain ROI, with a few exceptions (Table S4). The highest correlation (*r* = 0.60) was between the lateral OFC and the medial OFC.

### 3.1. Symptom Presentation

An overview of the relationships between limbic ROI and symptom presentation is displayed in Table 3, with coefficients reported in Table 4, Tables S5 and S6. Participants who reported LOC at the time of injury had greater odds of presenting with balance problems (odds ratio (OR) = 1.91), cognitive deficits (2.11), fatigue (1.58), anxiety (1.50), depression (2.28), and emotional lability (2.09) but there was no evidence for an association with headache (1.48). Participants over 40 were more likely to present with balance problems (1.55) or cognitive deficits (1.51) but not headache, fatigue, anxiety, depression, or emotional lability (OR < 1.28). There was no evidence that symptom presentation was influenced by sex or the number of days between injury and diagnosis. Presentation of headaches was less likely in participants with large TIV-adjusted thalamus volumes (OR = 0.51, mean thalamus z-score with/without headache = −0.02/0.59). There was no evidence after *p*-value adjustment that the presentation of the other six symptoms was associated with limbic system volumetrics.

### 3.2. Symptom Persistence

**Figure 3:**
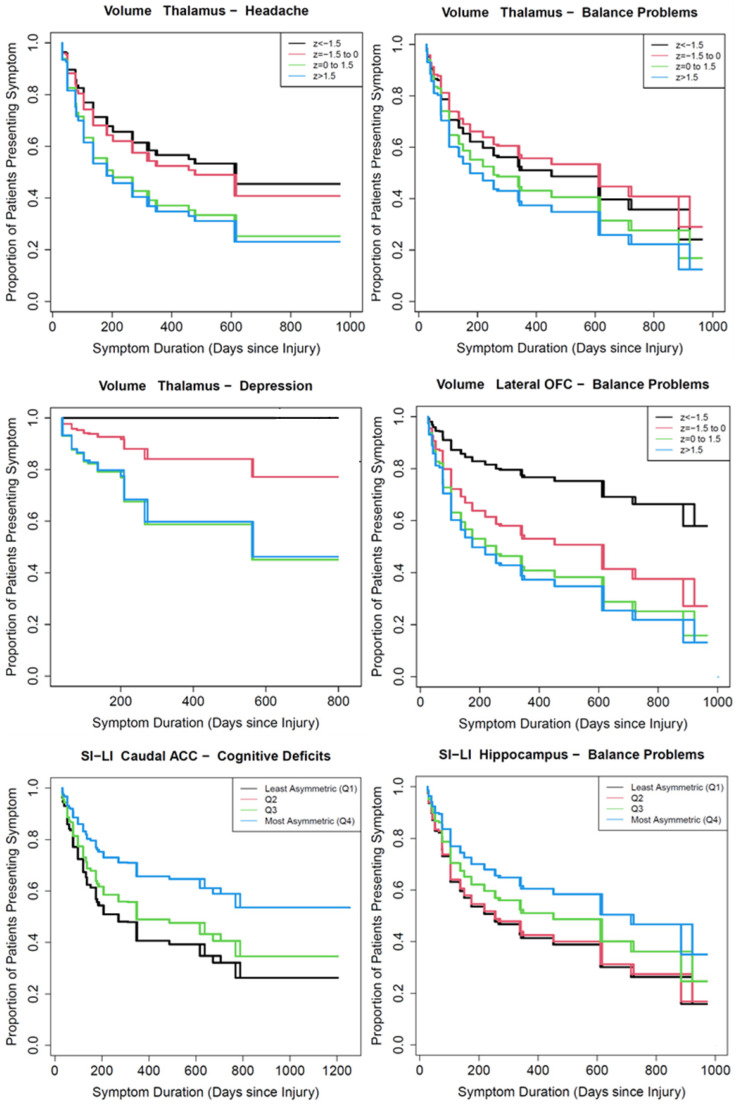
Kaplan–Meier curves of symptom persistence for headache, balance problems, cognitive deficits, and depression relative to volume, LI, or siLI of selected brain ROI. TIV-adjusted volumes were divided into groups based on z-scores, and LI and siLI were divided into quartiles, from most right-biased (least asymmetric for siLI) to most left-biased (most asymmetric for siLI), for headache, balance, or cognitive symptoms, or into two groups for the other symptoms, to illustrate trends from Table 5. The brain ROI and symptoms shown are the subset with the strongest statistical support for a relationship between symptom persistence and brain ROI. Relevant sample sizes are shown in Table 2.

**Figure 4:**
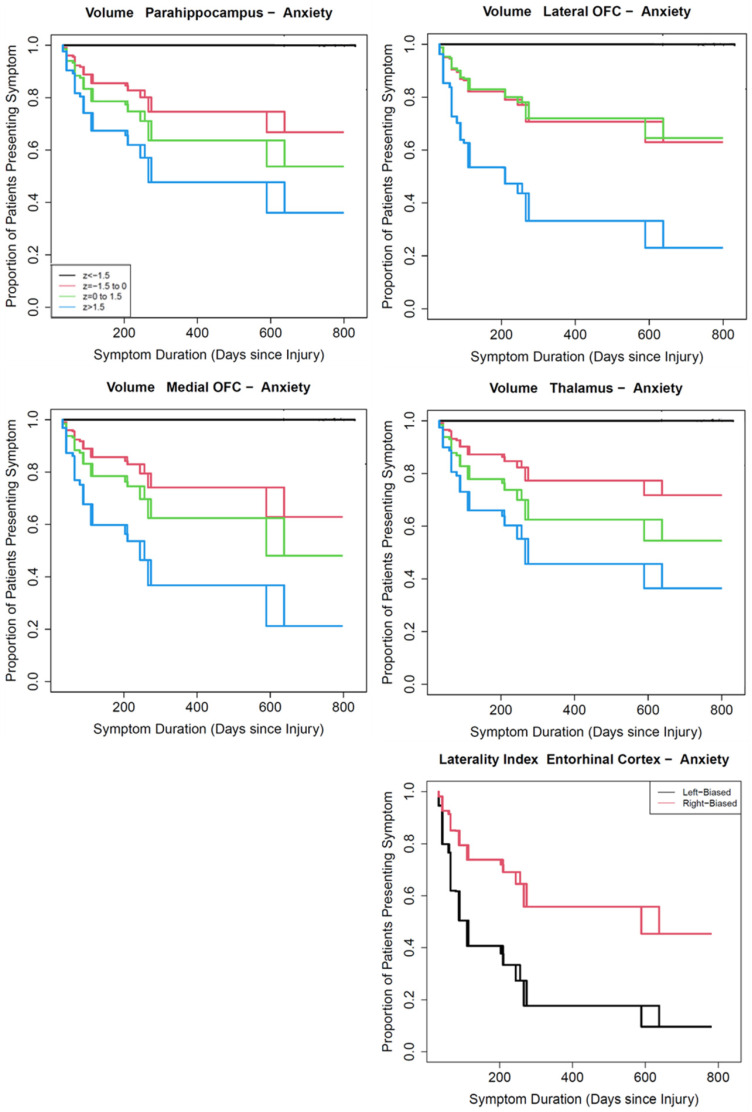
Anxiety persistence relative to volume, LI, or siLI of selected brain ROI. Details follow Figure 3. Relevant sample sizes are shown in Table 2.

An overview of relationships between limbic ROI and symptom persistence is displayed in Table 3, with coefficients reported in Table 5 and visualized in Figure 2, Figure 3 and Figure 4 and Supplementary Figures S1–S3. Participants who reported LOC at time of injury had headaches (HR = 1.46), balance problems (HR = 1.70), and cognitive deficits (HR = 1.49) that persisted longer than those who did not report LOC (Table 5). Sex was not associated with persistence of any symptoms studied, and age was associated only with persistence of cognitive deficits (OR = 1.57). After adjustment for multiple comparisons, no limbic structure volumetrics were associated with persistence of fatigue or emotional lability, although results for emotional lability should be considered preliminary due to the small number of participants with this symptom and the small number of those who improved or recovered.

Long-term headache persistence was associated with participants who had a relatively small thalamus (adjusted HR = 1.25) (Table 5, Figure 3, Supplementary Figures). Balance problems were more persistent in participants who had smaller lateral OFC (HR = 1.33) and thalamus (HR = 1.28) volumes, and large siLI—indicating high asymmetry of the hippocampus (HR = 0.27)—was also associated with slower recovery from balance problems. There was no strong evidence for an association between the volume and laterality of limbic system ROI and persistence of cognitive deficits, but greater overall asymmetry (based on the siLI) of the caudal ACC (HR = 0.67) was associated with longer persistence of cognitive deficits. Persistent depression was associated with smaller thalamus (HR = 1.79) volumes and with a less asymmetric isthmus of the cingulate gyrus (5.39).

Slower improvement or resolution of anxiety was associated with having smaller TIV-adjusted parahippocampal gyrus (HR = 1.67), lateral OFC (HR = 1.97), medial OFC (HR = 2.23), and thalamus (HR = 1.94) volumes (Figure 4). Participants with right-biased LI in the entorhinal cortex had slower recovery from anxiety than those with left-biased LI (HR = 0.52). There was no evidence that side-independent asymmetry was associated with persistence of anxiety.

## 4. Discussion

This study investigated the relationship between regional volumetrics of limbic structures and symptom presentation and longevity in a large cohort of patients with mTBI. While previous work has investigated relationships between symptoms and relative volume or atrophy in brain structures, this study directly addressed laterality and asymmetry. The most novel finding is that small volume is associated with persistence of symptoms for some ROI, such as the parahippocampal gyrus, OFC, and thalamus, while the asymmetry (with or without regard to side) appears to be more closely related to symptom longevity for other ROI, such as the hippocampus, caudal ACC, isthmus of the cingulate cortex, and entorhinal cortex. There was a unique set of volumetric findings that predicted the longevity of each symptom, with a unifying theme that smaller volumes and greater asymmetry were associated with longer persistence of symptoms.

Patient prognosis after mTBI is complicated by the fact that the injuring forces—particularly the rotational forces—act on the midbrain, inducing neuronal injuries and irritation to blood vessels in areas known to impact consciousness, emotions, memory, headaches, fatigue, and hormone levels [4]. However, mechanisms of injury vary significantly in patients with mTBI and often include acceleration, deceleration, and direct impact, stemming from multidirectional force and resulting in disparate brain injury. Even in patients with a similar common injuring force, injury to disparate brain structures and resulting symptoms is not unusual. This phenomenon is a limitation in previous studies that have associated PCS with brain biomarkers given that two patients diagnosed with PCS may have non-overlapping sets of symptoms. Some insights might be gained by studying symptom co-occurrence and the regional effects of injury.

Efforts to connect observations from neuroimaging and symptoms can be complicated by symptom co-occurrence. The results of the symptom co-occurrence analysis raise questions about using the diagnostic criteria for PCS, where three or more symptoms must co-occur for at least three months, in studies that seek potential biomarkers. In this study population, the most common symptoms occurred in isolation in large numbers. Patients who have only one persistent symptom would not meet the diagnostic criteria for PCS but they may still experience reduced quality of life following mTBI. The patterns of symptom co-occurrence in this study contrast with the patterns reported in a study of a subset of 108 patients in the CENTER-TBI study [39], where chronic cognitive and emotional domain symptoms were rarely observed without the other domain, and twice as many patients as expected had both cognitive- and emotional-domain symptoms. Notably, damage to limbic structures in patients with mTBI has been associated with the development and persistence of PTSD [40,41,42]. Patterns of symptom co-occurrence may be an avenue allowing for a better understanding of patient-specific neuronal damage and patient prognosis, in addition to the potential for better identification of malingering.

Regional effects of injury that affect the volumetrics of multiple ROI can also complicate efforts to connect neuroimaging and symptoms. For example, injury related to mTBI tends to affect not only the hippocampus but also its connectivity to other limbic regions [43,44]. Functionally, the hippocampus is primarily involved in learning, explicit memory, long-term potentiation, spatial navigation, and cortisol regulation [45]. Single or repeated experimental mTBIs cause lasting neuroinflammation associated with vascular disruption, neuronal cell death, and glial proliferation in experimental studies [46]. There was minimal evidence for regional effects of injury in this study. If there was substantial atrophy in several ROI after injury, or a particular vector of force was applied that set a process of asymmetric atrophy in motion, there should have been evidence in the form of stronger relationships among the ICV-adjusted ROI volume and asymmetry. Furthermore, excluding the lateral and medial OFC, ROI did not tend to be similar in associations with the presentation or longevity of symptoms. These observations do not agree with previous explorations of multifactorial effects of mTBI on the limbic system, with atrophy to one structure subsequently affecting functioning of neighboring regions [42,44]. It is possible that multi-region effects can be detected in diffusion or functional MRI studies but not using volumetrics in the time frame covered in this study.

Our findings build on prior work that used a subset of the civilian mTBI population used in this study, which reported that LOC was associated with the greater persistence of all symptoms except anxiety, age was associated with the persistence of balance problems, and MRI abnormalities were associated with anxiety and depression [26]. The sample size (and statistical power) was greater in this study, allowing the inclusion of additional covariates. LOC had the strongest associations with the presentation and longevity of symptoms. Unlike the previous study, age was related to both the presence and persistence of cognitive deficits and the presence of balance problems. This study and others [47] suggest that inclusion of covariates such as LOC and age is critical to accurately understand the relationships between brain volumetrics and mTBI symptoms, but incorporating covariates requires sample sizes that are historically rare in mTBI studies.

There are several past and ongoing efforts to understand why symptoms persist after TBI [48,49,50]. Further work is needed to contextualize our findings relevant to mechanistic explanations of brain response to injury. Symptoms presented by patients in this study could be understood based on impaired functioning as a direct result of an injury, or may in some cases be a consequence of normal activation in response to an abnormal (i.e., injured) neurological environment. A recent review highlighted the relevance of Active Interference Theory (AIT) and Defensive Activation Theory (DAT) as they apply to neurodegenerative disease, psychiatric problems, and phantom limb disease, but these models may also apply to the mTBI population.

AIT posits that expectations, resulting actions, and perceptions typically operate in a constantly updated loop in the normally functioning brain. From the AIT perspective, positive symptoms may arise when injury to brain networks interrupts the updating of expectations from sensory evidence, creating a mismatch between misguided predictions and observed outcomes. Positive symptoms result from the central nervous system’s active attempts to reconcile prediction and observed outcomes. Defensive activation theory (DAT) is the idea that there is competition for computational resources (cells) in the cerebral cortex of the brain. Although it is unclear if the DAT is relevant to the structures in the limbic system, it is worth considering that insights into the right–left balance of brain activation could be understood from this perspective. From the DAT perspective, disruption to networks that elicit appropriate activation in response to stimuli, or to networks that allow associated brain areas to assess the level of activation, may trigger abnormal activation.

Neuroinflammation can cause symptoms to persist after mTBIs. Immune system involvement in persistent symptoms post-mTBI in animal models and humans is reviewed in Verboon et al. [51]. When communication between the brain and the immune system is disrupted by injury, inflammation can result, and it has been suggested that long-term neurodegeneration could be initiated by injuries such as those causing mTBI. In brief, the injury creates a mechanical distortion which activates glial cells and damages the blood–brain barrier (BBB) and other areas where different types of tissue, such as gray matter and white matter, interface. Disrupted BBB allows fibrinogen deposition, which has been linked to white matter damage and cognitive deficits in animal models of neurodegenerative disease [52] and could presumably have a similar role in mTBI. Beyond the role of microglia, meningeal cell-mediated inflammation can induce anxiety in mouse models [53]. Evidence from human patients is still accumulating regarding immune involvement in symptoms of mTBI.

To date, studies report consistent presentation and continuation of some constellation of symptoms in all severities of TBI [49,50], even those with mild injury and minor or absent radiological findings. Below, associations between individual symptoms and limbic volumetrics are discussed by symptom due to the unique nature of findings and prior studies associated with each.

Some prior research demonstrates thalamic volume loss as a distinguishing characteristic for patients with mTBI presenting with headaches, while others report no significant thalamic volume loss in patients with mTBI with headaches versus those without [54,55]. Our results for the thalamus suggest one of two possible hypotheses: atrophy is a function of regional injury severity with proportional functional challenges [56], or pre-existing relatively large volumes confer a protective effect against headache in the setting of mTBI.

It is well-accepted that head trauma of varying severity is associated with volumetric decreases in both the thalamus and, although not observed in the current study, also the ACC [56,57,58]. Proposed mechanisms for these post-traumatic changes include Wallerian degeneration secondary to direct axonal trauma, decreased thalamic blood flow, and decreased functionality of interconnective neural pathways [59]. Loss of thalamic volume secondary to TBI appears to be proportional to the severity of the inciting injury [7]. Perhaps the strongest evidence in support of the previously mentioned trauma-driven neurodegenerative hypothesis is a 2009 study that investigated gray matter changes related to chronic post-traumatic headache [60]. Of the patients who developed chronic headaches post-injury, volume decreases in ACC and dorsolateral prefrontal cortex gray matter were observed between the fourteen-day and three-month imaging intervals; however, the one-year interval showed resolution of these structural abnormalities as well as the cessation of post-traumatic headache. While these results would seem to support the notion of a volume-based imaging biomarker for post-traumatic headache, further studies are needed to fully evaluate the significance of this relationship. On the other hand, to the best of our knowledge, no prior studies have sought to investigate a relationship between the volume of various limbic system structures and a hypothetical neurological protective effect in the setting of mTBI symptoms. There were multiple instances of an apparent neuroprotective effect or graded atrophy in association with symptom persistence in our results; distinguishing between the two hypotheses requires a different study design.

Persistent post-traumatic headache has been previously associated with decreased volumes in cortical and subcortical regions, including, similar to our findings, the OFC and thalamus [55,61,62]. Notably, Burrowes et al. [55] followed headache presence over time points up to 18 months post-injury, which more closely mirrors our study design as opposed to the single report of headache in the chronic phase (three or more months after injury).

Balance problems can lead to a decline and autonomy and overall health, yet neuroimaging biomarkers for persistent balance problems have received little research attention, and even less attention has been focused on balance problems after mTBI. The findings of this study are therefore novel, to the best of our knowledge. Surgent et al. [63] conducted meta-analyses summarizing 37 studies that associated balance with MRI findings in brain regions, linking volumetrics in several brain regions to static and dynamic balance. Smaller gray matter volumes within the basal ganglia [64,65] and thalamus were associated with poorer balance, while larger relative volumes were associated with exceptional balance. The basal ganglia and thalamus are involved in motor control and thus are regularly implicated in balance disorders. A previous study provides support of the positive relationship between basal ganglia volume and balance [63], and in this study, we observed that balance deficits persisted longer in participants with smaller lateral OFC and thalamus. A novel result in this study was that patients with higher asymmetry of the hippocampus experienced faster recovery from balance problems. Though it is unclear what advantage a more asymmetric hippocampus may have in the context of our study, Boisgontier et al. [66] hypothesized that greater brain volumes could potentially reduce postural stability via hyper-movement. If true, in the context of asymmetric contributions to stability, it is possible asymmetric regions are an adaptive response to postural instability.

Many patients report cognitive deficits following mTBI [67,68,69]. Persistent and pervasive post-concussive cognitive deficits are well-documented, from reports of significant impairment many years after injury [70] to long-term impairment resulting from a single TBI [5]. The cingulate gyrus has known cognitive functions with significant differences between controls and mTBI populations reported in diffusion tensor imaging studies in adults [71] and children [72,73]. The degree of cingulate gyrus post-traumatic atrophy has been related to injury severity in TBI subjects [74]. In this study, cognitive deficit persistence related only to side-independent asymmetry of the caudal ACC. While asymmetry has not been previously used to analyze patients with mTBI, volumetric studies frequently imply asymmetry is important by reporting an association between a symptom and atrophy on only one side of the brain. For example, in apparent agreement with our findings, atrophy in the right ACC (but not the left) has been correlated with performance on the Paced Auditory Serial Addition Test (PASAT), whereas atrophy in the left ACC (but not the right) correlated with performance on the California Verbal Learning Test (CVLT) [75]. Xue et al. reported associations between volume of the left ACC and presentation of cognitive symptoms [76]. Although we did not see an association between the volume of limbic structures and cognitive dysfunction, other studies have [77]. For example, verbal and declarative memory impairment, cognitive deficits, and psychological issues were associated with hippocampal atrophy or smaller hippocampal size bilaterally in patients with mTBI [78,79,80].

New or worsening fatigue is common following mTBI [81,82]. There was no evidence that fatigue was related to volumetrics of the limbic structures in this study. Previous findings on volumetric changes due to TBI-related fatigue are limited, with only one study showing reduced thalamic volume in patients with mTBI [83]. Another study, utilizing functional connectivity during an attention task as a proxy for fatigue, reported an association between chronic fatigue outcomes in patients with mTBI and altered striato–thalamic–cortical connectivity [83]. It is possible that the connectivity changes in question do not lead to volumetric changes in the time frame of this study. Moreover, self-reports of fatigue can encompass a variety of more specific symptoms, such as cognitive fatigue, central fatigue, and skeletal fatigue [84].

Based on the results of this study, anxiety is the symptom most closely associated with volumetric characteristics of the limbic system. Persistence of patient-reported anxiety was associated with the volume of four limbic structures and laterality of the entorhinal cortex. Other studies have found associations between limbic system volumes and anxiety, although not always in the same structures as observed in this study. Xue et al. [76] associated anxiety and depression with left hippocampal volume. In a study of 34 healthy people, higher levels of self-reported anxiety were negatively associated with the thickness of the right medial OFC [85], which supports the association between OFC volume and anxiety persistence in this study. However, the same study also found a positive association between anxiety and nucleus accumbens volume, which was not observed in this study. The discrepancy may be at least partly explained by the adjustment for TIV and inclusion of covariates in this study. Furthermore, although we observed no association between anxiety and cingulate volume, Zhou et al. found that atrophy in the left isthmus of the cingulate gyrus correlated with higher anxiety scores one year post-injury [75]. The observed associations between persistence of anxiety and laterality of the entorhinal cortex and small volumes of the parahippocampal gyrus and thalamus are novel, to the best of our knowledge.

Depression is three times more likely in those affected by mTBI compared to those who are not [86], and there has been considerable focus in the literature on the neurological structures correlated with depression, with or without injury. Some studies have found associations between depression after TBI and limbic ROI that we did not detect in this study, including the ACC [87,88], hippocampus, and the lateral OFC [87]. Medeiros et al. [89] noted convergence between neuroimaging findings in depression after TBI and those in non-TBI depression, demonstrated by reduced gray matter measures in the rostral anterior cingulate cortex, prefrontal cortex, and hippocampus in patients with non-injury related depression and depression after TBI. Volume loss in limbic regions has also been associated with idiopathic depression [90]. Notably, volumetric changes in the thalamus in the above studies aligned with observations in this study. More work is required to understand the clinical meaning of side-independent asymmetry of the isthmus of the cingulate relative to depression.

The results for emotional lability should be considered preliminary due to the small number of participants with this symptom and the small number of those who improved or recovered, providing little statistical power to detect associations between ROI volumes or asymmetry and emotional lability presentation or persistence. There is justification to pursue emotional lability with a larger sample, with particular attention toward distinguishing emotional lability from depression. A systematic review by Bryant et al. found that cortical volume changes in the prefrontal cortex and the OFC, as well as in the frontal and temporal lobes, were most likely to be associated with emotional control issues [91].

The results of this study suggest that insights may be gained by considering side-independent asymmetry, particularly in its association with persistence of balance problems, cognitive deficits, and depression, and in relation to the cingulate and the hippocampus. The laterality index in this paper was formulated to be directionally intuitive (large numbers indicate right bias) and to have a bounded scale between 1 and −1. Side-independent asymmetry was more closely related to symptom persistence than laterality. However, the best metrics to capture the mechanistic underpinnings of asymmetry or laterality remains an area for more study, as discussed above in the context of neuroinflammation, AIT, and DAT.

There are several future directions that would better elucidate the relationship between volumetric neuroimaging and the persistence of symptoms. For example, the implications of the same LI or siLI being achieved by hypertrophy of one side compared to the other, atrophy of one side compared to the other, or disproportionate atrophy or hypertrophy between sides, is not currently understood. For some ROI, symptom persistence was related to volume or asymmetry in a graduated way, not just for the patients with the most extreme volumes or asymmetries. This observation suggests either a gradation of structural atrophy resulting from mTBI or that the pre-mTBI volume or asymmetry of structures, after adjusting for TIV, may predispose a patient to a faster or slower recovery.

The patient population in this study was in litigation, which could bias the sample size by including patients with more significant mTBI. Some studies have described prolongation of self-reported symptoms in this population [4]. However, even non-litigant populations (e.g., TRACK-TBI) appear to experience long-term sequelae in substantial numbers [9], and the disadvantage of having participants in litigation is partially offset by the longitudinal tracking of symptoms and inclusion of many participants affected by mTBI who did and did not have symptoms [4]. The relatively low incidence of fatigue, anxiety, depression, and emotional lability provided less statistical power to detect associations with limbic brain regions, so results cannot be interpreted as having equal power to detect differences across symptoms. Moreover, animal models of hippocampal injury have demonstrated that force vector plays a significant role in sidedness of hippocampal atrophy, and force vector information was not available in this study. The handedness of participants was also potentially informative but not available.

A limitation of the siLI as formulated in this study is that there are no published reference values for a median LI for each ROI in a normal population, so the median of the study population of 524 patients with mTBI was used as the best available estimate. Although there is no indication that the LI should be systematically biased in this mTBI population, we cannot entirely exclude that possibility. A limitation of most mTBI studies is that it is difficult to conclude whether volumetric biomarkers associated with symptom persistence precede TBI or are a result of it. Both models may be important for clinical prognostication. In a study that attempted to address this issue by grouping patients as having either major depression, TBI, or both, the authors concluded smaller volumes in certain regions may contribute to susceptibility to developing depression post-mTBI [88]. The distinction may be important in effectively treating patients with mTBI.

## 5. Conclusions

We conclude that smaller overall volumes of the thalamus, orbitofrontal cortex, or parahippocampal gyrus post-mTBI are prognostic for longer symptom persistence. Post-mTBI asymmetry between the left and right hemisphere in the hippocampus, caudal anterior cingulate cortex, or isthmus of the cingulate cortex are also prognostic for longer symptom persistence. Total volume was associated with persistence of headache, balance problems, anxiety, and depression, whereas asymmetry was associated with persistence of balance problems, cognitive deficits, anxiety, and depression. From a clinical perspective, non-invasive brain stimulation (NIBS) has gained traction in recent years as a viable treatment option for patients suffering from the physical and neuropsychological sequelae of mTBI [92,93]. Studies have shown that treatment regimens combining NIBS with other rehabilitation methods (e.g., physical therapy, neurocognitive rehabilitation) hold promise in providing better long-term outcomes for patients with mTBI [94]. Further investigation is needed to better characterize the relationship between trauma-associated volume loss and any potential therapeutic benefit that may be provided with NIBS, particularly for sequelae that appear to be associated with asymmetry of brain structures [92].

## Figures and Tables

**Table 1:** Participant characteristics by sample size (N) and percent representation (%) for sample size and cause of injury; median and interquartile range for days between injury and first exam or scan; mean, standard deviation, and percent under 40 for age. *p*-values comparing no LOC and LOC groups are based on Fisher’s exact tests, Mann–Whitney tests, or *t*-tests.

	All	no LOC	LOC	*p*
Sample size	524	375 (72%)	149 (28%)	
Age (mean, SD, % under 40)	42 (14) 45.6%	43 (15) 44.3%	40 (13) 49.0%	0.026
Sex (F)	302 (58%)	234 (65%)	68 (48%)	<0.001
Days from injury to first exam (median, IQR)	69 (34, 132)	70 (35, 129)	66 (32, 139)	0.778
Days from injury to scan (median, IQR)	156 (56, 174)	102 (57, 173)	94(53, 190)	0.515
Diagnosed with PTSD	108 (21%)	68 (18%)	40 (27%)	0.031
Cause of injury				0.019
Assault	15 (3%)	10 (3%)	5 (3%)	
Falling	40 (8%)	20 (5%)	20 (13%)	
Motor vehicle accident	430 (82%)	315 (84%)	115 (77%)	
All other causes, or unknown	39 (7%)	30 (8%)	9 (6%)	

**Table 2:** Symptom diagnosis and resolution or improvement, for all participants and in sub-groups based on LOC at time of injury.

	Number (%) Diagnosed			Number (%) with Symptoms Improved or Resolved		
	All	No LOC	LOC	All	No LOC	LOC
Headache	503 96%	359 96%	144 97%	199 40%	152 42%	47 33%
Balance	375 72%	257 69%	118 79%	141 38%	109 42%	32 27%
Cognitive	405 77%	278 74%	127 85%	141 35%	107 38%	34 27%
Fatigue	118 23%	75 20%	43 29%	18 15%	15 20%	3 7%
Anxiety	180 34%	120 32%	60 40%	32 18%	23 19%	9 15%
Depression	153 29%	91 24%	62 42%	25 16%	16 18%	9 15%
Emotional Lability	90 17%	52 14%	38 26%	10 11%	5 10%	5 13%

**Table 3:** Summary of associations between covariates and limbic system metrics with symptom presence (upper section) and persistence (lower section). Coefficients from Table 4 and Table 5 with *p* < 0.05 are indicated by letters: covariates (X), volume (Vol), laterality index (LI), and side-independent laterality index (siLI). Presence or longer persistence of symptoms was associated with participants who had smaller volume, right-biased laterality, or greater asymmetry (except the isthmus of the cingulate gyrus). Sex and time between injury and scan had no significant associations and are not shown; *n* = 524.

Presence							
	Headache	Balance	Cognitive	Fatigue	Anxiety	Depression	Emot. Lab.
Age		X	X				
LOC		X	X	X	X	X	X
Thalamus	Vol						
**Persistence**							
Age			X				
LOC	X	X	X				
Amygdala							
Parahipp. Gyrus					Vol		
Hippocampus		siLI					
Entorh Cort.					LI		
Rostral ACC							
Caudal ACC			siLI				
PCC							
Isthmus Cing.						siLI	
Lateral OFC		Vol			Vol		
Medial OFC					Vol		
Ventral Dienc.							
Nucleus Accum.							
Thalamus	Vol	Vol			Vol	Vol	

**Table 4:** Symptom presentation relative to covariates, and adjusted odds of symptom presentation relative to ROI volume (LI and siLI in Table S6). Odds ratios and 95% confidence intervals are presented; bold indicates adjusted *p*-value < 0.05. Covariates from Lasso regression are indicated: A = age, S = sex, L = LOC, D = days between injury and scan, L* = interaction between L and volume, D* = interaction between D and volume. Reference condition for age was <40, for sex was female, and for LOC, none.

		Headache	Balance Problems	Cognitive Deficits	Fatigue	Anxiety	Depression	Emotional Lability
**Cov**	Age (<40)	0.79 0.32, 1.95	1.55 1.05, 2.28	1.51 1.00, 2.29	1.10 0.72, 1.66	1.10 0.76, 1.58	1.10 0.75, 1.62	1.28 0.80, 2.05
	Sex (F)	0.41 0.16, 1.02	0.68 0.46, 1.02	0.91 0.60, 1.40	1.22 0.80, 1.85	0.81 0.56, 1.18	0.92 0.62, 1.37	1.22 0.77, 1.95
	LOC (none)	1.48 0.52, 4.19	**1.91** **1.20, 3.04**	**2.11** **1.26, 3.53**	1.58 1.01, 2.45	1.50 1.00, 2.23	**2.28** **1.51, 3.44**	**2.09** **1.29, 3.36**
	DOI to DOS (z-score)	0.69 0.45, 1.05	0.88 0.72, 1.06	1.11 0.89, 1.37	1.00 0.81, 1.23	1.06 0.88, 1.27	1.12 0.92, 1.35	0.99 0.79, 1.25
**Vol**								
	Amygdala	0.92 0.59, 73.71 ASLD	0.84 0.54, 1.21 ASLD	0.77 0.31, 1.11 ASLD D*	0.78 0.60, 1.35 ASLD	0.88 0.73, 5.16 ASLD	0.86 0.69, 3.26 ASLD	0.89 0.22, 1.84 ASL
	Parahipp. Gyrus	0.90 0.61, 5.73 ASLD	0.96 0.81, 2.60 ASLD	0.85 0.03, 1.03 ASLD	0.91 0.74, 1.50 ASL	0.98 0.86, 6.83 ASLD	1.01 0.00, 1.08 ASLD	0.78 0.53, 1.03 ASLD
	Hippocampus	0.80 0.52, 1.98 ASLD	0.85 0.70, 1.15 ASLD	0.93 0.75, 1.82 ASLD	0.80 0.67, 4.77 ASLD	0.99 0.88, 429.77 ASLD	0.95 0.64, 2.24 ASLD	0.80 0.57, 1.10 ASLD
	Entorhinal cortex	1.04 0.00, 1.36 ASLD	0.89 0.73, 1.26 ASLD	0.78 0.64, 1.96 ASLD	1.00 0.00, 1.45 ASL	0.91 0.74, 1.35 ASLD	0.93 0.26, 1.53 ASLD	0.96 0.80, 6.29 ASLD
	Rostral ACC	1.30 0.58, 2.03 ASLD	0.92 0.76, 1.44 ASLD	0.83 0.64, 1.19 ASLD L*	0.89 0.70, 1.32 ASL	1.11 0.78, 1.33 ASLD	1.07 0.61, 1.29 ASLD	1.16 0.80, 1.46 ASLD
	Caudal ACC	0.69 0.00, 0.92 ASLD	0.94 0.78, 1.90 ASLD	0.85 0.67, 1.25 ASLD L*	0.82 0.66, 1.09 ASL	0.99 0.96, >100 ASLD	0.94 0.78, 1.81 ASLD	0.93 0.75, 2.21 ASLD
	PCC	1.04 0.00, 1.35 ASLD	1.27 0.97, 1.66 ASLD	1.26 0.92, 1.71 ASLD	1.02 0.11, 2.28 ASL	1.34 1.00, 1.64 ASLD	1.19 0.39, 1.43 ASLD	1.05 0.30, 1.43 ASLD
	Isthmus Cingulate	0.87 0.55, 3.26 ASLD	1.01 0.00, 1.05 ASLD	0.86 0.63, 1.18 ASLD	1.03 0.27, 1.24 ASL	1.13 0.82, 1.38 ASLD	0.92 0.72, 1.46 ASLD	0.75 0.59, 0.98 ASLD
	Lateral OFC	1.06 0.02, 3.15 ASLD	0.75 0.58, 0.95 ASLD	0.79 0.60, 1.04 ASLD	0.91 0.74, 1.48 SL	0.99 0.84, >100 ASLD	1.03 0.20, 1.22 ASLD	0.91 0.61, 1.78 ASLD
	Medial OFC	1.27 0.54, 23.1 ASLD	0.94 0.78, 2.00 ASLD	0.85 0.68, 1.20 ASLD	0.91 0.02, 1.30 ASL	1.05 0.40, 1.24 ASLD	1.11 0.73, 1.37 ASLD	0.96 0.77, 4.83 ASLD
	Ventral Diencephalon	1.18 0.32, 3.83 ASLD	0.87 0.71, 1.24 ASLD	1.03 0.18, 1.25 ASLD	0.85 0.69, 1.14 SL	0.81 0.67, 1.84 ASLD	0.82 0.69, 4.27 ASLD	0.85 0.61, 1.28 ASL
	Nucleus Accumbens	1.03 0.00, 3.91 ASLD	0.83 0.60, 1.16 ASLD	0.63 0.49, 1.39 ASLD	0.69 0.53, 1.17 ASLD	0.74 0.59, 1.00 ASLD	0.71 0.55, 0.96 ASLD	0.80 0.63, 1.08 SL
	Thalamus	**0.51** **0.32, 0.85** **ASLD**	1.04 0.35, 1.24 ASLD	0.94 0.68, 2.01 ASLD	1.16 0.80, 1.44 ASL	1.22 0.96, 1.48 ASLD	1.22 0.94, 1.49 ASLD	0.92 0.71, 1.87 ASLD

**Table 5:** Relationships between covariates, limbic volume, LI, siLI, and symptom persistence, including hazard ratios (HR), 95% confidence intervals, and raw *p*-values. Bold indicates *p*-values < 0.05 after Benjamini–Hochberg adjustment.

	Headache	Balance	Cognitive	Fatigue	Anxiety	Depression	Emotional Lability
Covariates							
Age (<40)	1.19 (0.89, 1.59)	1.30 (0.92, 1.83)	**1.57** **(1.11, 2.22)**	1.43 (0.48, 4.26)	1.46 (0.67, 3.19)	1.69 (0.67, 4.31)	0.54 (0, 282.13)
Sex (F)	1.22 (0.92, 1.62)	1.01 (0.72, 1.42)	0.97 (0.68, 1.39)	1.28 (0.46, 3.61)	1.30 (0.61, 2.76)	1.33 (0.55, 3.21)	2.50 (0.03,217.64)
LOC (none)	**1.46** **(1.05, 2.03)**	**1.70** **(1.13, 2.54)**	**1.49** **(1.02, 2.17)**	3.03 (0.08, 119.58)	1.38 (0.56, 3.39)	1.45 (0.61, 3.48)	0.68 (0.04, 11.94)
**Volume z-scores**							
Amygdala	1.06 (0.92, 1.23) 0.391	1.17 (0.98, 1.39) 0.076	0.98 (0.79, 1.22) 0.845	0.91 (0.48, 1.73) 0.777	1.08 (0.73, 1.58) 0.704	1.29 (0.84, 1.99) 0.247	0.75 (0.28, 2.05) 0.578
Parahippocampal Gyrus	1.12 (0.97, 1.30) 0.113	1.17 (0.98, 1.39) 0.077	0.98 (0.81, 1.17) 0.797	1.49 (0.75, 2.97) 0.259	**1.67** **(1.14, 2.44)** **0.008**	1.64 (1.06, 2.56) 0.028	1.99 (1.09, 3.62) 0.025
Hippocampus	1.14 (0.98, 1.32) 0.084	1.17 (0.98, 1.39) 0.082	1.09 (0.91, 1.30) 0.338	1.06 (0.67, 1.68) 0.811	1.66 (1.09, 2.53) 0.018	1.77 (1.08, 2.91) 0.023	1.27 (0.62, 2.58) 0.516
Entorhinal Cortex	1.14 (1.00, 1.30) 0.042	1.09 (0.92, 1.30) 0.328	1.14 (0.95, 1.36) 0.150	1.22 (0.81, 1.84) 0.345	1.06 (0.74, 1.54) 0.742	1.01 (0.65, 1.58) 0.954	1.02 (0.46, 2.27) 0.970
Rostral ACC	0.95 (0.82, 1.09) 0.437	0.90 (0.76, 1.06) 0.211	0.93 (0.78, 1.09) 0.362	0.97 (0.63, 1.47) 0.869	1.22 (0.84, 1.78) 0.297	1.58 (0.90, 2.77) 0.114	1.15 (0.51, 2.58) 0.738
Caudal ACC	0.96 (0.84, 1.10) 0.571	0.96 (0.81, 1.13) 0.586	1.03 (0.86, 1.24) 0.754	0.67 (0.44, 1.02) 0.062	1.15 (0.79, 1.68) 0.460	0.93 (0.62, 1.39) 0.728	1.27 (0.01, 223.67) 0.928
PCC	0.97 (0.85, 1.12) 0.711	0.89 (0.76, 1.05) 0.172	0.92 (0.76, 1.11) 0.386	0.59 (0.32, 1.08) 0.089	0.90 (0.61, 1.35) 0.620	0.89 (0.57, 1.39) 0.600	0.89 (0.39, 2.01) 0.773
Isthmus Cingulate	1.20 (1.04, 1.39) 0.014	1.07 (0.90, 1.27) 0.461	1.00 (0.83, 1.21) 0.995	0.92 (0.51, 1.67) 0.787	1.06 (0.70, 1.61) 0.791	0.91 (0.52, 1.61) 0.756	1.78 (0.77, 4.13) 0.178
Lateral OFC	1.19 (1.02, 1.39) 0.031	**1.33** **(1.11, 1.58)** **0.002**	1.04 (0.86, 1.25) 0.719	1.73 (1.07, 2.77) 0.024	**1.97** **(1.27, 3.06)** **0.002**	1.34 (0.81, 2.23) 0.260	1.54 (0.82, 2.90) 0.178
Medial OFC	1.16 (1.01, 1.34) 0.037	1.23 (1.04, 1.44) 0.013	1.05 (0.87, 1.27) 0.631	1.53 (0.94, 2.49) 0.090	**2.23** **(1.44, 3.45)** **<0.001**	1.64 (1.01, 2.65) 0.045	1.54 (0.92, 2.58) 0.099
Ventral Diencephalon	1.02 (0.89, 1.17) 0.771	1.06 (0.90, 1.26) 0.499	1.00 (0.83, 1.20) 0.983	0.92 (0.54, 1.57) 0.765	1.23 (0.85, 1.78) 0.265	1.25 (0.77, 2.02) 0.358	1.24 (0.52, 2.94) 0.623
Nucleus Accumbens	1.15 (1.00, 1.32) 0.051	1.20 (1.01, 1.42) 0.039	0.92 (0.74, 1.13) 0.420	1.73 (0.96, 3.10) 0.066	1.50 (1.05, 2.14) 0.026	1.33 (0.93, 1.90) 0.123	1.05 (0.51, 2.18) 0.899
Thalamus	**1.25** **(1.08, 1.44)** **0.002**	**1.28** **(1.07, 1.53)** **0.006**	1.17 (0.99, 1.38) 0.062	1.50 (0.83, 2.73) 0.181	**1.94** **(1.32, 2.85)** **0.001**	**1.79** **(1.21, 2.64)** **0.003**	1.75 (0.72, 4.24) 0.217
**LI**							
Amygdala	1.04 (0.79, 1.36) 0.788	1.05 (0.77, 1.43) 0.766	0.89 (0.66, 1.19) 0.426	0.96 (0.44, 2.12) 0.926	1.06 (0.55, 2.01) 0.871	0.90 (0.42, 1.92) 0.788	0.83 (0.18, 3.88) 0.811
Parahippocampal Gyrus	1.30 (1.00, 1.70) 0.049	1.19 (0.89, 1.60) 0.239	1.02 (0.75, 1.40) 0.894	2.51 (0.99, 6.40) 0.054	1.30 (0.70, 2.41) 0.411	1.20 (0.55, 2.58) 0.650	0.99 (0.19, 5.14) 0.990
Hippocampus	1.03 (0.69, 1.56) 0.876	1.26 (0.78, 2.02) 0.353	1.02 (0.65, 1.61) 0.917	1.55 (0.49, 4.94) 0.457	1.09 (0.33, 3.58) 0.888	1.92 (0.60, 6.14) 0.271	1.74 (0.20, 14.84) 0.613
Entorhinal Cortex	0.99 (0.86, 1.15) 0.912	0.91 (0.76, 1.09) 0.315	0.91 (0.78, 1.06) 0.234	0.78 (0.47, 1.30) 0.346	**0.52** **(0.33, 0.83)** **0.006**	0.50 (0.29, 0.87) 0.014	0.41 (0.08, 1.97) 0.264
Rostral ACC	1.03 (0.83, 1.28) 0.797	1.10 (0.82, 1.46) 0.530	0.90 (0.67, 1.22) 0.506	1.17 (0.49, 2.80) 0.717	1.47 (0.91, 2.38) 0.115	1.39 (0.70, 2.76) 0.347	1.10 (0.42, 2.83) 0.851
Caudal ACC	1.06 (0.93, 1.20) 0.409	1.07 (0.92, 1.23) 0.395	1.04 (0.88, 1.22) 0.651	1.42 (0.91, 2.21) 0.125	1.21 (0.90, 1.63) 0.215	1.29 (0.85, 1.96) 0.238	1.01 (0.50, 2.03) 0.982
PCC	1.01 (0.81, 1.25) 0.941	0.97 (0.74, 1.26) 0.796	0.97 (0.73, 1.28) 0.803	0.38 (0.16, 0.88) 0.025	0.56 (0.30, 1.03) 0.064	0.65 (0.31, 1.35) 0.244	0.69 (0.13, 3.70) 0.664
Isthmus Cingulate	1.07 (0.81, 1.41) 0.657	0.99 (0.70, 1.40) 0.940	1.00 (0.71, 1.41) 0.983	1.23 (0.32, 4.76) 0.761	0.98 (0.45, 2.14) 0.963	0.82 (0.28, 2.41) 0.719	2.46 (0.53, 11.54) 0.252
Lateral OFC	0.90 (0.66, 1.24) 0.532	0.73 (0.50, 1.06) 0.099	1.00 (0.70, 1.42) 0.985	0.85 (0.29, 2.52) 0.773	1.17 (0.54, 2.51) 0.691	1.04 (0.39, 2.78) 0.936	0.84 (0.10, 7.19) 0.870
Medial OFC	1.01 (0.68, 1.49) 0.965	0.94 (0.57, 1.56) 0.820	0.88 (0.53, 1.45) 0.603	2.58 (0.57, 11.74) 0.220	2.59 (0.83, 8.10) 0.101	1.51 (0.37, 6.09) 0.562	1.29 (0.05, 31.48) 0.878
Ventral Diencephalon	0.93 (0.68, 1.26) 0.625	1.00 (0.69, 1.46) 0.998	0.94 (0.64, 1.37) 0.734	1.22 (0.42, 3.53) 0.711	1.07 (0.48, 2.39) 0.877	1.31 (0.44, 3.86) 0.624	0.42 (0.06, 2.92) 0.379
Nucleus Accumbens	0.94 (0.75, 1.18) 0.614	0.85 (0.65, 1.10) 0.218	1.02 (0.77, 1.36) 0.873	0.67 (0.20, 2.29) 0.524	0.81 (0.45, 1.45) 0.479	0.72 (0.37, 1.43) 0.348	1.15 (0.25, 5.27) 0.860
Thalamus	0.93 (0.61, 1.41) 0.740	1.20 (0.74, 1.96) 0.465	1.13 (0.70, 1.81) 0.617	0.43 (0.07, 2.79) 0.374	0.40 (0.13, 1.22) 0.108	0.85 (0.22, 3.25) 0.809	1.41 (0.08, 24.38) 0.813
**siLI**							
Amygdala	0.75 (0.49, 1.17) 0.206	0.68 (0.40, 1.17) 0.167	0.78 (0.47, 1.31) 0.350	0.26 (0.04, 1.73) 0.163	0.41 (0.11, 1.55) 0.188	0.34 (0.07, 1.68) 0.184	1.03 (0.07, 14.19) 0.982
Parahippocampal Gyrus	0.99 (0.66, 1.50) 0.973	0.84 (0.51, 1.38) 0.485	1.03 (0.65, 1.64) 0.901	0.55 (0.14, 2.07) 0.375	0.62 (0.20, 1.93) 0.405	1.22 (0.36, 4.14) 0.751	1.68 (0.24, 11.76) 0.600
Hippocampus	0.51 (0.25, 1.06) 0.072	**0.27** **(0.11, 0.68)** **0.006**	0.37 (0.15, 0.92) 0.032	0.01 (0.00, 0.40) 0.015	0.57 (0.04, 7.33) 0.665	0.15 (0.01, 2.66) 0.194	0.25 (0.00, 21.32) 0.543
Entorhinal Cortex	0.97 (0.77, 1.22) 0.777	0.86 (0.66, 1.13) 0.274	0.88 (0.68, 1.15) 0.359	0.54 (0.22, 1.35) 0.190	0.84 (0.43, 1.63) 0.599	0.99 (0.48, 2.05) 0.981	1.12 (0.30, 4.19) 0.871
Rostral ACC	0.84 (0.57, 1.24) 0.381	0.66 (0.38, 1.16) 0.149	0.87 (0.53, 1.43) 0.578	0.85 (0.18, 4.03) 0.836	0.38 (0.13, 1.13) 0.082	0.58 (0.17, 2.03) 0.398	0.10 (0.00, 6.01) 0.270
Caudal ACC	0.78 (0.63, 0.98) 0.032	0.74 (0.56, 0.96) 0.025	**0.67** **(0.50, 0.90)** **0.008**	0.69 (0.28, 1.75) 0.438	0.62 (0.34, 1.13) 0.119	0.88 (0.43, 1.80) 0.733	1.16 (0.03, 40.25) 0.936
PCC	0.67 (0.43, 1.06) 0.087	0.64 (0.38, 1.09) 0.103	0.48 (0.27, 0.85) 0.012	1.68 (0.38, 7.38) 0.490	0.64 (0.18, 2.30) 0.499	0.58 (0.08, 4.26) 0.594	1.33 (0.07, 24.08) 0.847
Isthmus Cingulate	1.04 (0.65, 1.67) 0.866	1.04 (0.60, 1.82) 0.883	1.38 (0.82, 2.34) 0.224	2.14 (0.44, 10.38) 0.345	1.60 (0.53, 4.89) 0.407	**5.39** **(1.63, 17.78)** **0.006**	1.03 (0.08, 12.98) 0.983
Lateral OFC	1.09 (0.64, 1.86) 0.752	0.62 (0.30, 1.27) 0.190	0.73 (0.37, 1.46) 0.378	0.66 (0.12, 3.64) 0.633	0.28 (0.05, 1.49) 0.135	0.40 (0.05, 3.03) 0.376	0.31 (0.01, 16.38) 0.560
Medial OFC	1.01 (0.51, 2.00) 0.971	0.86 (0.35, 2.13) 0.749	1.24 (0.56, 2.74) 0.596	0.92 (0.09, 9.61) 0.947	1.30 (0.19, 9.04) 0.789	1.23 (0.12, 12.46) 0.862	3.20 (0.04, 292.33) 0.613
Ventral Diencephalon	1.14 (0.74, 1.76) 0.550	0.84 (0.47, 1.51) 0.563	1.35 (0.85, 2.14) 0.209	0.49 (0.09, 2.57) 0.396	0.40 (0.08, 2.06) 0.271	1.18 (0.28, 4.92) 0.817	1.46 (0.24, 8.97) 0.682
Nucleus Accumbens	1.13 (0.78, 1.62) 0.526	1.51 (1.02, 2.23) 0.040	1.16 (0.75, 1.78) 0.510	1.84 (0.47, 7.25) 0.383	0.98 (0.31, 3.16) 0.979	1.55 (0.67, 3.60) 0.309	0.69 (0.03, 17.92) 0.822
Thalamus	0.86 (0.41, 1.80) 0.686	0.87 (0.39, 1.92) 0.729	1.02 (0.48, 2.16) 0.957	1.18 (0.05, 30.33) 0.922	1.78 (0.40, 7.89) 0.450	1.69 (0.25, 11.57) 0.595	6.00 (0.24, 149.37) 0.275

## Data Availability

The original data presented in the study are openly available in FigShare at 10.6084/m9.figshare.26308042.

## Supplementary Materials

The following supporting information can be downloaded at: https://www.mdpi.com/article/10.3390/jcm13175154/s1, Figure S1: Limbic volume Kaplan–Meier plots; Figure S2: Limbic LI Kaplan–Meier plots; Figure S3: Limbic asymmetry (siLI) Kaplan–Meier plots; Table S1: Symptom co-incidence summary; Table S2: Summary of TIV-adjusted volumes for left- and right-hemisphere limbic structures; Table S3: Summary statistics for limbic system regional volumes, LI, and siLI; Table S4: Correlations among volumes, LI, and siLI of limbic structures; Table S5: Summary statistics for covariates and limbic system volumes, LI, and siLI, grouped by symptom presence or absence; Table S6: Adjusted odds of symptom presentation relative to LI and siLI; Table S7: Cut points for quartiles in Figures S2 and S3.
