# Supplementary material for: Symptom Persistence Relates to Volume and Asymmetry of the Limbic System after Mild Traumatic Brain Injury
Source: J Clin Med. 2024 Aug 30;13(17):5154. doi: 10.3390/jcm13175154 (PMC11396354; doi:10.3390/jcm13175154)
Supplement: Supplementary file 1 [file jcm-13-05154-s001.zip › Figure S1 Limbic volume clinical symptom graphs.pdf]

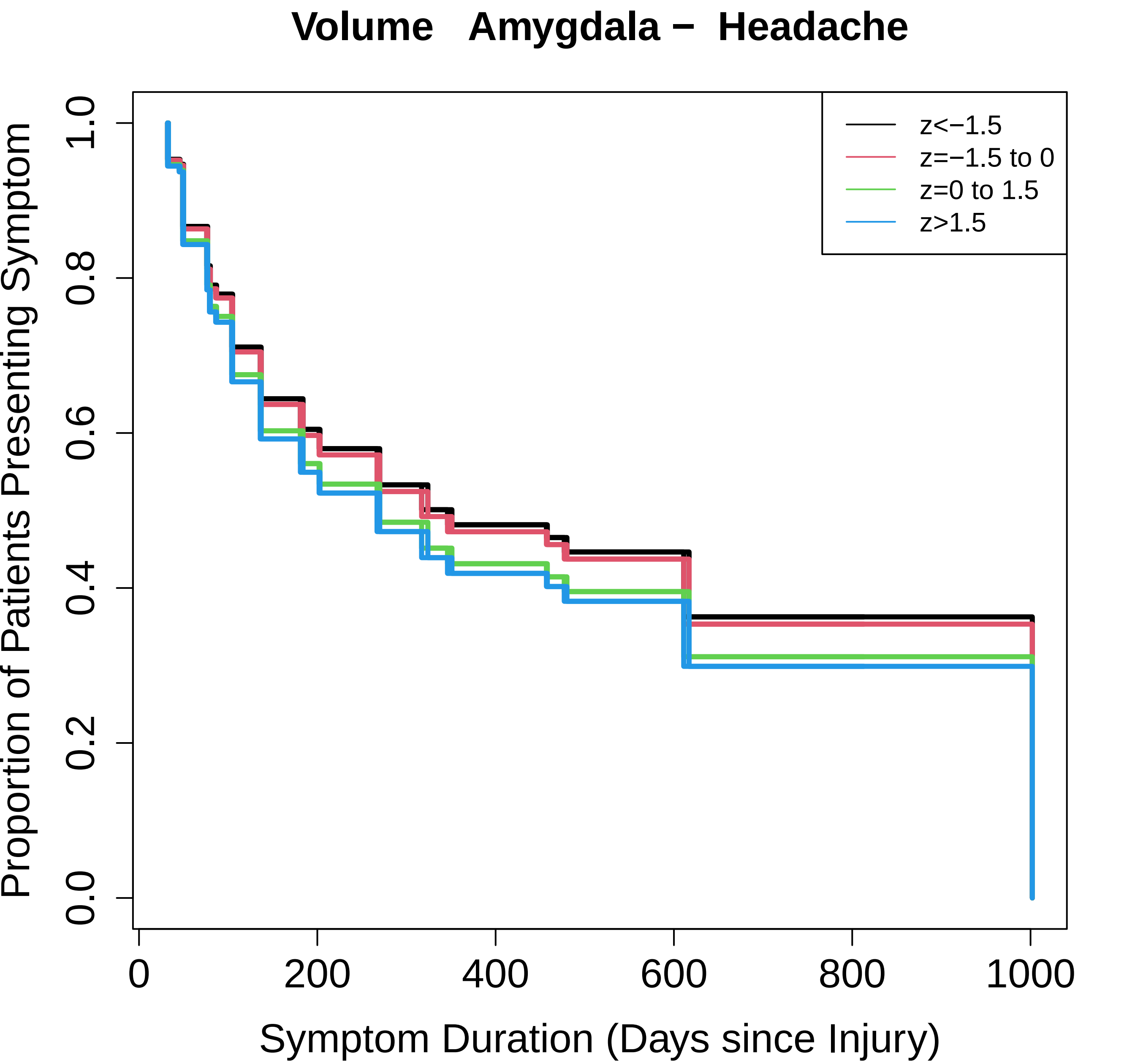
Volume Amygdala − Headache
Proportion of Patients Presenting Symptom
0.0
0.2
0.4
0.6
0.8
1.0
0
200
400
600
800
1000
Symptom Duration (Days since Injury)
z<−1.5
z=−1.5 to 0
z=0 to 1.5
z>1.5

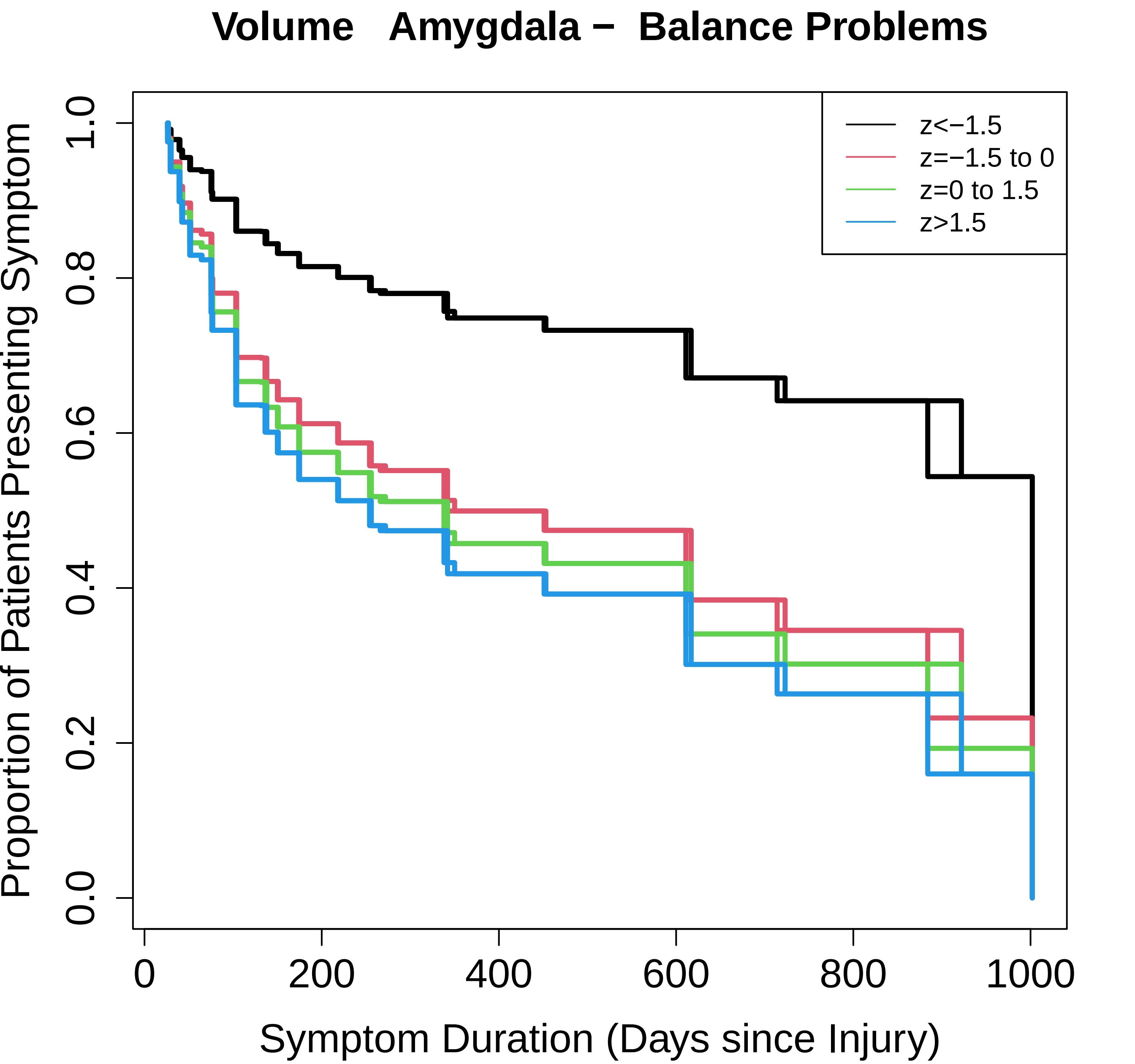

Volume Amygdala − Balance Problems
Proportion of Patients Presenting Symptom
Symptom Duration (Days since Injury)
z<−1.5
z=−1.5 to 0
z=0 to 1.5
z>1.5
0.0
0.2
0.4
0.6
0.8
1.0
0
200
400
600
800
1000

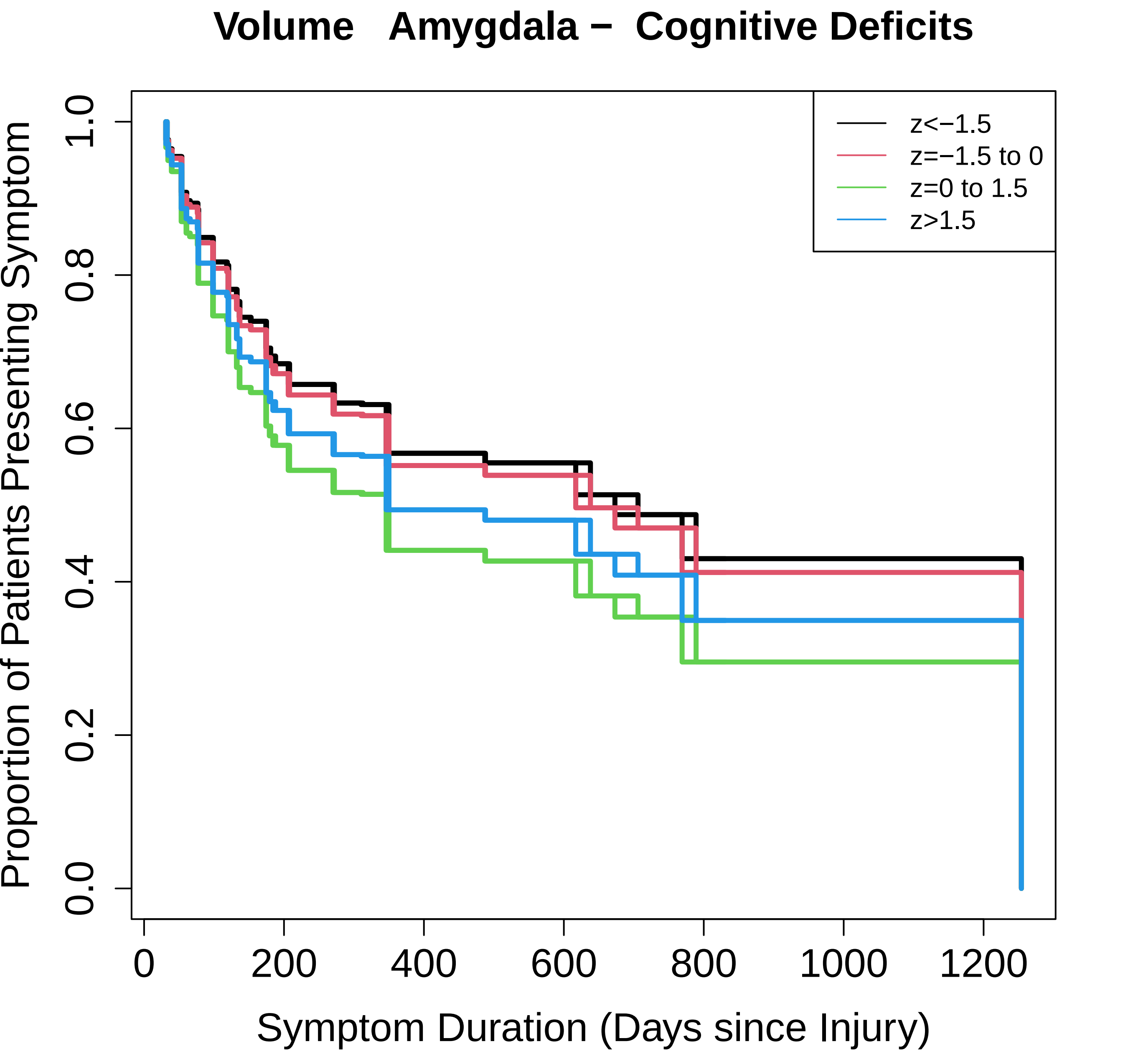
Volume Amygdala − Cognitive Deficits
z<−1.5
z=−1.5 to 0
z=0 to 1.5
z>1.5
Proportion of Patients Presenting Symptom
0.0
0.2
0.4
0.6
0.8
1.0
0
200
400
600
800
1000
1200
Symptom Duration (Days since Injury)

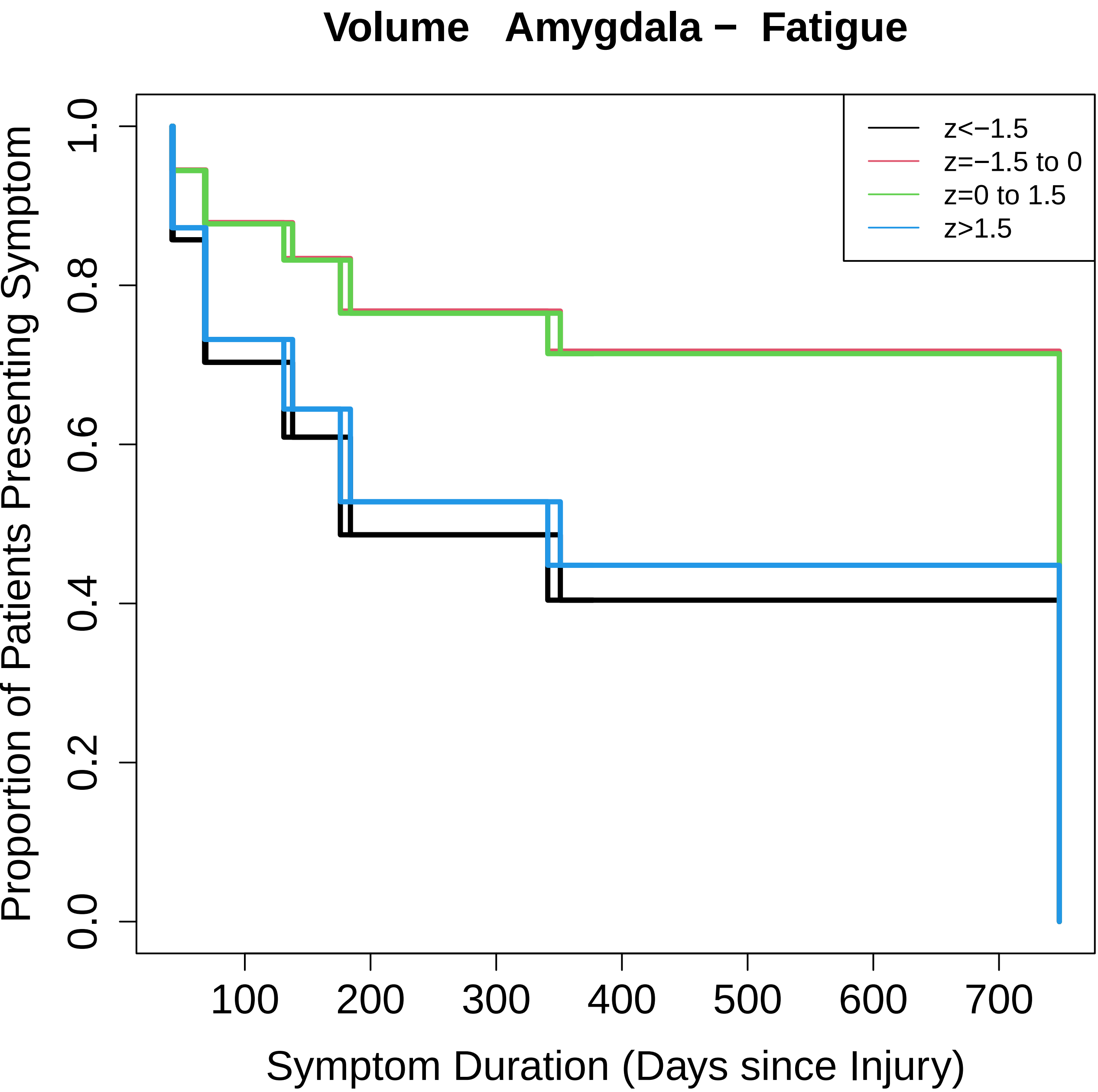

Volume Amygdala − Fatigue
Proportion of Patients Presenting Symptom
Symptom Duration (Days since Injury)
0.0
0.2
0.4
0.6
0.8
1.0
100
200
300
400
500
600
700
z<−1.5
z=−1.5 to 0
z=0 to 1.5
z>1.5

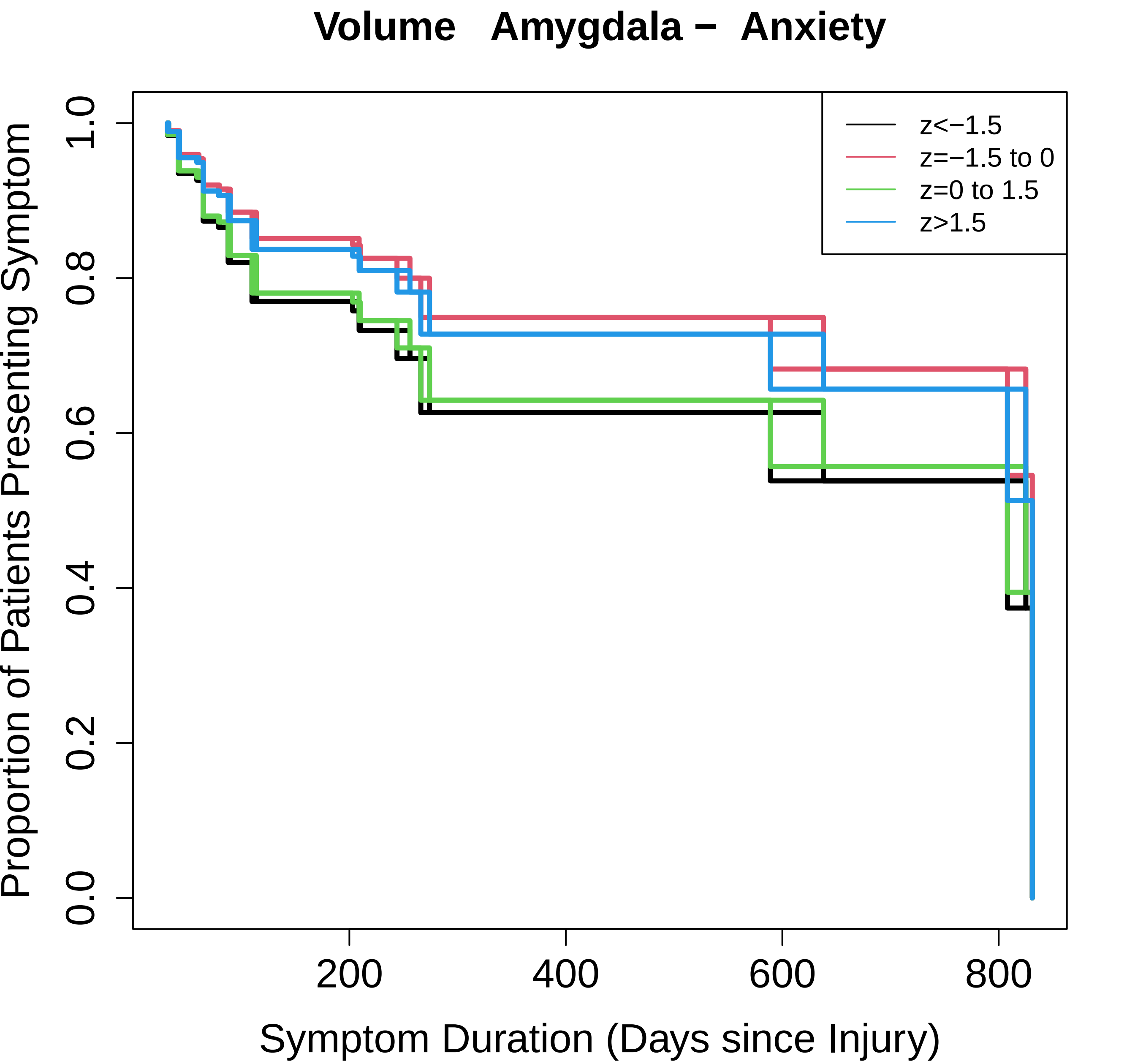
Volume Amygdala − Anxiety
Proportion of Patients Presenting Symptom
Symptom Duration (Days since Injury)
0.0
0.2
0.4
0.6
0.8
1.0
200
400
600
800
z<−1.5
z=−1.5 to 0
z=0 to 1.5
z>1.5

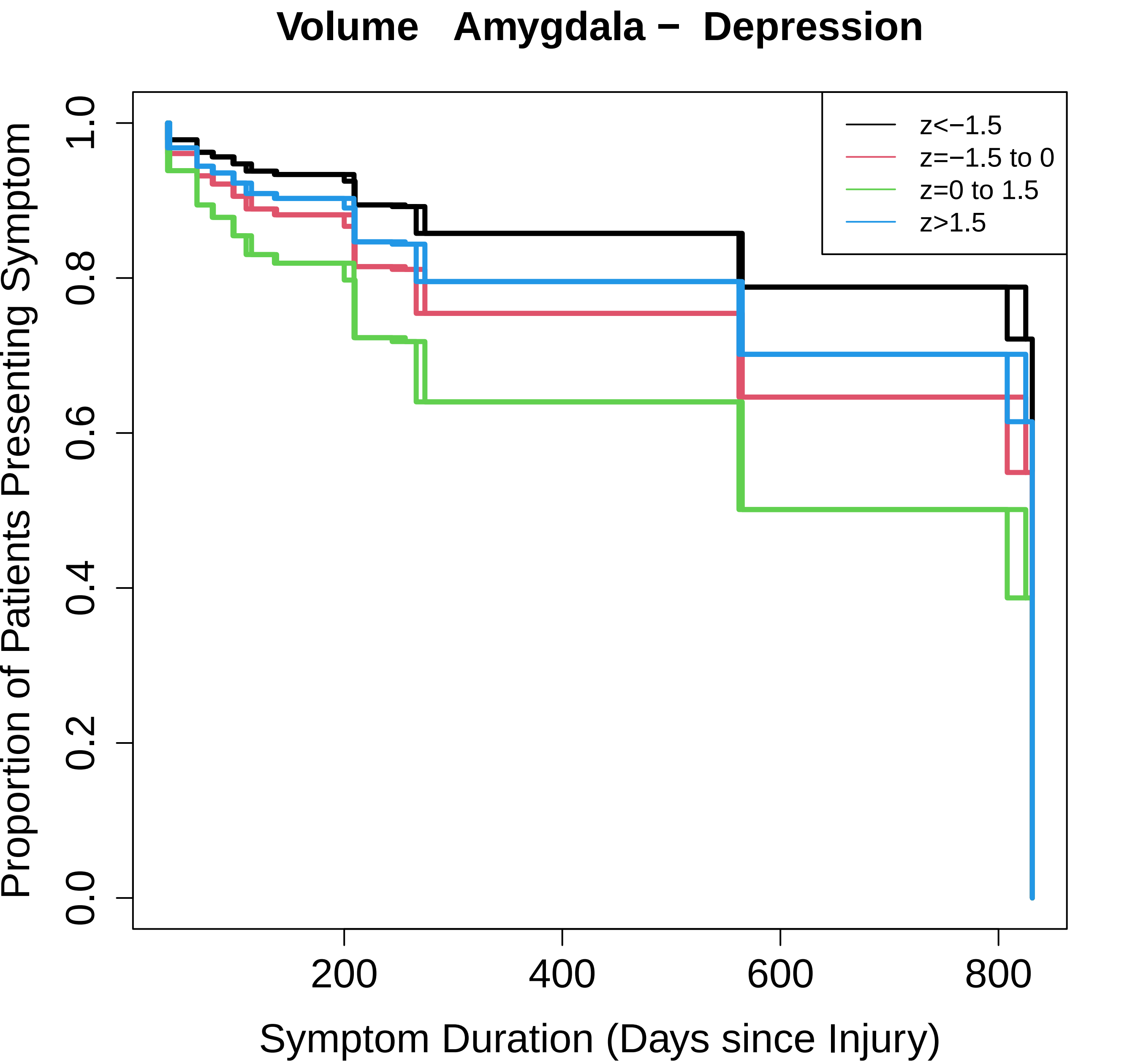
Volume Amygdala − Depression
Proportion of Patients Presenting Symptom
Symptom Duration (Days since Injury)
0.0
0.2
0.4
0.6
0.8
1.0
200
400
600
800
z<−1.5
z=−1.5 to 0
z=0 to 1.5
z>1.5

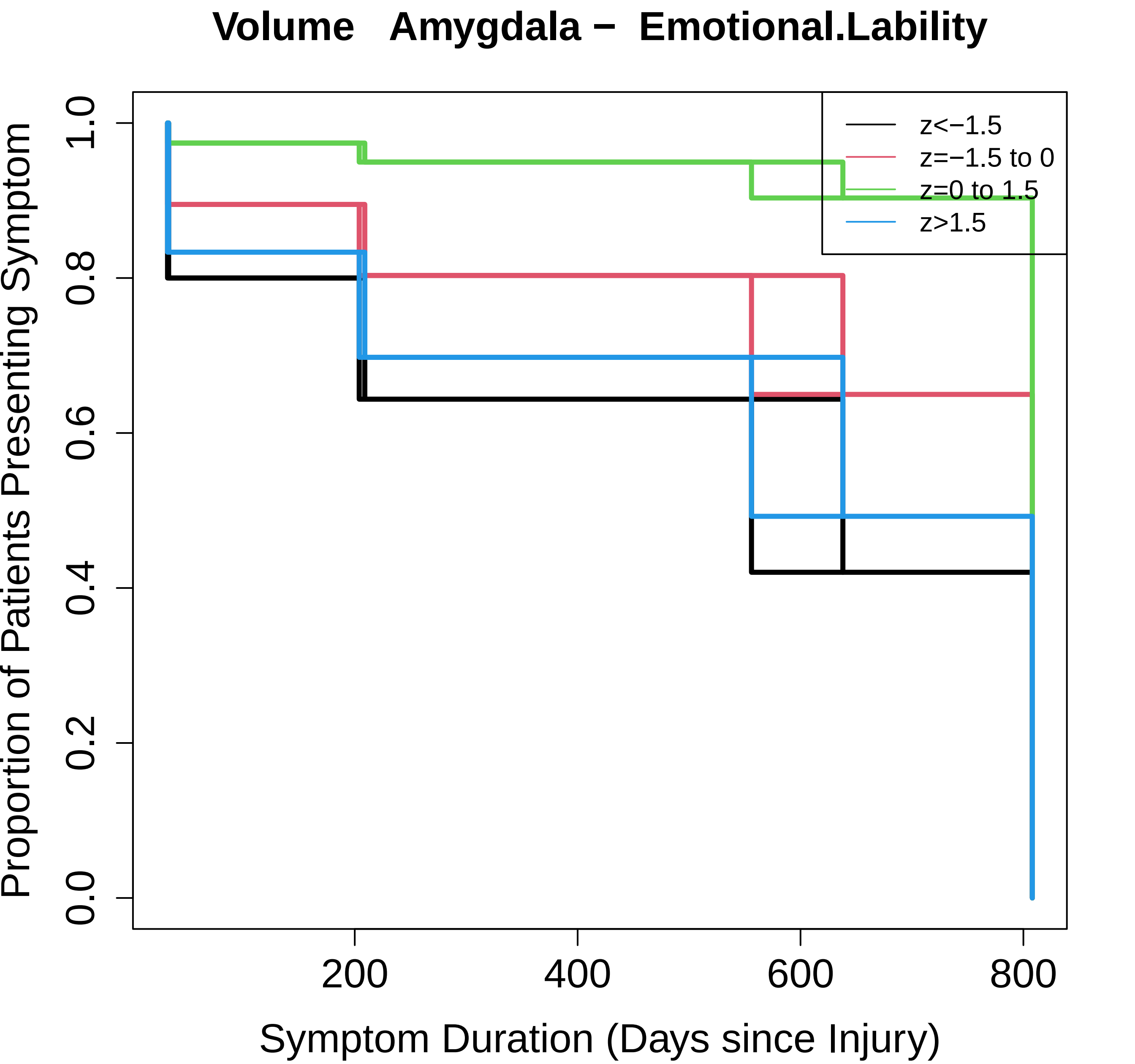

Volume Amygdala − Emotional.Lability
Proportion of Patients Presenting Symptom
0.0
0.2
0.4
0.6
0.8
1.0
200
400
600
800
Symptom Duration (Days since Injury)
z<−1.5
z=−1.5 to 0
z=0 to 1.5
z>1.5

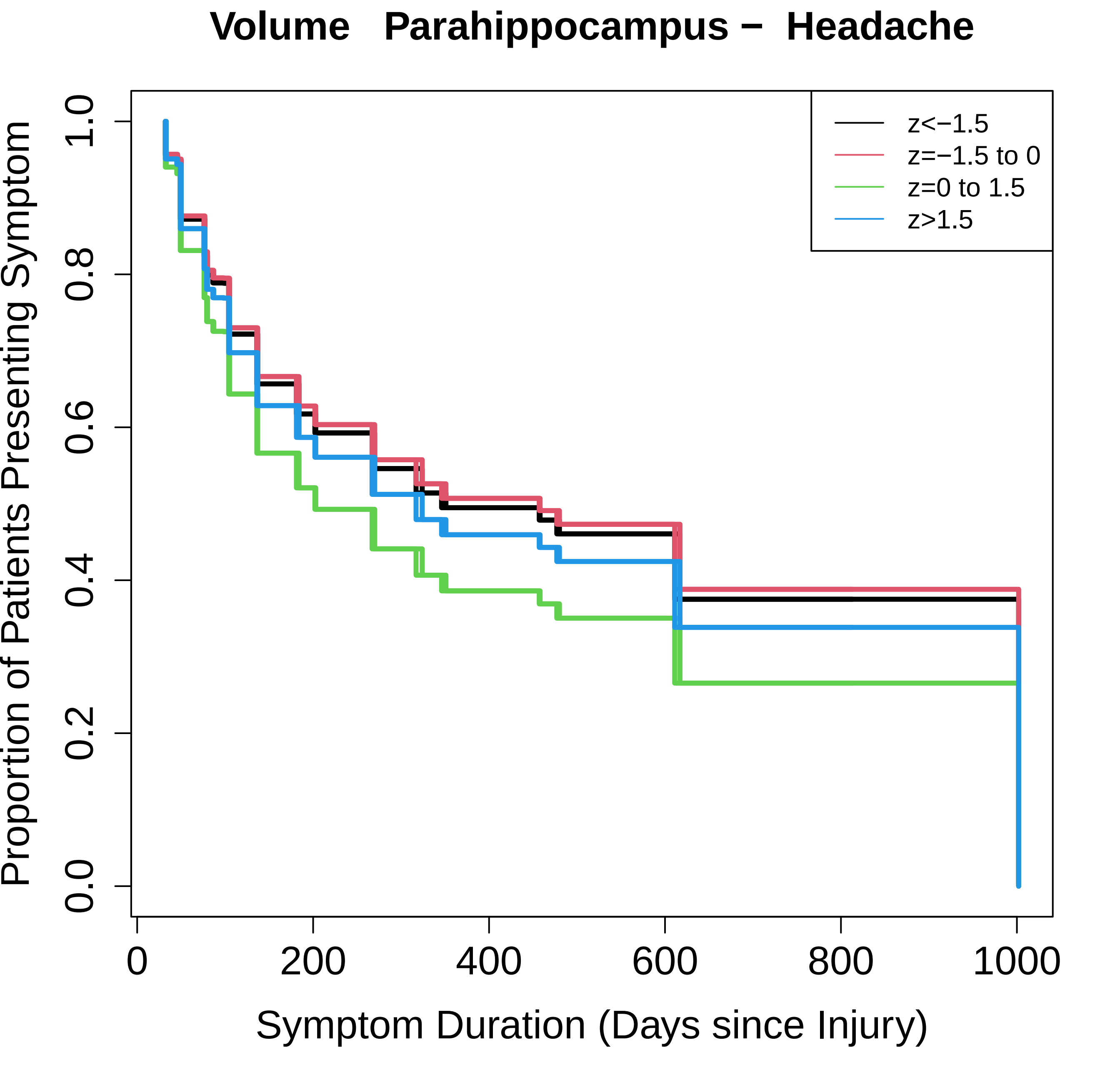

Volume Parahippocampus − Headache
Proportion of Patients Presenting Symptom
0.0
0.2
0.4
0.6
0.8
1.0
0
200
400
600
800
1000
Symptom Duration (Days since Injury)
z<−1.5
z=−1.5 to 0
z=0 to 1.5
z>1.5

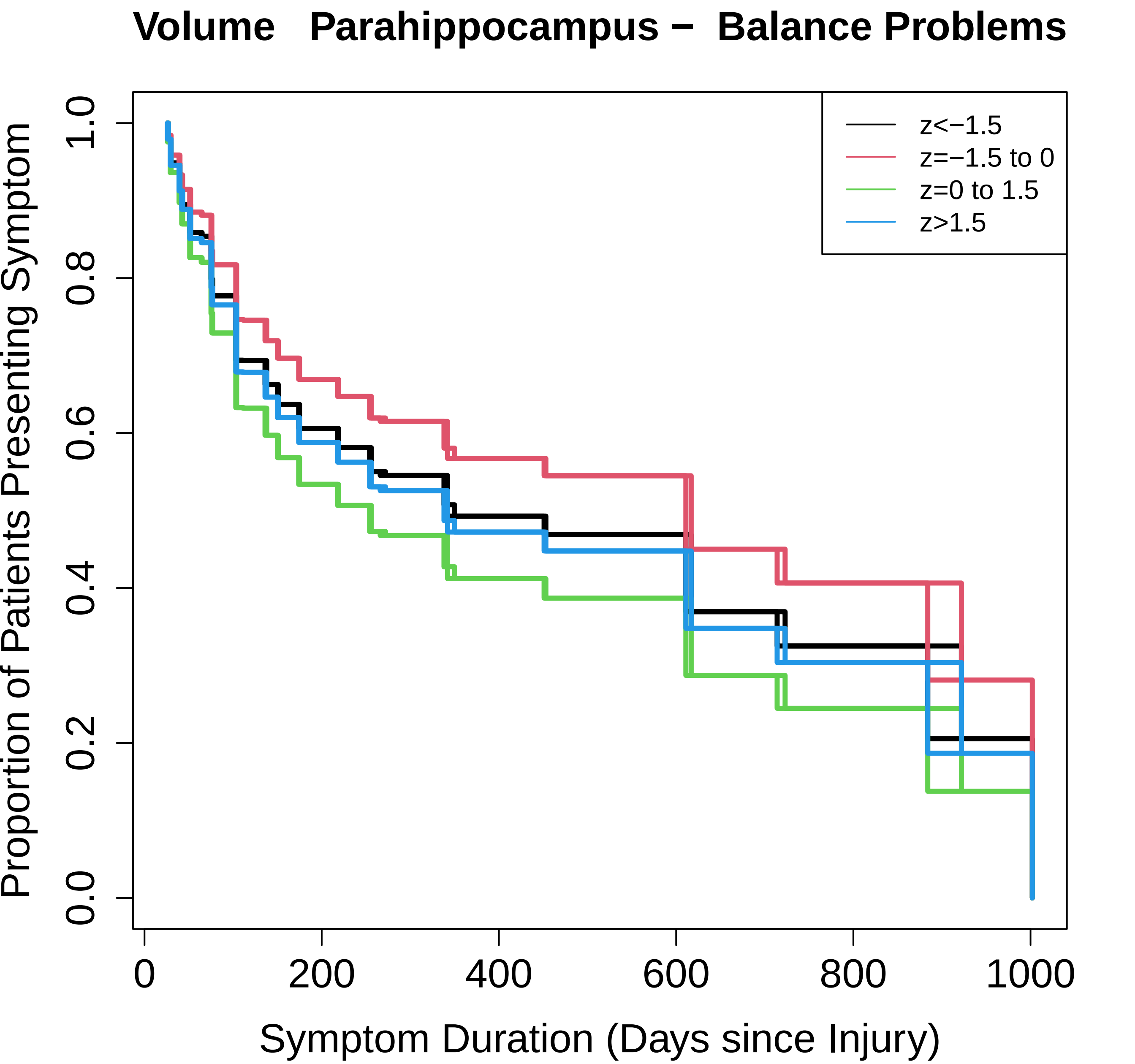
Volume Parahippocampus − Balance Problems
z<−1.5
z=−1.5 to 0
z=0 to 1.5
z>1.5
Proportion of Patients Presenting Symptom
0.0
0.2
0.4
0.6
0.8
1.0
0
200
400
600
800
1000
Symptom Duration (Days since Injury)

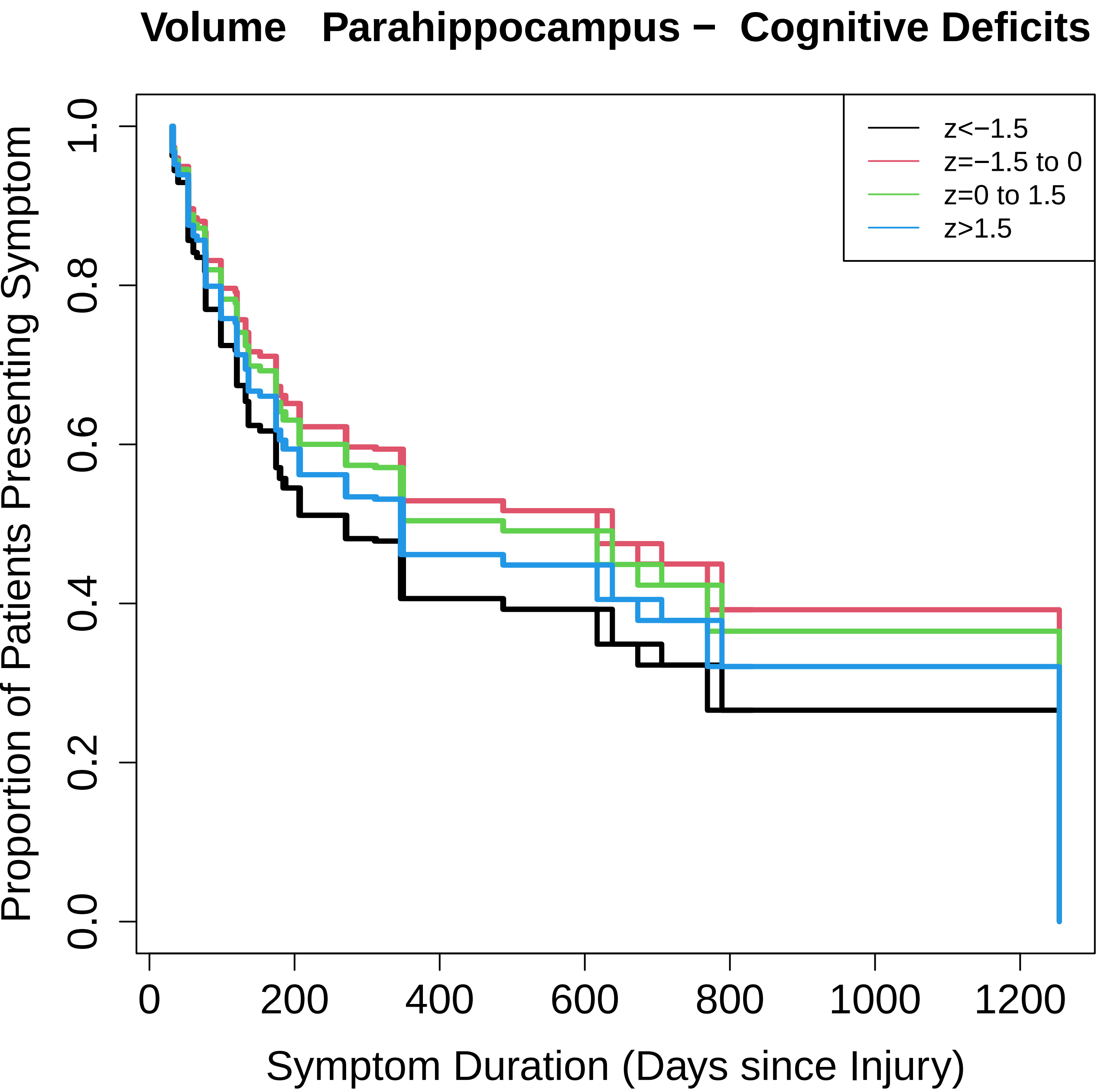

Volume Parahippocampus − Cognitive Deficits
Proportion of Patients Presenting Symptom
0.0
0.2
0.4
0.6
0.8
1.0
0
200
400
600
800
1000
1200
Symptom Duration (Days since Injury)
z<−1.5
z=−1.5 to 0
z=0 to 1.5
z>1.5

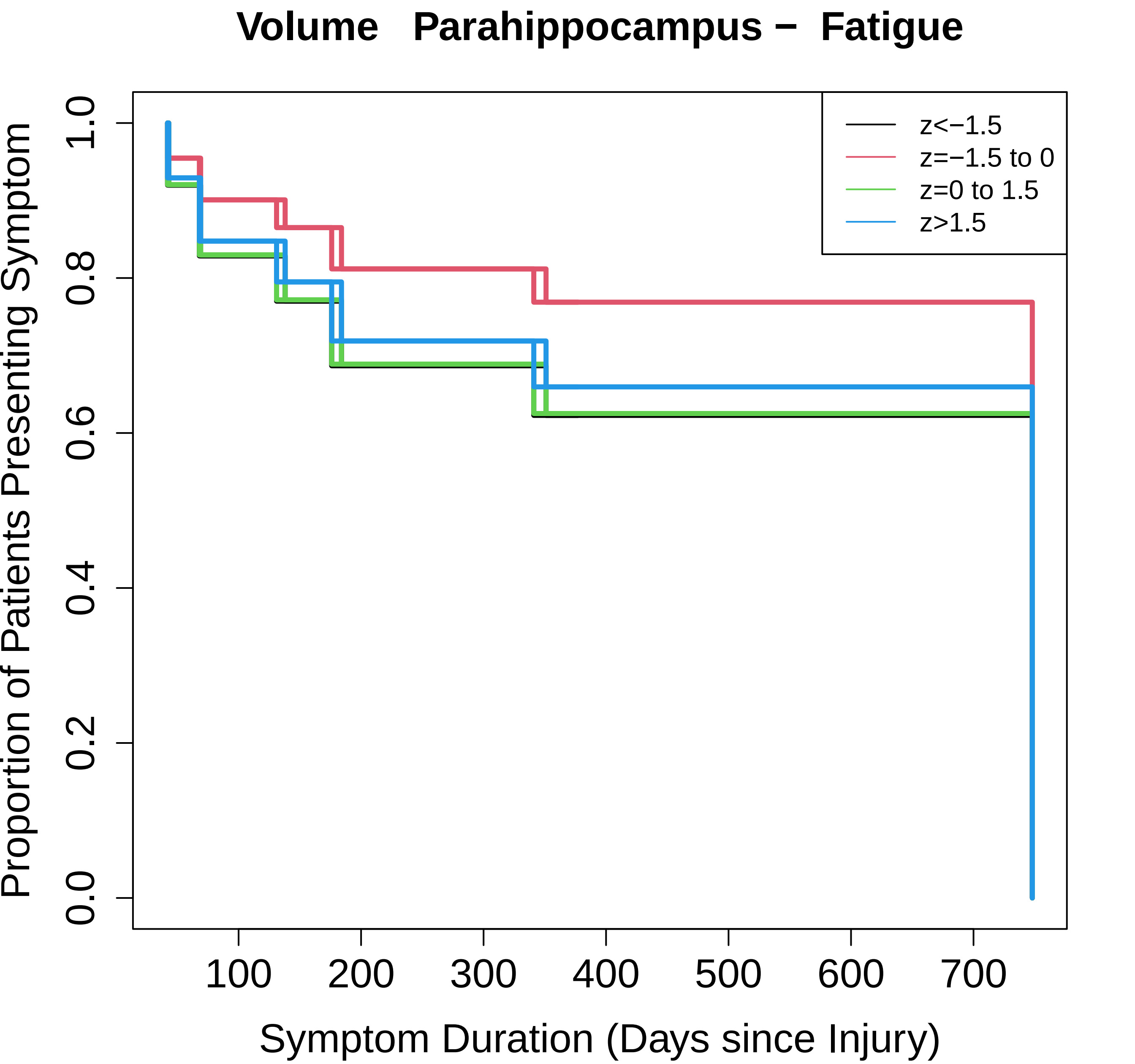

Volume Parahippocampus − Fatigue
z<−1.5
z=−1.5 to 0
z=0 to 1.5
z>1.5
Proportion of Patients Presenting Symptom
0.0
0.2
0.4
0.6
0.8
1.0
100
200
300
400
500
600
700
Symptom Duration (Days since Injury)

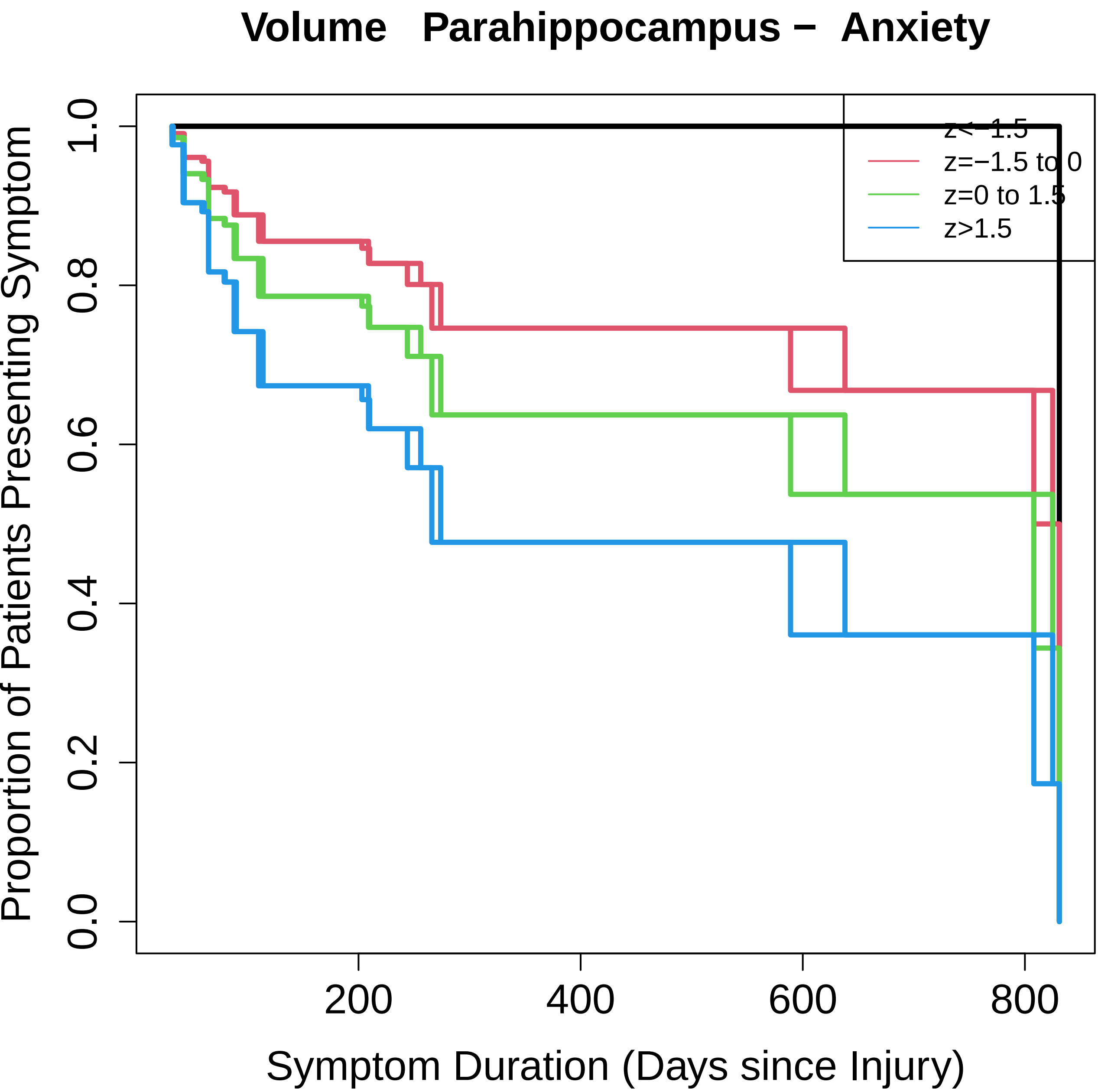
Volume Parahippocampus − Anxiety
Proportion of Patients Presenting Symptom
Symptom Duration (Days since Injury)
z<−1.5
z=−1.5 to 0
z=0 to 1.5
z>1.5
0.0
0.2
0.4
0.6
0.8
1.0
200
400
600
800

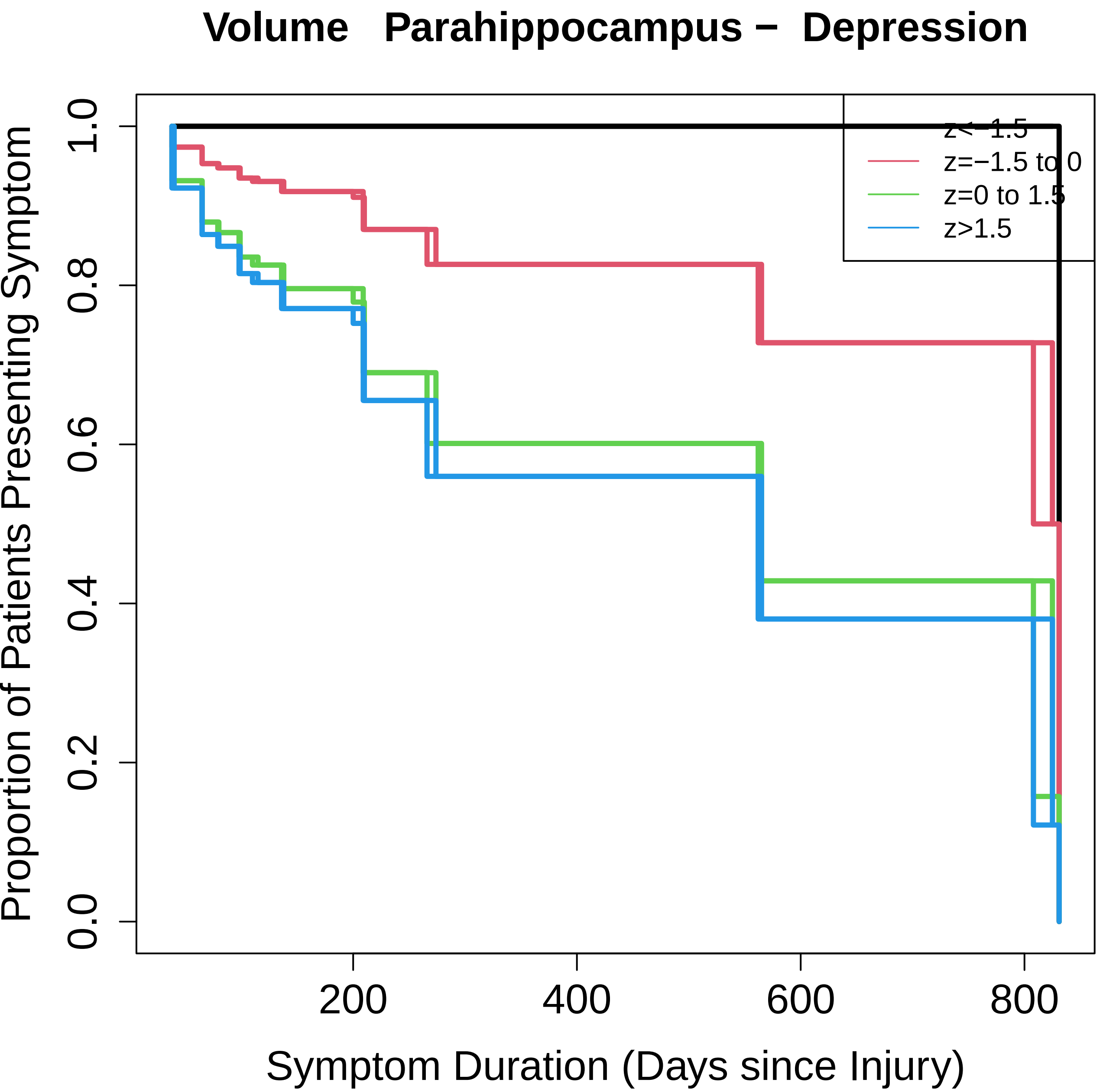

Volume Parahippocampus − Depression
z<−1.5
z=−1.5 to 0
z=0 to 1.5
z>1.5
Proportion of Patients Presenting Symptom
0.0
0.2
0.4
0.6
0.8
1.0
200
400
600
800
Symptom Duration (Days since Injury)

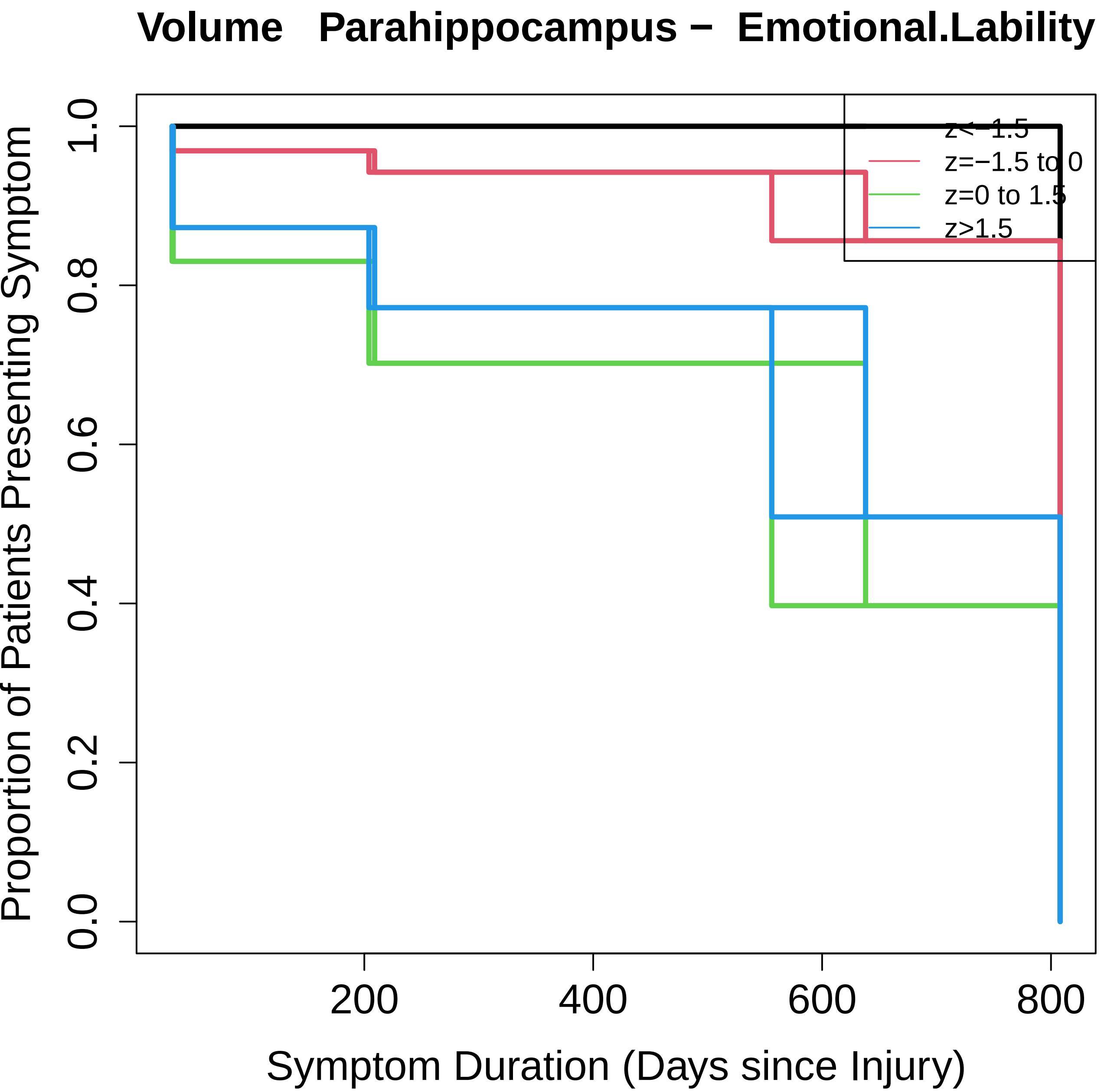
Volume Parahippocampus − Emotional.Lability
Proportion of Patients Presenting Symptom
0.0
0.2
0.4
0.6
0.8
1.0
200
400
600
800
Symptom Duration (Days since Injury)
z<−1.5
z=−1.5 to 0
z=0 to 1.5
z>1.5

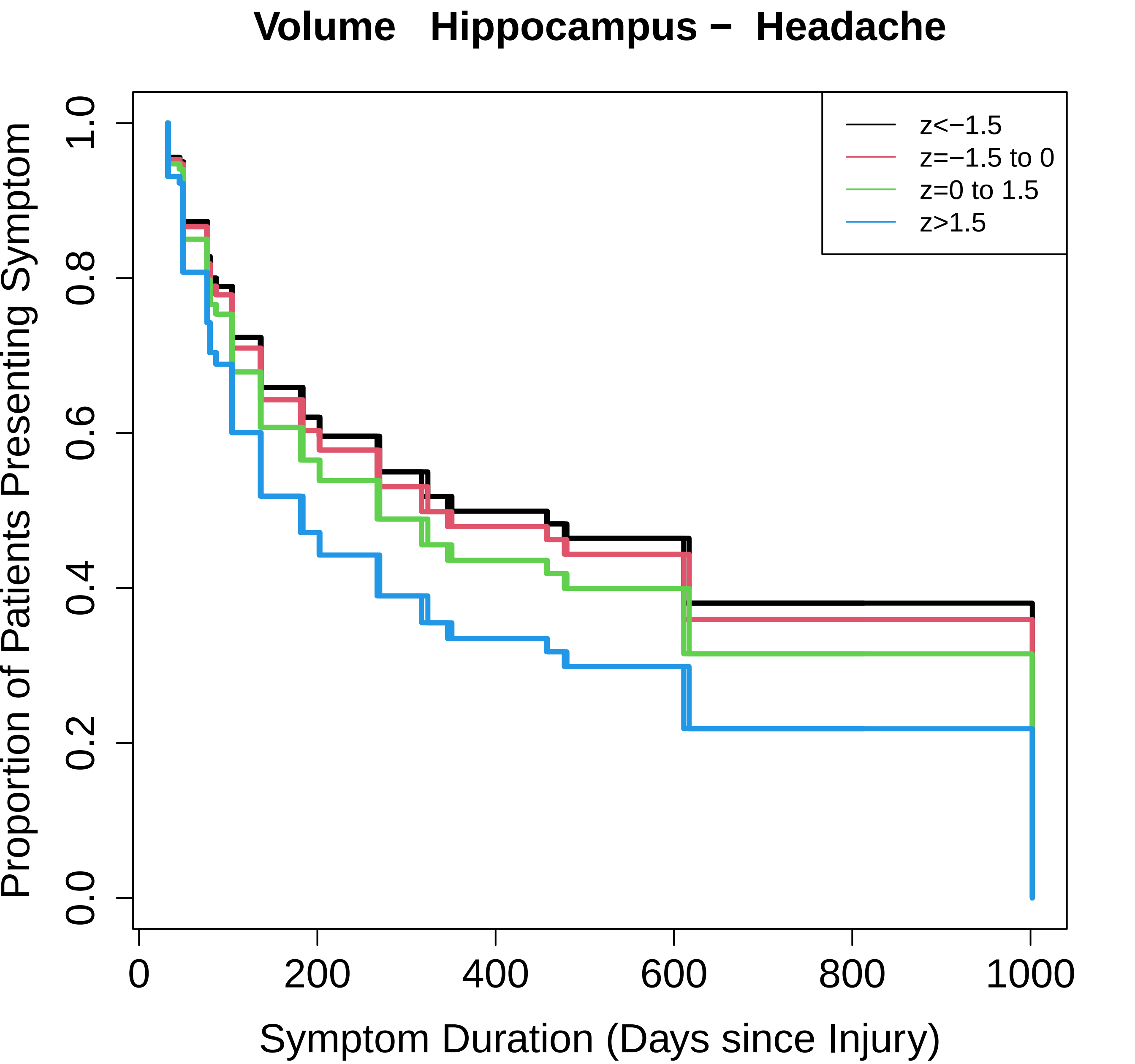

Volume Hippocampus − Headache
Proportion of Patients Presenting Symptom
Symptom Duration (Days since Injury)
0.0
0.2
0.4
0.6
0.8
1.0
0
200
400
600
800
1000
z<−1.5
z=−1.5 to 0
z=0 to 1.5
z>1.5

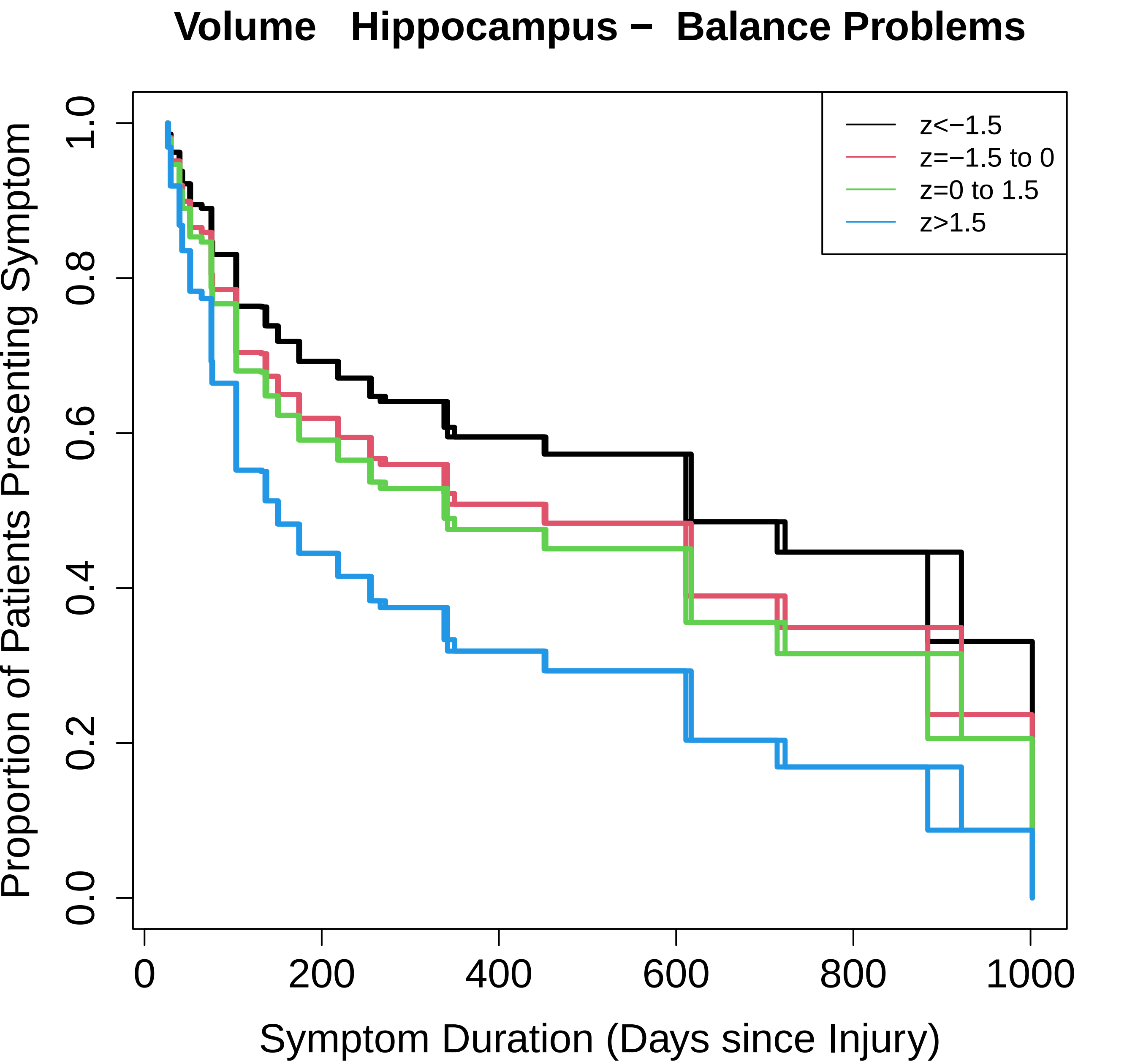
Volume Hippocampus − Balance Problems
Proportion of Patients Presenting Symptom
Symptom Duration (Days since Injury)
0.0
0.2
0.4
0.6
0.8
1.0
0
200
400
600
800
1000
z<−1.5
z=−1.5 to 0
z=0 to 1.5
z>1.5

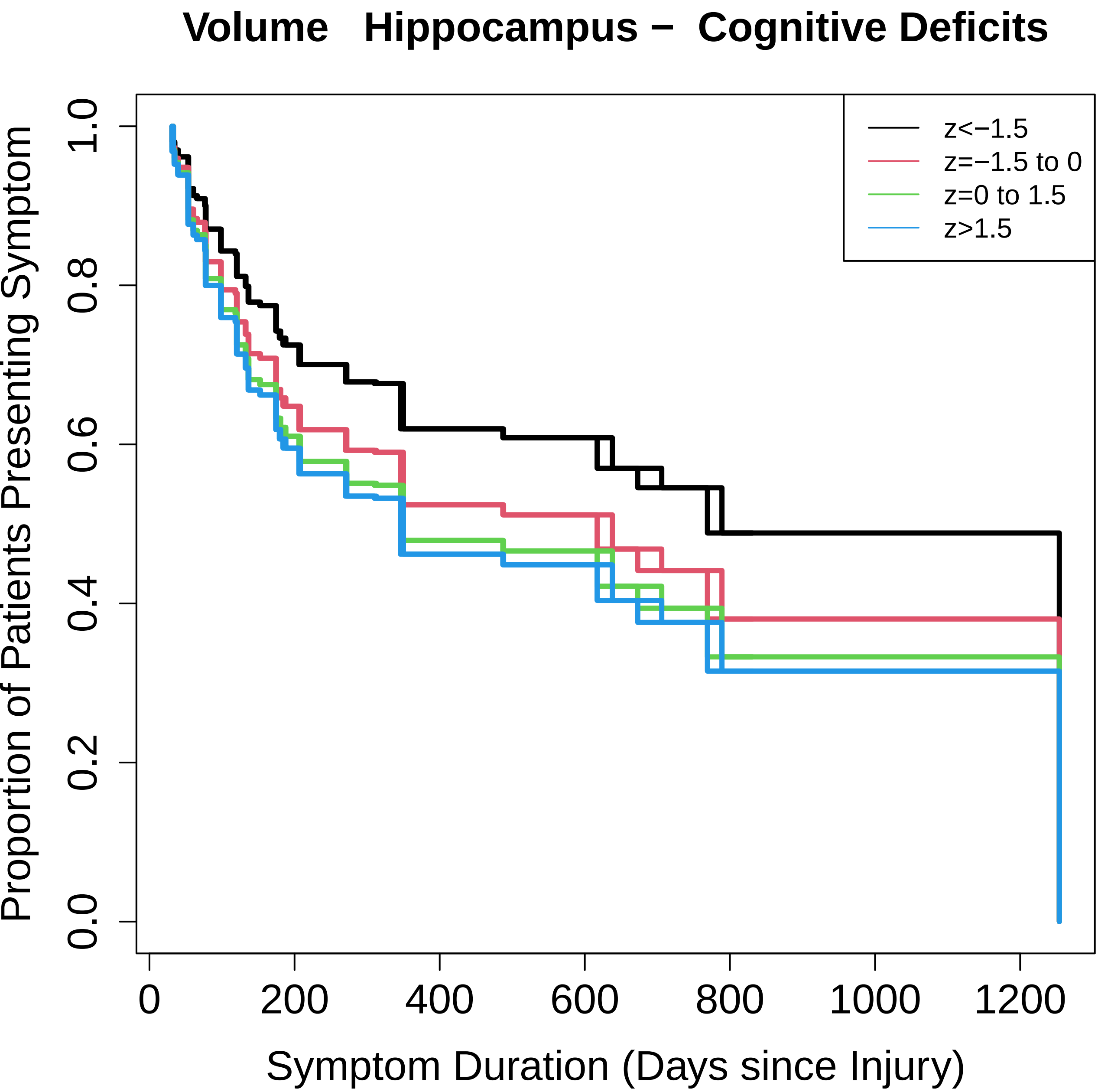
Volume Hippocampus − Cognitive Deficits
z<−1.5
z=−1.5 to 0
z=0 to 1.5
z>1.5
Proportion of Patients Presenting Symptom
0.0
0.2
0.4
0.6
0.8
1.0
0
200
400
600
800
1000
1200
Symptom Duration (Days since Injury)

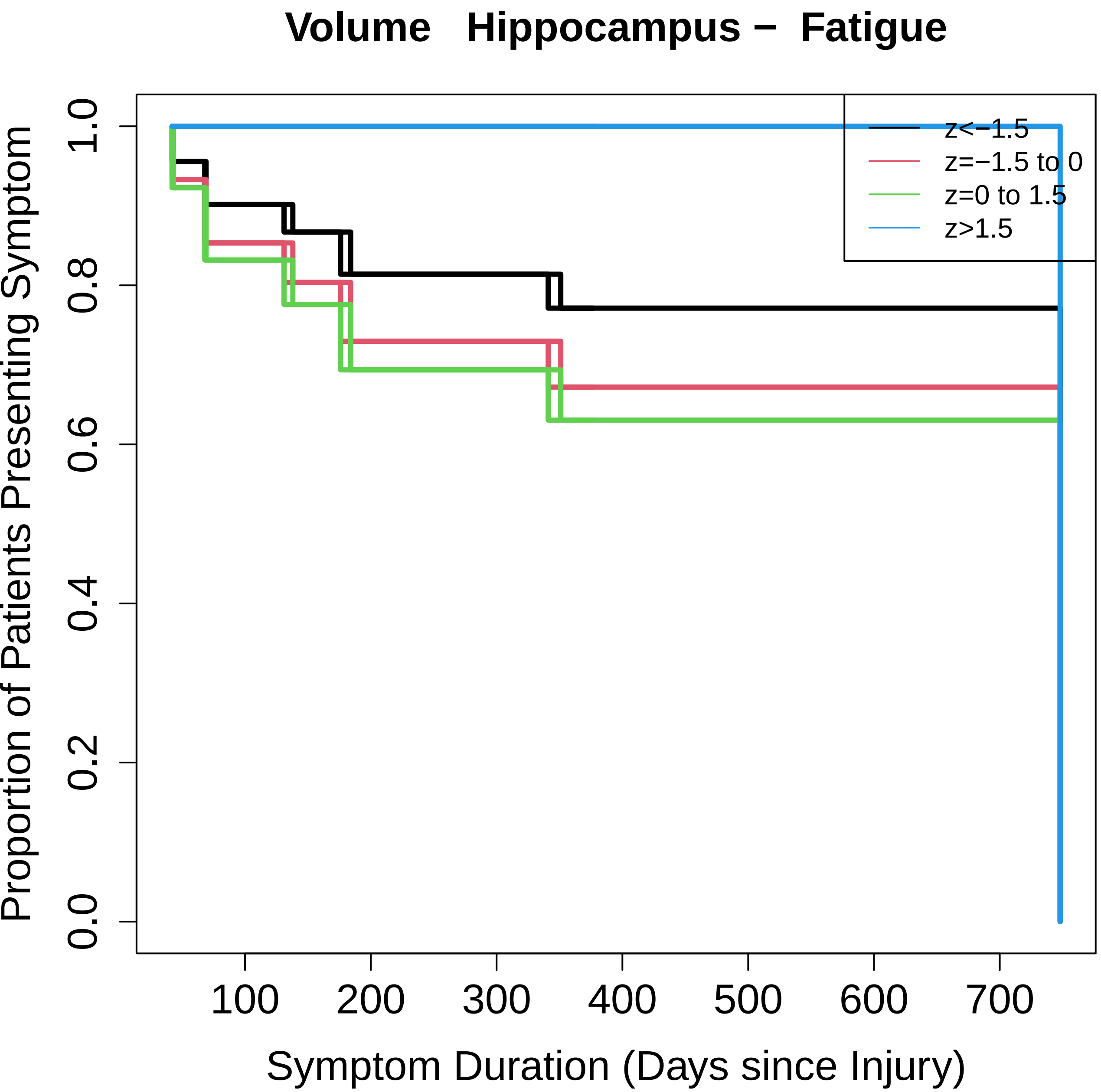
Volume Hippocampus − Fatigue
Proportion of Patients Presenting Symptom
Symptom Duration (Days since Injury)
0.0
0.2
0.4
0.6
0.8
1.0
100
200
300
400
500
600
700
z<−1.5
z=−1.5 to 0
z=0 to 1.5
z>1.5

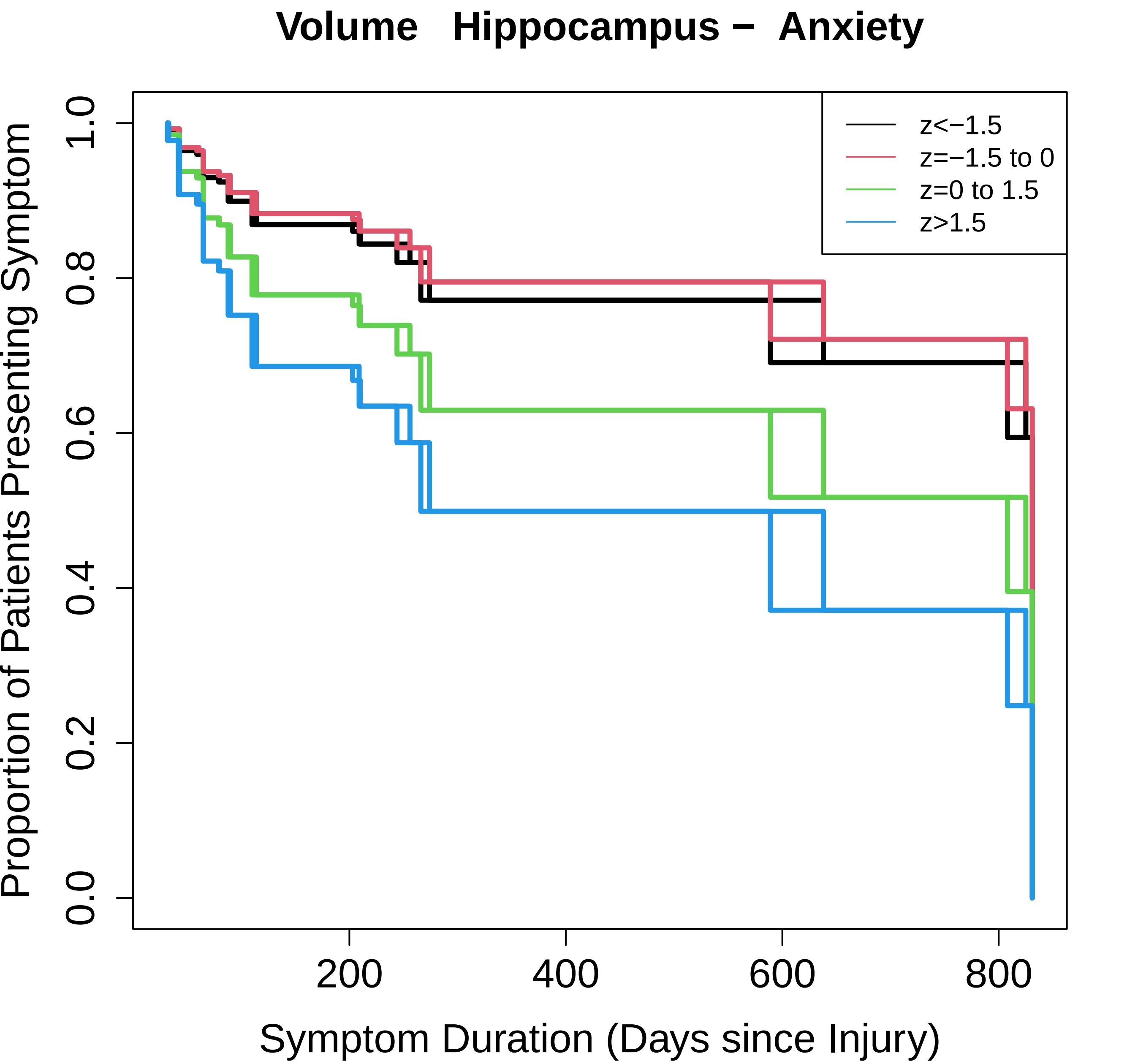

Volume Hippocampus − Anxiety
Proportion of Patients Presenting Symptom
Symptom Duration (Days since Injury)
0.0
0.2
0.4
0.6
0.8
1.0
200
400
600
800
z<−1.5
z=−1.5 to 0
z=0 to 1.5
z>1.5

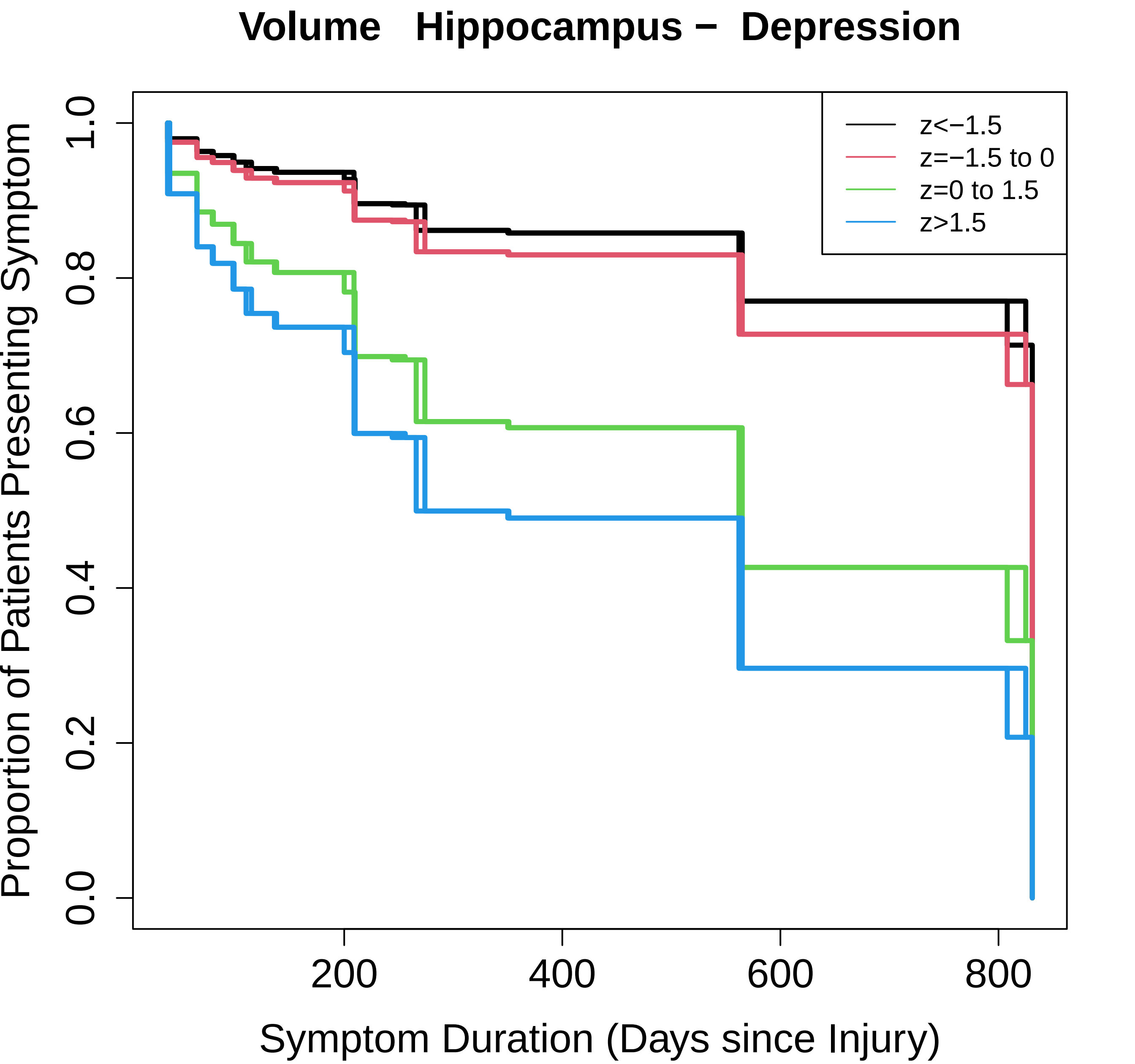
Volume Hippocampus − Depression
Proportion of Patients Presenting Symptom
Symptom Duration (Days since Injury)
0.0
0.2
0.4
0.6
0.8
1.0
200
400
600
800
z<−1.5
z=−1.5 to 0
z=0 to 1.5
z>1.5

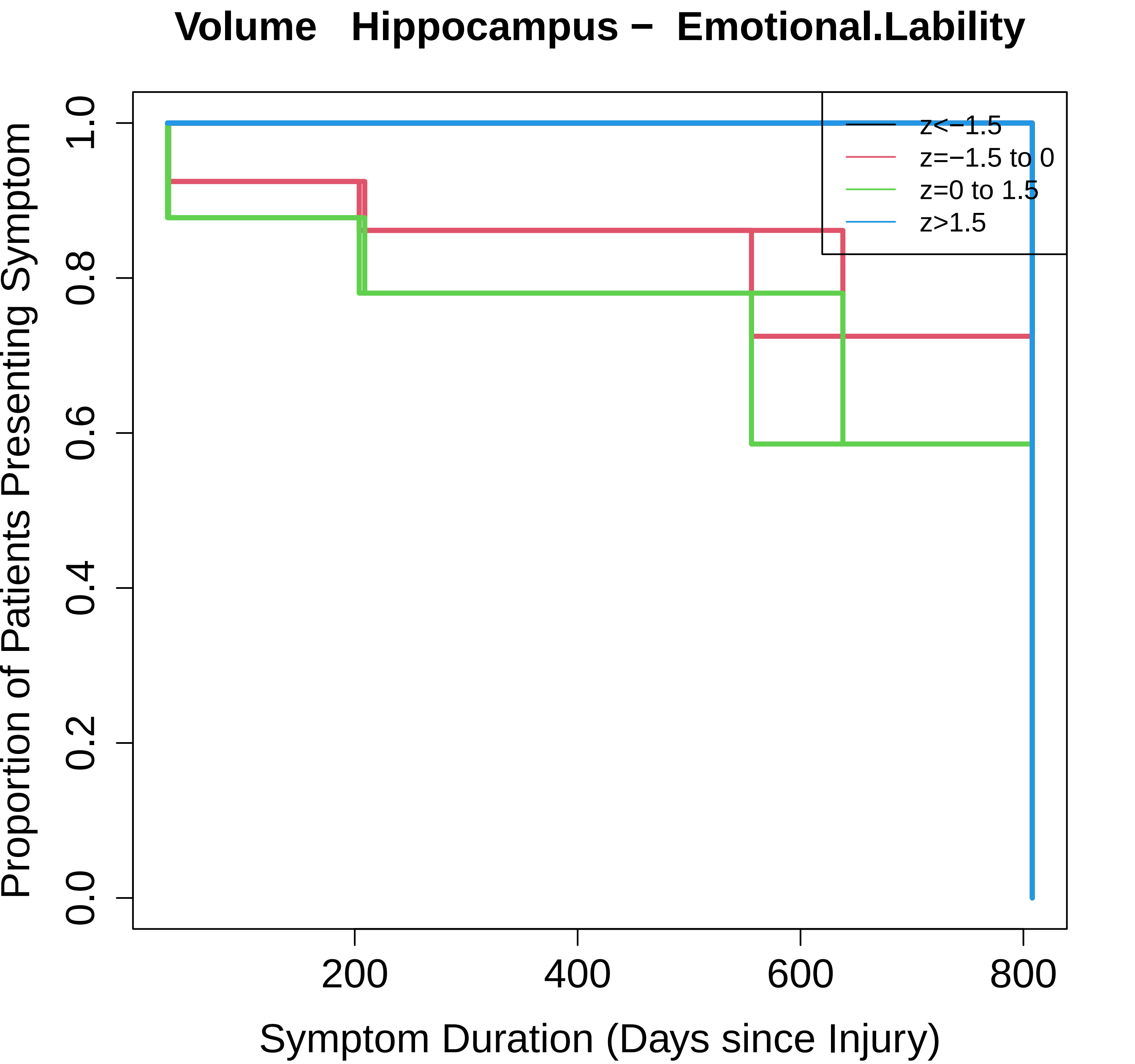

Volume Hippocampus − Emotional.Lability
Proportion of Patients Presenting Symptom
0.0
0.2
0.4
0.6
0.8
1.0
200
400
600
800
Symptom Duration (Days since Injury)
z<−1.5
z=−1.5 to 0
z=0 to 1.5
z>1.5

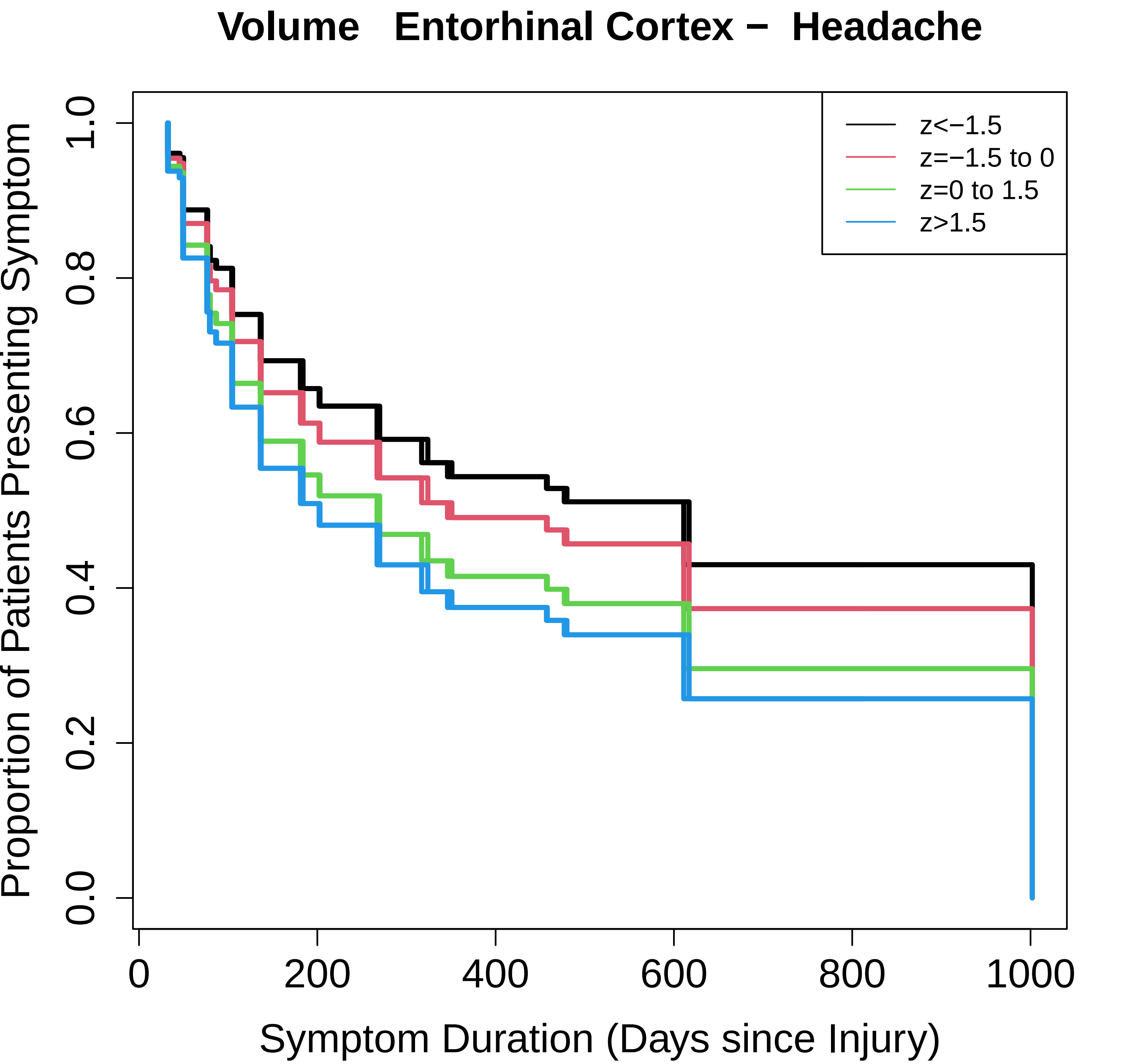
Volume Entorhinal Cortex − Headache
Proportion of Patients Presenting Symptom
Symptom Duration (Days since Injury)
z<−1.5
z=−1.5 to 0
z=0 to 1.5
z>1.5
0.0
0.2
0.4
0.6
0.8
1.0
0
200
400
600
800
1000

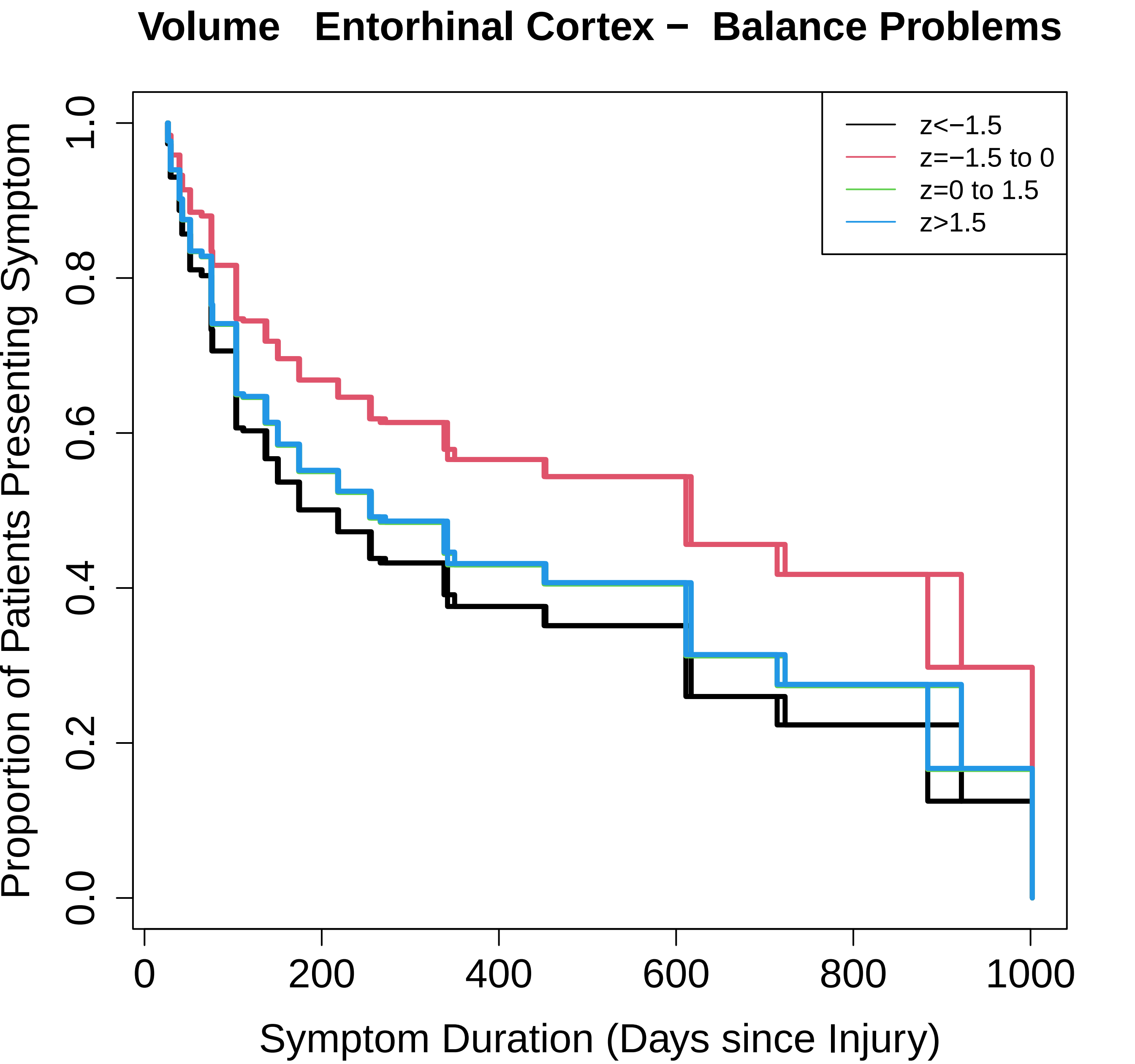
Volume Entorhinal Cortex − Balance Problems
Proportion of Patients Presenting Symptom
Symptom Duration (Days since Injury)
z<−1.5
z=−1.5 to 0
z=0 to 1.5
z>1.5
0.0
0.2
0.4
0.6
0.8
1.0
0
200
400
600
800
1000

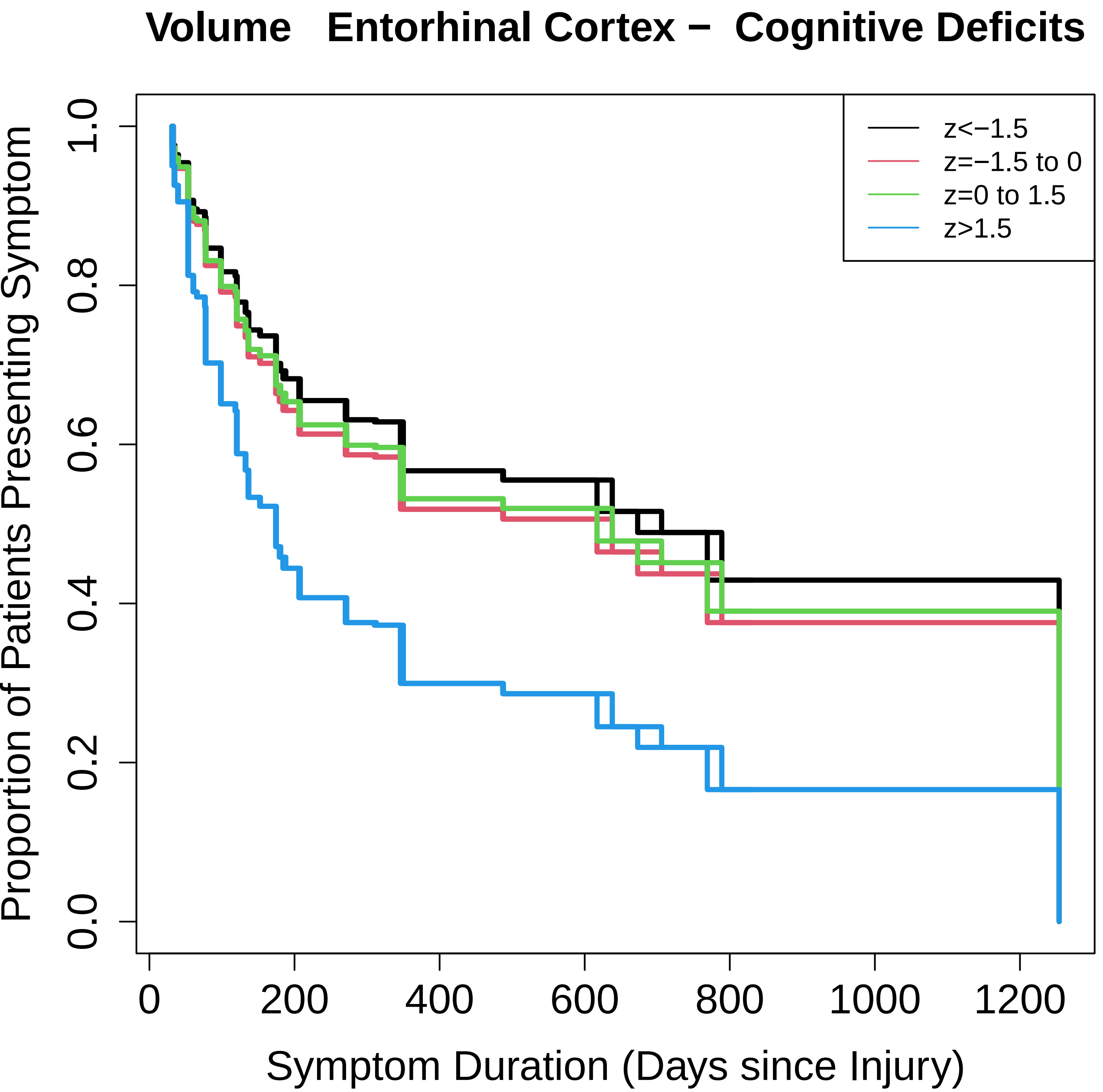
Volume Entorhinal Cortex − Cognitive Deficits
Proportion of Patients Presenting Symptom
0.0
0.2
0.4
0.6
0.8
1.0
0
200
400
600
800
1000
1200
Symptom Duration (Days since Injury)
z<−1.5
z=−1.5 to 0
z=0 to 1.5
z>1.5

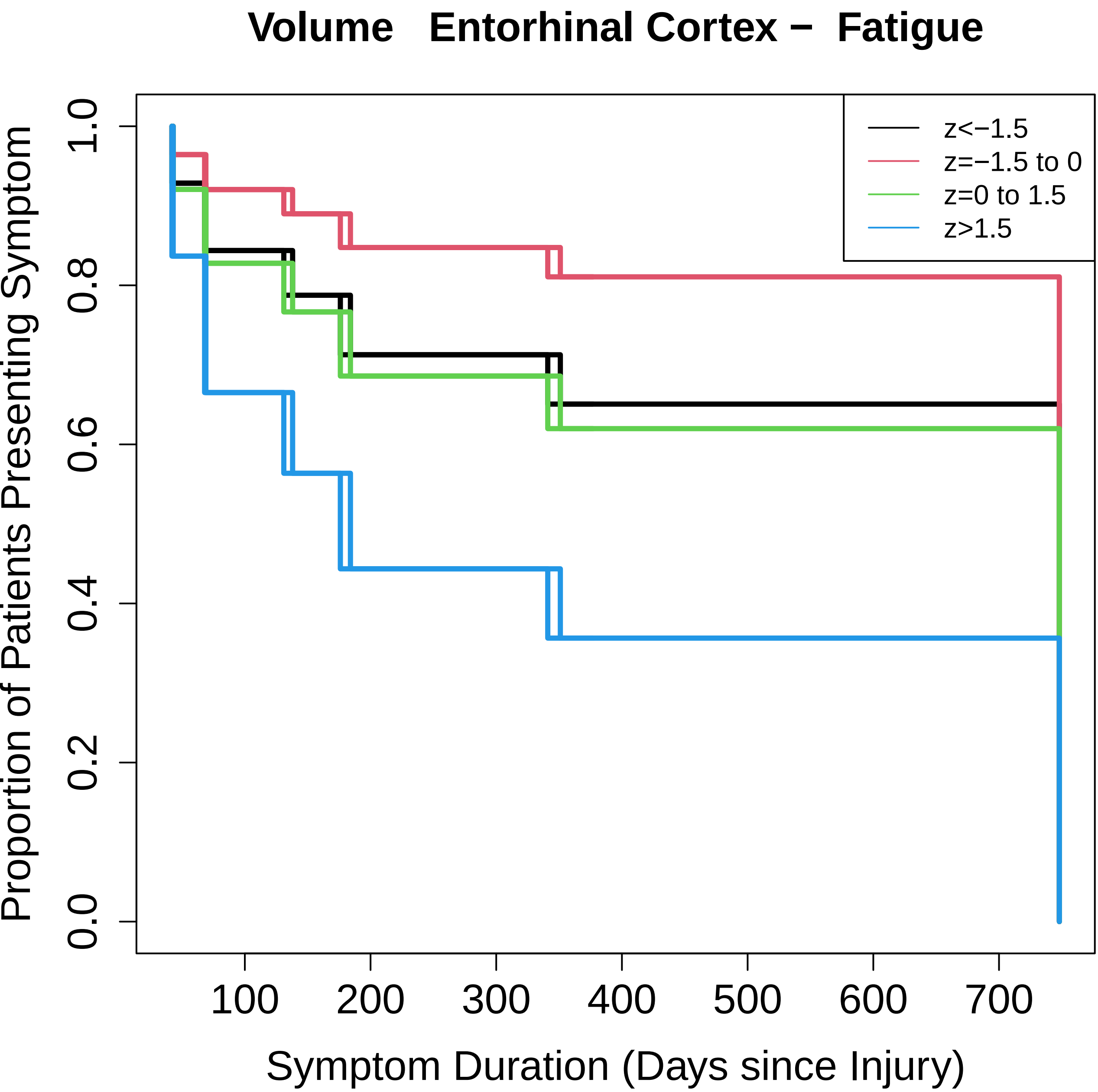

Volume Entorhinal Cortex − Fatigue
Proportion of Patients Presenting Symptom
Symptom Duration (Days since Injury)
0.0
0.2
0.4
0.6
0.8
1.0
100
200
300
400
500
600
700
z<−1.5
z=−1.5 to 0
z=0 to 1.5
z>1.5

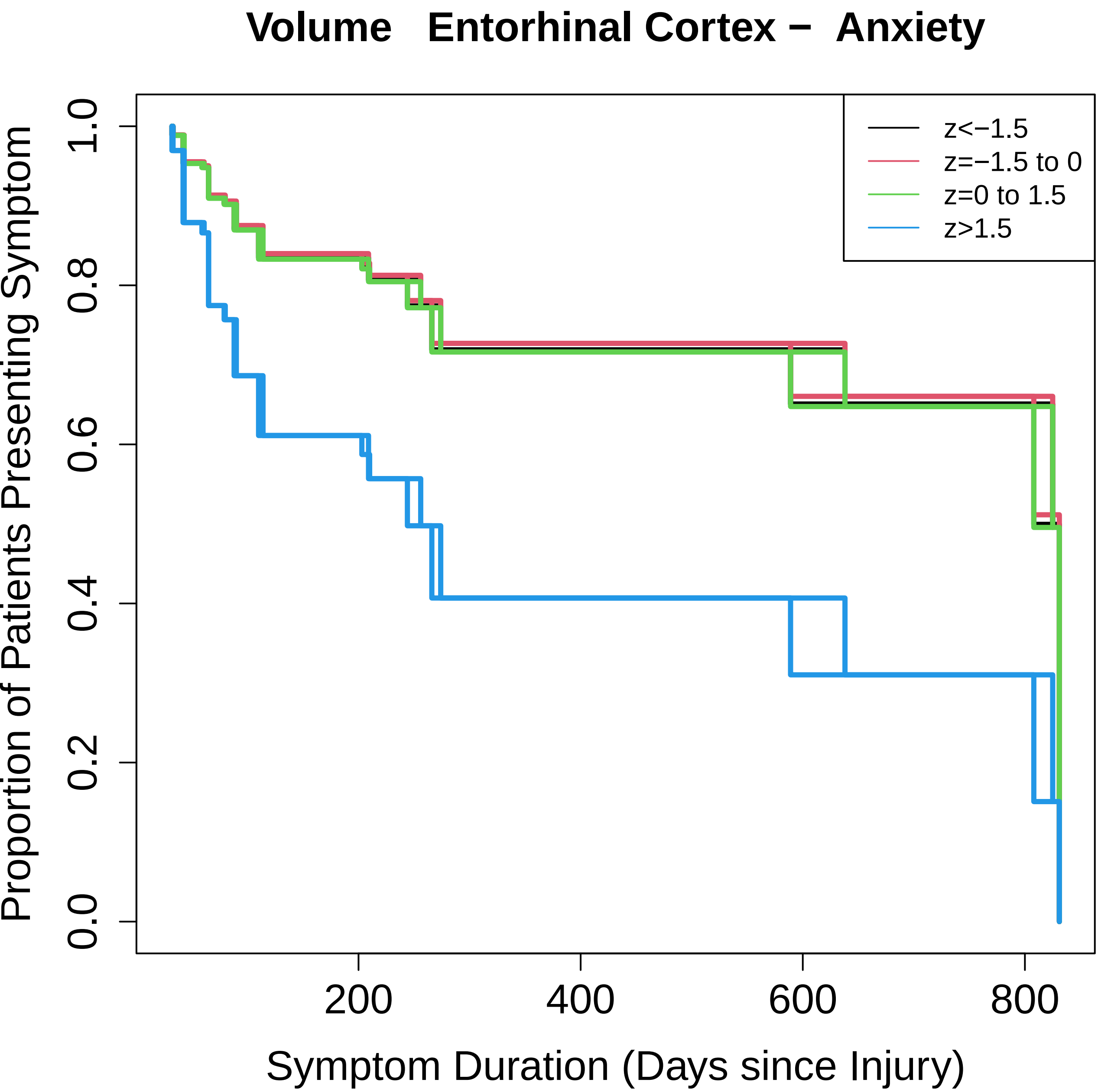

Volume Entorhinal Cortex − Anxiety
z<−1.5
z=−1.5 to 0
z=0 to 1.5
z>1.5
Proportion of Patients Presenting Symptom
0.0
0.2
0.4
0.6
0.8
1.0
200
400
600
800
Symptom Duration (Days since Injury)

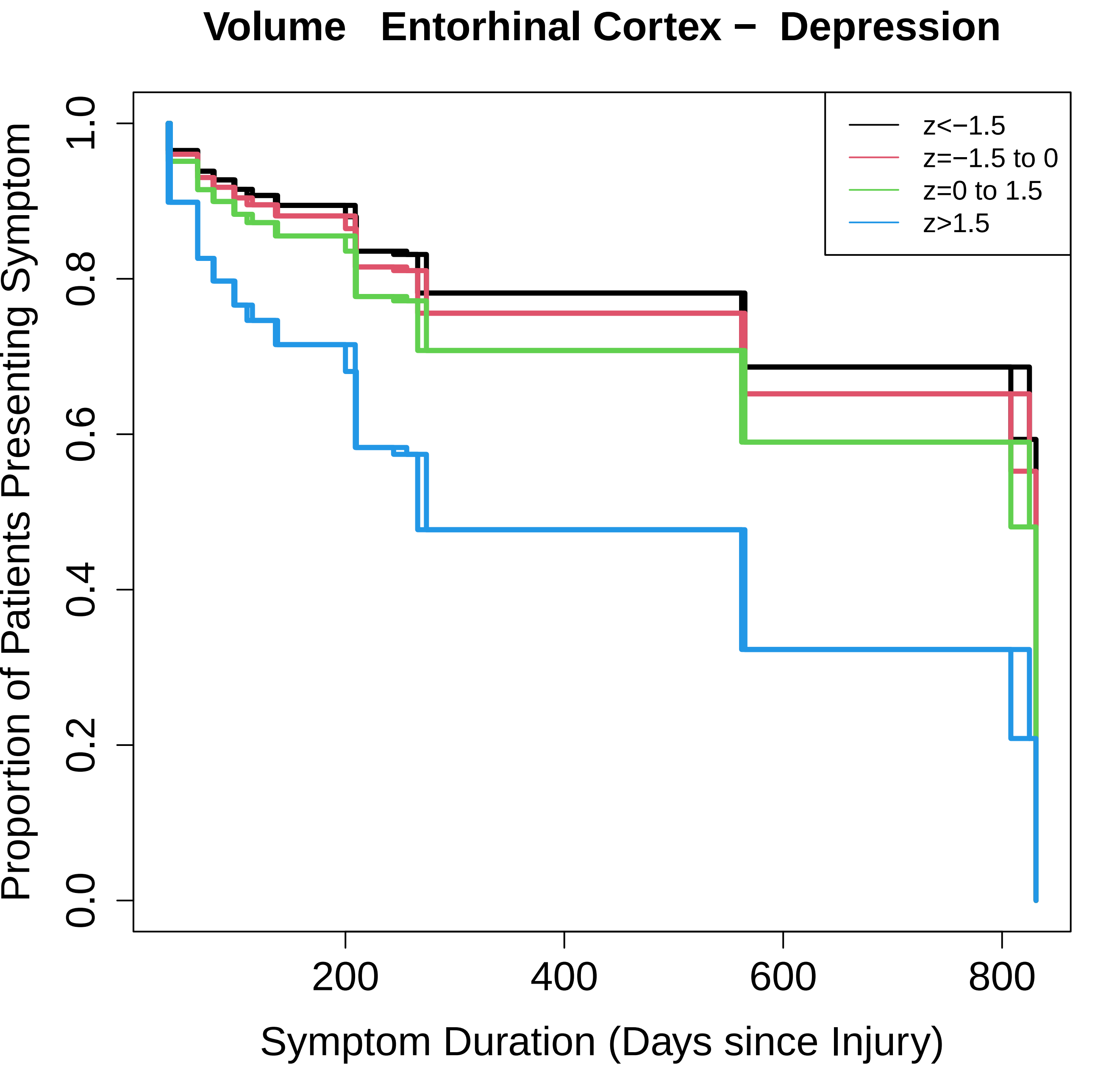
Volume Entorhinal Cortex − Depression
Proportion of Patients Presenting Symptom
Symptom Duration (Days since Injury)
0.0
0.2
0.4
0.6
0.8
1.0
200
400
600
800
z<−1.5
z=−1.5 to 0
z=0 to 1.5
z>1.5

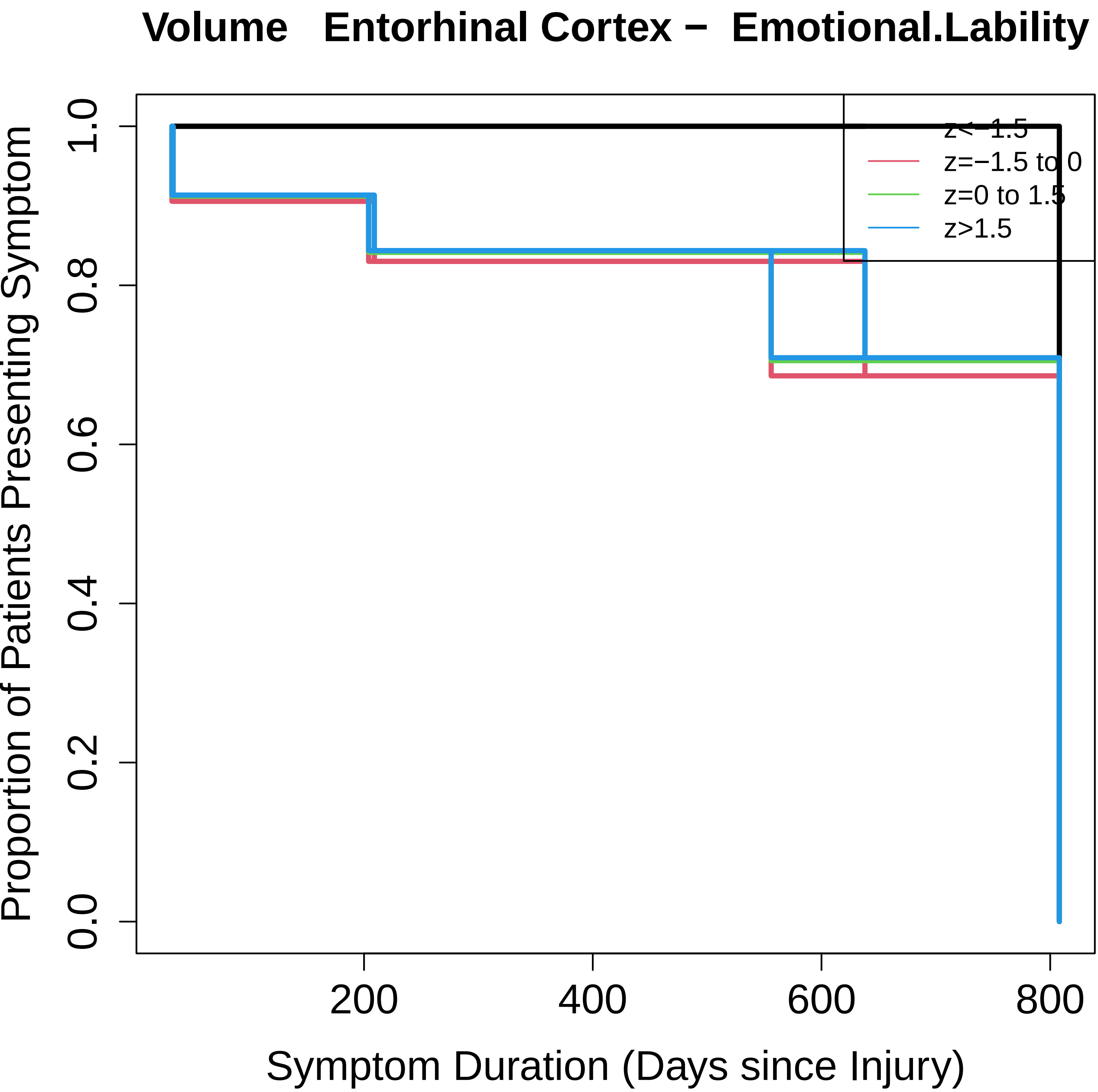

Volume Entorhinal Cortex − Emotional.Lability
z<−1.5
z=−1.5 to 0
z=0 to 1.5
z>1.5
Proportion of Patients Presenting Symptom
0.0
0.2
0.4
0.6
0.8
1.0
200
400
600
800
Symptom Duration (Days since Injury)

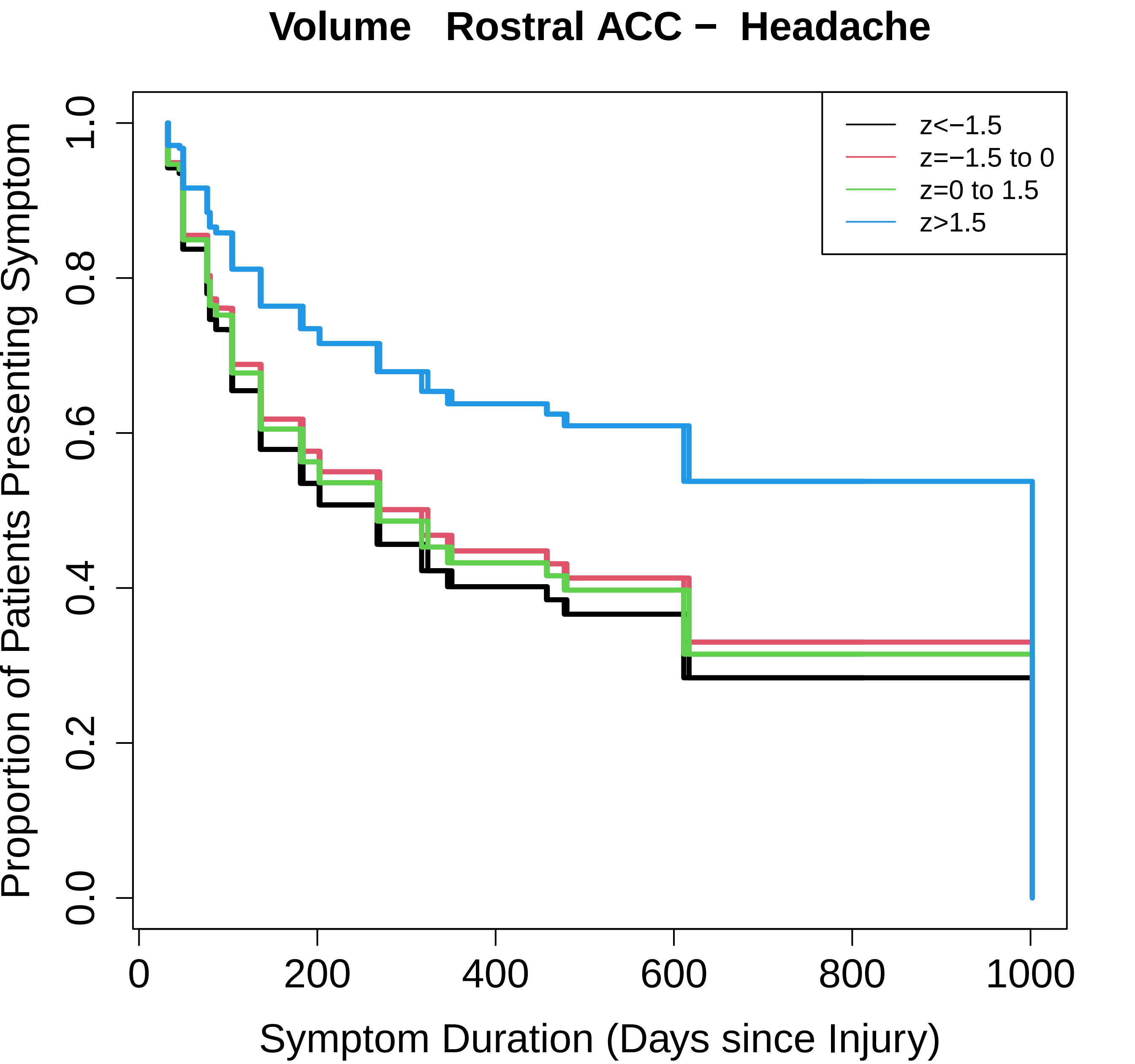

Volume Rostral ACC − Headache
Proportion of Patients Presenting Symptom
Symptom Duration (Days since Injury)
z<−1.5
z=−1.5 to 0
z=0 to 1.5
z>1.5
0.0
0.2
0.4
0.6
0.8
1.0
0
200
400
600
800
1000

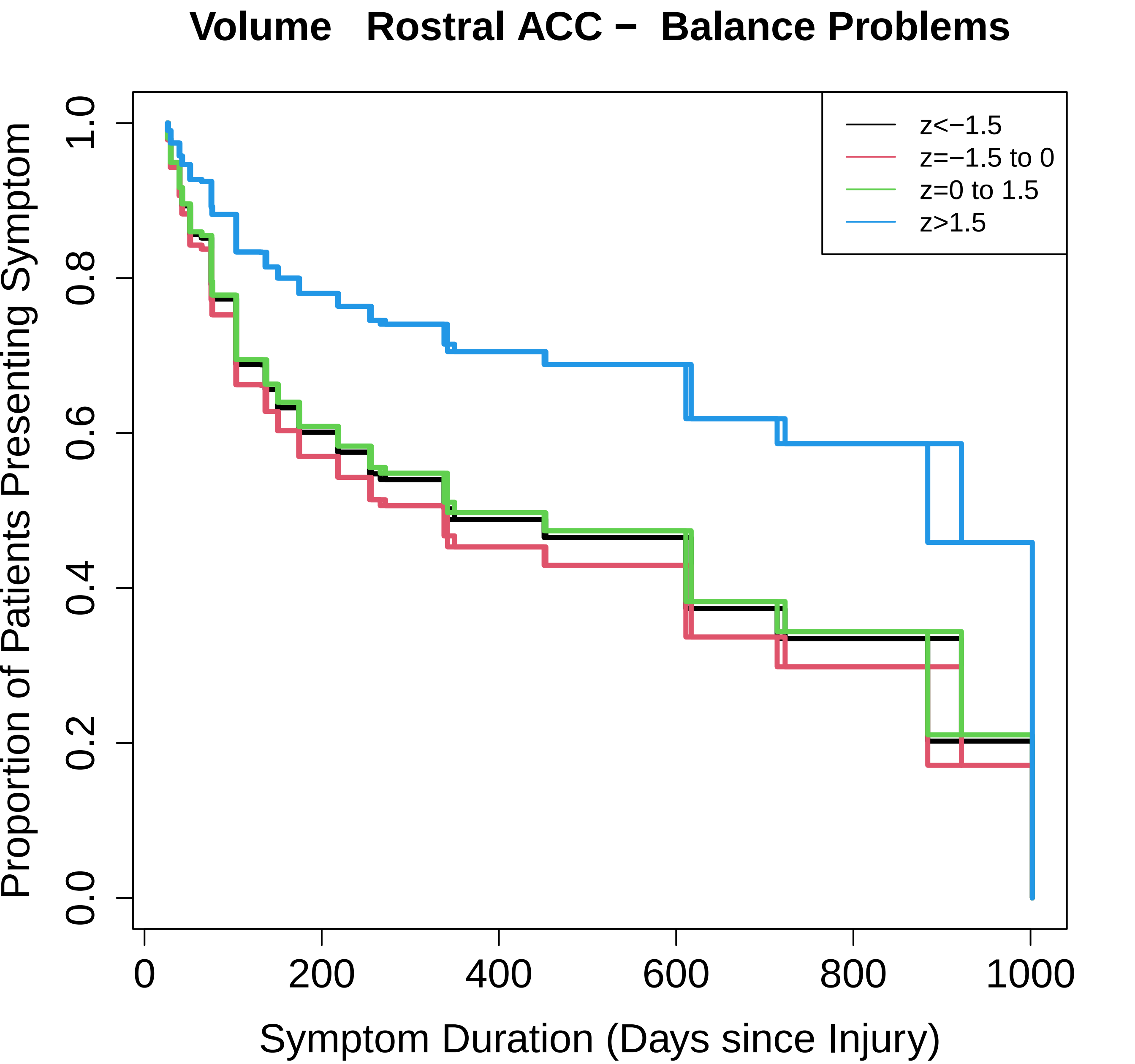

Volume Rostral ACC − Balance Problems
Proportion of Patients Presenting Symptom
Symptom Duration (Days since Injury)
0.0
0.2
0.4
0.6
0.8
1.0
0
200
400
600
800
1000
z<−1.5
z=−1.5 to 0
z=0 to 1.5
z>1.5

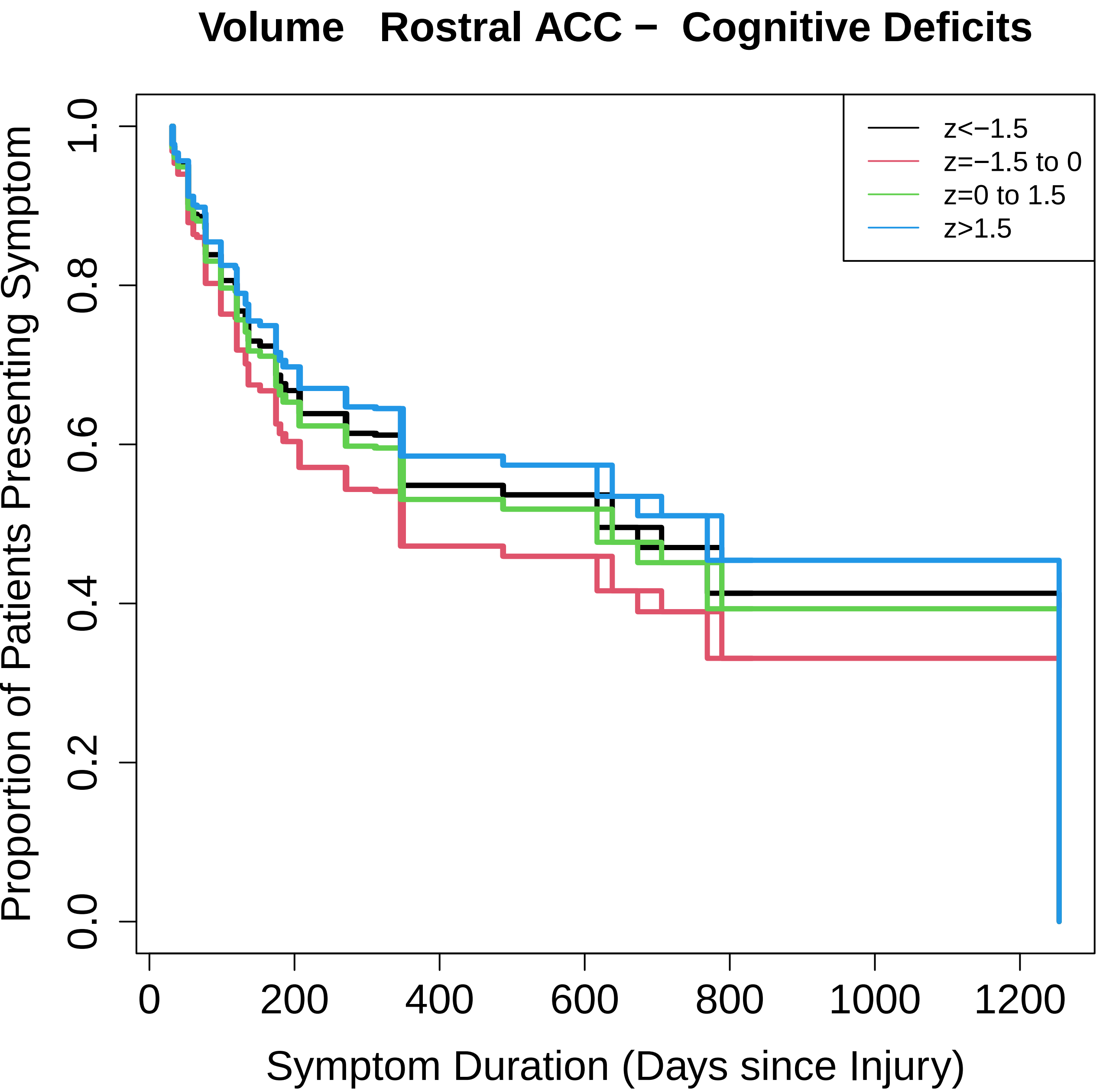

Volume Rostral ACC − Cognitive Deficits
Proportion of Patients Presenting Symptom
Symptom Duration (Days since Injury)
0.0
0.2
0.4
0.6
0.8
1.0
0
200
400
600
800
1000
1200
z<−1.5
z=−1.5 to 0
z=0 to 1.5
z>1.5

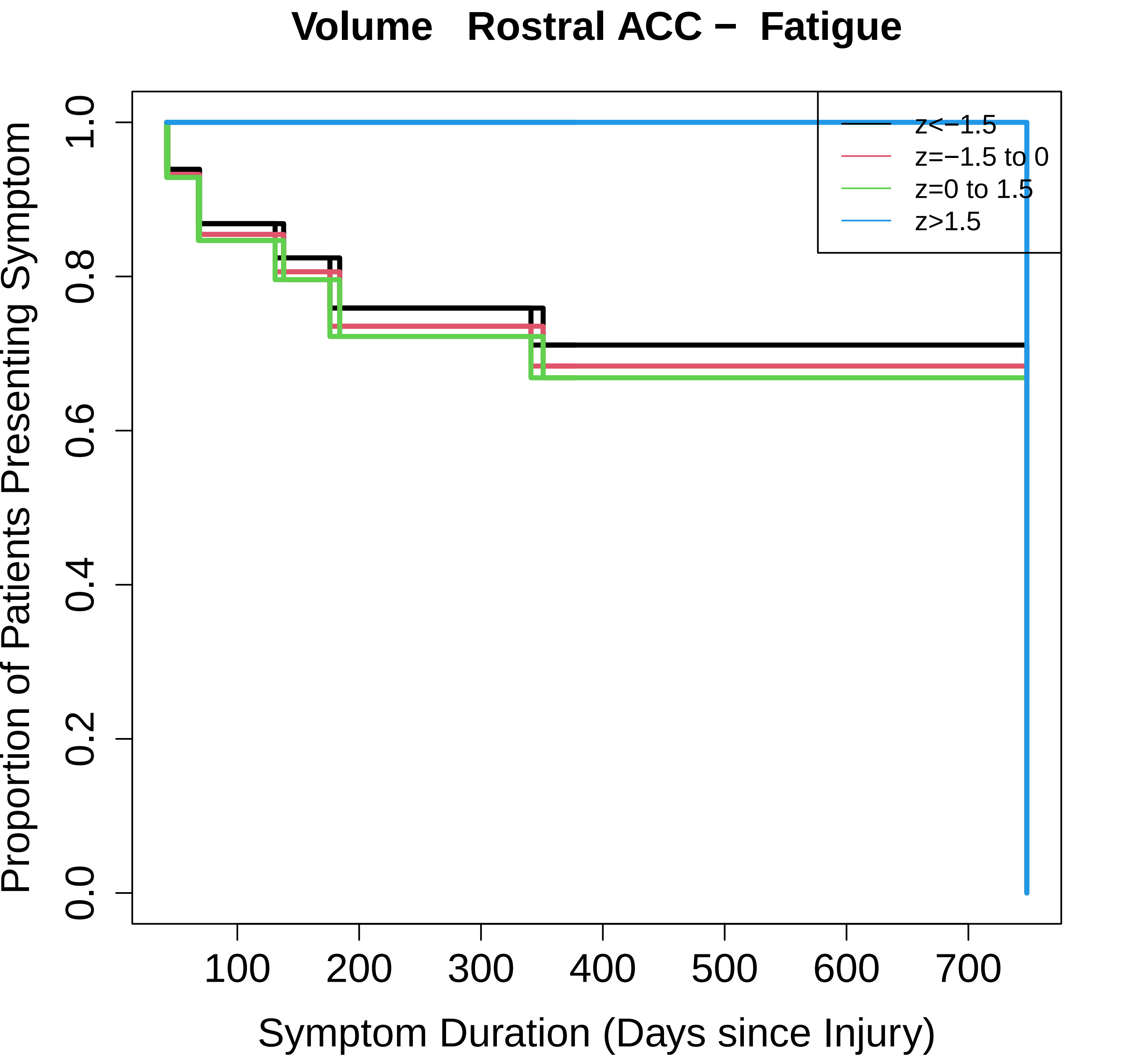
Volume Rostral ACC − Fatigue
Proportion of Patients Presenting Symptom
Symptom Duration (Days since Injury)
z<−1.5
z=−1.5 to 0
z=0 to 1.5
z>1.5
0.0
0.2
0.4
0.6
0.8
1.0
100
200
300
400
500
600
700

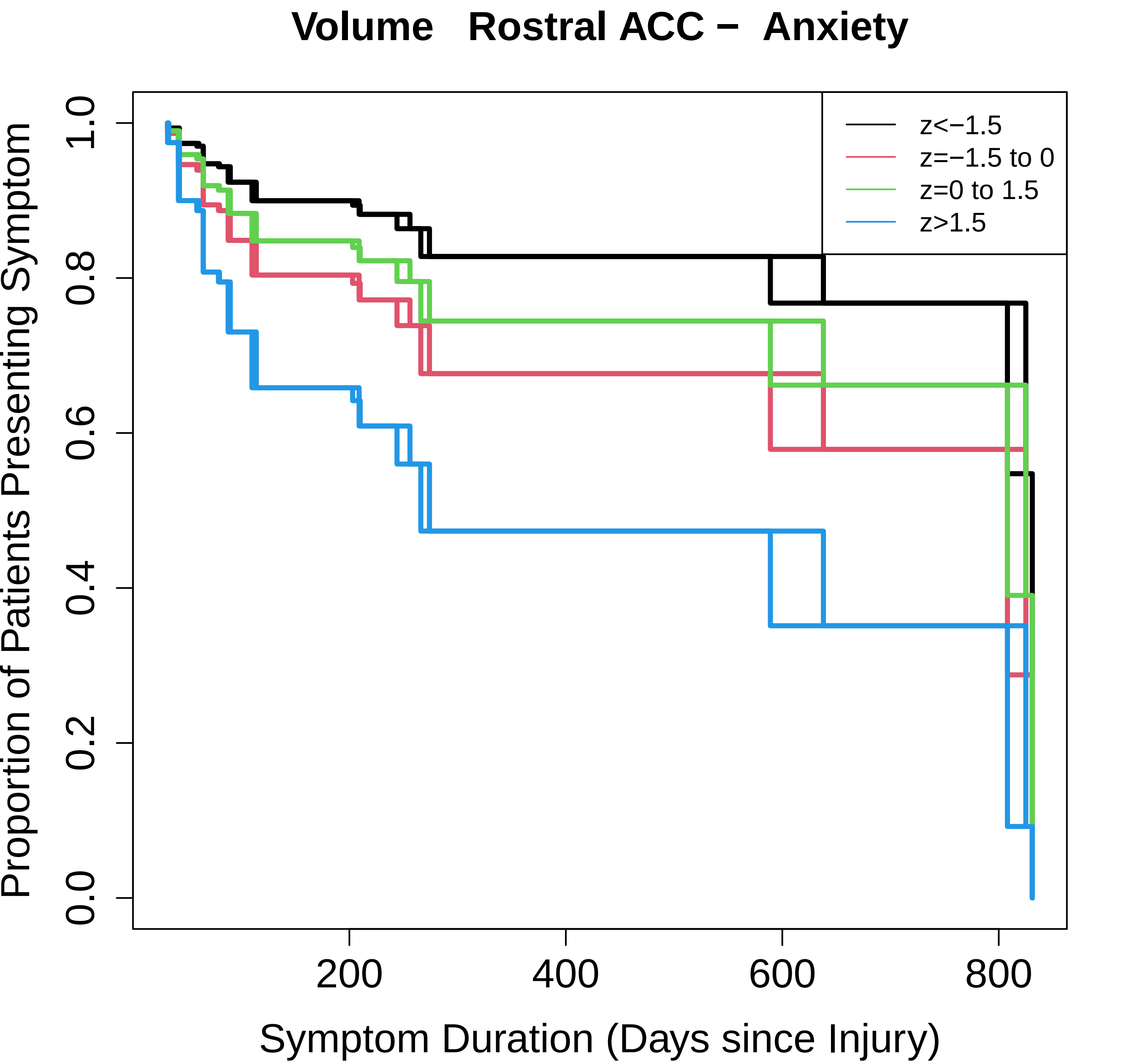

Volume Rostral ACC − Anxiety
Proportion of Patients Presenting Symptom
0.0
0.2
0.4
0.6
0.8
1.0
200
400
600
800
Symptom Duration (Days since Injury)
z<−1.5
z=−1.5 to 0
z=0 to 1.5
z>1.5

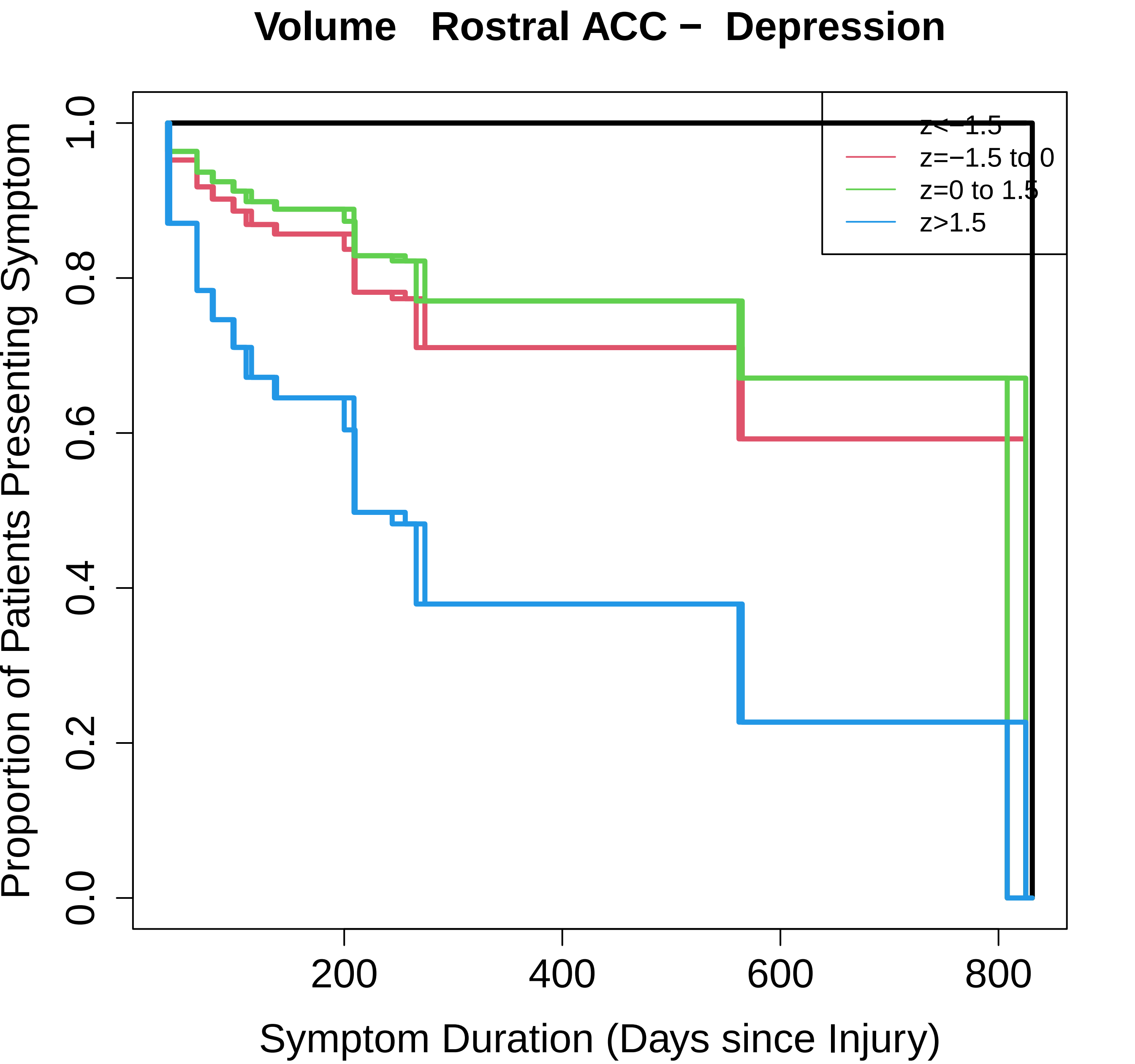

Volume Rostral ACC − Depression
Proportion of Patients Presenting Symptom
Symptom Duration (Days since Injury)
0.0
0.2
0.4
0.6
0.8
1.0
200
400
600
800
z<−1.5
z=−1.5 to 0
z=0 to 1.5
z>1.5

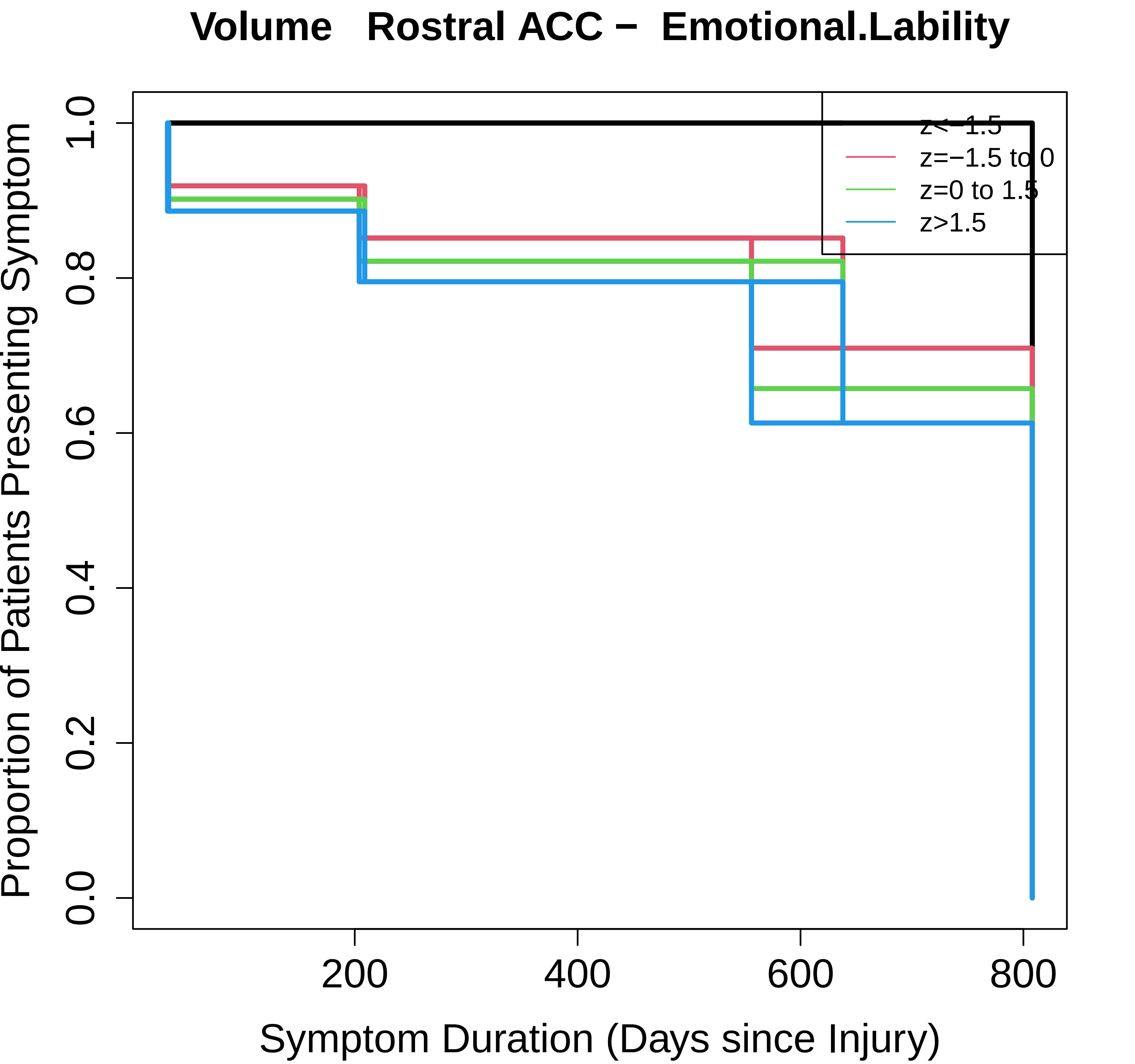

Volume Rostral ACC − Emotional.Lability
Proportion of Patients Presenting Symptom
0.0
0.2
0.4
0.6
0.8
1.0
200
400
600
800
Symptom Duration (Days since Injury)
z<−1.5
z=−1.5 to 0
z=0 to 1.5
z>1.5

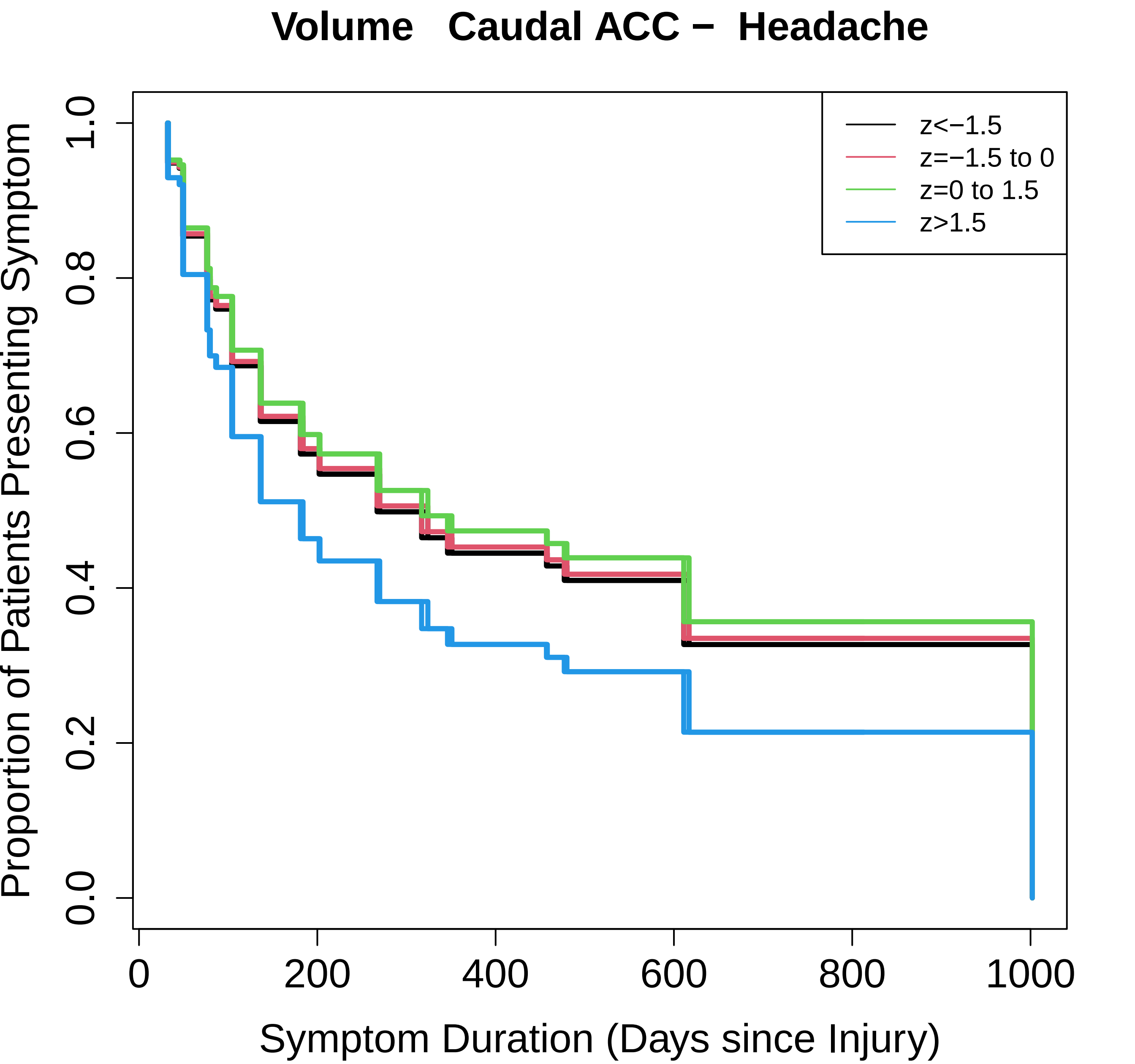
Volume Caudal ACC − Headache
z<−1.5
z=−1.5 to 0
z=0 to 1.5
z>1.5
Proportion of Patients Presenting Symptom
0.0
0.2
0.4
0.6
0.8
1.0
0
200
400
600
800
1000
Symptom Duration (Days since Injury)

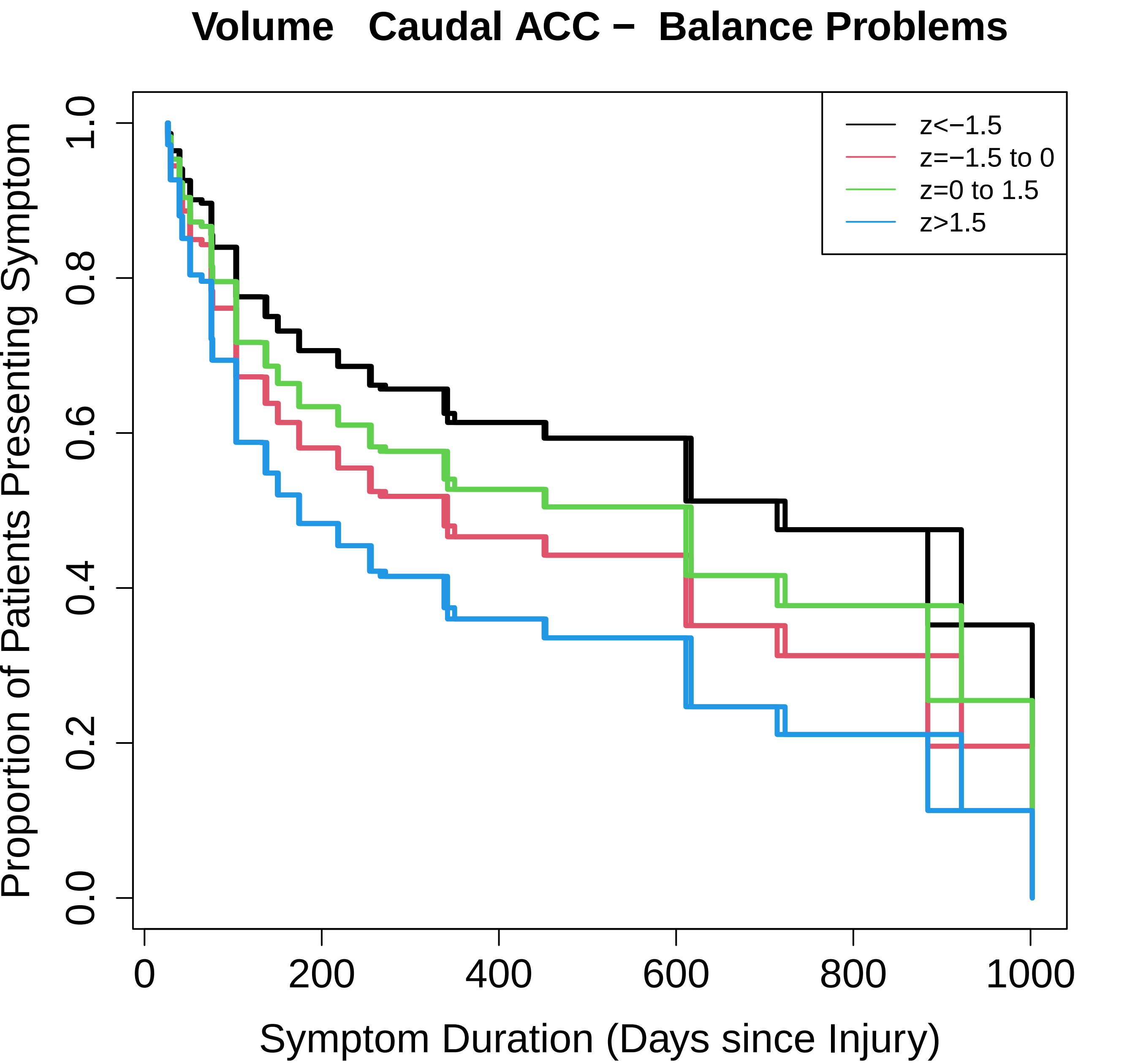

Volume Caudal ACC − Balance Problems
z<−1.5
z=−1.5 to 0
z=0 to 1.5
z>1.5
Proportion of Patients Presenting Symptom
0.0
0.2
0.4
0.6
0.8
1.0
0
200
400
600
800
1000
Symptom Duration (Days since Injury)

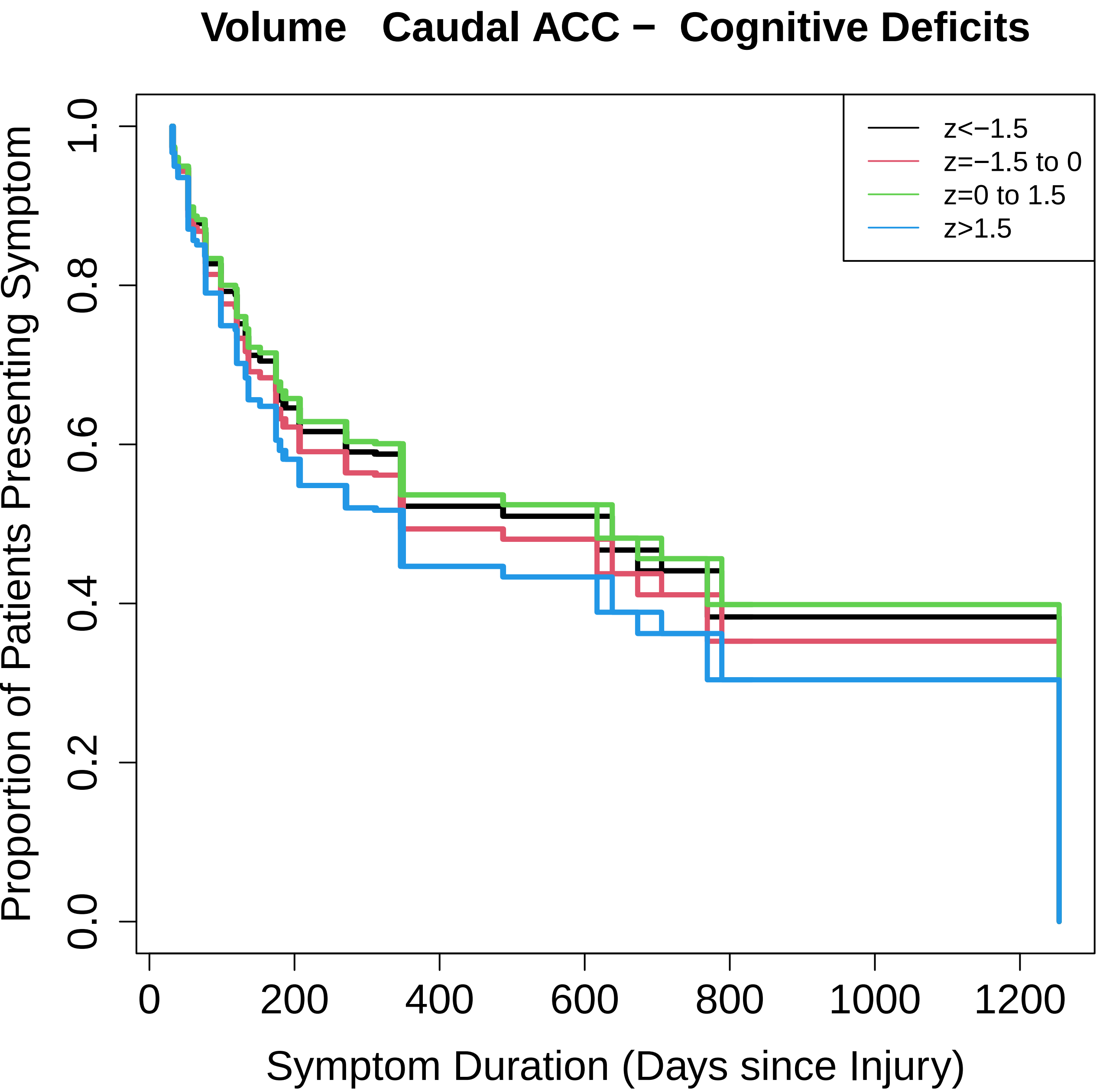

Volume Caudal ACC − Cognitive Deficits
Proportion of Patients Presenting Symptom
0.0
0.2
0.4
0.6
0.8
1.0
0
200
400
600
800
1000
1200
Symptom Duration (Days since Injury)
z<−1.5
z=−1.5 to 0
z=0 to 1.5
z>1.5

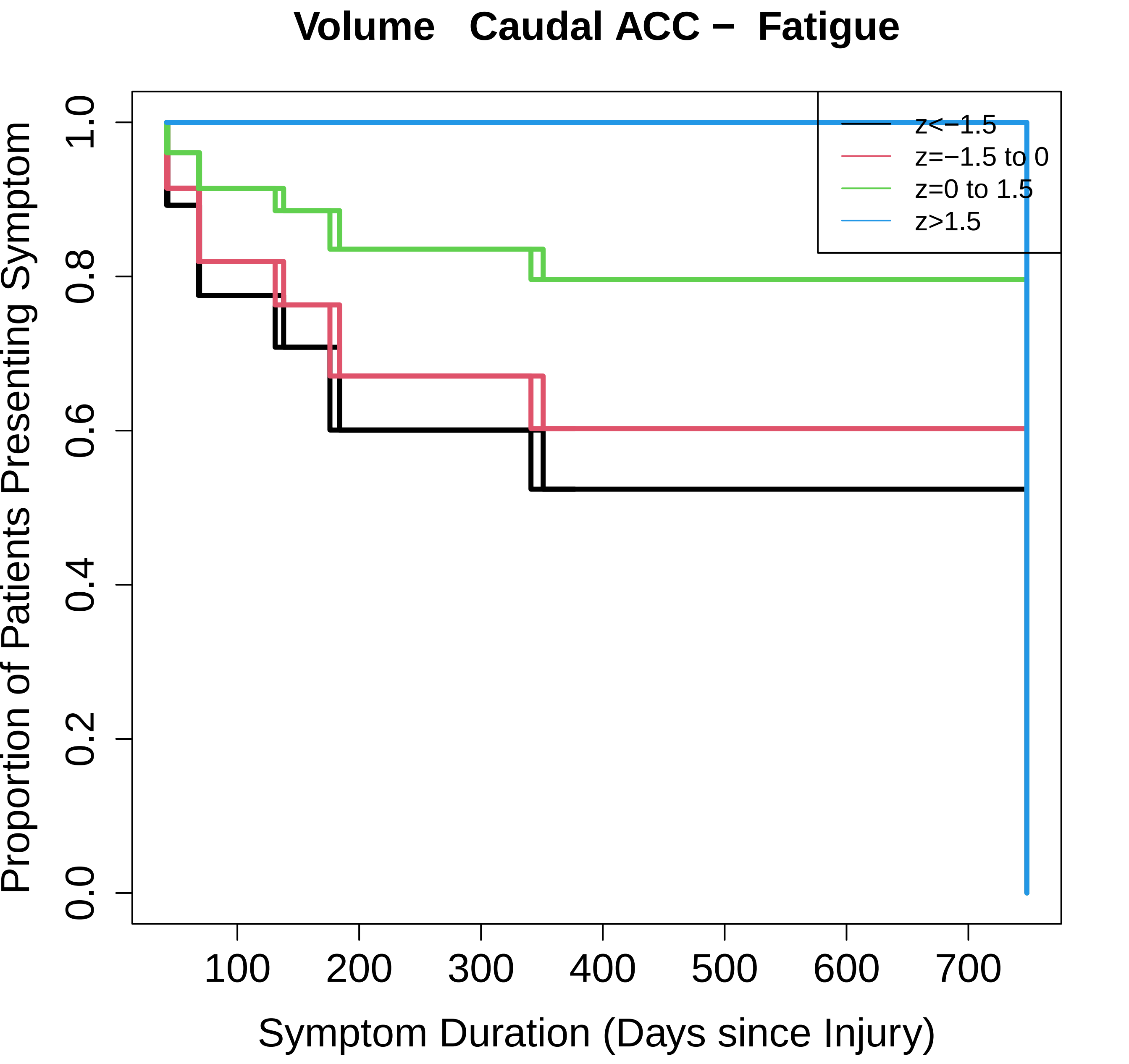

Volume Caudal ACC − Fatigue
Proportion of Patients Presenting Symptom
0.0
0.2
0.4
0.6
0.8
1.0
100
200
300
400
500
600
700
Symptom Duration (Days since Injury)
z<−1.5
z=−1.5 to 0
z=0 to 1.5
z>1.5

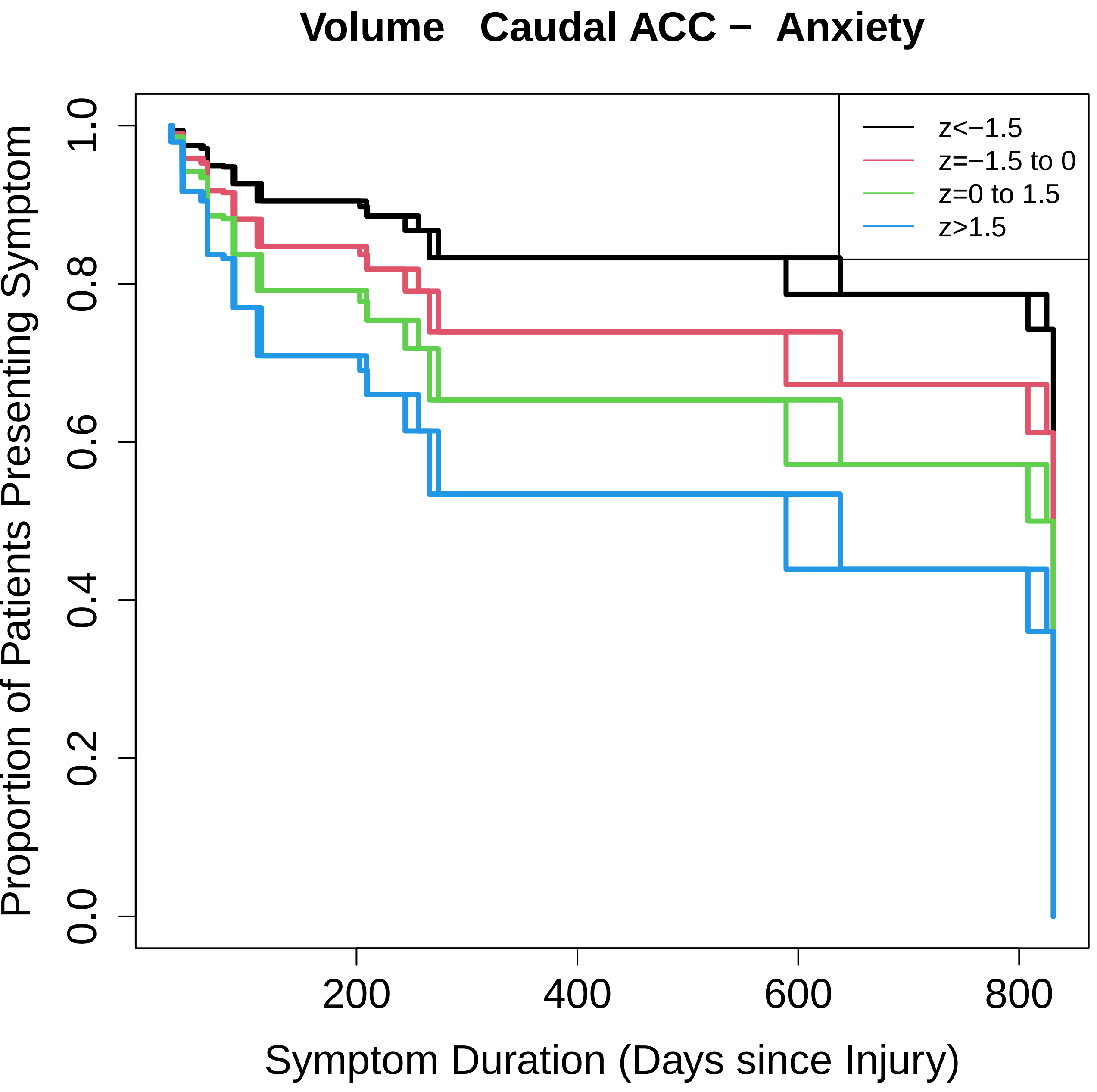

Volume Caudal ACC − Anxiety
Proportion of Patients Presenting Symptom
Symptom Duration (Days since Injury)
0.0
0.2
0.4
0.6
0.8
1.0
200
400
600
800
z<−1.5
z=−1.5 to 0
z=0 to 1.5
z>1.5

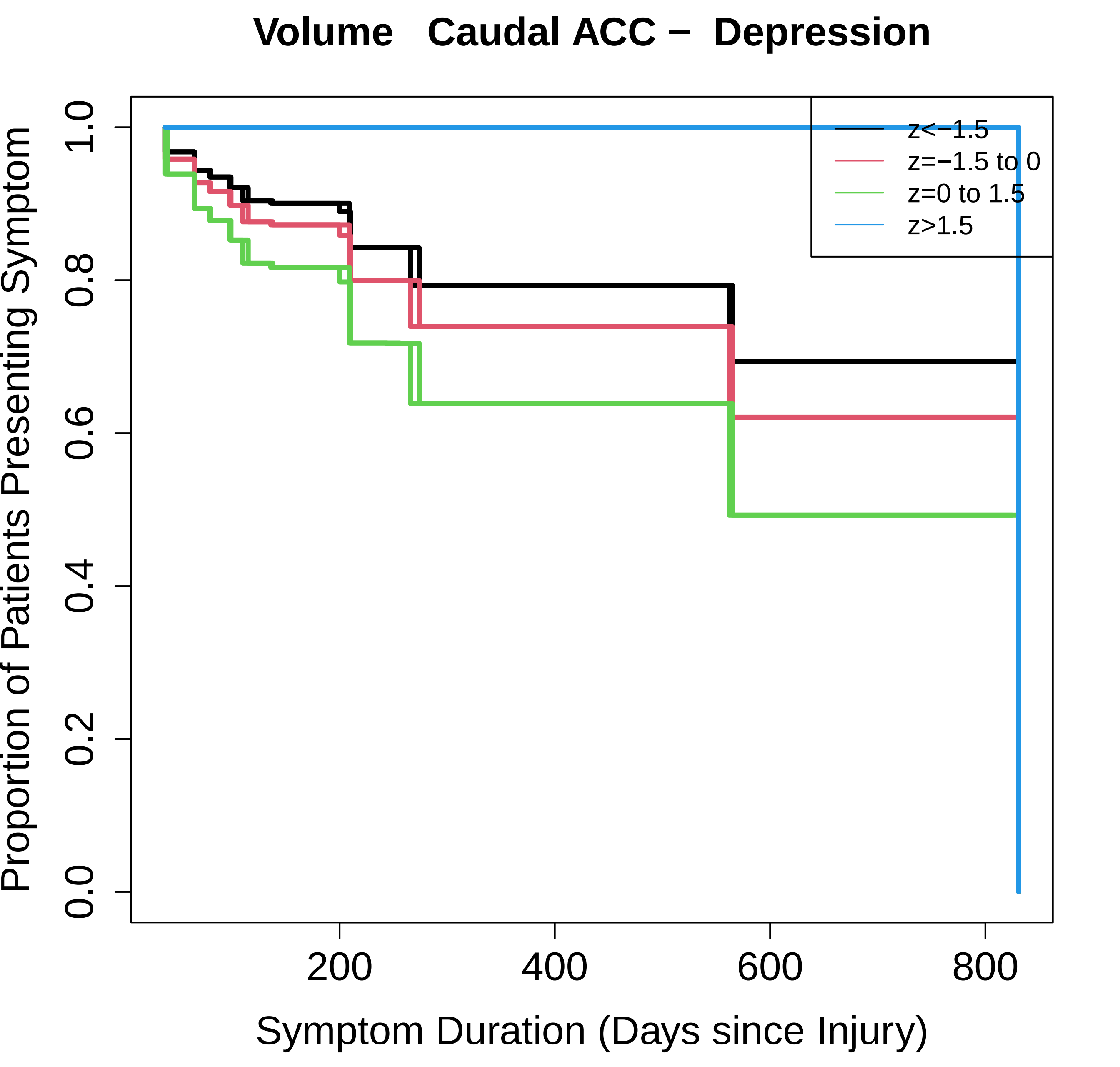
Volume Caudal ACC − Depression
Proportion of Patients Presenting Symptom
Symptom Duration (Days since Injury)
1.0
0.8
0.6
0.4
0.2
0.0
200
400
600
800
z<−1.5
z=−1.5 to 0
z=0 to 1.5
z>1.5

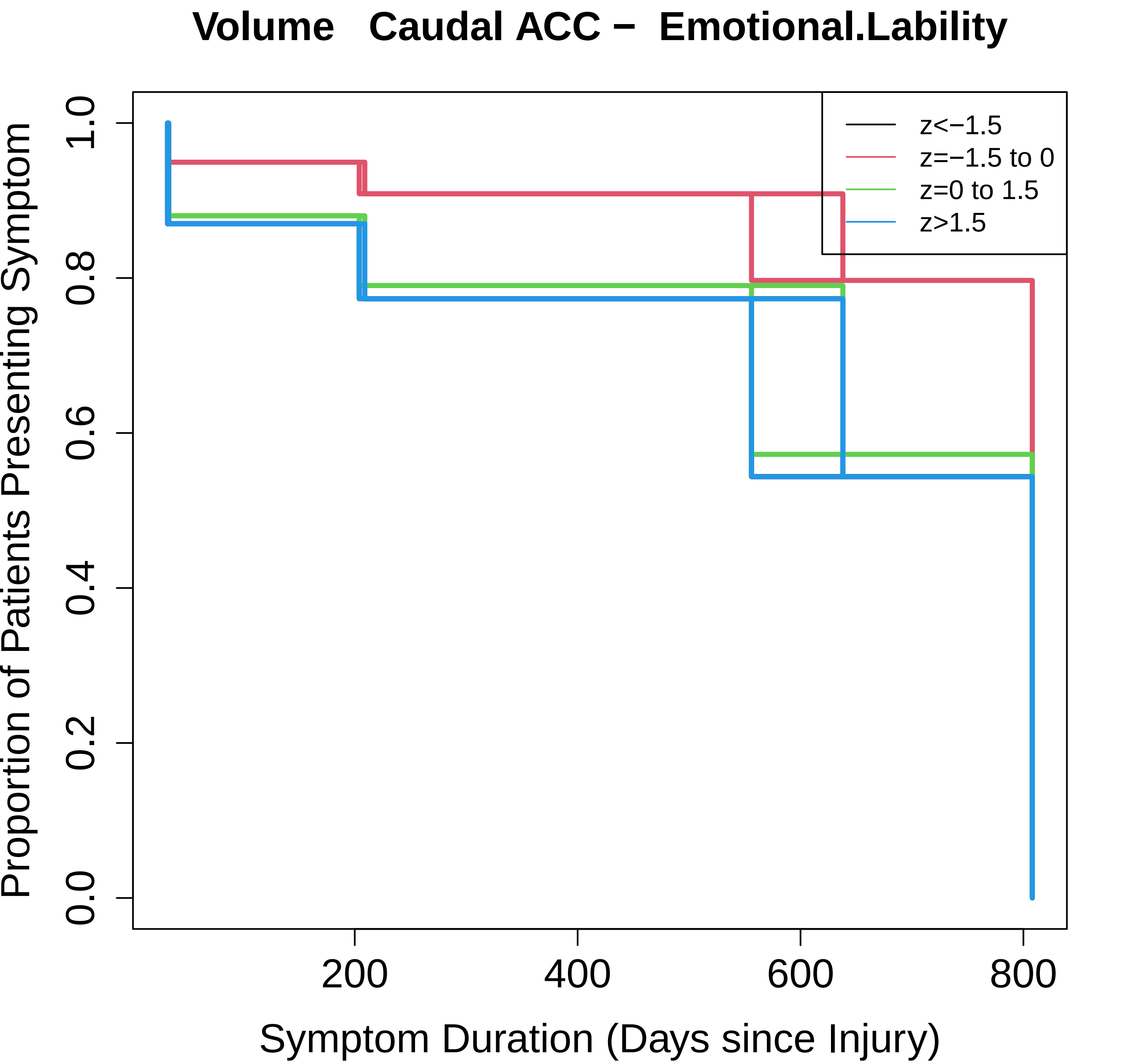
Volume Caudal ACC − Emotional.Lability
Proportion of Patients Presenting Symptom
Symptom Duration (Days since Injury)
0.0
0.2
0.4
0.6
0.8
1.0
200
400
600
800
z<−1.5
z=−1.5 to 0
z=0 to 1.5
z>1.5

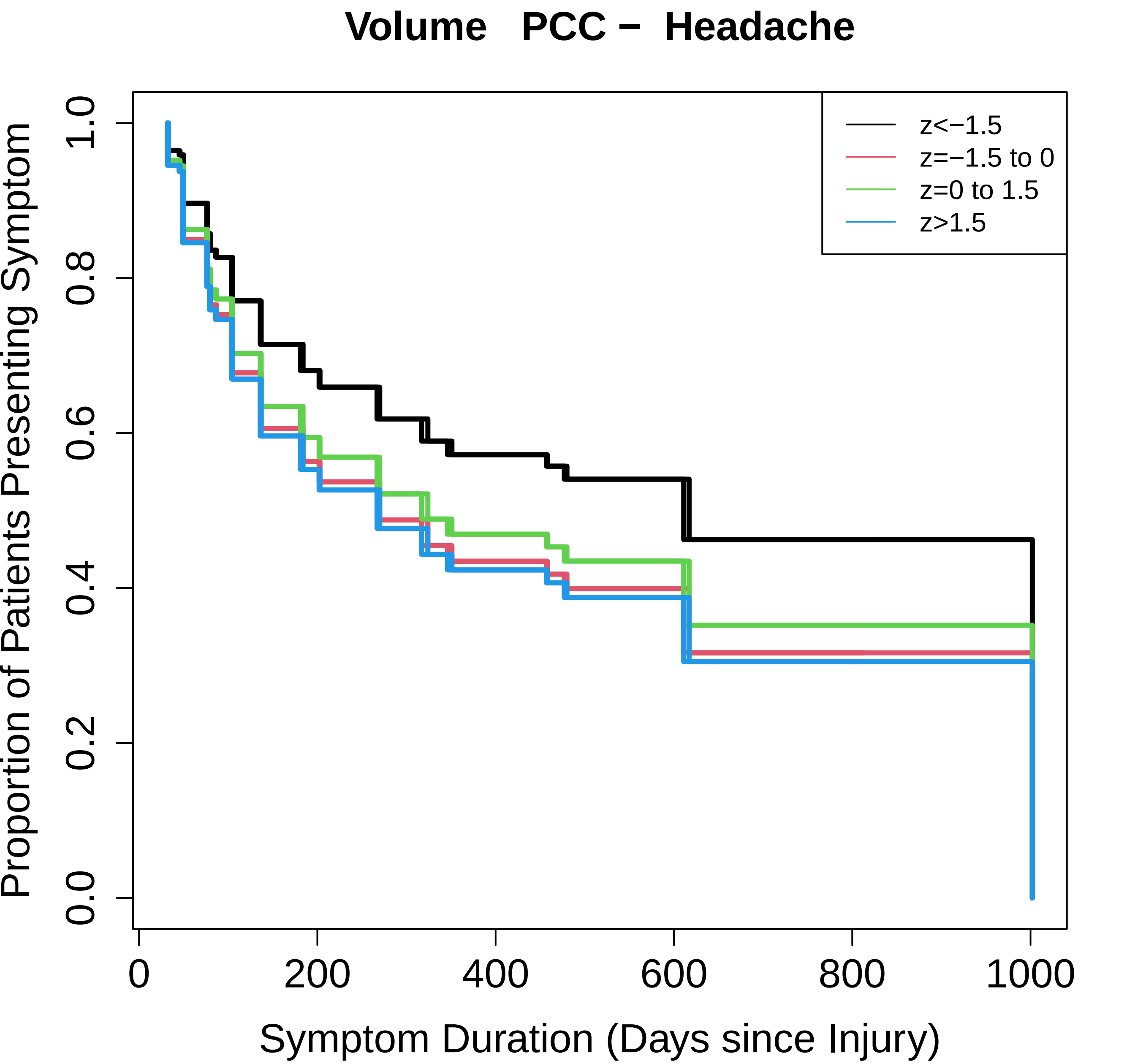

Volume PCC − Headache
Proportion of Patients Presenting Symptom
Symptom Duration (Days since Injury)
0.0
0.2
0.4
0.6
0.8
1.0
0
200
400
600
800
1000
z<−1.5
z=−1.5 to 0
z=0 to 1.5
z>1.5

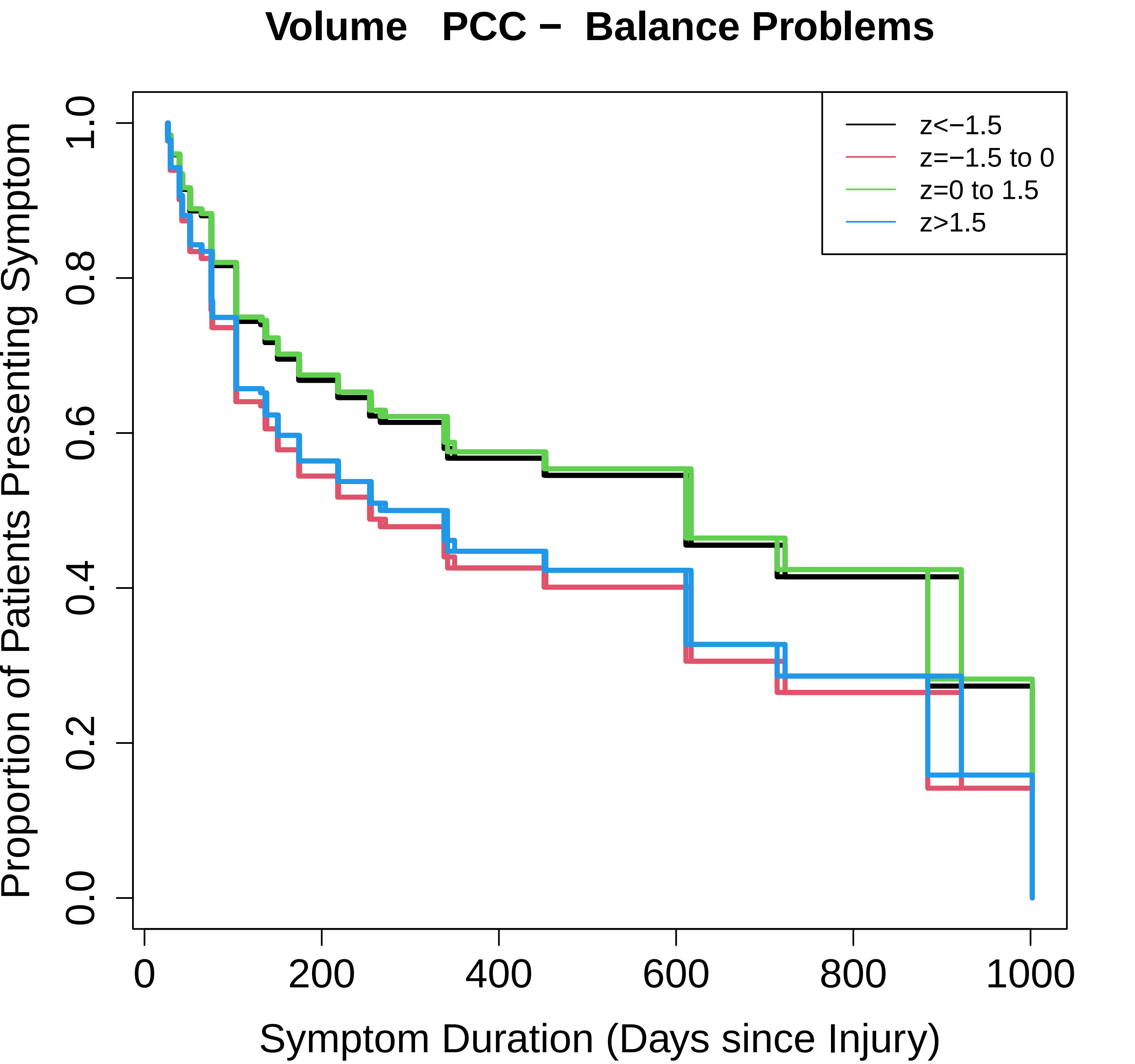
Volume PCC − Balance Problems
z<−1.5
z=−1.5 to 0
z=0 to 1.5
z>1.5
Proportion of Patients Presenting Symptom
0.0
0.2
0.4
0.6
0.8
1.0
0
200
400
600
800
1000
Symptom Duration (Days since Injury)

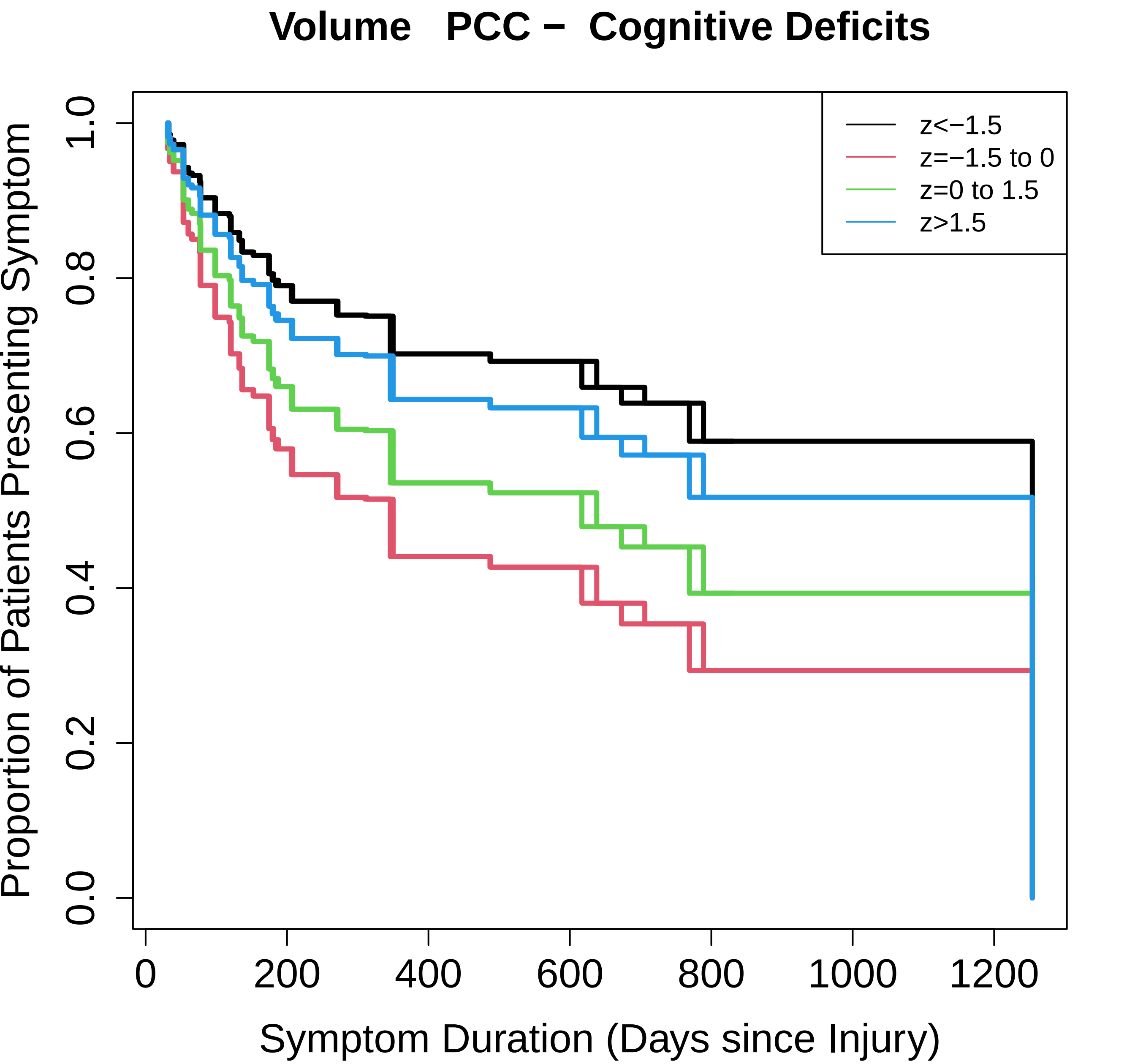

Volume PCC − Cognitive Deficits
Proportion of Patients Presenting Symptom
Symptom Duration (Days since Injury)
z<−1.5
z=−1.5 to 0
z=0 to 1.5
z>1.5
0.0
0.2
0.4
0.6
0.8
1.0
0
200
400
600
800
1000
1200

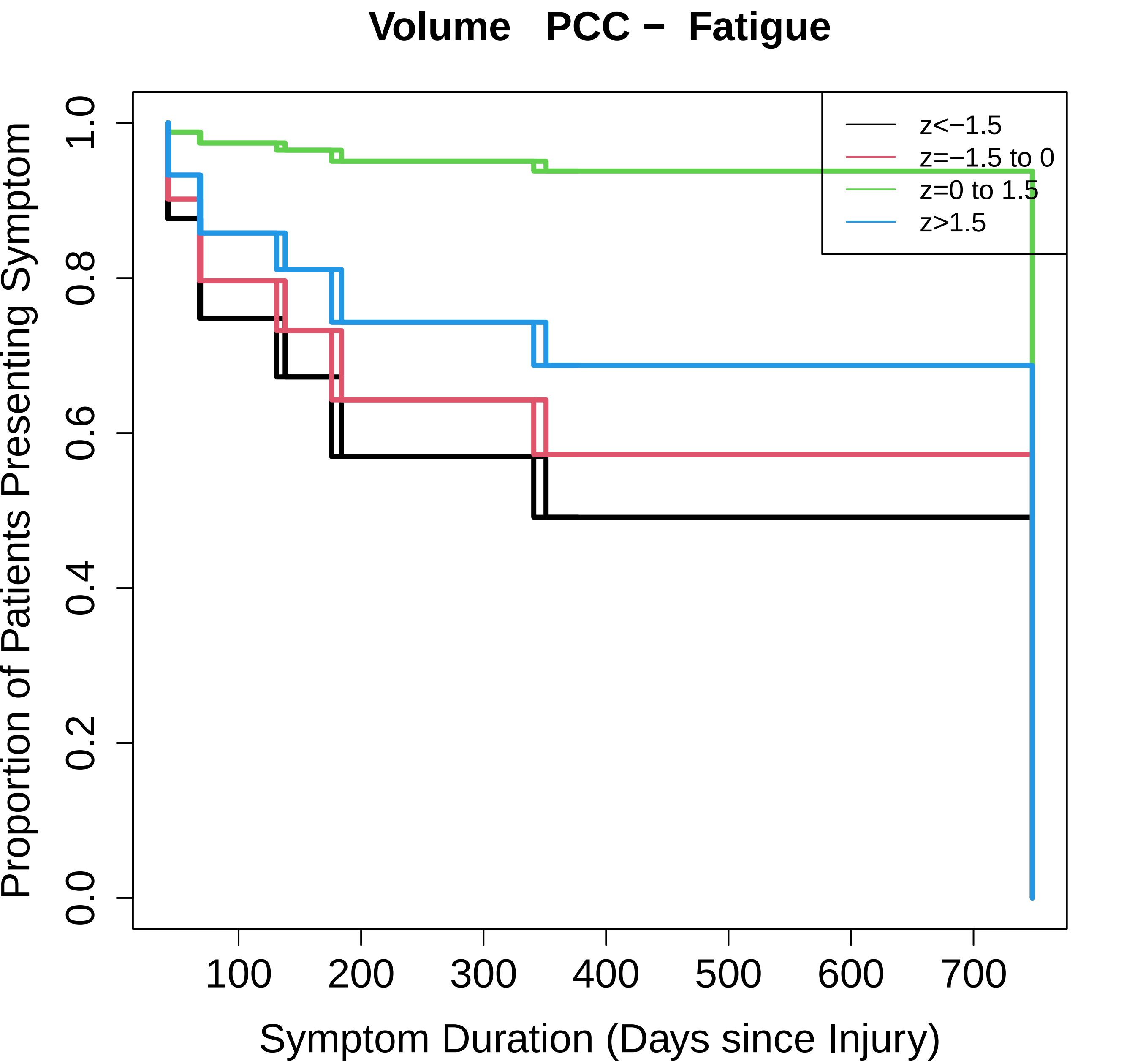
Volume PCC − Fatigue
Proportion of Patients Presenting Symptom
Symptom Duration (Days since Injury)
0.0
0.2
0.4
0.6
0.8
1.0
100
200
300
400
500
600
700
z<−1.5
z=−1.5 to 0
z=0 to 1.5
z>1.5

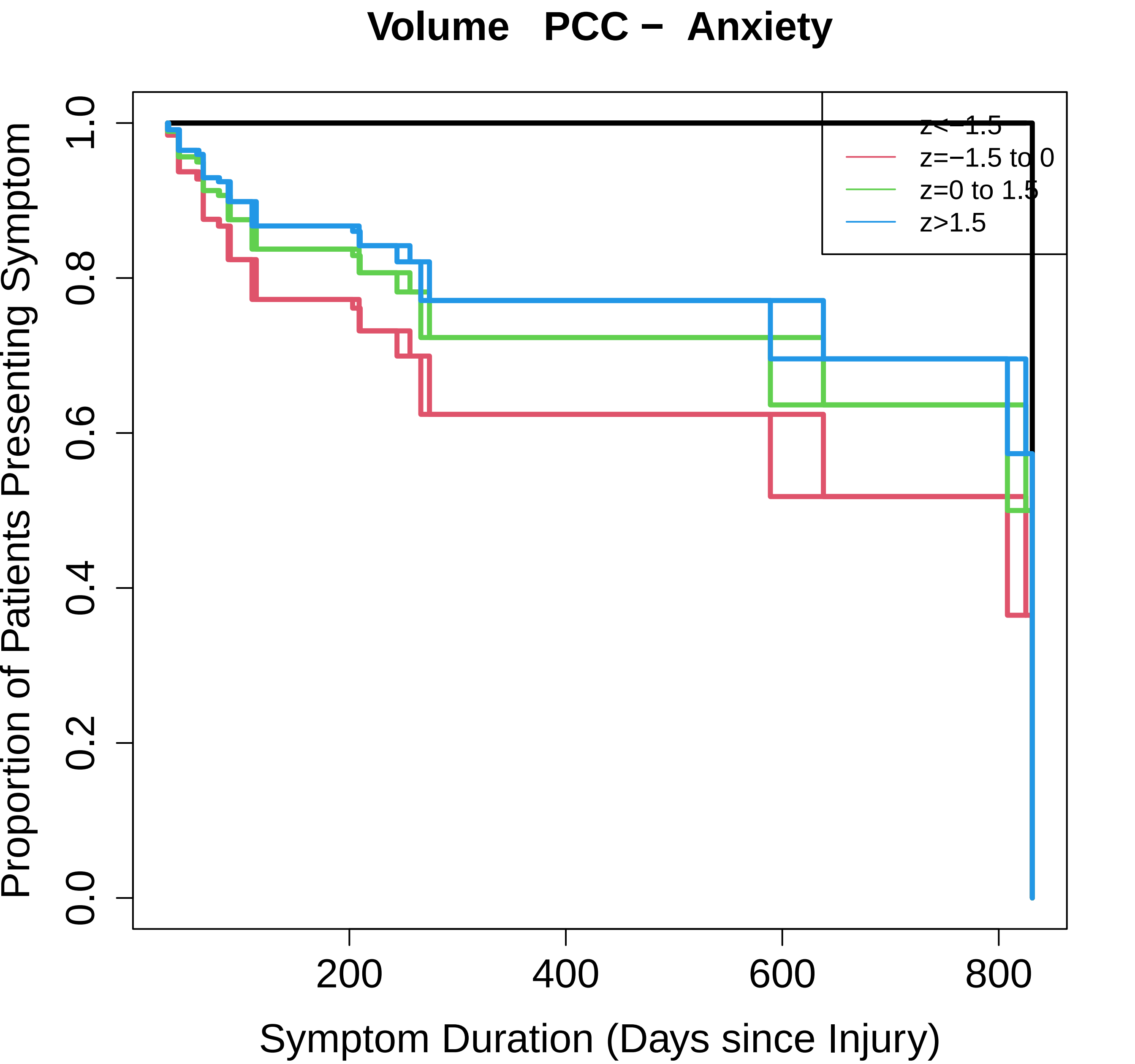

Volume PCC − Anxiety
z<−1.5
z=−1.5 to 0
z=0 to 1.5
z>1.5
Proportion of Patients Presenting Symptom
0.0
0.2
0.4
0.6
0.8
1.0
200
400
600
800
Symptom Duration (Days since Injury)

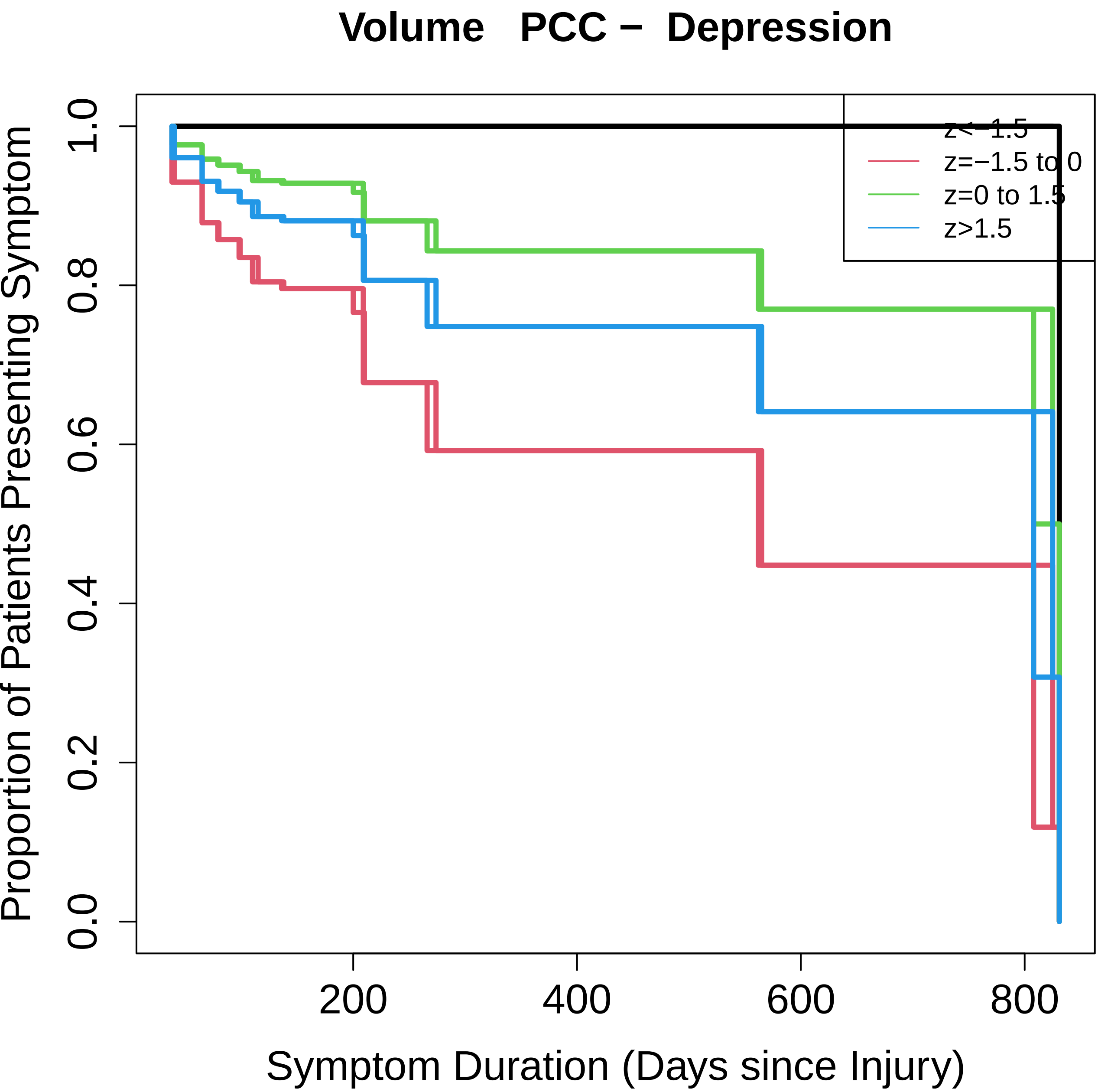

Volume PCC − Depression
Proportion of Patients Presenting Symptom
Symptom Duration (Days since Injury)
0.0
0.2
0.4
0.6
0.8
1.0
200
400
600
800
z<−1.5
z=−1.5 to 0
z=0 to 1.5
z>1.5

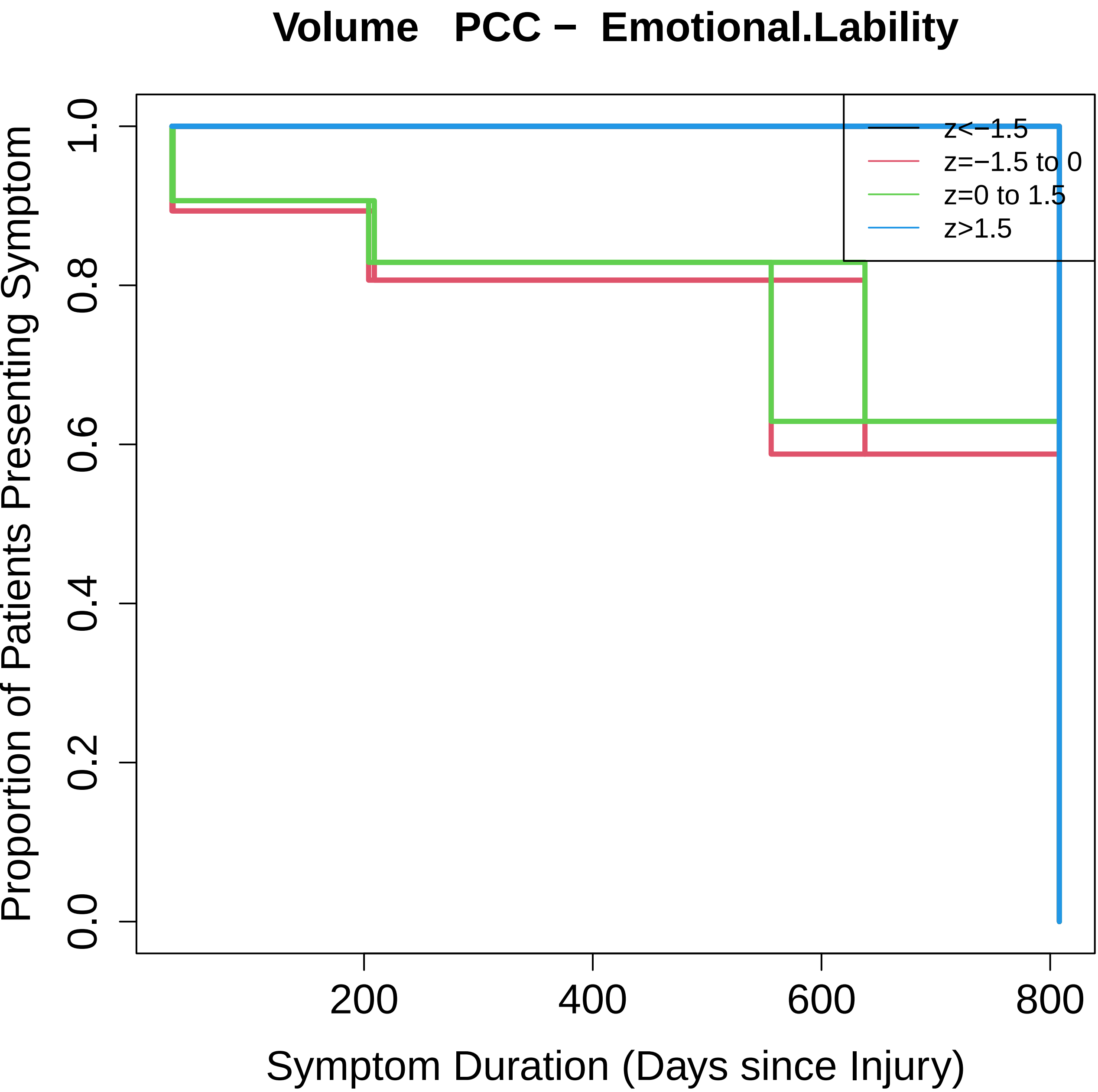

Volume PCC − Emotional.Lability
Proportion of Patients Presenting Symptom
Symptom Duration (Days since Injury)
0.0
0.2
0.4
0.6
0.8
1.0
200
400
600
800
z<−1.5
z=−1.5 to 0
z=0 to 1.5
z>1.5

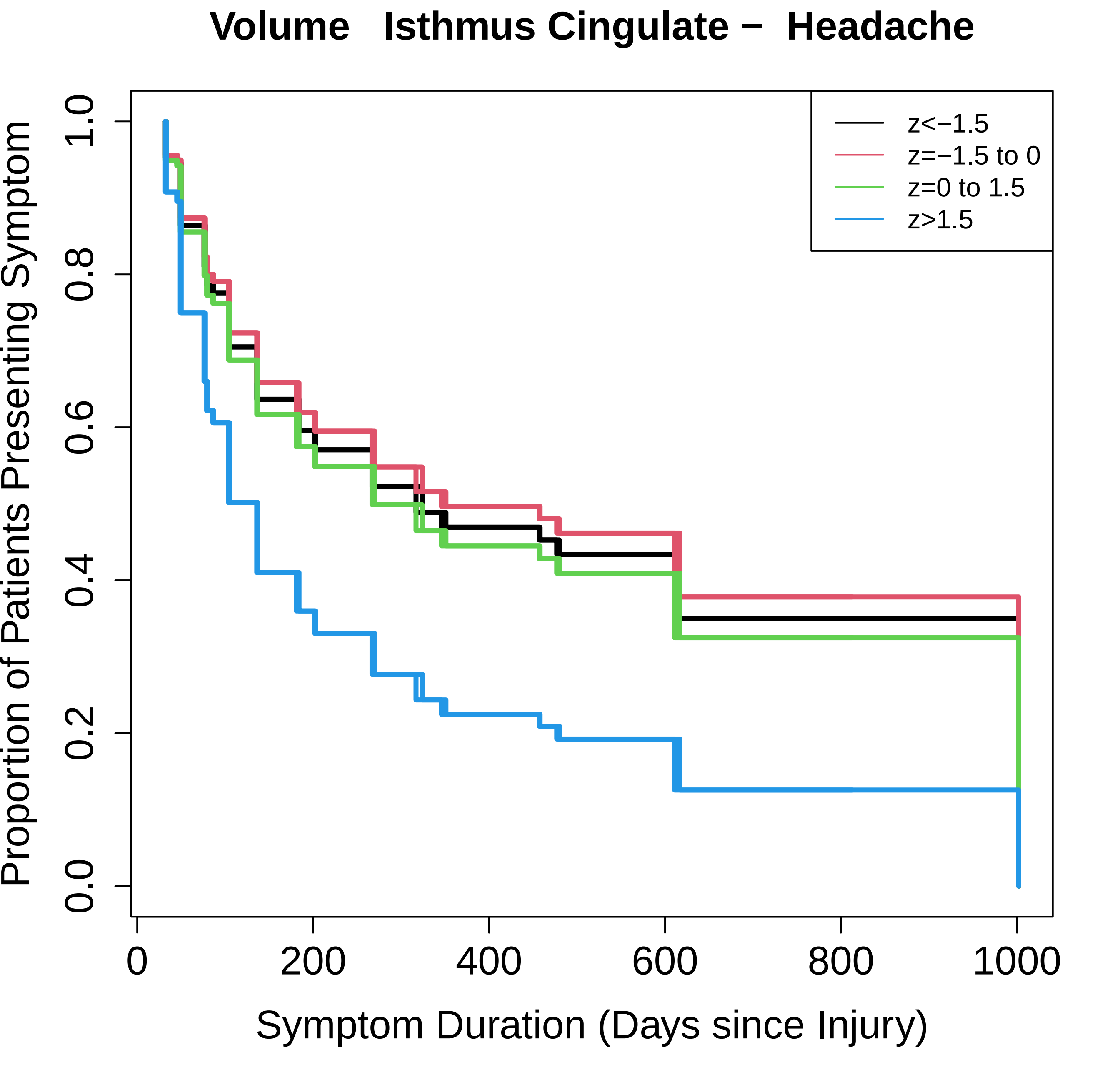

Volume Isthmus Cingulate − Headache
z<−1.5
z=−1.5 to 0
z=0 to 1.5
z>1.5
Proportion of Patients Presenting Symptom
0.0
0.2
0.4
0.6
0.8
1.0
0
200
400
600
800
1000
Symptom Duration (Days since Injury)

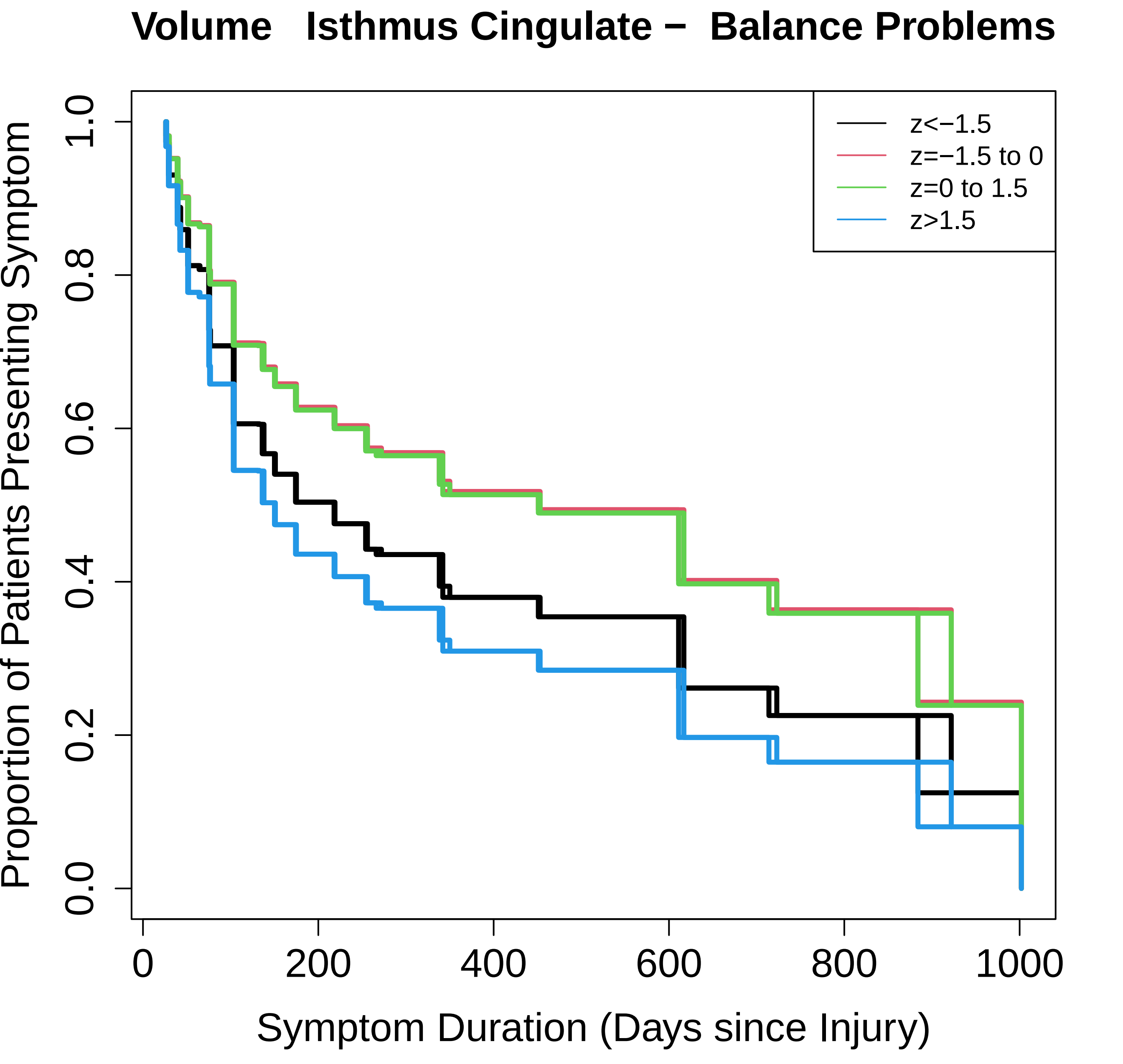

Volume Isthmus Cingulate − Balance Problems
Proportion of Patients Presenting Symptom
0.0
0.2
0.4
0.6
0.8
1.0
0
200
400
600
800
1000
Symptom Duration (Days since Injury)
z<−1.5
z=−1.5 to 0
z=0 to 1.5
z>1.5

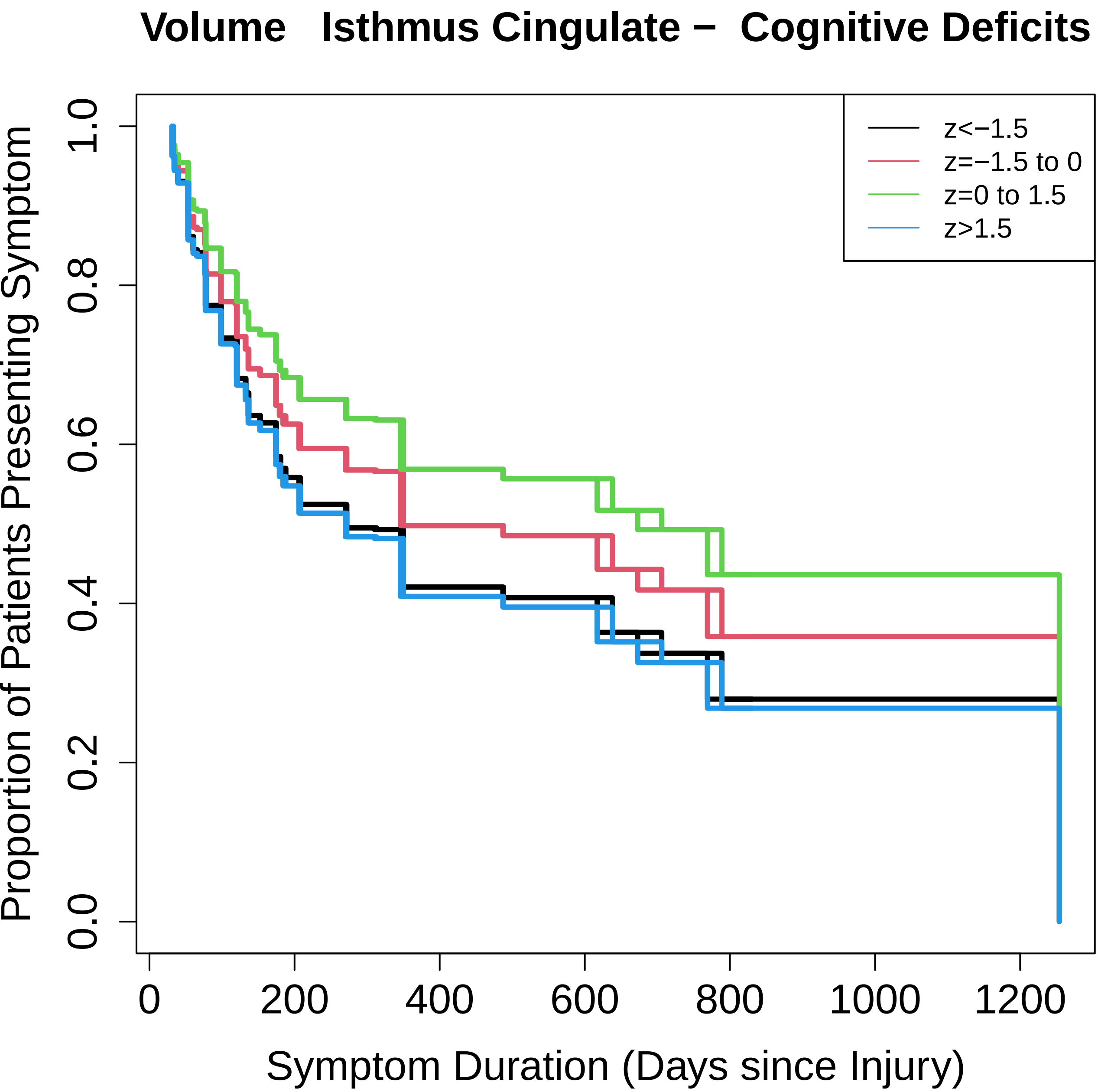
Volume Isthmus Cingulate − Cognitive Deficits
z<−1.5
z=−1.5 to 0
z=0 to 1.5
z>1.5
Proportion of Patients Presenting Symptom
0.0
0.2
0.4
0.6
0.8
1.0
0
200
400
600
800
1000
1200
Symptom Duration (Days since Injury)

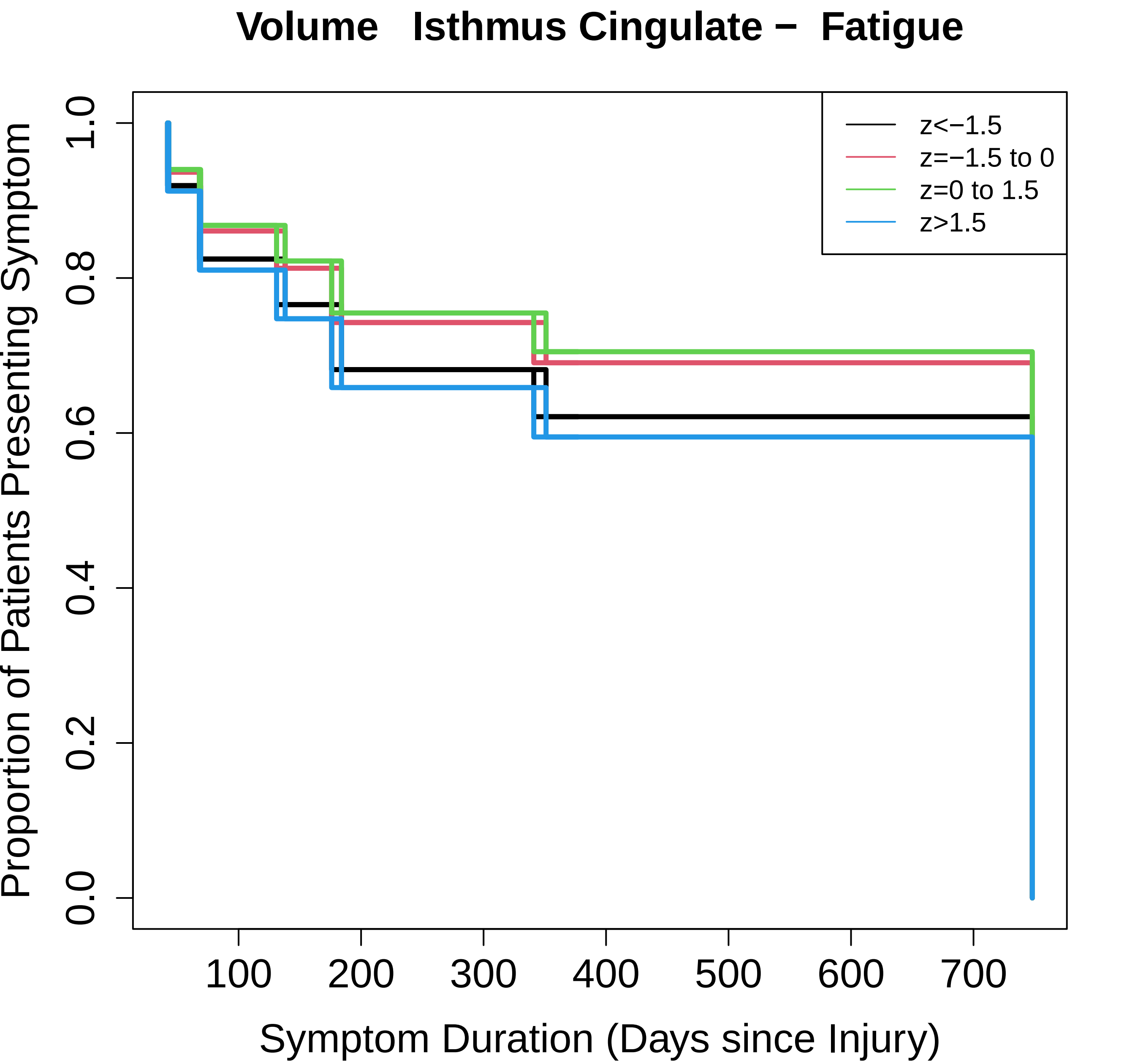

Volume Isthmus Cingulate − Fatigue
Proportion of Patients Presenting Symptom
0.0
0.2
0.4
0.6
0.8
1.0
100
200
300
400
500
600
700
Symptom Duration (Days since Injury)
z<−1.5
z=−1.5 to 0
z=0 to 1.5
z>1.5

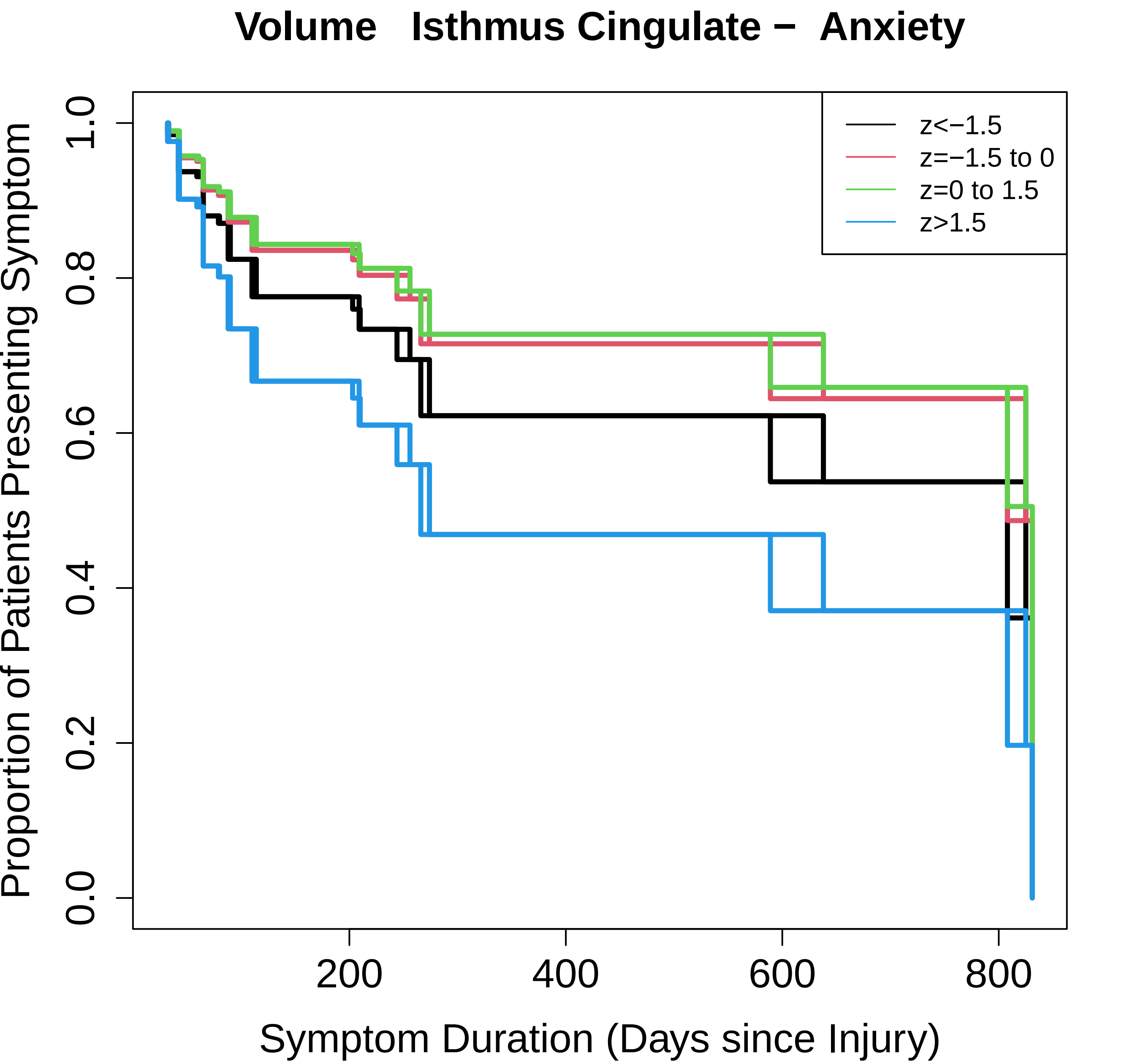
Volume Isthmus Cingulate − Anxiety
z<−1.5
z=−1.5 to 0
z=0 to 1.5
z>1.5
Proportion of Patients Presenting Symptom
0.0
0.2
0.4
0.6
0.8
1.0
200
400
600
800
Symptom Duration (Days since Injury)

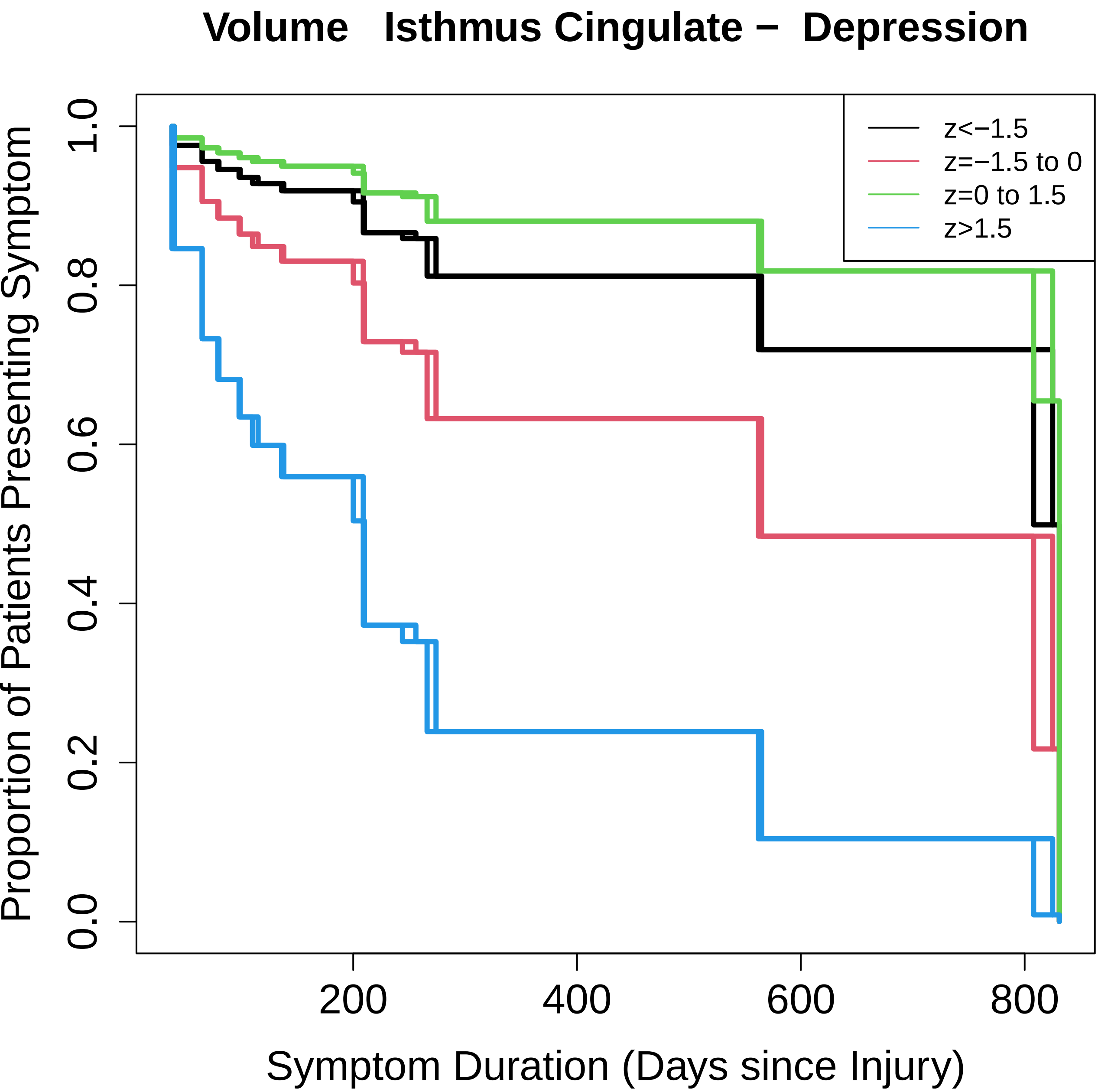

Volume Isthmus Cingulate − Depression
Proportion of Patients Presenting Symptom
Symptom Duration (Days since Injury)
0.0
0.2
0.4
0.6
0.8
1.0
200
400
600
800
z<−1.5
z=−1.5 to 0
z=0 to 1.5
z>1.5

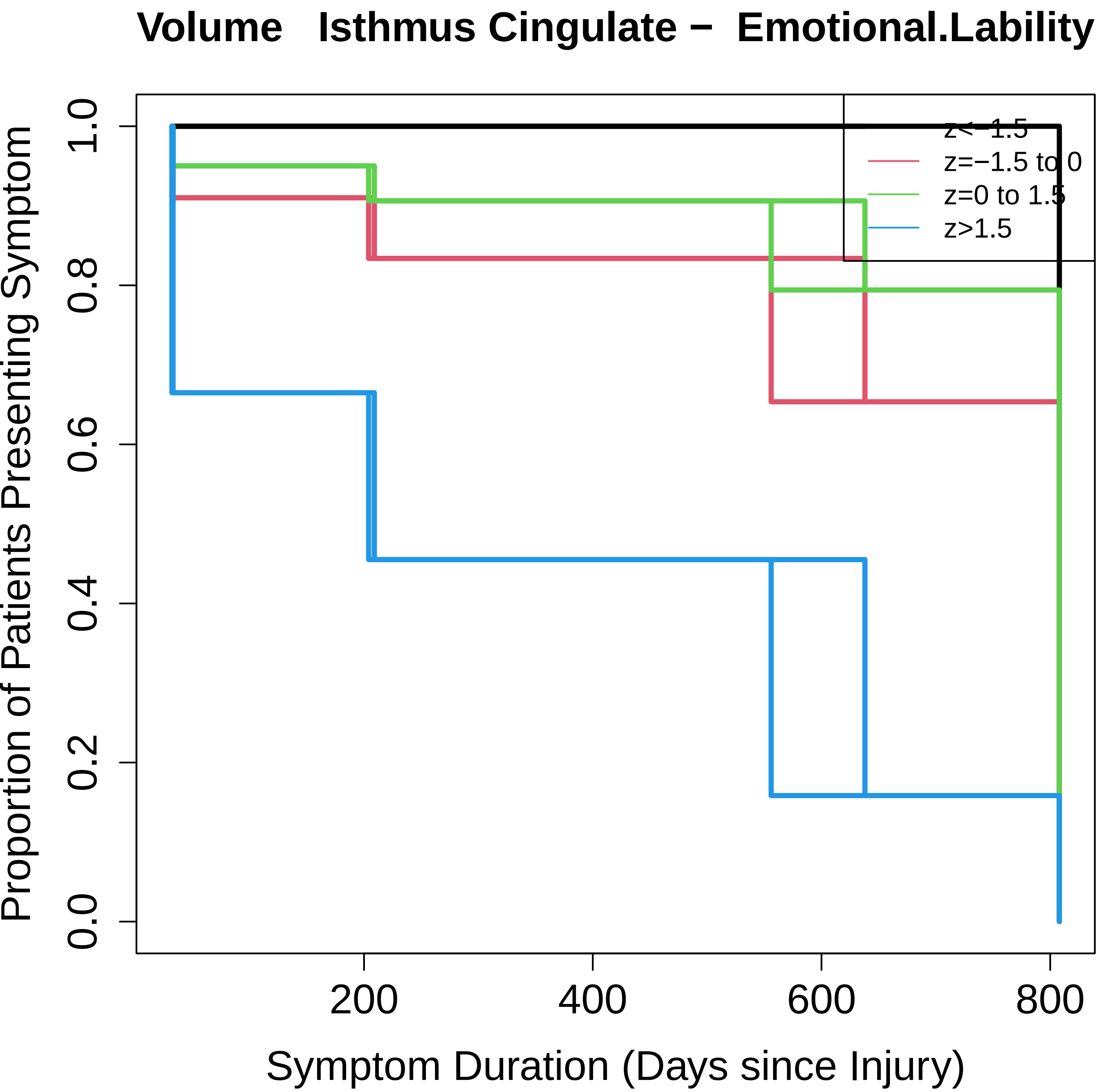
Volume Isthmus Cingulate − Emotional.Lability
z<−1.5
z=−1.5 to 0
z=0 to 1.5
z>1.5
1.0
0.8
0.6
0.4
0.2
0.0
200
400
600
800
Proportion of Patients Presenting Symptom
Symptom Duration (Days since Injury)

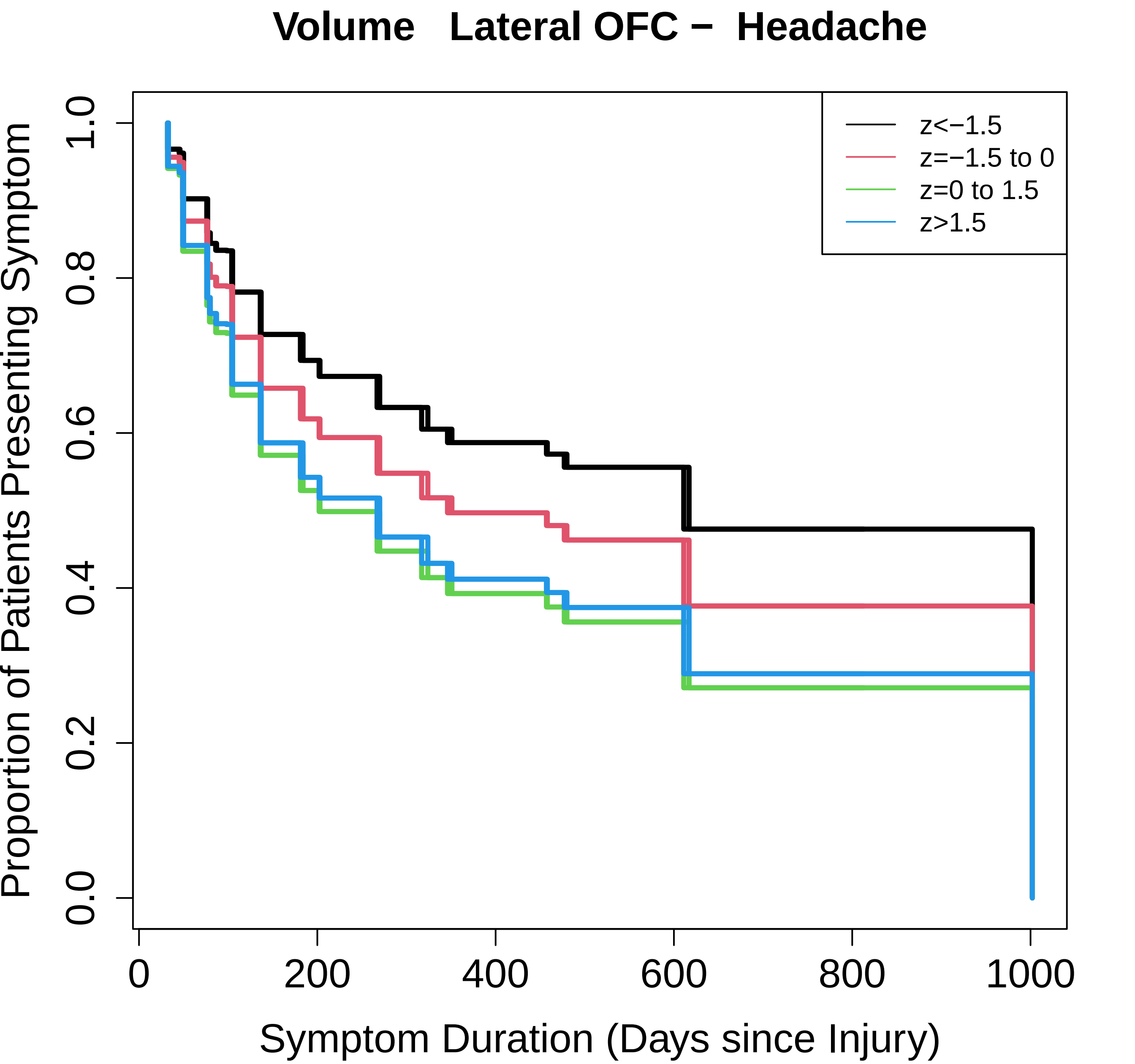

Volume Lateral OFC − Headache
z<−1.5
z=−1.5 to 0
z=0 to 1.5
z>1.5
Proportion of Patients Presenting Symptom
0.0
0.2
0.4
0.6
0.8
1.0
0
200
400
600
800
1000
Symptom Duration (Days since Injury)

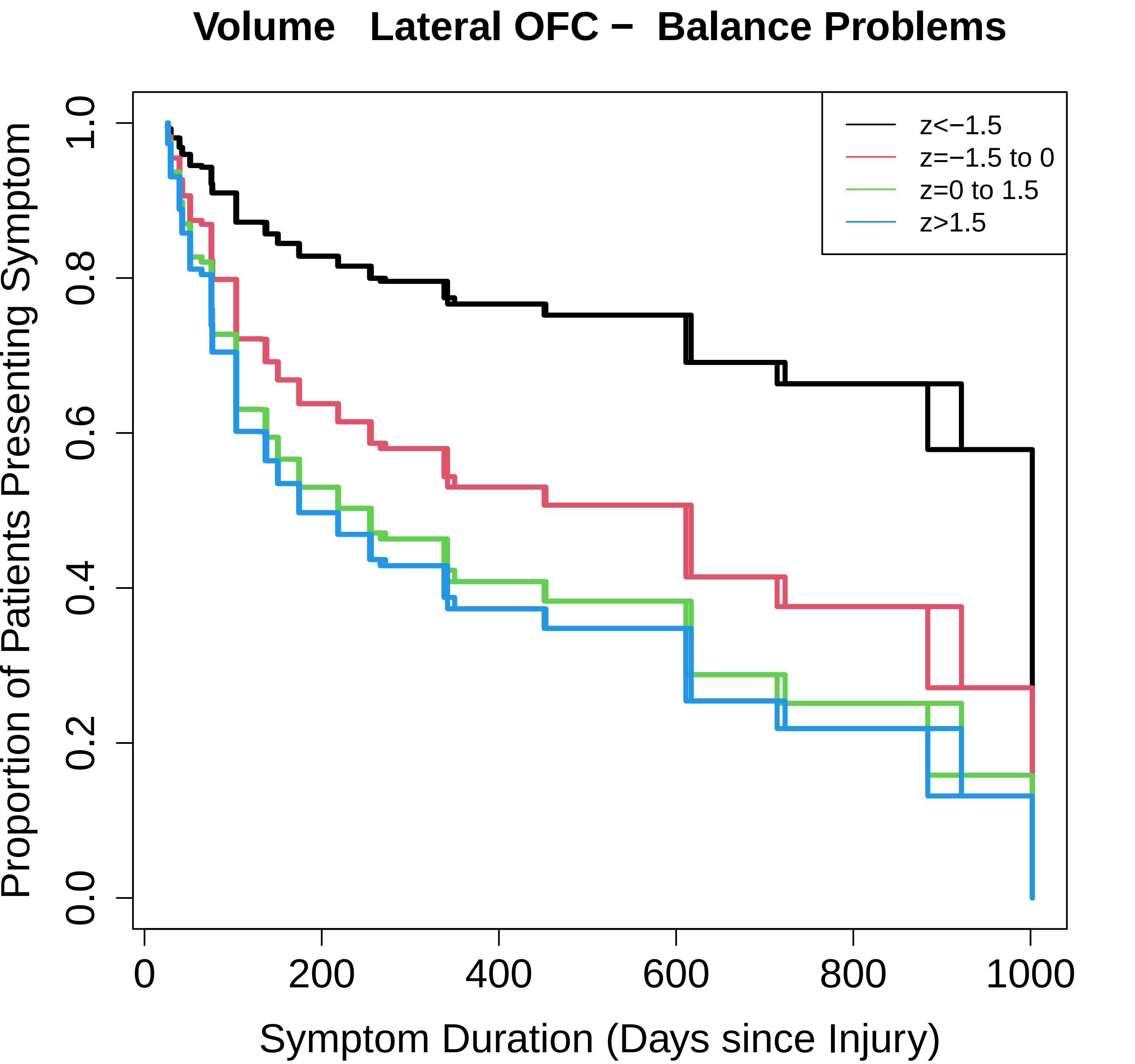

Volume Lateral OFC − Balance Problems
z<−1.5
z=−1.5 to 0
z=0 to 1.5
z>1.5
Proportion of Patients Presenting Symptom
0.0
0.2
0.4
0.6
0.8
1.0
0
200
400
600
800
1000
Symptom Duration (Days since Injury)

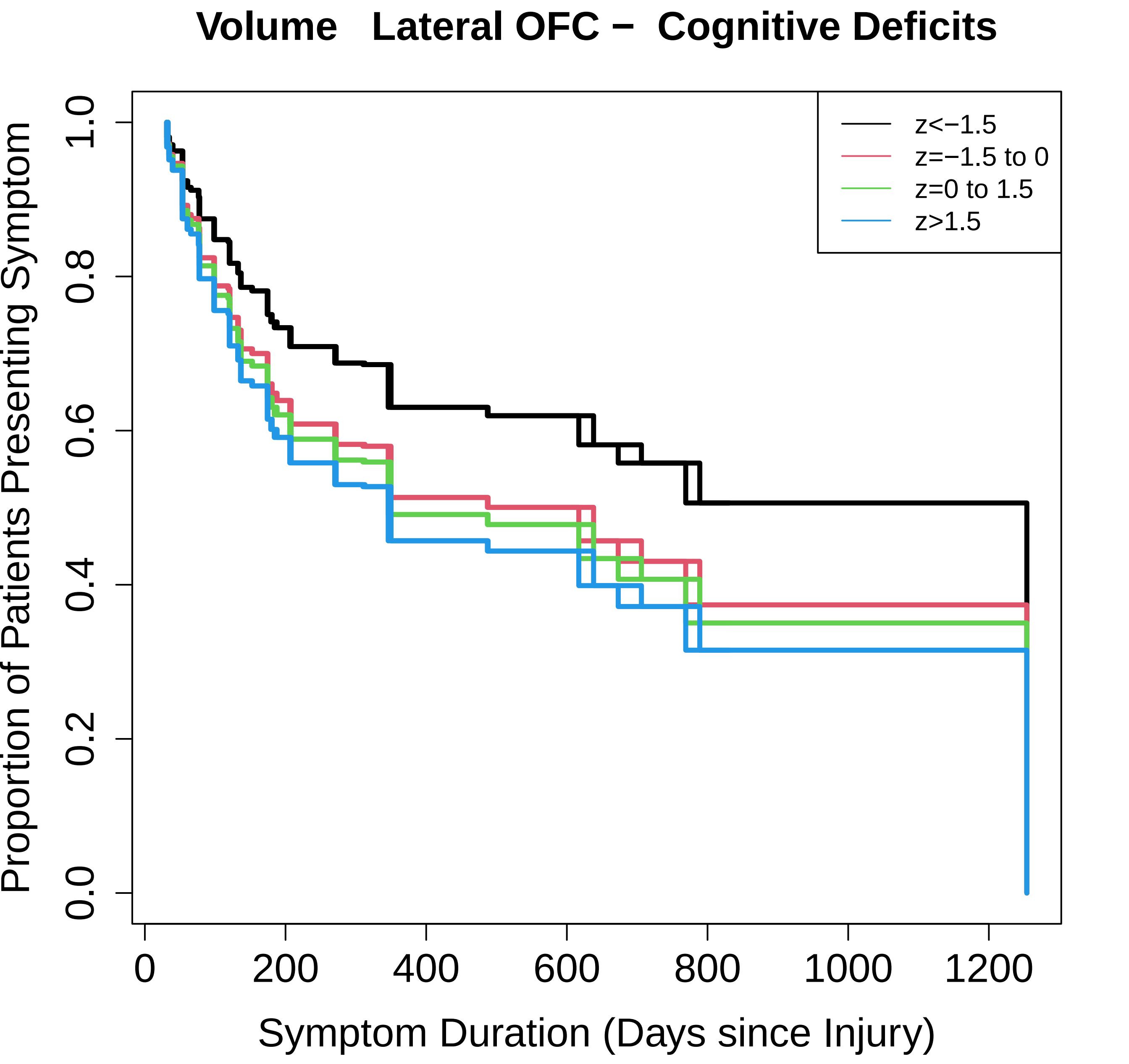
Volume Lateral OFC − Cognitive Deficits
z<−1.5
z=−1.5 to 0
z=0 to 1.5
z>1.5
Proportion of Patients Presenting Symptom
0.0
0.2
0.4
0.6
0.8
1.0
0
200
400
600
800
1000
1200
Symptom Duration (Days since Injury)

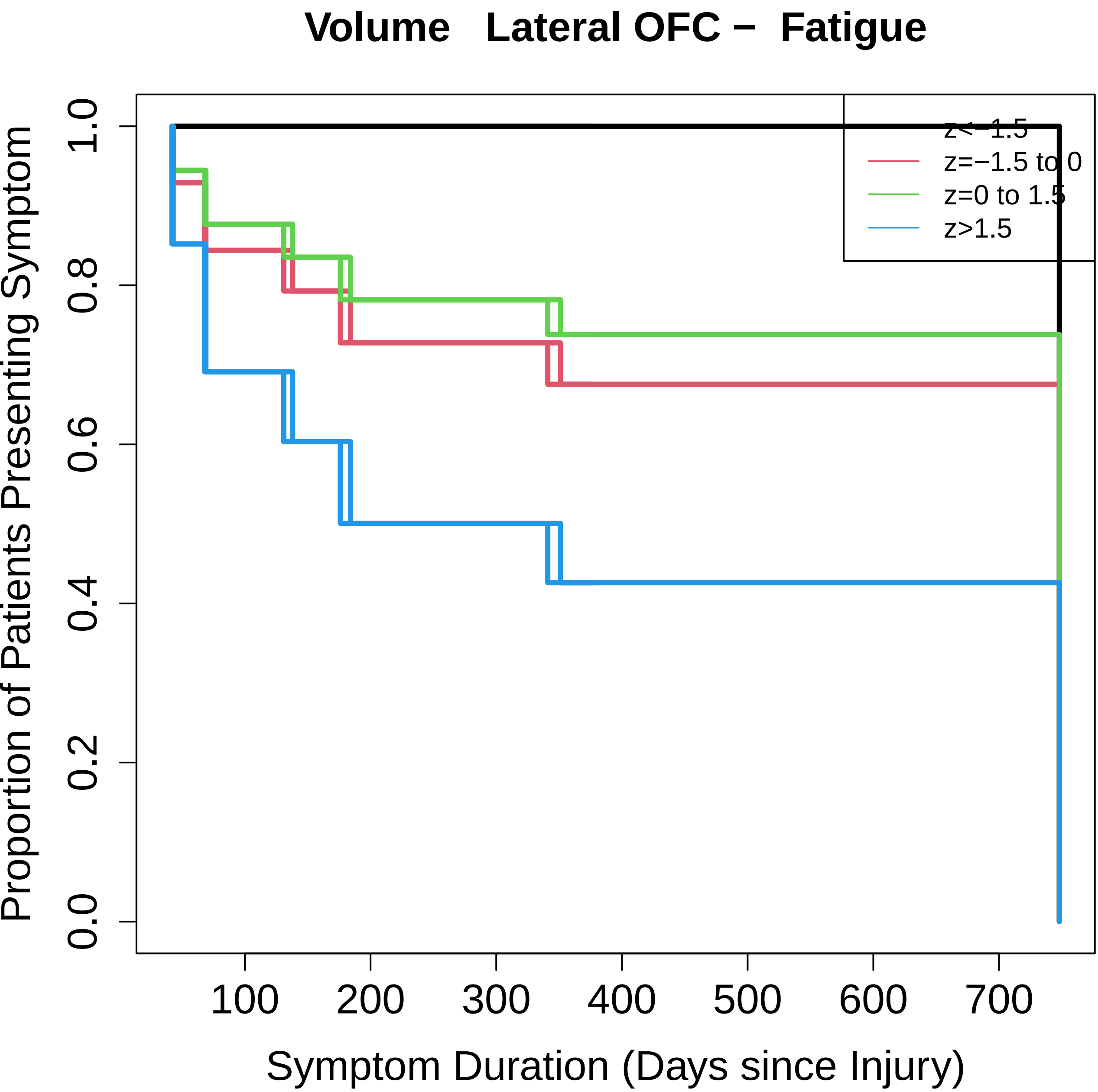
Volume Lateral OFC − Fatigue
z<−1.5
z=−1.5 to 0
z=0 to 1.5
z>1.5
0.0
0.2
0.4
0.6
0.8
1.0
100
200
300
400
500
600
700
Proportion of Patients Presenting Symptom
Symptom Duration (Days since Injury)

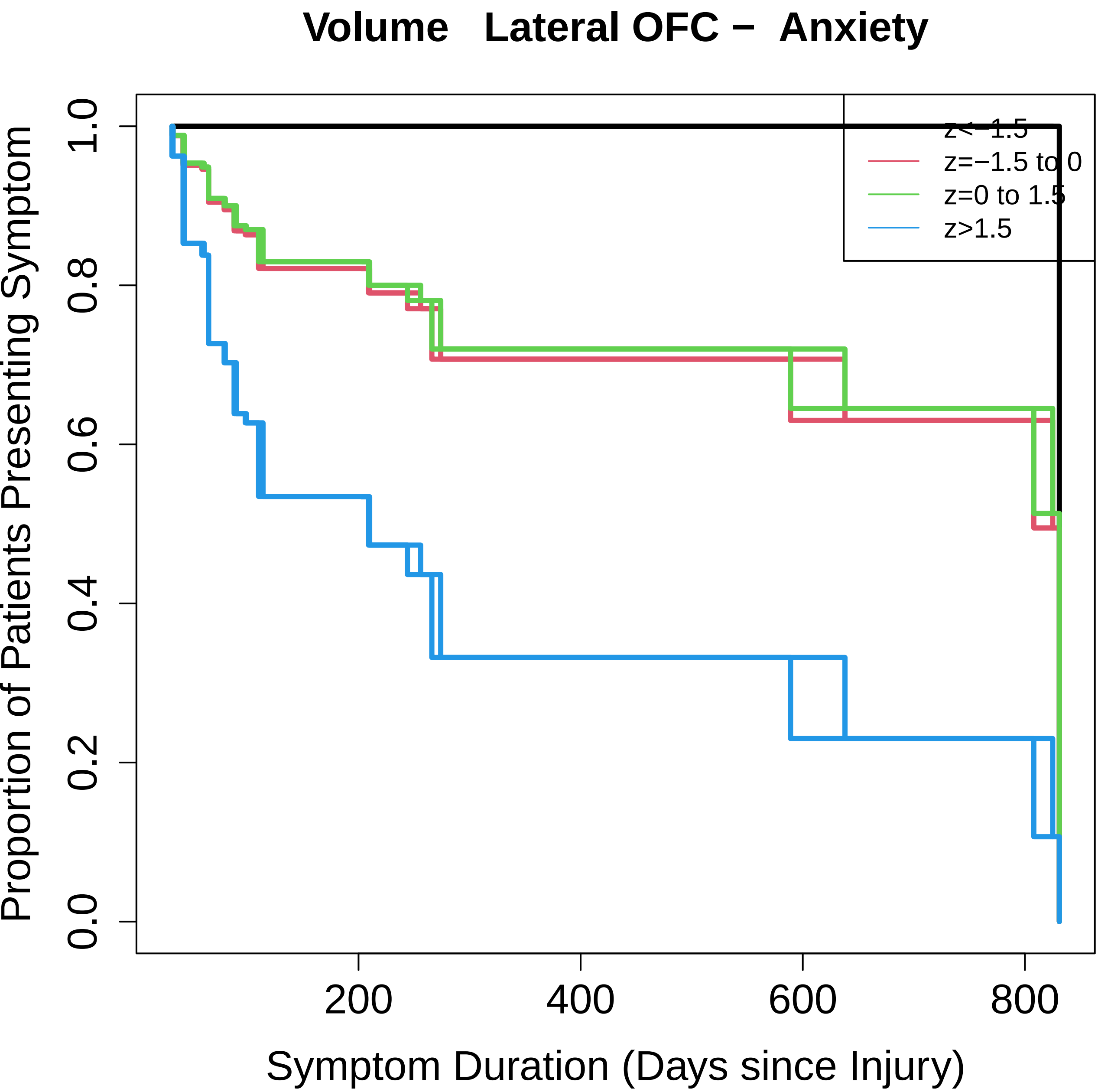
Volume Lateral OFC − Anxiety
z<−1.5
z=−1.5 to 0
z=0 to 1.5
z>1.5
Proportion of Patients Presenting Symptom
0.0
0.2
0.4
0.6
0.8
1.0
200
400
600
800
Symptom Duration (Days since Injury)

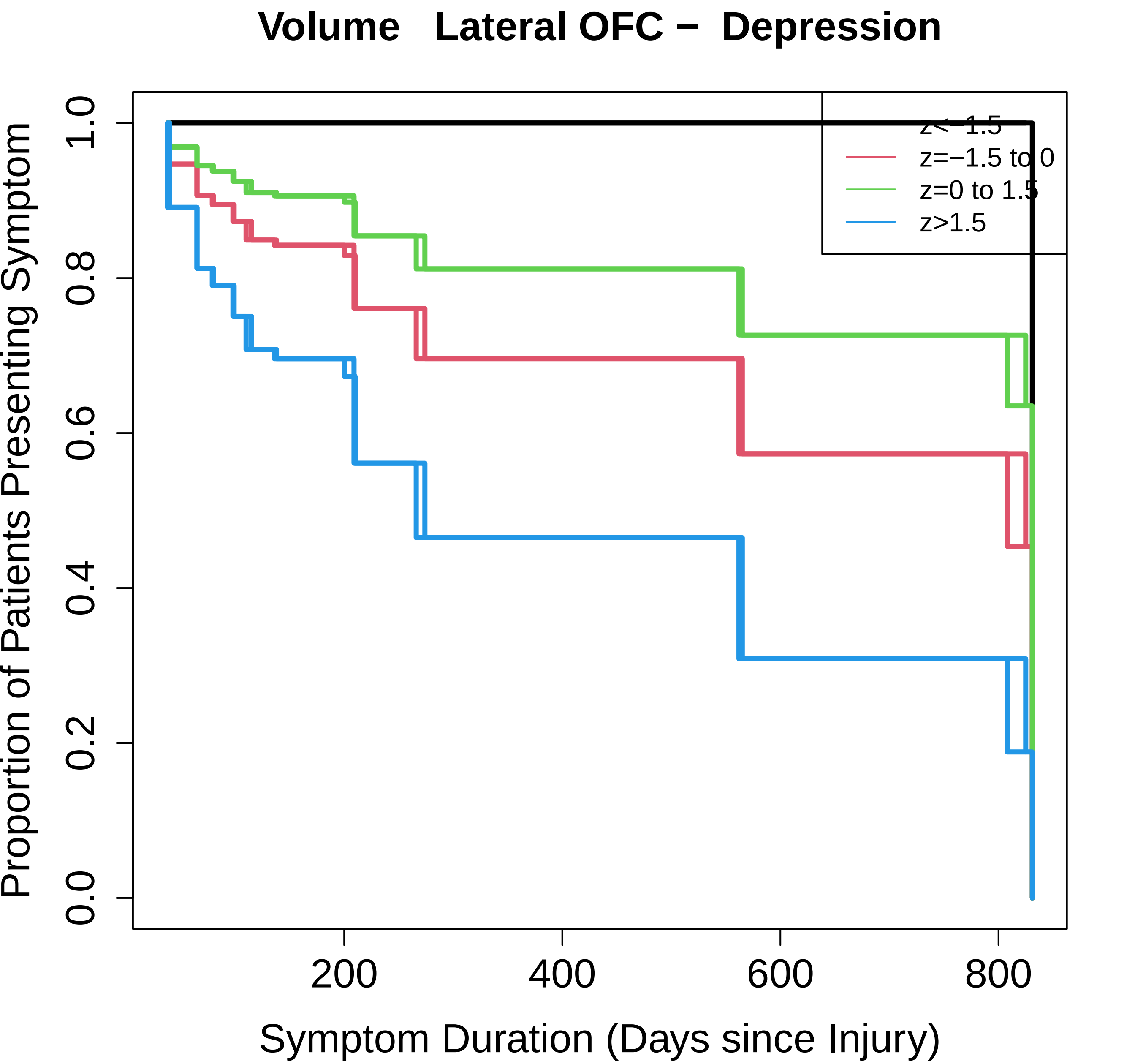

Volume Lateral OFC − Depression
Proportion of Patients Presenting Symptom
Symptom Duration (Days since Injury)
1.0
0.8
0.6
0.4
0.2
0.0
200
400
600
800
z<−1.5
z=−1.5 to 0
z=0 to 1.5
z>1.5

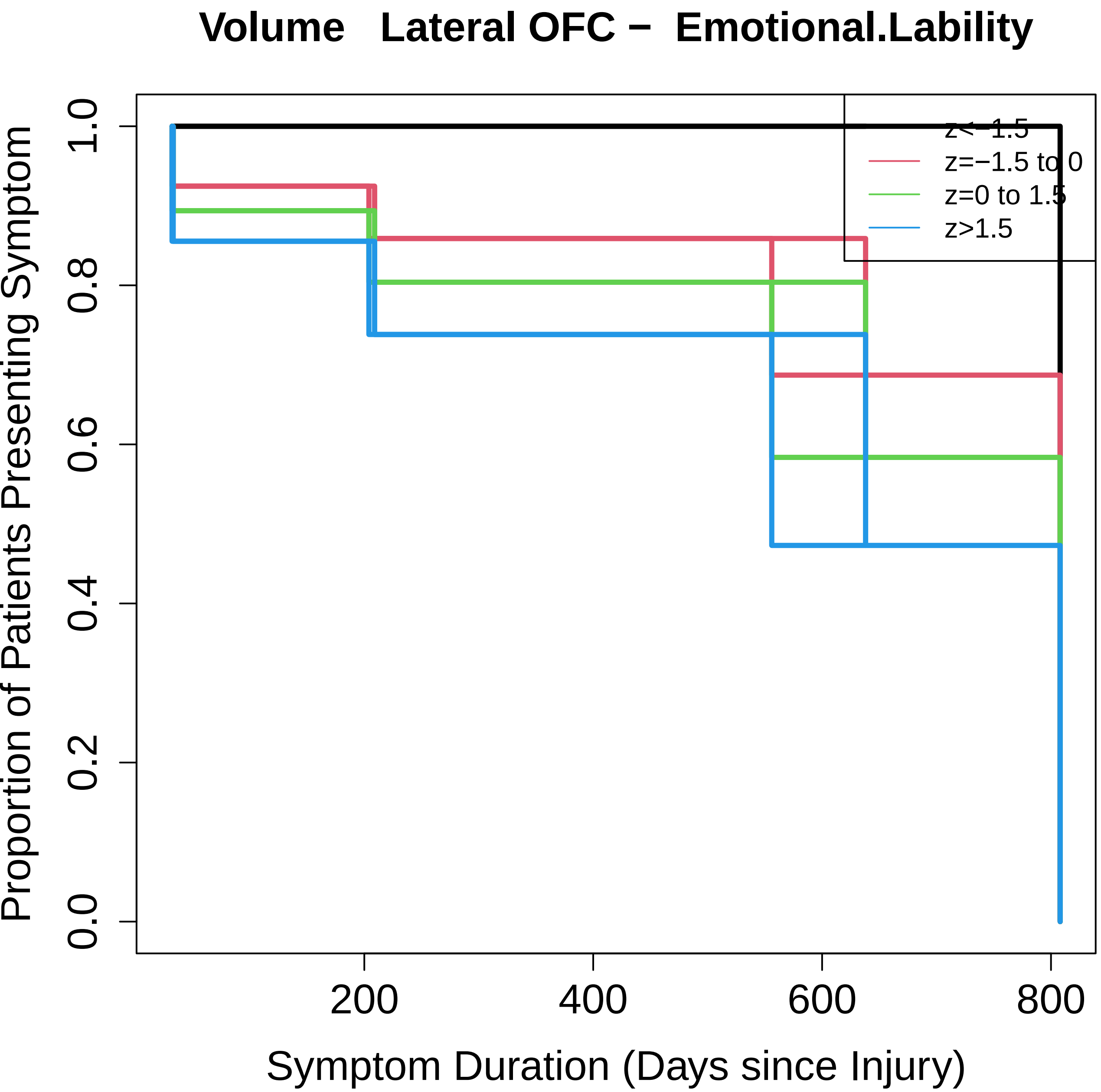

Volume Lateral OFC − Emotional.Lability
Proportion of Patients Presenting Symptom
Symptom Duration (Days since Injury)
0.0
0.2
0.4
0.6
0.8
1.0
200
400
600
800
z<−1.5
z=−1.5 to 0
z=0 to 1.5
z>1.5

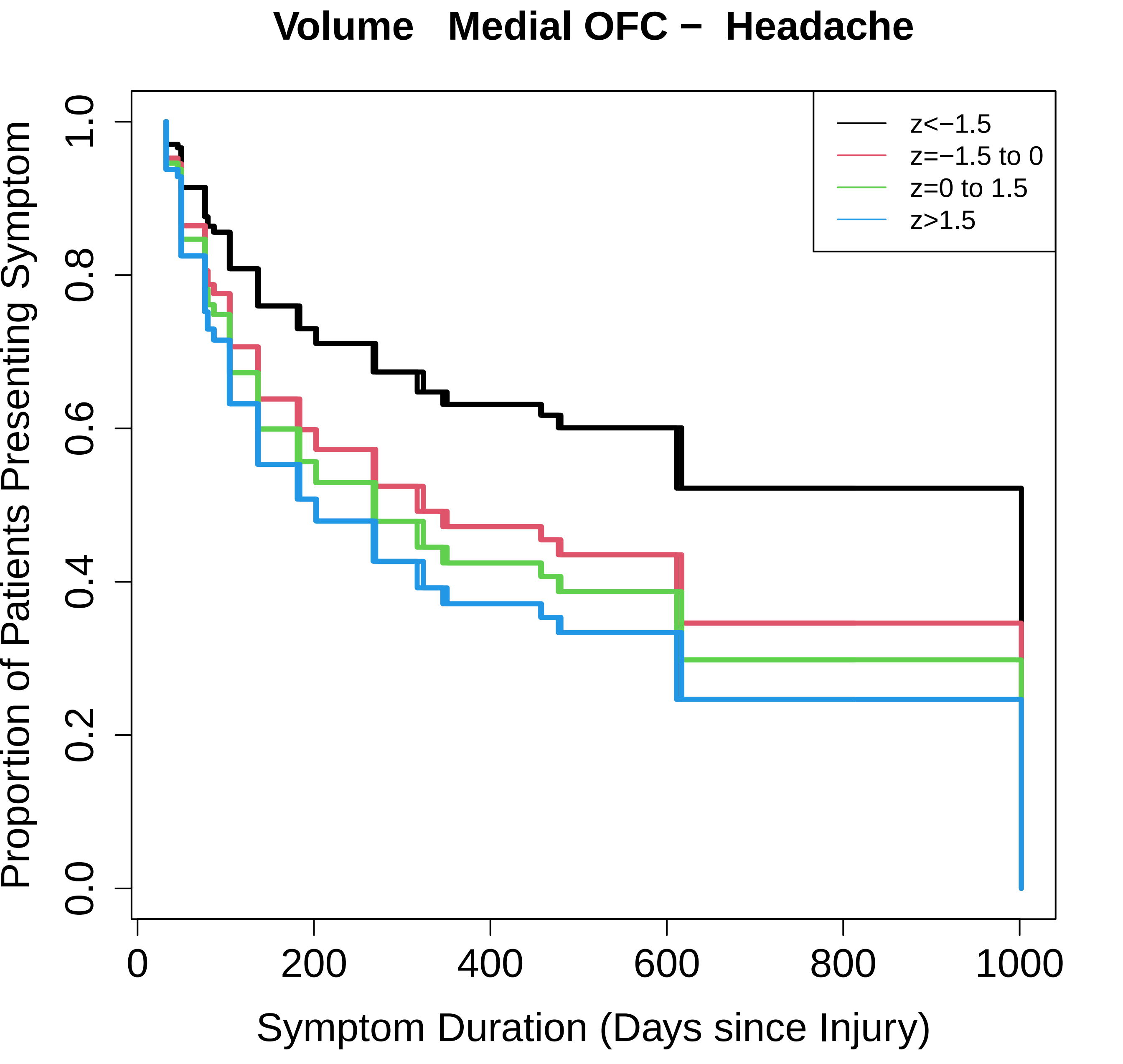

Volume Medial OFC − Headache
Proportion of Patients Presenting Symptom
Symptom Duration (Days since Injury)
z<−1.5
z=−1.5 to 0
z=0 to 1.5
z>1.5
0.0
0.2
0.4
0.6
0.8
1.0
0
200
400
600
800
1000

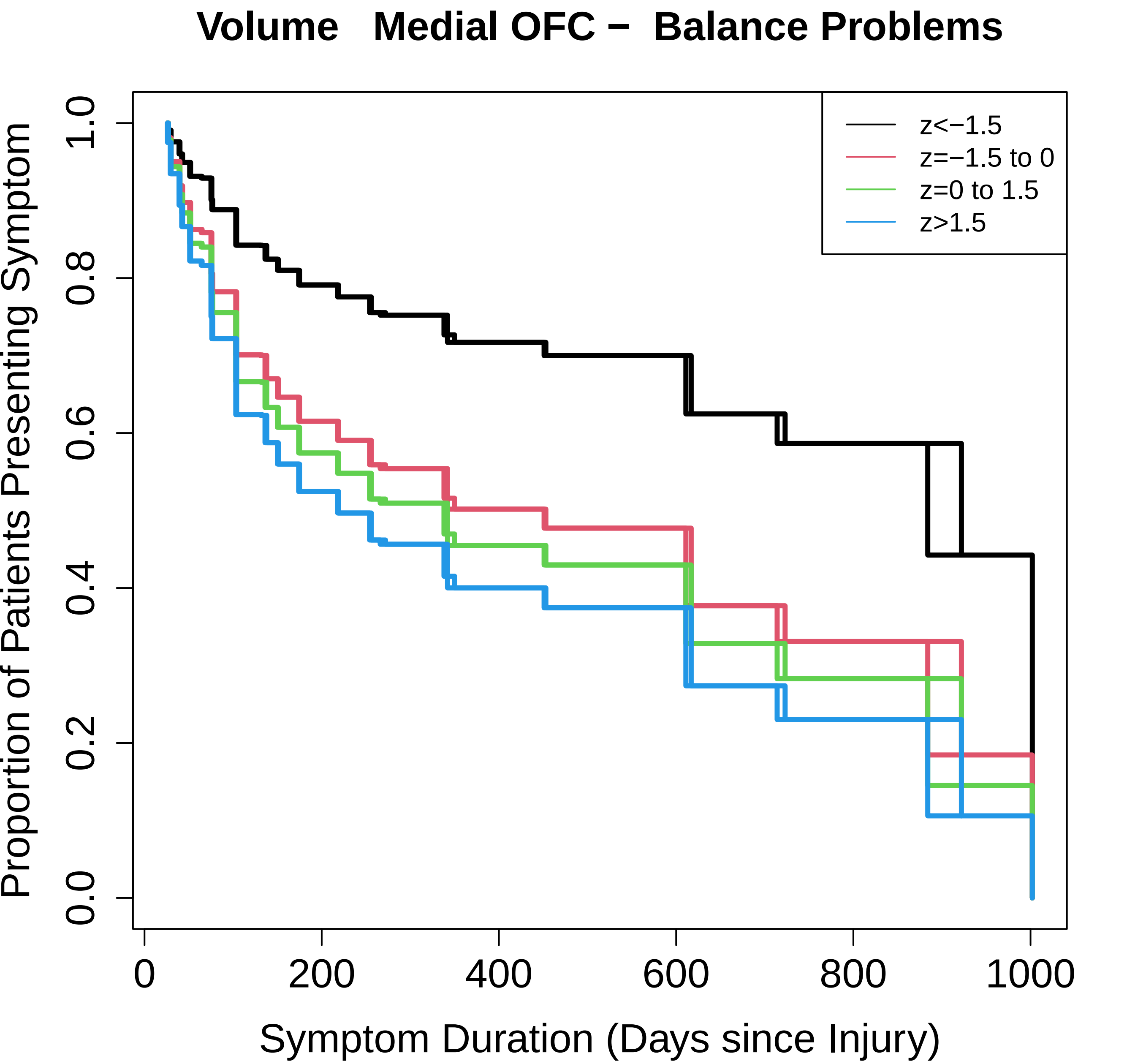

Volume Medial OFC − Balance Problems
z<−1.5
z=−1.5 to 0
z=0 to 1.5
z>1.5
Proportion of Patients Presenting Symptom
0.0
0.2
0.4
0.6
0.8
1.0
0
200
400
600
800
1000
Symptom Duration (Days since Injury)

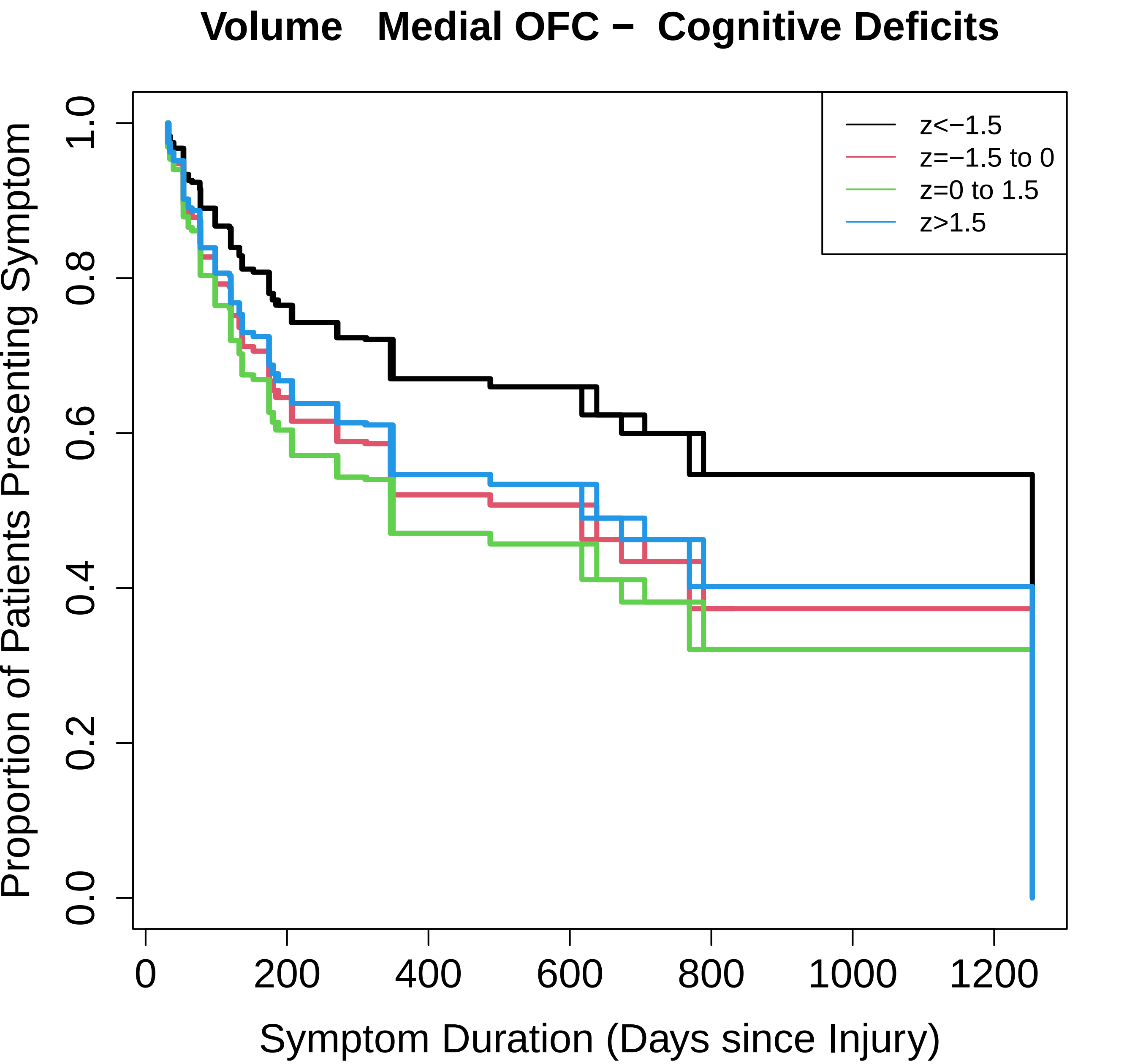

Volume Medial OFC − Cognitive Deficits
z<−1.5
z=−1.5 to 0
z=0 to 1.5
z>1.5
Proportion of Patients Presenting Symptom
0.0
0.2
0.4
0.6
0.8
1.0
0
200
400
600
800
1000
1200
Symptom Duration (Days since Injury)

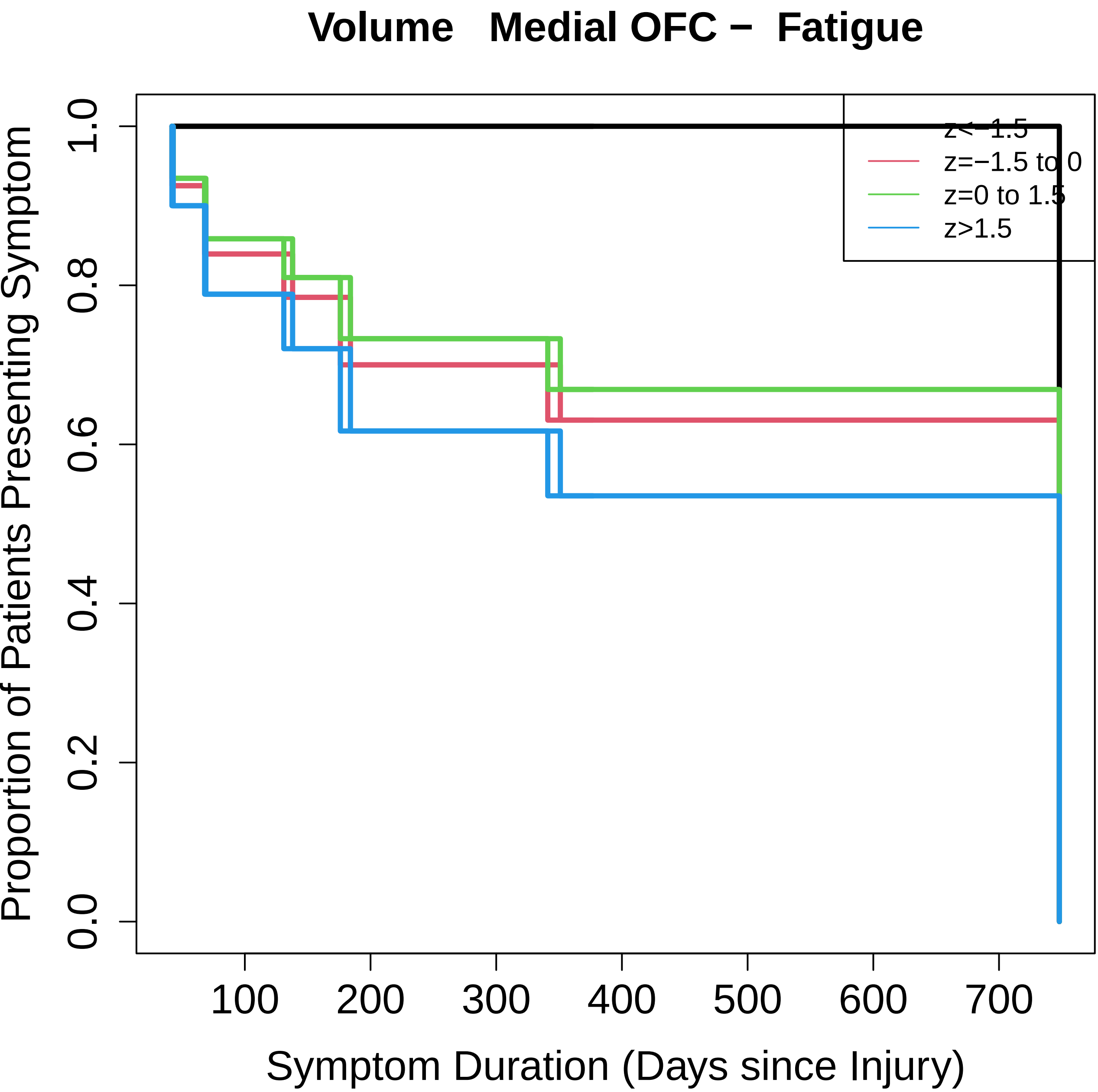

Volume Medial OFC − Fatigue
Proportion of Patients Presenting Symptom
0.0
0.2
0.4
0.6
0.8
1.0
100
200
300
400
500
600
700
Symptom Duration (Days since Injury)
z<−1.5
z=−1.5 to 0
z=0 to 1.5
z>1.5

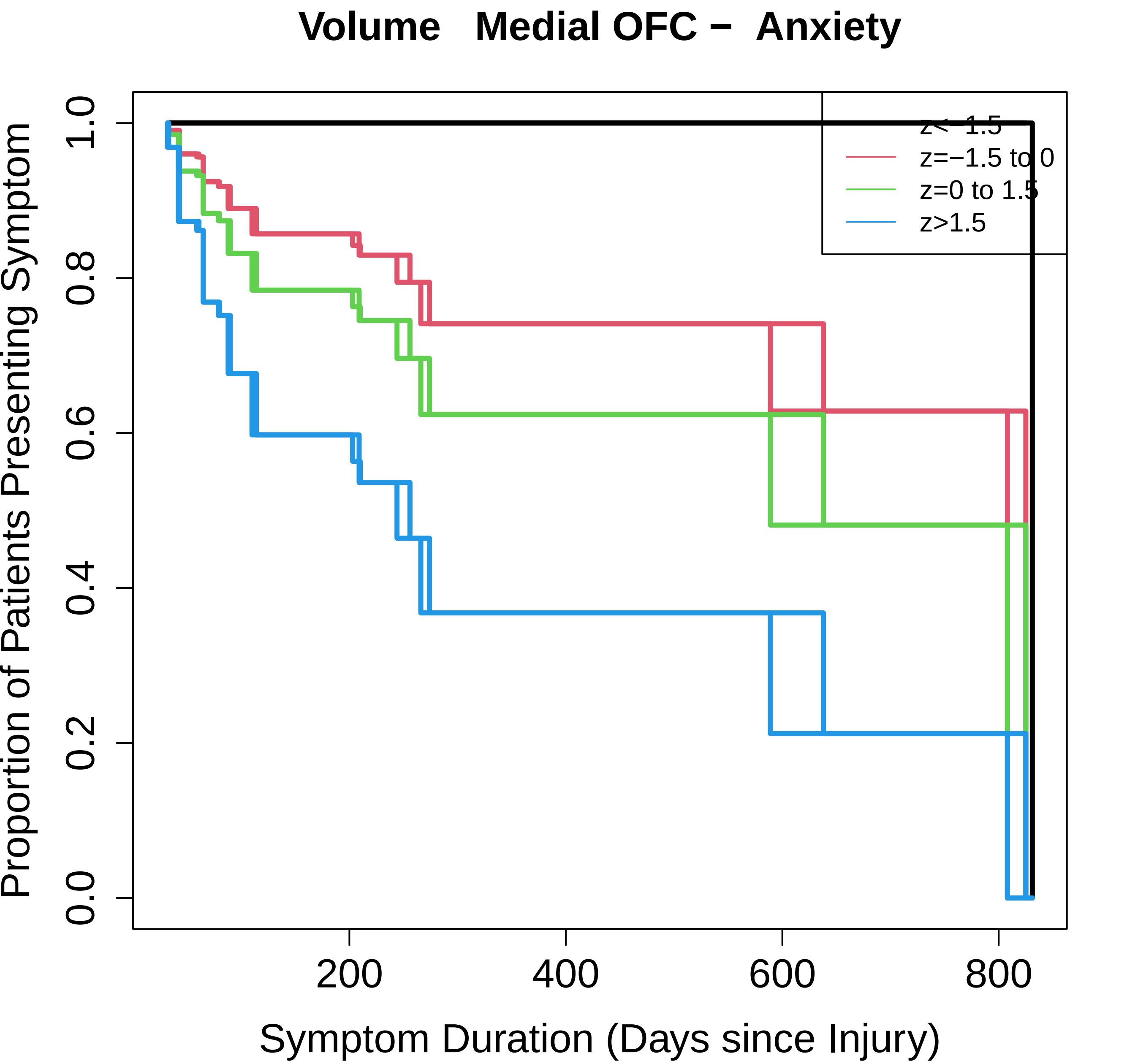
Volume Medial OFC − Anxiety
z<−1.5
z=−1.5 to 0
z=0 to 1.5
z>1.5
Proportion of Patients Presenting Symptom
0.0
0.2
0.4
0.6
0.8
1.0
200
400
600
800
Symptom Duration (Days since Injury)

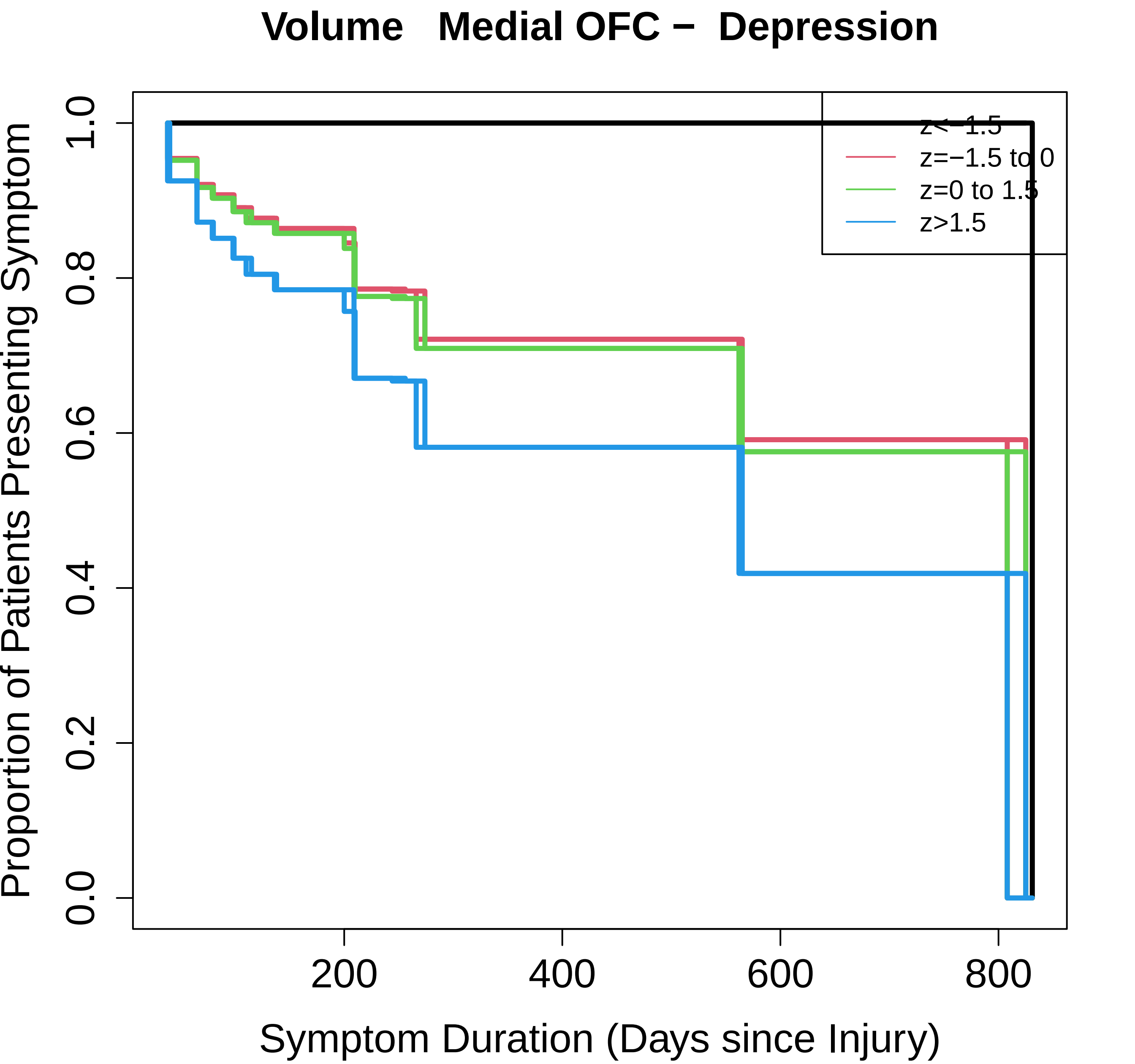

Volume Medial OFC − Depression
Proportion of Patients Presenting Symptom
Symptom Duration (Days since Injury)
0.0
0.2
0.4
0.6
0.8
1.0
200
400
600
800
z<−1.5
z=−1.5 to 0
z=0 to 1.5
z>1.5

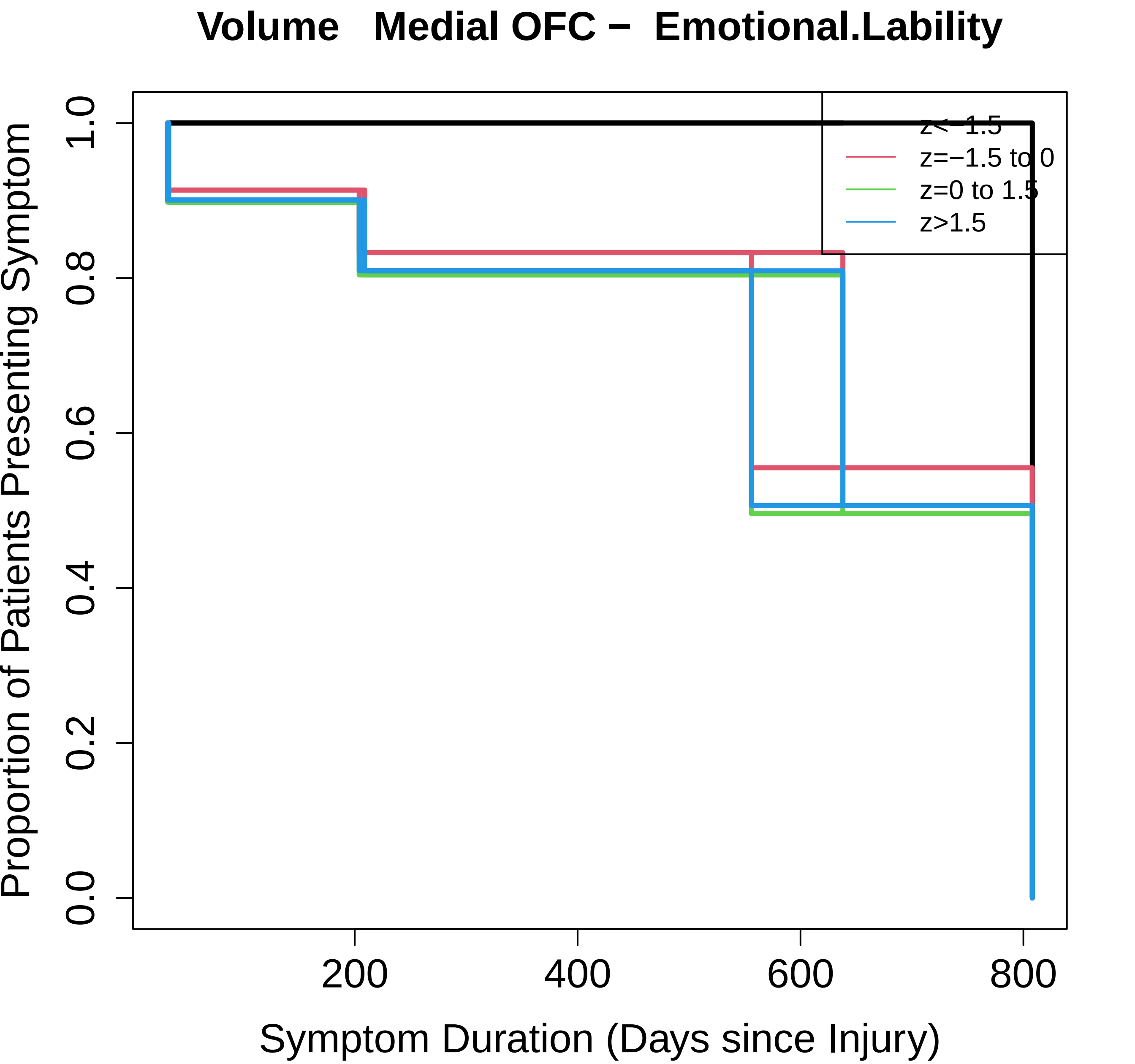

Volume Medial OFC − Emotional.Lability
Proportion of Patients Presenting Symptom
Symptom Duration (Days since Injury)
0.0
0.2
0.4
0.6
0.8
1.0
200
400
600
800
z<−1.5
z=−1.5 to 0
z=0 to 1.5
z>1.5

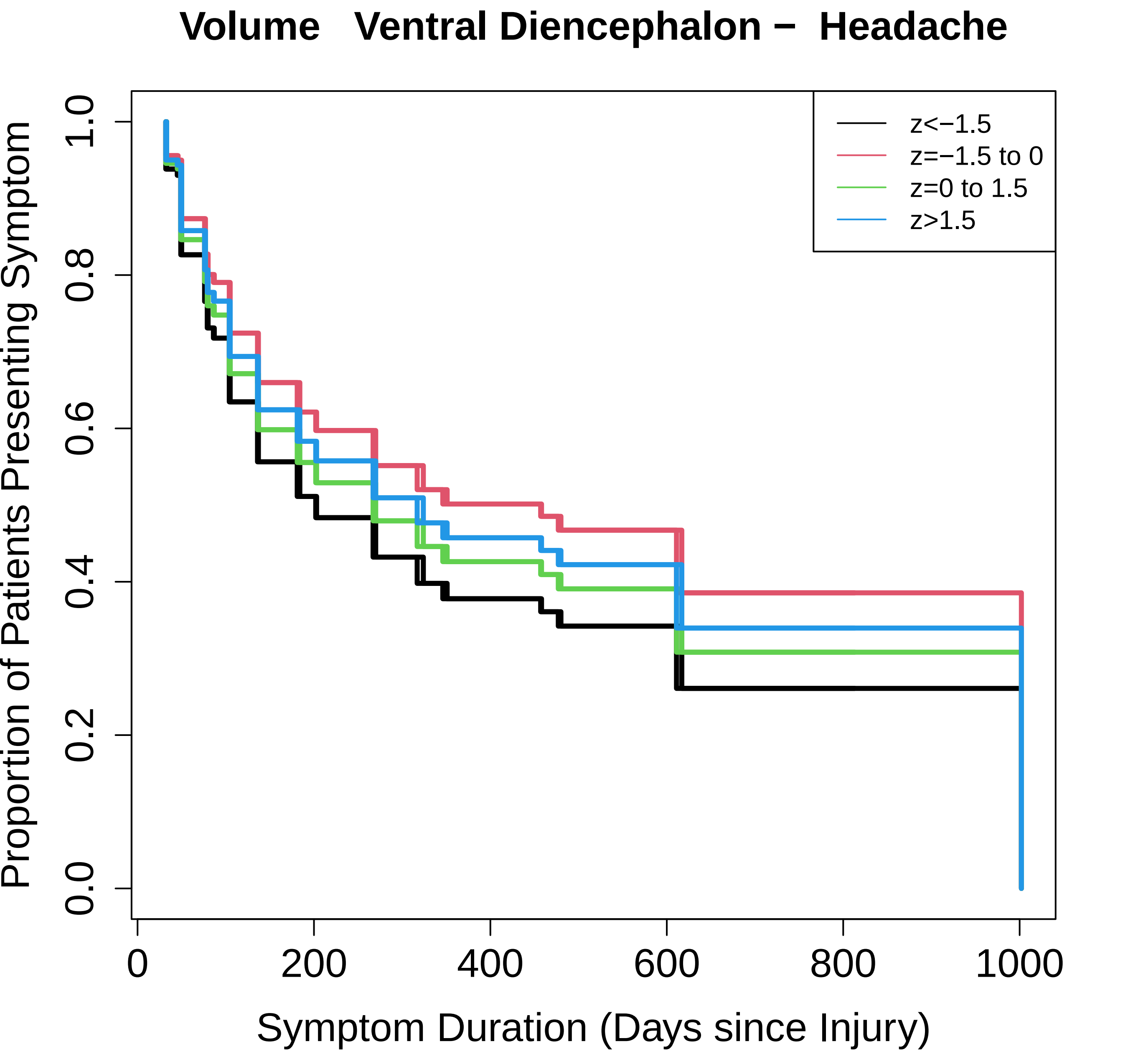

Volume Ventral Diencephalon − Headache
Proportion of Patients Presenting Symptom
0.0
0.2
0.4
0.6
0.8
1.0
0
200
400
600
800
1000
Symptom Duration (Days since Injury)
z<−1.5
z=−1.5 to 0
z=0 to 1.5
z>1.5

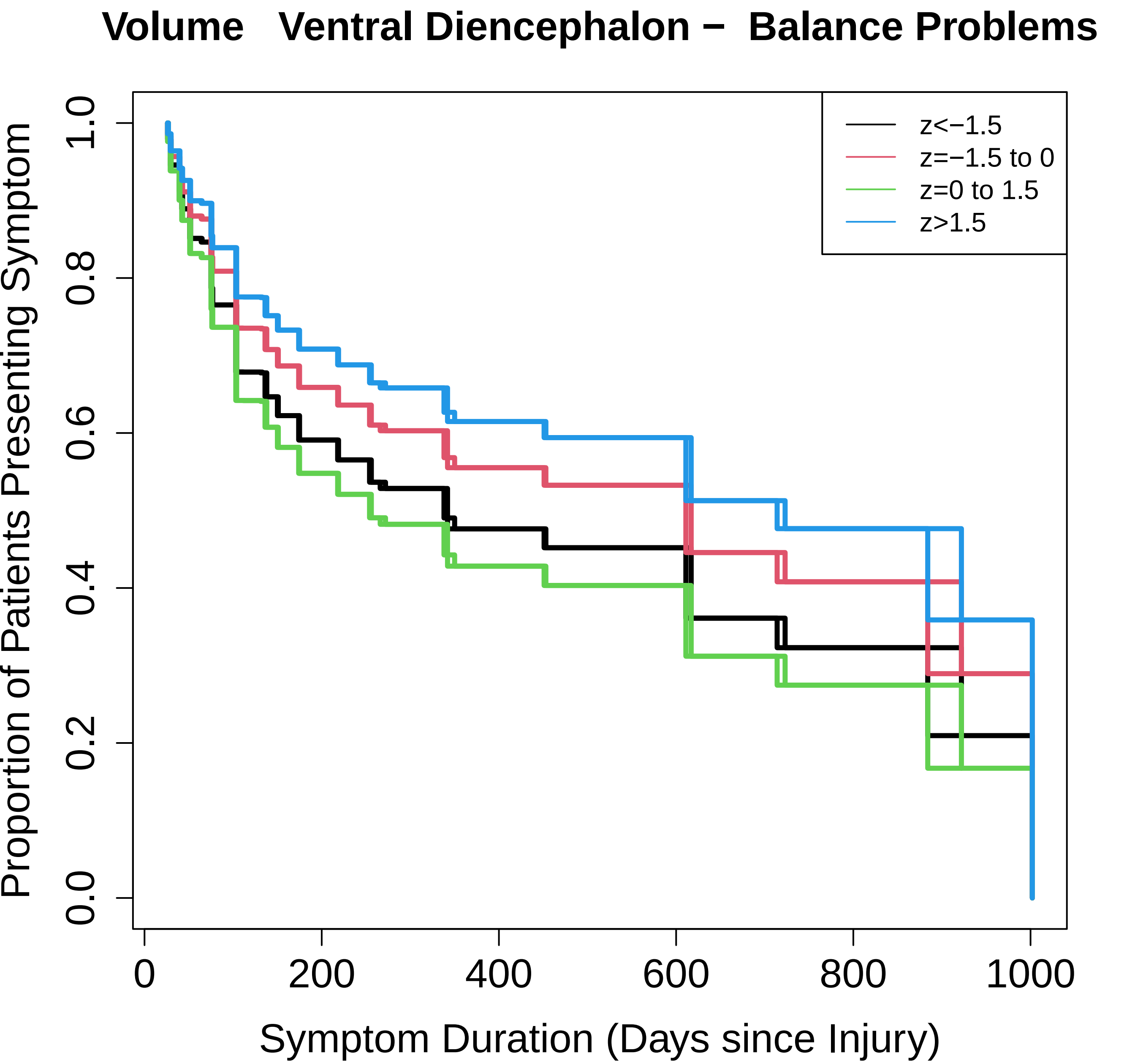

Volume Ventral Diencephalon − Balance Problems
Proportion of Patients Presenting Symptom
Symptom Duration (Days since Injury)
0.0
0.2
0.4
0.6
0.8
1.0
0
200
400
600
800
1000
z<−1.5
z=−1.5 to 0
z=0 to 1.5
z>1.5

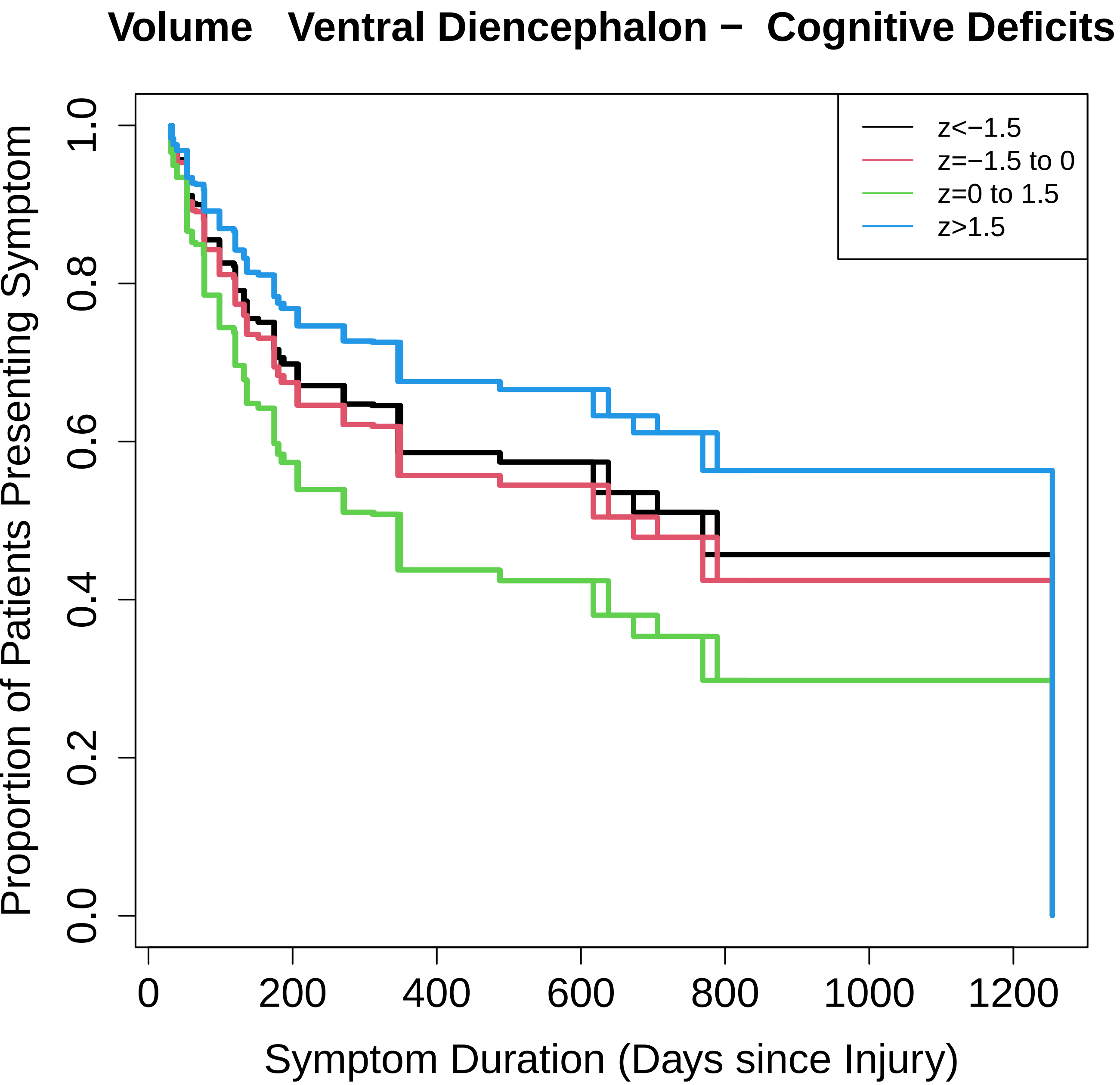

Volume Ventral Diencephalon − Cognitive Deficits
Proportion of Patients Presenting Symptom
Symptom Duration (Days since Injury)
z<−1.5
z=−1.5 to 0
z=0 to 1.5
z>1.5
0.0
0.2
0.4
0.6
0.8
1.0
0
200
400
600
800
1000
1200

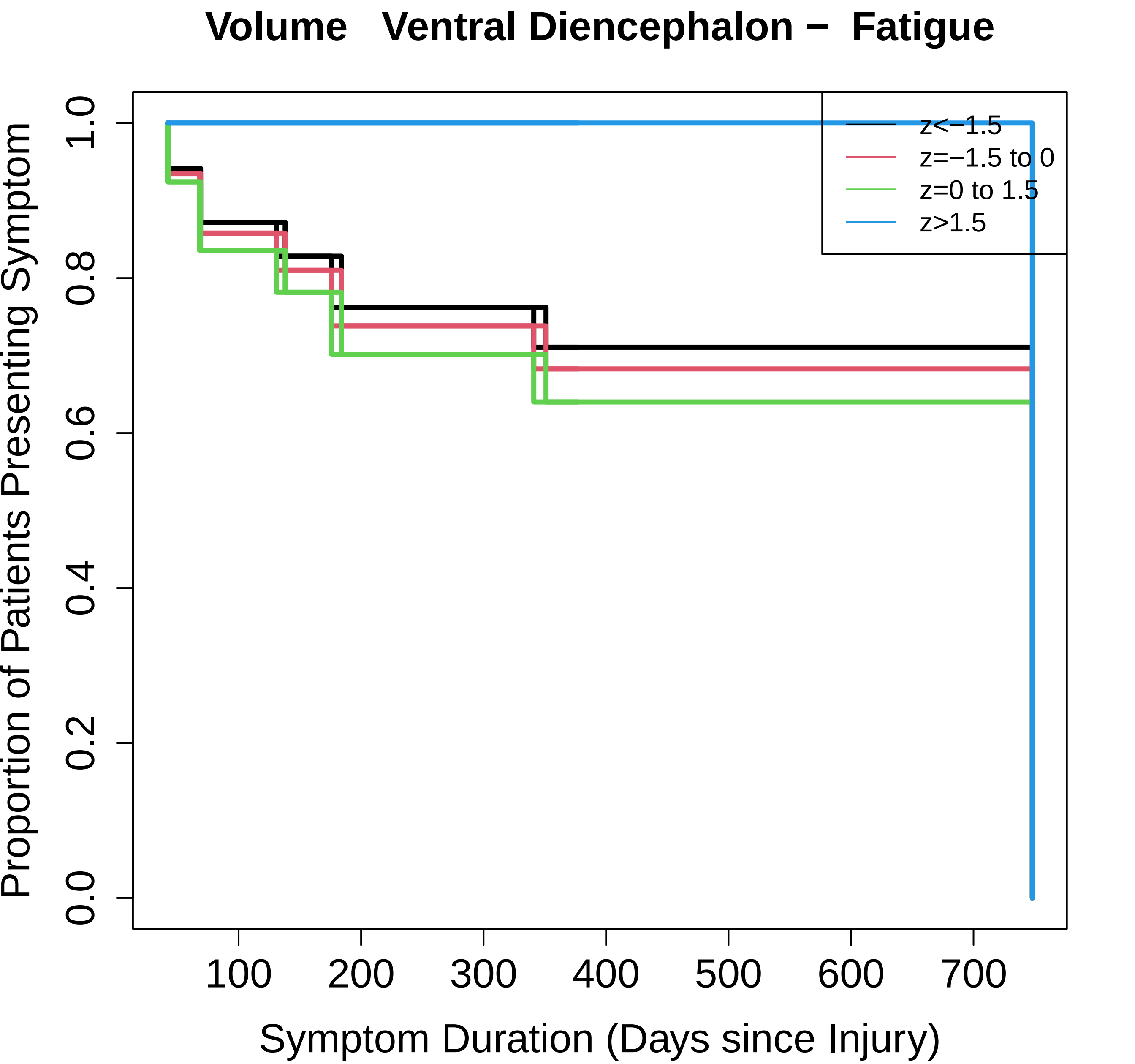
Volume Ventral Diencephalon − Fatigue
Proportion of Patients Presenting Symptom
Symptom Duration (Days since Injury)
0.0
0.2
0.4
0.6
0.8
1.0
100
200
300
400
500
600
700
z<−1.5
z=−1.5 to 0
z=0 to 1.5
z>1.5

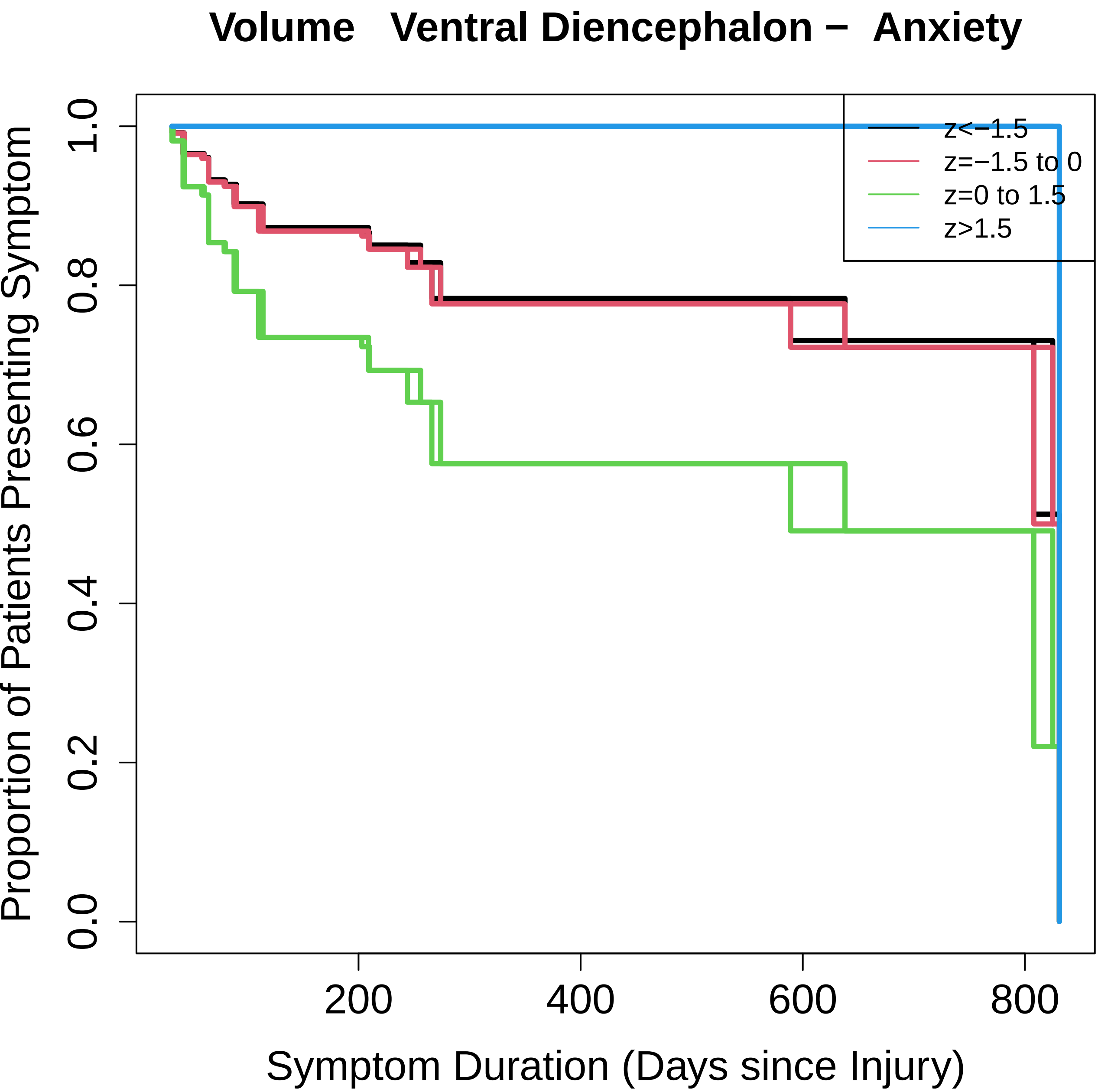
Volume Ventral Diencephalon − Anxiety
Proportion of Patients Presenting Symptom
Symptom Duration (Days since Injury)
0.0
0.2
0.4
0.6
0.8
1.0
200
400
600
800
z<−1.5
z=−1.5 to 0
z=0 to 1.5
z>1.5

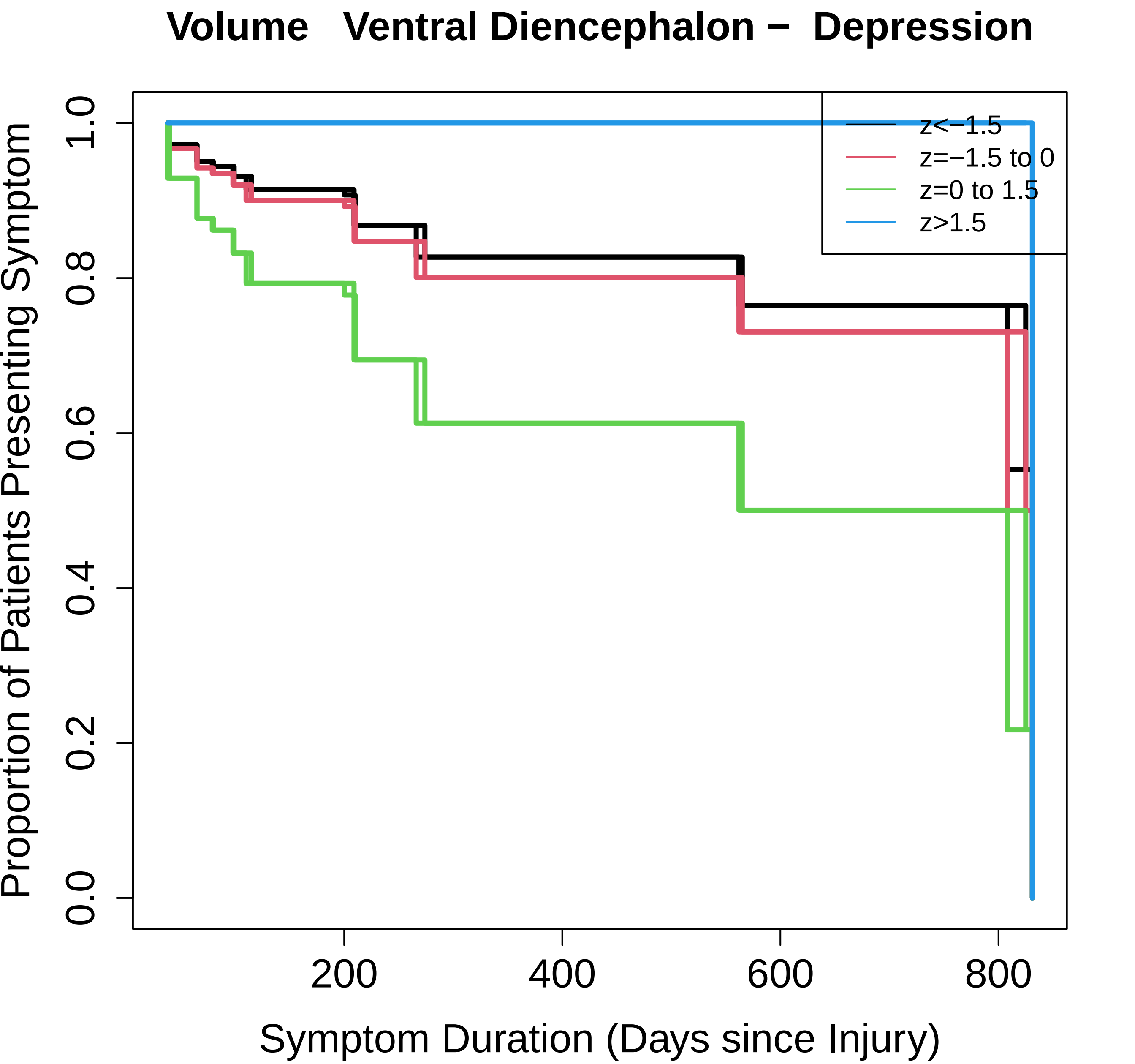

Volume Ventral Diencephalon − Depression
Proportion of Patients Presenting Symptom
Symptom Duration (Days since Injury)
0.0
0.2
0.4
0.6
0.8
1.0
200
400
600
800
z<−1.5
z=−1.5 to 0
z=0 to 1.5
z>1.5

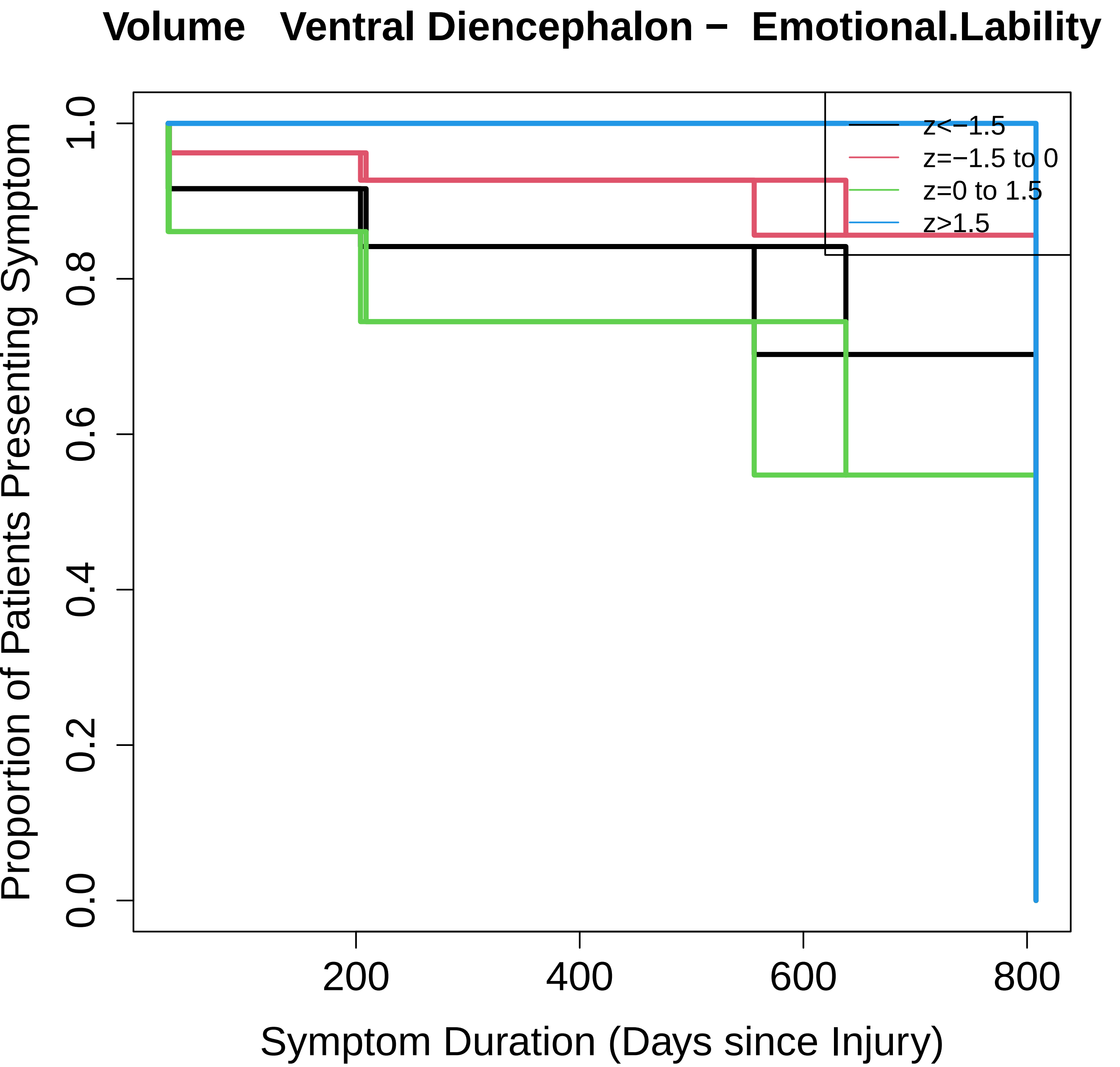

Volume Ventral Diencephalon − Emotional.Lability
z<−1.5
z=−1.5 to 0
z=0 to 1.5
z>1.5
Proportion of Patients Presenting Symptom
0.0
0.2
0.4
0.6
0.8
1.0
200
400
600
800
Symptom Duration (Days since Injury)

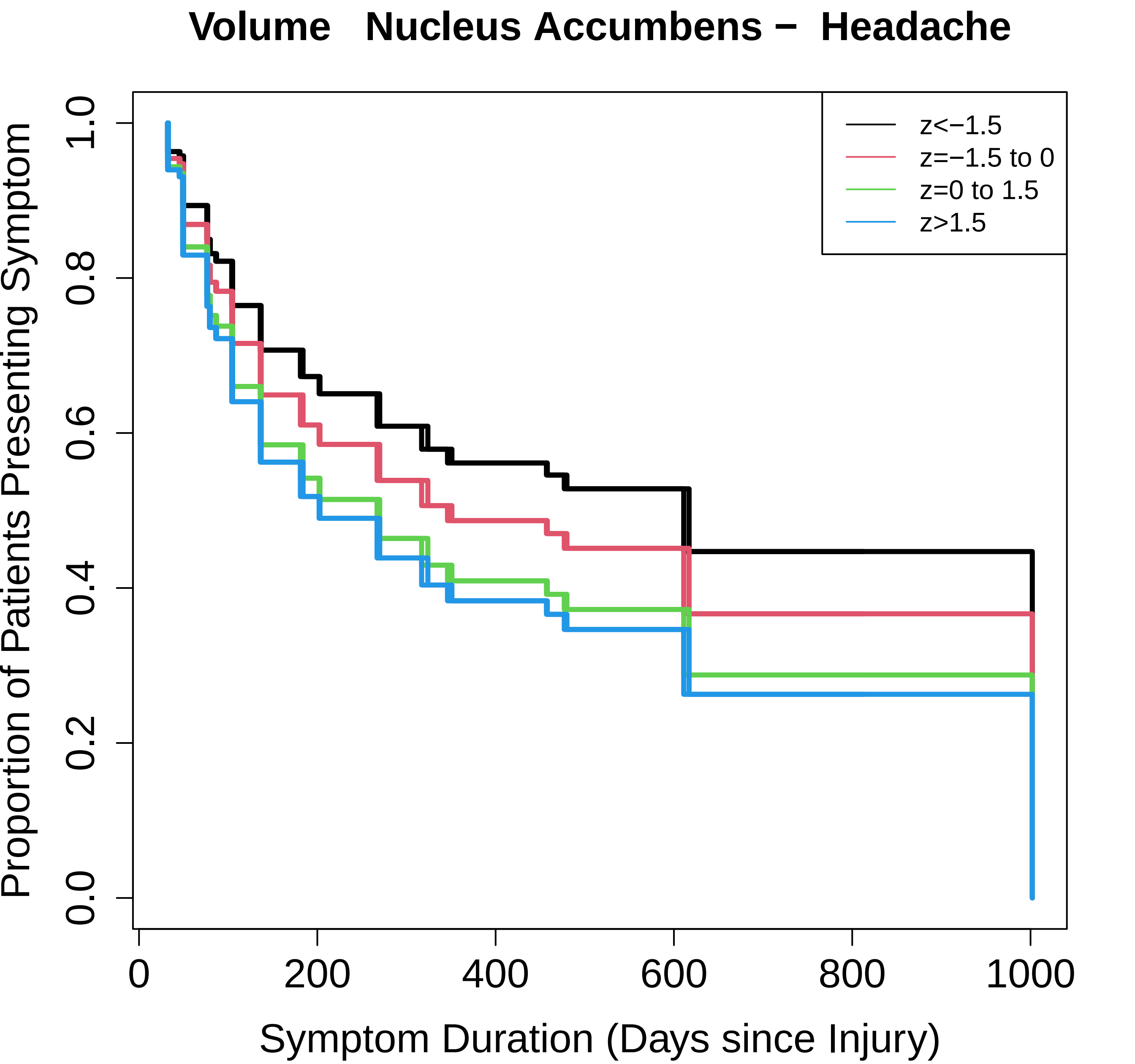
Volume Nucleus Accumbens − Headache
Proportion of Patients Presenting Symptom
Symptom Duration (Days since Injury)
0.0
0.2
0.4
0.6
0.8
1.0
0
200
400
600
800
1000
z<−1.5
z=−1.5 to 0
z=0 to 1.5
z>1.5

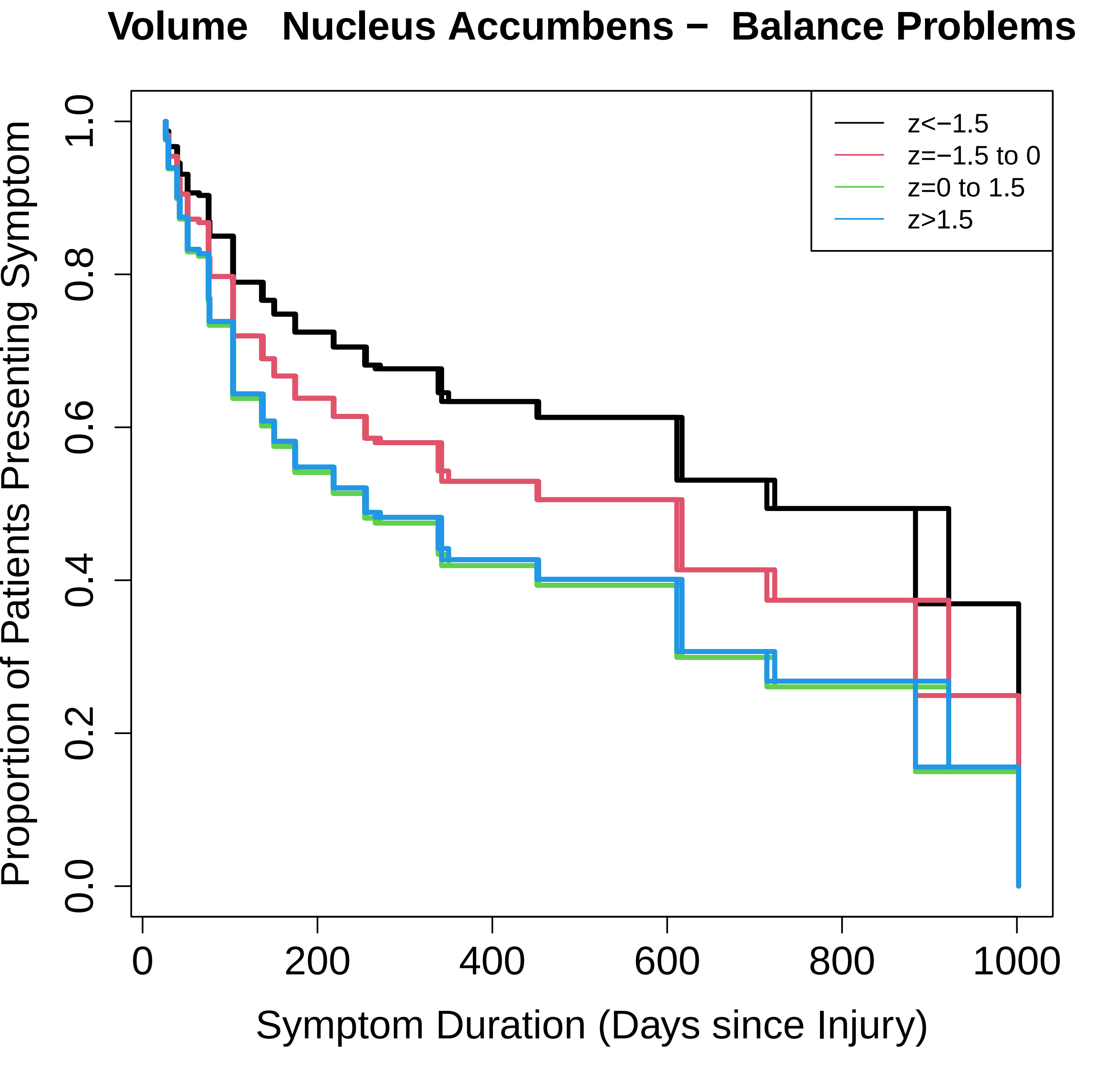

Volume Nucleus Accumbens − Balance Problems
z<−1.5
z=−1.5 to 0
z=0 to 1.5
z>1.5
Proportion of Patients Presenting Symptom
0.0
0.2
0.4
0.6
0.8
1.0
0
200
400
600
800
1000
Symptom Duration (Days since Injury)

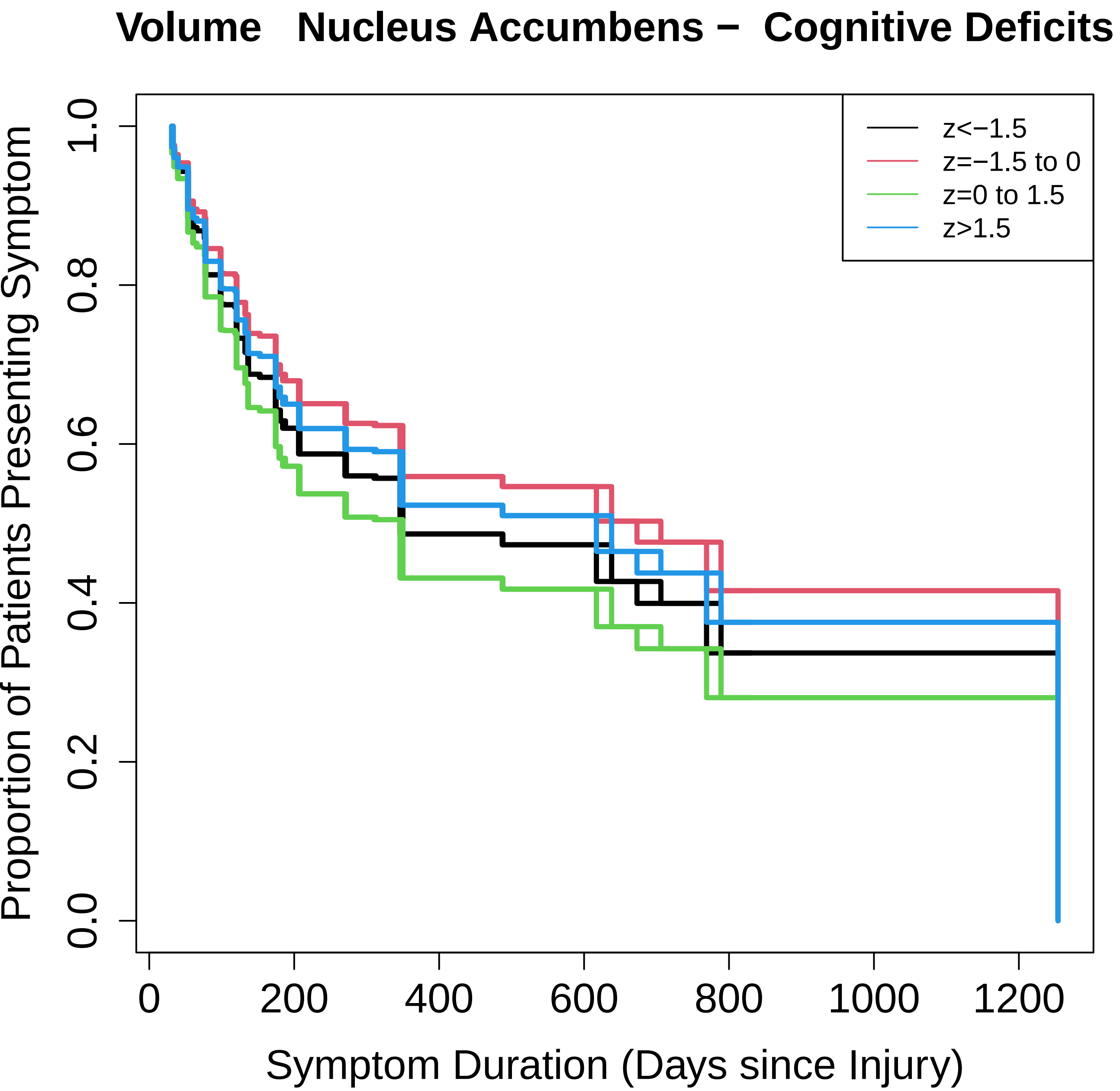
Volume Nucleus Accumbens − Cognitive Deficits
z<−1.5
z=−1.5 to 0
z=0 to 1.5
z>1.5
Proportion of Patients Presenting Symptom
0.0
0.2
0.4
0.6
0.8
1.0
0
200
400
600
800
1000
1200
Symptom Duration (Days since Injury)

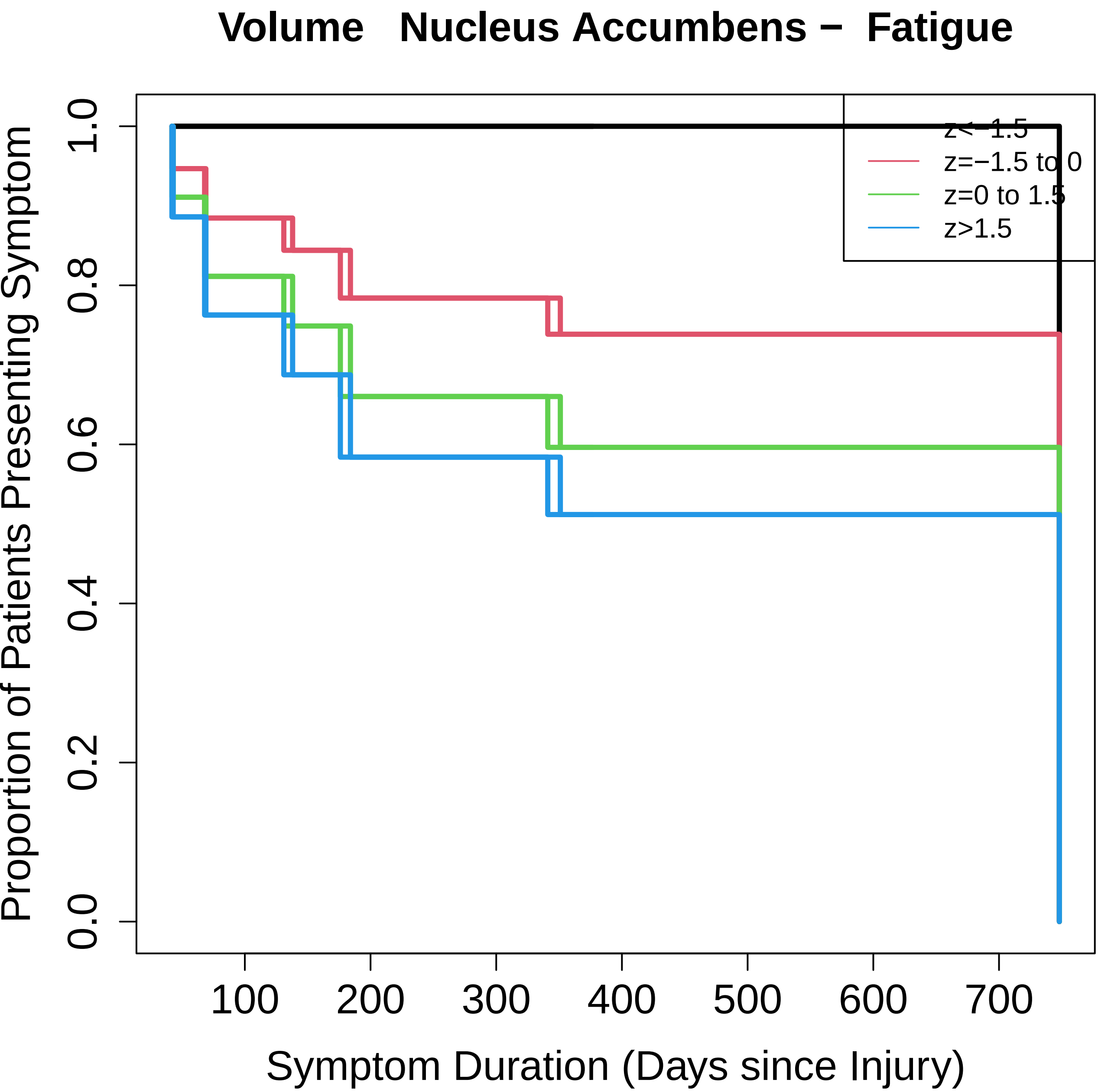

Volume Nucleus Accumbens − Fatigue
z<−1.5
z=−1.5 to 0
z=0 to 1.5
z>1.5
Proportion of Patients Presenting Symptom
0.0
0.2
0.4
0.6
0.8
1.0
100
200
300
400
500
600
700
Symptom Duration (Days since Injury)

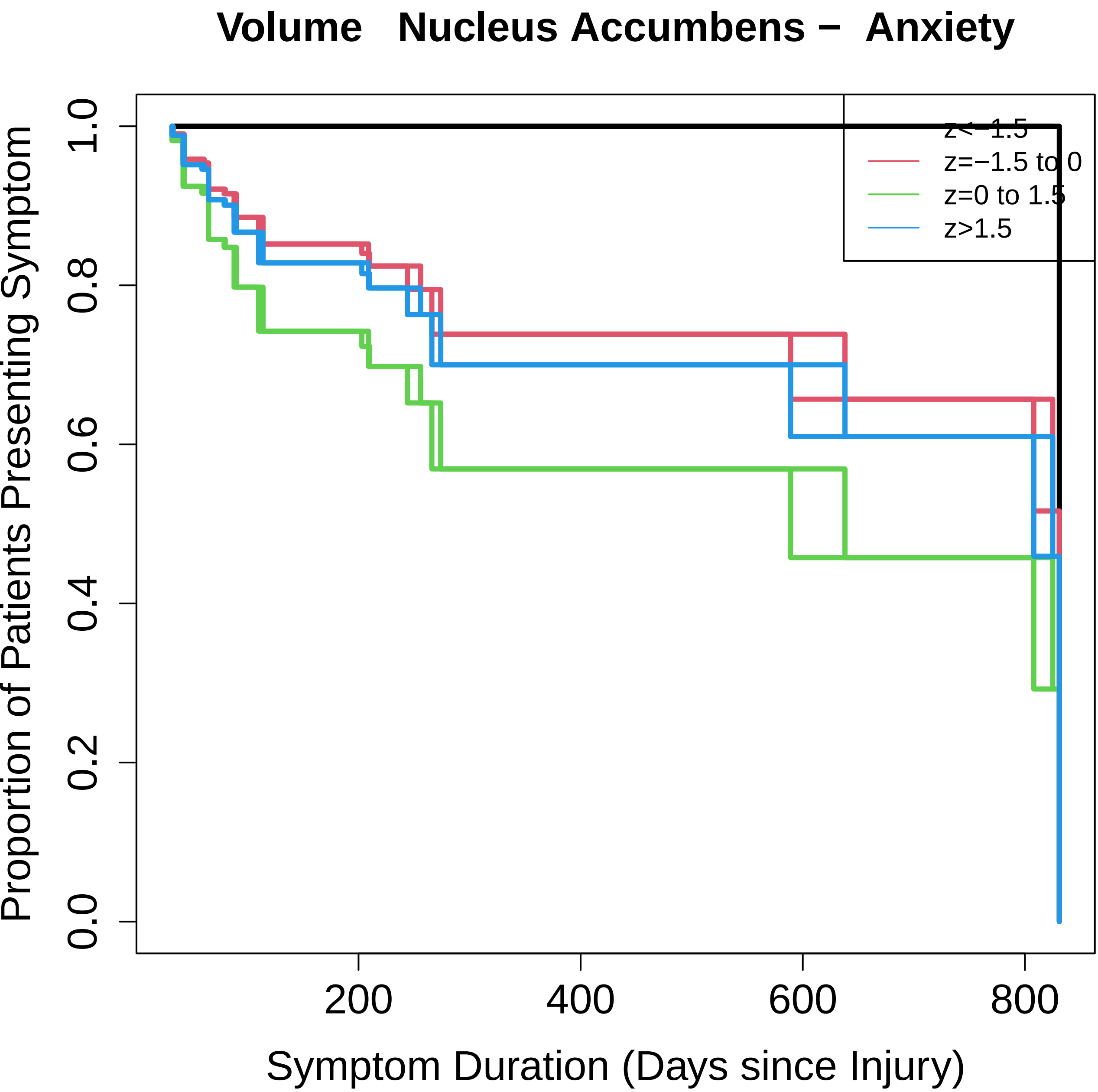
Volume Nucleus Accumbens − Anxiety
Proportion of Patients Presenting Symptom
Symptom Duration (Days since Injury)
0.0
0.2
0.4
0.6
0.8
1.0
200
400
600
800
z<−1.5
z=−1.5 to 0
z=0 to 1.5
z>1.5

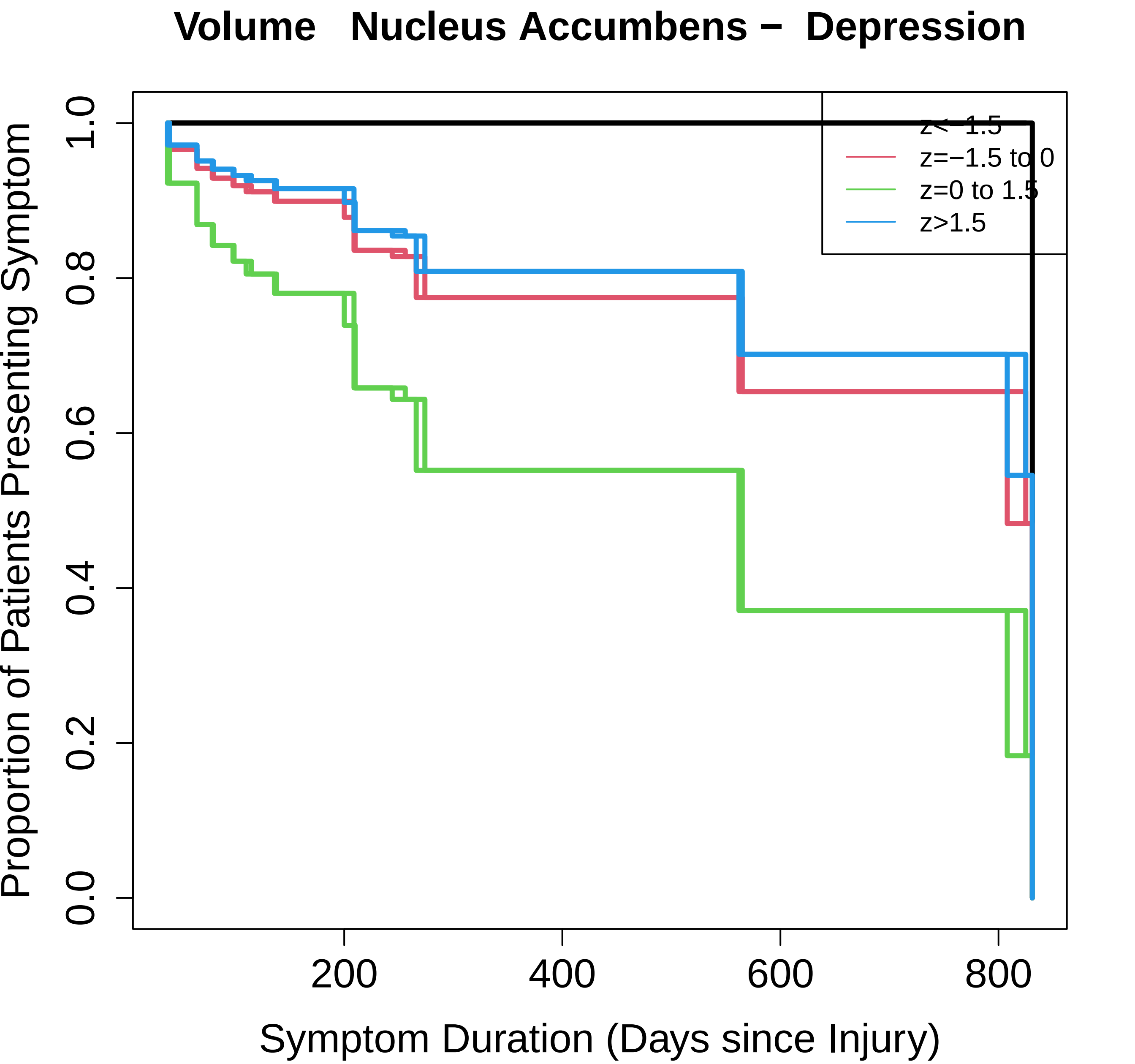
Volume Nucleus Accumbens − Depression
z<−1.5
z=−1.5 to 0
z=0 to 1.5
z>1.5
Proportion of Patients Presenting Symptom
0.0
0.2
0.4
0.6
0.8
1.0
200
400
600
800
Symptom Duration (Days since Injury)

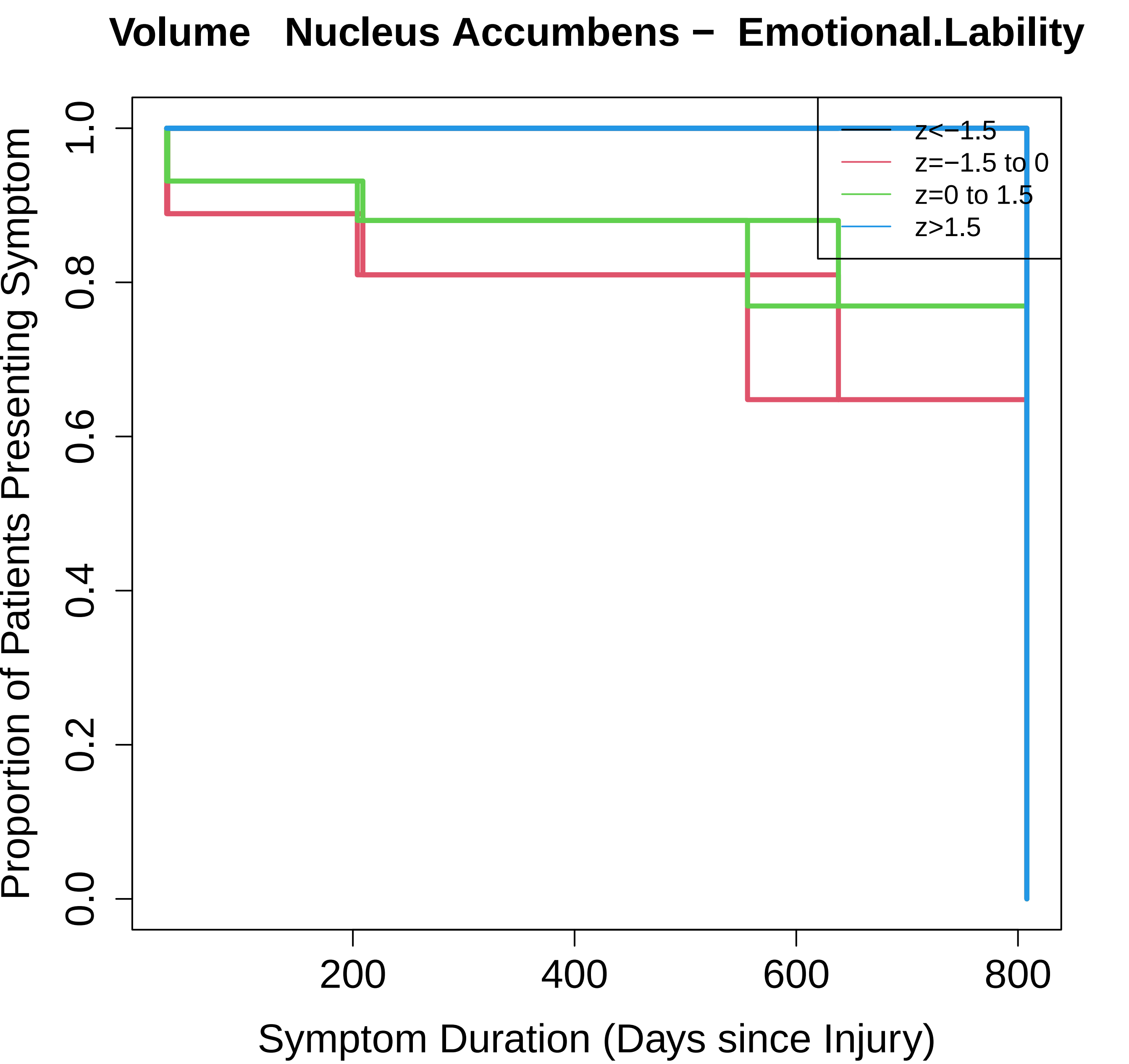
Volume Nucleus Accumbens − Emotional.Lability
Proportion of Patients Presenting Symptom
0.0
0.2
0.4
0.6
0.8
1.0
200
400
600
800
Symptom Duration (Days since Injury)
z<−1.5
z=−1.5 to 0
z=0 to 1.5
z>1.5

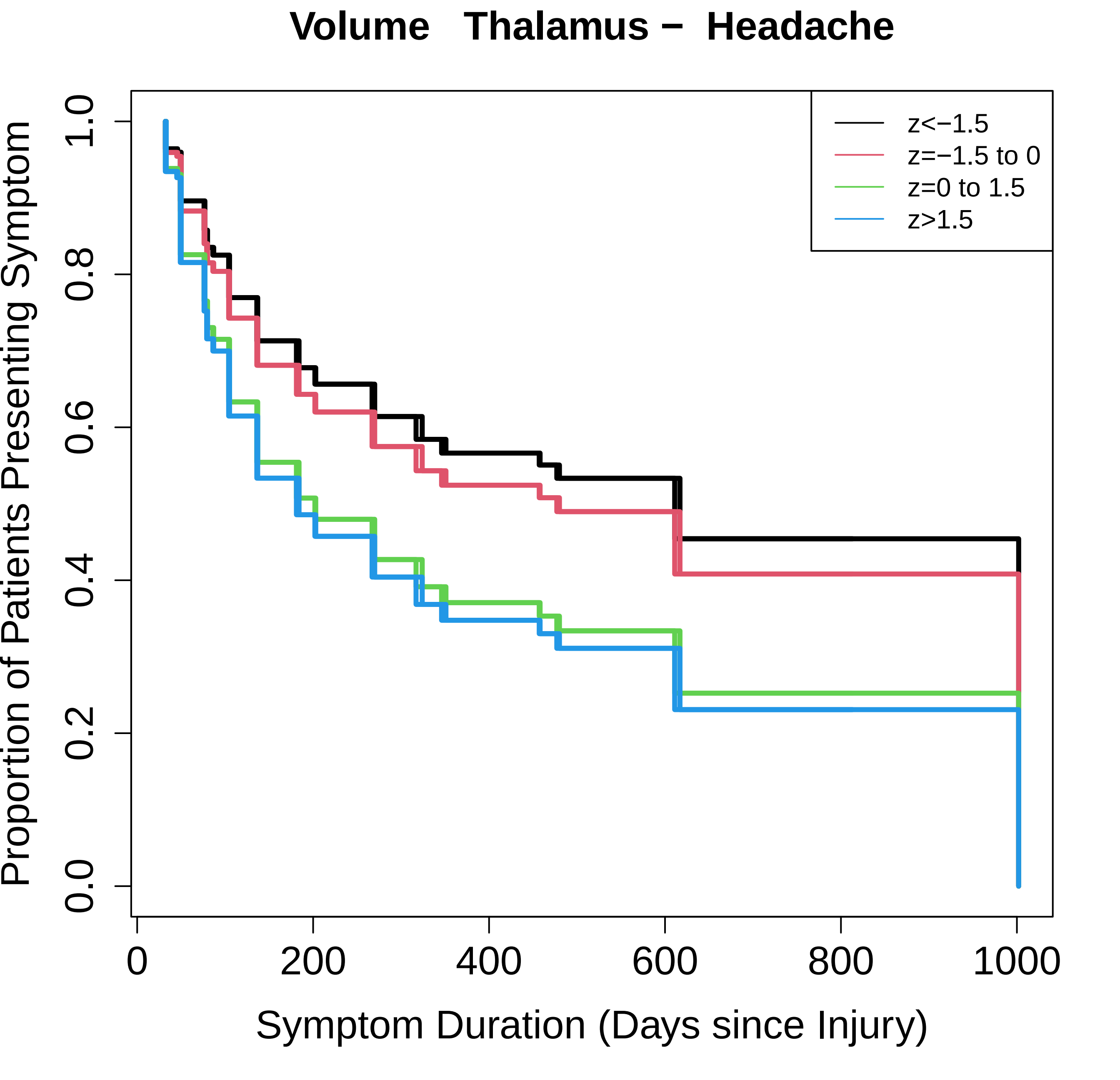

Volume Thalamus − Headache
Proportion of Patients Presenting Symptom
Symptom Duration (Days since Injury)
0.0
0.2
0.4
0.6
0.8
1.0
0
200
400
600
800
1000
z<−1.5
z=−1.5 to 0
z=0 to 1.5
z>1.5

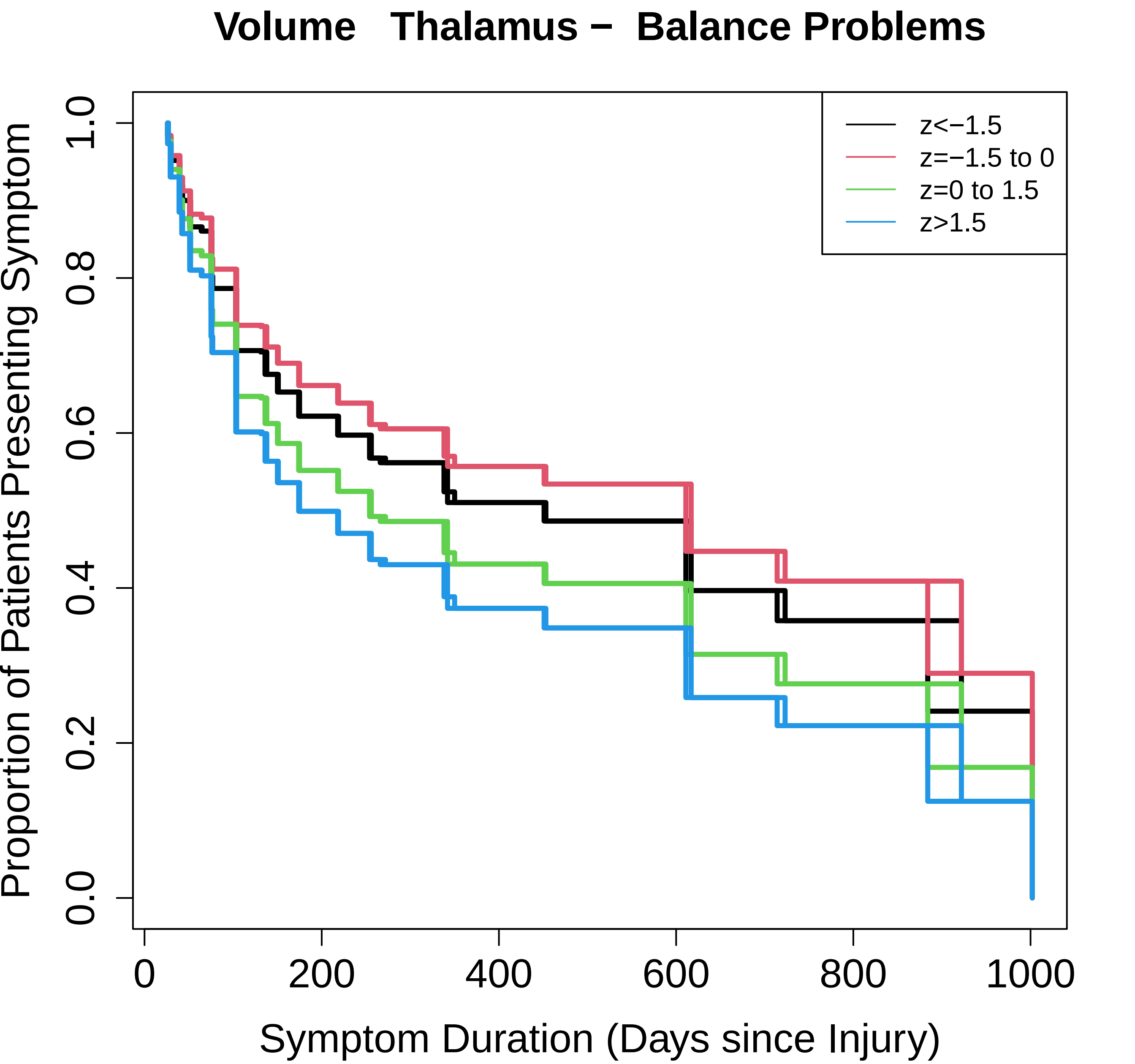

Volume Thalamus − Balance Problems
Proportion of Patients Presenting Symptom
Symptom Duration (Days since Injury)
0.0
0.2
0.4
0.6
0.8
1.0
0
200
400
600
800
1000
z<−1.5
z=−1.5 to 0
z=0 to 1.5
z>1.5

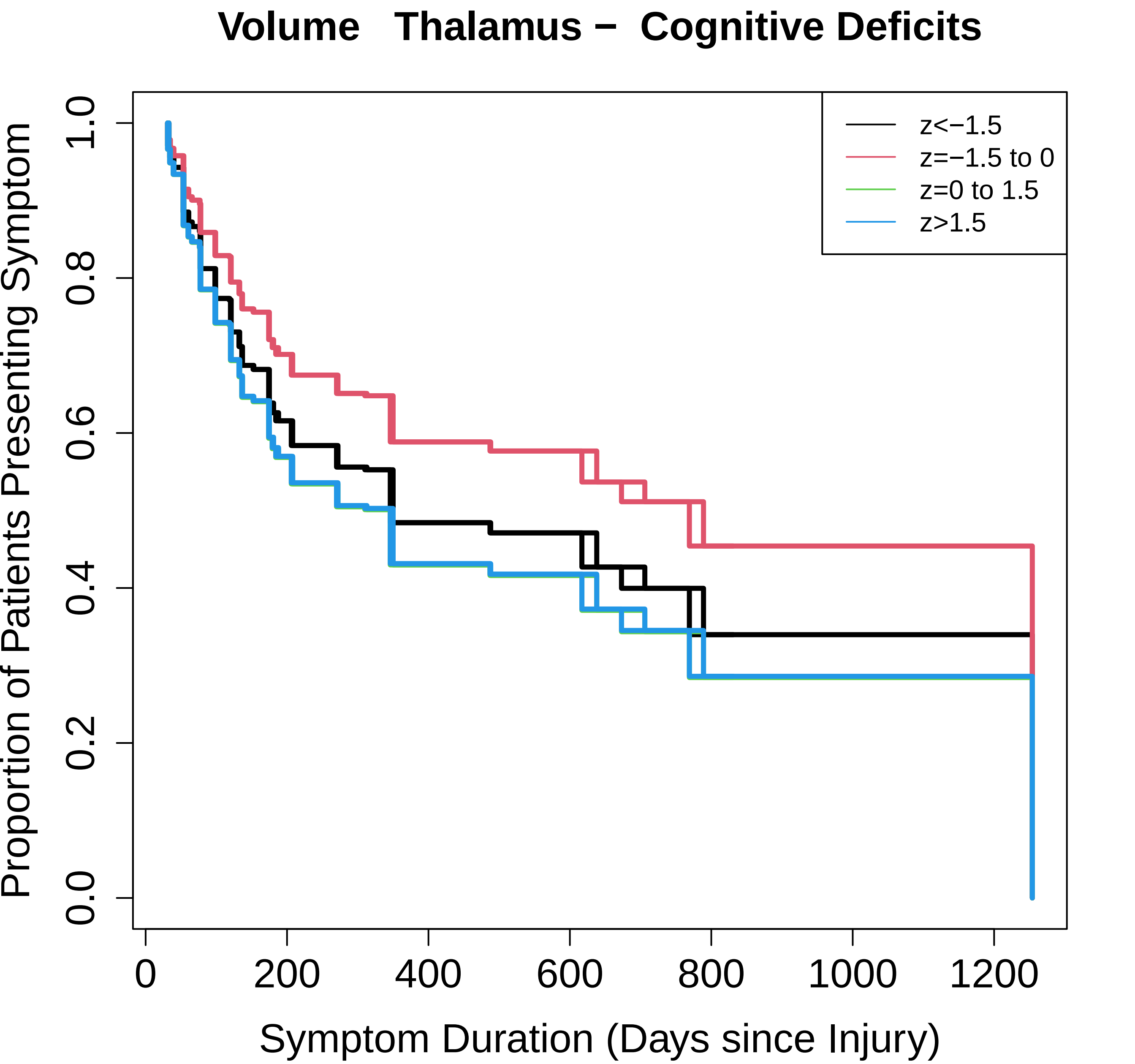

Volume Thalamus − Cognitive Deficits
Proportion of Patients Presenting Symptom
Symptom Duration (Days since Injury)
0.0
0.2
0.4
0.6
0.8
1.0
0
200
400
600
800
1000
1200
z<−1.5
z=−1.5 to 0
z=0 to 1.5
z>1.5

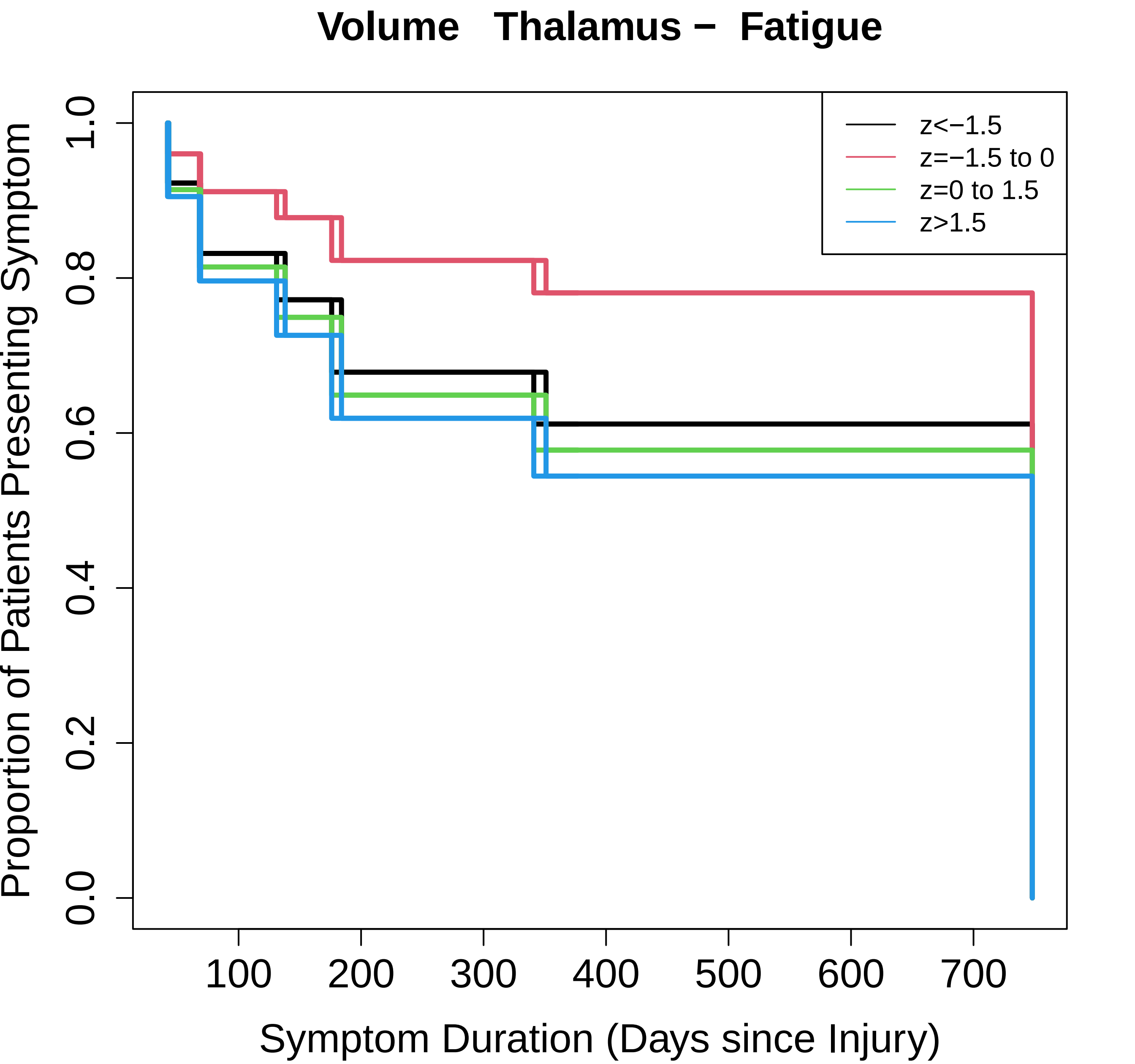

Volume Thalamus − Fatigue
Proportion of Patients Presenting Symptom
Symptom Duration (Days since Injury)
0.0
0.2
0.4
0.6
0.8
1.0
100
200
300
400
500
600
700
z<−1.5
z=−1.5 to 0
z=0 to 1.5
z>1.5

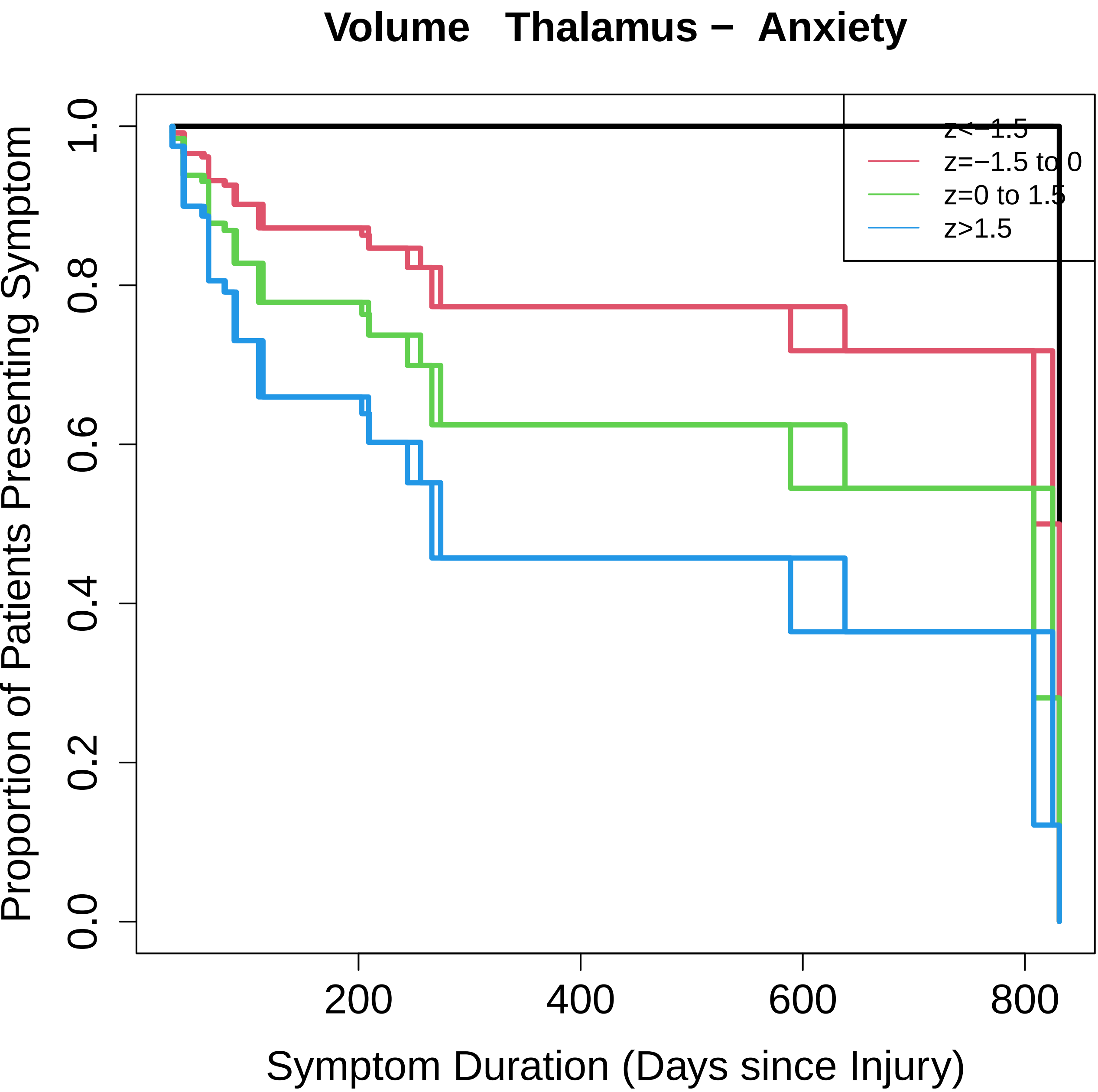
Volume Thalamus − Anxiety
Proportion of Patients Presenting Symptom
0.0
0.2
0.4
0.6
0.8
1.0
200
400
600
800
Symptom Duration (Days since Injury)
z<−1.5
z=−1.5 to 0
z=0 to 1.5
z>1.5

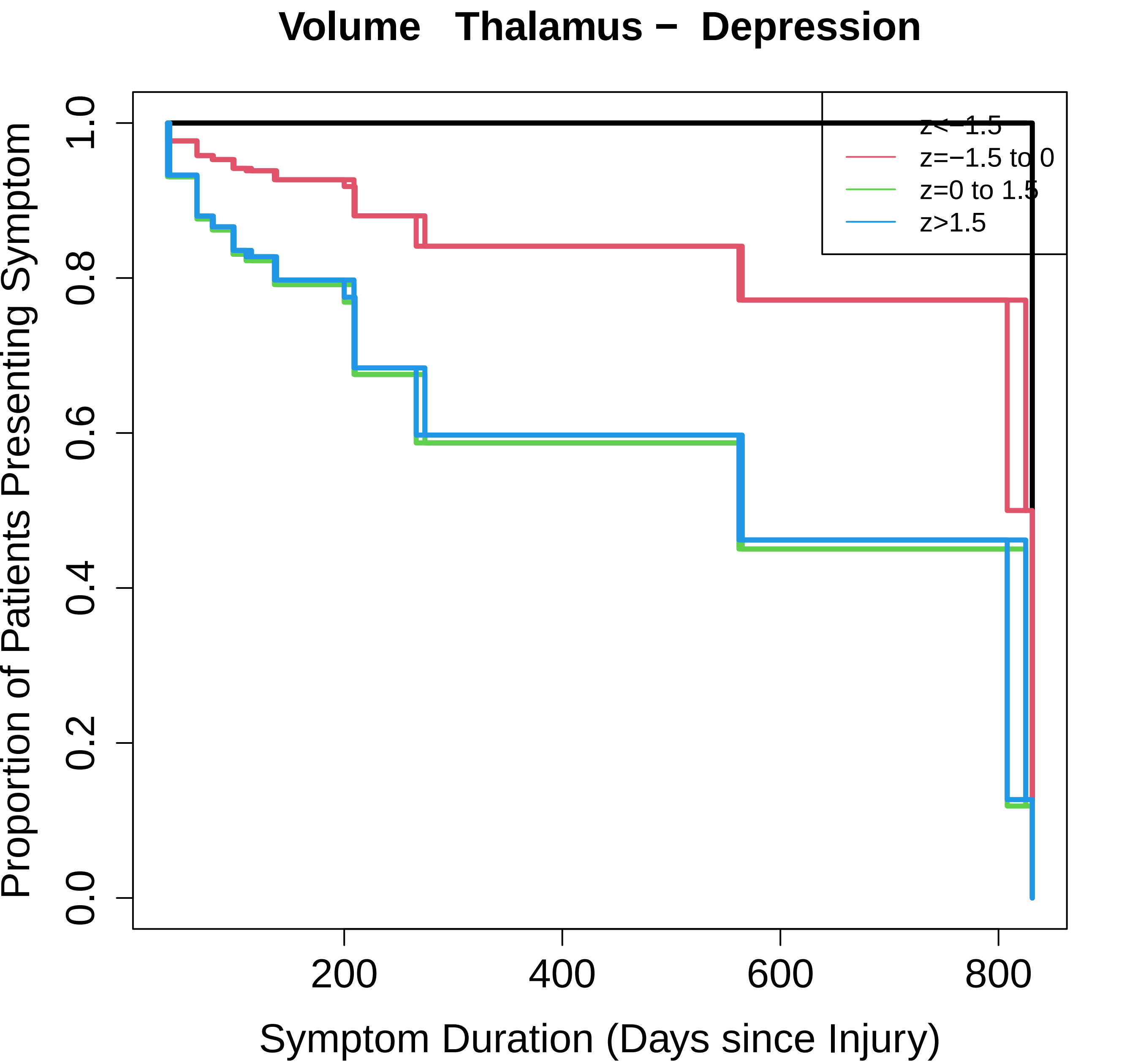

Volume Thalamus − Depression
Proportion of Patients Presenting Symptom
Symptom Duration (Days since Injury)
0.0
0.2
0.4
0.6
0.8
1.0
200
400
600
800
z<−1.5
z=−1.5 to 0
z=0 to 1.5
z>1.5

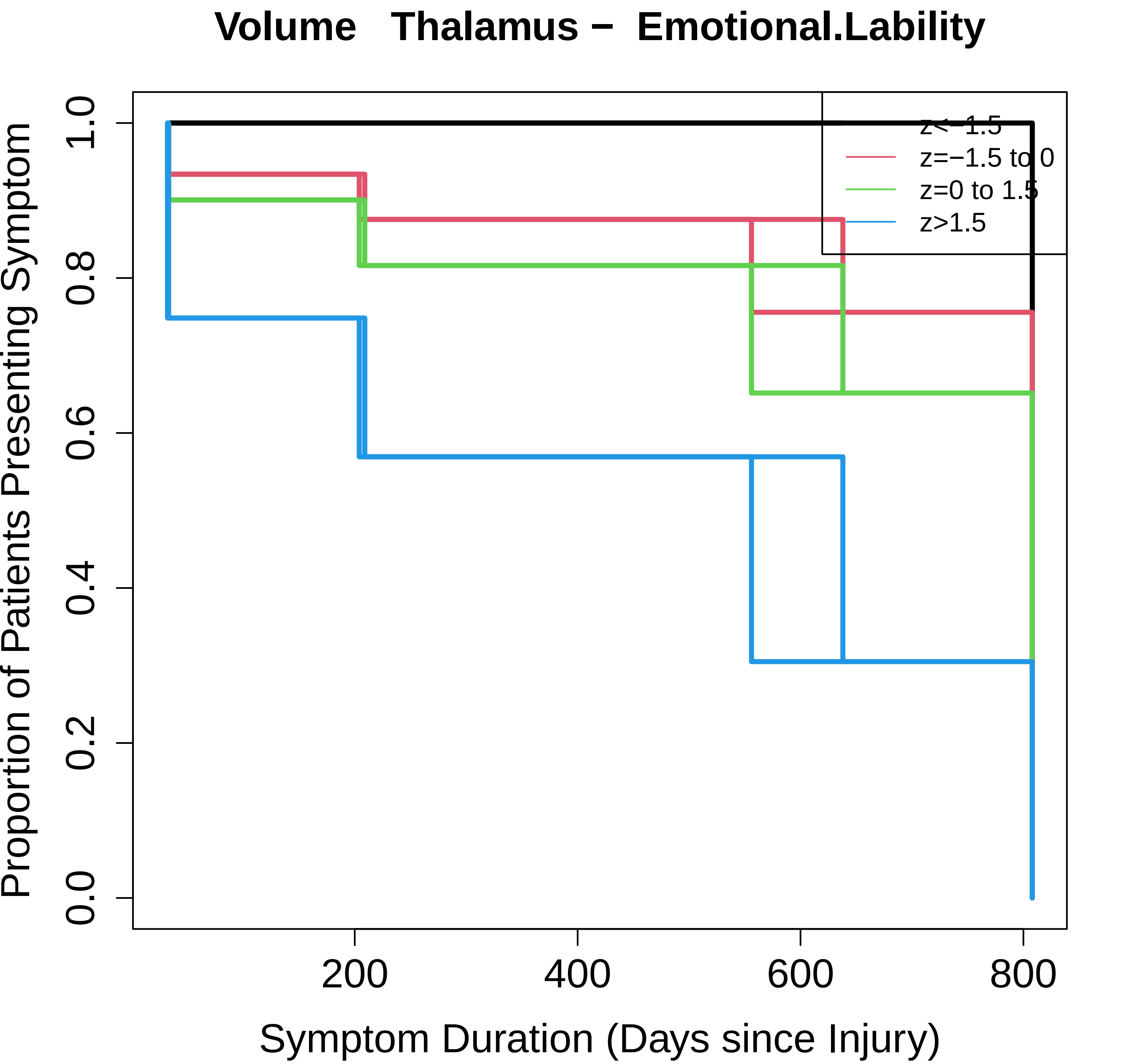
Volume Thalamus − Emotional.Lability
Proportion of Patients Presenting Symptom
Symptom Duration (Days since Injury)
0.0
0.2
0.4
0.6
0.8
1.0
200
400
600
800
z<−1.5
z=−1.5 to 0
z=0 to 1.5
z>1.5
