# Supplementary material for: Symptom Persistence Relates to Volume and Asymmetry of the Limbic System after Mild Traumatic Brain Injury
Source: J Clin Med. 2024 Aug 30;13(17):5154. doi: 10.3390/jcm13175154 (PMC11396354; doi:10.3390/jcm13175154)
Supplement: Supplementary file 1 [file jcm-13-05154-s001.zip › Figure S2 Limbic LI clinical symptoms graphs.pdf]

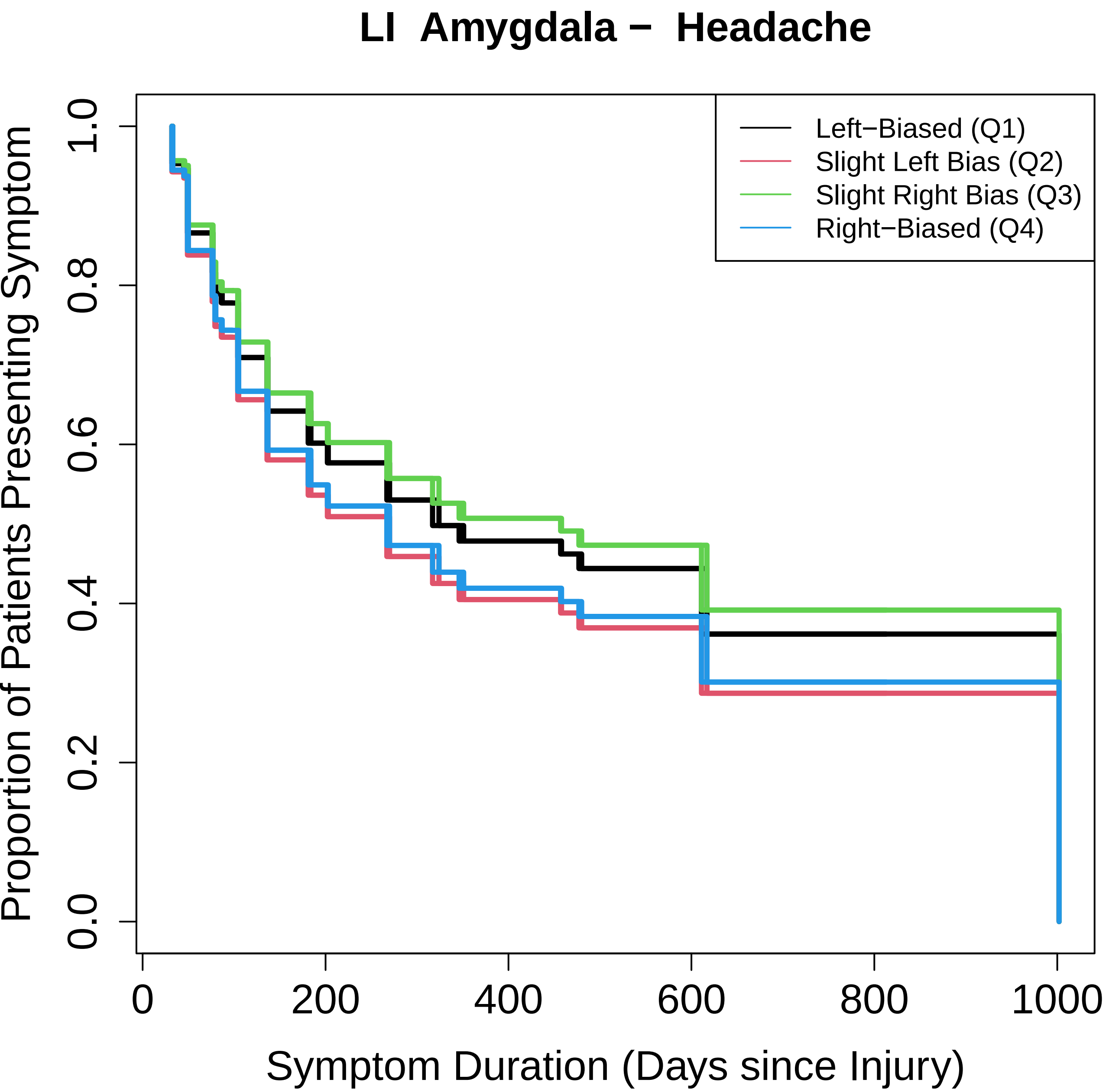

LI Amygdala − Headache
Left−Biased (Q1)
Slight Left Bias (Q2)
Slight Right Bias (Q3)
Right−Biased (Q4)
Proportion of Patients Presenting Symptom
0.0
0.2
0.4
0.6
0.8
1.0
0
200
400
600
800
1000
Symptom Duration (Days since Injury)

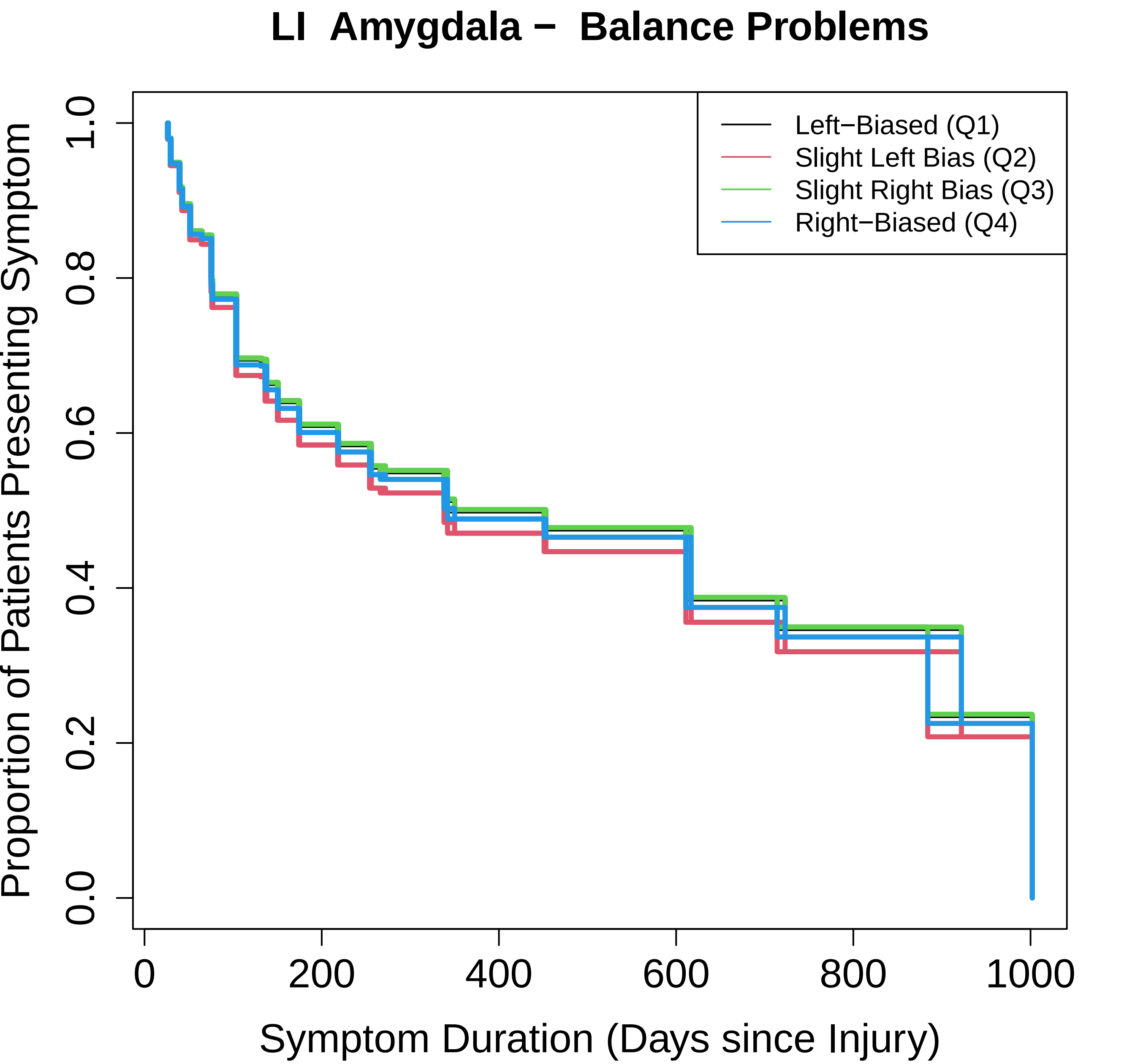

LI Amygdala − Balance Problems
Left−Biased (Q1)
Slight Left Bias (Q2)
Slight Right Bias (Q3)
Right−Biased (Q4)
Proportion of Patients Presenting Symptom
0.0
0.2
0.4
0.6
0.8
1.0
0
200
400
600
800
1000
Symptom Duration (Days since Injury)

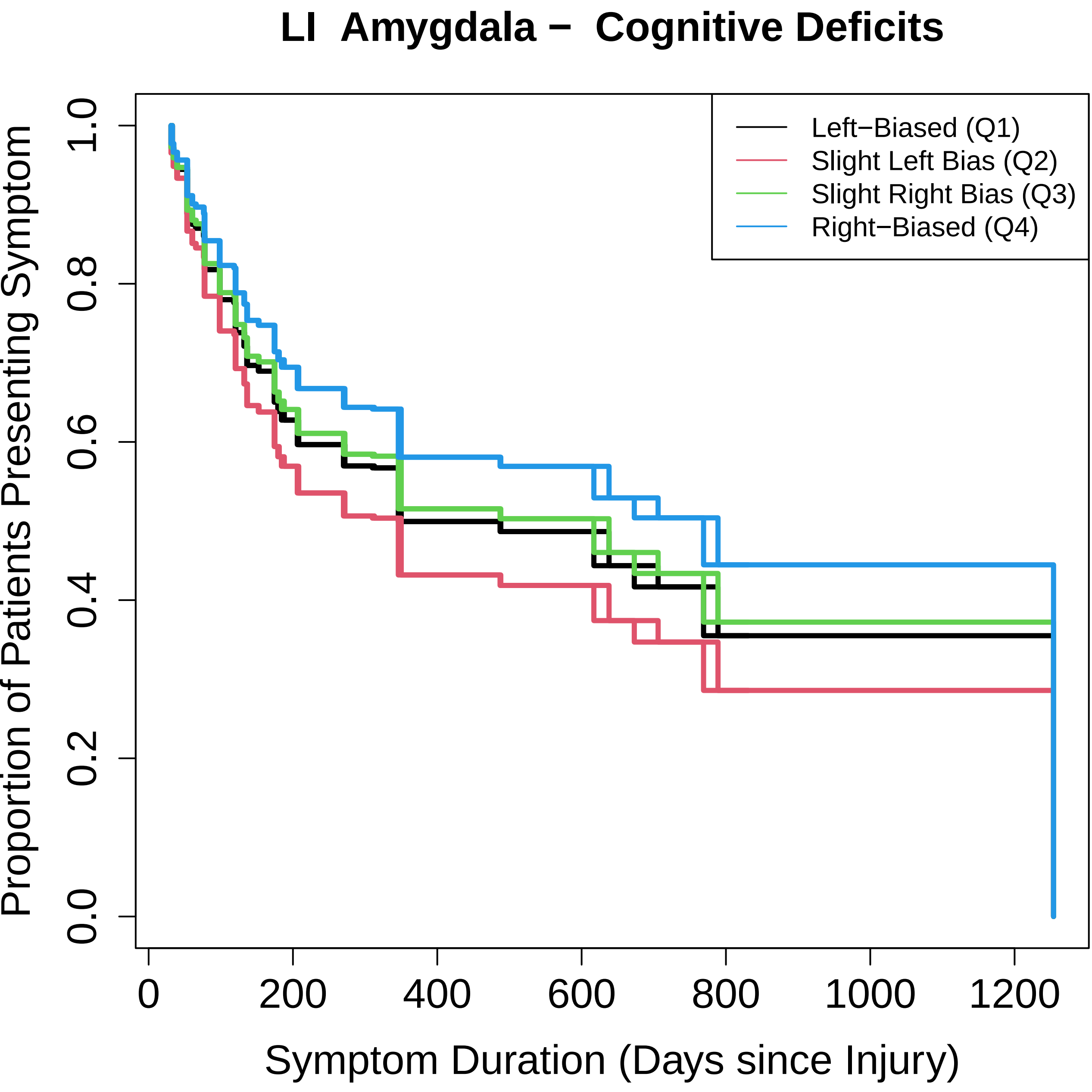

LI Amygdala − Cognitive Deficits
Left−Biased (Q1)
Slight Left Bias (Q2)
Slight Right Bias (Q3)
Right−Biased (Q4)
Proportion of Patients Presenting Symptom
0.0
0.2
0.4
0.6
0.8
1.0
0
200
400
600
800
1000
1200
Symptom Duration (Days since Injury)

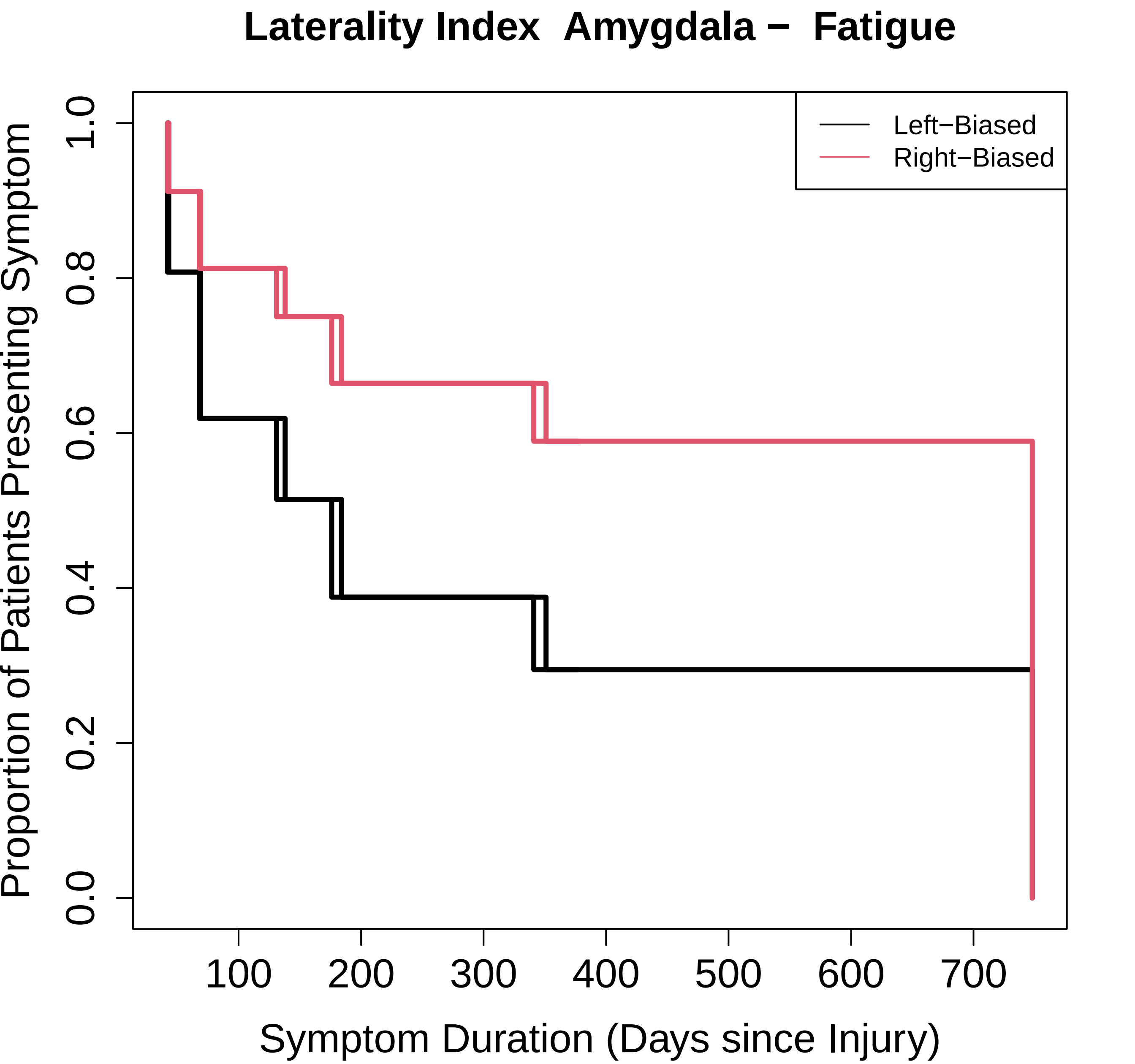
Laterality Index Amygdala − Fatigue
Left−Biased
Right−Biased
Proportion of Patients Presenting Symptom
0.0
0.2
0.4
0.6
0.8
1.0
100
200
300
400
500
600
700
Symptom Duration (Days since Injury)

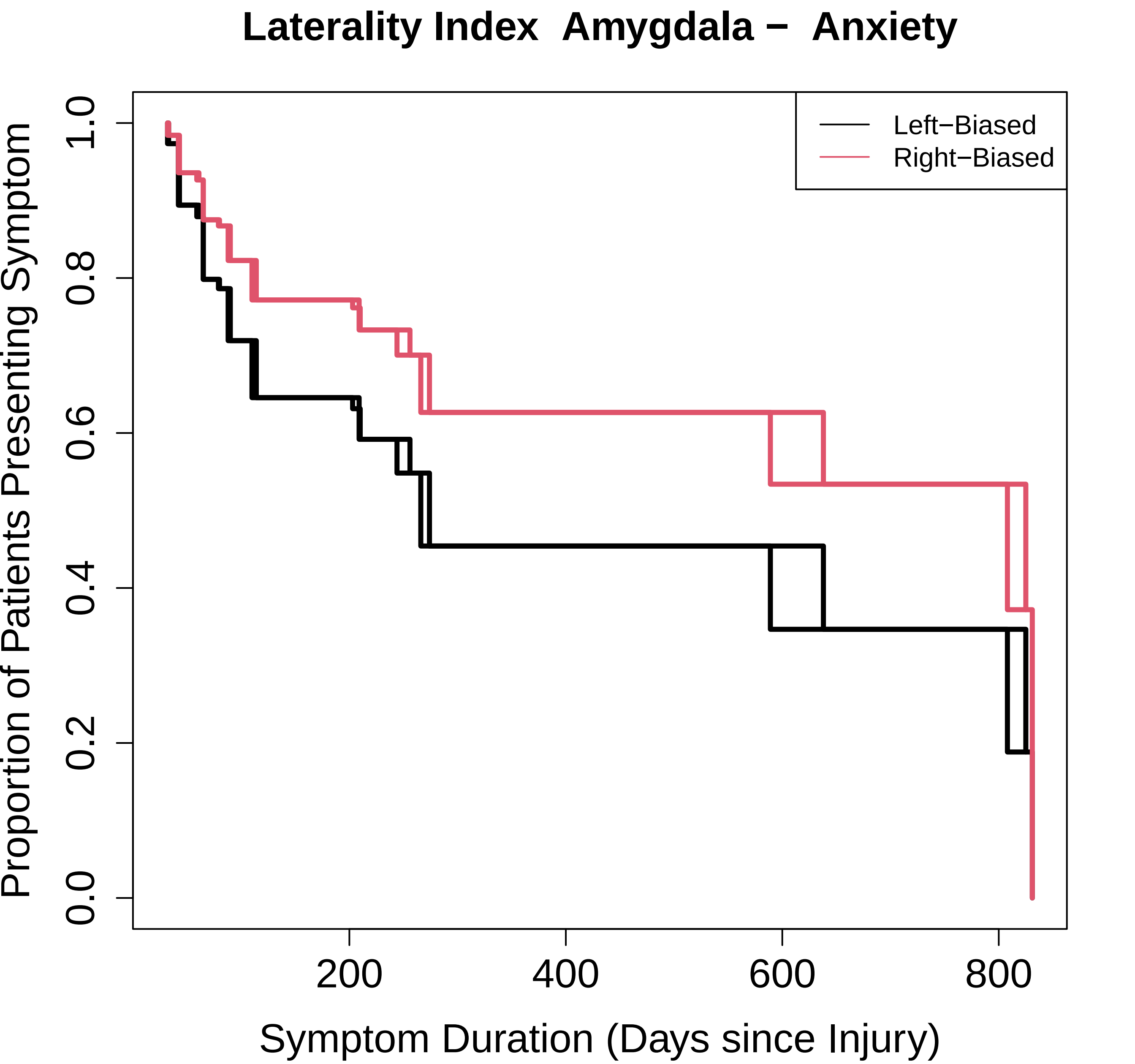

Laterality Index Amygdala − Anxiety
Left−Biased
Right−Biased
Proportion of Patients Presenting Symptom
0.0
0.2
0.4
0.6
0.8
1.0
200
400
600
800
Symptom Duration (Days since Injury)

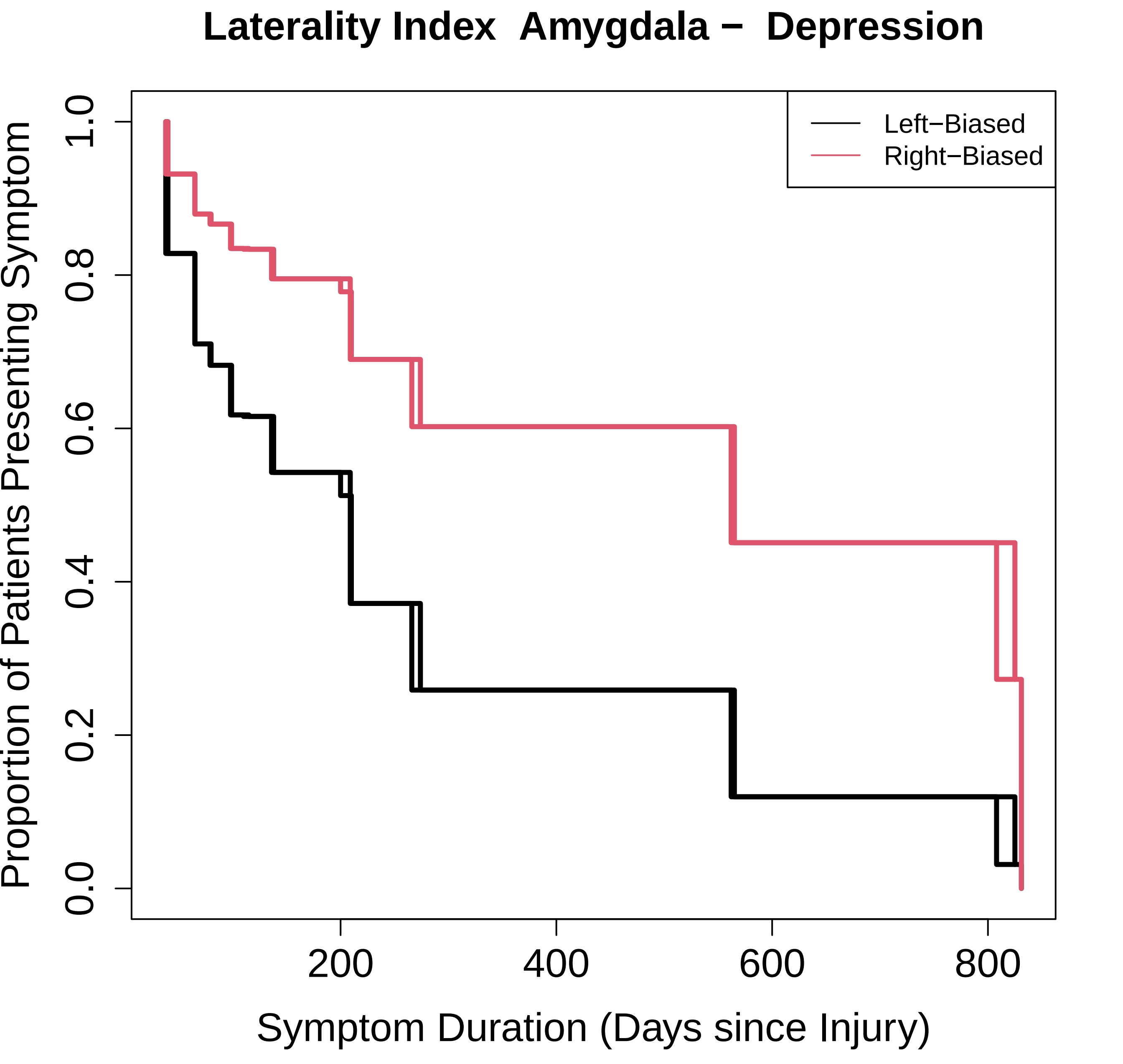
Laterality Index Amygdala − Depression
Left−Biased
Right−Biased
Proportion of Patients Presenting Symptom
0.0
0.2
0.4
0.6
0.8
1.0
200
400
600
800
Symptom Duration (Days since Injury)

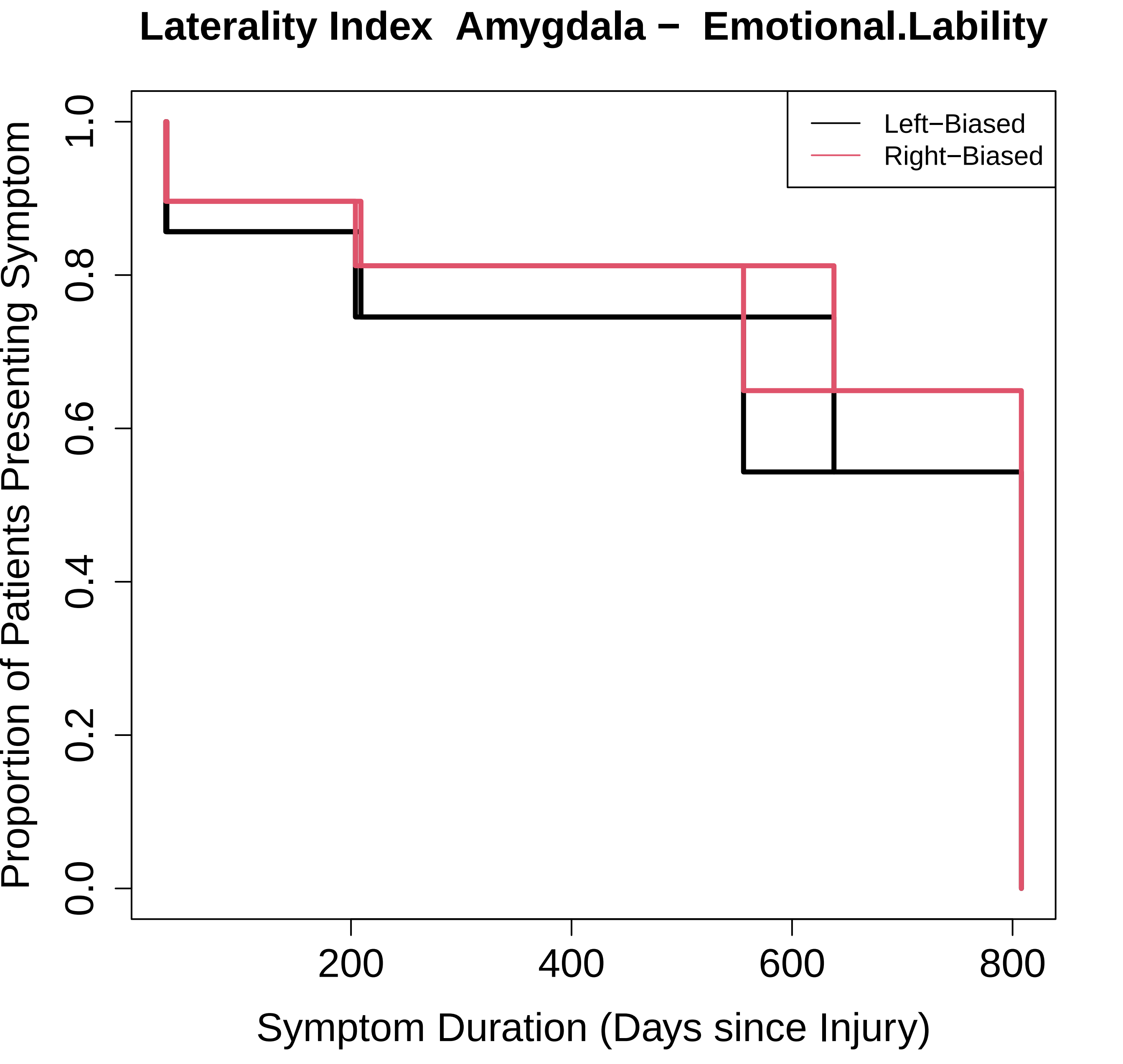

Laterality Index Amygdala − Emotional.Lability
Left−Biased
Right−Biased
Proportion of Patients Presenting Symptom
0.0
0.2
0.4
0.6
0.8
1.0
200
400
600
800
Symptom Duration (Days since Injury)

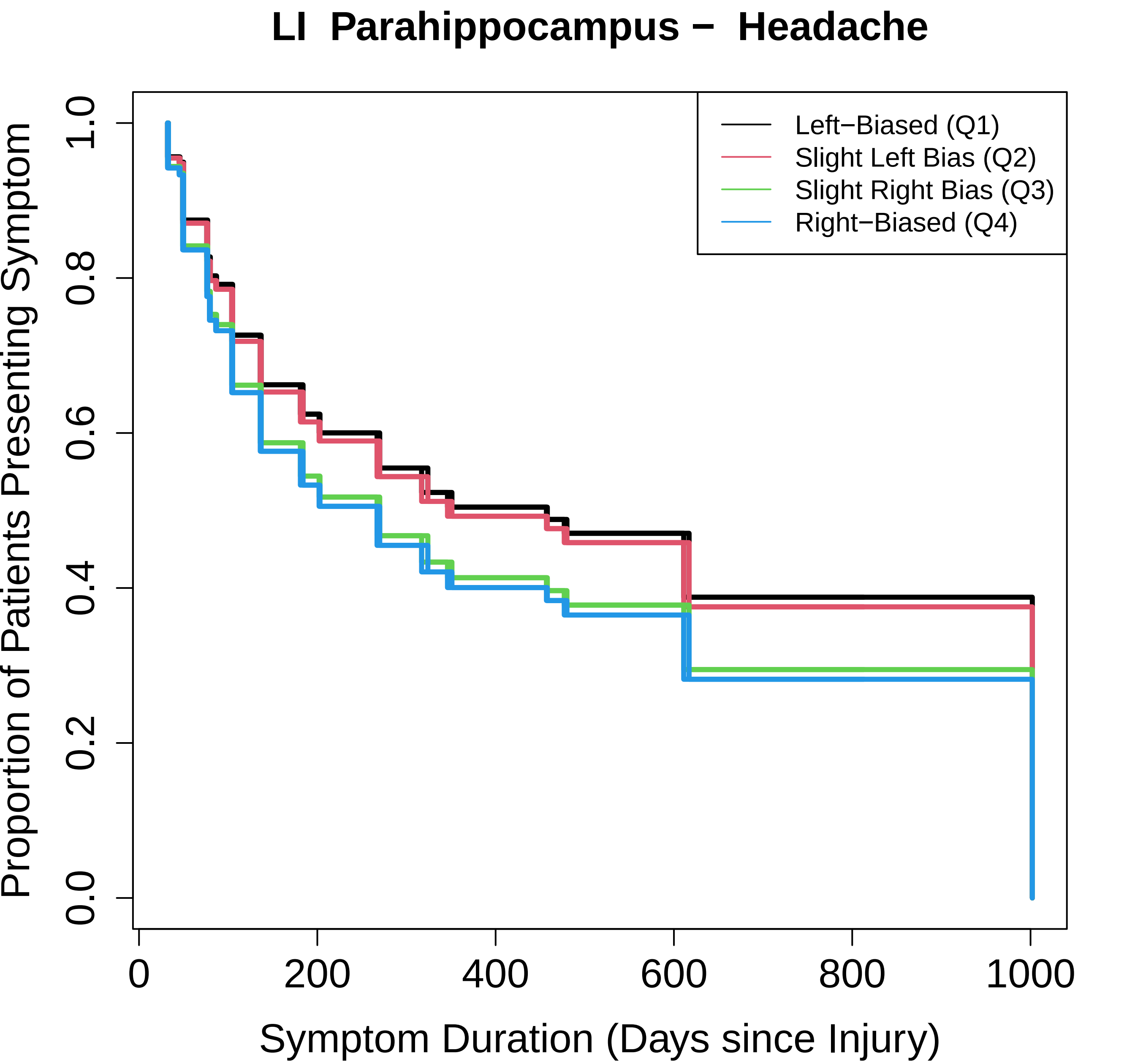

LI Parahippocampus − Headache
Left−Biased (Q1)
Slight Left Bias (Q2)
Slight Right Bias (Q3)
Right−Biased (Q4)
Proportion of Patients Presenting Symptom
0.0
0.2
0.4
0.6
0.8
1.0
0
200
400
600
800
1000
Symptom Duration (Days since Injury)

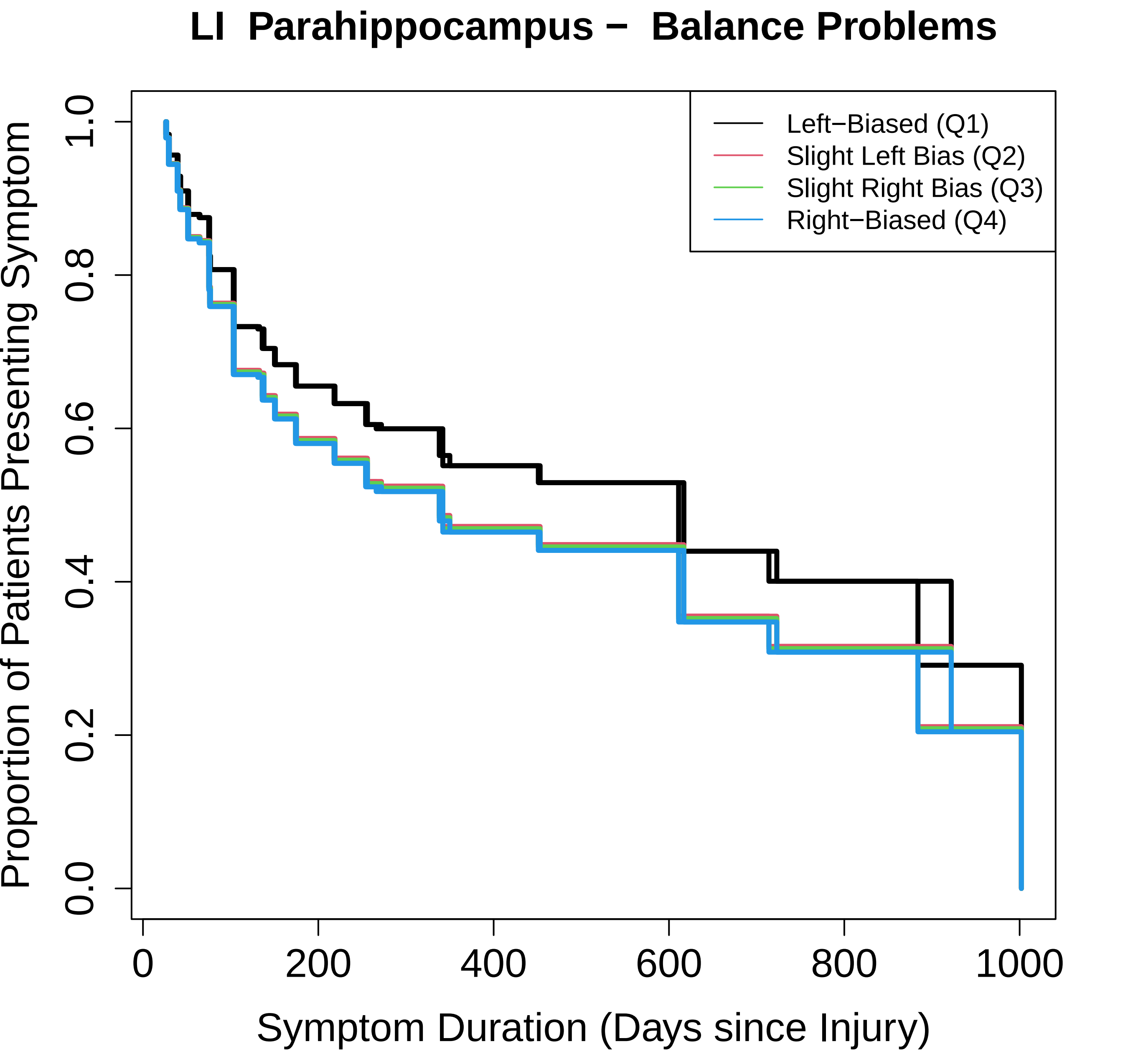

LI Parahippocampus − Balance Problems
Left−Biased (Q1)
Slight Left Bias (Q2)
Slight Right Bias (Q3)
Right−Biased (Q4)
Proportion of Patients Presenting Symptom
0.0
0.2
0.4
0.6
0.8
1.0
0
200
400
600
800
1000
Symptom Duration (Days since Injury)

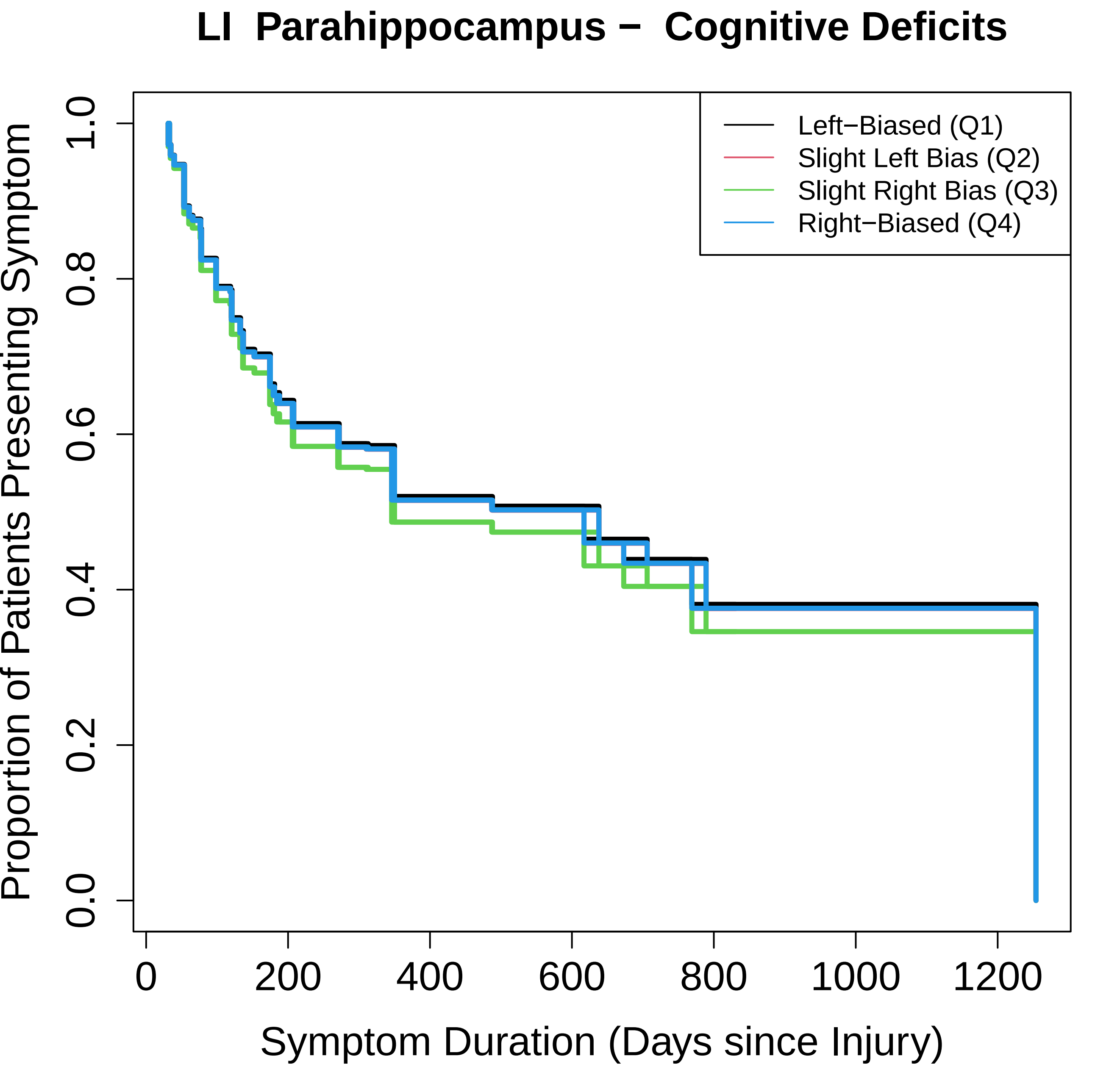
LI Parahippocampus − Cognitive Deficits
Left−Biased (Q1)
Slight Left Bias (Q2)
Slight Right Bias (Q3)
Right−Biased (Q4)
Proportion of Patients Presenting Symptom
0.0
0.2
0.4
0.6
0.8
1.0
0
200
400
600
800
1000
1200
Symptom Duration (Days since Injury)

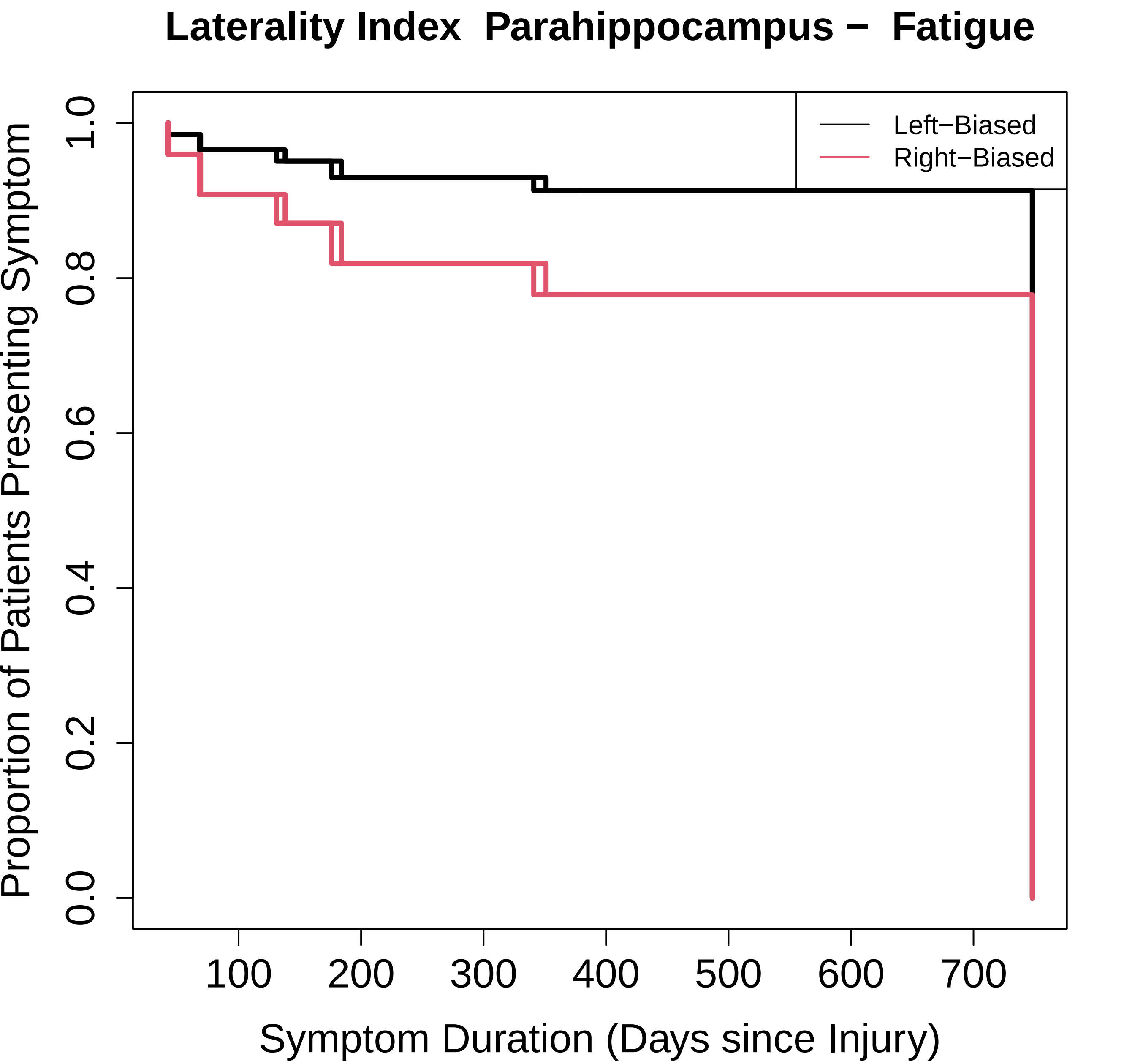
Laterality Index Parahippocampus − Fatigue
Proportion of Patients Presenting Symptom
0.0
0.2
0.4
0.6
0.8
1.0
100
200
300
400
500
600
700
Symptom Duration (Days since Injury)
Left−Biased
Right−Biased

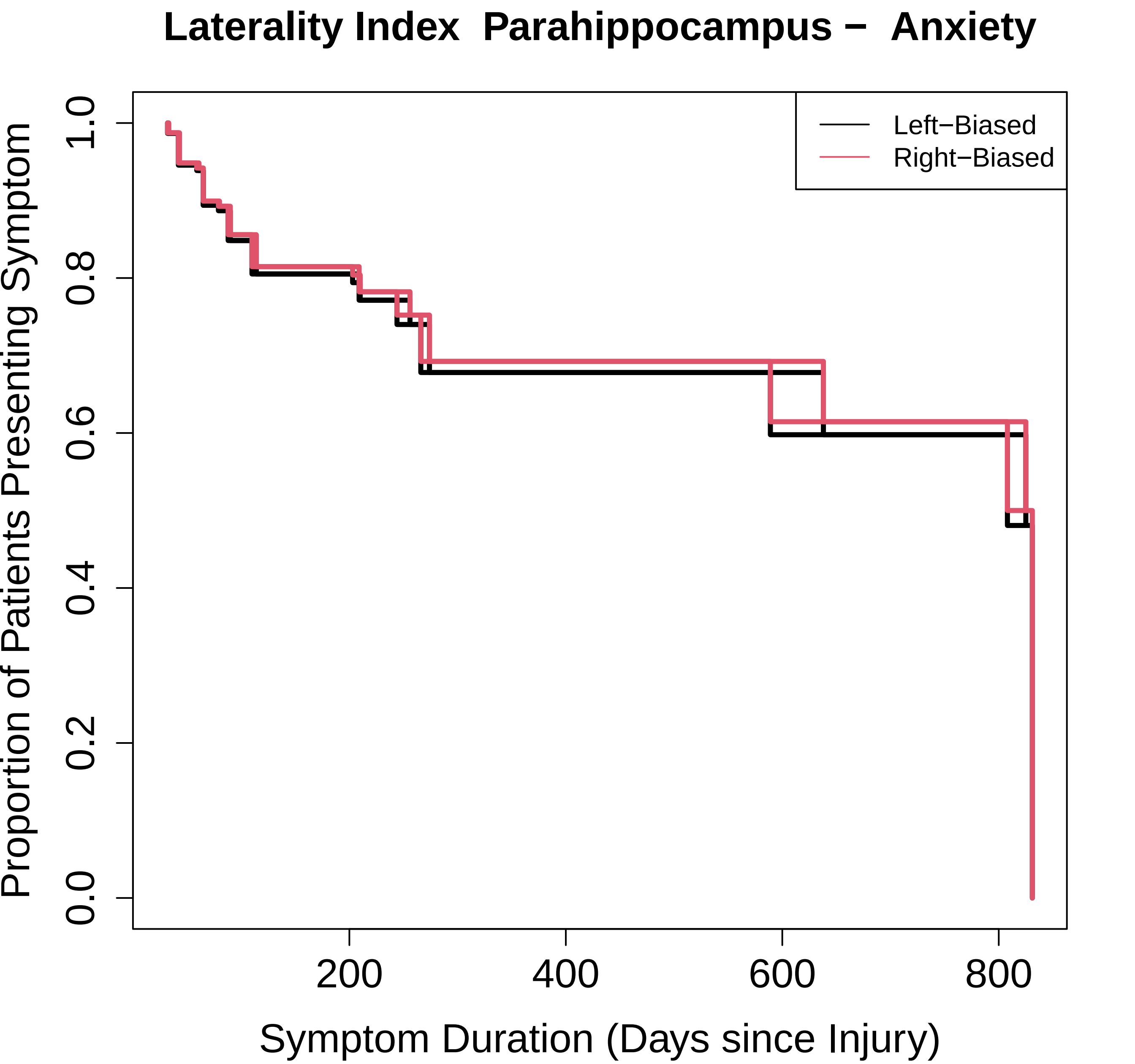

Laterality Index Parahippocampus − Anxiety
Left−Biased
Right−Biased
Proportion of Patients Presenting Symptom
0.0
0.2
0.4
0.6
0.8
1.0
200
400
600
800
Symptom Duration (Days since Injury)

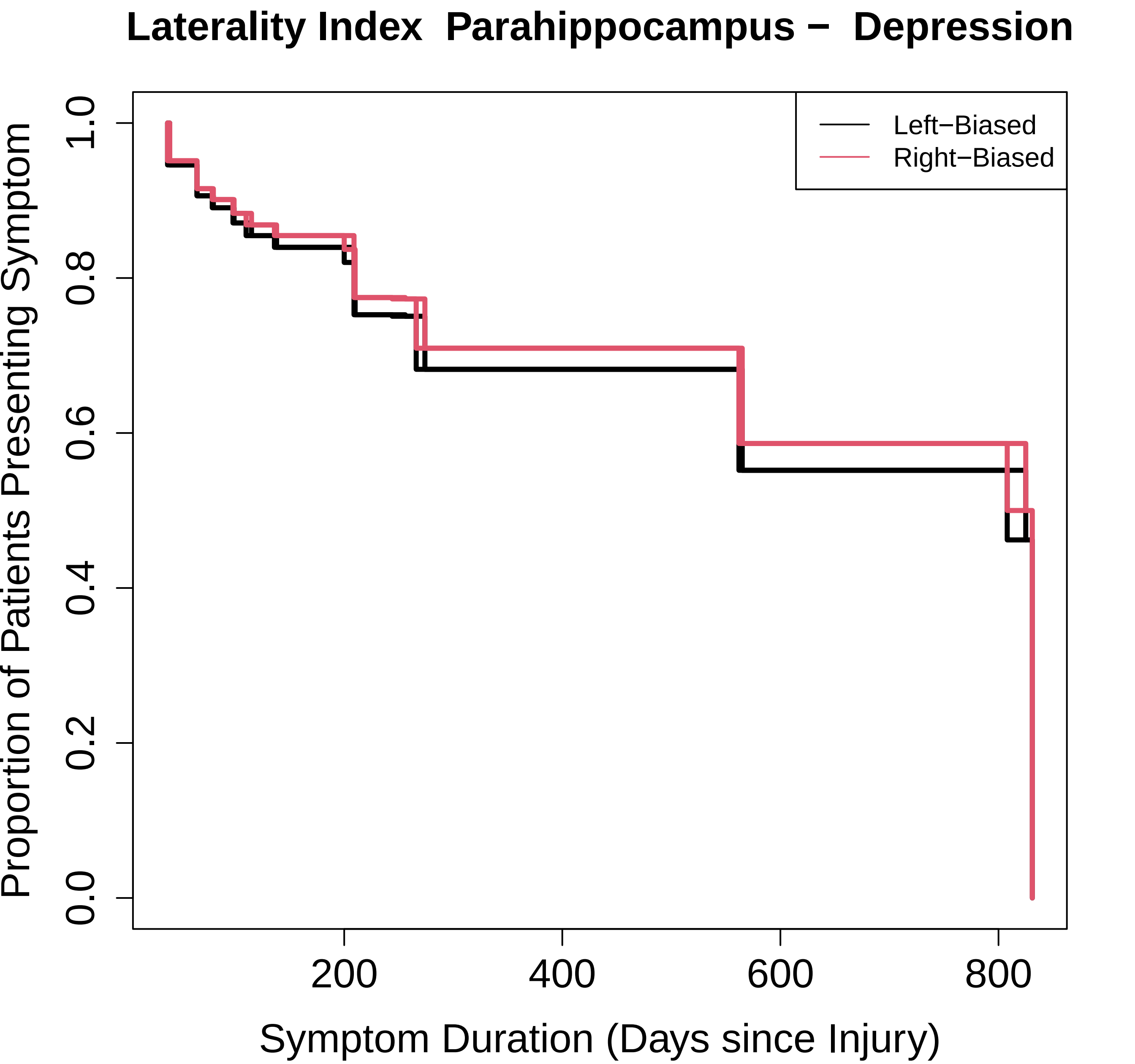
Laterality Index Parahippocampus − Depression
Left−Biased
Right−Biased
Proportion of Patients Presenting Symptom
0.0
0.2
0.4
0.6
0.8
1.0
200
400
600
800
Symptom Duration (Days since Injury)

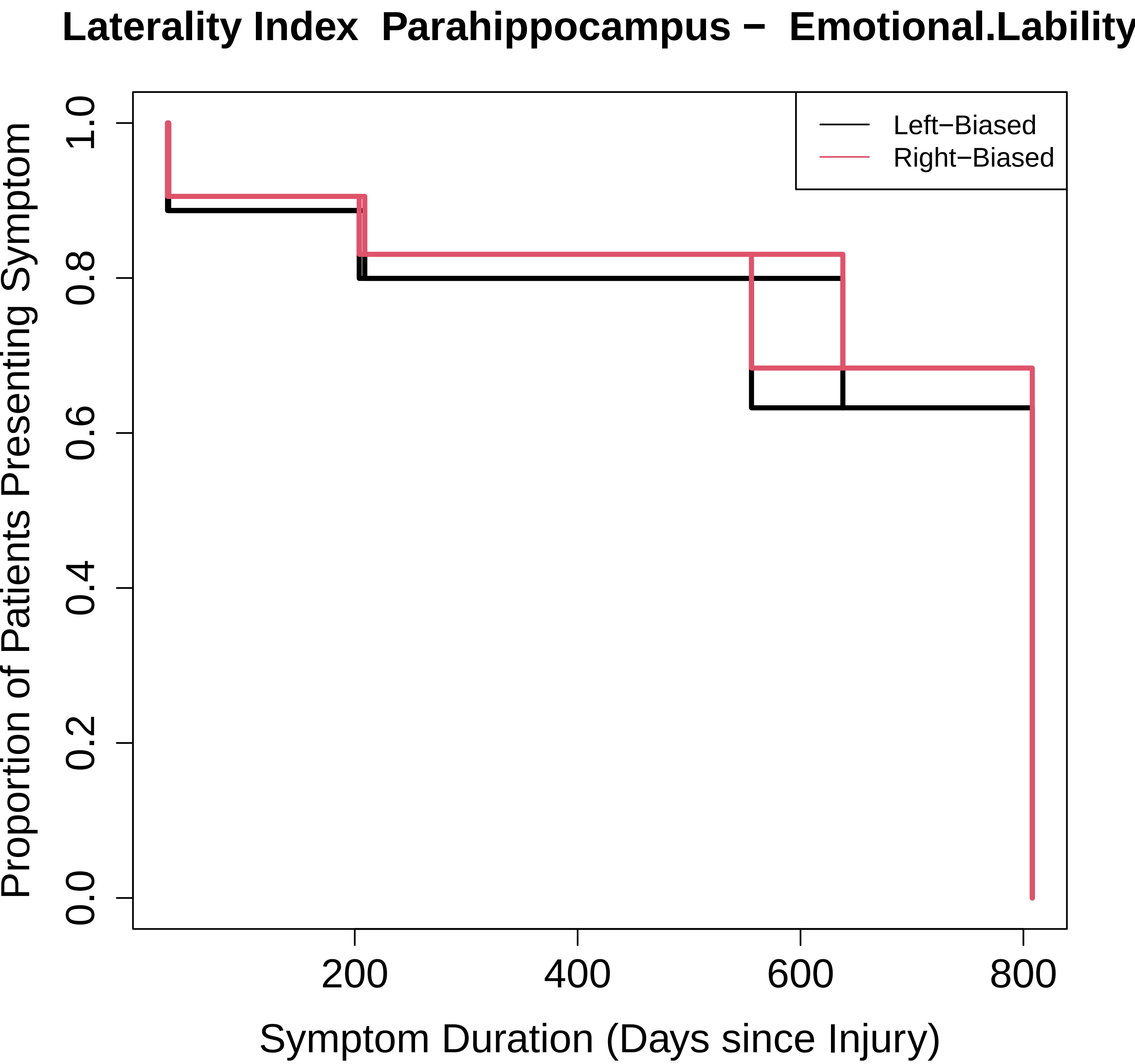

Laterality Index Parahippocampus − Emotional.Lability
Left−Biased
Right−Biased
Proportion of Patients Presenting Symptom
0.0
0.2
0.4
0.6
0.8
1.0
200
400
600
800
Symptom Duration (Days since Injury)

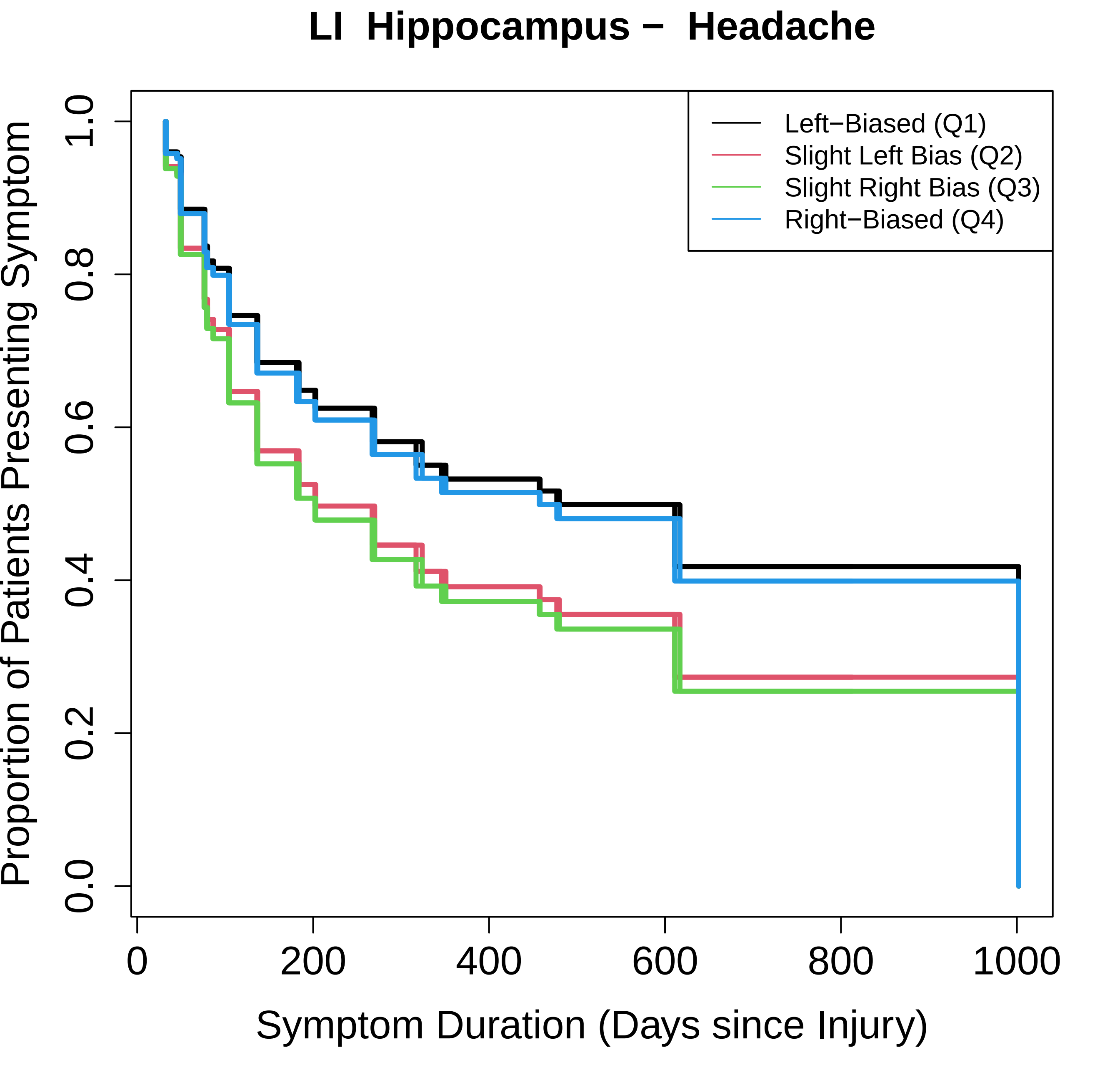

LI Hippocampus − Headache
Left−Biased (Q1)
Slight Left Bias (Q2)
Slight Right Bias (Q3)
Right−Biased (Q4)
Proportion of Patients Presenting Symptom
0.0
0.2
0.4
0.6
0.8
1.0
0
200
400
600
800
1000
Symptom Duration (Days since Injury)

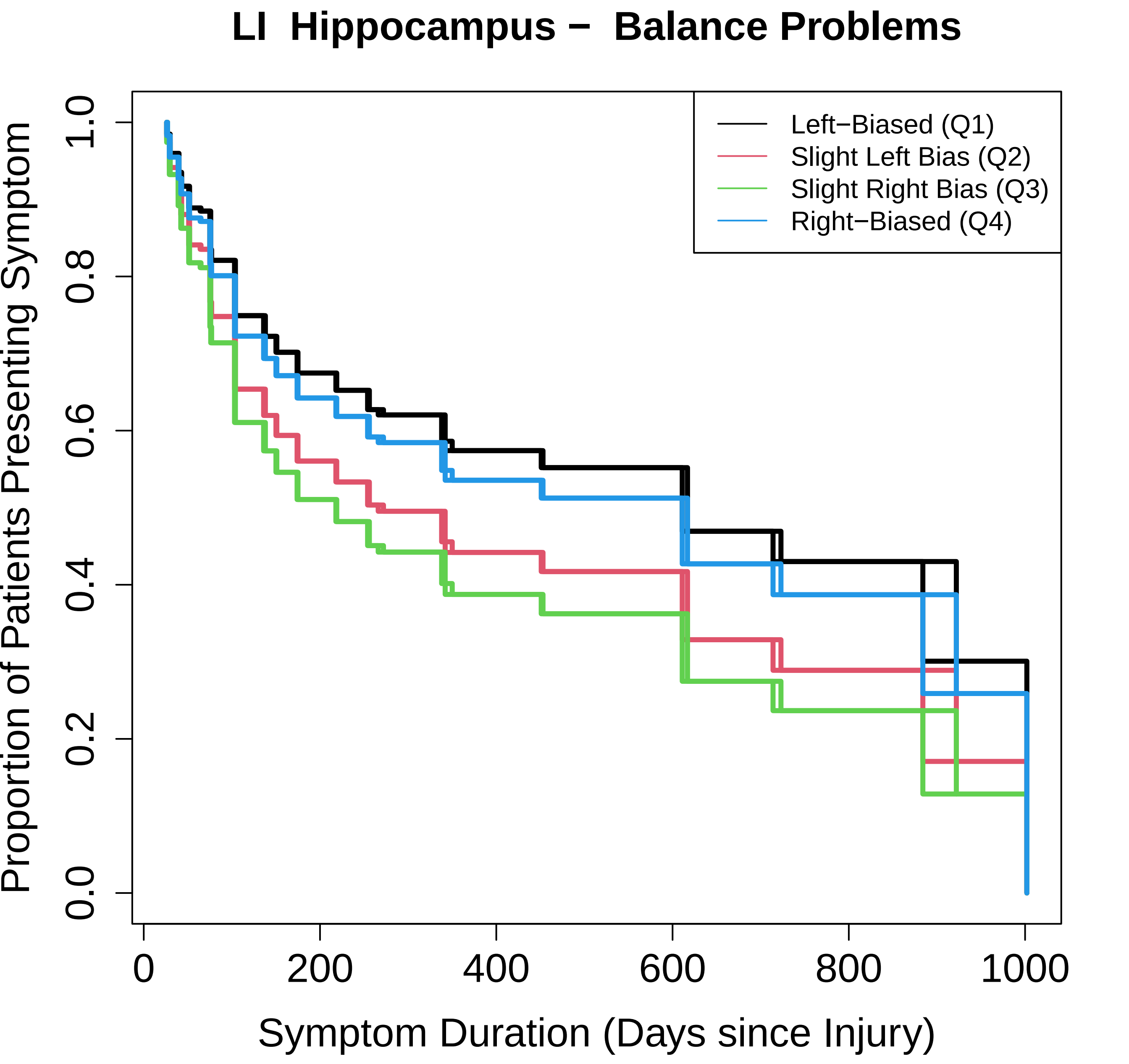
LI Hippocampus − Balance Problems
Left−Biased (Q1)
Slight Left Bias (Q2)
Slight Right Bias (Q3)
Right−Biased (Q4)
Proportion of Patients Presenting Symptom
0.0
0.2
0.4
0.6
0.8
1.0
0
200
400
600
800
1000
Symptom Duration (Days since Injury)

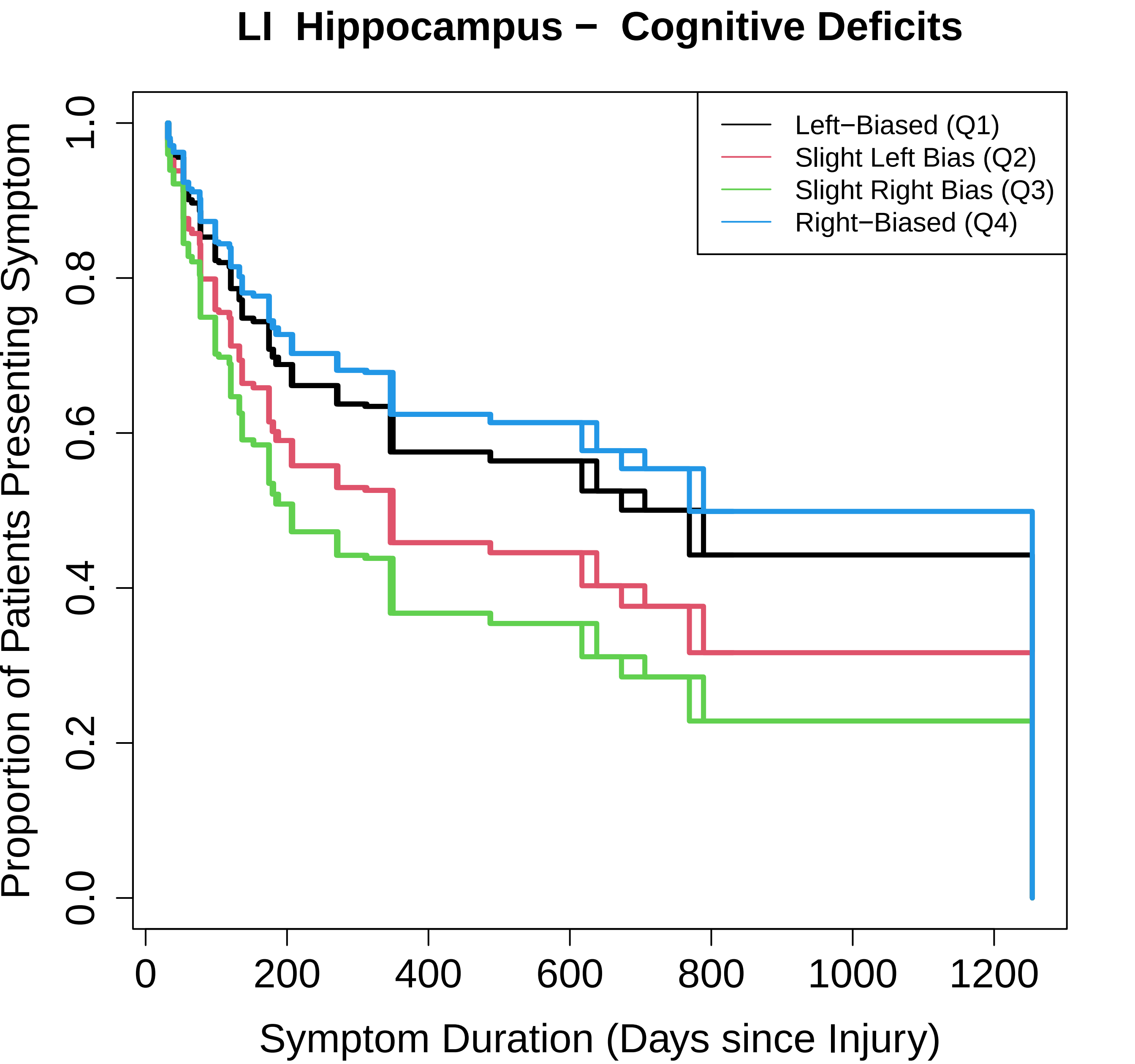

LI Hippocampus − Cognitive Deficits
Left−Biased (Q1)
Slight Left Bias (Q2)
Slight Right Bias (Q3)
Right−Biased (Q4)
Proportion of Patients Presenting Symptom
0.0
0.2
0.4
0.6
0.8
1.0
0
200
400
600
800
1000
1200
Symptom Duration (Days since Injury)

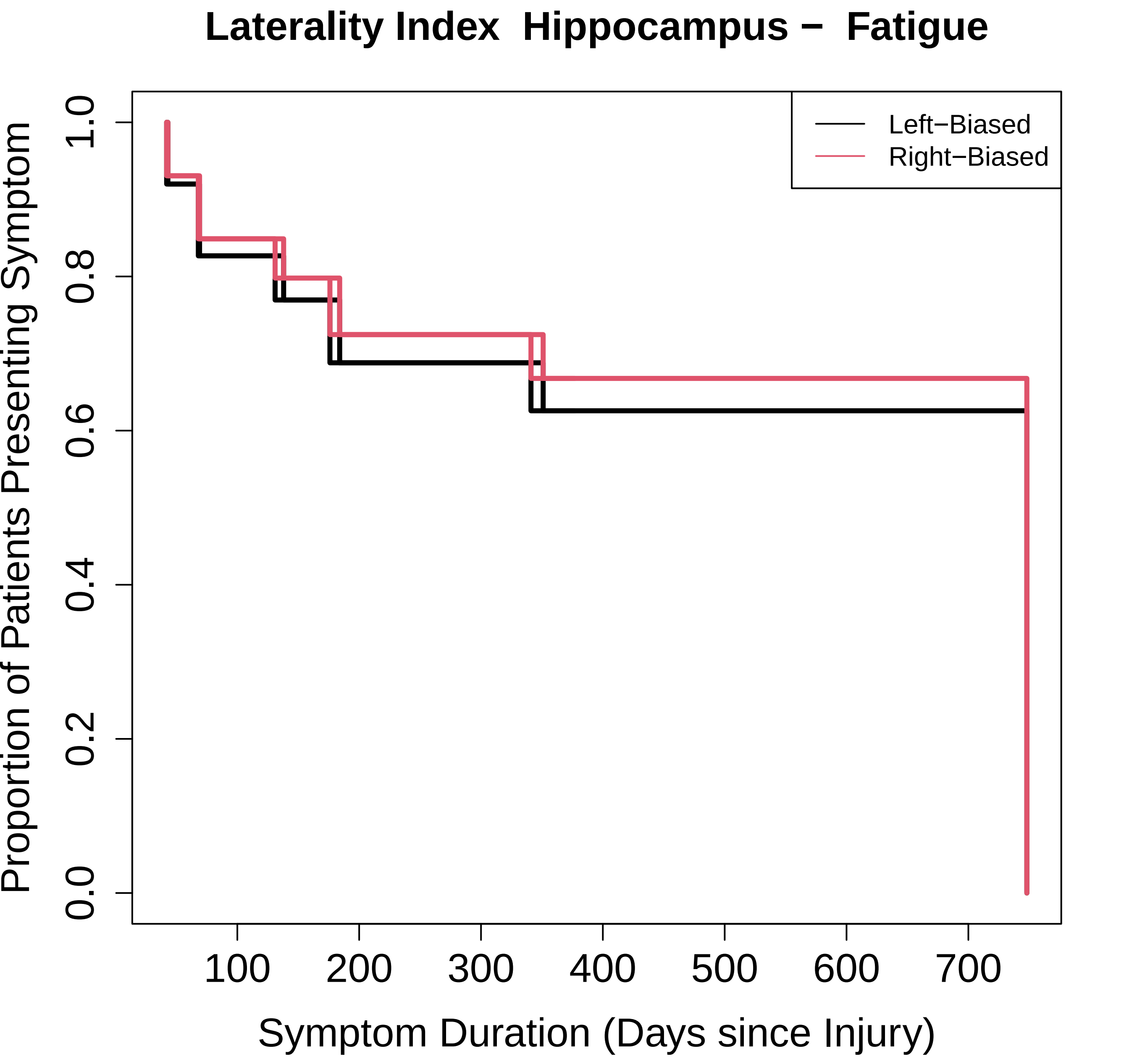

Laterality Index Hippocampus − Fatigue
Left−Biased
Right−Biased
Proportion of Patients Presenting Symptom
0.0
0.2
0.4
0.6
0.8
1.0
100
200
300
400
500
600
700
Symptom Duration (Days since Injury)

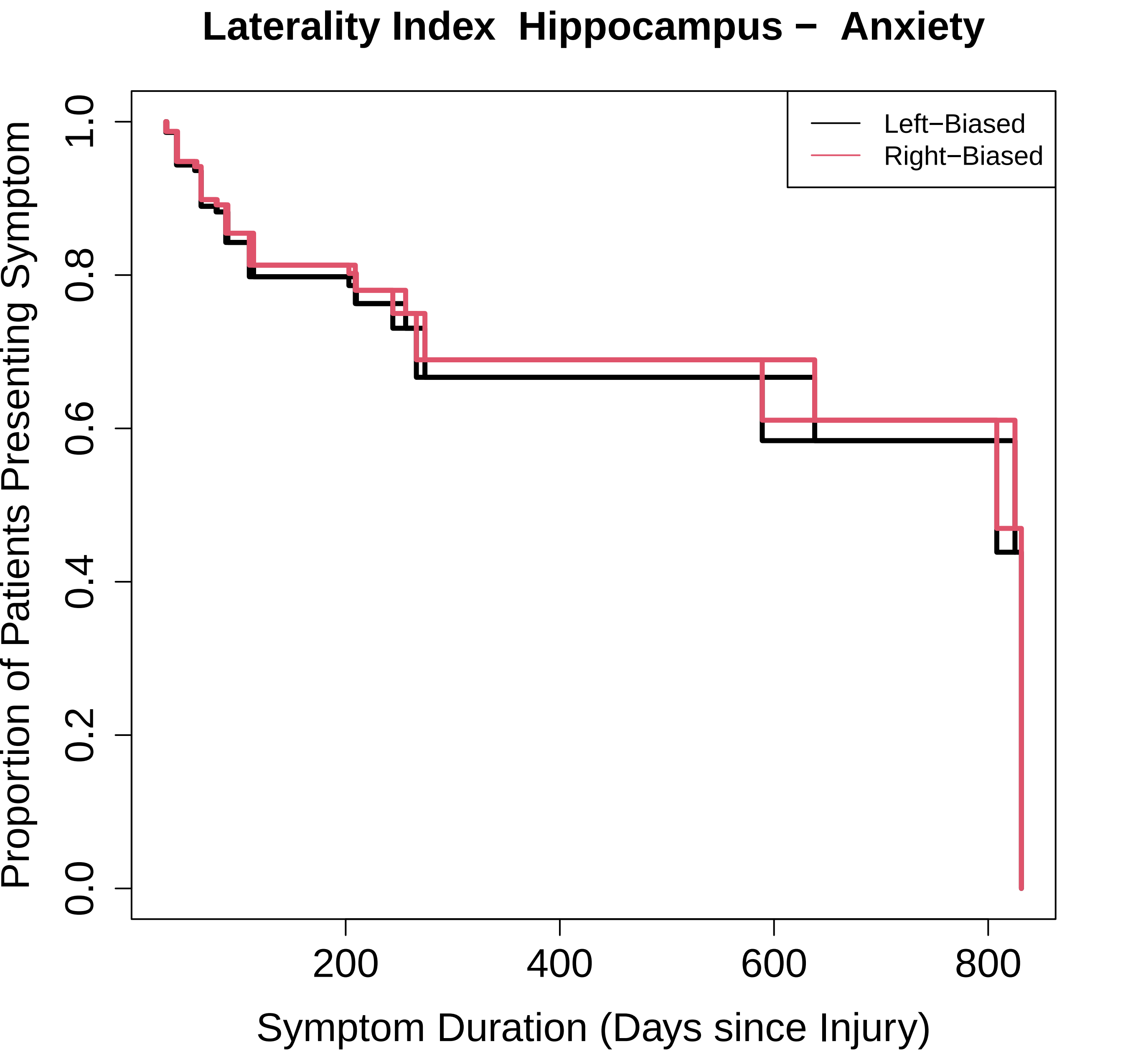

Laterality Index Hippocampus − Anxiety
Left−Biased
Right−Biased
Proportion of Patients Presenting Symptom
0.0
0.2
0.4
0.6
0.8
1.0
200
400
600
800
Symptom Duration (Days since Injury)

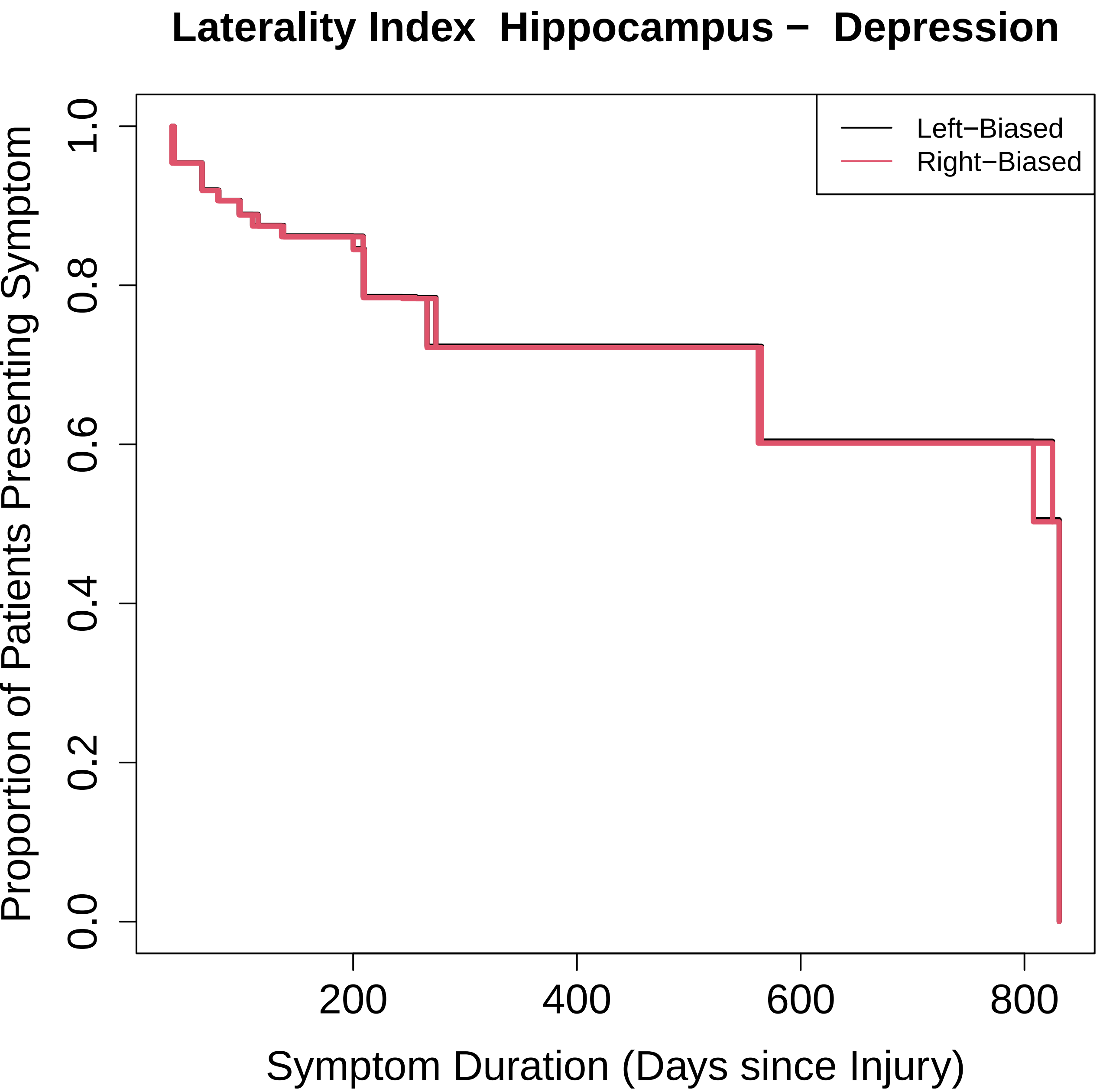

Laterality Index Hippocampus − Depression
Left−Biased
Right−Biased
Proportion of Patients Presenting Symptom
0.0
0.2
0.4
0.6
0.8
1.0
200
400
600
800
Symptom Duration (Days since Injury)

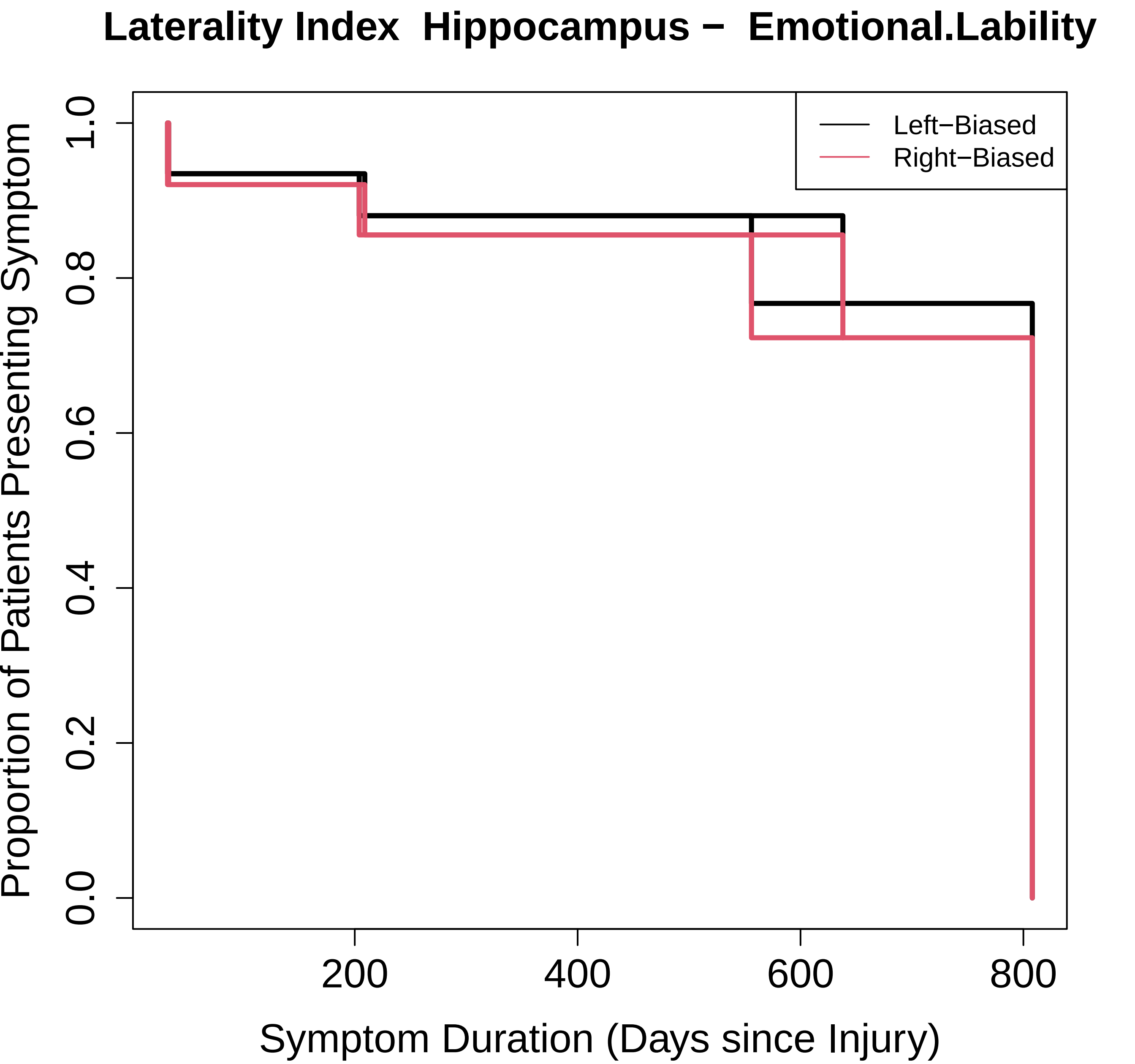

Laterality Index Hippocampus − Emotional.Lability
Left−Biased
Right−Biased
Proportion of Patients Presenting Symptom
0.0
0.2
0.4
0.6
0.8
1.0
200
400
600
800
Symptom Duration (Days since Injury)

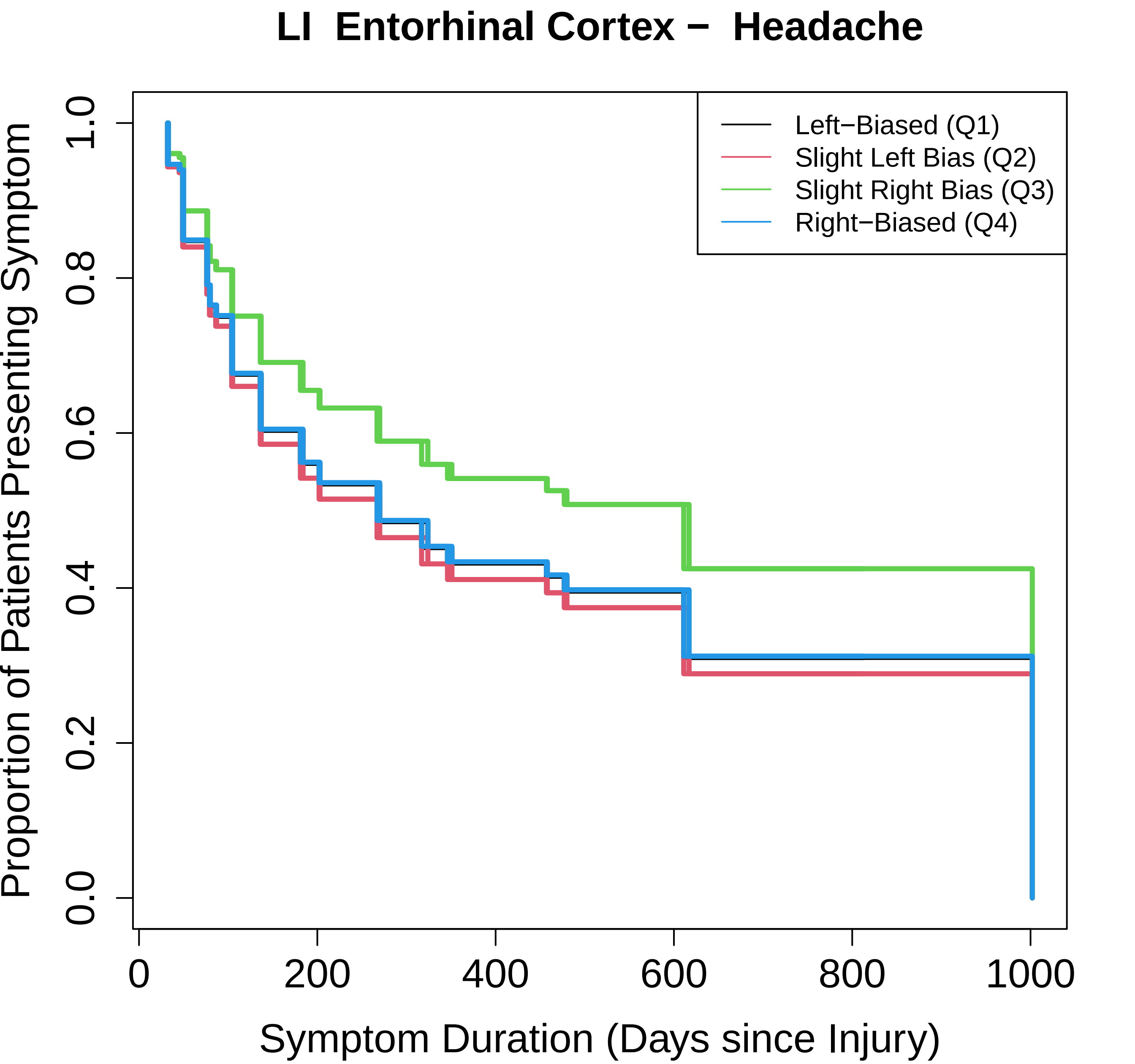

LI Entorhinal Cortex − Headache
Proportion of Patients Presenting Symptom
Symptom Duration (Days since Injury)
0.0
0.2
0.4
0.6
0.8
1.0
0
200
400
600
800
1000
Left−Biased (Q1)
Slight Left Bias (Q2)
Slight Right Bias (Q3)
Right−Biased (Q4)

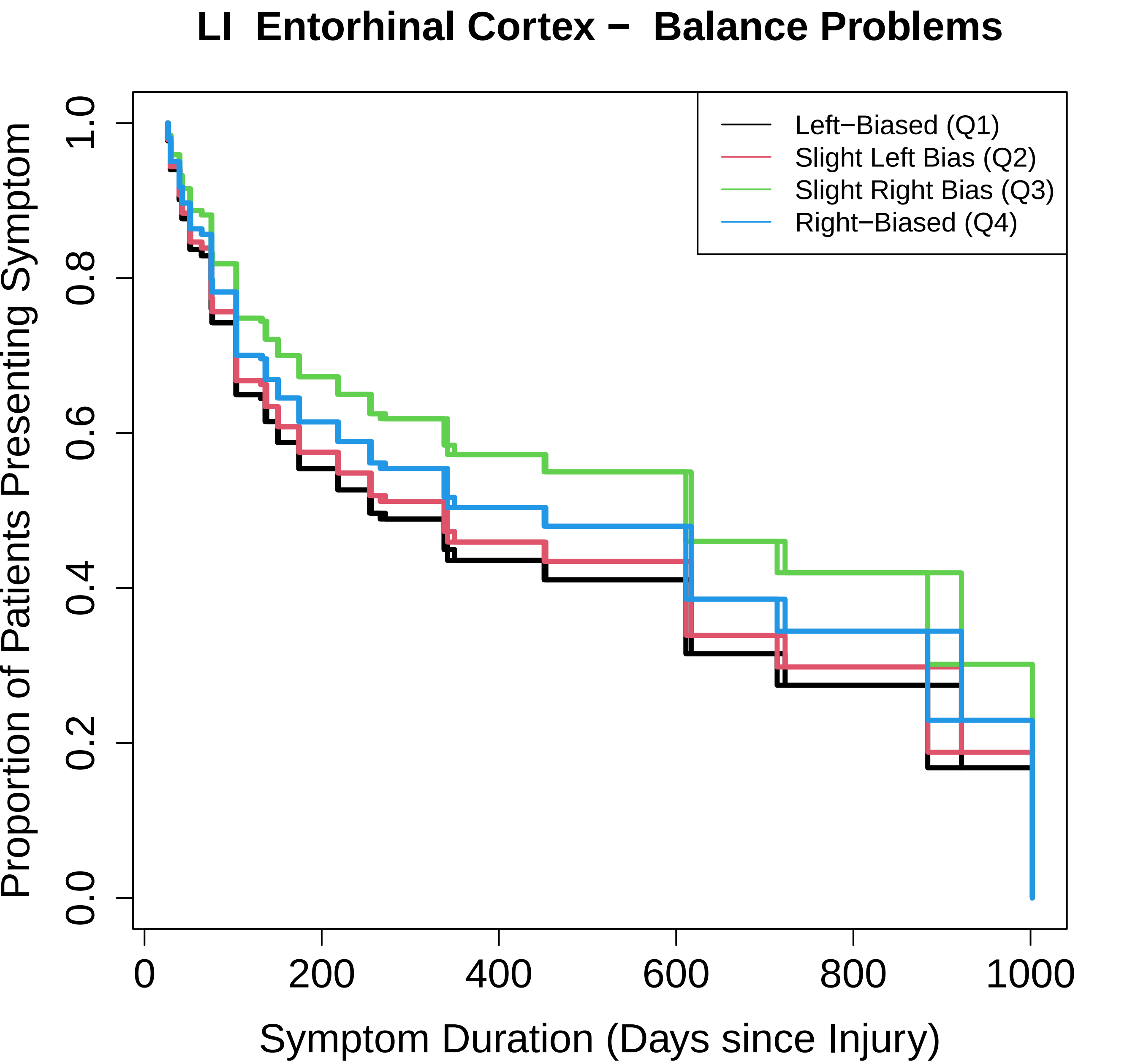

LI Entorhinal Cortex − Balance Problems
Left−Biased (Q1)
Slight Left Bias (Q2)
Slight Right Bias (Q3)
Right−Biased (Q4)
Proportion of Patients Presenting Symptom
0.0
0.2
0.4
0.6
0.8
1.0
0
200
400
600
800
1000
Symptom Duration (Days since Injury)

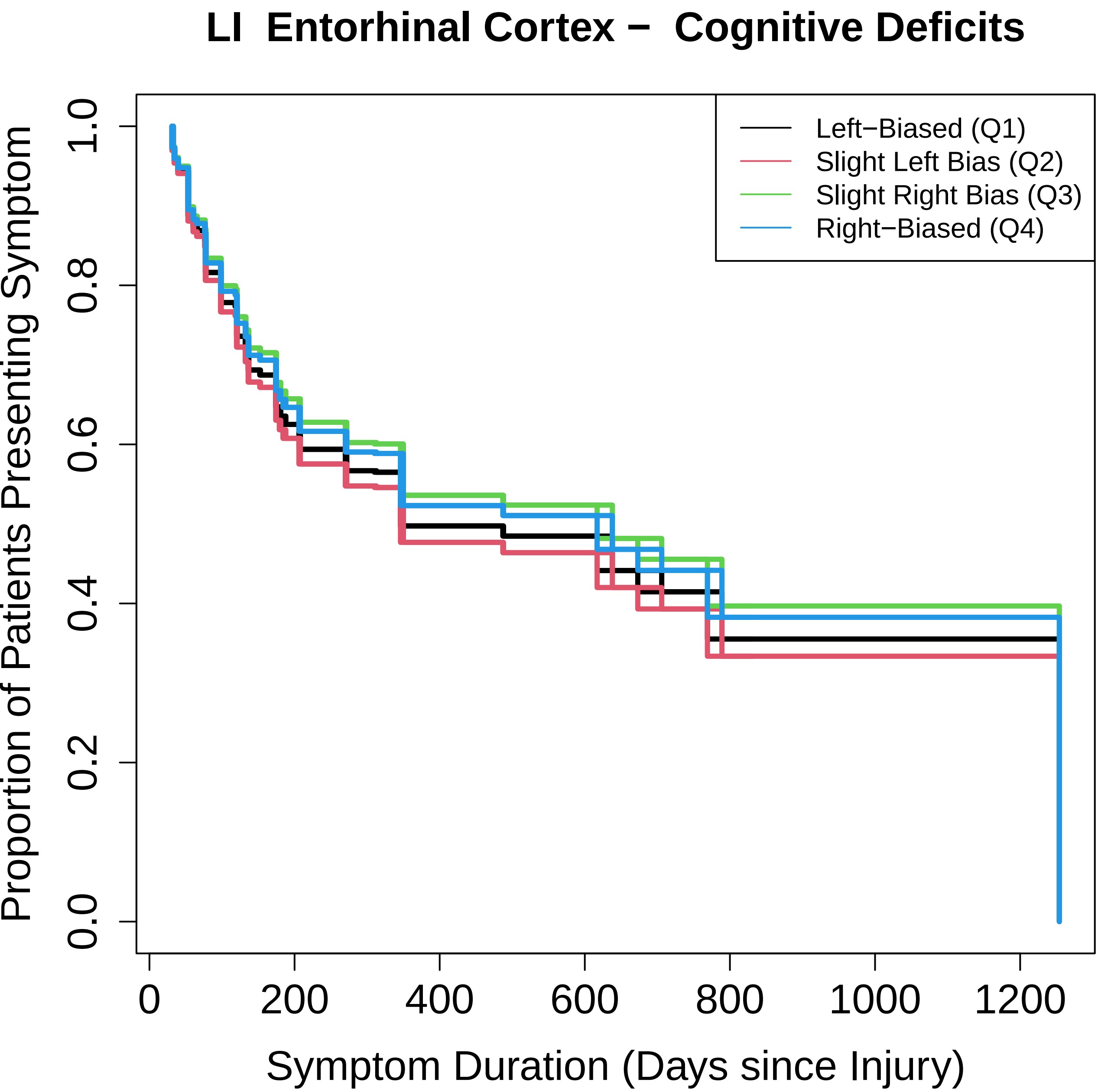

LI Entorhinal Cortex − Cognitive Deficits
Proportion of Patients Presenting Symptom
Symptom Duration (Days since Injury)
Left−Biased (Q1)
Slight Left Bias (Q2)
Slight Right Bias (Q3)
Right−Biased (Q4)
0.0
0.2
0.4
0.6
0.8
1.0
0
200
400
600
800
1000
1200

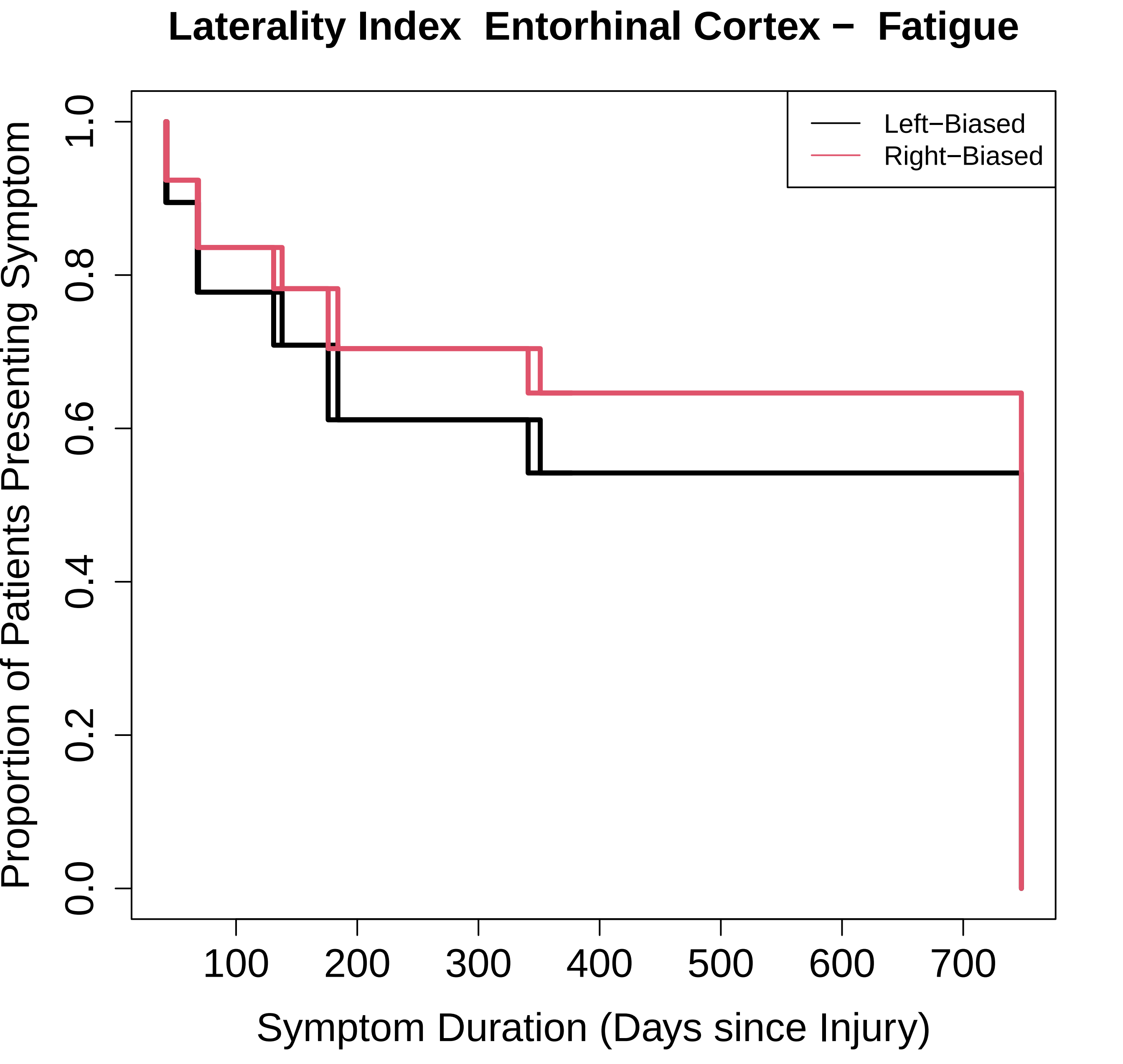

Laterality Index Entorhinal Cortex − Fatigue
Left−Biased
Right−Biased
Proportion of Patients Presenting Symptom
0.0
0.2
0.4
0.6
0.8
1.0
100
200
300
400
500
600
700
Symptom Duration (Days since Injury)

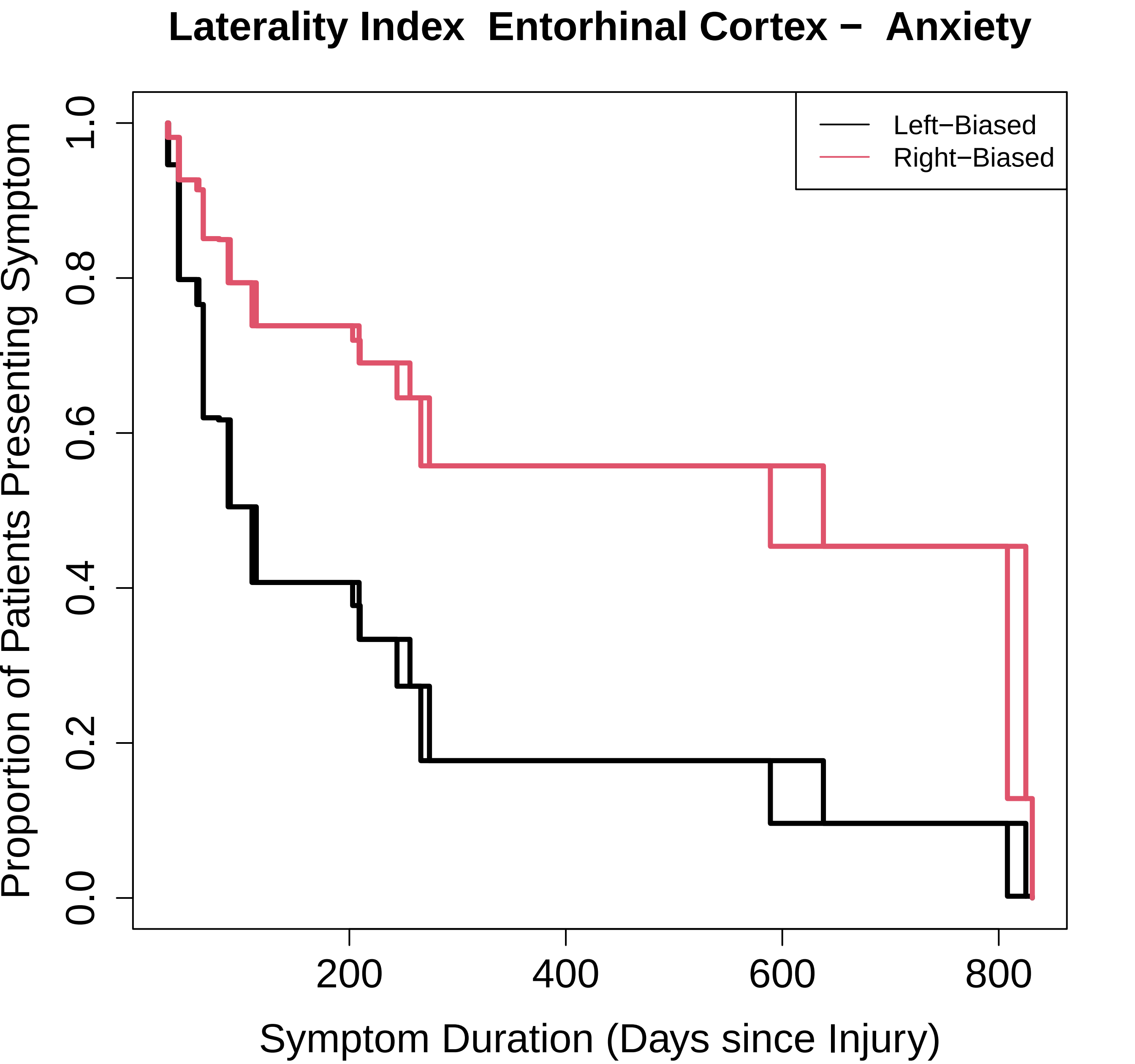

Laterality Index Entorhinal Cortex − Anxiety
Left−Biased
Right−Biased
Proportion of Patients Presenting Symptom
Symptom Duration (Days since Injury)
0.0
0.2
0.4
0.6
0.8
1.0
200
400
600
800

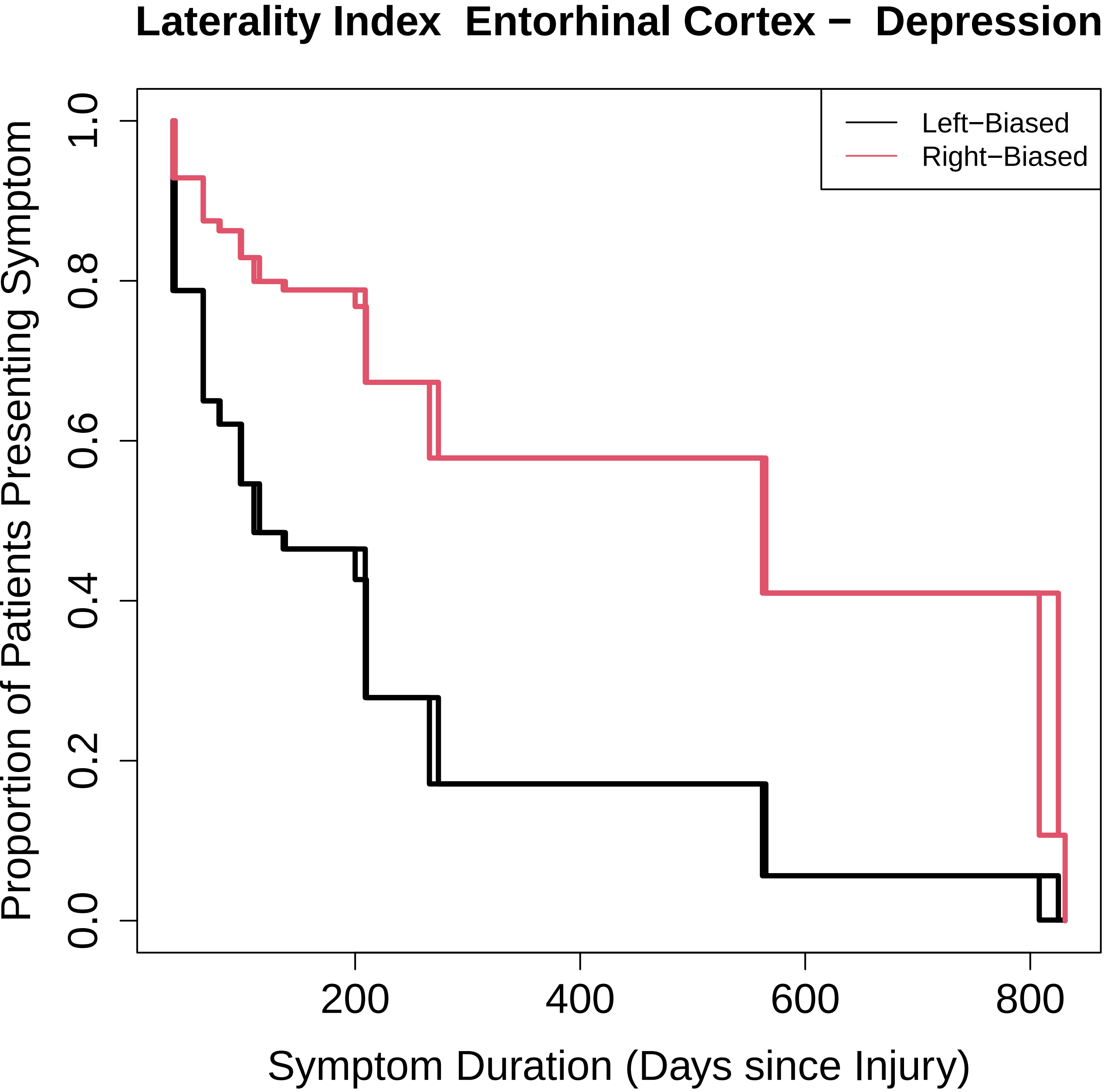

Laterality Index Entorhinal Cortex − Depression
Left−Biased
Right−Biased
Proportion of Patients Presenting Symptom
0.0
0.2
0.4
0.6
0.8
1.0
200
400
600
800
Symptom Duration (Days since Injury)

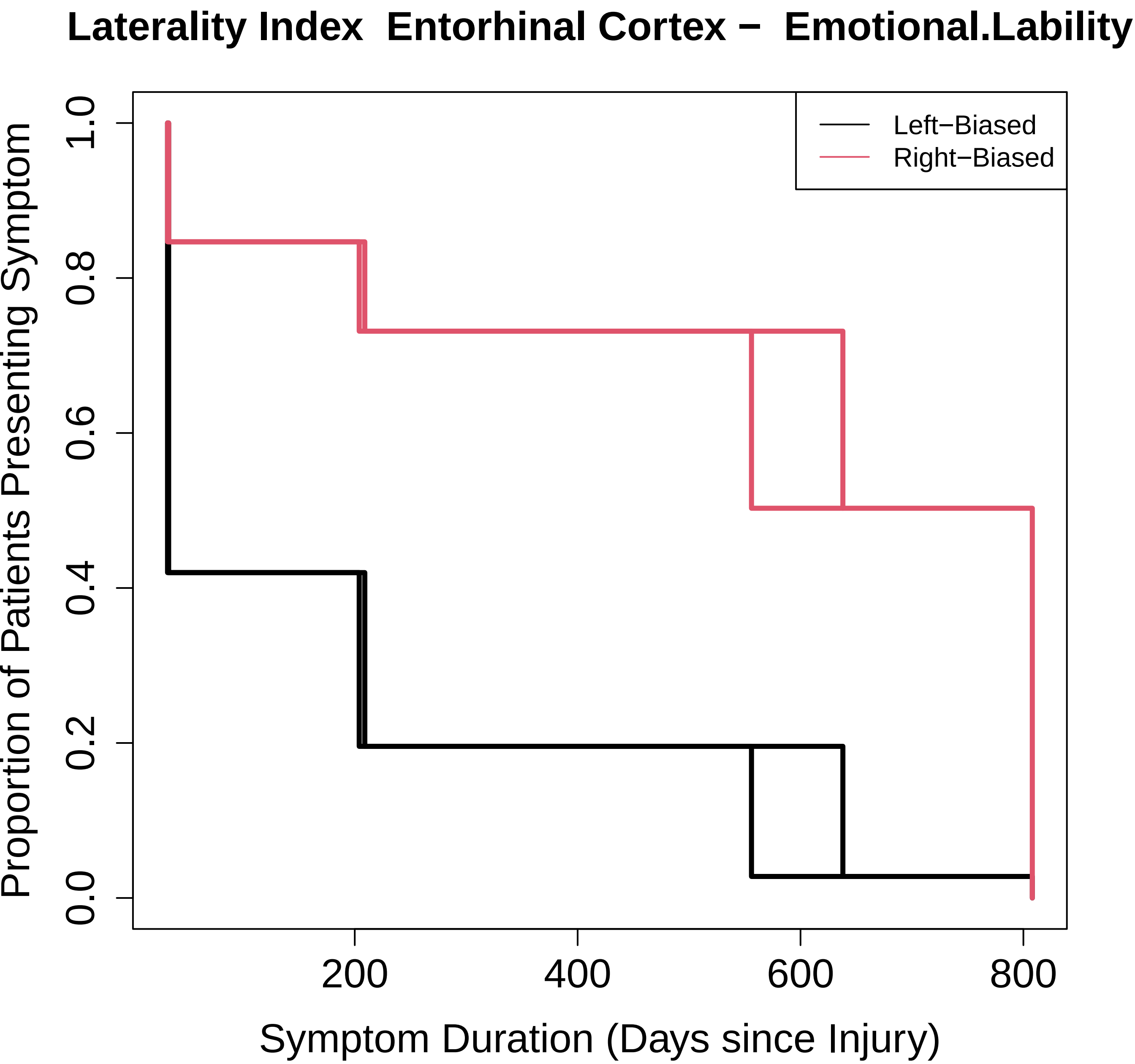

Laterality Index Entorhinal Cortex − Emotional.Lability
Left−Biased
Right−Biased
Proportion of Patients Presenting Symptom
0.0
0.2
0.4
0.6
0.8
1.0
200
400
600
800
Symptom Duration (Days since Injury)

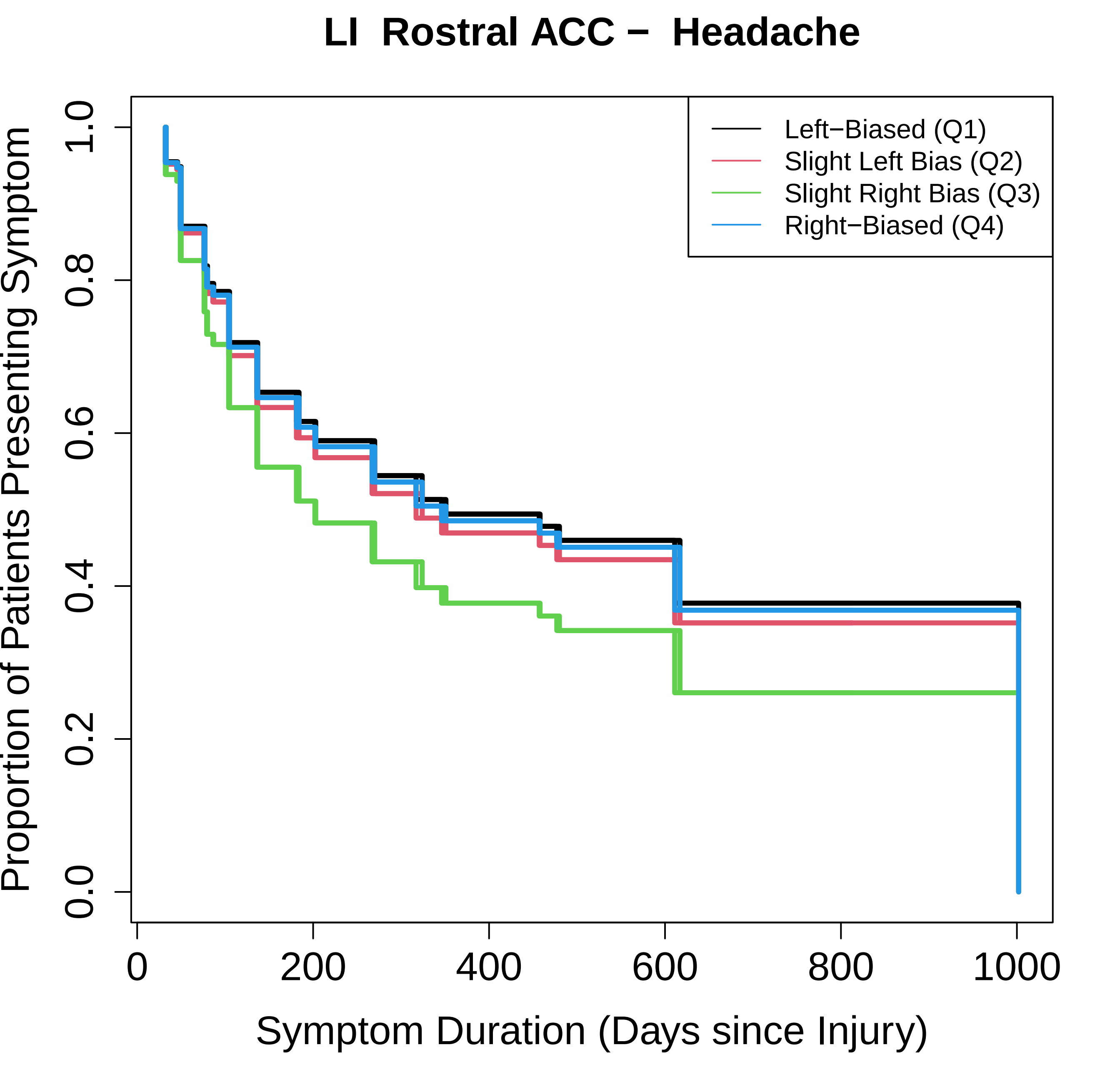

LI Rostral ACC − Headache
Proportion of Patients Presenting Symptom
Symptom Duration (Days since Injury)
0.0
0.2
0.4
0.6
0.8
1.0
0
200
400
600
800
1000
Left−Biased (Q1)
Slight Left Bias (Q2)
Slight Right Bias (Q3)
Right−Biased (Q4)

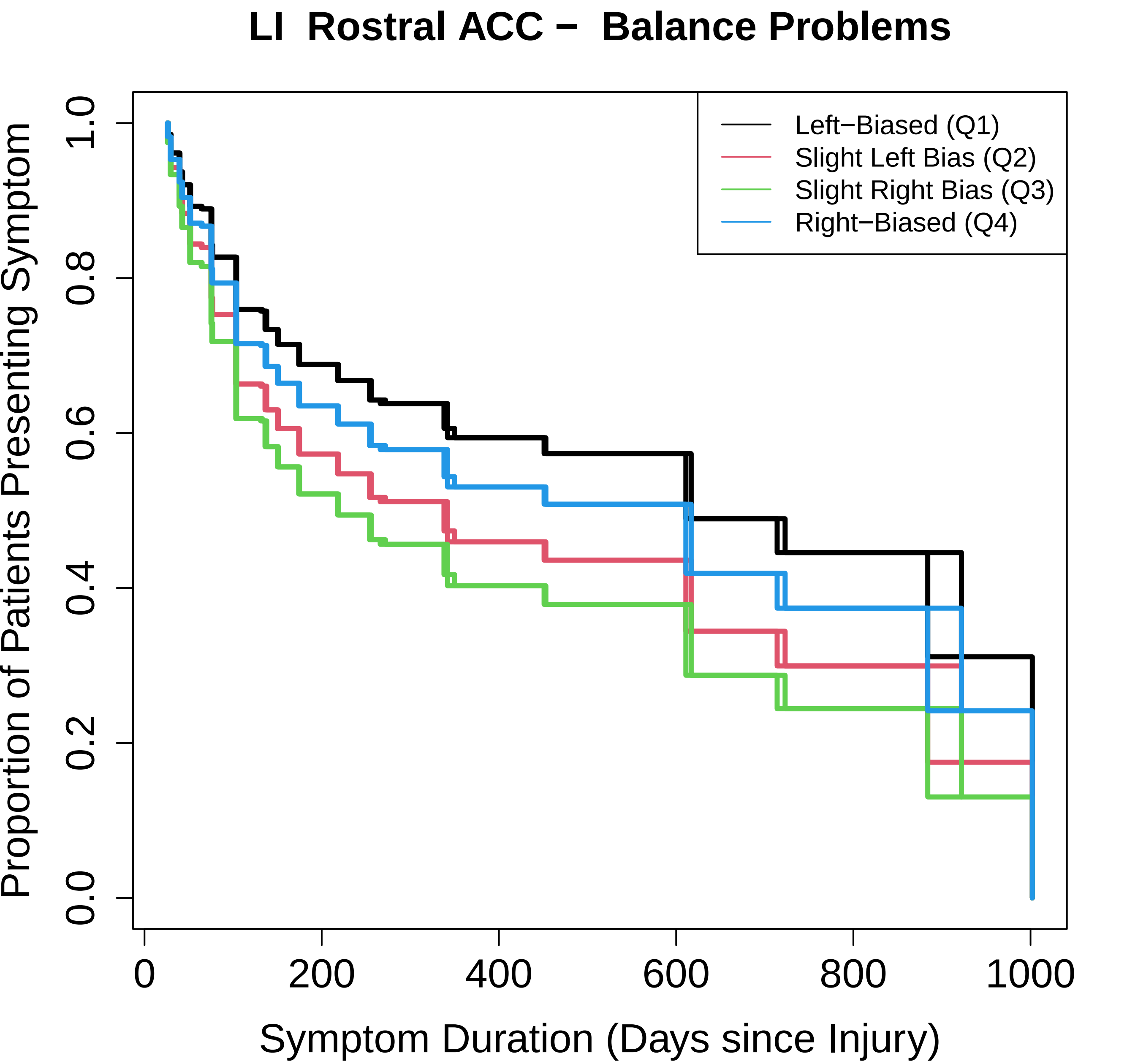
LI Rostral ACC − Balance Problems
Proportion of Patients Presenting Symptom
Symptom Duration (Days since Injury)
0.0
0.2
0.4
0.6
0.8
1.0
0
200
400
600
800
1000
Left−Biased (Q1)
Slight Left Bias (Q2)
Slight Right Bias (Q3)
Right−Biased (Q4)

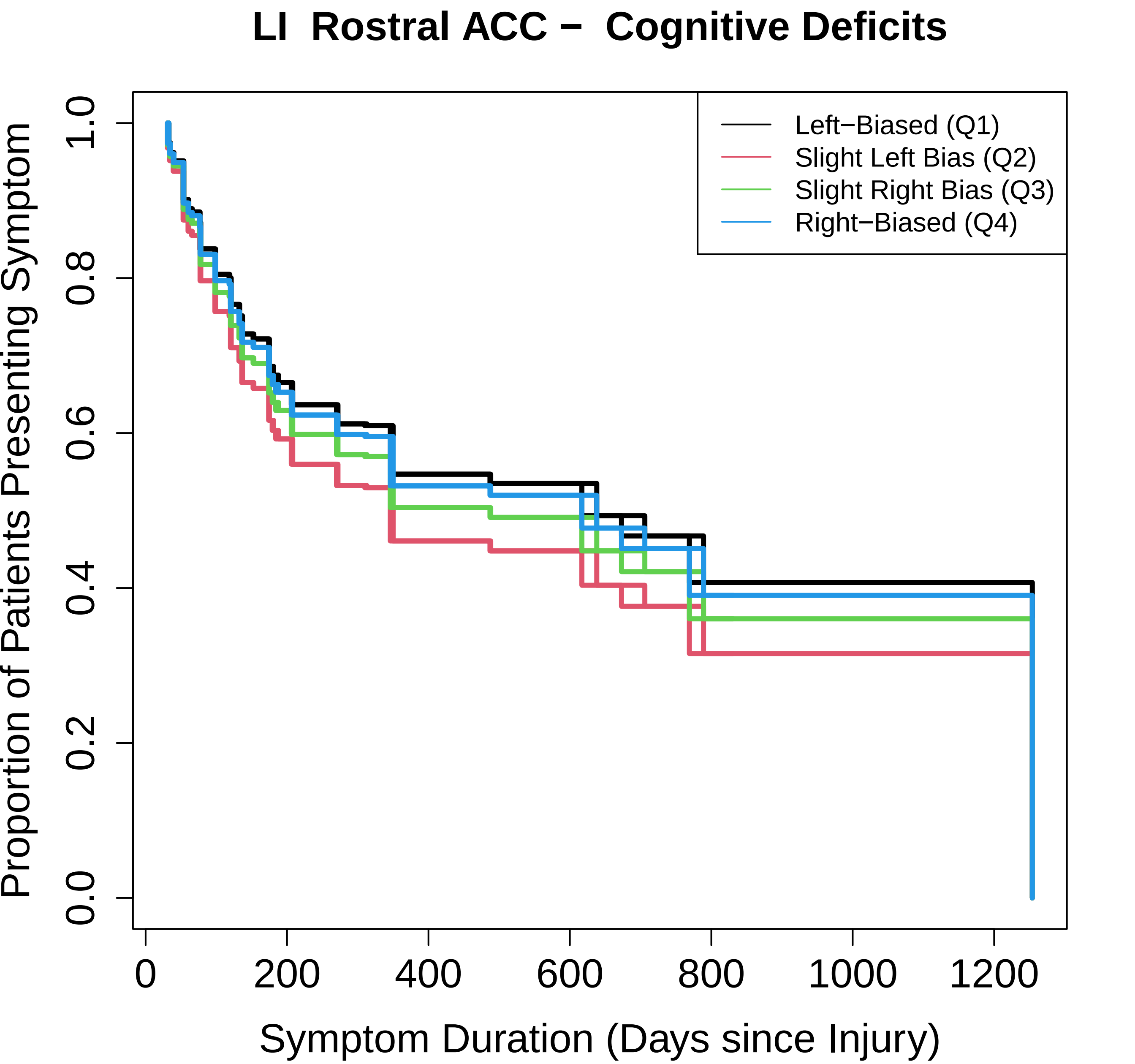

LI Rostral ACC − Cognitive Deficits
Left−Biased (Q1)
Slight Left Bias (Q2)
Slight Right Bias (Q3)
Right−Biased (Q4)
Proportion of Patients Presenting Symptom
0.0
0.2
0.4
0.6
0.8
1.0
0
200
400
600
800
1000
1200
Symptom Duration (Days since Injury)

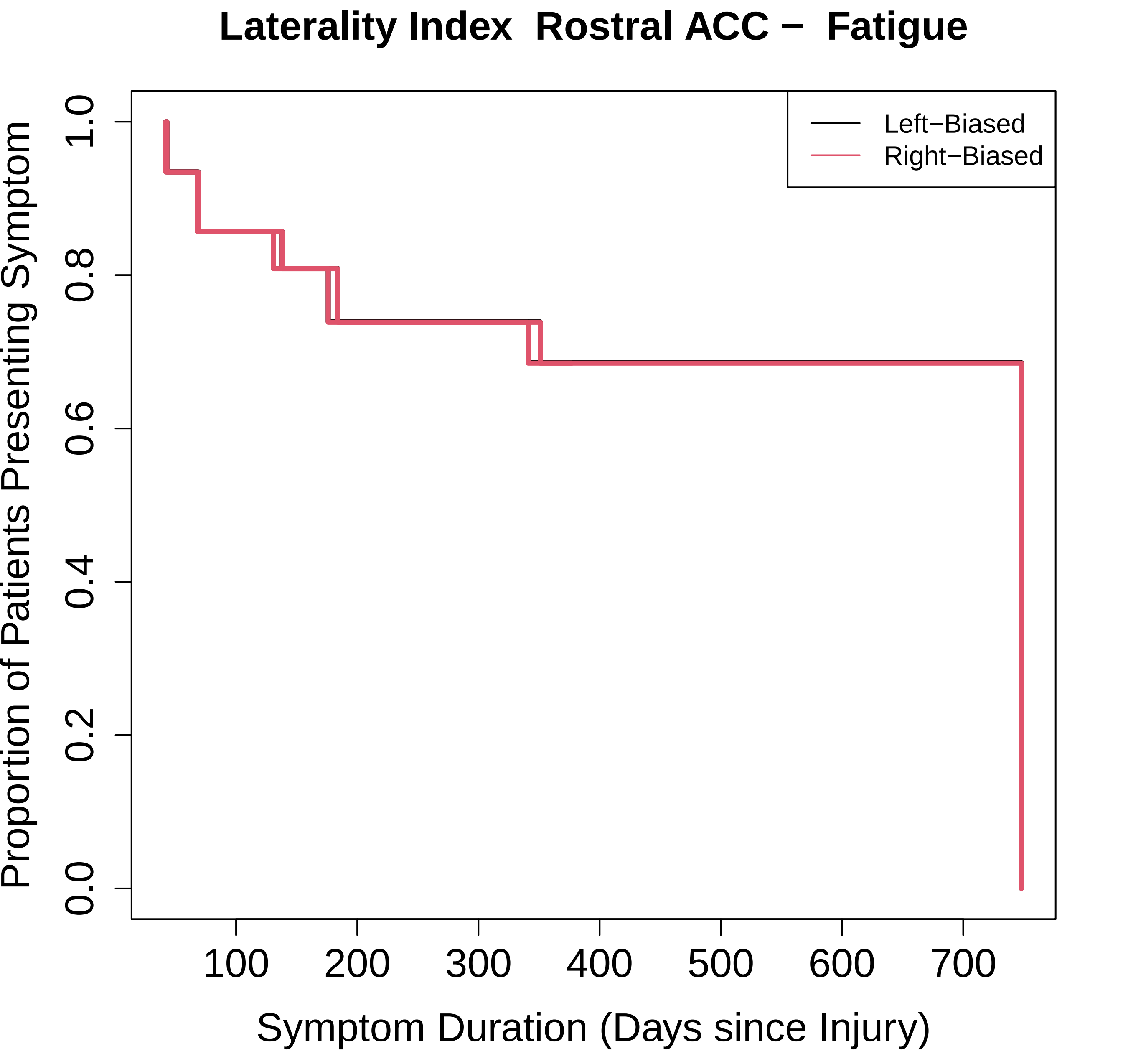
Laterality Index Rostral ACC − Fatigue
Left−Biased
Right−Biased
Proportion of Patients Presenting Symptom
0.0
0.2
0.4
0.6
0.8
1.0
100
200
300
400
500
600
700
Symptom Duration (Days since Injury)

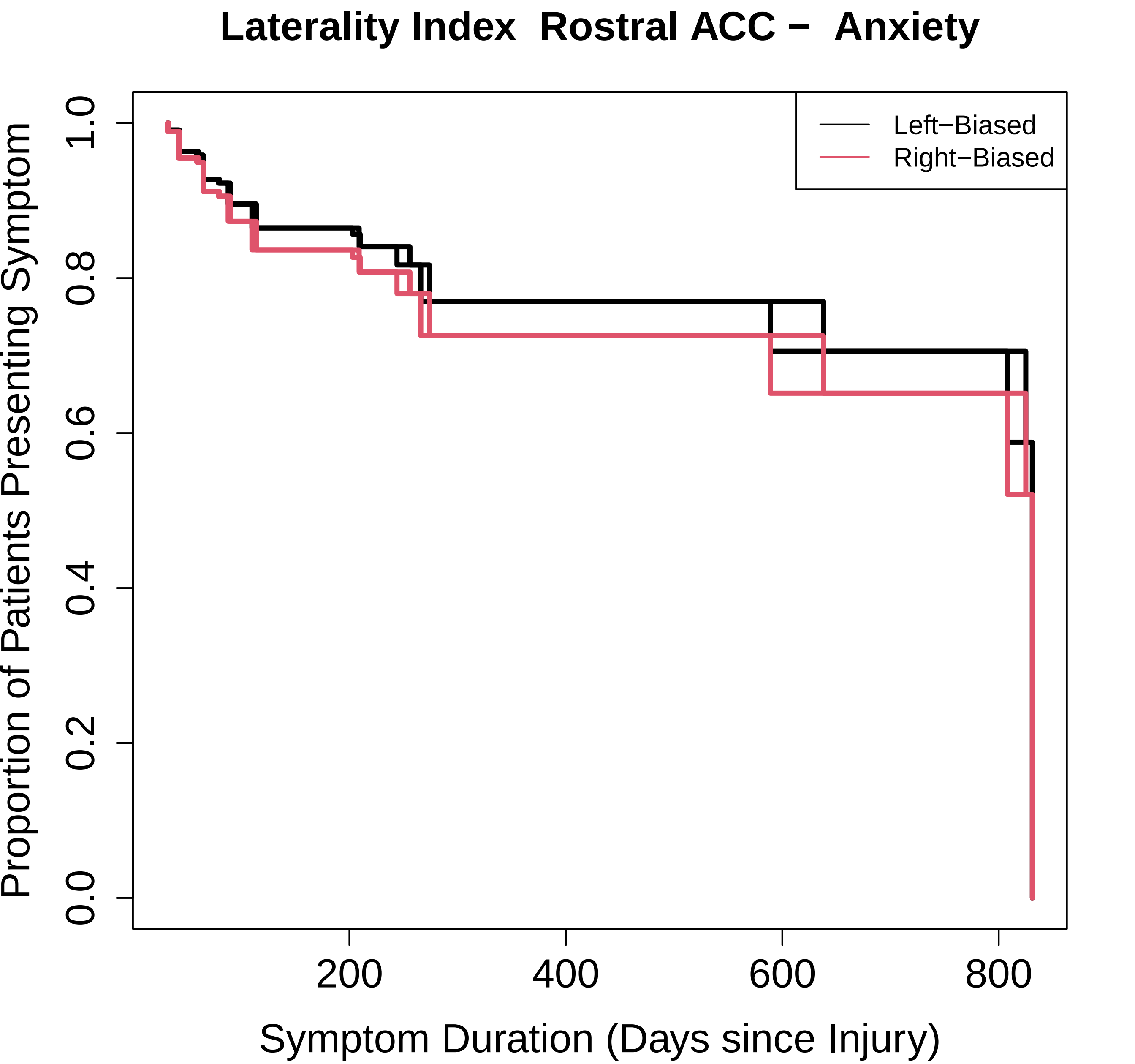

Laterality Index Rostral ACC − Anxiety
Left−Biased
Right−Biased
Proportion of Patients Presenting Symptom
0.0
0.2
0.4
0.6
0.8
1.0
200
400
600
800
Symptom Duration (Days since Injury)

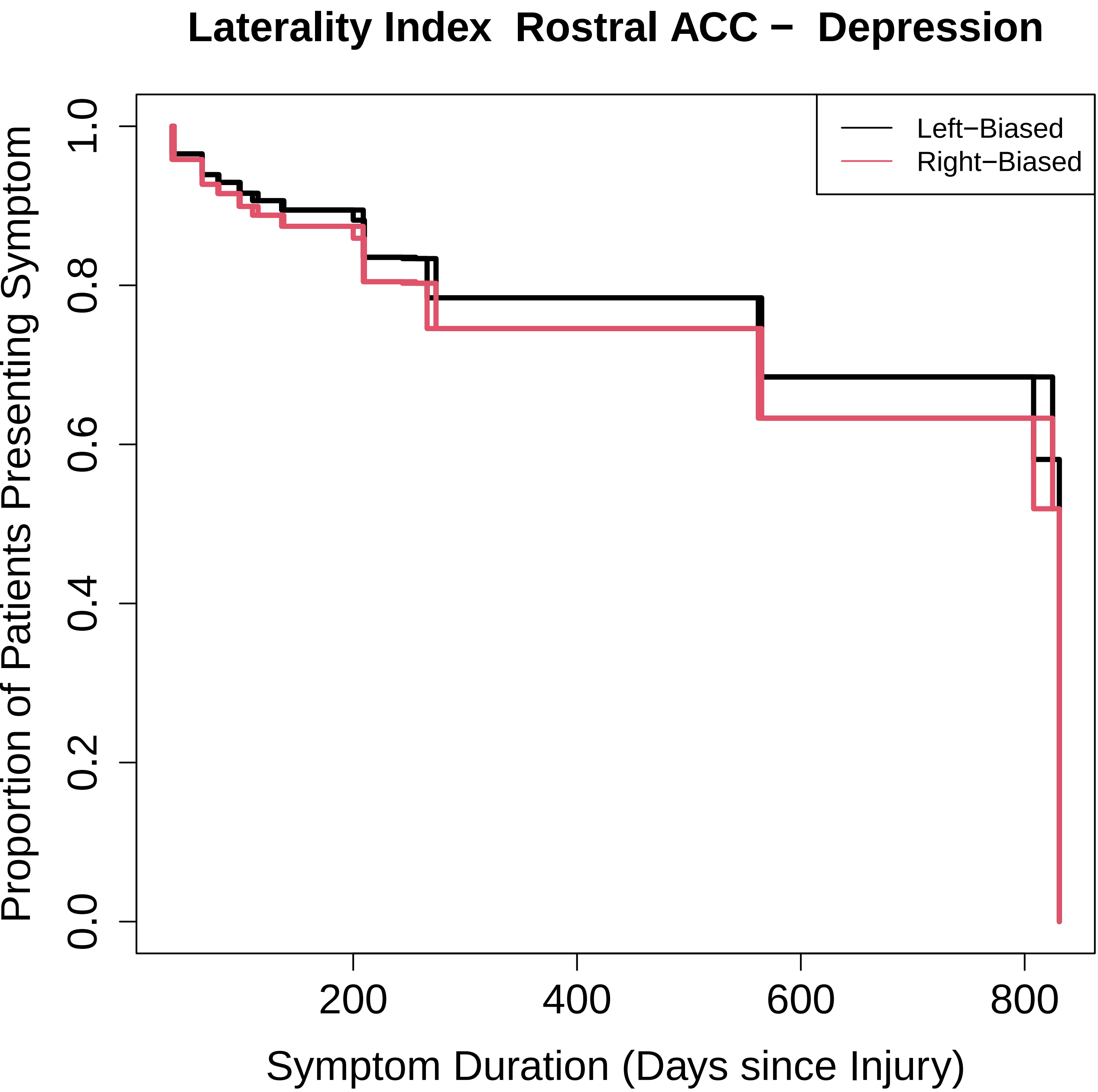

Laterality Index Rostral ACC − Depression
Left−Biased
Right−Biased
Proportion of Patients Presenting Symptom
0.0
0.2
0.4
0.6
0.8
1.0
200
400
600
800
Symptom Duration (Days since Injury)

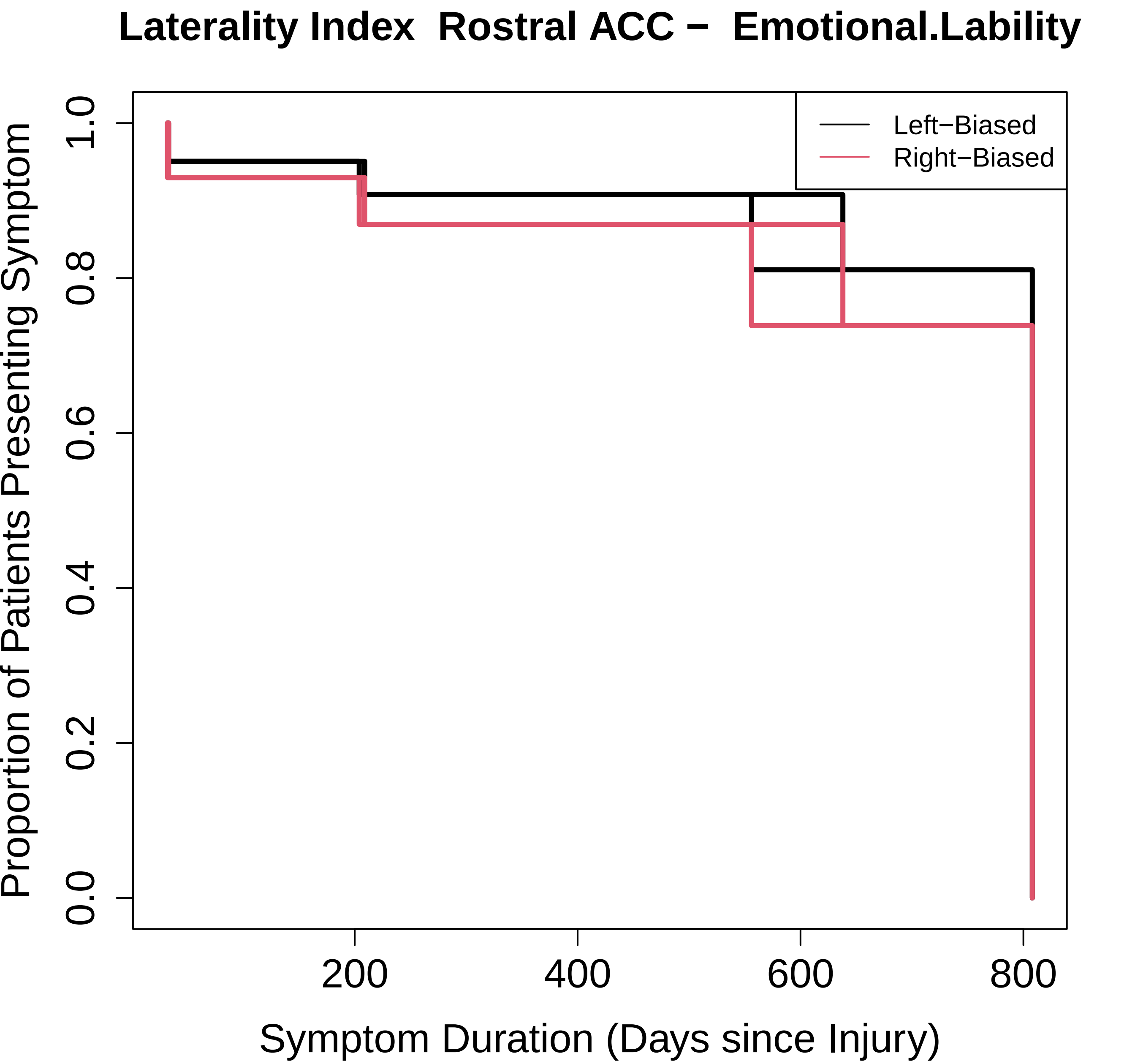

Laterality Index Rostral ACC − Emotional.Lability
Left−Biased
Right−Biased
Proportion of Patients Presenting Symptom
0.0
0.2
0.4
0.6
0.8
1.0
200
400
600
800
Symptom Duration (Days since Injury)

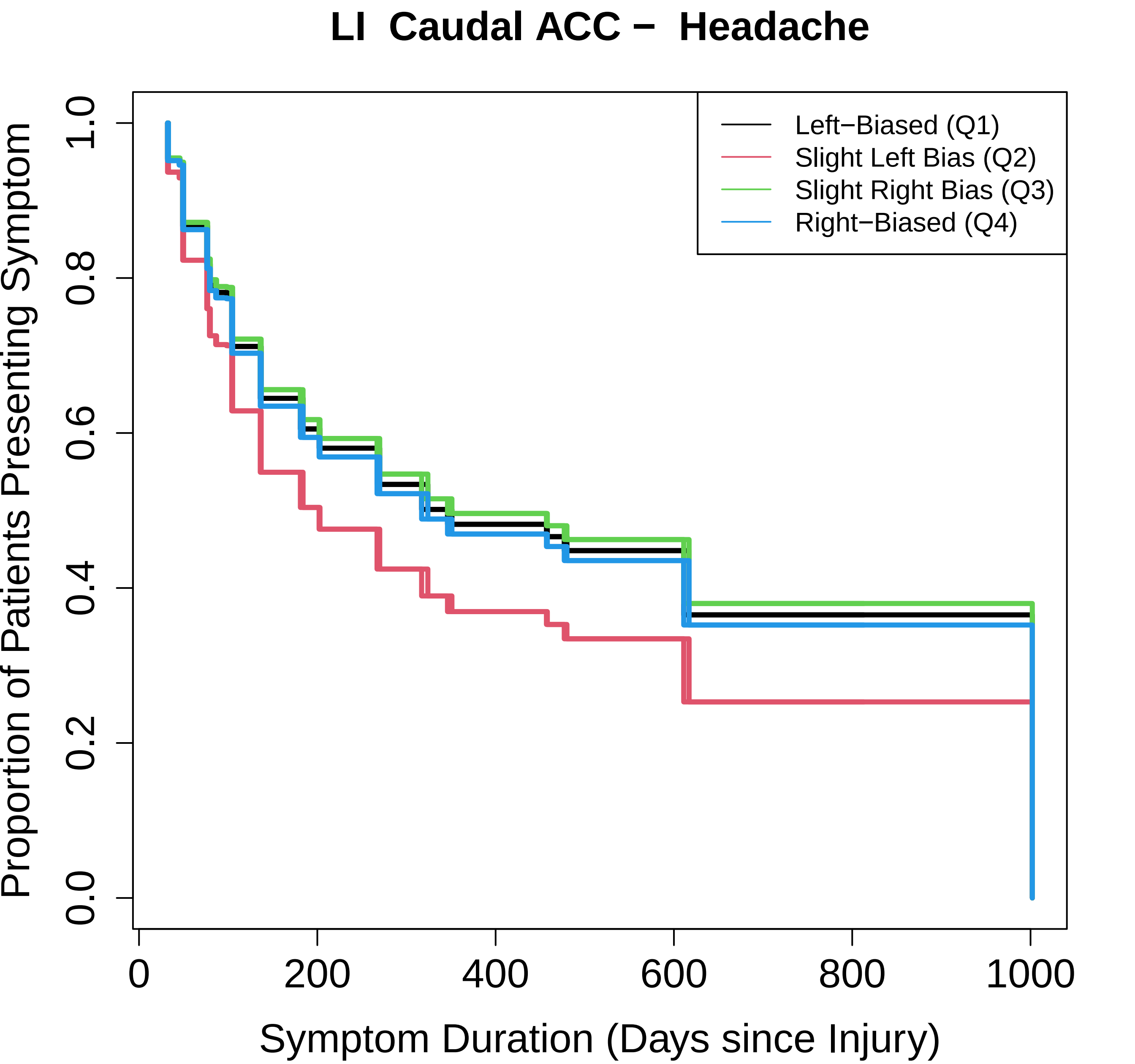
LI Caudal ACC − Headache
Proportion of Patients Presenting Symptom
Symptom Duration (Days since Injury)
Left−Biased (Q1)
Slight Left Bias (Q2)
Slight Right Bias (Q3)
Right−Biased (Q4)
0.0
0.2
0.4
0.6
0.8
1.0
0
200
400
600
800
1000

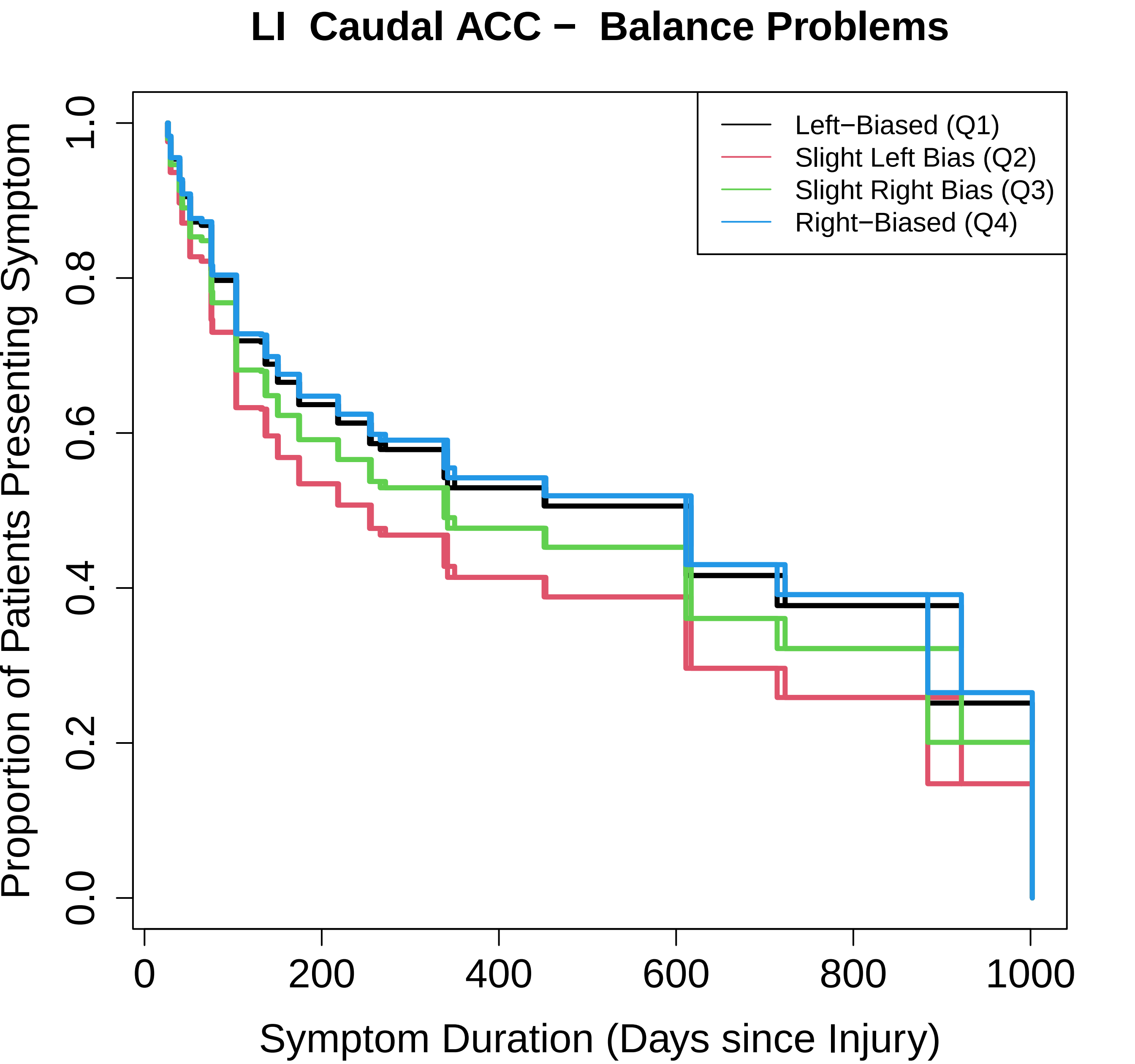

LI Caudal ACC − Balance Problems
Proportion of Patients Presenting Symptom
Symptom Duration (Days since Injury)
0.0
0.2
0.4
0.6
0.8
1.0
0
200
400
600
800
1000
Left−Biased (Q1)
Slight Left Bias (Q2)
Slight Right Bias (Q3)
Right−Biased (Q4)

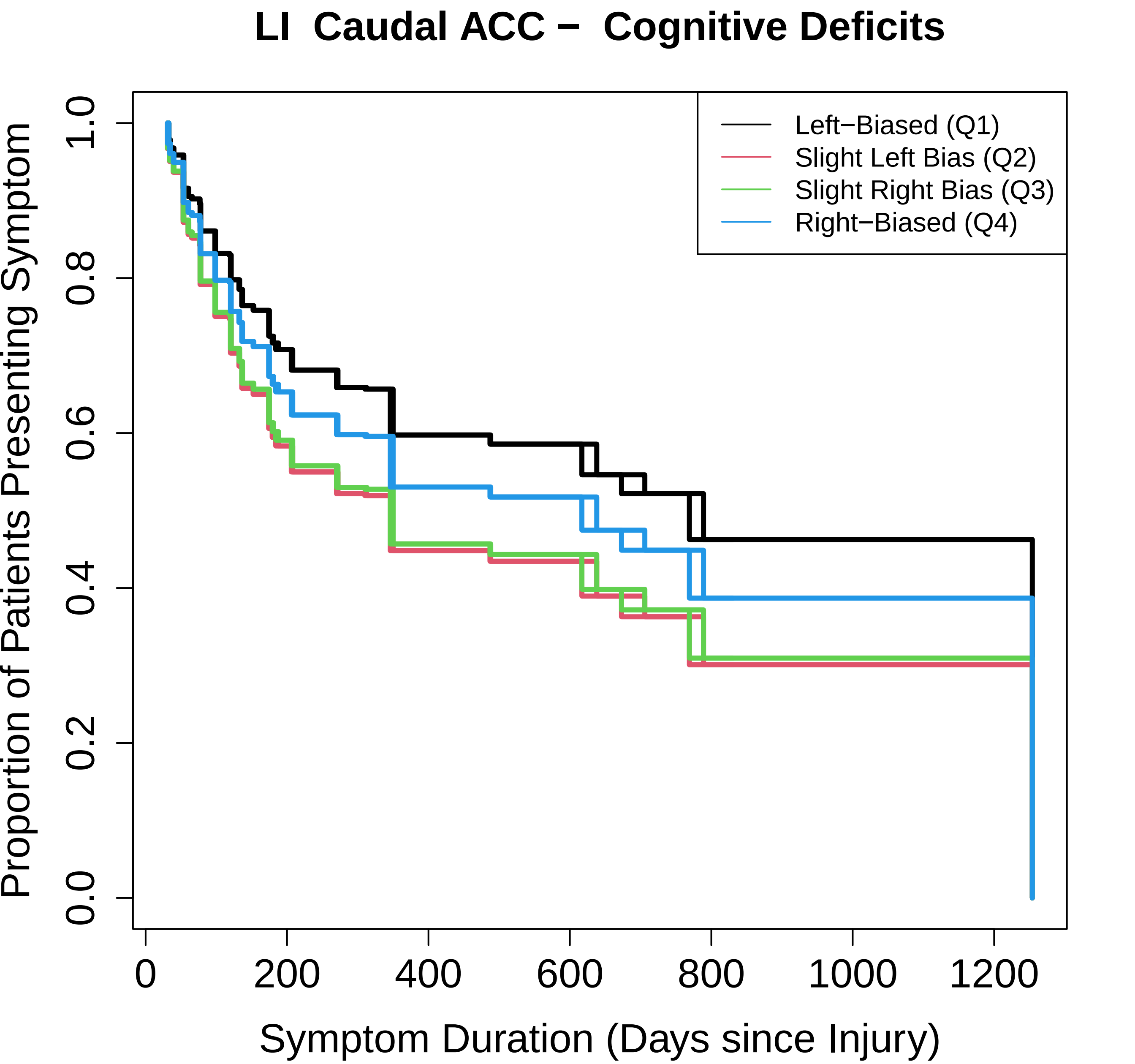

LI Caudal ACC − Cognitive Deficits
Left−Biased (Q1)
Slight Left Bias (Q2)
Slight Right Bias (Q3)
Right−Biased (Q4)
Proportion of Patients Presenting Symptom
0.0
0.2
0.4
0.6
0.8
1.0
0
200
400
600
800
1000
1200
Symptom Duration (Days since Injury)

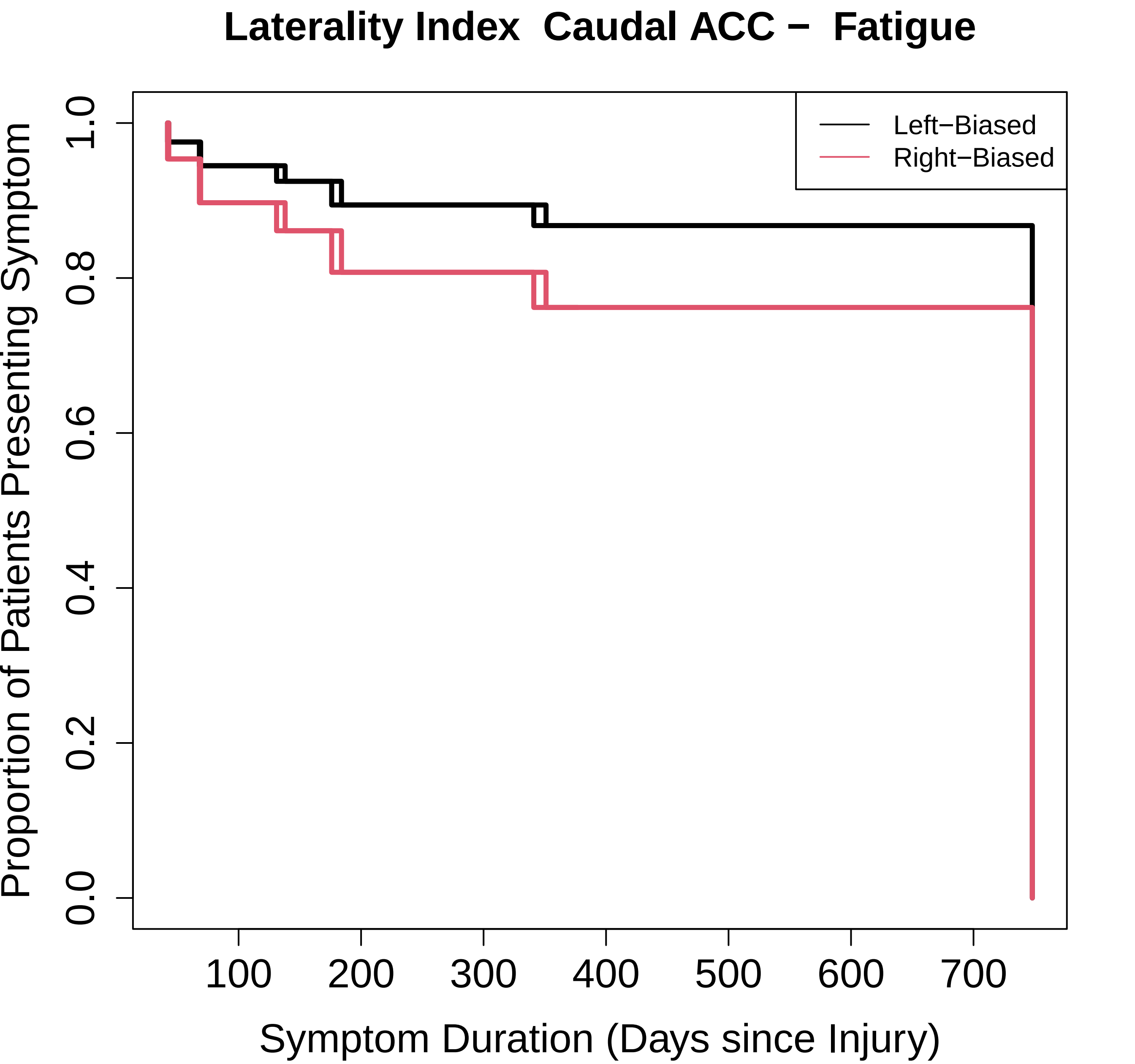

Laterality Index Caudal ACC − Fatigue
Left−Biased
Right−Biased
Proportion of Patients Presenting Symptom
0.0
0.2
0.4
0.6
0.8
1.0
100
200
300
400
500
600
700
Symptom Duration (Days since Injury)

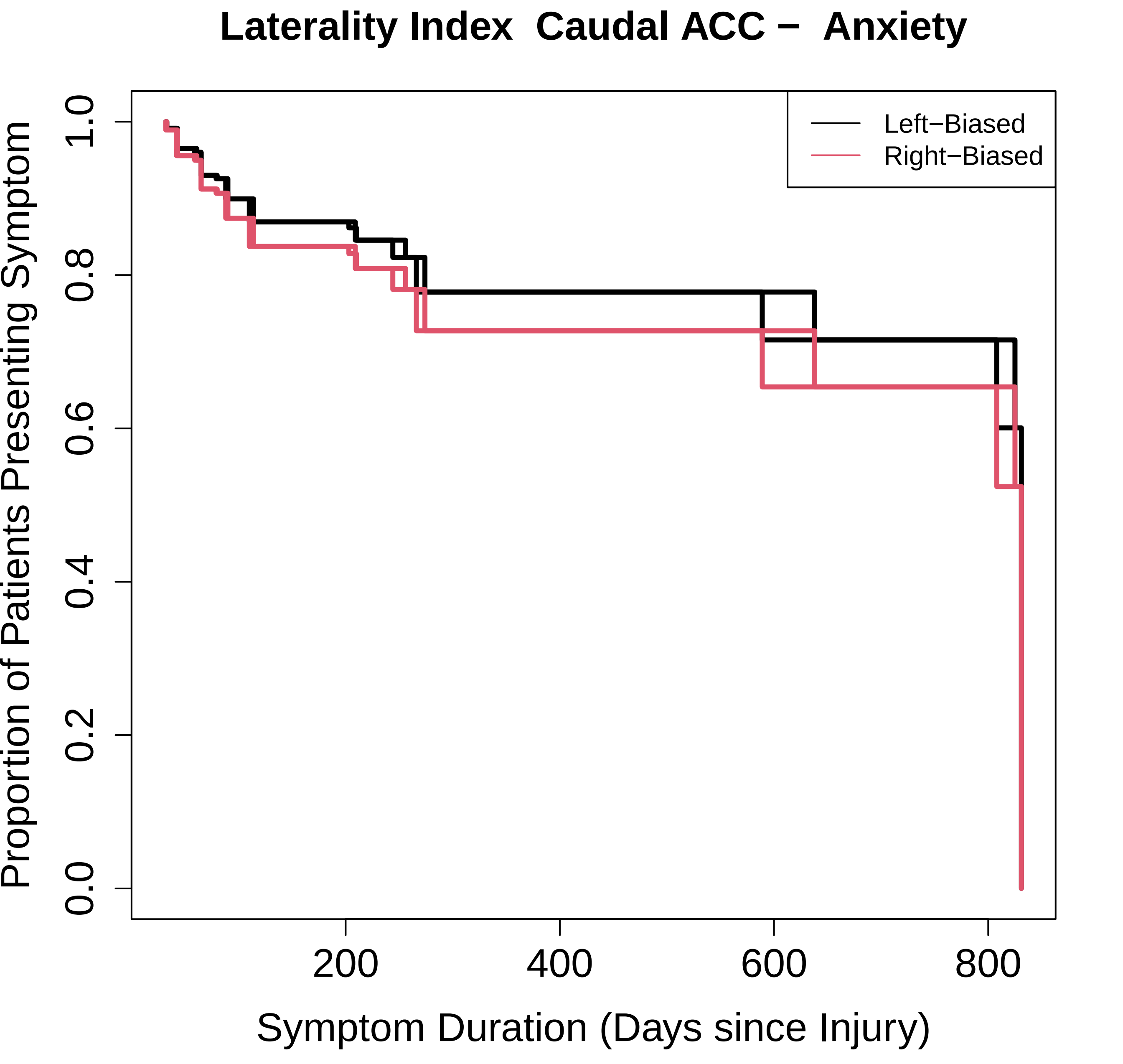

Laterality Index Caudal ACC − Anxiety
Left−Biased
Right−Biased
0.0
0.2
0.4
0.6
0.8
1.0
Proportion of Patients Presenting Symptom
200
400
600
800
Symptom Duration (Days since Injury)

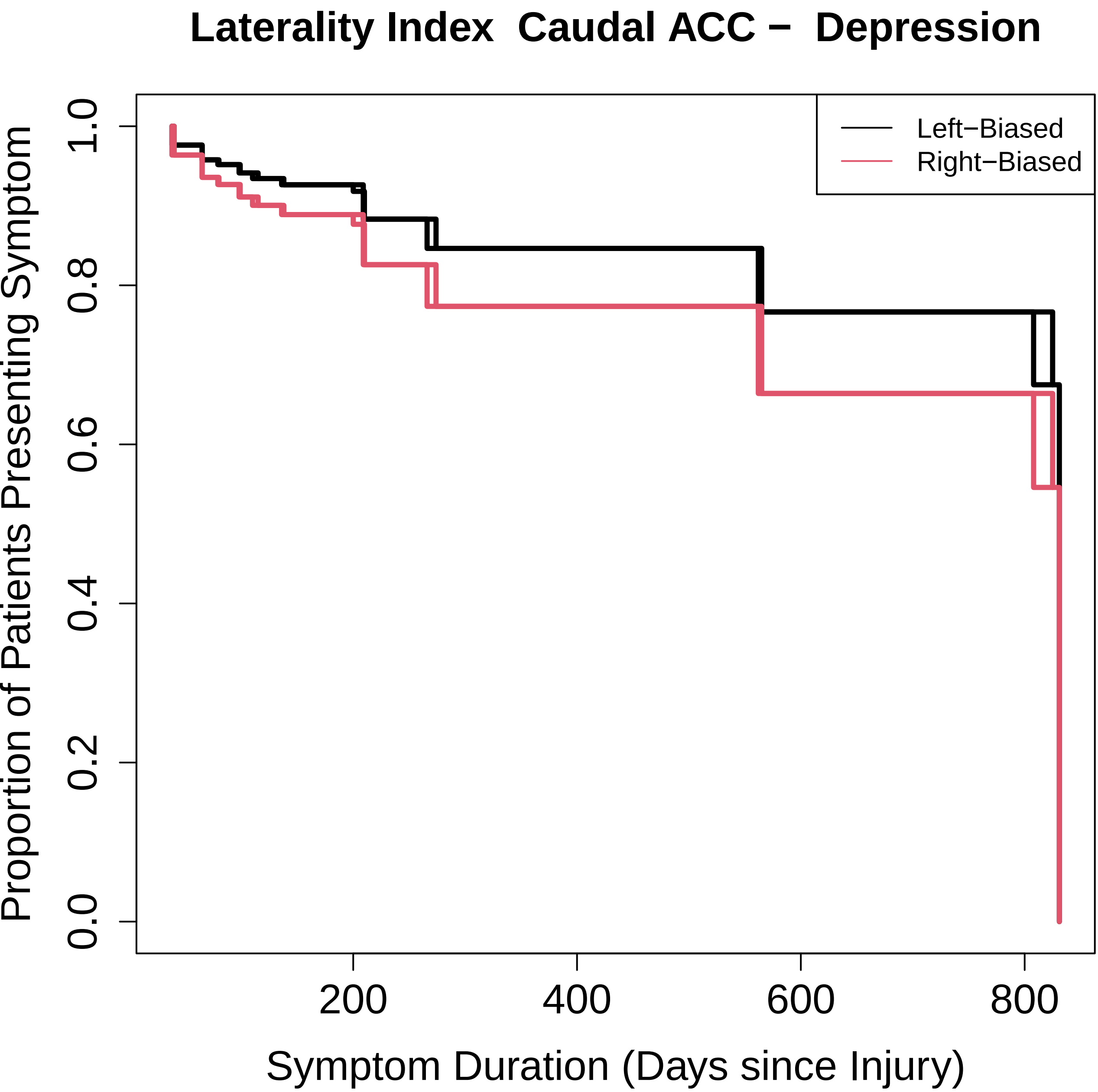

Laterality Index Caudal ACC − Depression
Left−Biased
Right−Biased
Proportion of Patients Presenting Symptom
0.0
0.2
0.4
0.6
0.8
1.0
200
400
600
800
Symptom Duration (Days since Injury)

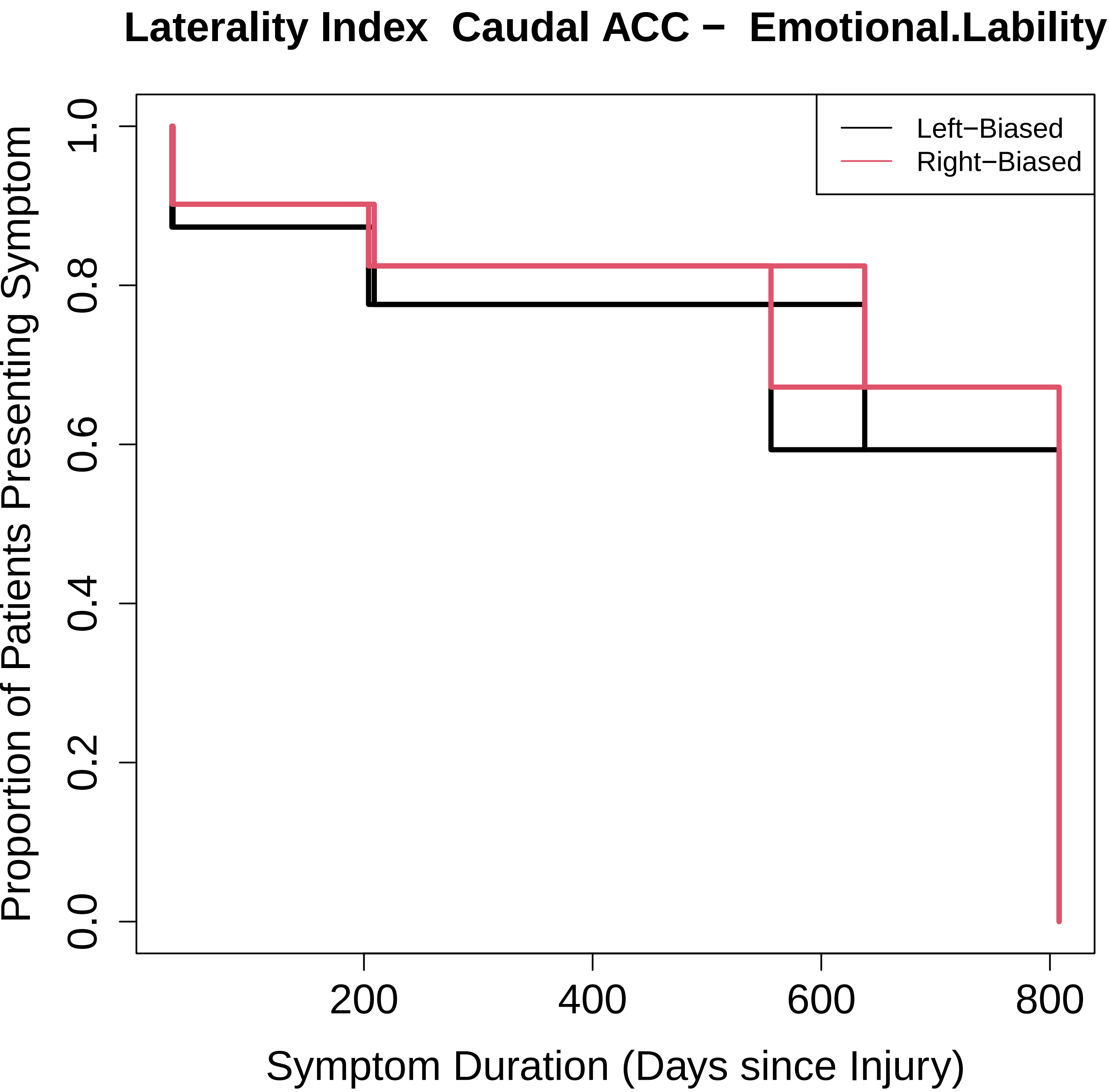

Laterality Index Caudal ACC − Emotional.Lability
Left−Biased
Right−Biased
1.0
0.8
0.6
0.4
0.2
0.0
Proportion of Patients Presenting Symptom
200
400
600
800
Symptom Duration (Days since Injury)

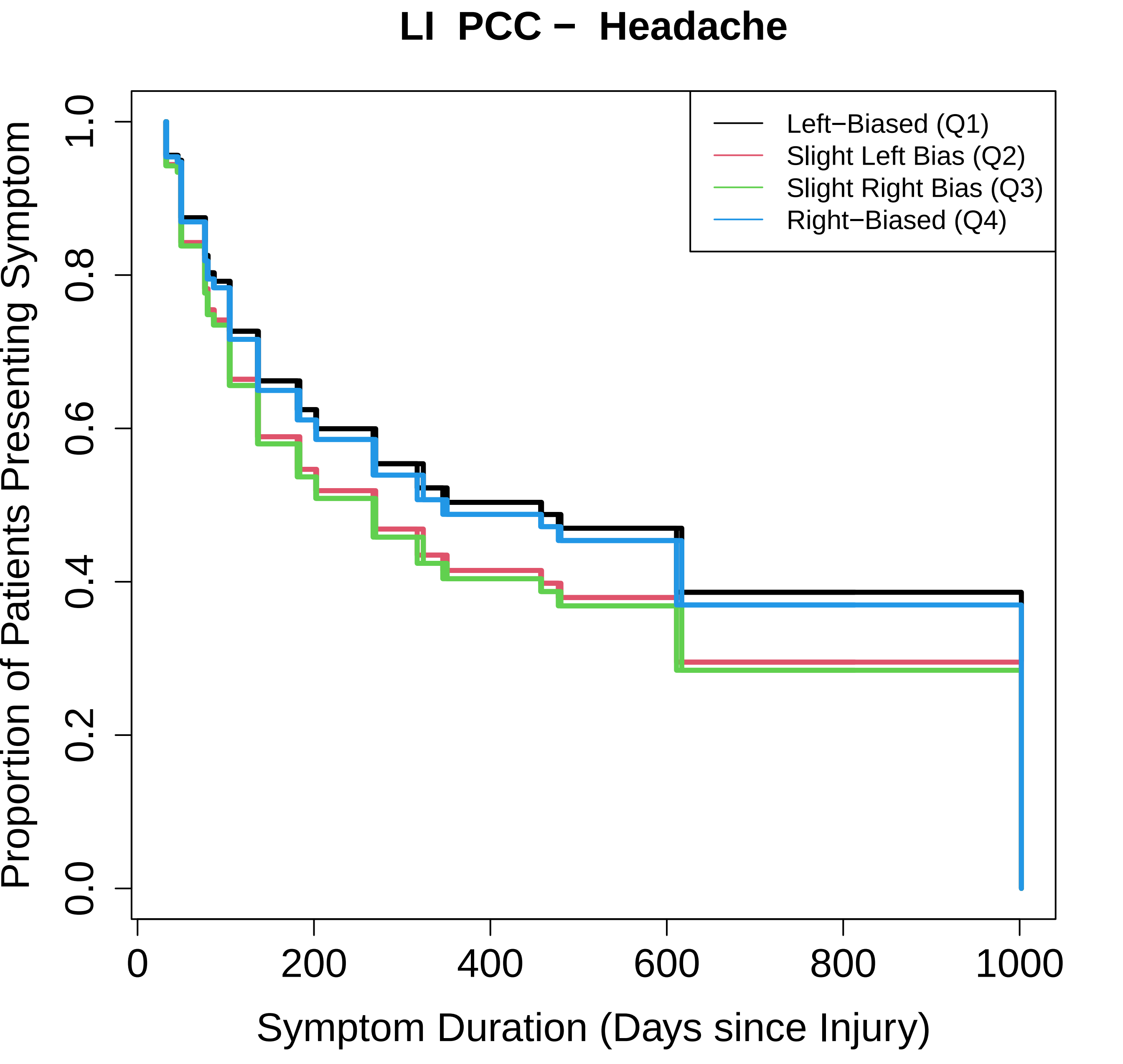
LI PCC − Headache
Left−Biased (Q1)
Slight Left Bias (Q2)
Slight Right Bias (Q3)
Right−Biased (Q4)
Proportion of Patients Presenting Symptom
0.0
0.2
0.4
0.6
0.8
1.0
0
200
400
600
800
1000
Symptom Duration (Days since Injury)

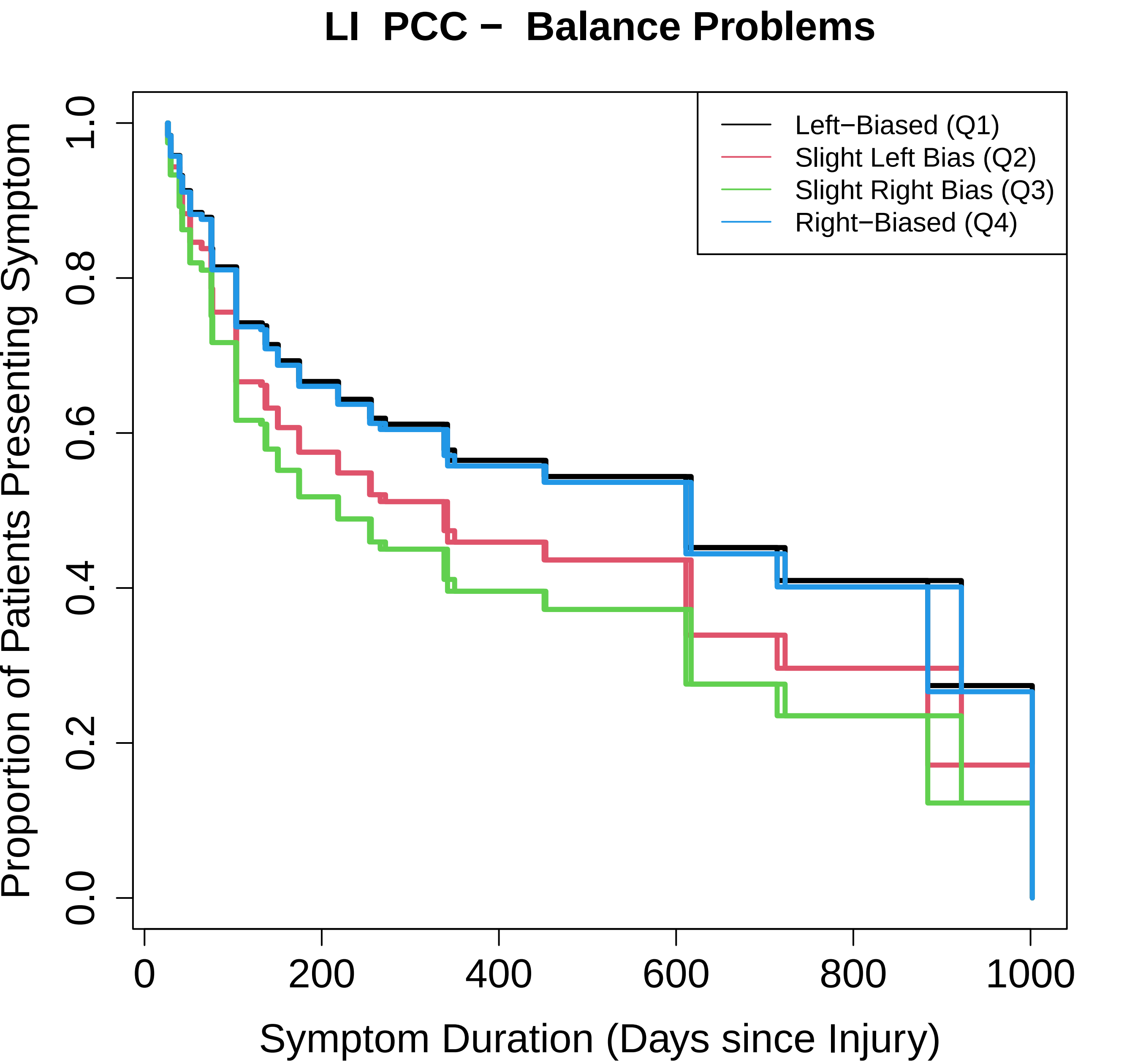

LI PCC – Balance Problems
Proportion of Patients Presenting Symptom
0.0
0.2
0.4
0.6
0.8
1.0
0
200
400
600
800
1000
Symptom Duration (Days since Injury)
Left−Biased (Q1)
Slight Left Bias (Q2)
Slight Right Bias (Q3)
Right−Biased (Q4)

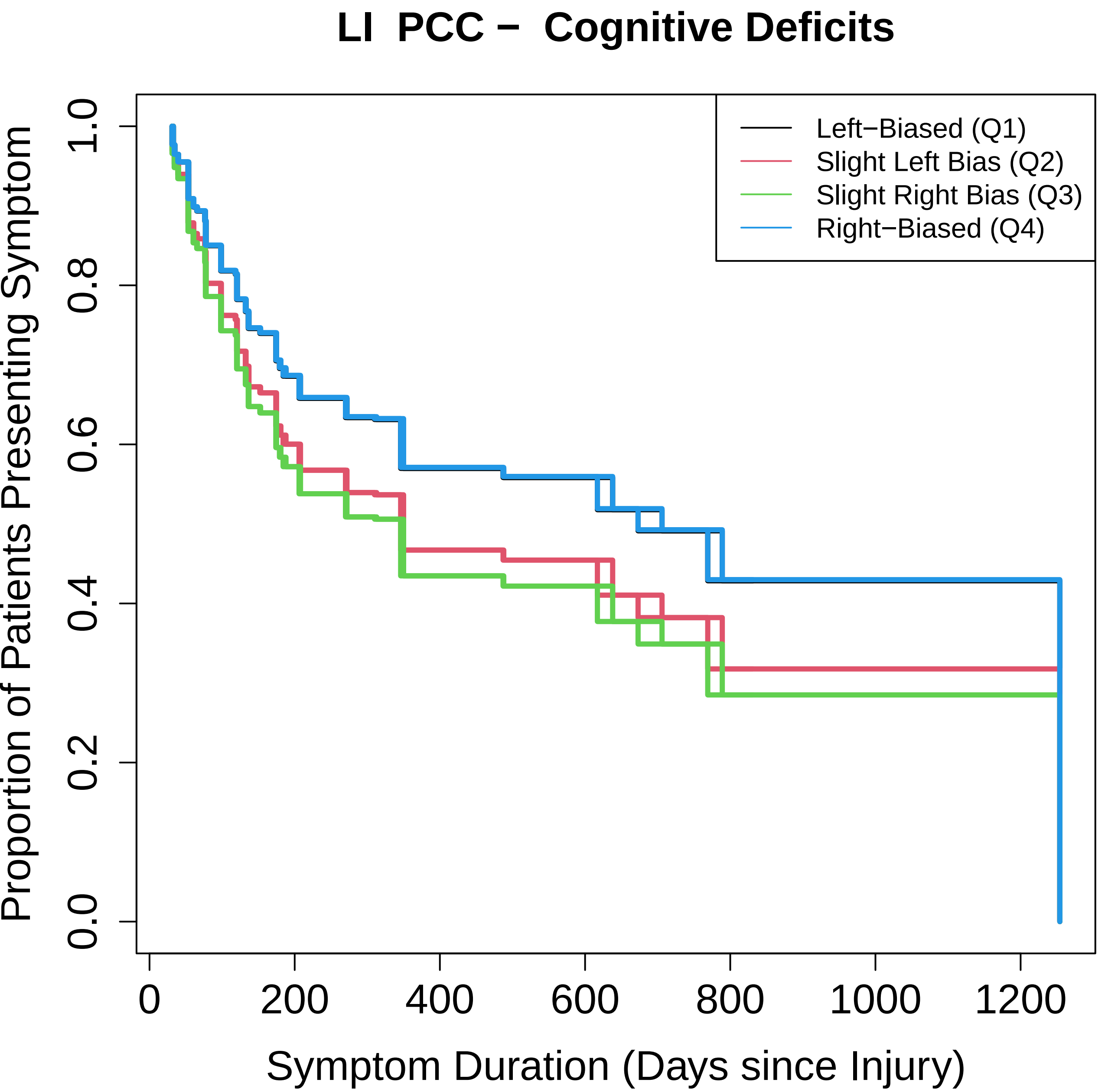
LI PCC − Cognitive Deficits
Left−Biased (Q1)
Slight Left Bias (Q2)
Slight Right Bias (Q3)
Right−Biased (Q4)
Proportion of Patients Presenting Symptom
0.0
0.2
0.4
0.6
0.8
1.0
0
200
400
600
800
1000
1200
Symptom Duration (Days since Injury)

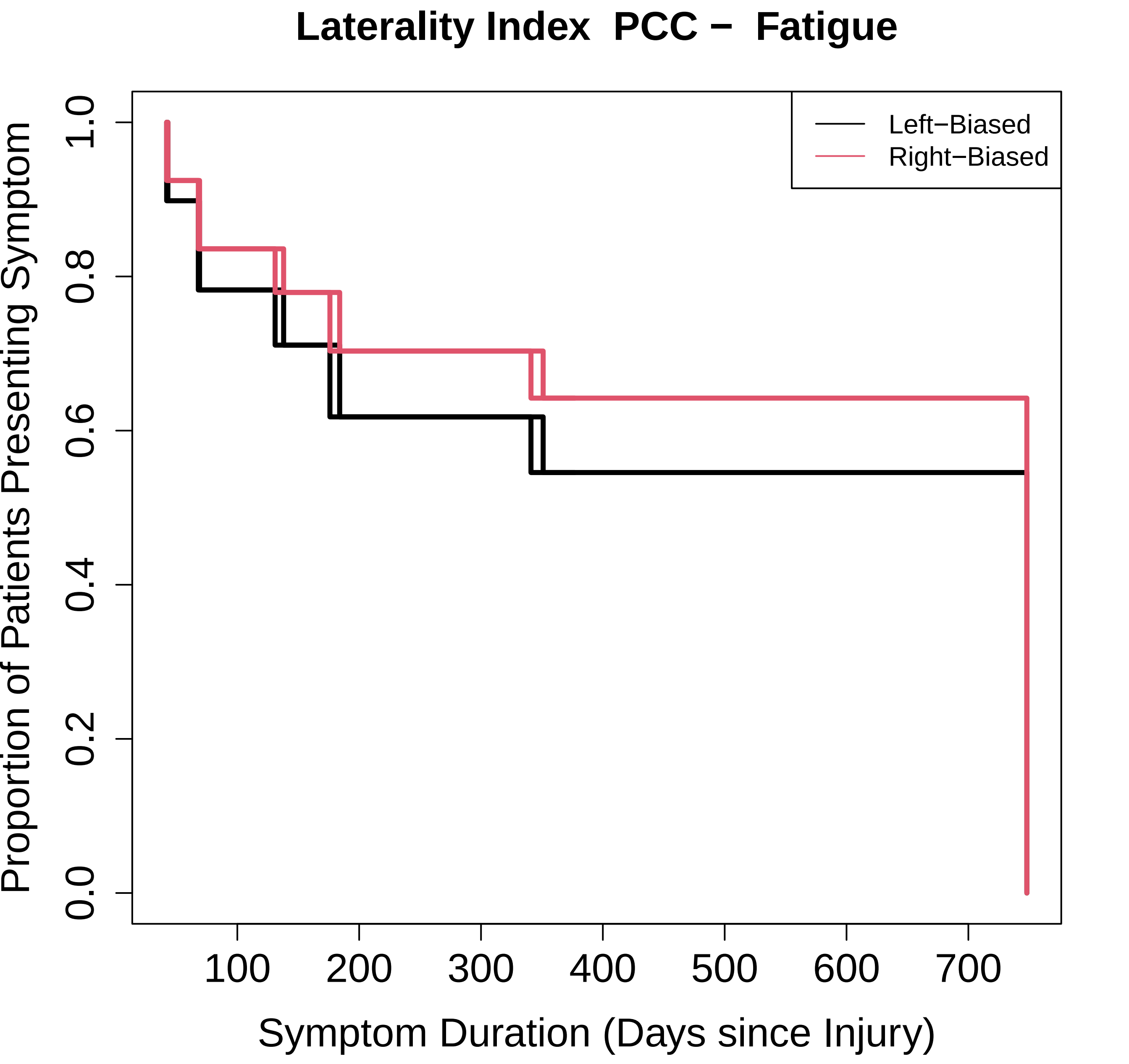

Laterality Index PCC − Fatigue
Proportion of Patients Presenting Symptom
Symptom Duration (Days since Injury)
0.0
0.2
0.4
0.6
0.8
1.0
100
200
300
400
500
600
700
Left−Biased
Right−Biased

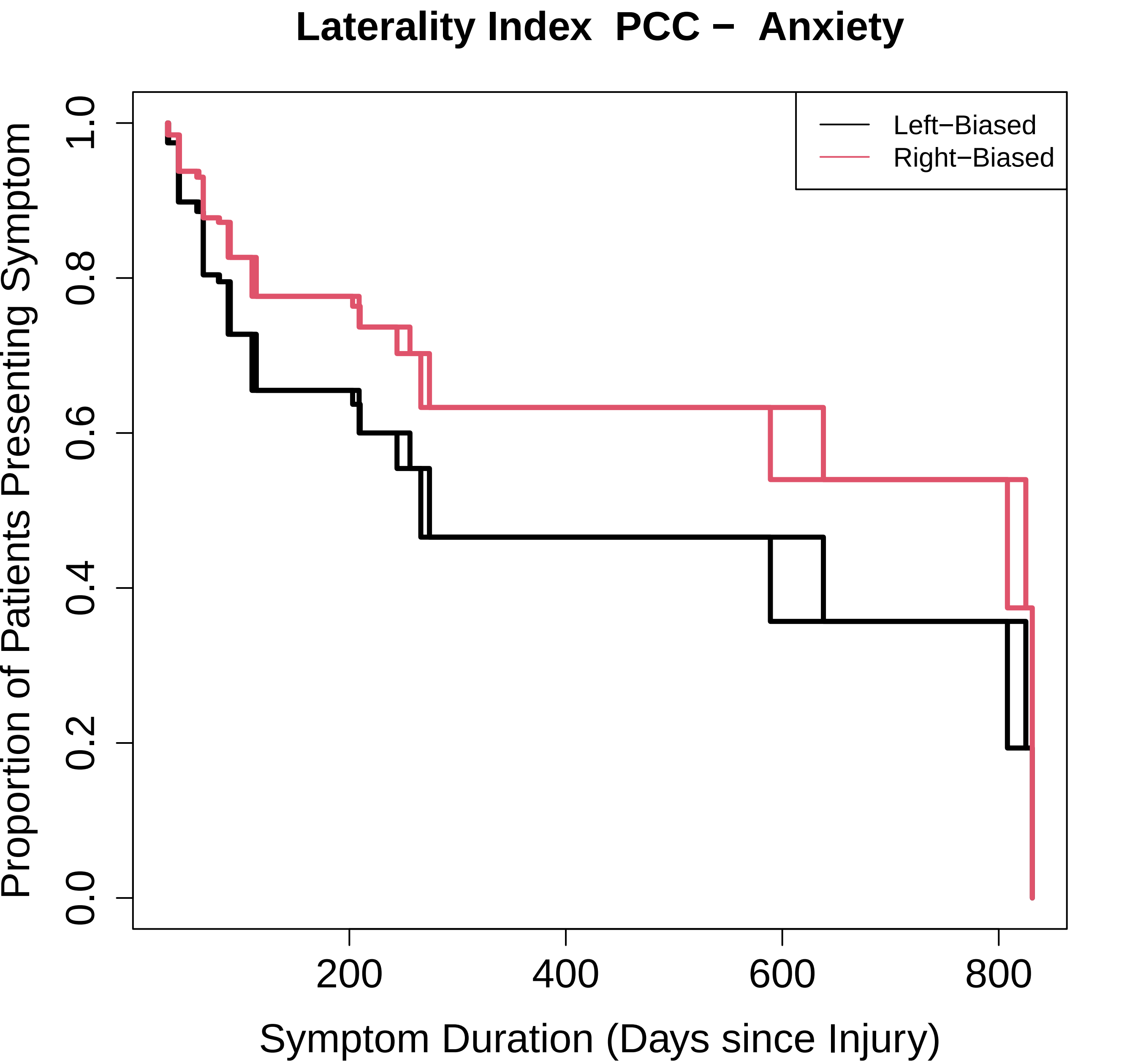

Laterality Index PCC − Anxiety
Left−Biased
Right−Biased
Proportion of Patients Presenting Symptom
0.0
0.2
0.4
0.6
0.8
1.0
200
400
600
800
Symptom Duration (Days since Injury)

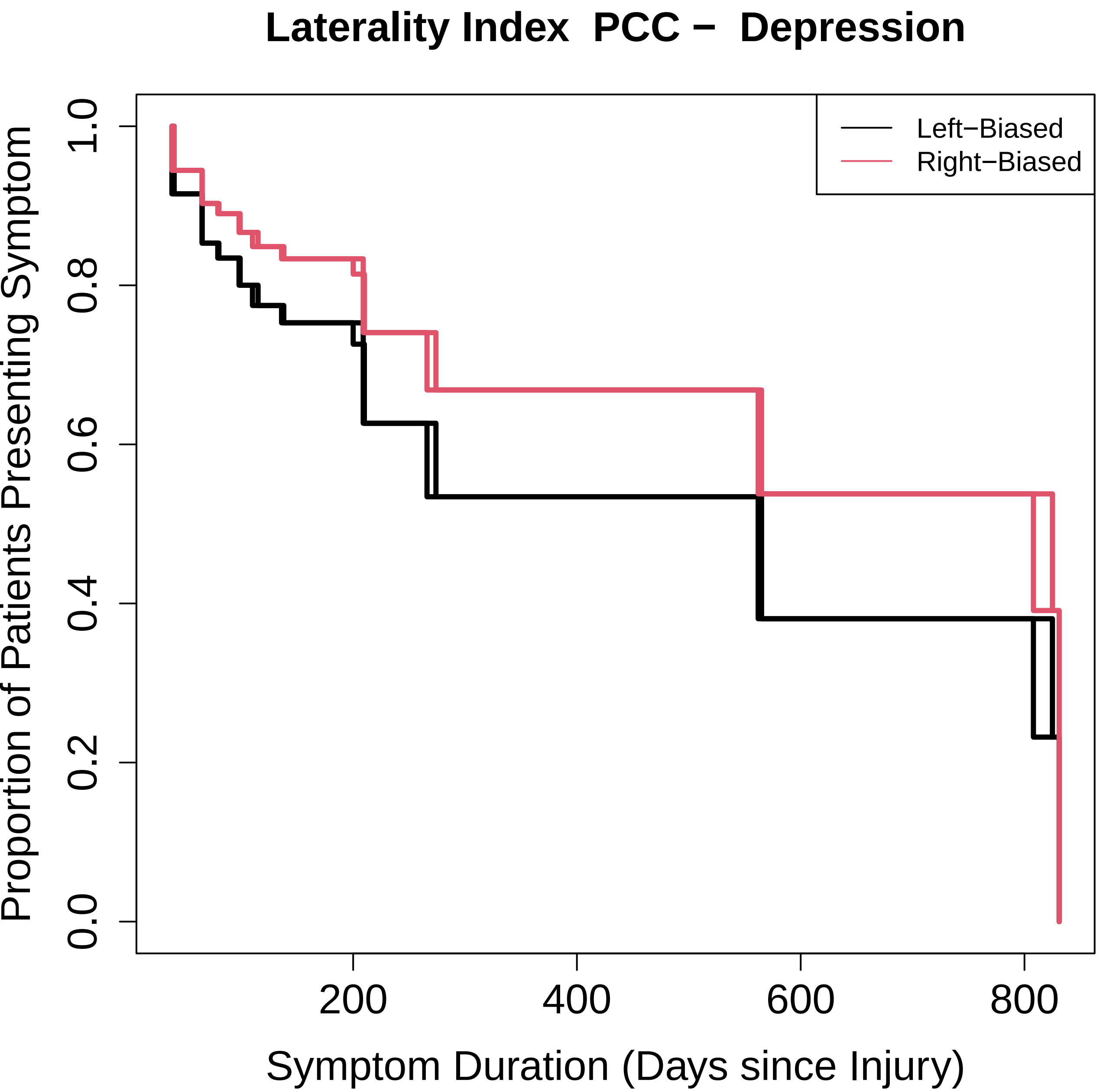
Laterality Index PCC − Depression
Left−Biased
Right−Biased
Proportion of Patients Presenting Symptom
0.0
0.2
0.4
0.6
0.8
1.0
200
400
600
800
Symptom Duration (Days since Injury)

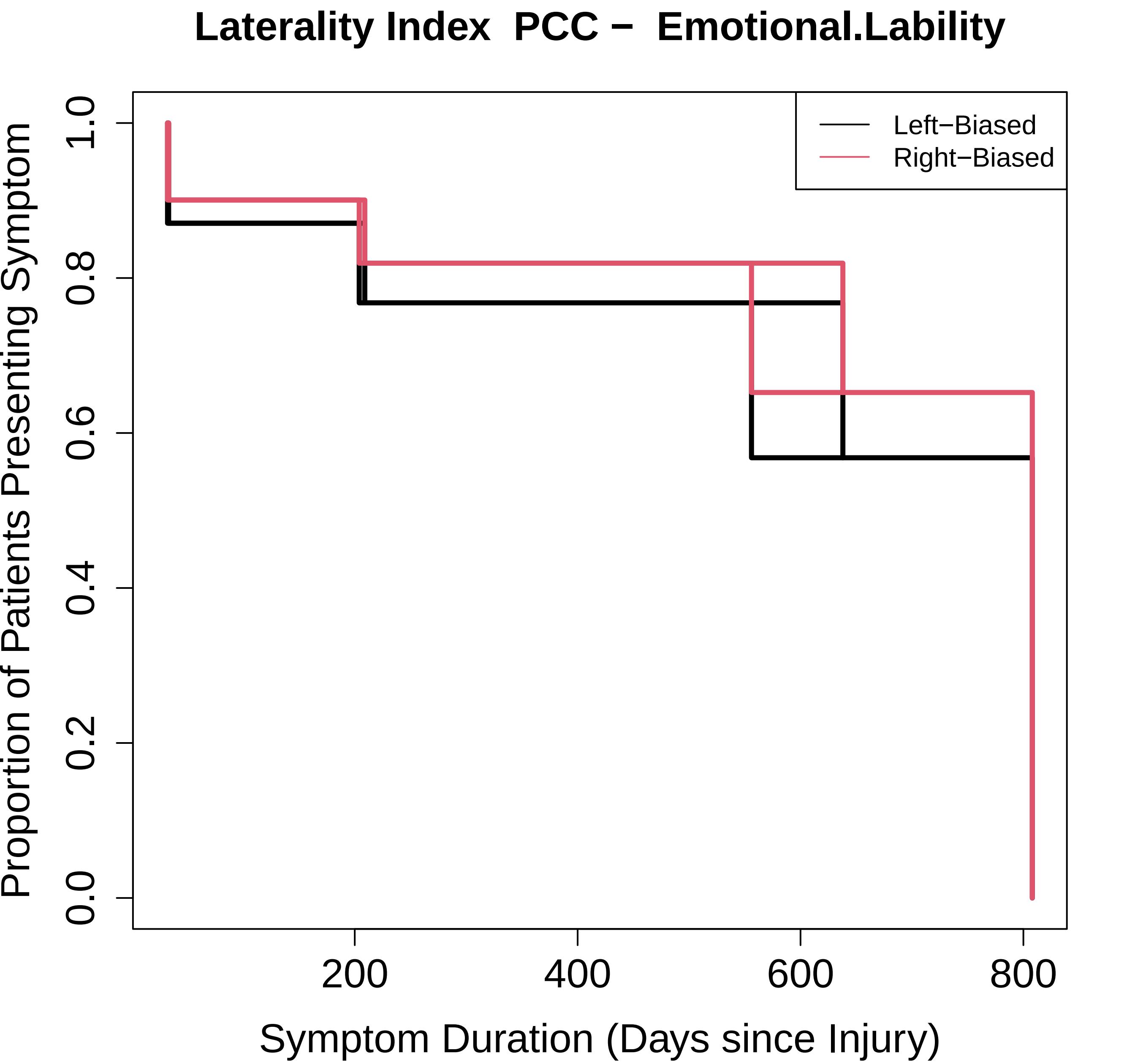

Laterality Index PCC − Emotional.Lability
Left−Biased
Right−Biased
Proportion of Patients Presenting Symptom
0.0
0.2
0.4
0.6
0.8
1.0
200
400
600
800
Symptom Duration (Days since Injury)

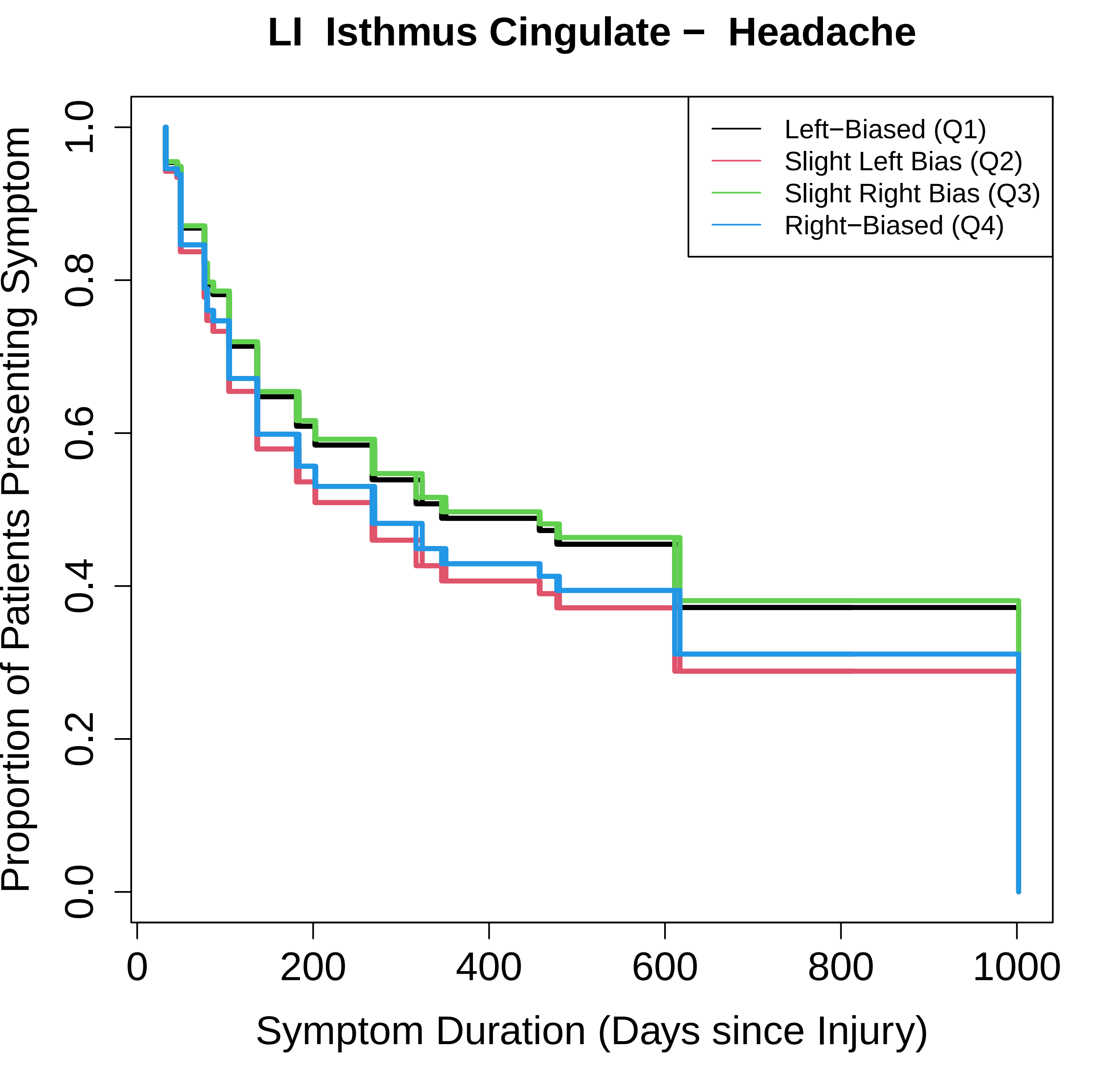

LI Isthmus Cingulate − Headache
Left−Biased (Q1)
Slight Left Bias (Q2)
Slight Right Bias (Q3)
Right−Biased (Q4)
Proportion of Patients Presenting Symptom
0.0
0.2
0.4
0.6
0.8
1.0
0
200
400
600
800
1000
Symptom Duration (Days since Injury)

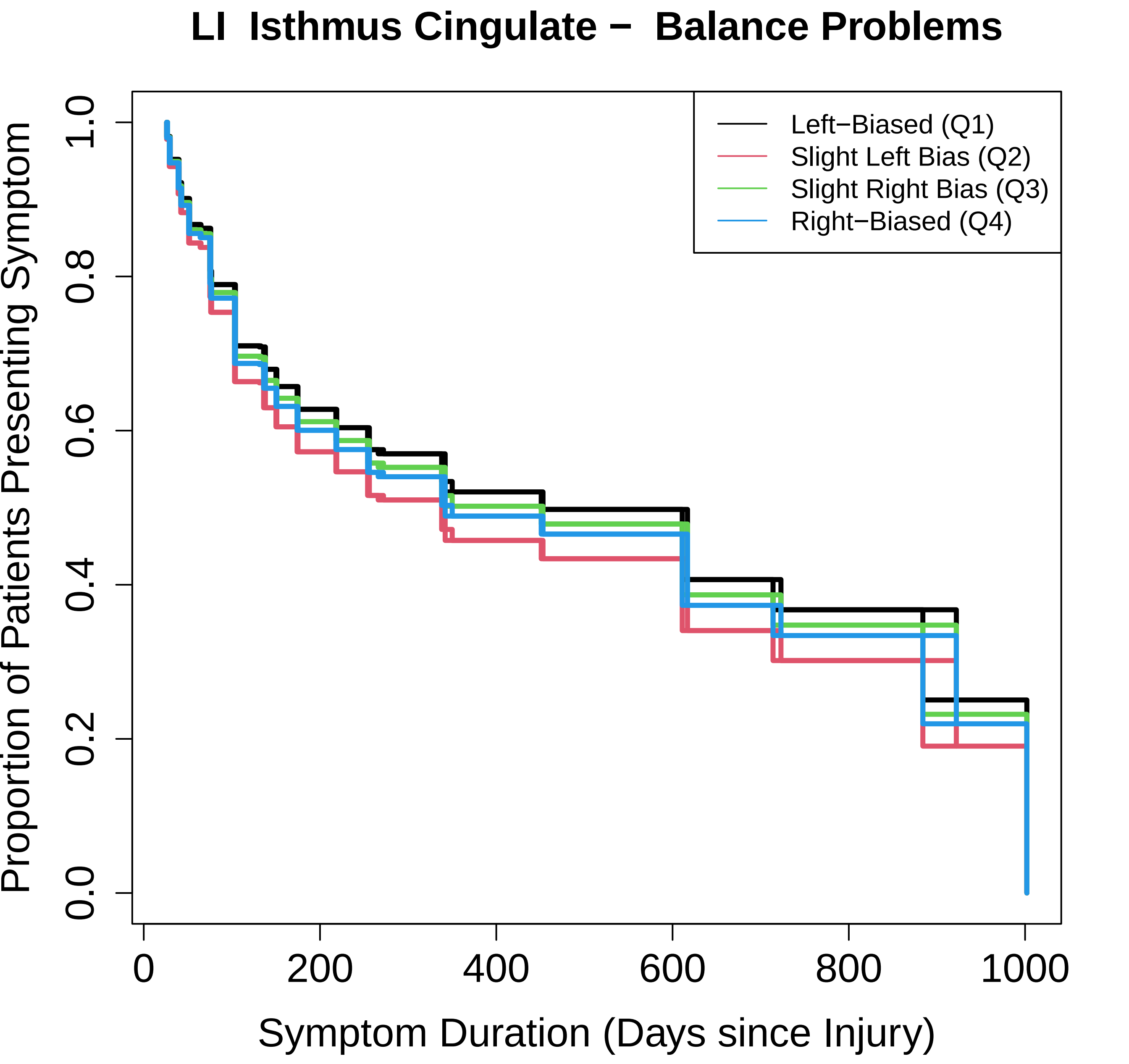
LI Isthmus Cingulate − Balance Problems
Left−Biased (Q1)
Slight Left Bias (Q2)
Slight Right Bias (Q3)
Right−Biased (Q4)
Proportion of Patients Presenting Symptom
0.0
0.2
0.4
0.6
0.8
1.0
0
200
400
600
800
1000
Symptom Duration (Days since Injury)

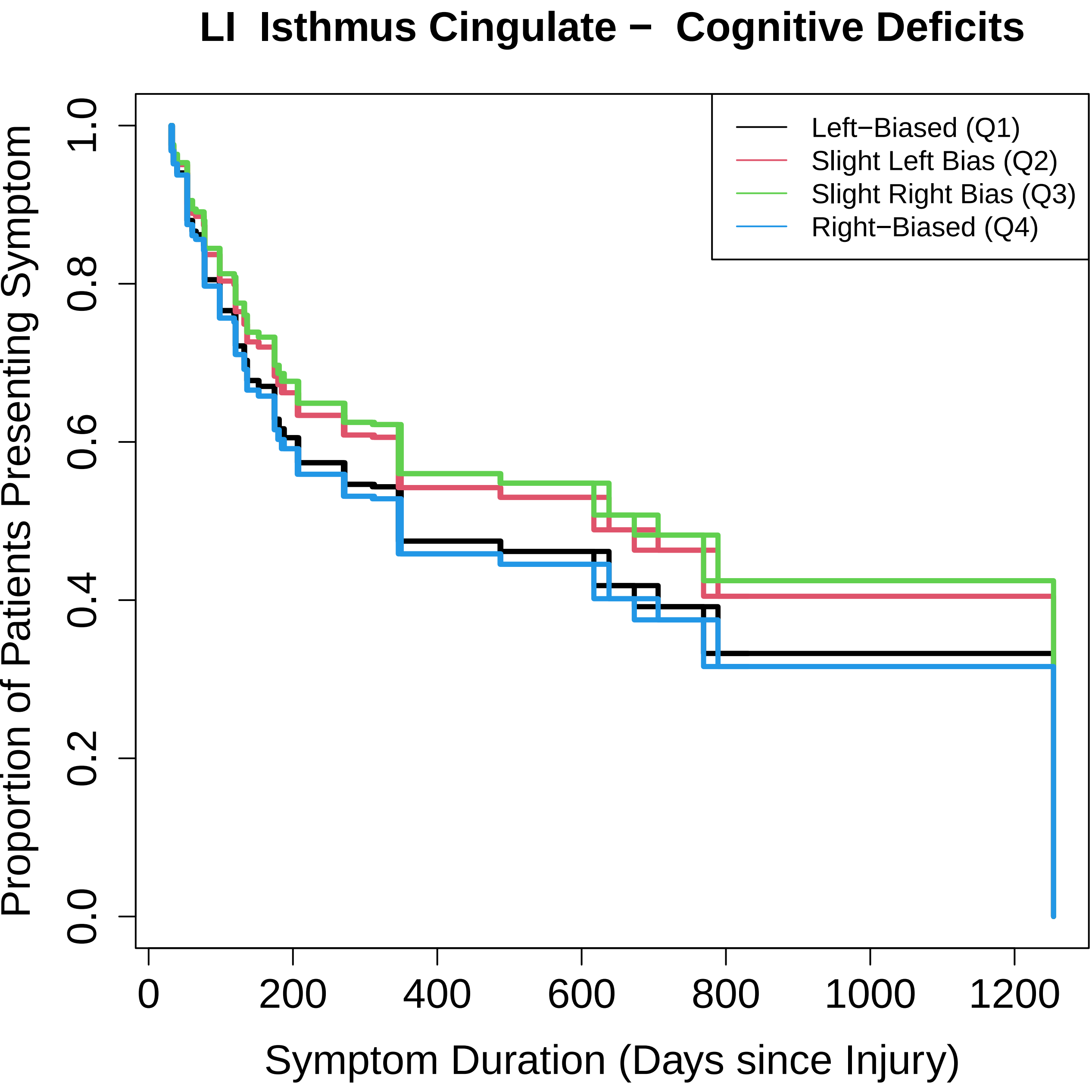

LI Isthmus Cingulate − Cognitive Deficits
Proportion of Patients Presenting Symptom
0.0
0.2
0.4
0.6
0.8
1.0
0
200
400
600
800
1000
1200
Symptom Duration (Days since Injury)
Left−Biased (Q1)
Slight Left Bias (Q2)
Slight Right Bias (Q3)
Right−Biased (Q4)

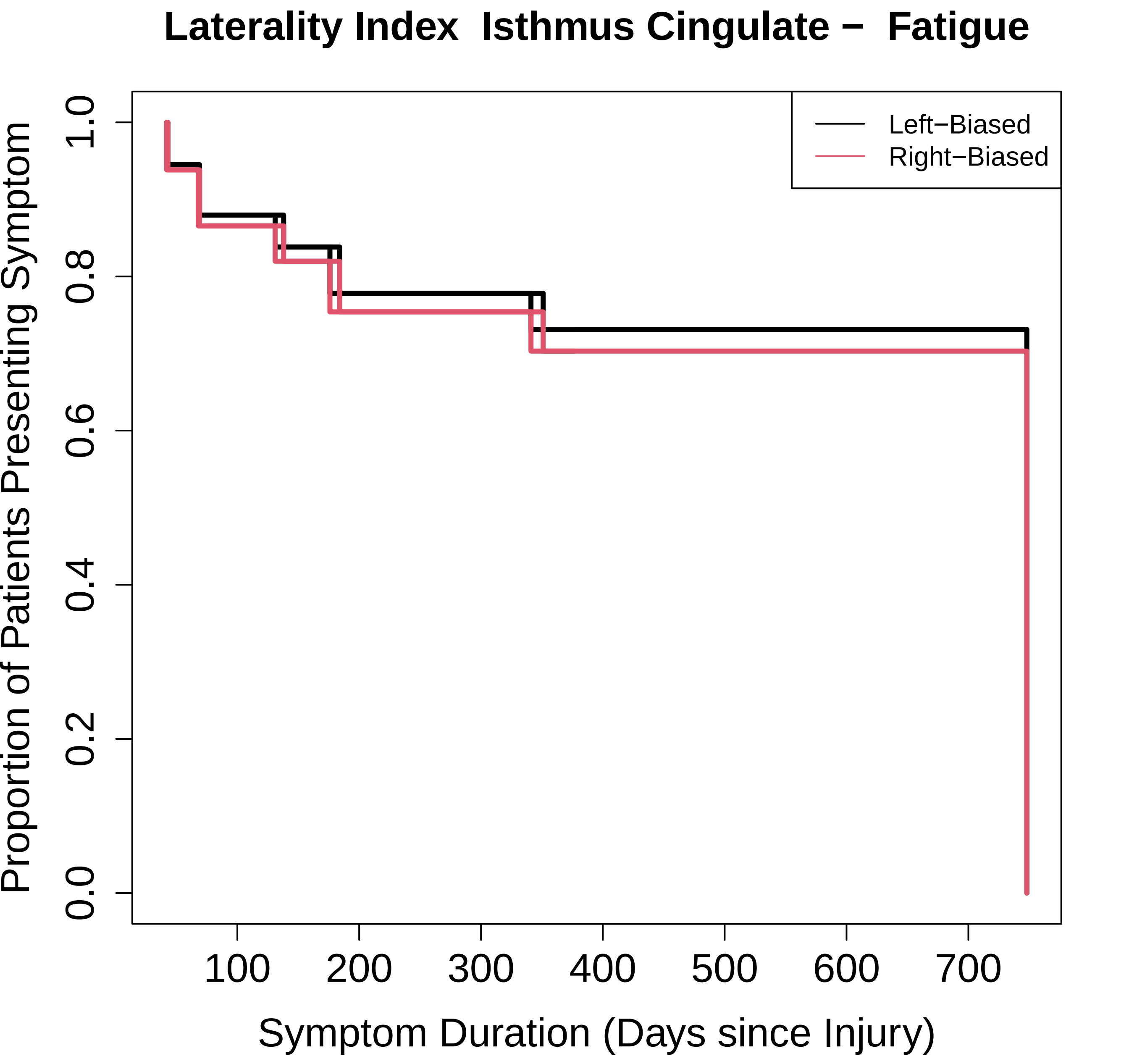

Laterality Index Isthmus Cingulate − Fatigue
Left−Biased
Right−Biased
Proportion of Patients Presenting Symptom
0.0
0.2
0.4
0.6
0.8
1.0
100
200
300
400
500
600
700
Symptom Duration (Days since Injury)

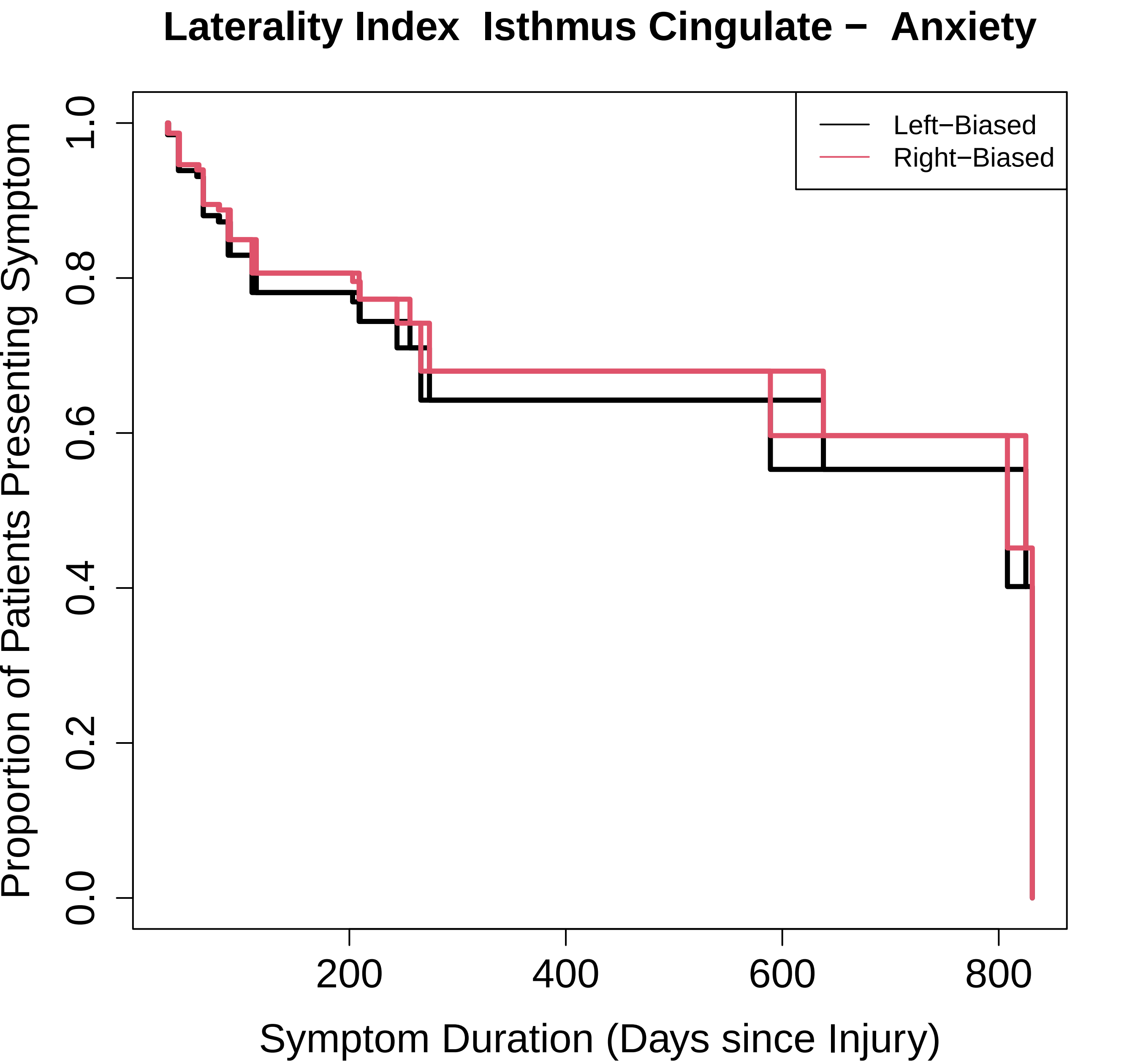

Laterality Index Isthmus Cingulate − Anxiety
Left−Biased
Right−Biased
Proportion of Patients Presenting Symptom
0.0
0.2
0.4
0.6
0.8
1.0
200
400
600
800
Symptom Duration (Days since Injury)

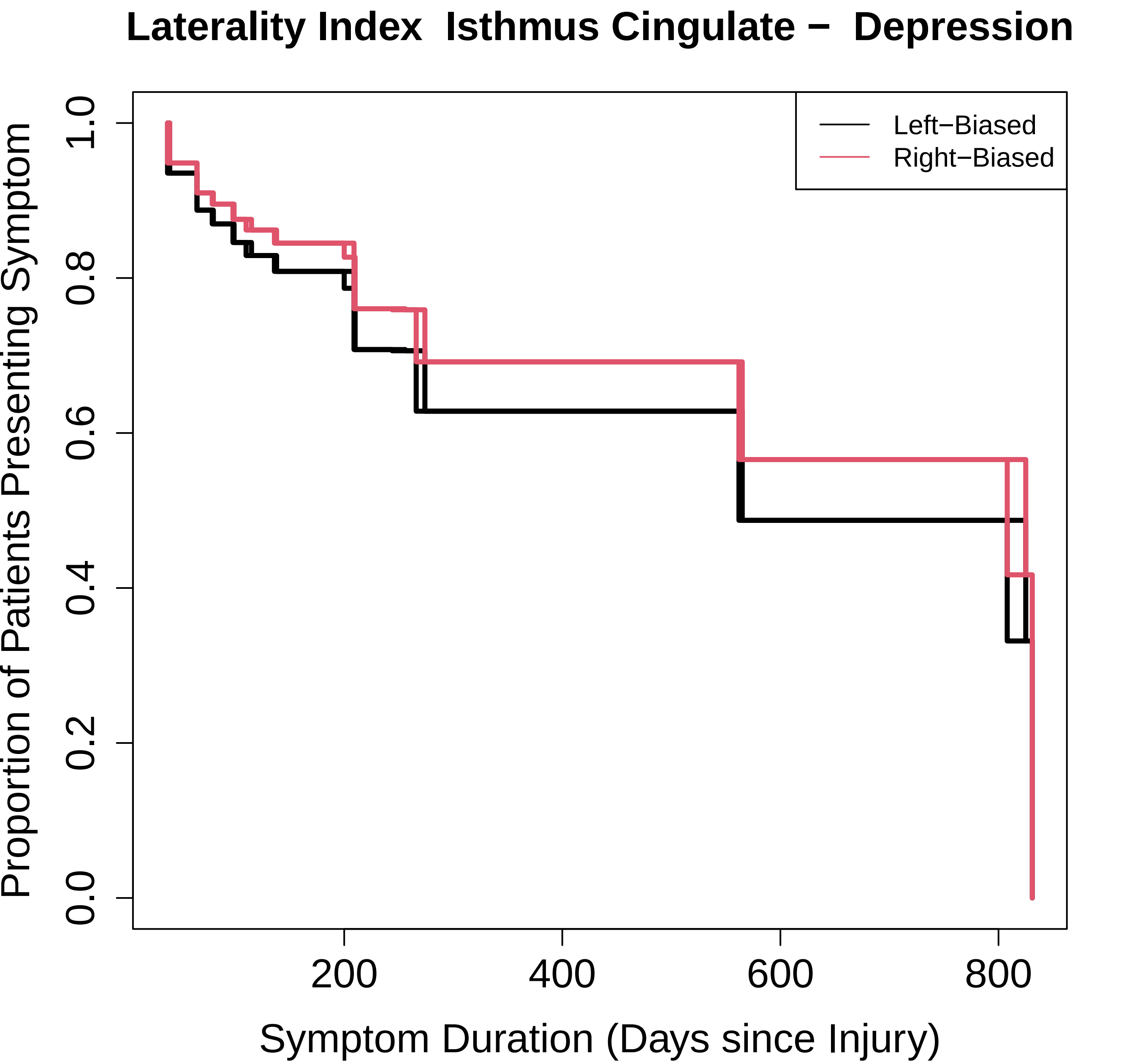

Laterality Index Isthmus Cingulate − Depression
Left−Biased
Right−Biased
Proportion of Patients Presenting Symptom
0.0
0.2
0.4
0.6
0.8
1.0
200
400
600
800
Symptom Duration (Days since Injury)

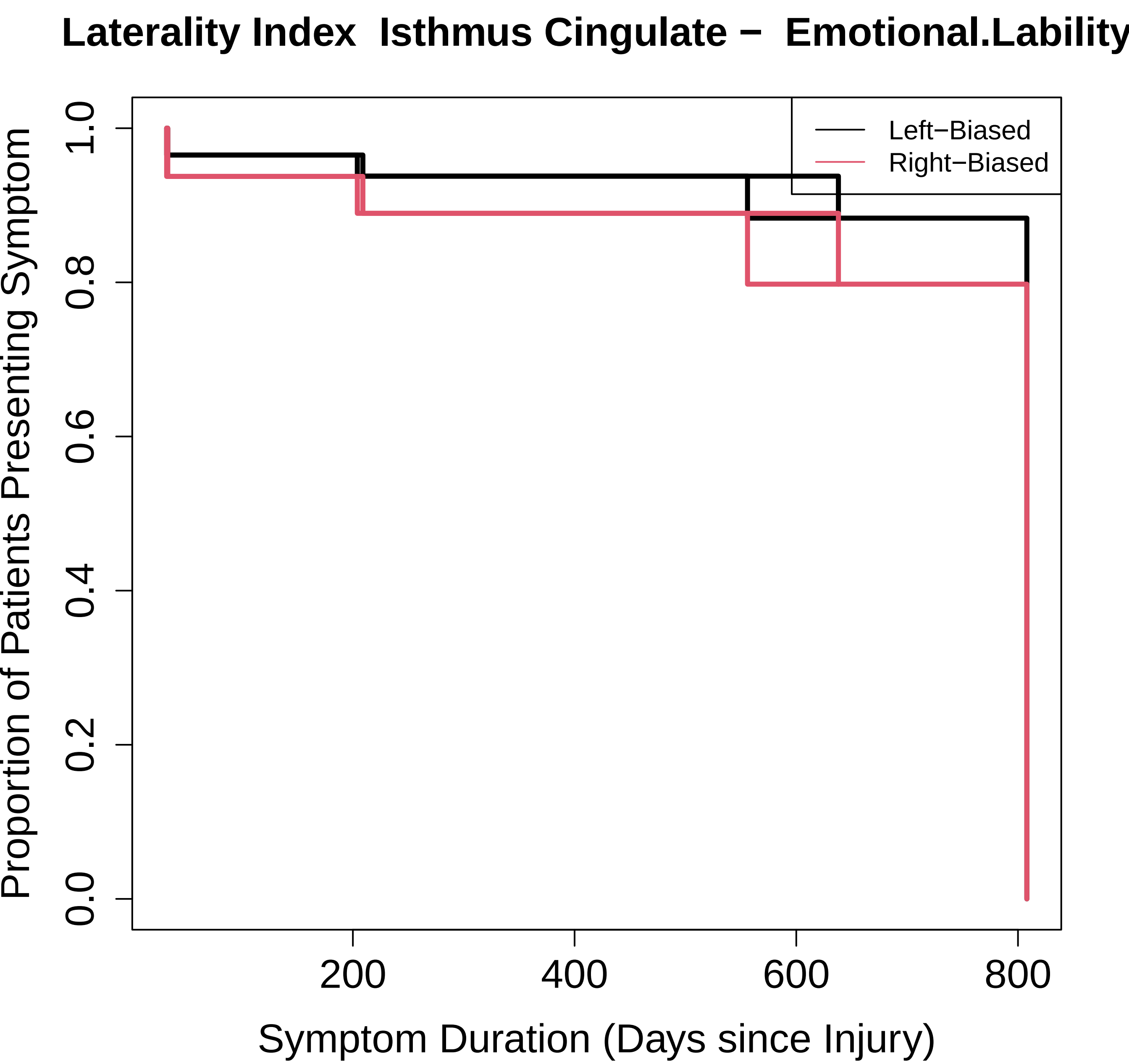

Laterality Index Isthmus Cingulate − Emotional.Lability
Left−Biased
Right−Biased
Proportion of Patients Presenting Symptom
0.0
0.2
0.4
0.6
0.8
1.0
200
400
600
800
Symptom Duration (Days since Injury)

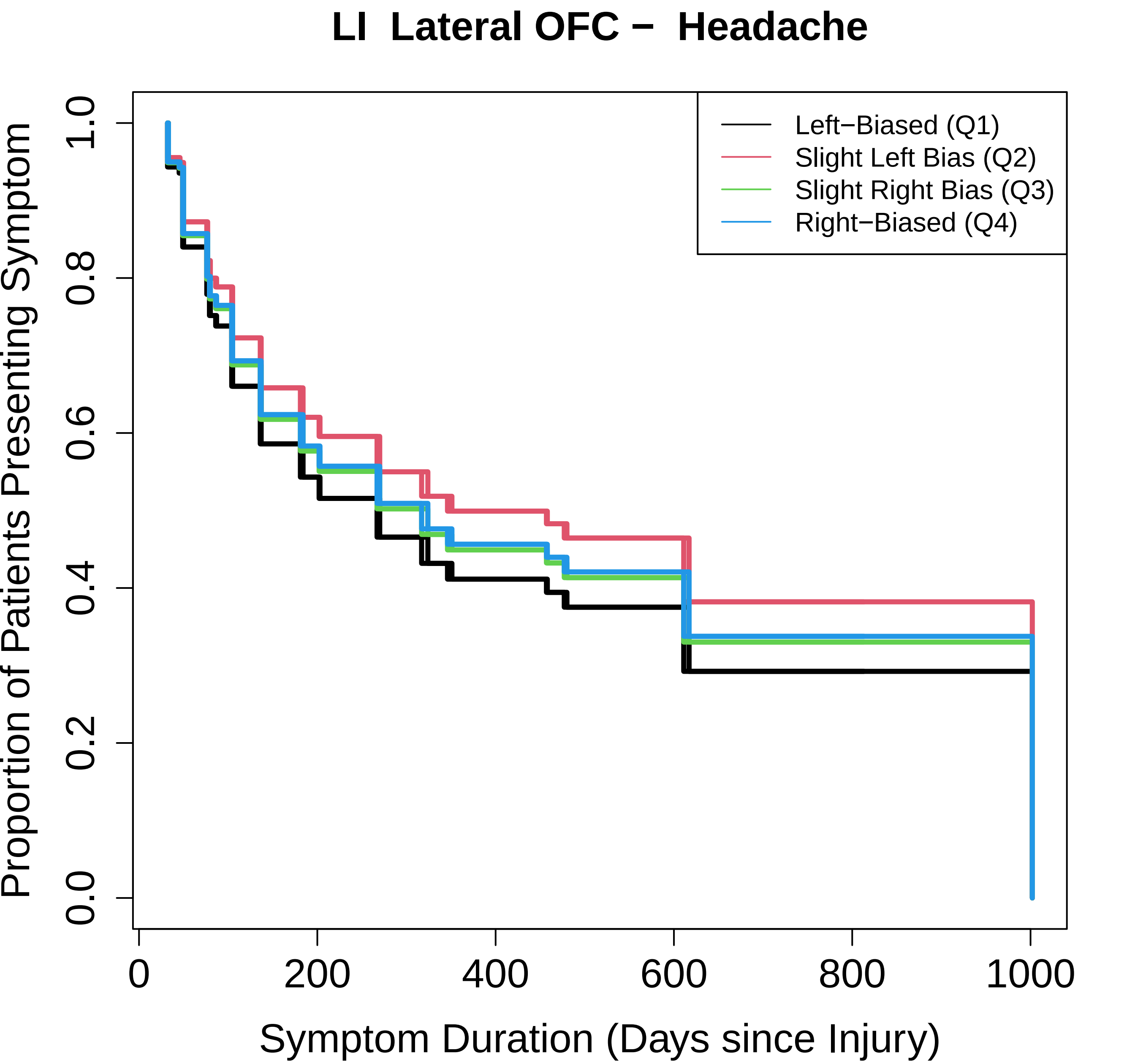

LI Lateral OFC − Headache
Left−Biased (Q1)
Slight Left Bias (Q2)
Slight Right Bias (Q3)
Right−Biased (Q4)
Proportion of Patients Presenting Symptom
0.0
0.2
0.4
0.6
0.8
1.0
0
200
400
600
800
1000
Symptom Duration (Days since Injury)

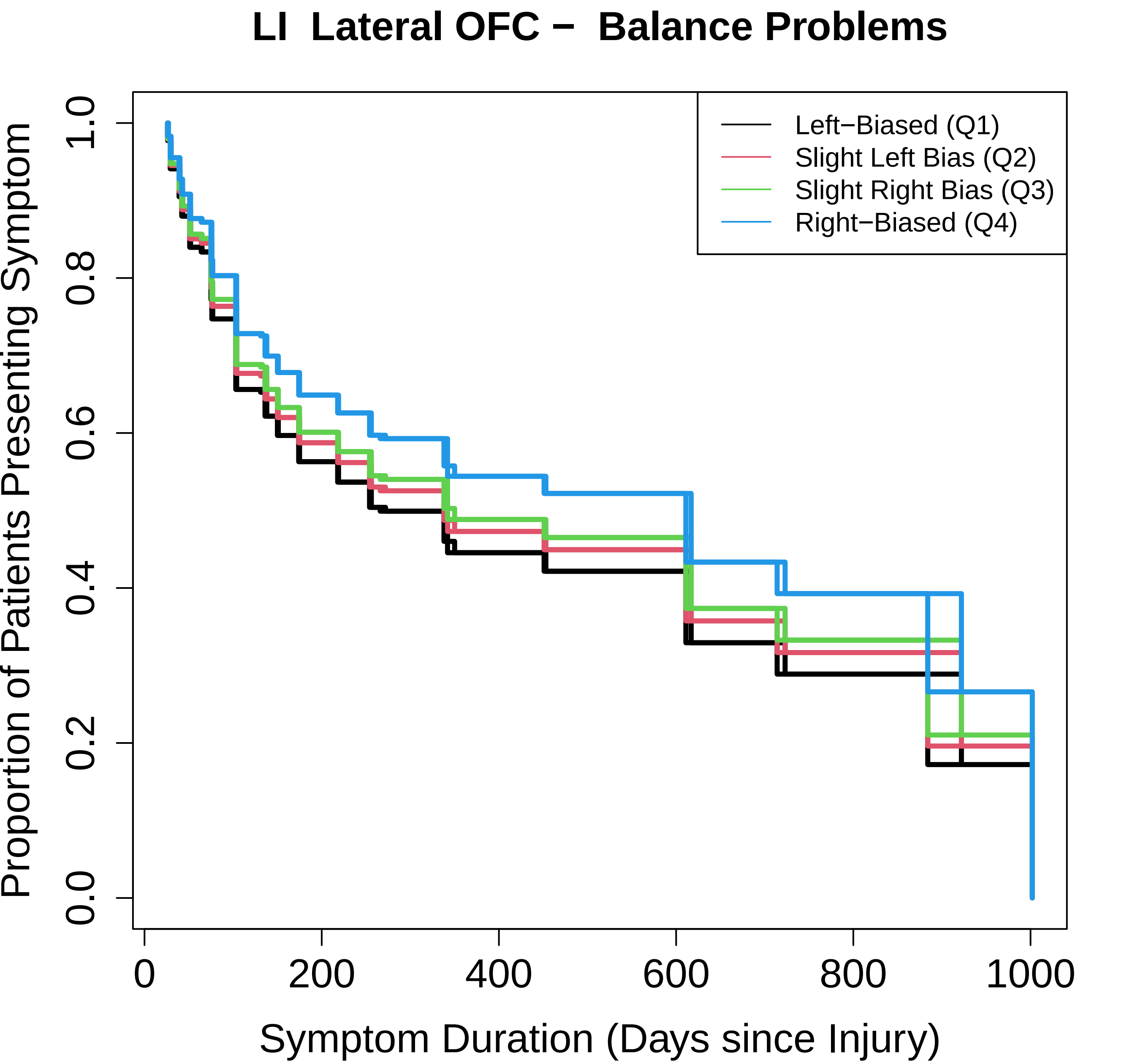

LI Lateral OFC − Balance Problems
Left−Biased (Q1)
Slight Left Bias (Q2)
Slight Right Bias (Q3)
Right−Biased (Q4)
Proportion of Patients Presenting Symptom
0.0
0.2
0.4
0.6
0.8
1.0
0
200
400
600
800
1000
Symptom Duration (Days since Injury)

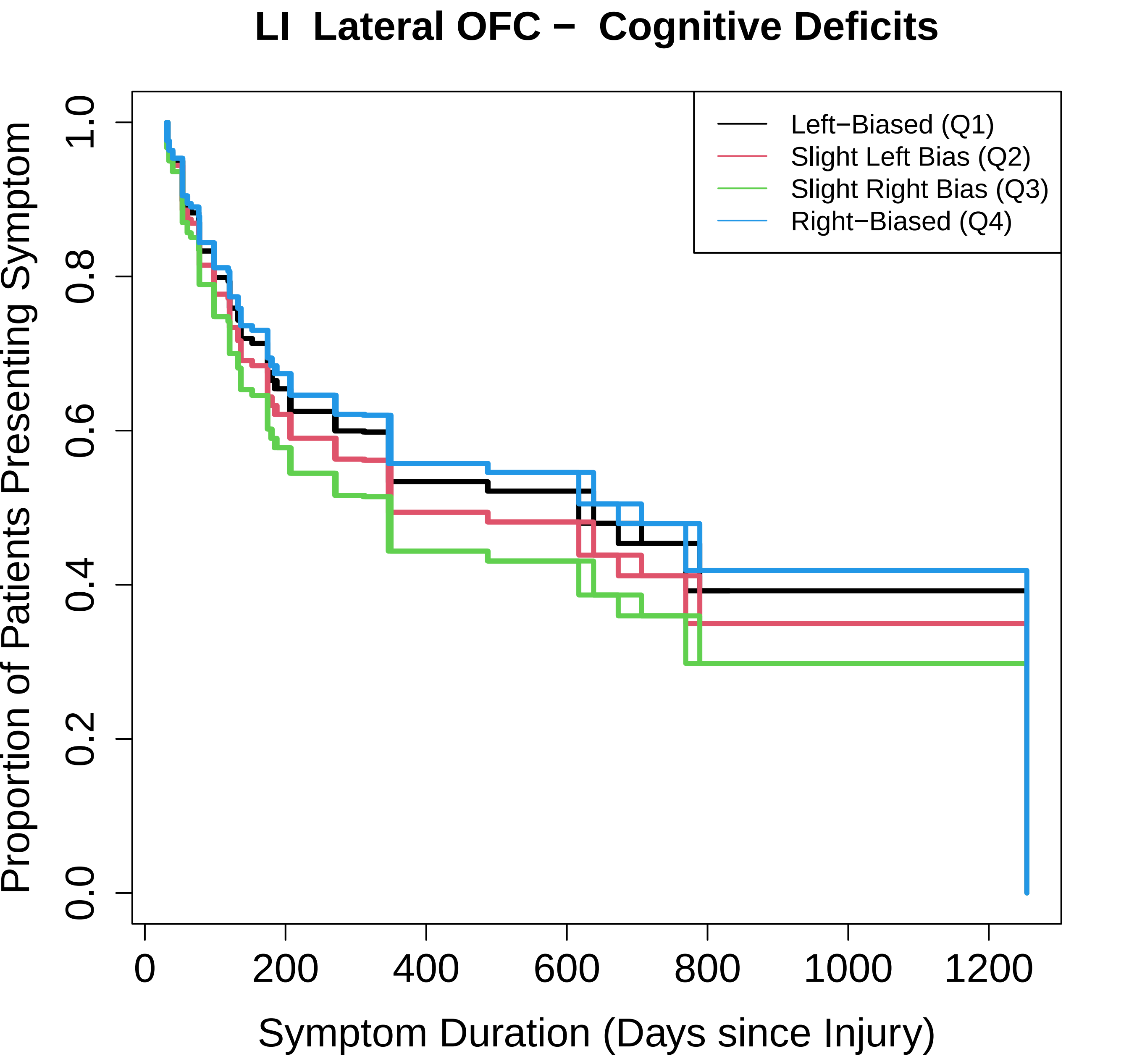

LI Lateral OFC − Cognitive Deficits
Left−Biased (Q1)
Slight Left Bias (Q2)
Slight Right Bias (Q3)
Right−Biased (Q4)
Proportion of Patients Presenting Symptom
0.0
0.2
0.4
0.6
0.8
1.0
0
200
400
600
800
1000
1200
Symptom Duration (Days since Injury)

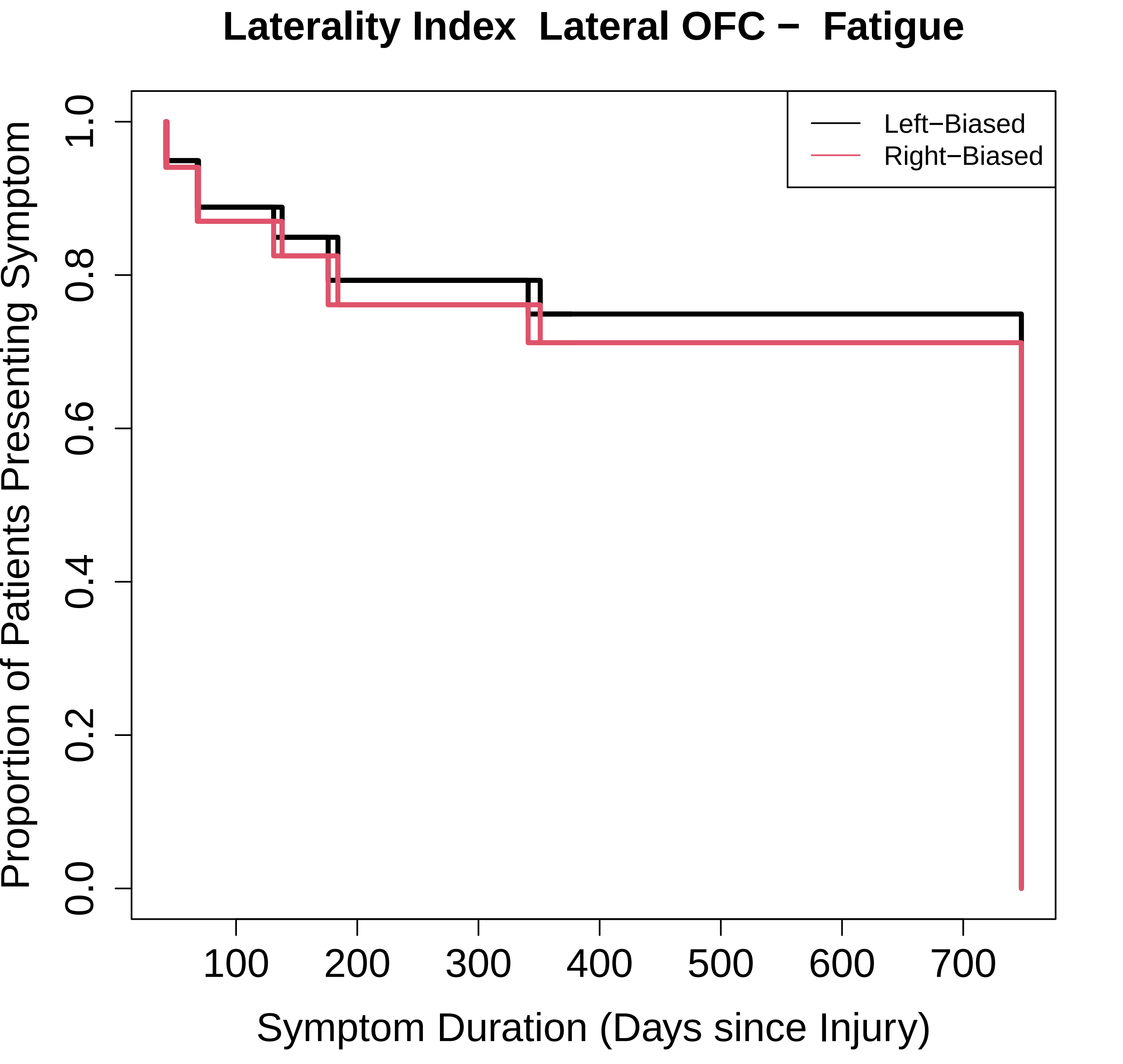

Laterality Index Lateral OFC − Fatigue
Left−Biased
Right−Biased
Proportion of Patients Presenting Symptom
1.0
0.8
0.6
0.4
0.2
0.0
100
200
300
400
500
600
700
Symptom Duration (Days since Injury)

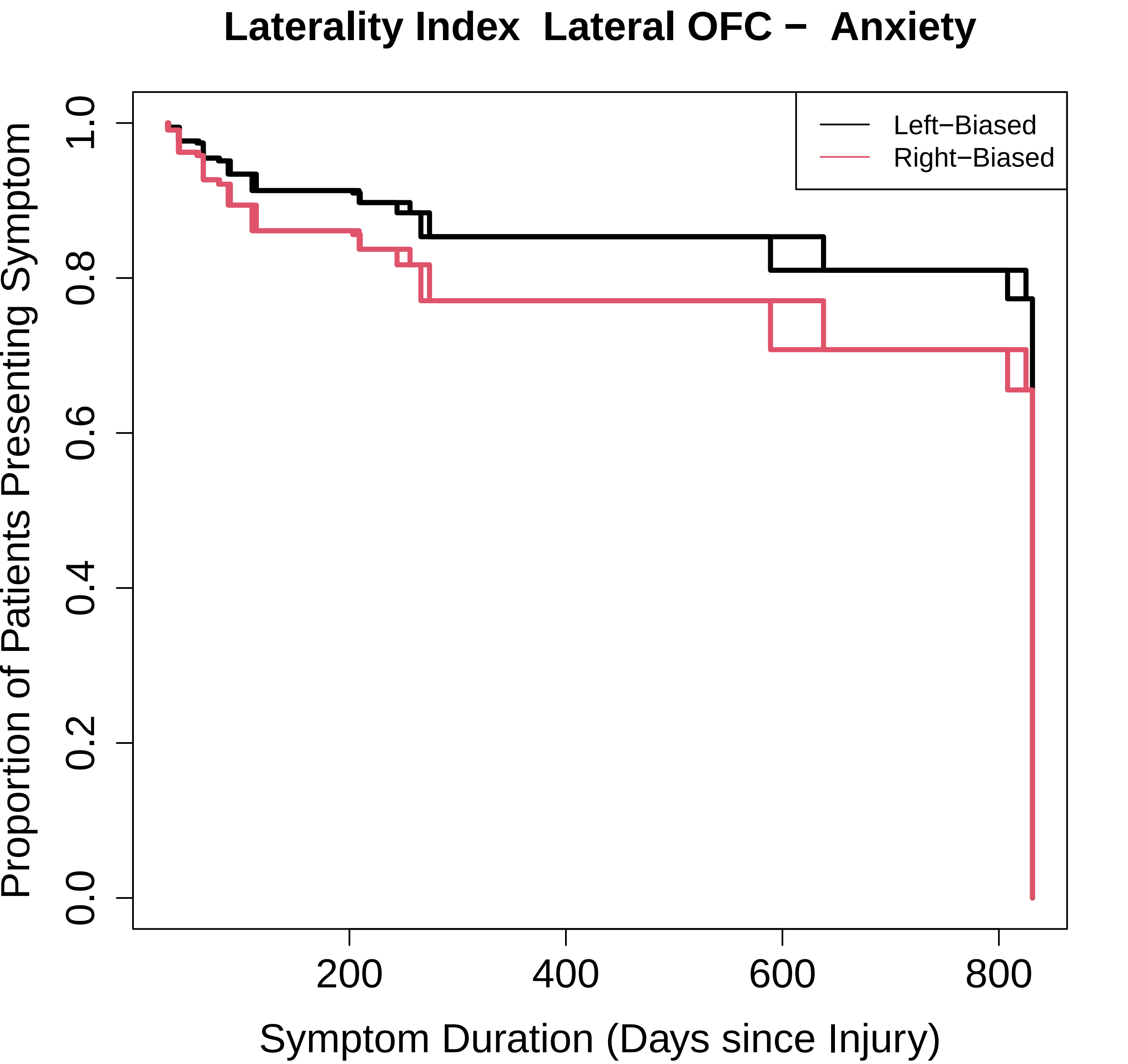

Laterality Index Lateral OFC − Anxiety
Proportion of Patients Presenting Symptom
Symptom Duration (Days since Injury)
0.0
0.2
0.4
0.6
0.8
1.0
200
400
600
800
Left−Biased
Right−Biased

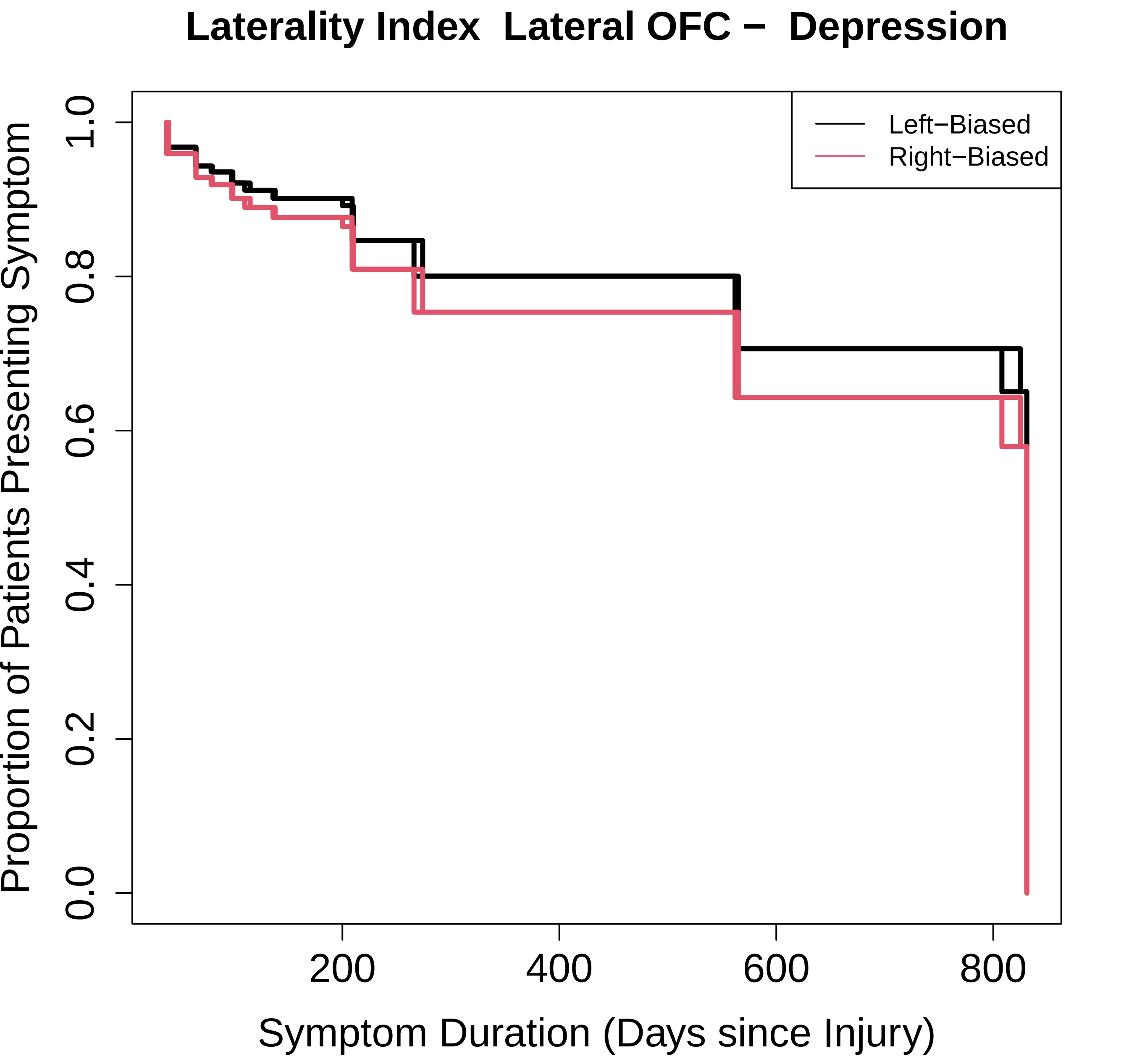

Laterality Index Lateral OFC − Depression
Left−Biased
Right−Biased
Proportion of Patients Presenting Symptom
0.0
0.2
0.4
0.6
0.8
1.0
200
400
600
800
Symptom Duration (Days since Injury)

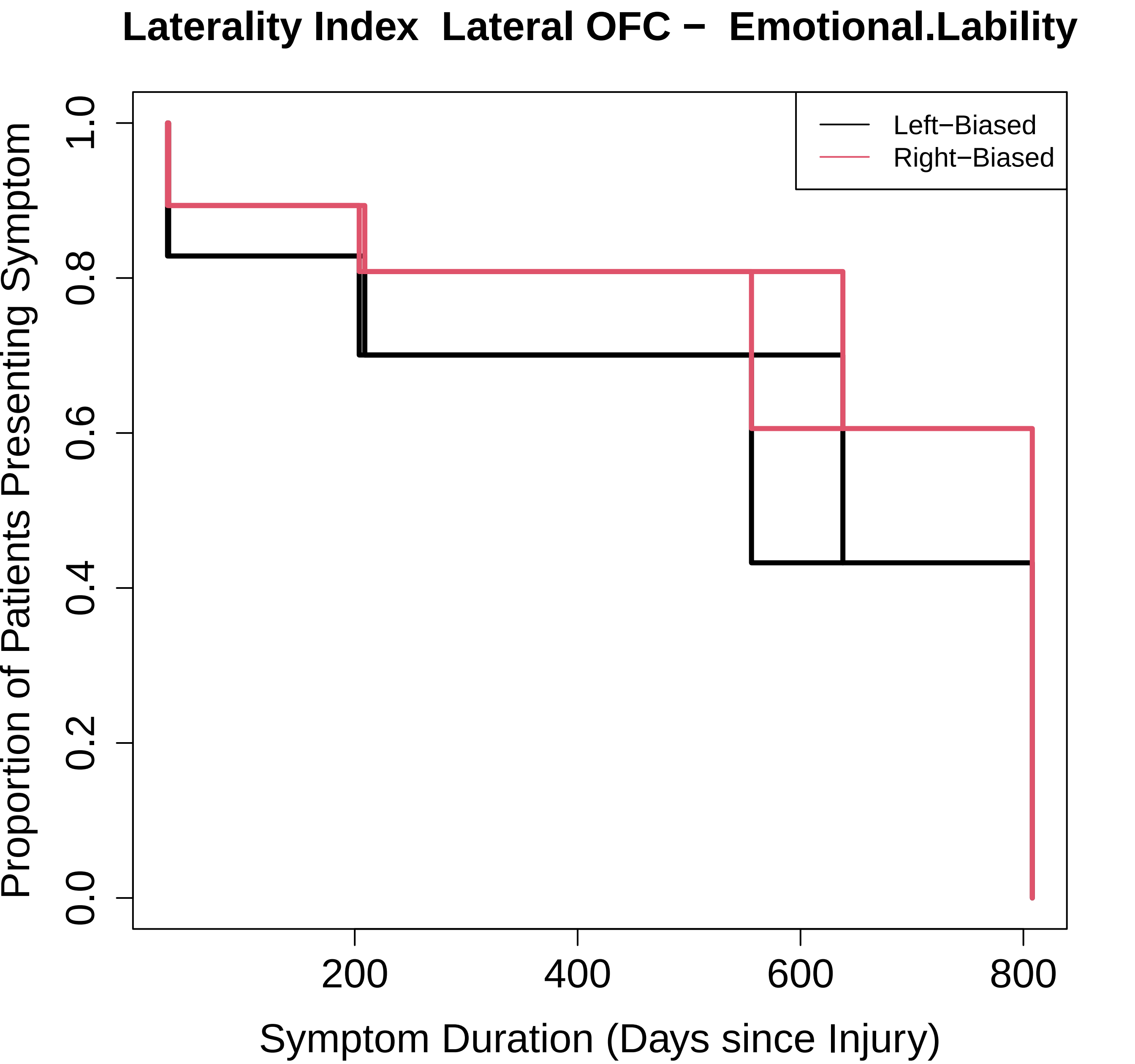

Laterality Index Lateral OFC − Emotional.Lability
Left−Biased
Right−Biased
Proportion of Patients Presenting Symptom
0.0
0.2
0.4
0.6
0.8
1.0
200
400
600
800
Symptom Duration (Days since Injury)

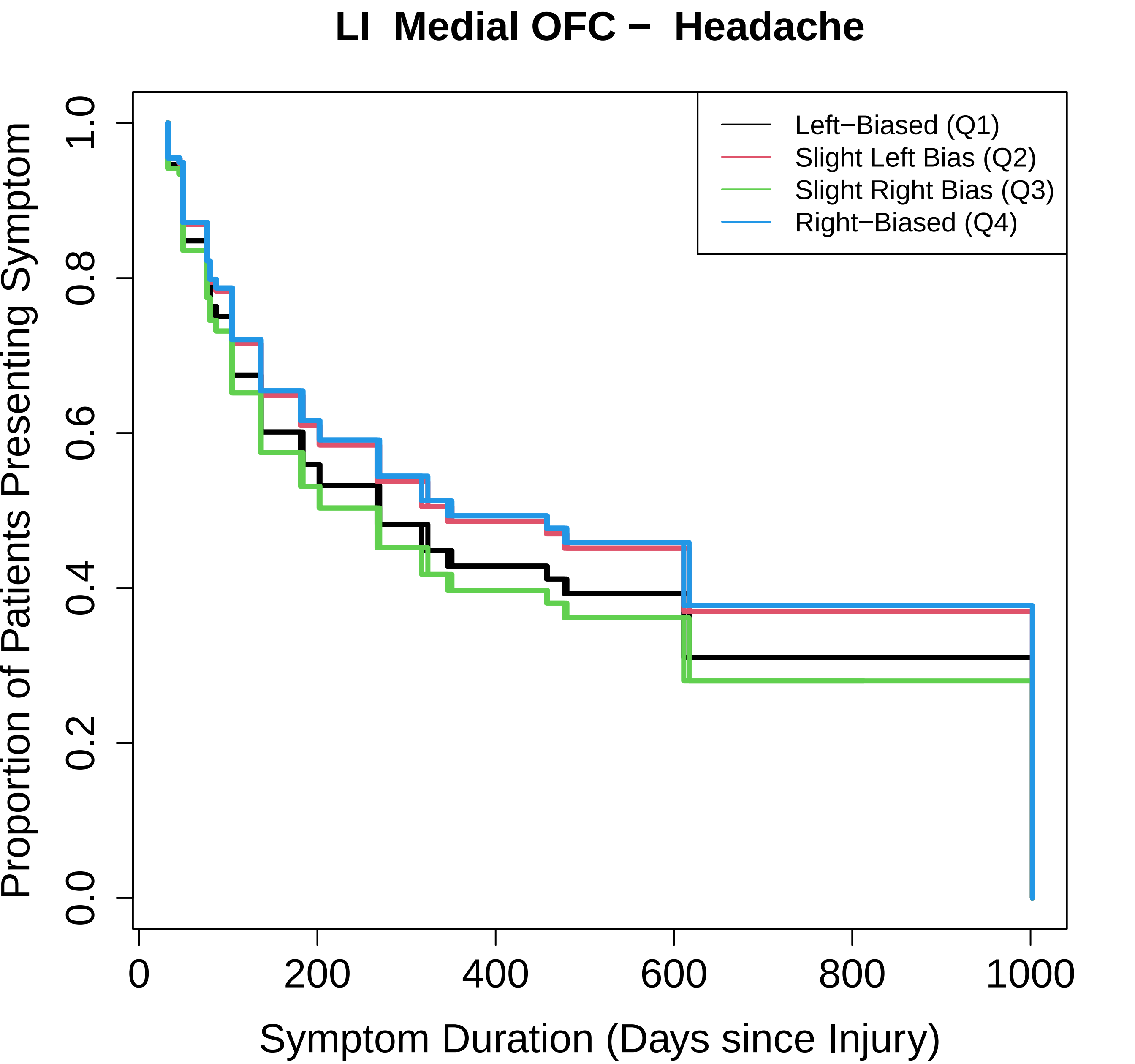
LI Medial OFC − Headache
Left−Biased (Q1)
Slight Left Bias (Q2)
Slight Right Bias (Q3)
Right−Biased (Q4)
Proportion of Patients Presenting Symptom
0.0
0.2
0.4
0.6
0.8
1.0
0
200
400
600
800
1000
Symptom Duration (Days since Injury)

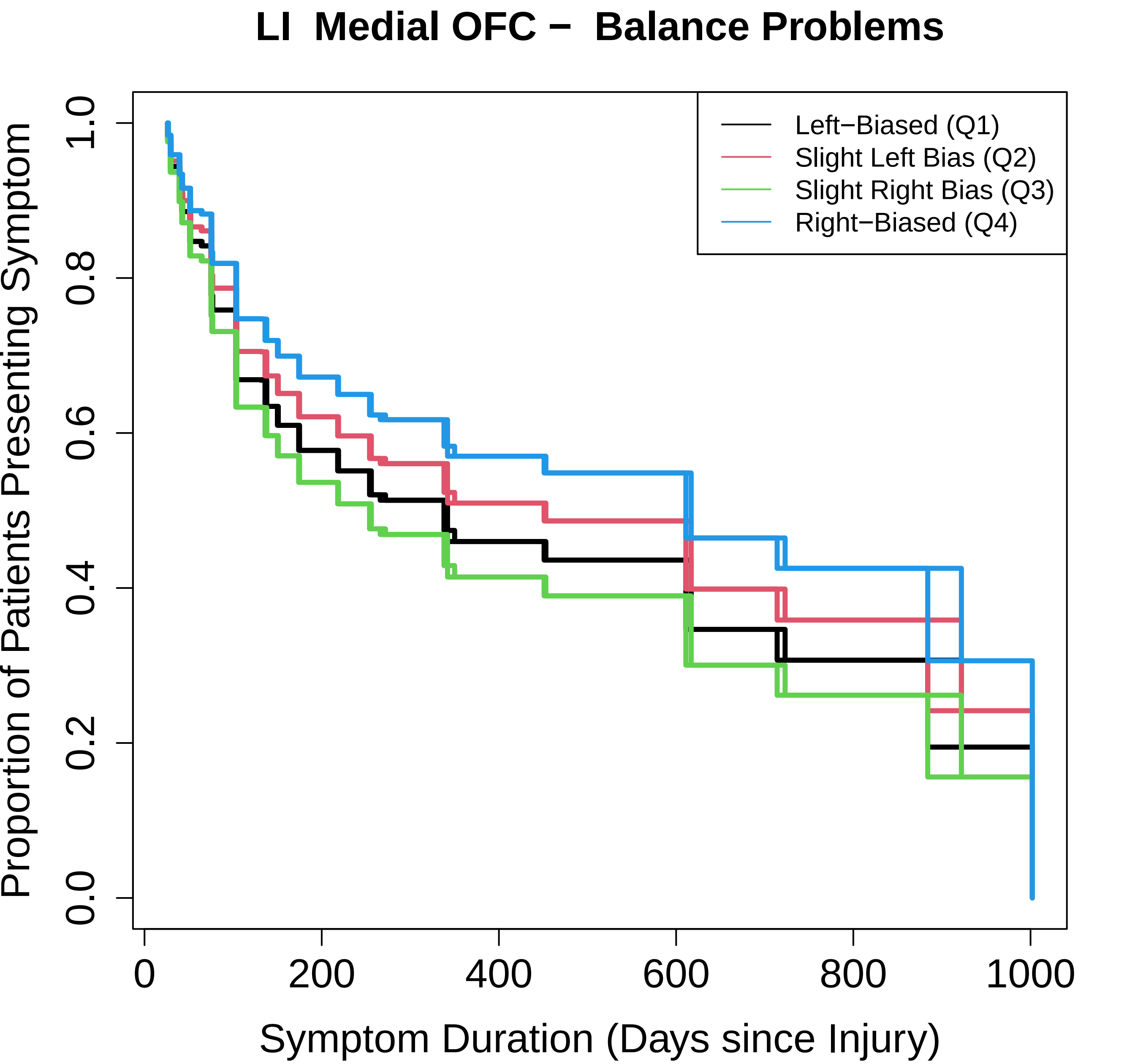
LI Medial OFC − Balance Problems
Left−Biased (Q1)
Slight Left Bias (Q2)
Slight Right Bias (Q3)
Right−Biased (Q4)
Proportion of Patients Presenting Symptom
0.0
0.2
0.4
0.6
0.8
1.0
0
200
400
600
800
1000
Symptom Duration (Days since Injury)

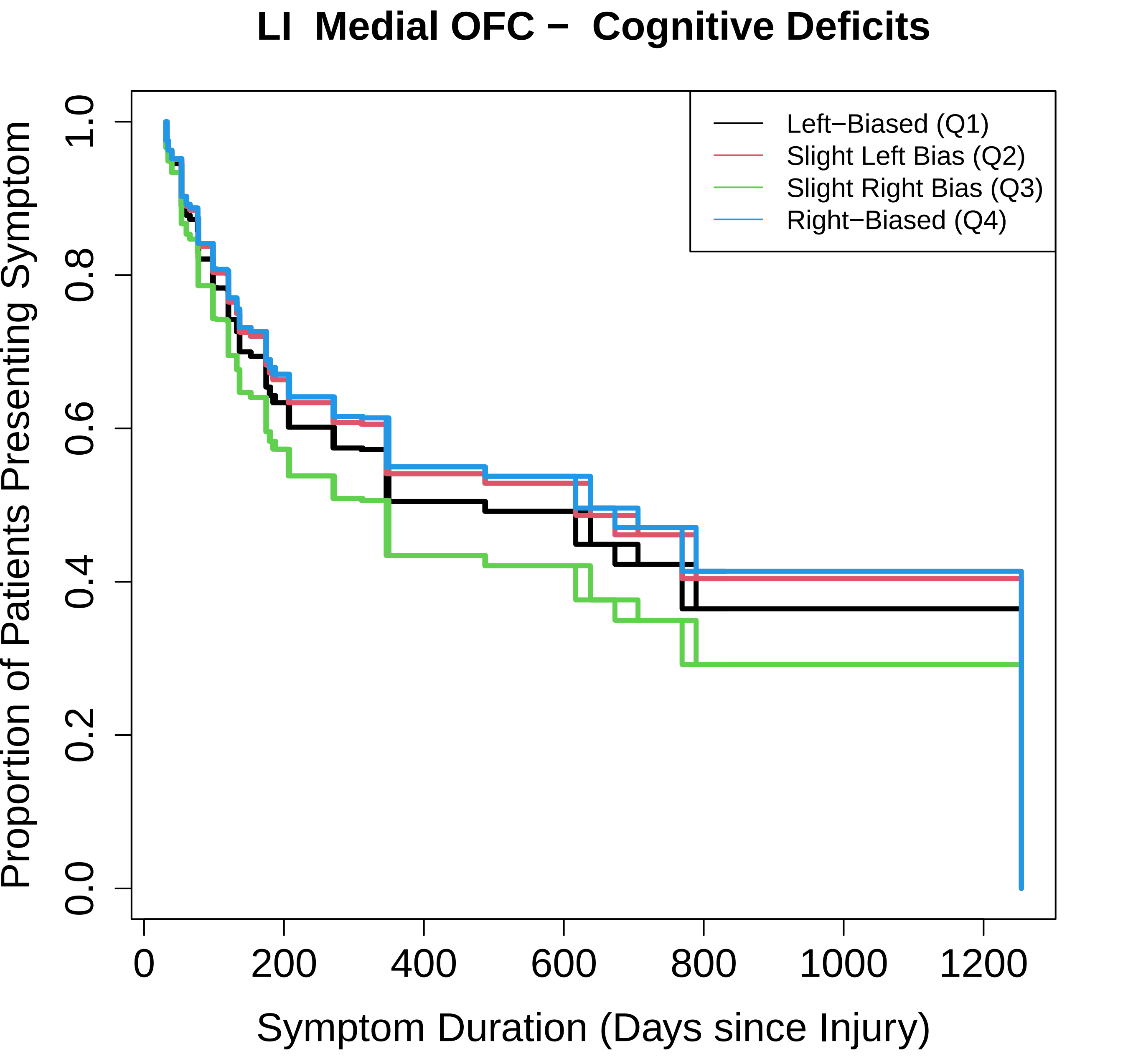

LI Medial OFC − Cognitive Deficits
Left−Biased (Q1)
Slight Left Bias (Q2)
Slight Right Bias (Q3)
Right−Biased (Q4)
Proportion of Patients Presenting Symptom
0.0
0.2
0.4
0.6
0.8
1.0
Symptom Duration (Days since Injury)
0
200
400
600
800
1000
1200

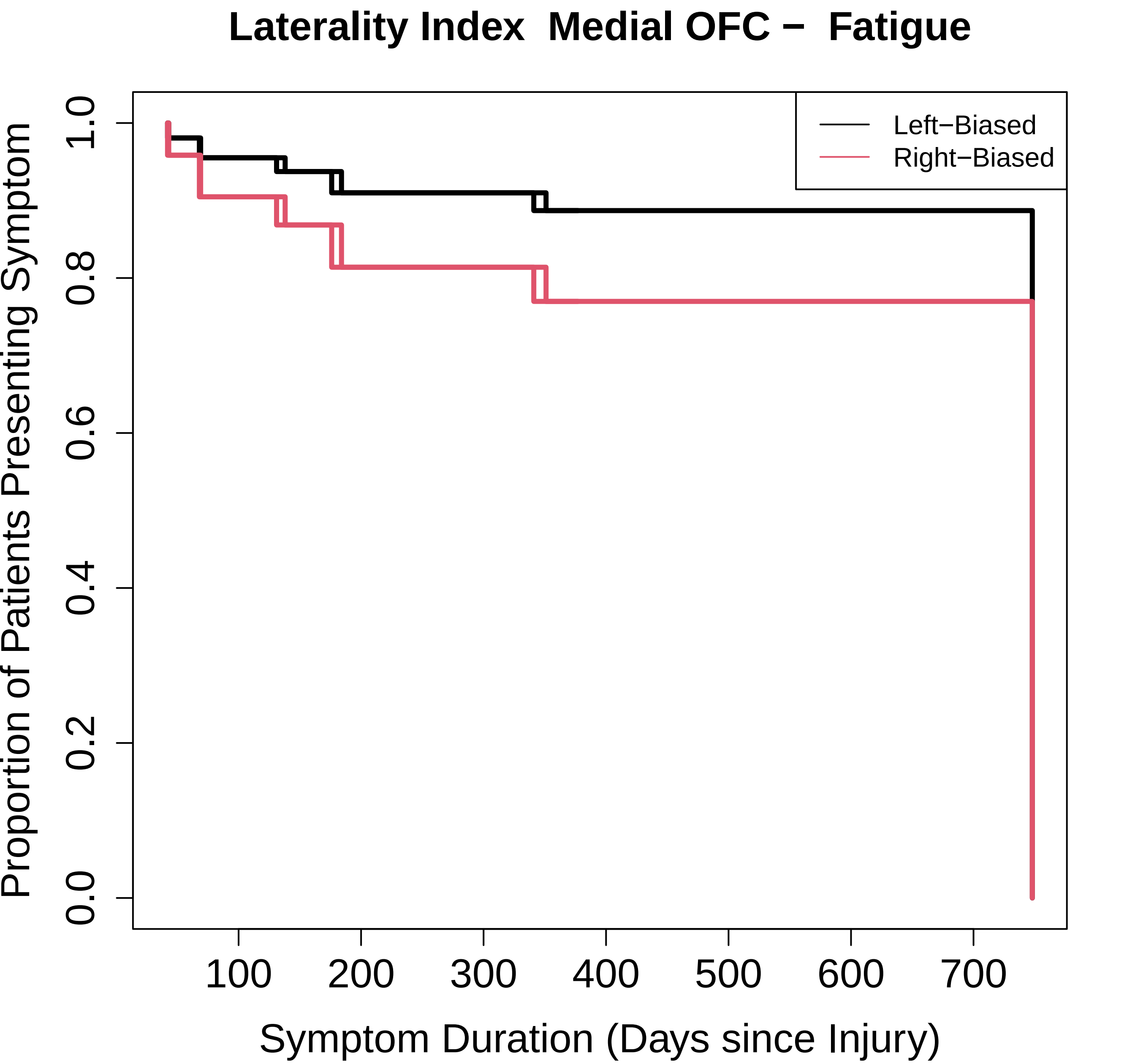

Laterality Index Medial OFC − Fatigue
Proportion of Patients Presenting Symptom
Symptom Duration (Days since Injury)
Left−Biased
Right−Biased
0.0
0.2
0.4
0.6
0.8
1.0
100
200
300
400
500
600
700

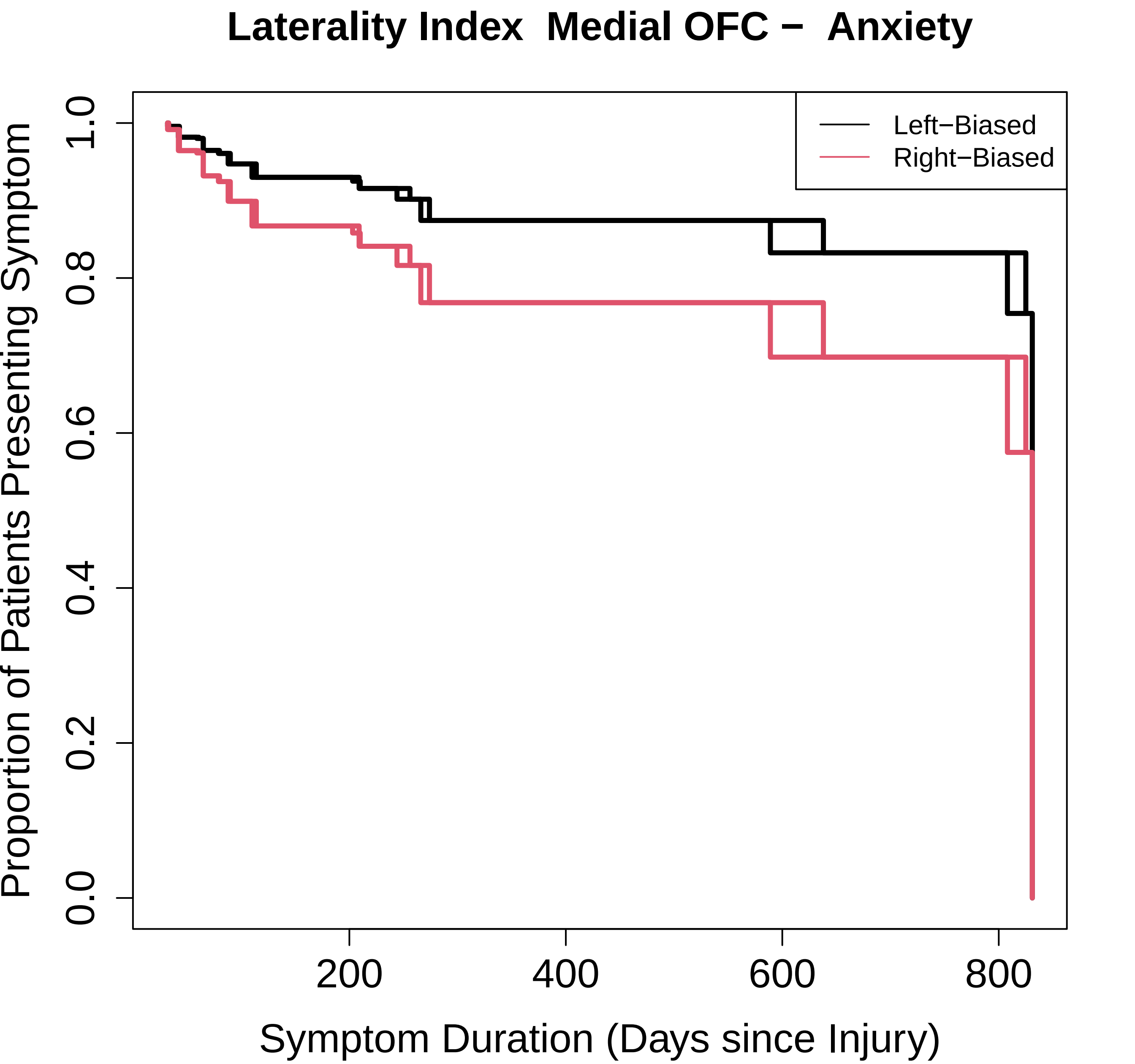

Laterality Index Medial OFC − Anxiety
Left−Biased
Right−Biased
Proportion of Patients Presenting Symptom
0.0
0.2
0.4
0.6
0.8
1.0
200
400
600
800
Symptom Duration (Days since Injury)

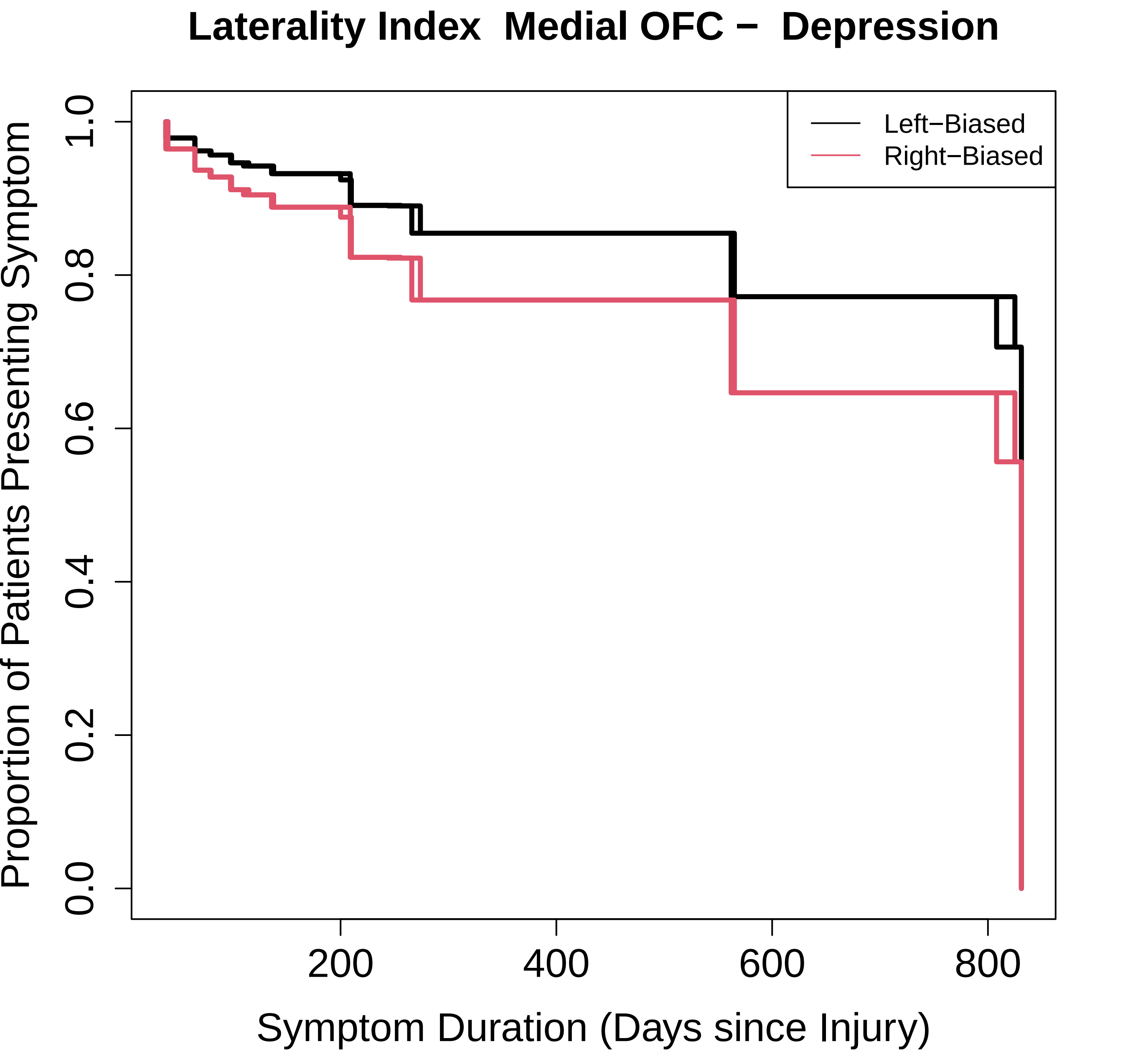
Laterality Index Medial OFC − Depression
Left−Biased
Right−Biased
Proportion of Patients Presenting Symptom
0.0
0.2
0.4
0.6
0.8
1.0
200
400
600
800
Symptom Duration (Days since Injury)

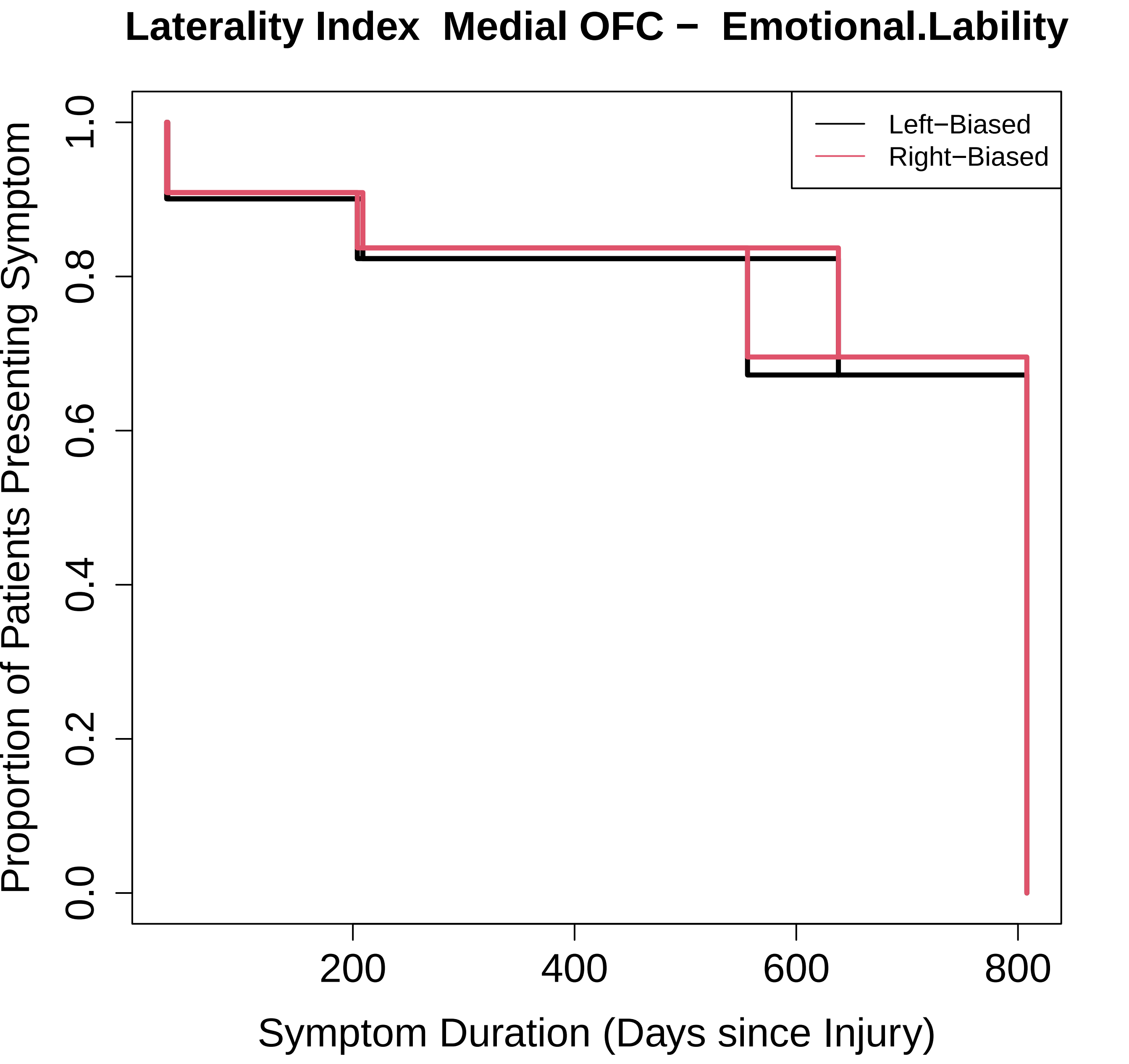

Laterality Index Medial OFC − Emotional.Lability
Left−Biased
Right−Biased
Proportion of Patients Presenting Symptom
0.0
0.2
0.4
0.6
0.8
1.0
200
400
600
800
Symptom Duration (Days since Injury)

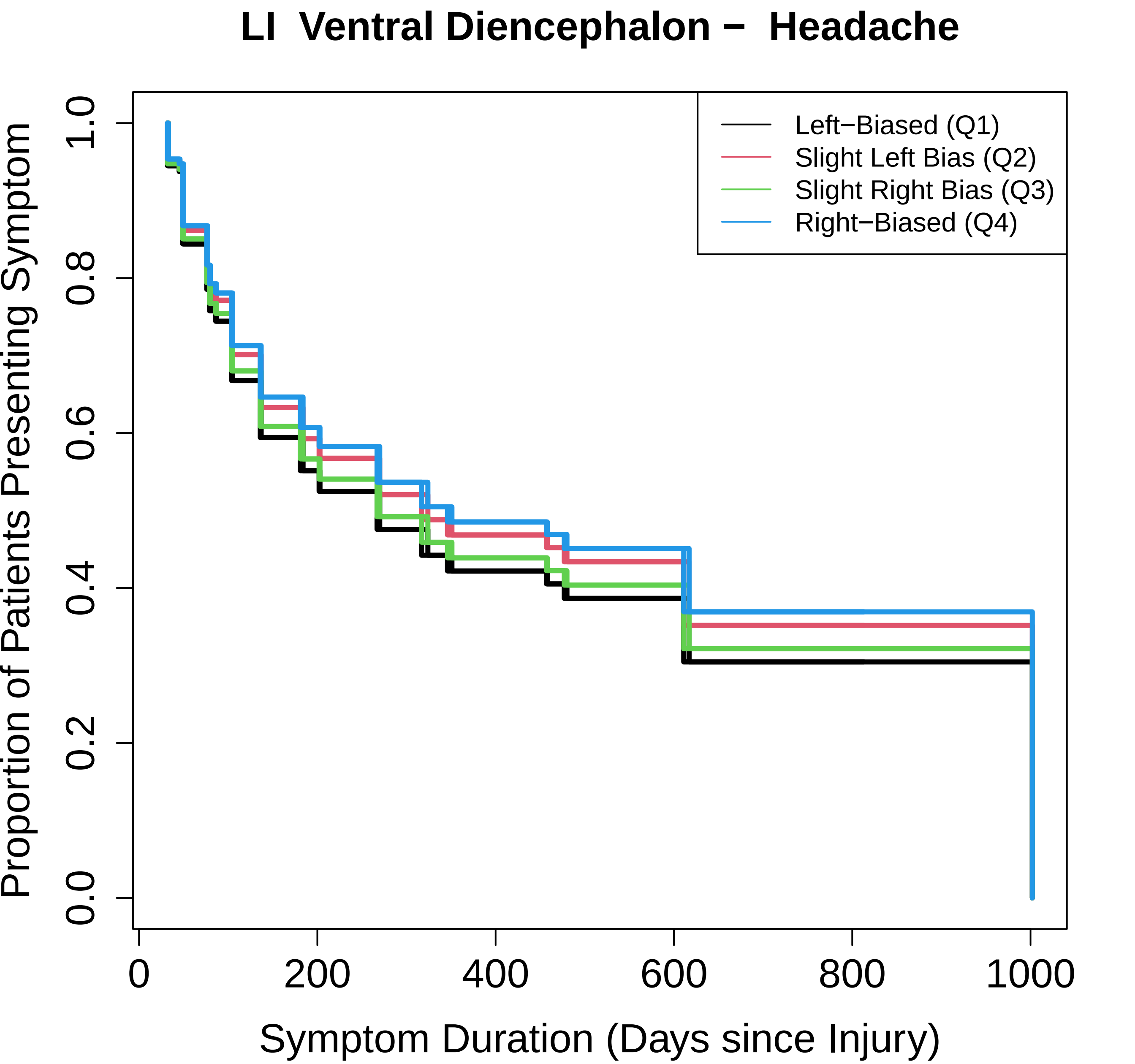
LI Ventral Diencephalon − Headache
Left−Biased (Q1)
Slight Left Bias (Q2)
Slight Right Bias (Q3)
Right−Biased (Q4)
Proportion of Patients Presenting Symptom
0.0
0.2
0.4
0.6
0.8
1.0
0
200
400
600
800
1000
Symptom Duration (Days since Injury)

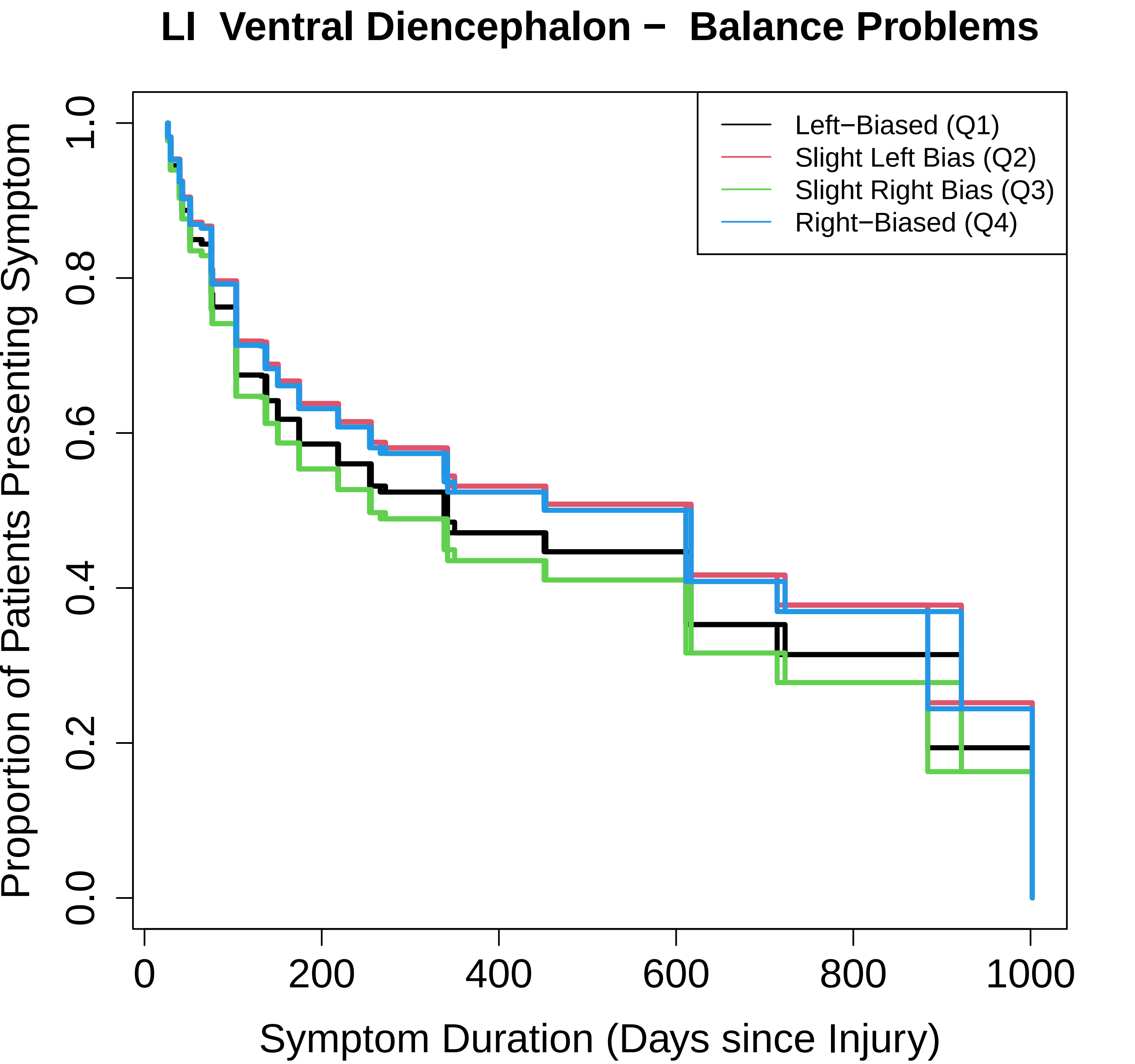

LI Ventral Diencephalon − Balance Problems
Left−Biased (Q1)
Slight Left Bias (Q2)
Slight Right Bias (Q3)
Right−Biased (Q4)
Proportion of Patients Presenting Symptom
0.0
0.2
0.4
0.6
0.8
1.0
0
200
400
600
800
1000
Symptom Duration (Days since Injury)

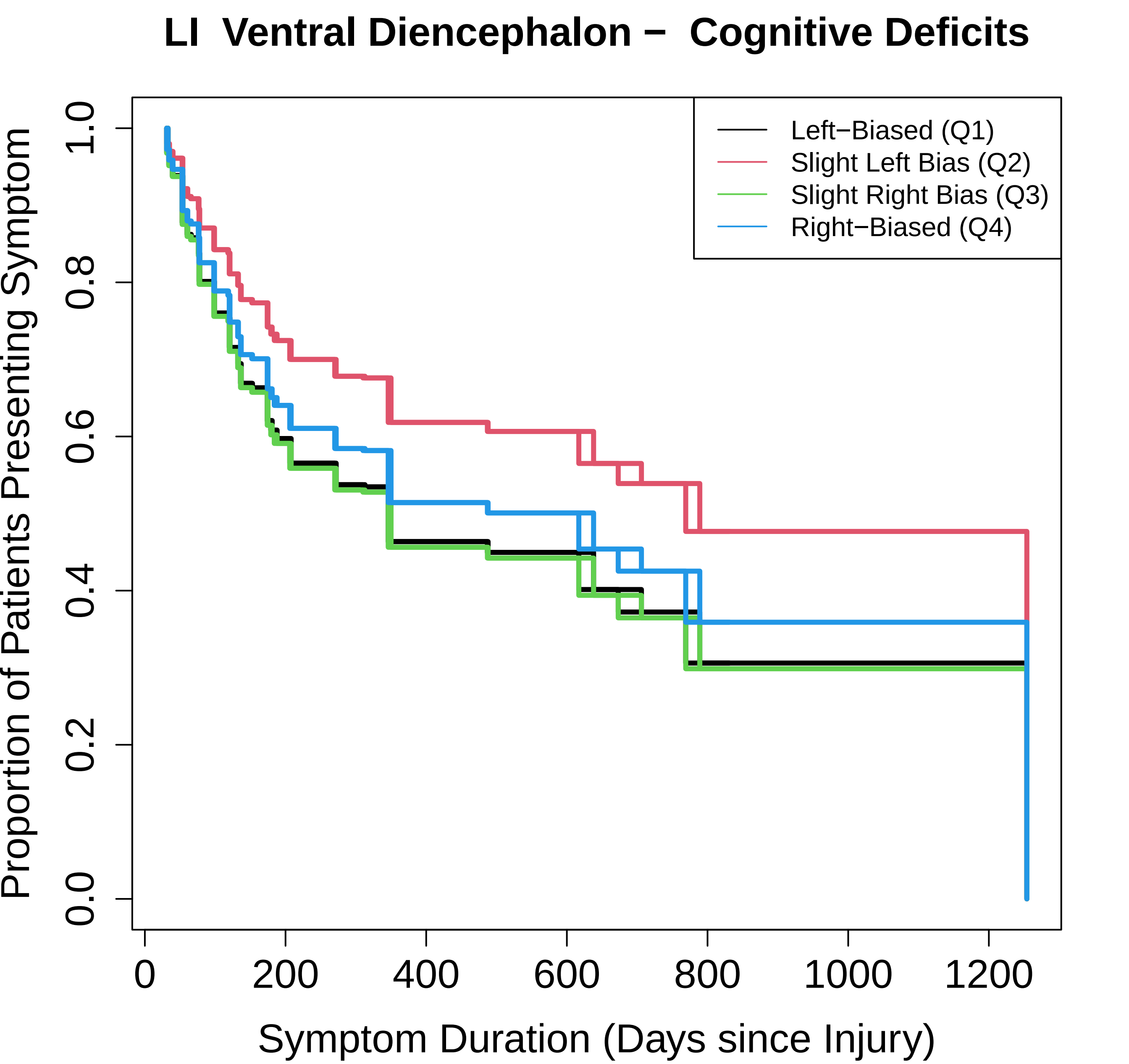

LI Ventral Diencephalon − Cognitive Deficits
Left−Biased (Q1)
Slight Left Bias (Q2)
Slight Right Bias (Q3)
Right−Biased (Q4)
Proportion of Patients Presenting Symptom
0.0
0.2
0.4
0.6
0.8
1.0
0
200
400
600
800
1000
1200
Symptom Duration (Days since Injury)

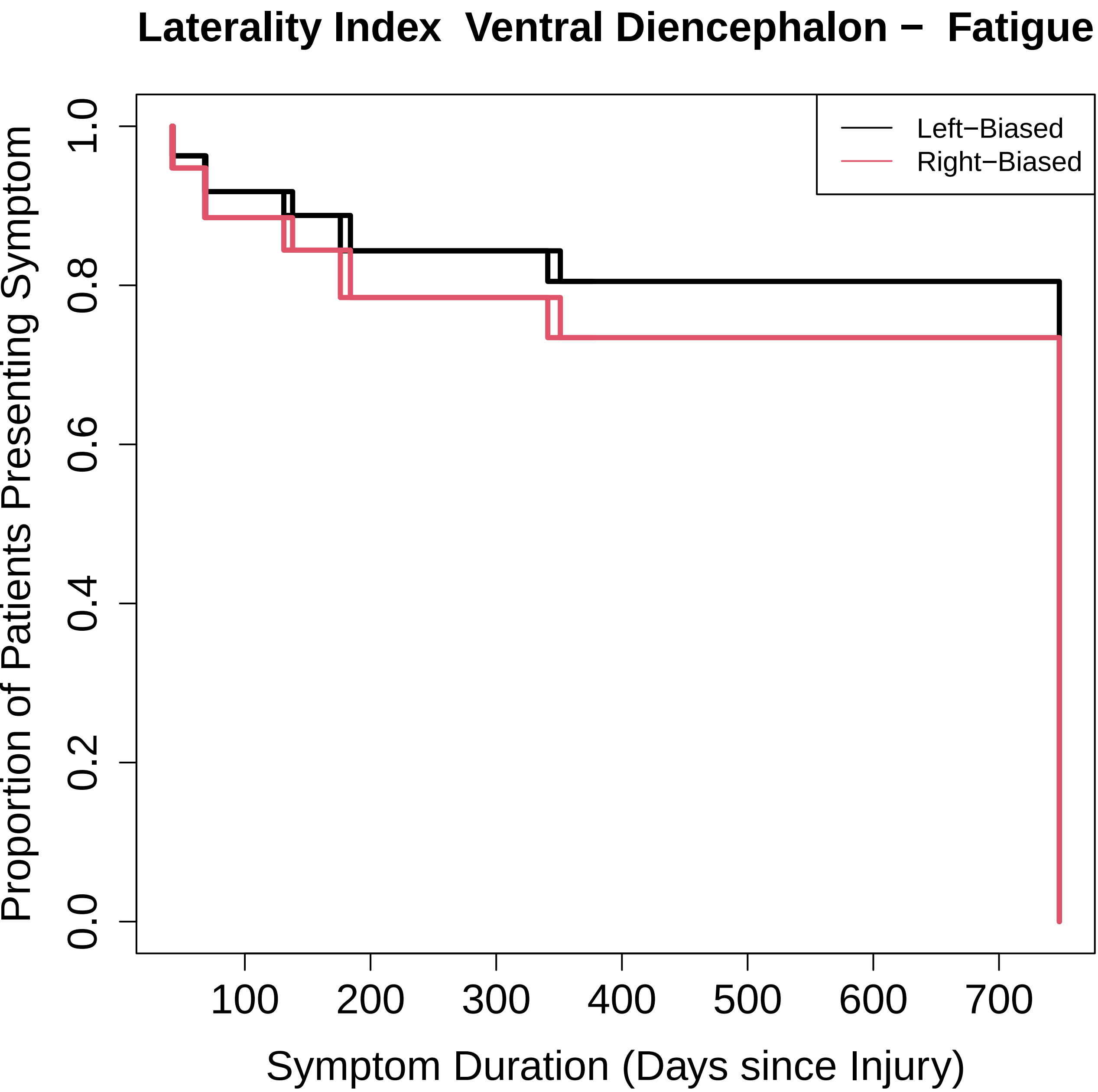
Laterality Index Ventral Diencephalon − Fatigue
Left−Biased
Right−Biased
Proportion of Patients Presenting Symptom
0.0
0.2
0.4
0.6
0.8
1.0
100
200
300
400
500
600
700
Symptom Duration (Days since Injury)

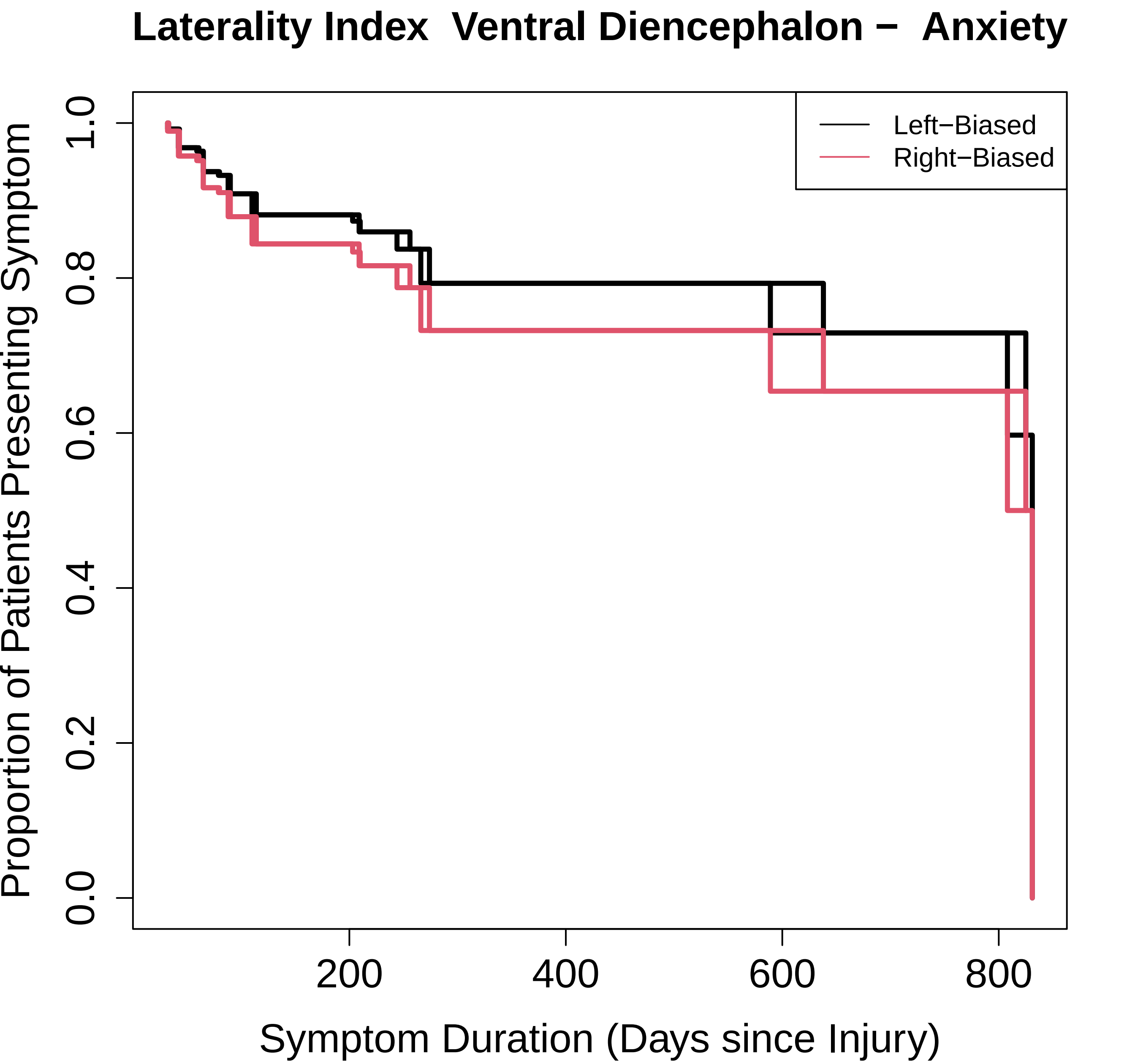

Laterality Index Ventral Diencephalon − Anxiety
Left−Biased
Right−Biased
Proportion of Patients Presenting Symptom
0.0
0.2
0.4
0.6
0.8
1.0
200
400
600
800
Symptom Duration (Days since Injury)

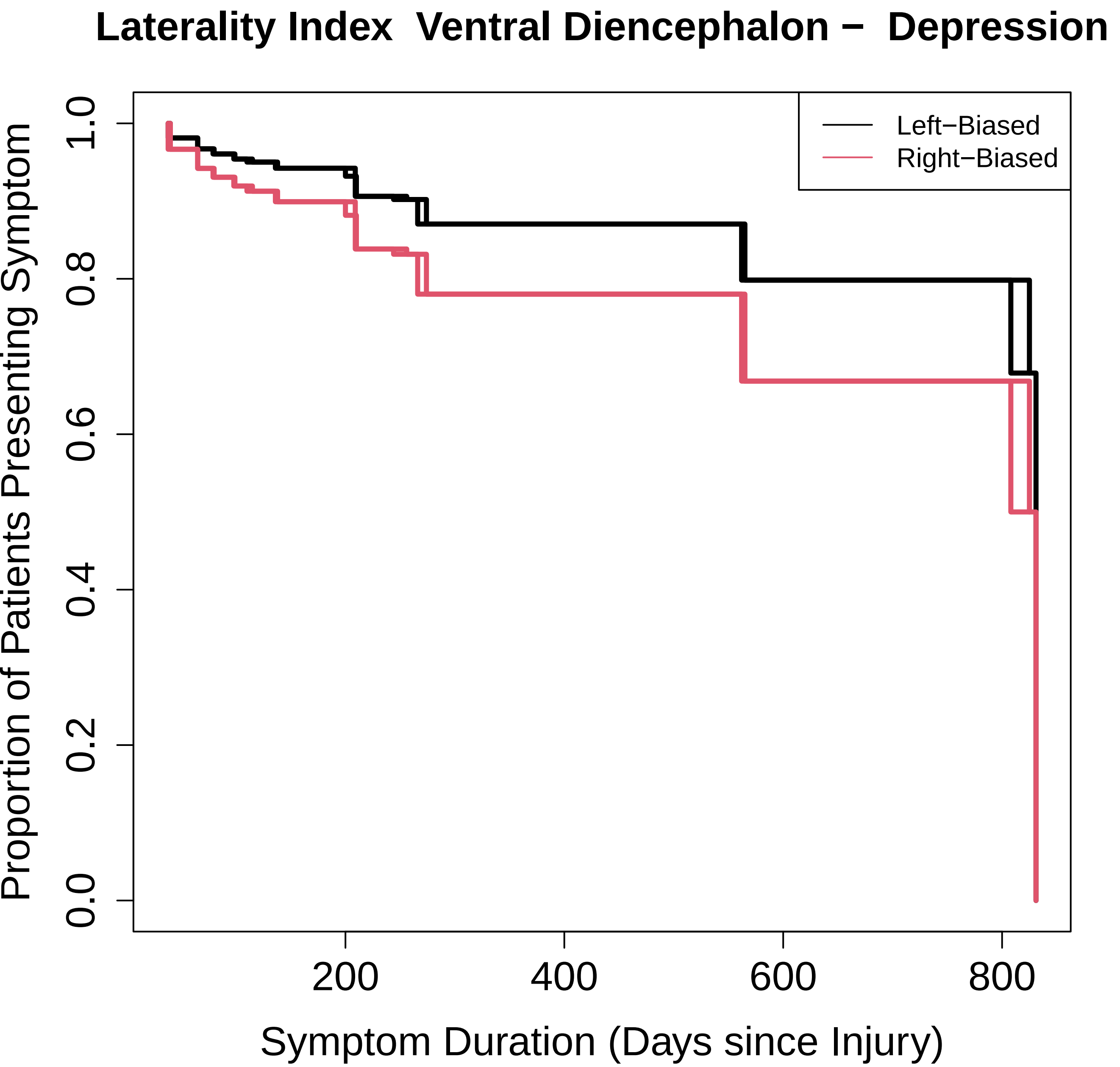
Laterality Index Ventral Diencephalon − Depression
Left−Biased
Right−Biased
Proportion of Patients Presenting Symptom
0.0
0.2
0.4
0.6
0.8
1.0
200
400
600
800
Symptom Duration (Days since Injury)

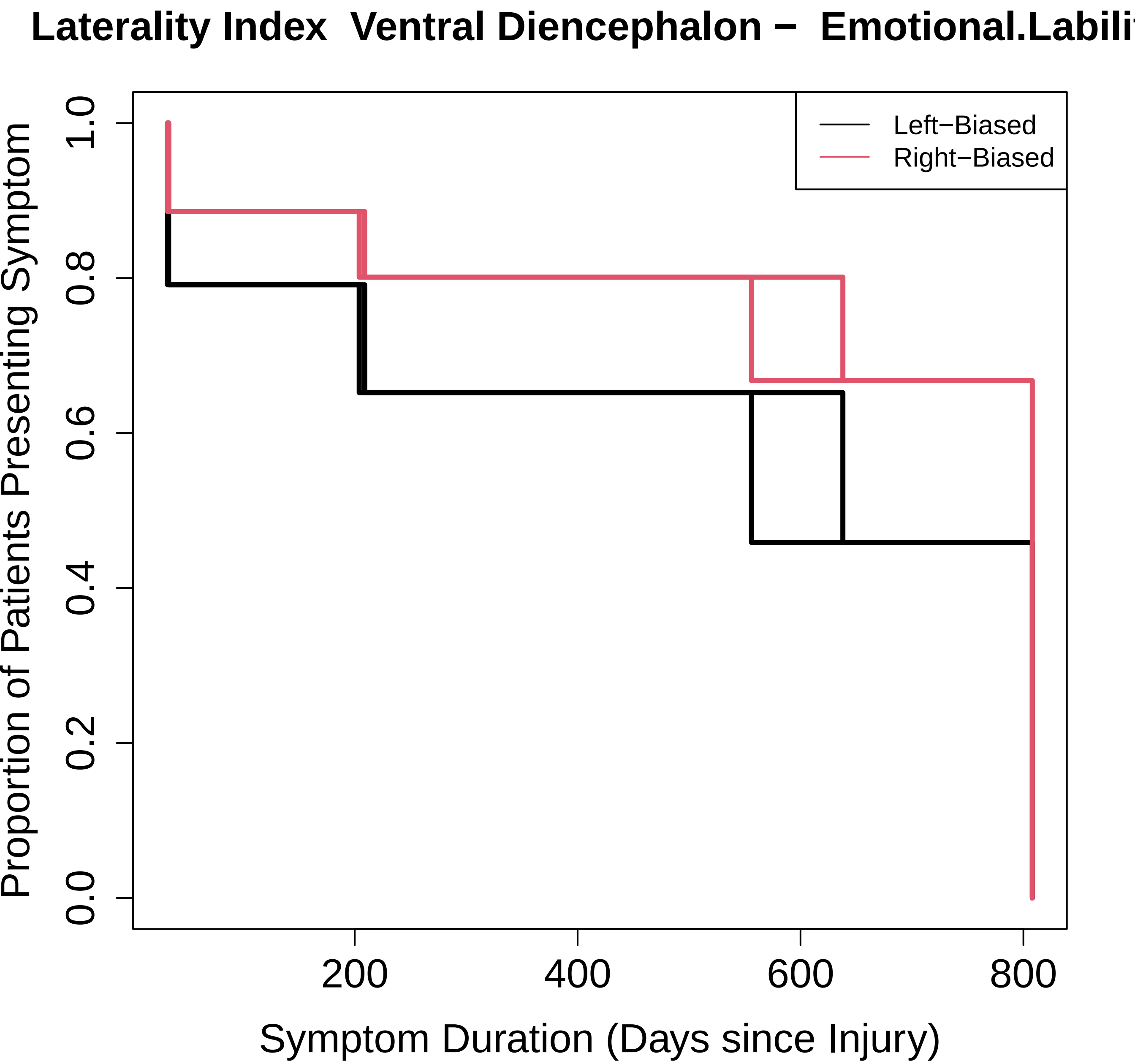

Laterality Index Ventral Diencephalon − Emotional.Labilit
Left−Biased
Right−Biased
Proportion of Patients Presenting Symptom
0.0
0.2
0.4
0.6
0.8
1.0
200
400
600
800
Symptom Duration (Days since Injury)

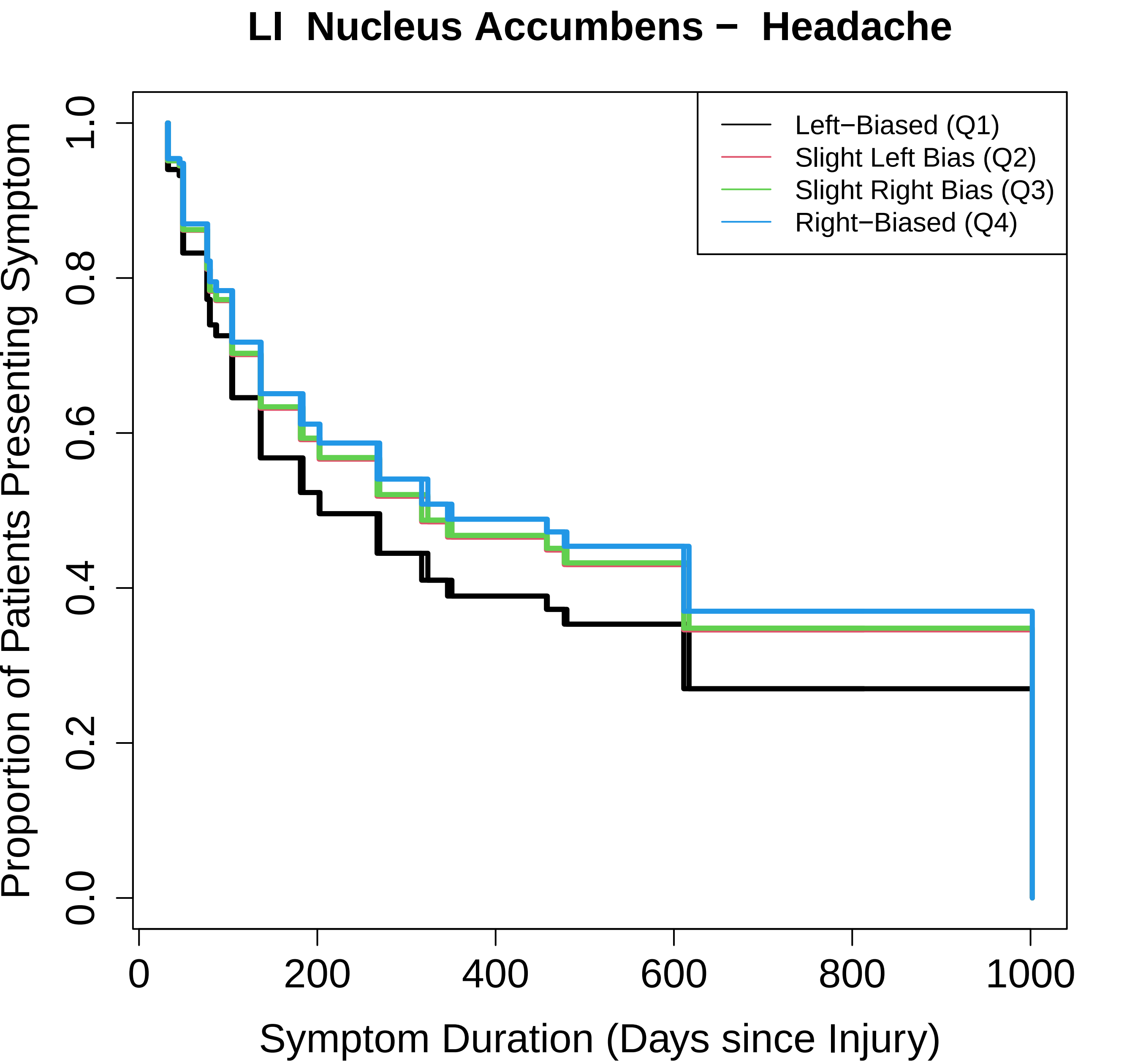
LI Nucleus Accumbens − Headache
Proportion of Patients Presenting Symptom
Symptom Duration (Days since Injury)
0.0
0.2
0.4
0.6
0.8
1.0
0
200
400
600
800
1000
Left−Biased (Q1)
Slight Left Bias (Q2)
Slight Right Bias (Q3)
Right−Biased (Q4)

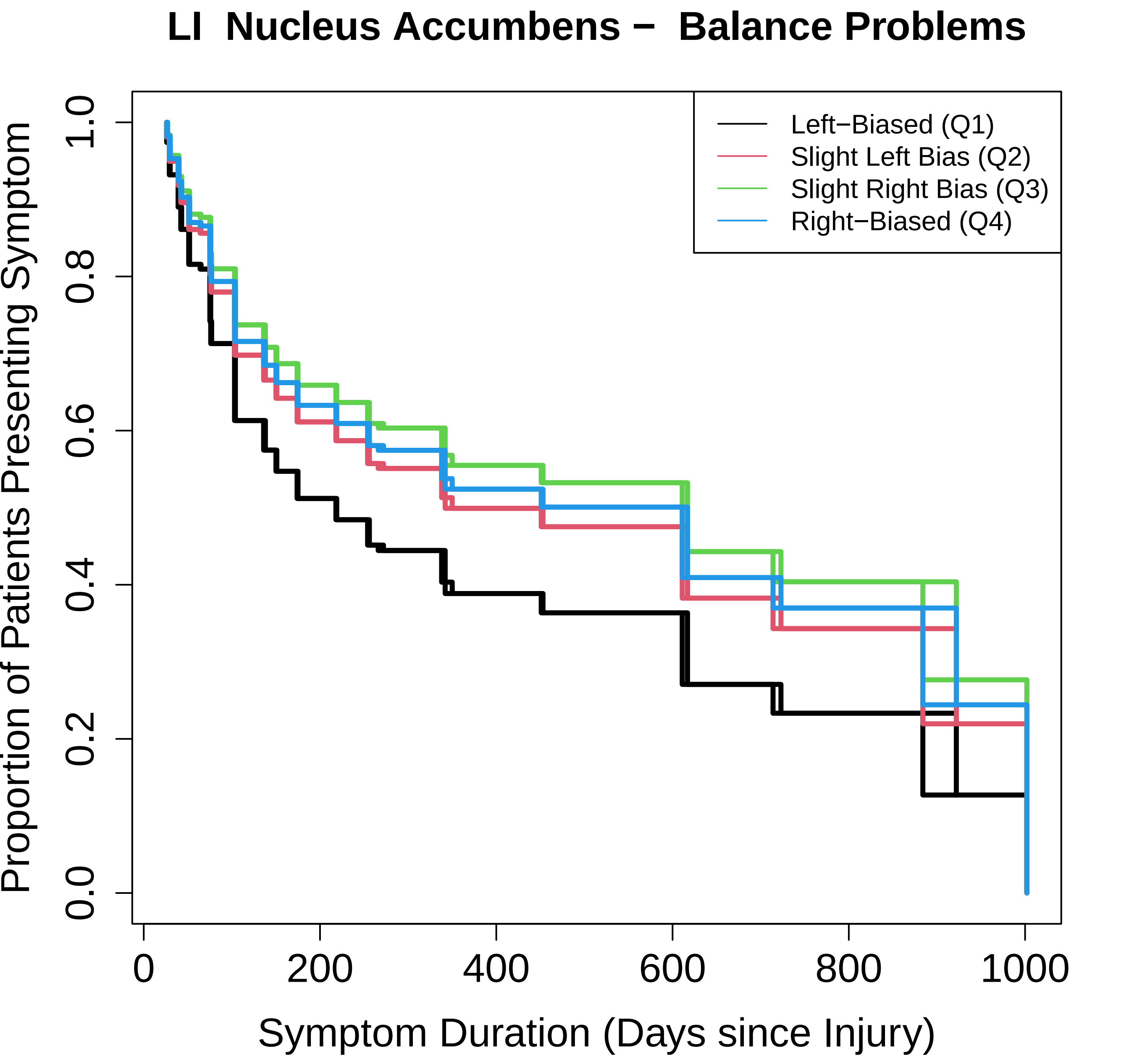

LI Nucleus Accumbens − Balance Problems
Left−Biased (Q1)
Slight Left Bias (Q2)
Slight Right Bias (Q3)
Right−Biased (Q4)
Proportion of Patients Presenting Symptom
0.0
0.2
0.4
0.6
0.8
1.0
0
200
400
600
800
1000
Symptom Duration (Days since Injury)

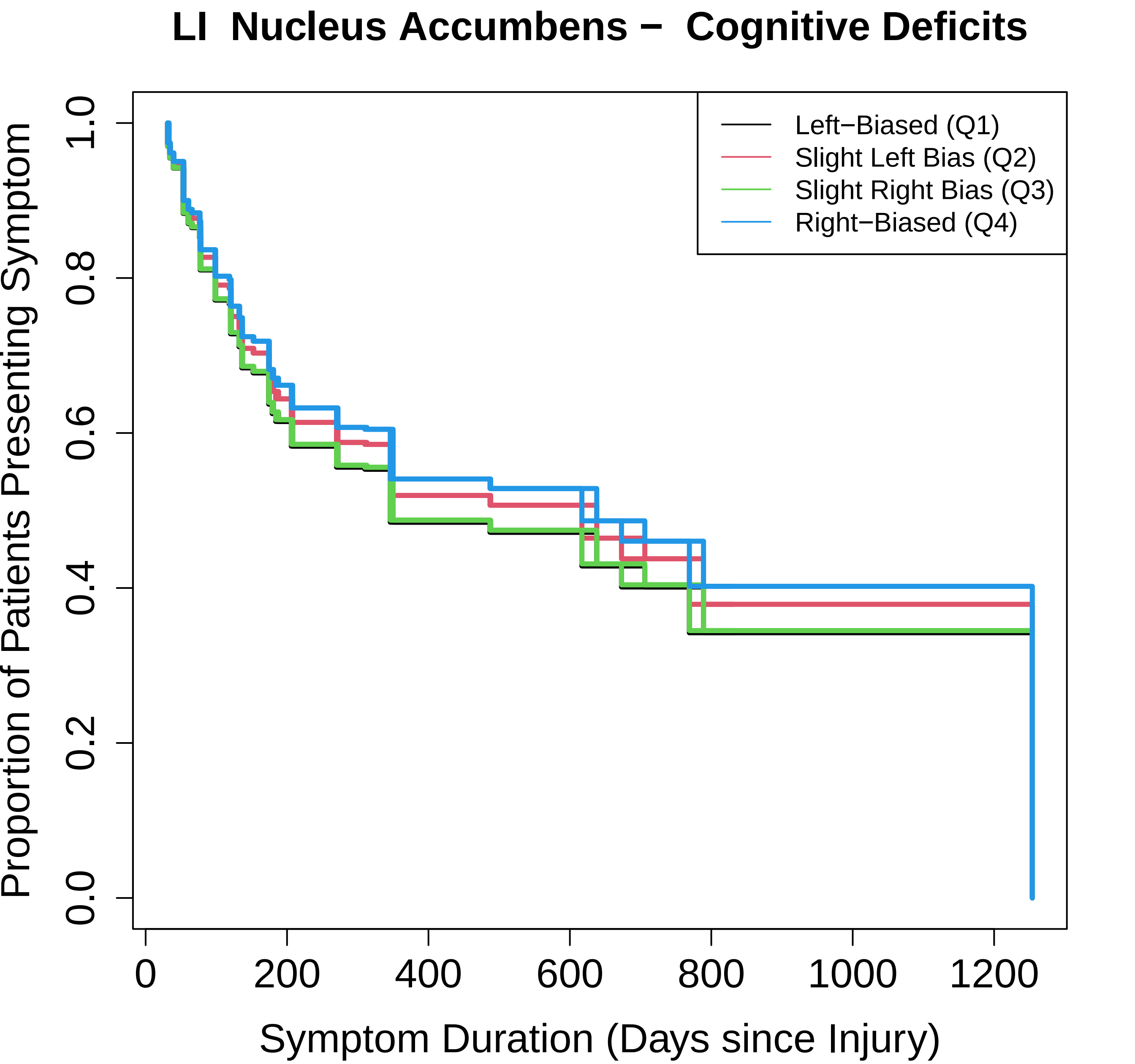

LI Nucleus Accumbens − Cognitive Deficits
Proportion of Patients Presenting Symptom
Symptom Duration (Days since Injury)
0.0
0.2
0.4
0.6
0.8
1.0
0
200
400
600
800
1000
1200
Left−Biased (Q1)
Slight Left Bias (Q2)
Slight Right Bias (Q3)
Right−Biased (Q4)

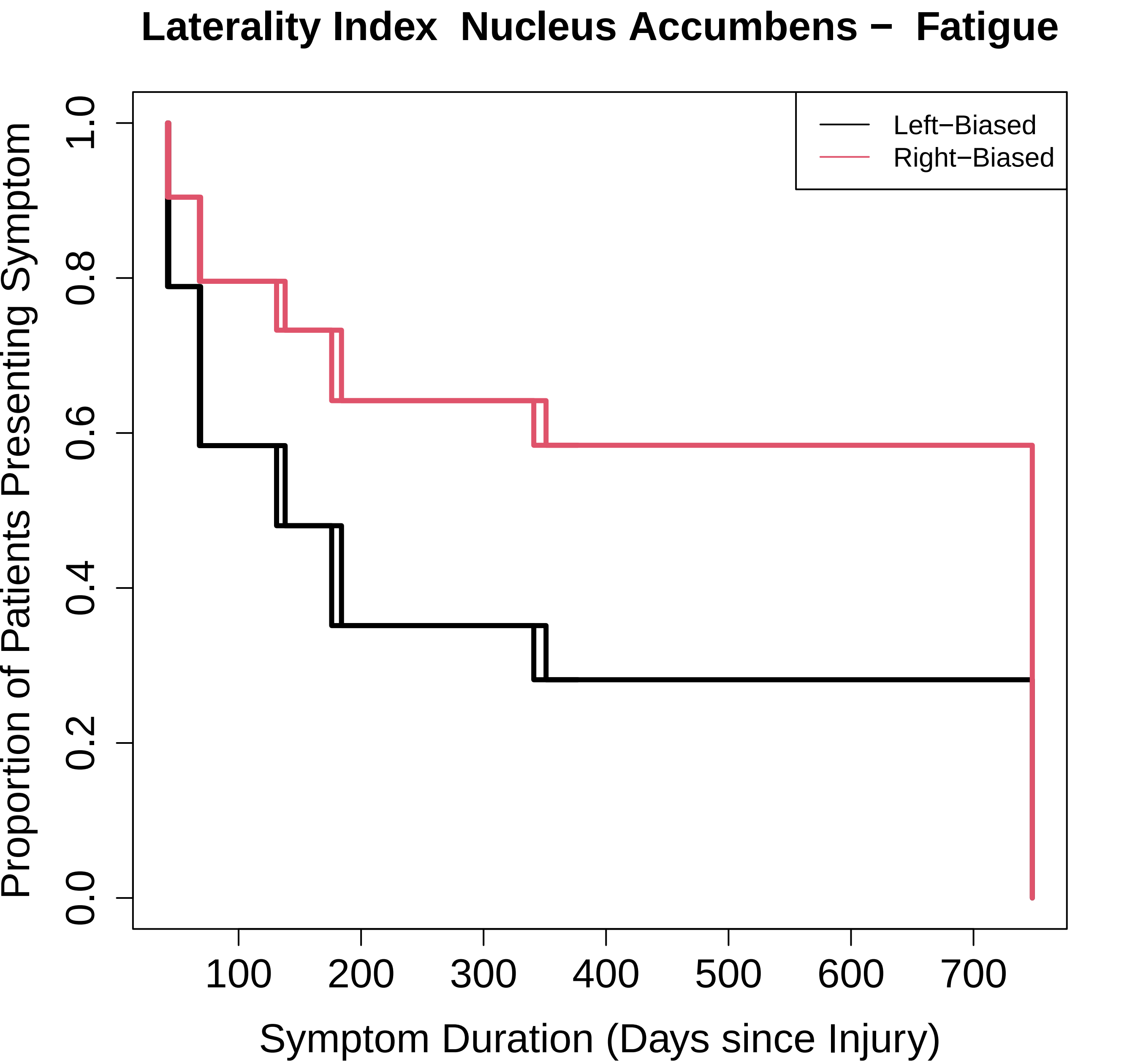

Laterality Index Nucleus Accumbens − Fatigue
Left−Biased
Right−Biased
Proportion of Patients Presenting Symptom
0.0
0.2
0.4
0.6
0.8
1.0
100
200
300
400
500
600
700
Symptom Duration (Days since Injury)

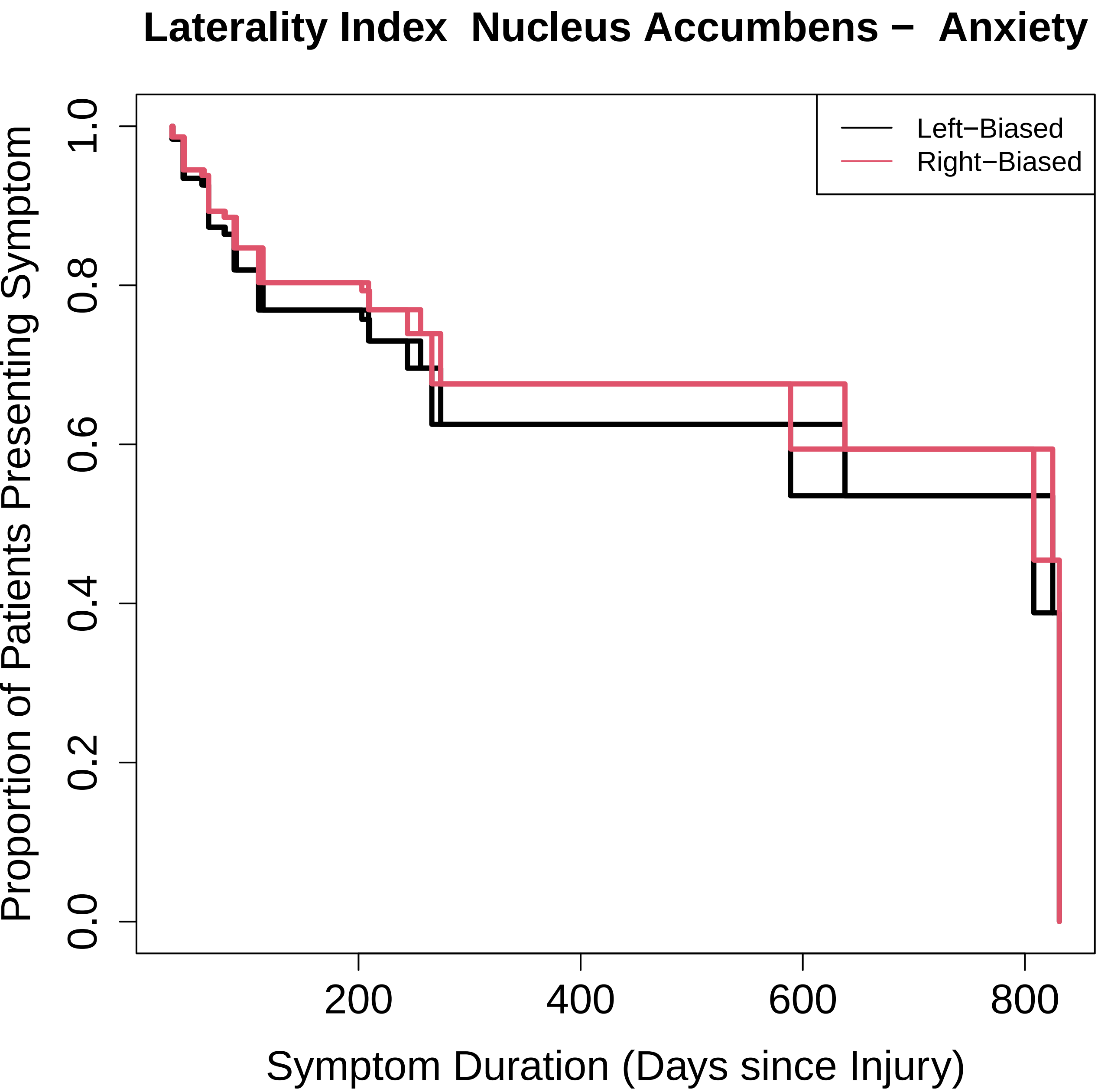

Laterality Index Nucleus Accumbens − Anxiety
Left−Biased
Right−Biased
Proportion of Patients Presenting Symptom
0.0
0.2
0.4
0.6
0.8
1.0
200
400
600
800
Symptom Duration (Days since Injury)

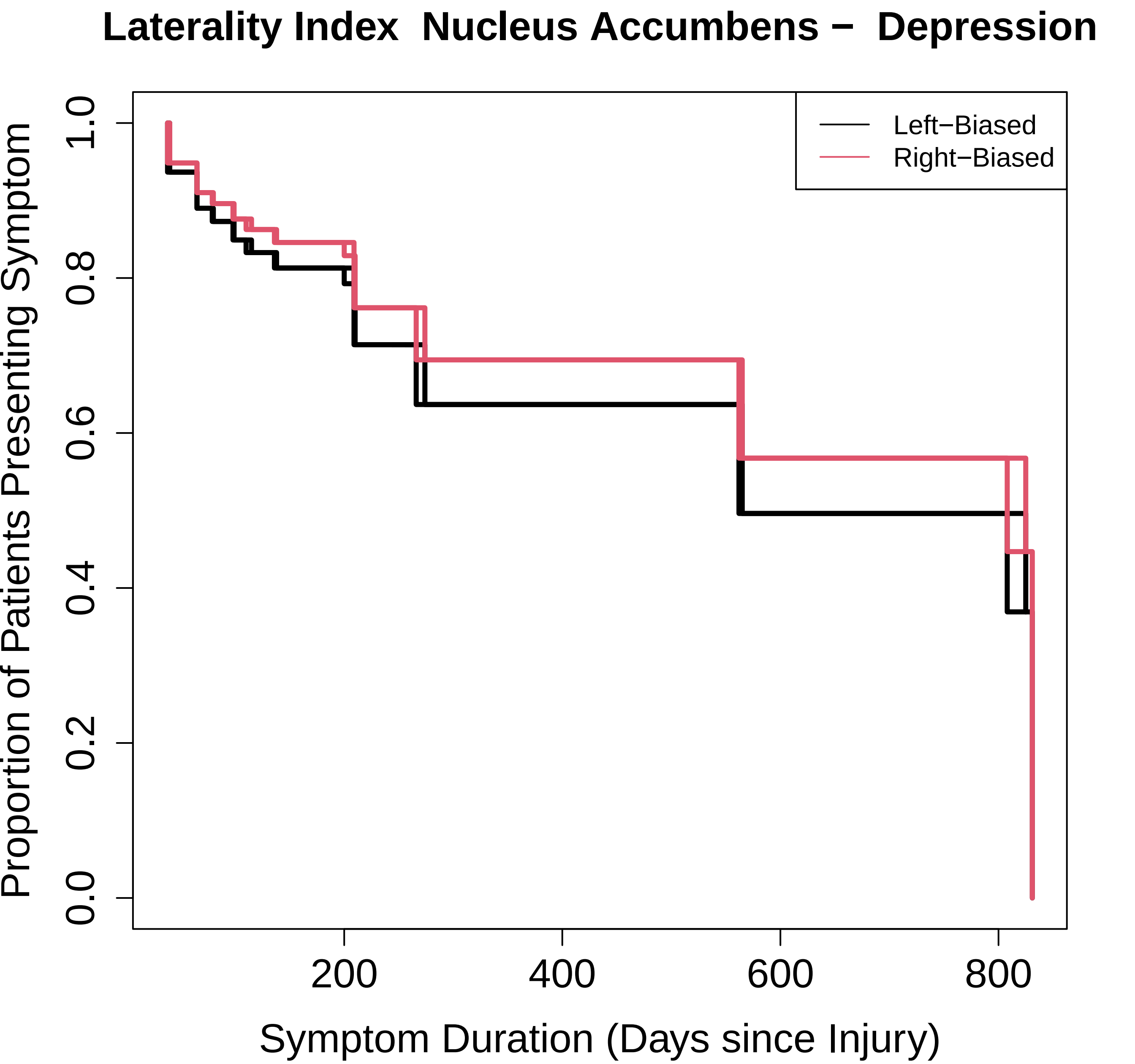
Laterality Index Nucleus Accumbens − Depression
Left−Biased
Right−Biased
Proportion of Patients Presenting Symptom
0.0
0.2
0.4
0.6
0.8
1.0
200
400
600
800
Symptom Duration (Days since Injury)

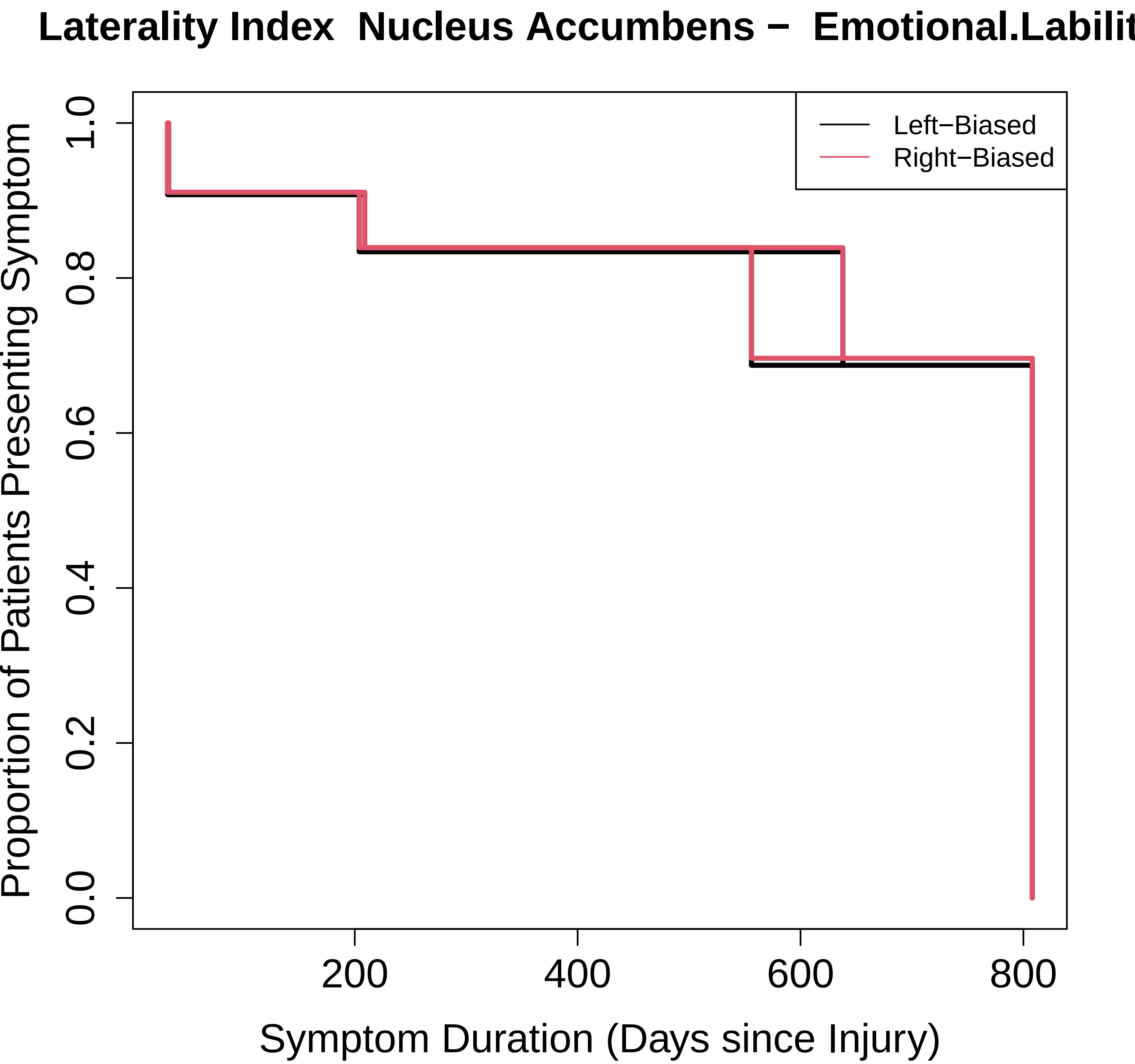

Laterality Index Nucleus Accumbens − Emotional.Labilit
Left−Biased
Right−Biased
Proportion of Patients Presenting Symptom
0.0
0.2
0.4
0.6
0.8
1.0
200
400
600
800
Symptom Duration (Days since Injury)

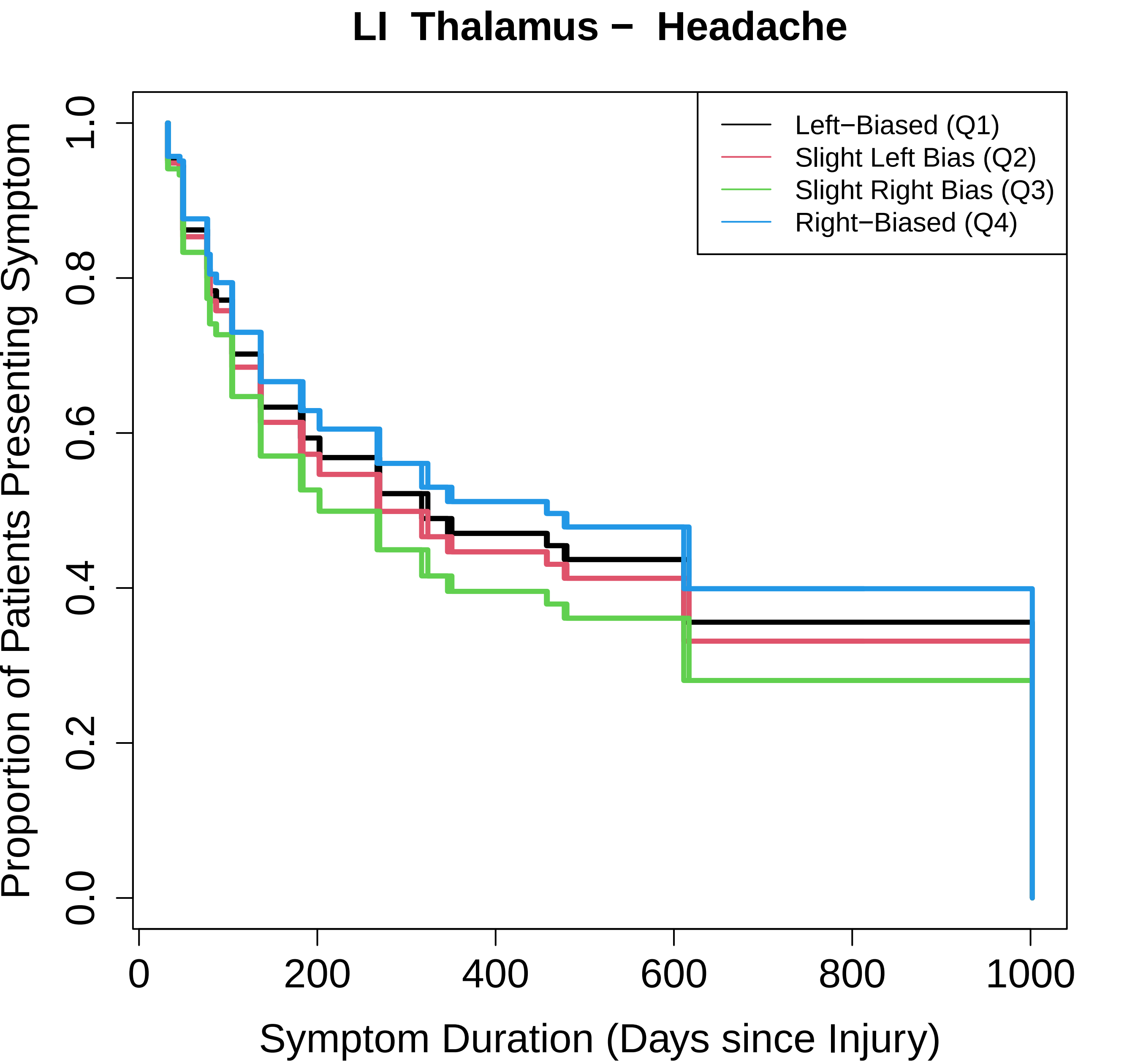
LI Thalamus − Headache
Left−Biased (Q1)
Slight Left Bias (Q2)
Slight Right Bias (Q3)
Right−Biased (Q4)
Proportion of Patients Presenting Symptom
0.0
0.2
0.4
0.6
0.8
1.0
0
200
400
600
800
1000
Symptom Duration (Days since Injury)

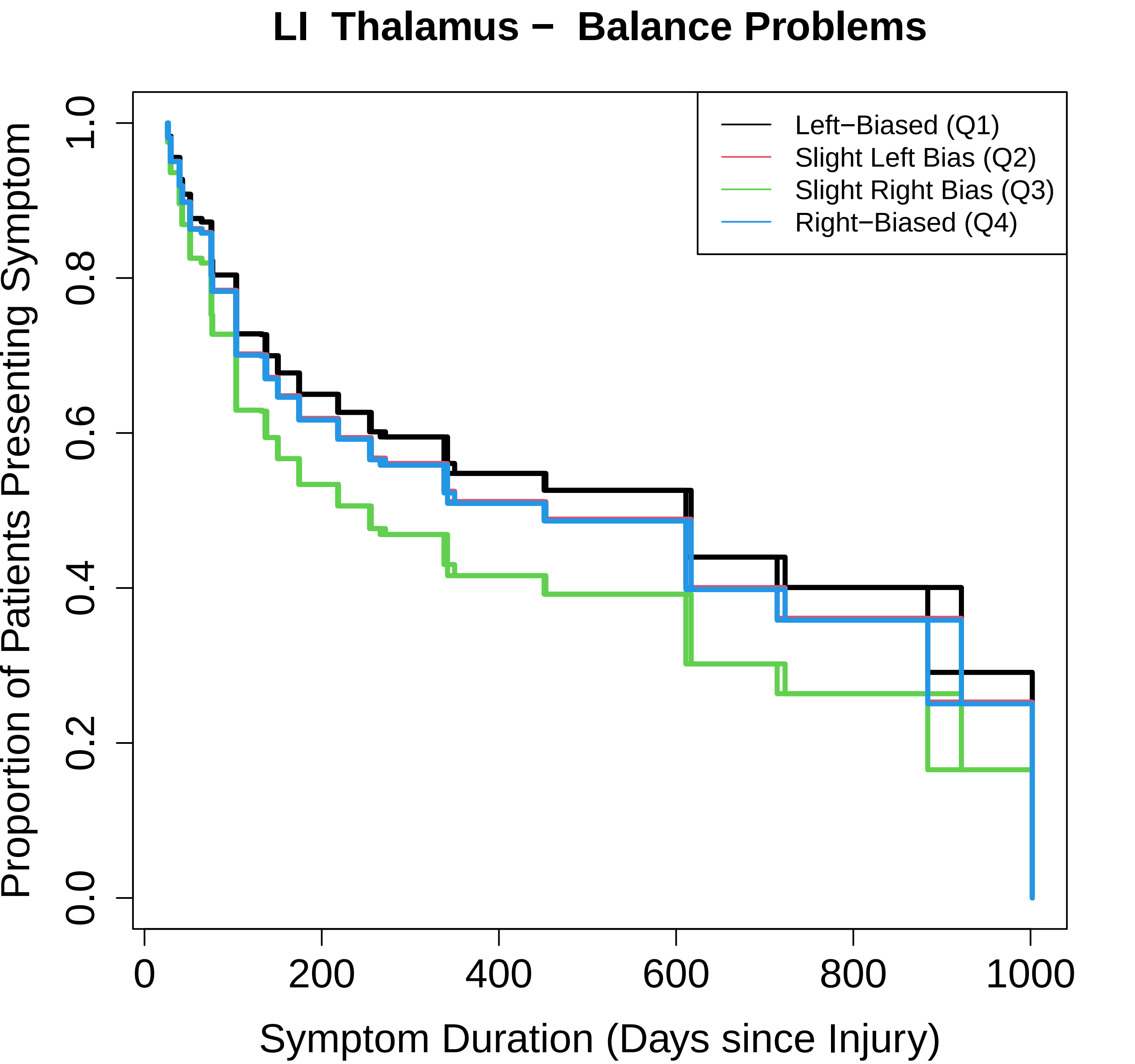
LI Thalamus − Balance Problems
Left−Biased (Q1)
Slight Left Bias (Q2)
Slight Right Bias (Q3)
Right−Biased (Q4)
Proportion of Patients Presenting Symptom
0.0
0.2
0.4
0.6
0.8
1.0
0
200
400
600
800
1000
Symptom Duration (Days since Injury)

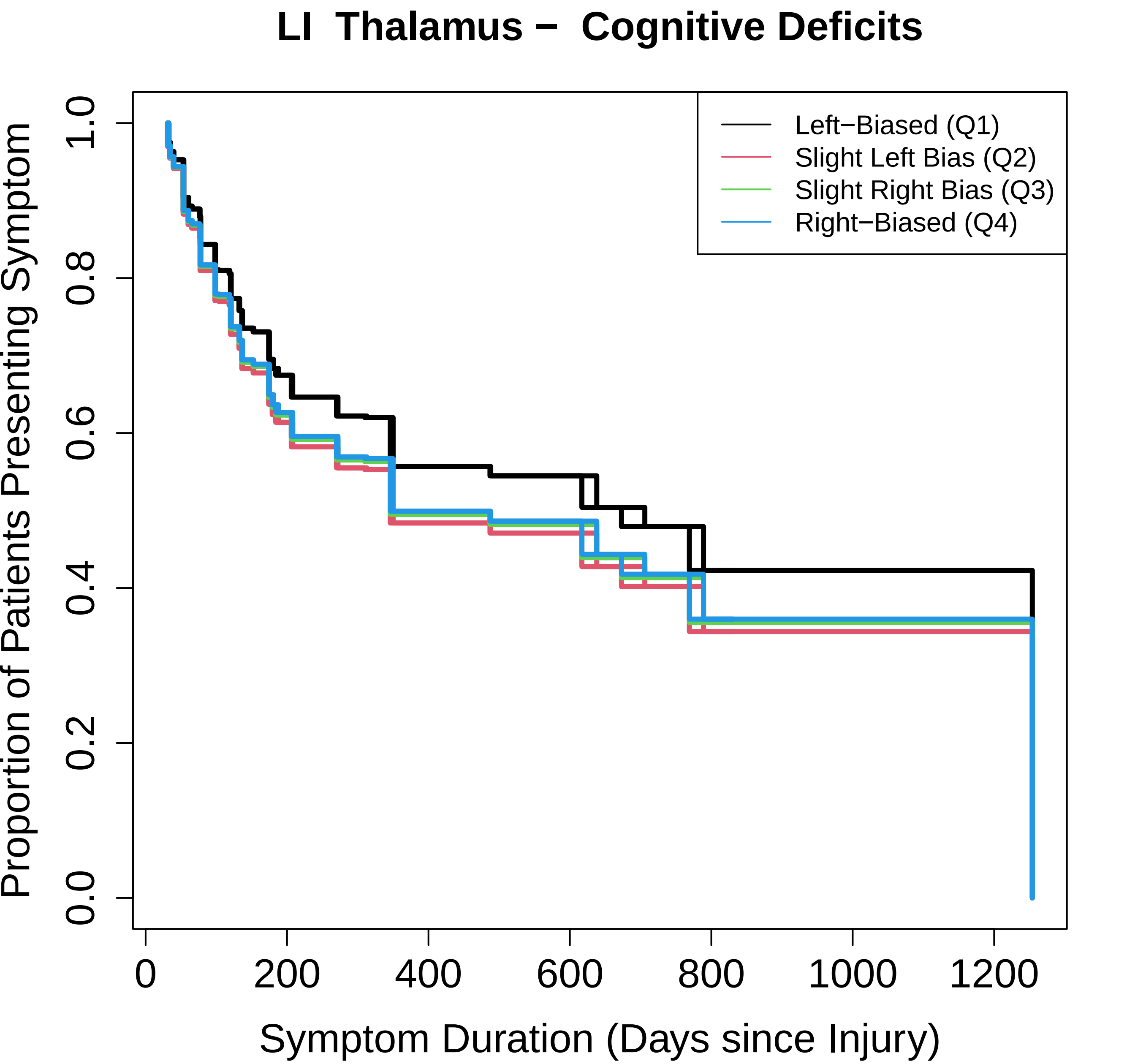

LI Thalamus − Cognitive Deficits
Left−Biased (Q1)
Slight Left Bias (Q2)
Slight Right Bias (Q3)
Right−Biased (Q4)
Proportion of Patients Presenting Symptom
0.0
0.2
0.4
0.6
0.8
1.0
0
200
400
600
800
1000
1200
Symptom Duration (Days since Injury)

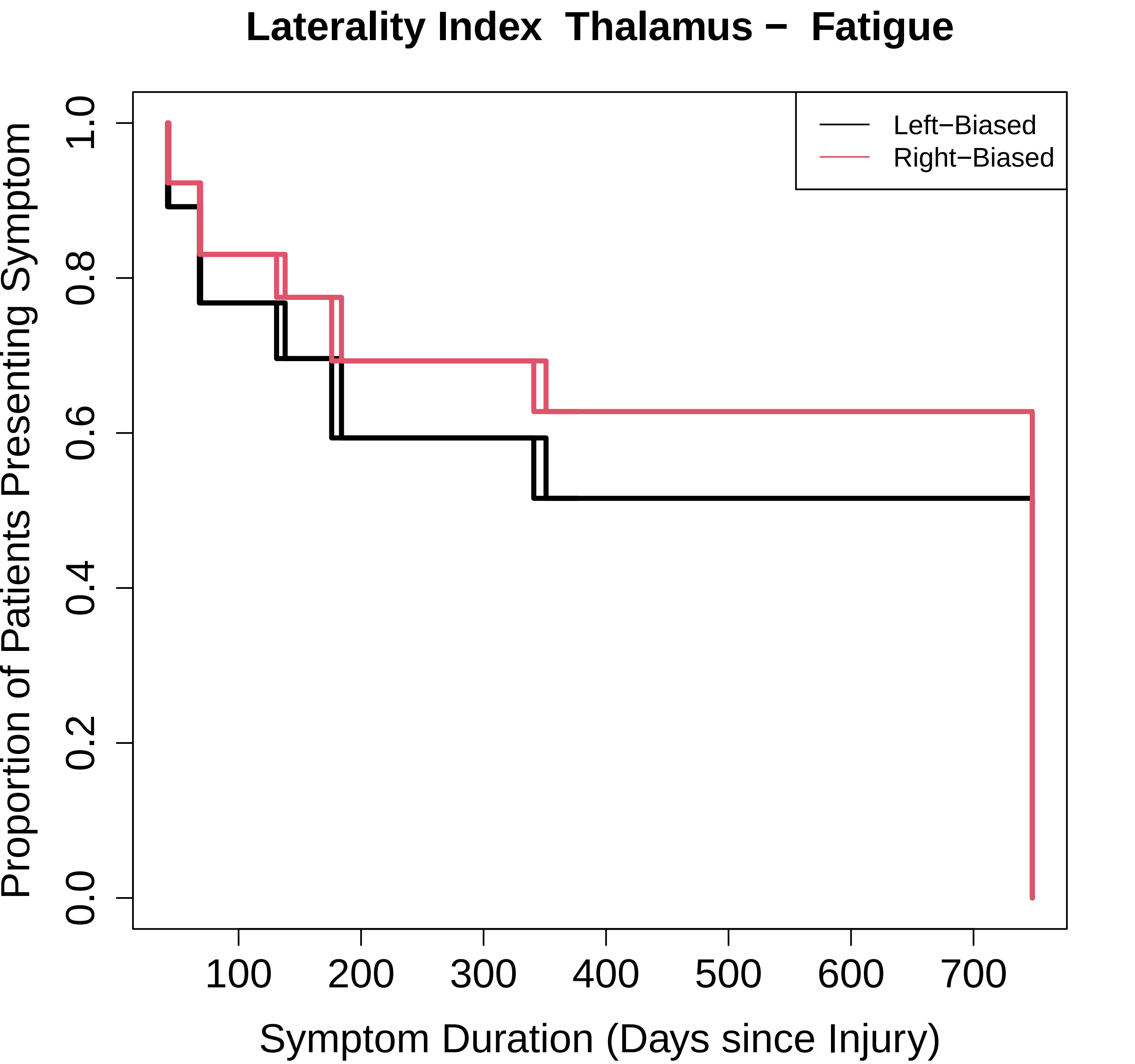

Laterality Index Thalamus − Fatigue
Left−Biased
Right−Biased
Proportion of Patients Presenting Symptom
0.0
0.2
0.4
0.6
0.8
1.0
100
200
300
400
500
600
700
Symptom Duration (Days since Injury)

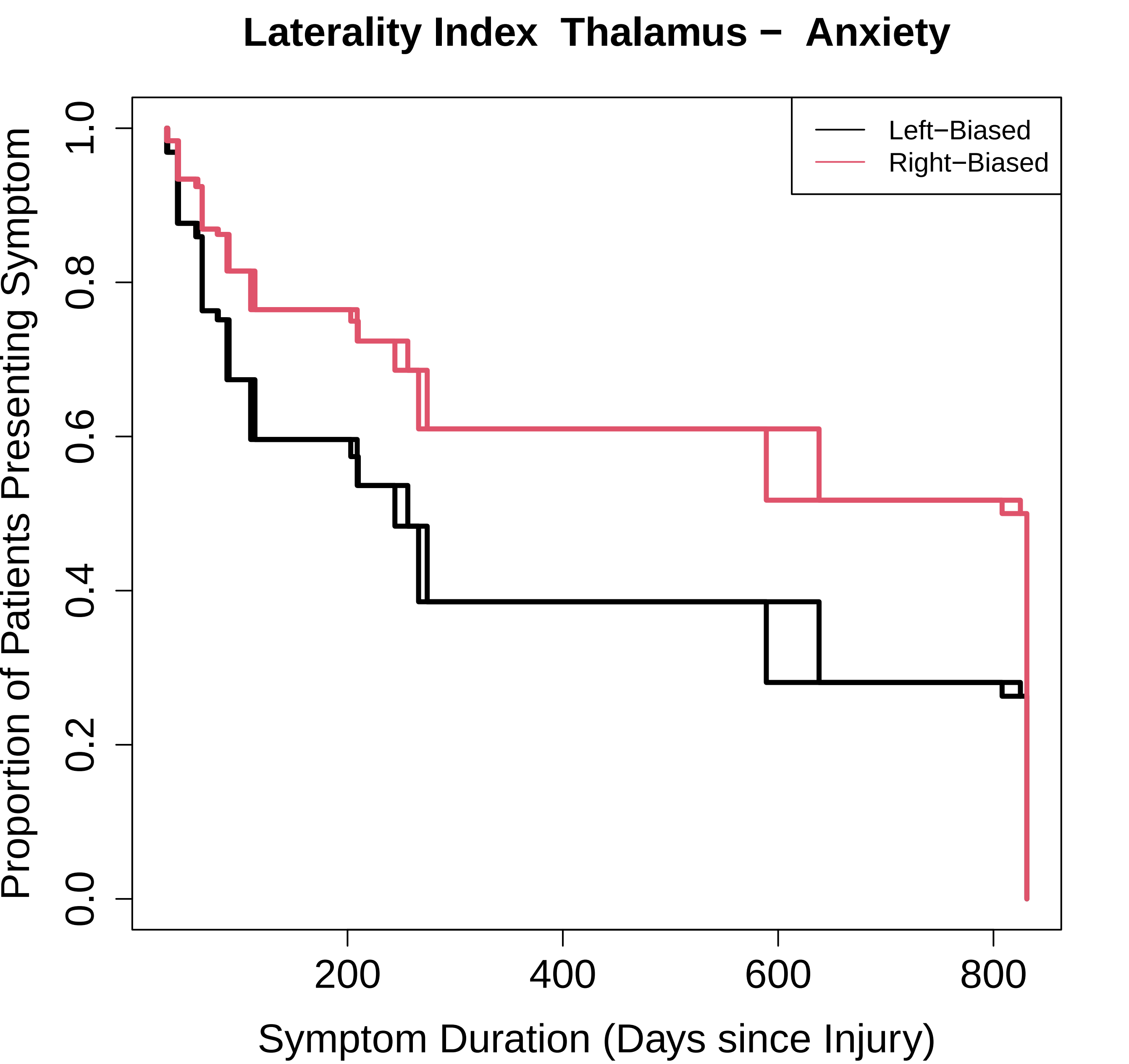
Laterality Index Thalamus − Anxiety
Left−Biased
Right−Biased
Proportion of Patients Presenting Symptom
0.0
0.2
0.4
0.6
0.8
1.0
200
400
600
800
Symptom Duration (Days since Injury)

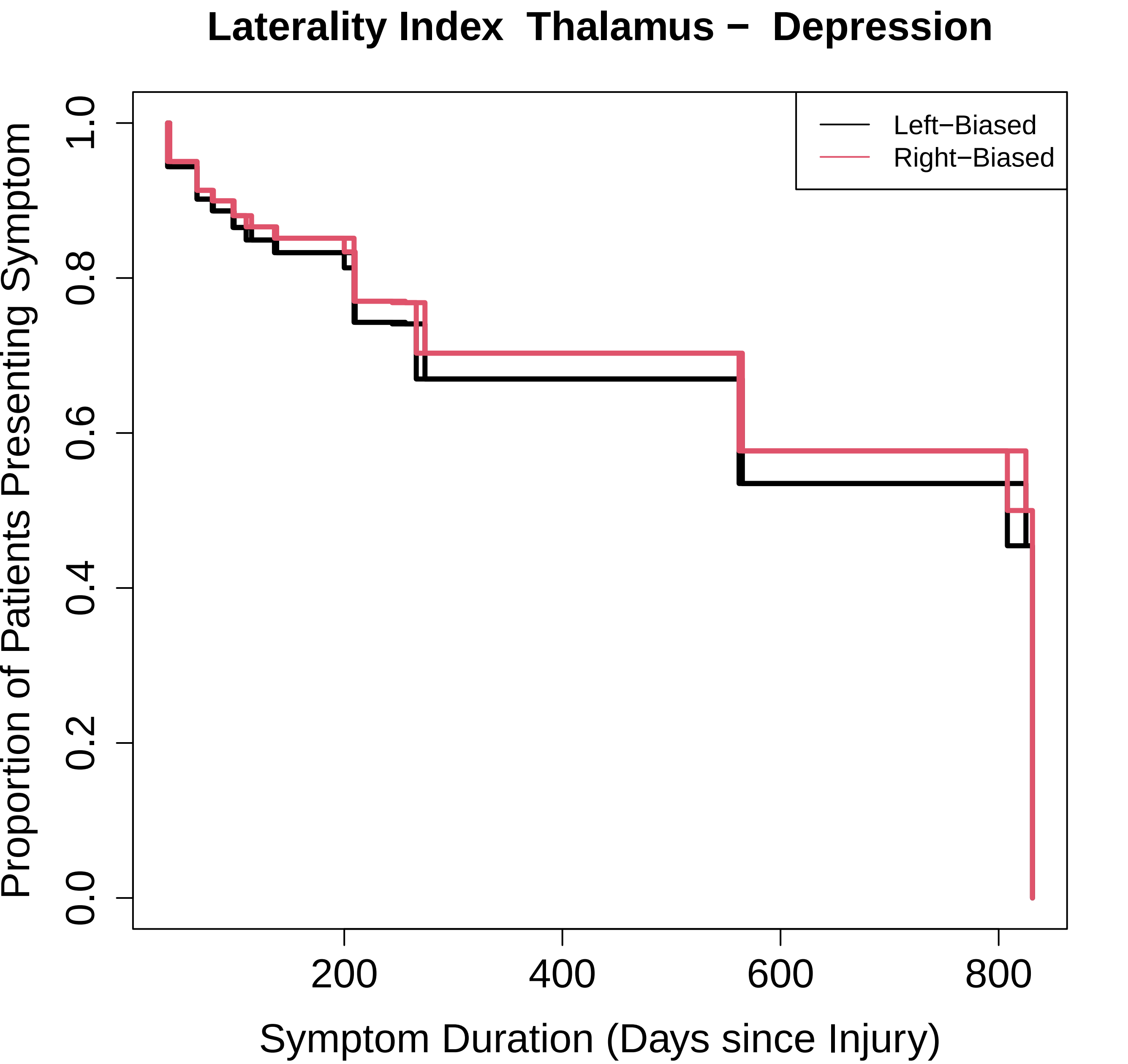

Laterality Index Thalamus − Depression
Left−Biased
Right−Biased
Proportion of Patients Presenting Symptom
0.0
0.2
0.4
0.6
0.8
1.0
200
400
600
800
Symptom Duration (Days since Injury)

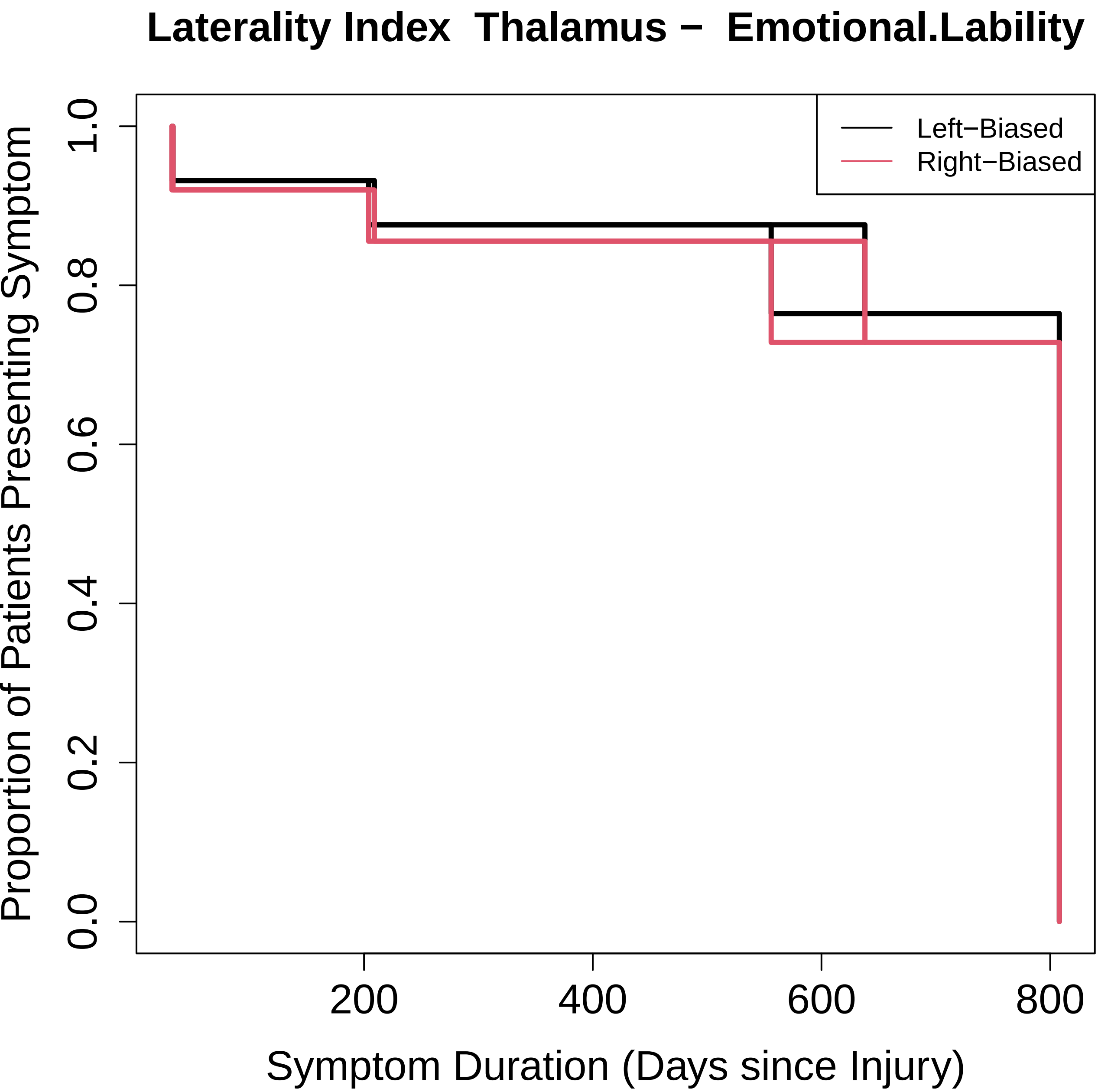

Laterality Index Thalamus − Emotional.Lability
Left−Biased
Right−Biased
Proportion of Patients Presenting Symptom
0.0
0.2
0.4
0.6
0.8
1.0
200
400
600
800
Symptom Duration (Days since Injury)
