# Supplementary material for: Symptom Persistence Relates to Volume and Asymmetry of the Limbic System after Mild Traumatic Brain Injury
Source: J Clin Med. 2024 Aug 30;13(17):5154. doi: 10.3390/jcm13175154 (PMC11396354; doi:10.3390/jcm13175154)
Supplement: Supplementary file 1 [file jcm-13-05154-s001.zip › Figure S3 Limbic siLI clinical symptoms graphs.pdf]

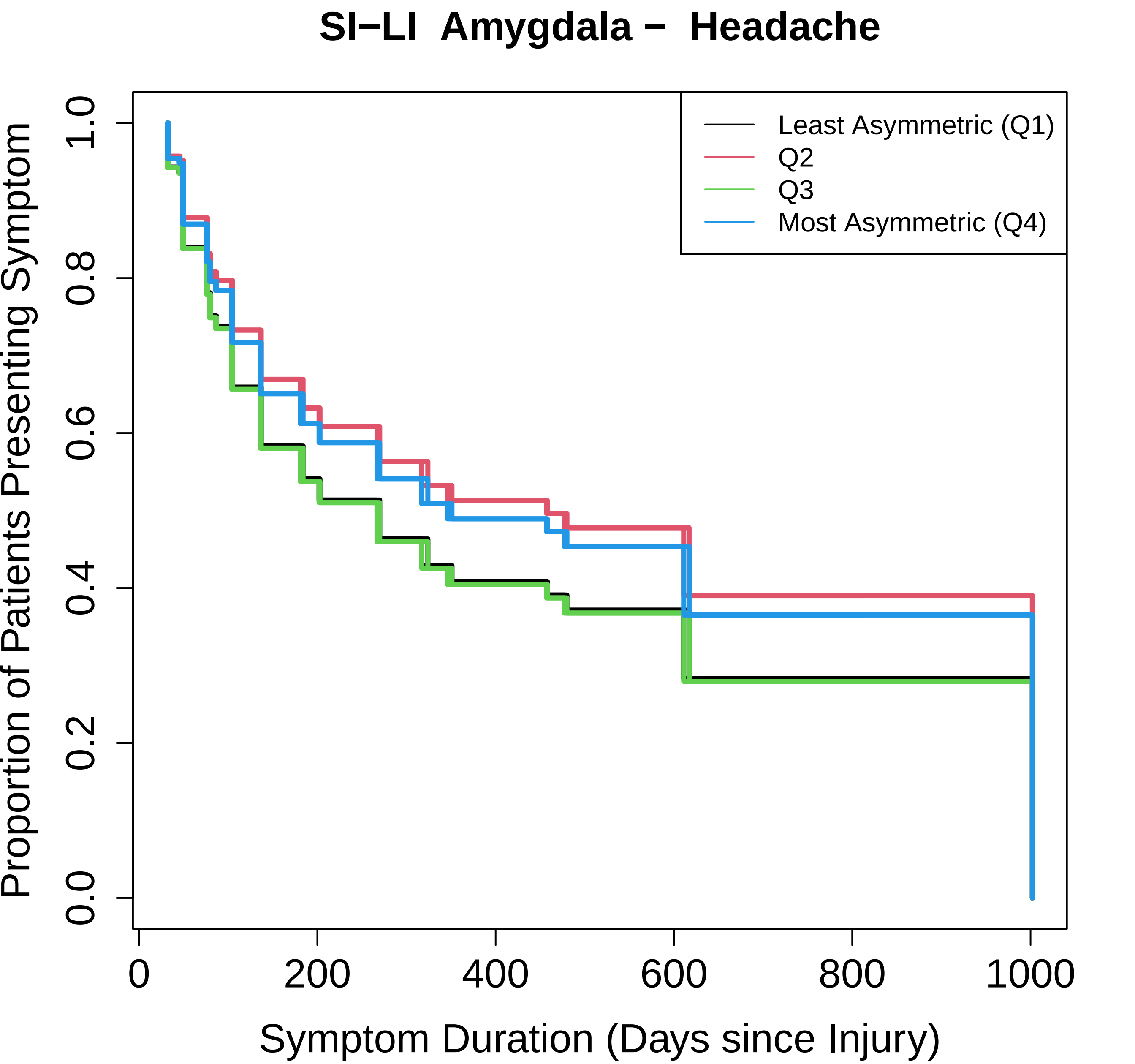
SI−LI Amygdala − Headache
Proportion of Patients Presenting Symptom
Symptom Duration (Days since Injury)
Least Asymmetric (Q1)
Q2
Q3
Most Asymmetric (Q4)
0.0
0.2
0.4
0.6
0.8
1.0
0
200
400
600
800
1000

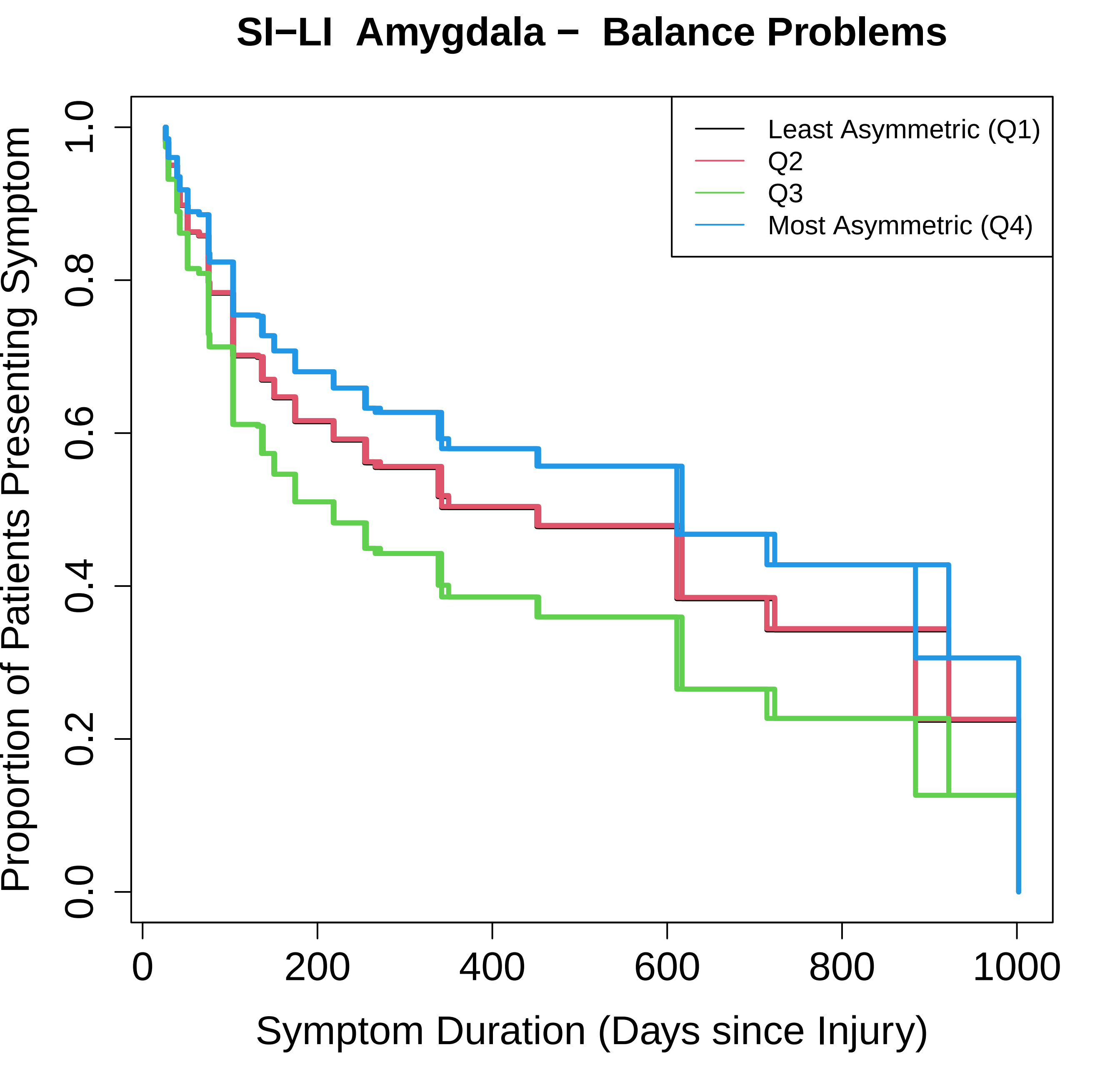
SI−LI Amygdala − Balance Problems
Least Asymmetric (Q1)
Q2
Q3
Most Asymmetric (Q4)
Proportion of Patients Presenting Symptom
0.0
0.2
0.4
0.6
0.8
1.0
0
200
400
600
800
1000
Symptom Duration (Days since Injury)

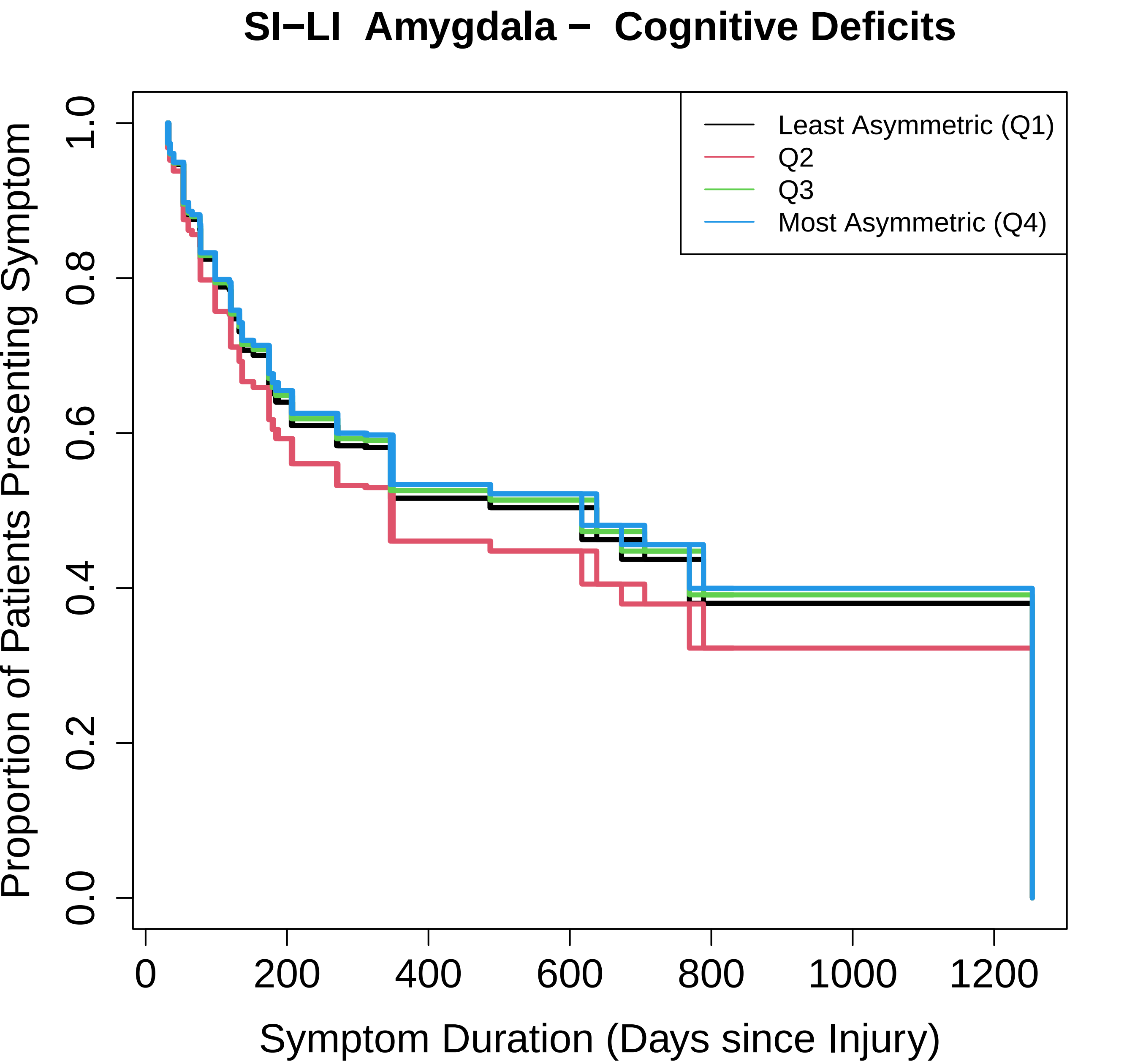

SI−LI Amygdala − Cognitive Deficits
Least Asymmetric (Q1)
Q2
Q3
Most Asymmetric (Q4)
Proportion of Patients Presenting Symptom
0.0
0.2
0.4
0.6
0.8
1.0
0
200
400
600
800
1000
1200
Symptom Duration (Days since Injury)

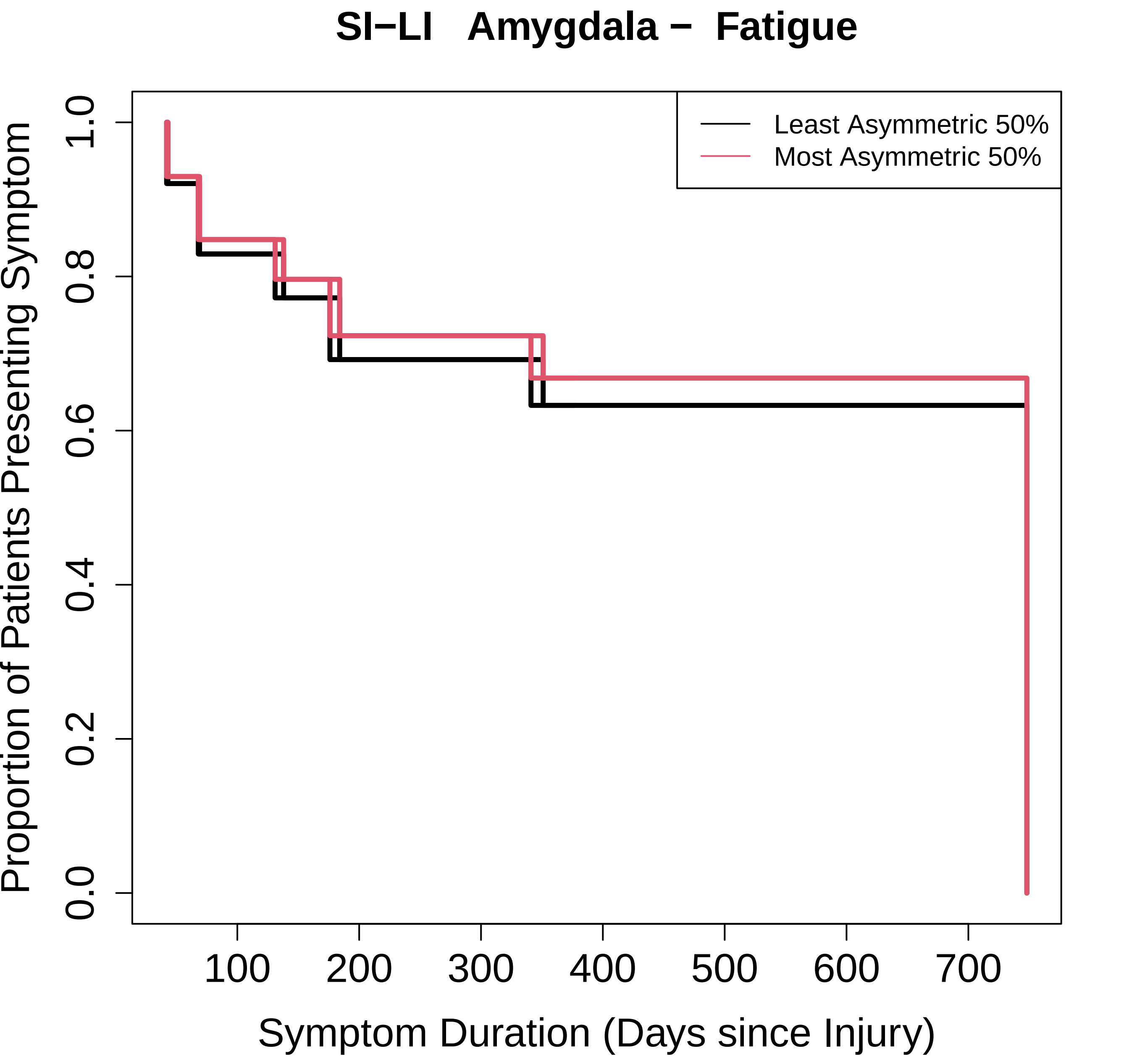
SI−LI Amygdala − Fatigue
Least Asymmetric 50%
Most Asymmetric 50%
Proportion of Patients Presenting Symptom
0.0
0.2
0.4
0.6
0.8
1.0
100
200
300
400
500
600
700
Symptom Duration (Days since Injury)

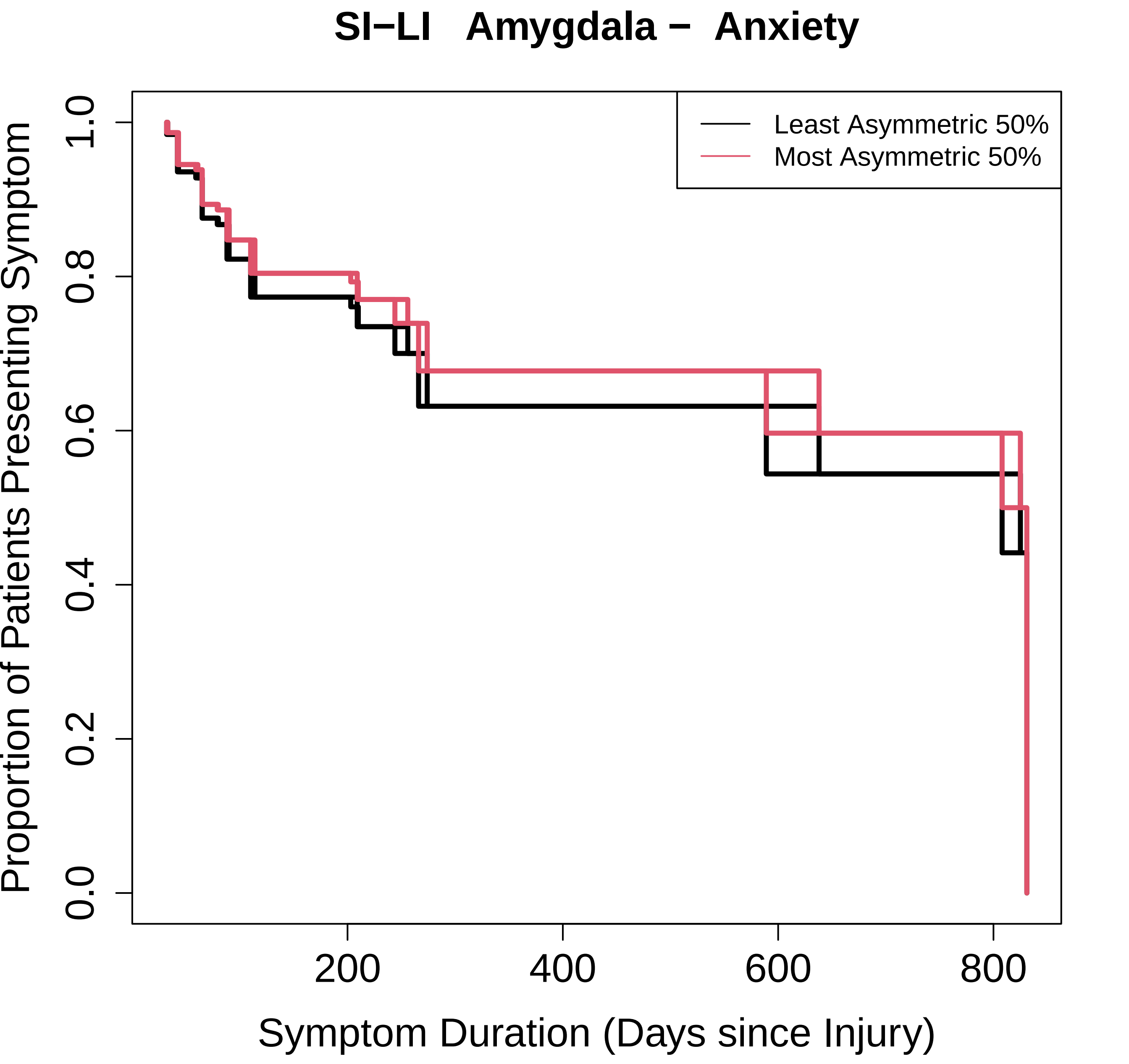
SI−LI Amygdala − Anxiety
Least Asymmetric 50%
Most Asymmetric 50%
Proportion of Patients Presenting Symptom
0.0
0.2
0.4
0.6
0.8
1.0
200
400
600
800
Symptom Duration (Days since Injury)

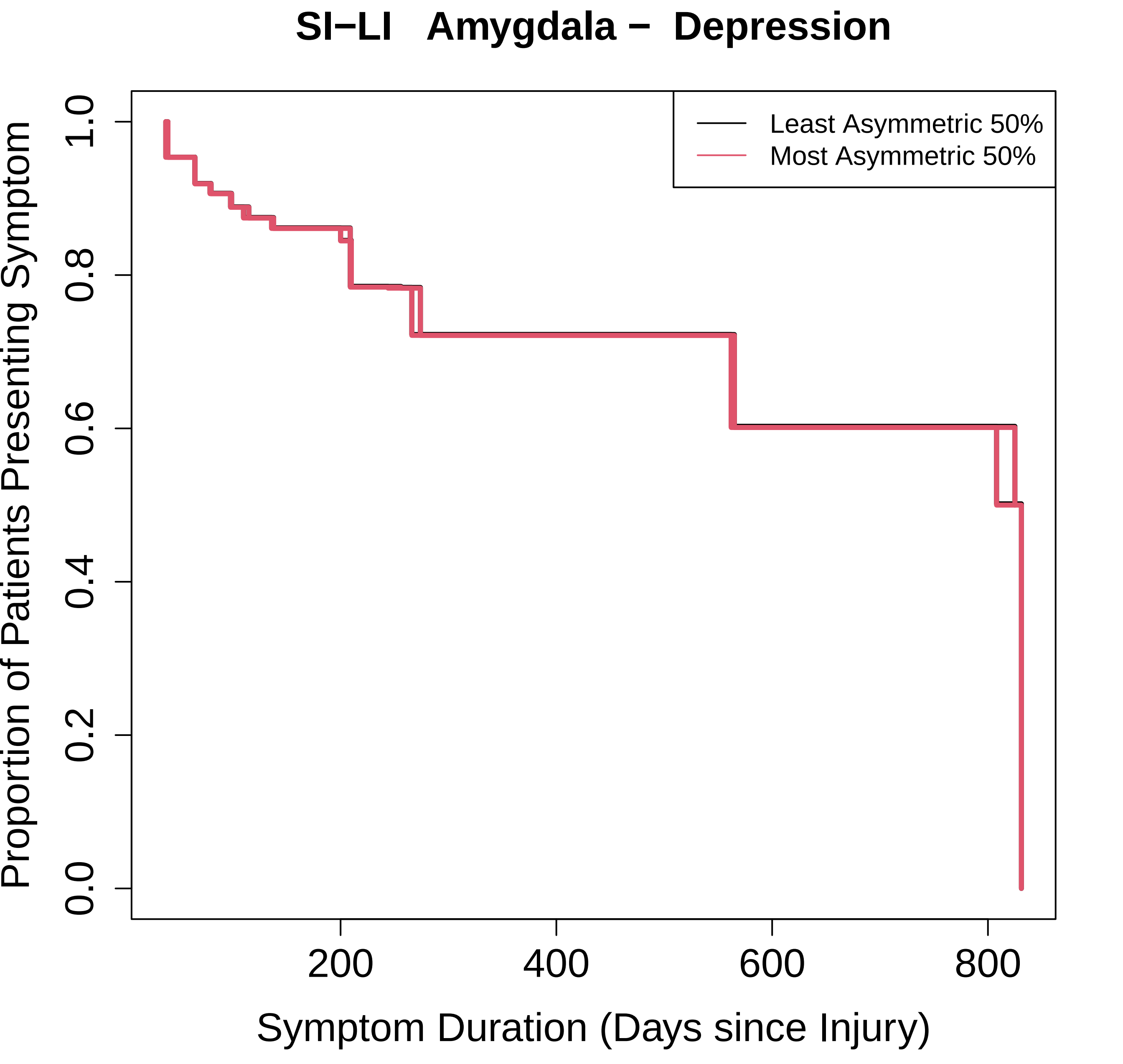
SI−LI Amygdala − Depression
Least Asymmetric 50%
Most Asymmetric 50%
Proportion of Patients Presenting Symptom
0.0
0.2
0.4
0.6
0.8
1.0
200
400
600
800
Symptom Duration (Days since Injury)

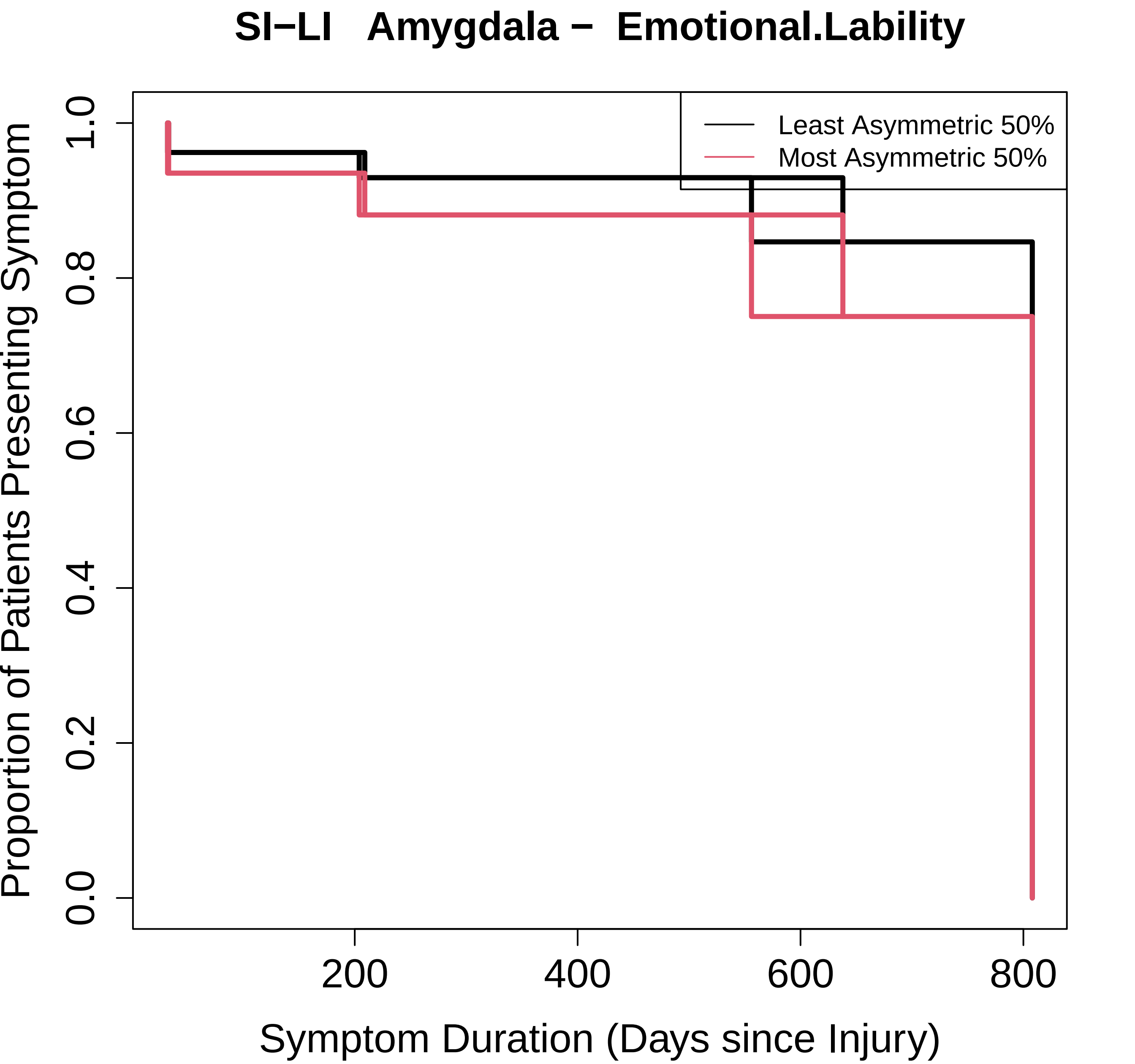

SI−LI Amygdala − Emotional.Lability
Least Asymmetric 50%
Most Asymmetric 50%
Proportion of Patients Presenting Symptom
0.0
0.2
0.4
0.6
0.8
1.0
200
400
600
800
Symptom Duration (Days since Injury)

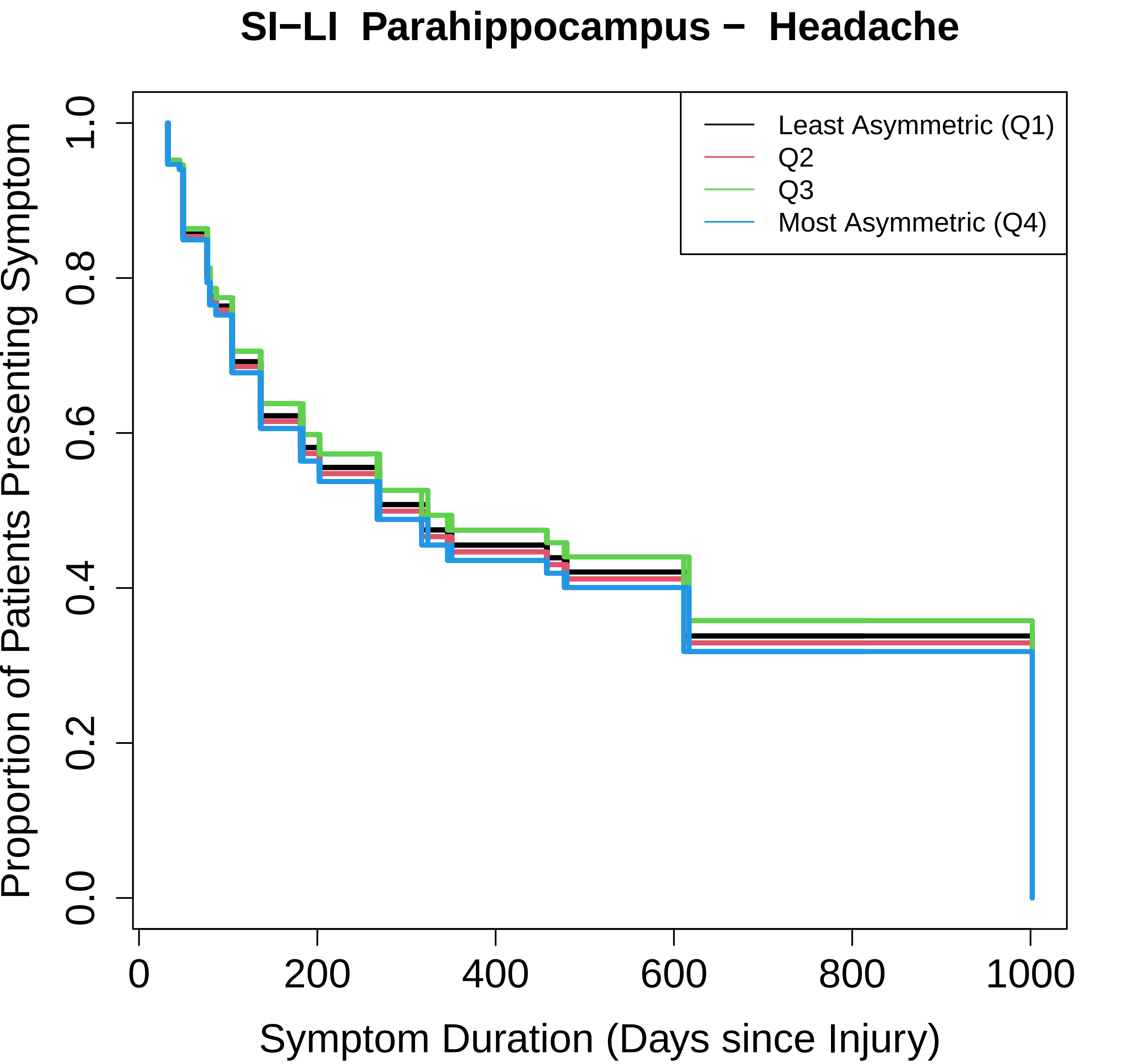

SI−LI Parahippocampus − Headache
Least Asymmetric (Q1)
Q2
Q3
Most Asymmetric (Q4)
Proportion of Patients Presenting Symptom
0.0
0.2
0.4
0.6
0.8
1.0
0
200
400
600
800
1000
Symptom Duration (Days since Injury)

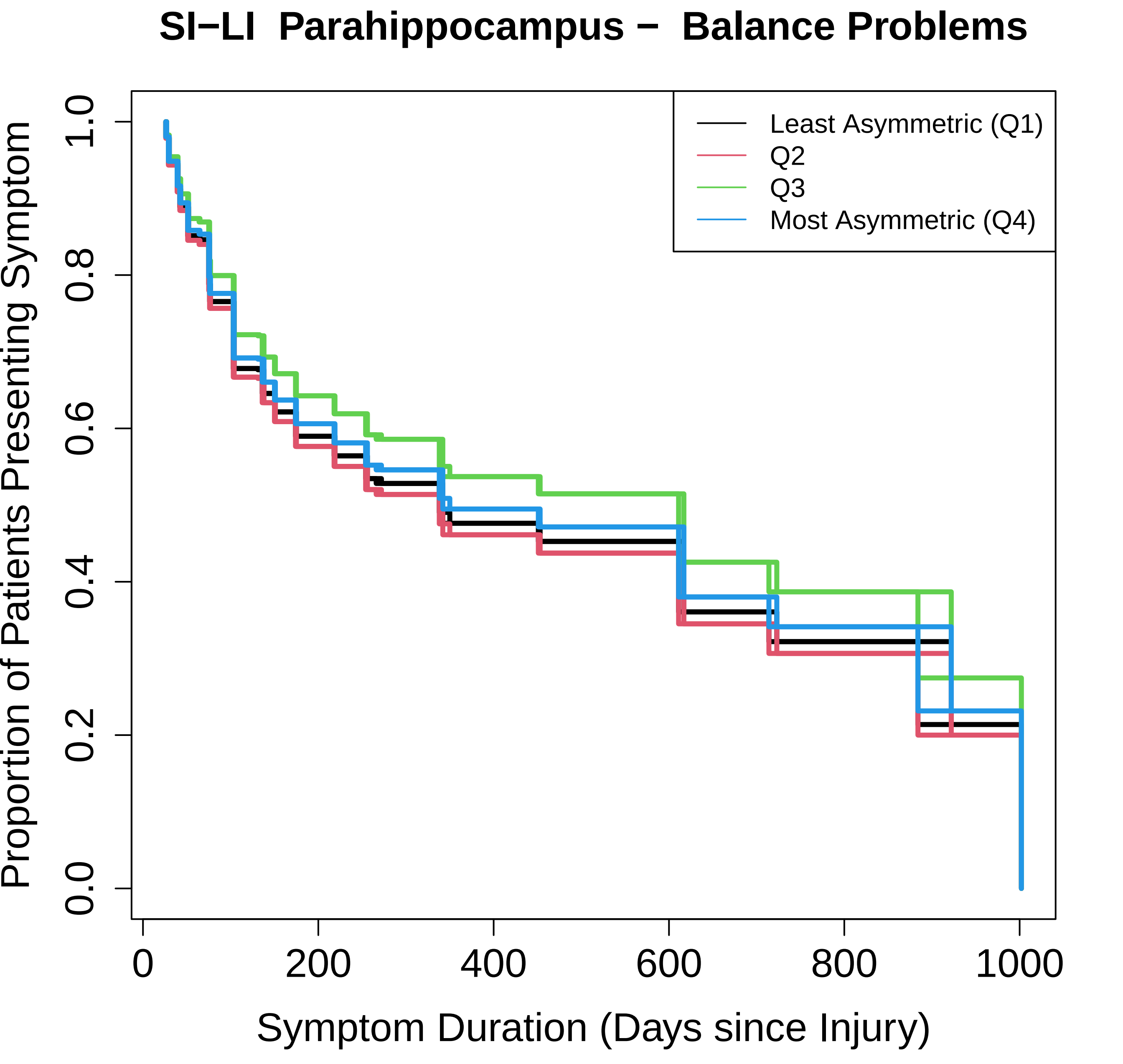
SI−LI Parahippocampus − Balance Problems
Least Asymmetric (Q1)
Q2
Q3
Most Asymmetric (Q4)
Proportion of Patients Presenting Symptom
0.0
0.2
0.4
0.6
0.8
1.0
0
200
400
600
800
1000
Symptom Duration (Days since Injury)

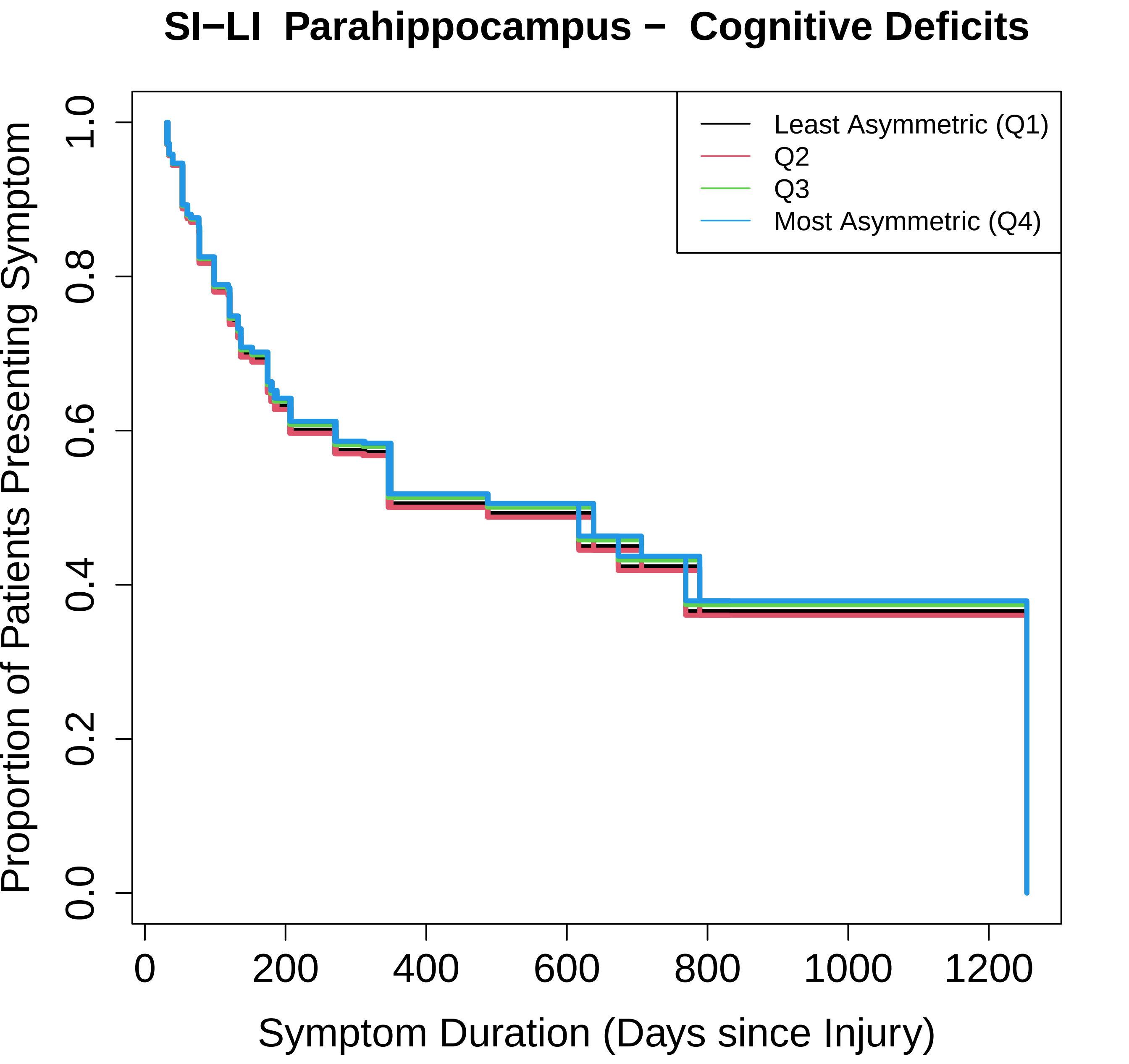
SI−LI Parahippocampus − Cognitive Deficits
Least Asymmetric (Q1)
Q2
Q3
Most Asymmetric (Q4)
Proportion of Patients Presenting Symptom
0.0
0.2
0.4
0.6
0.8
1.0
0
200
400
600
800
1000
1200
Symptom Duration (Days since Injury)

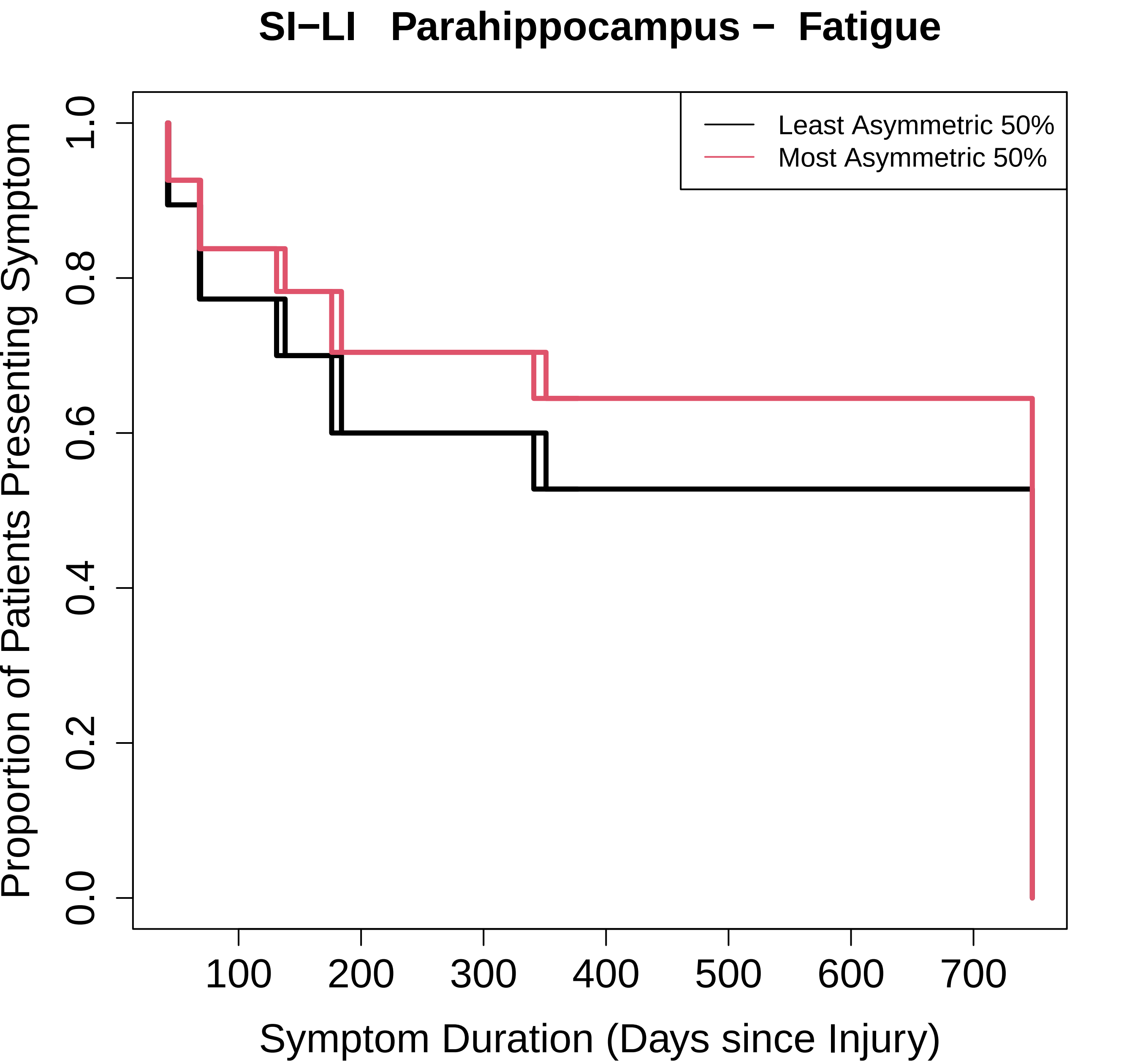

SI−LI Parahippocampus − Fatigue
Least Asymmetric 50%
Most Asymmetric 50%
1.0
0.8
0.6
0.4
0.2
0.0
Proportion of Patients Presenting Symptom
100
200
300
400
500
600
700
Symptom Duration (Days since Injury)

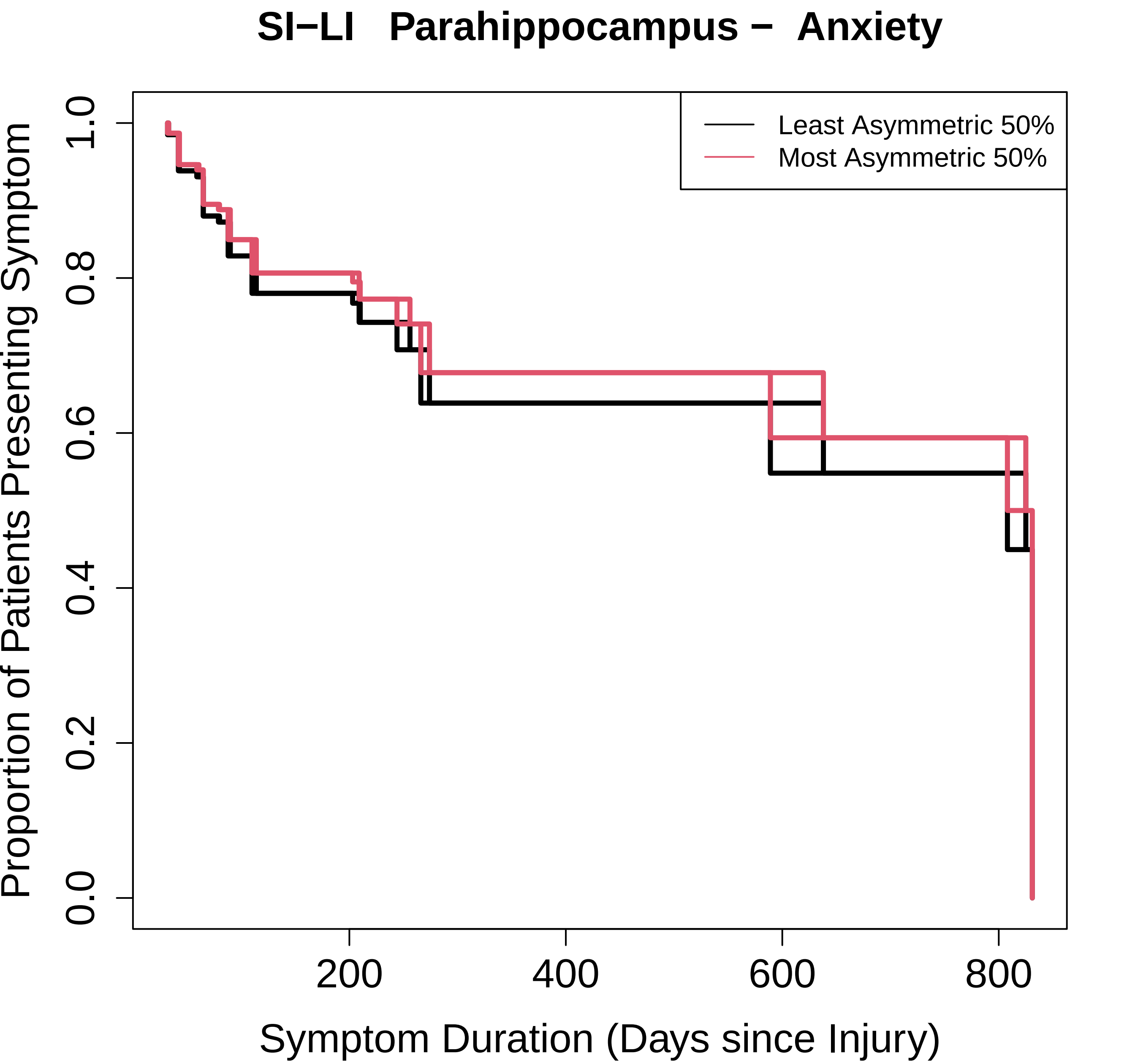
SI−LI Parahippocampus − Anxiety
Least Asymmetric 50%
Most Asymmetric 50%
Proportion of Patients Presenting Symptom
0.0
0.2
0.4
0.6
0.8
1.0
200
400
600
800
Symptom Duration (Days since Injury)

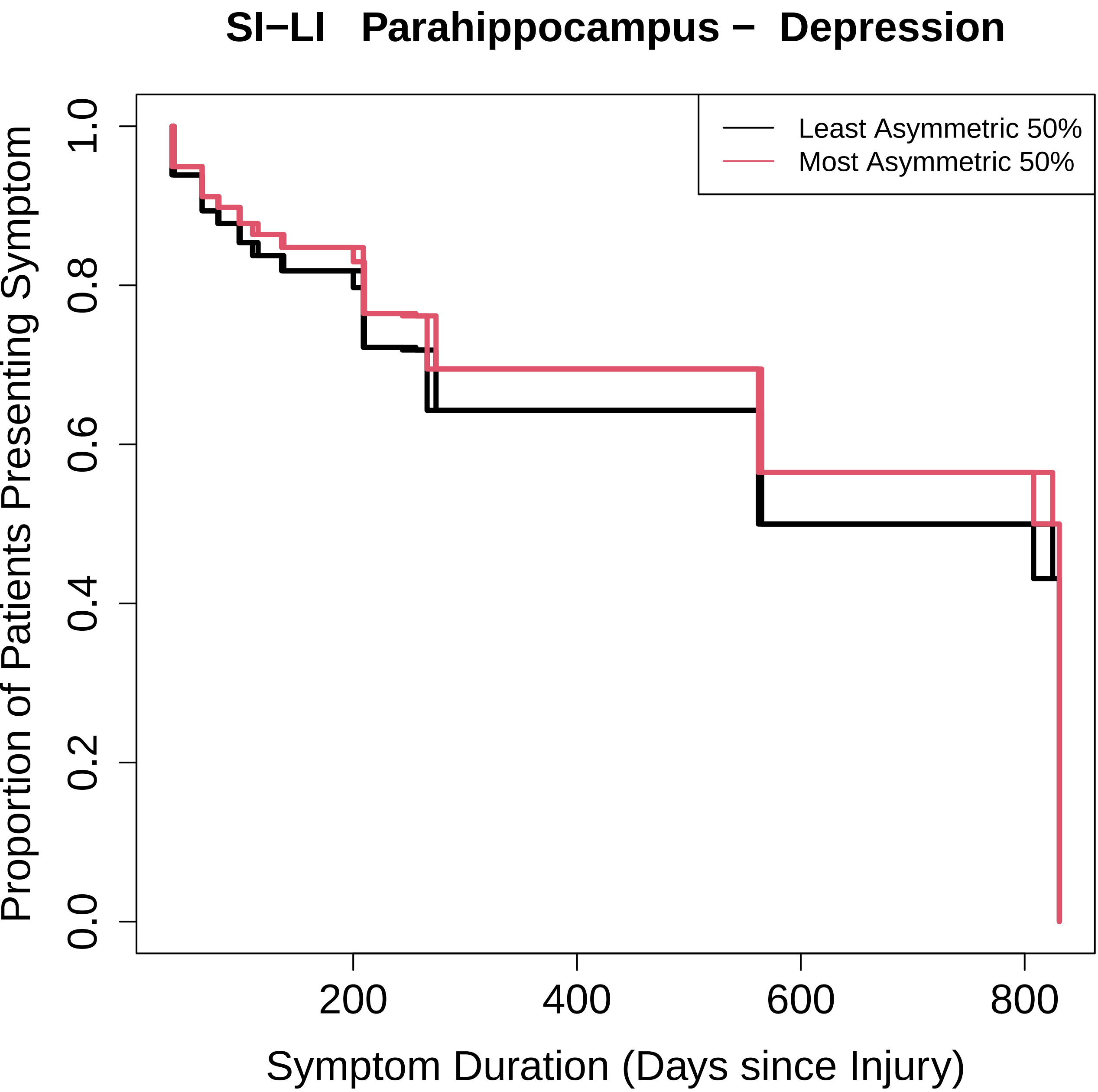

SI−LI Parahippocampus − Depression
Least Asymmetric 50%
Most Asymmetric 50%
Proportion of Patients Presenting Symptom
0.0
0.2
0.4
0.6
0.8
1.0
200
400
600
800
Symptom Duration (Days since Injury)

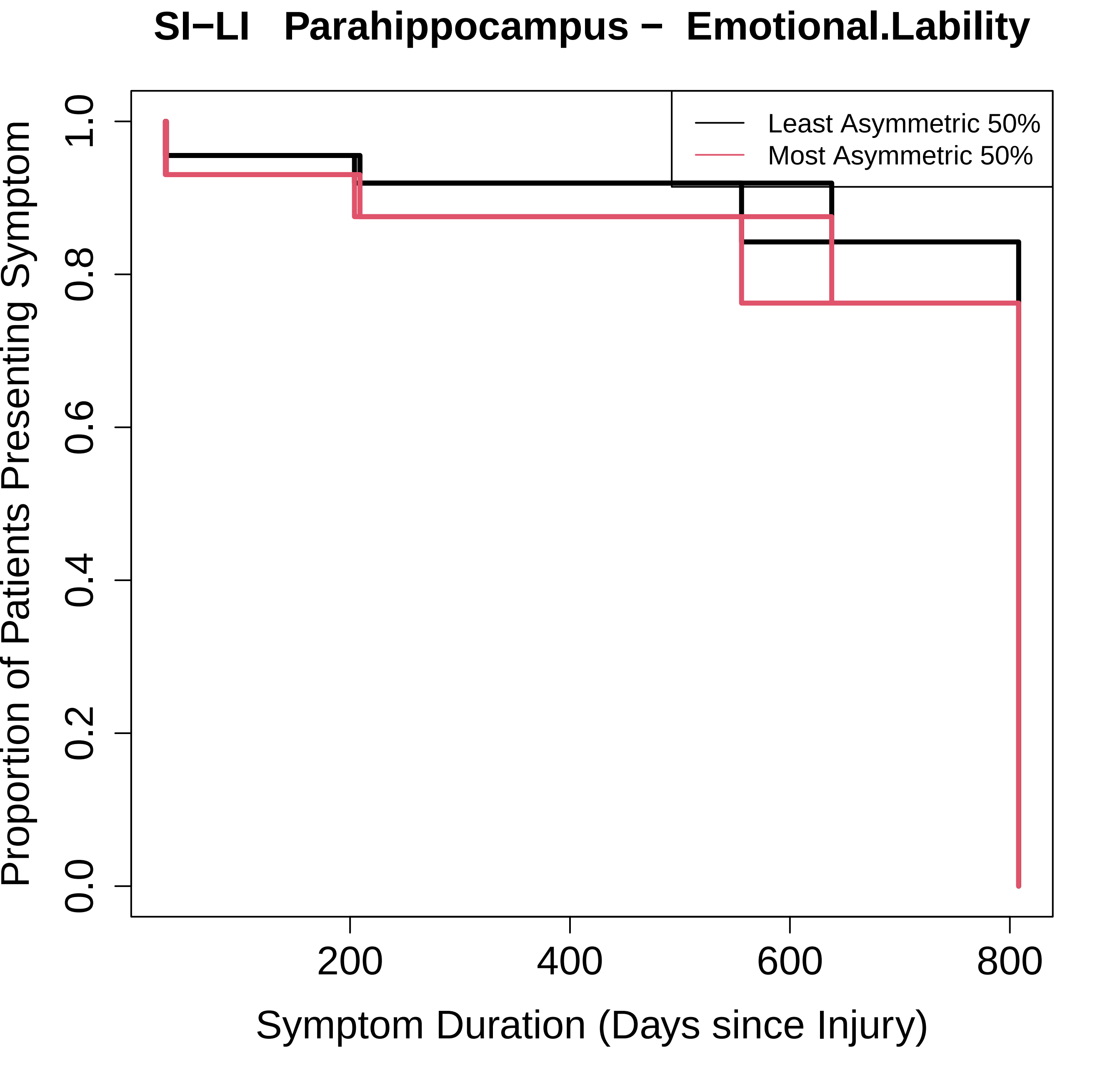

SI−LI Parahippocampus − Emotional.Lability
Least Asymmetric 50%
Most Asymmetric 50%
Proportion of Patients Presenting Symptom
0.0
0.2
0.4
0.6
0.8
1.0
200
400
600
800
Symptom Duration (Days since Injury)

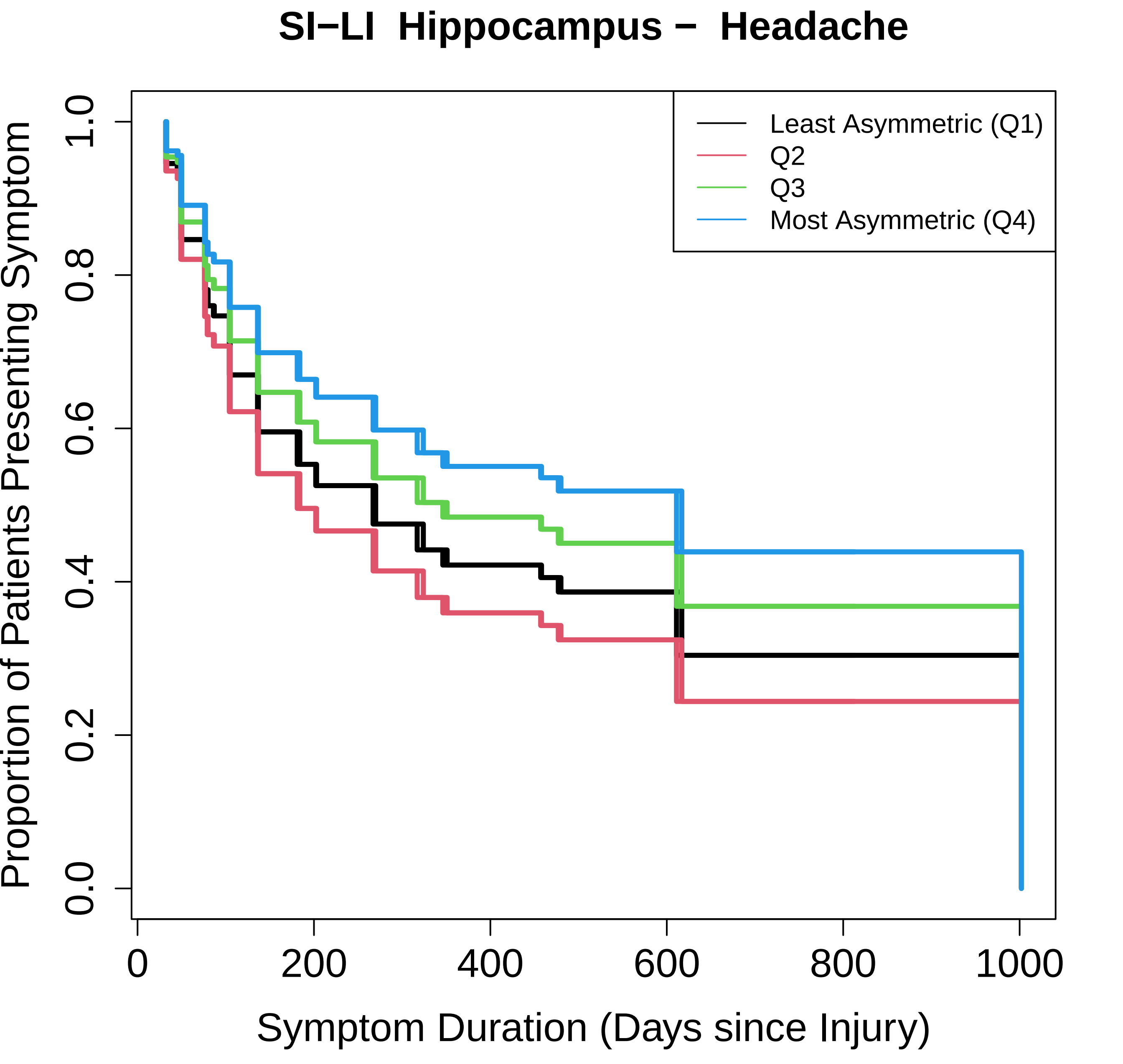

SI−LI Hippocampus − Headache
Least Asymmetric (Q1)
Q2
Q3
Most Asymmetric (Q4)
Proportion of Patients Presenting Symptom
0.0
0.2
0.4
0.6
0.8
1.0
0
200
400
600
800
1000
Symptom Duration (Days since Injury)

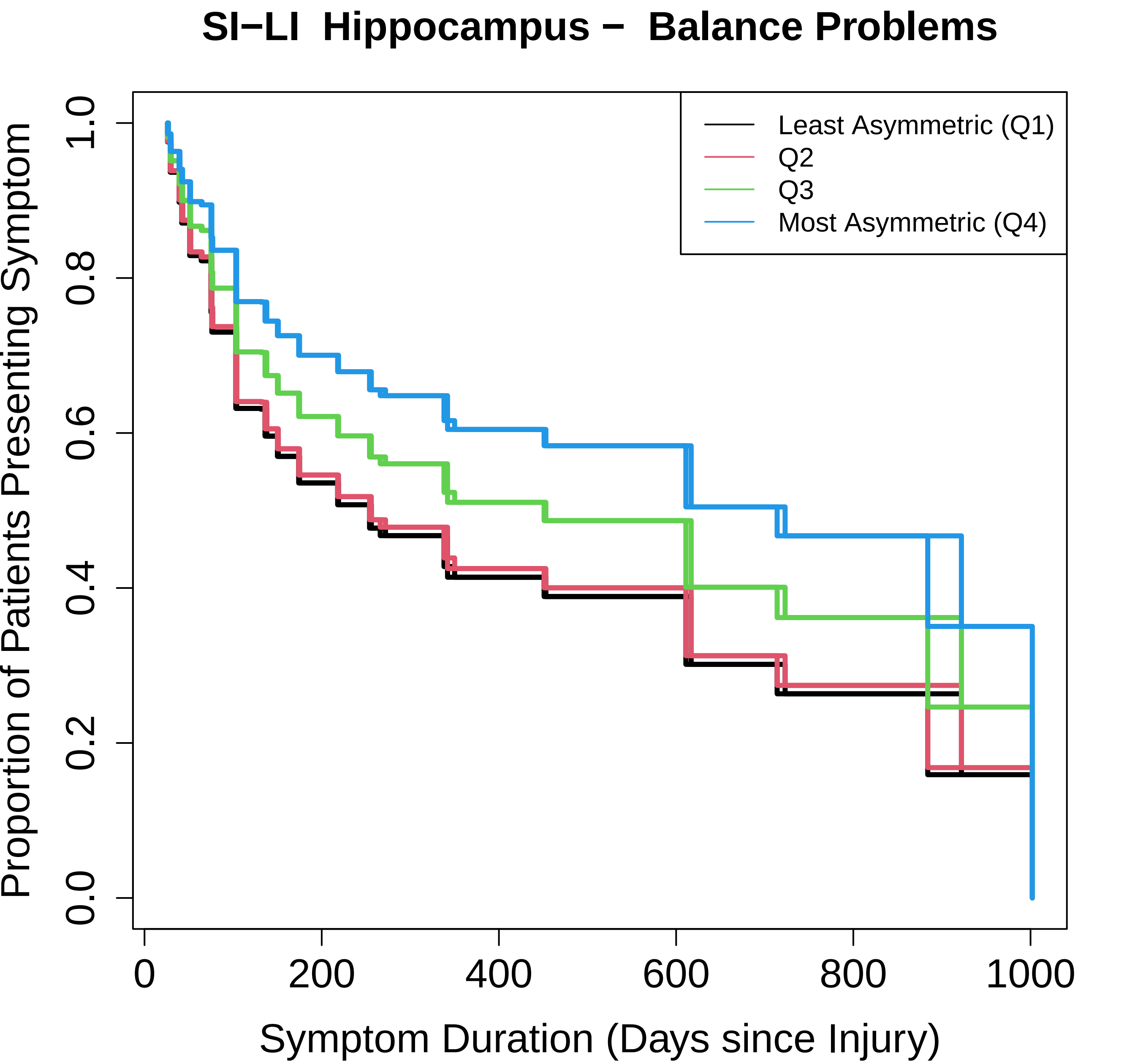

SI−LI Hippocampus − Balance Problems
Least Asymmetric (Q1)
Q2
Q3
Most Asymmetric (Q4)
Proportion of Patients Presenting Symptom
0.0
0.2
0.4
0.6
0.8
1.0
0
200
400
600
800
1000
Symptom Duration (Days since Injury)

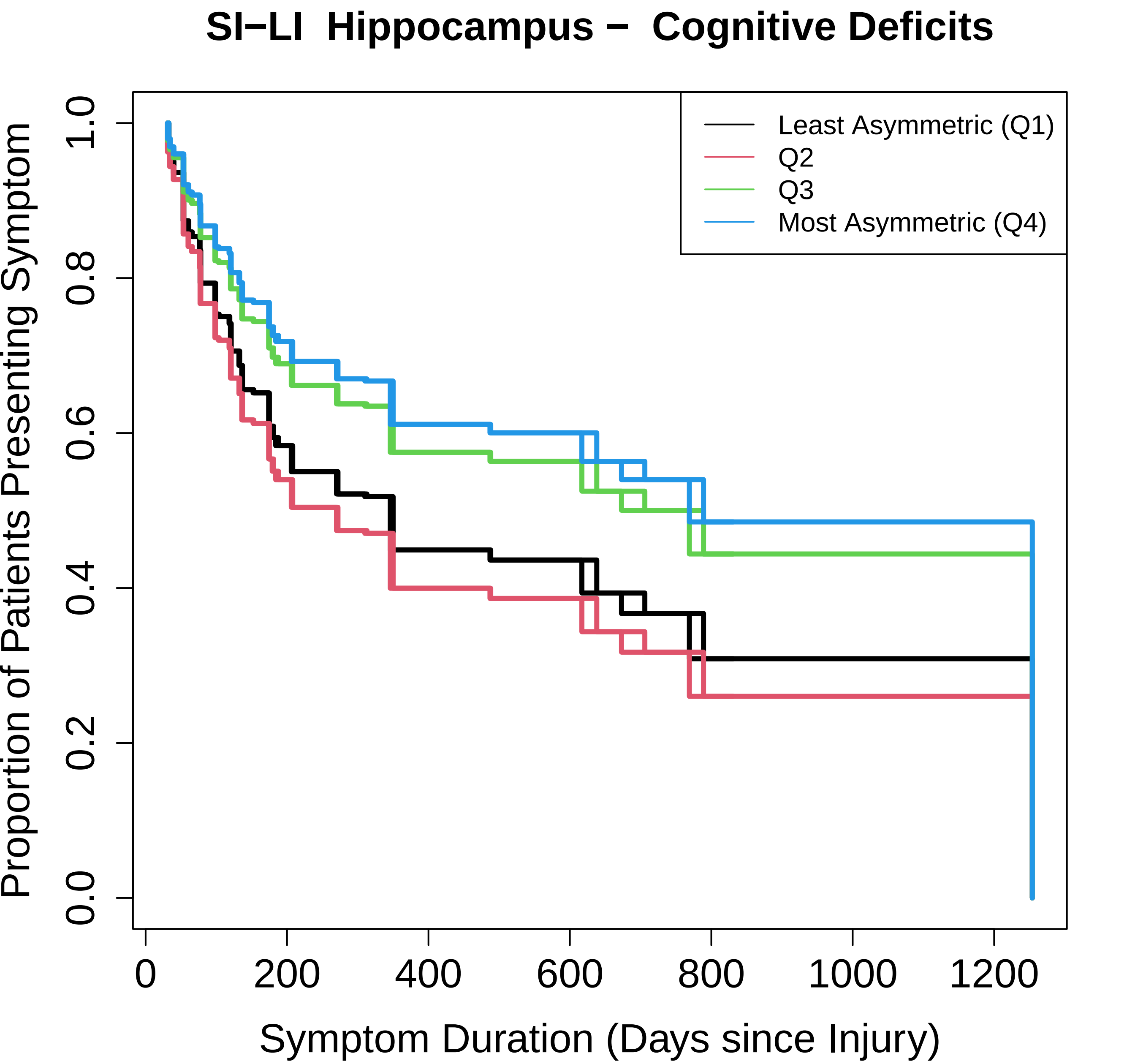
SI−LI Hippocampus − Cognitive Deficits
Least Asymmetric (Q1)
Q2
Q3
Most Asymmetric (Q4)
Proportion of Patients Presenting Symptom
0.0
0.2
0.4
0.6
0.8
1.0
0
200
400
600
800
1000
1200
Symptom Duration (Days since Injury)

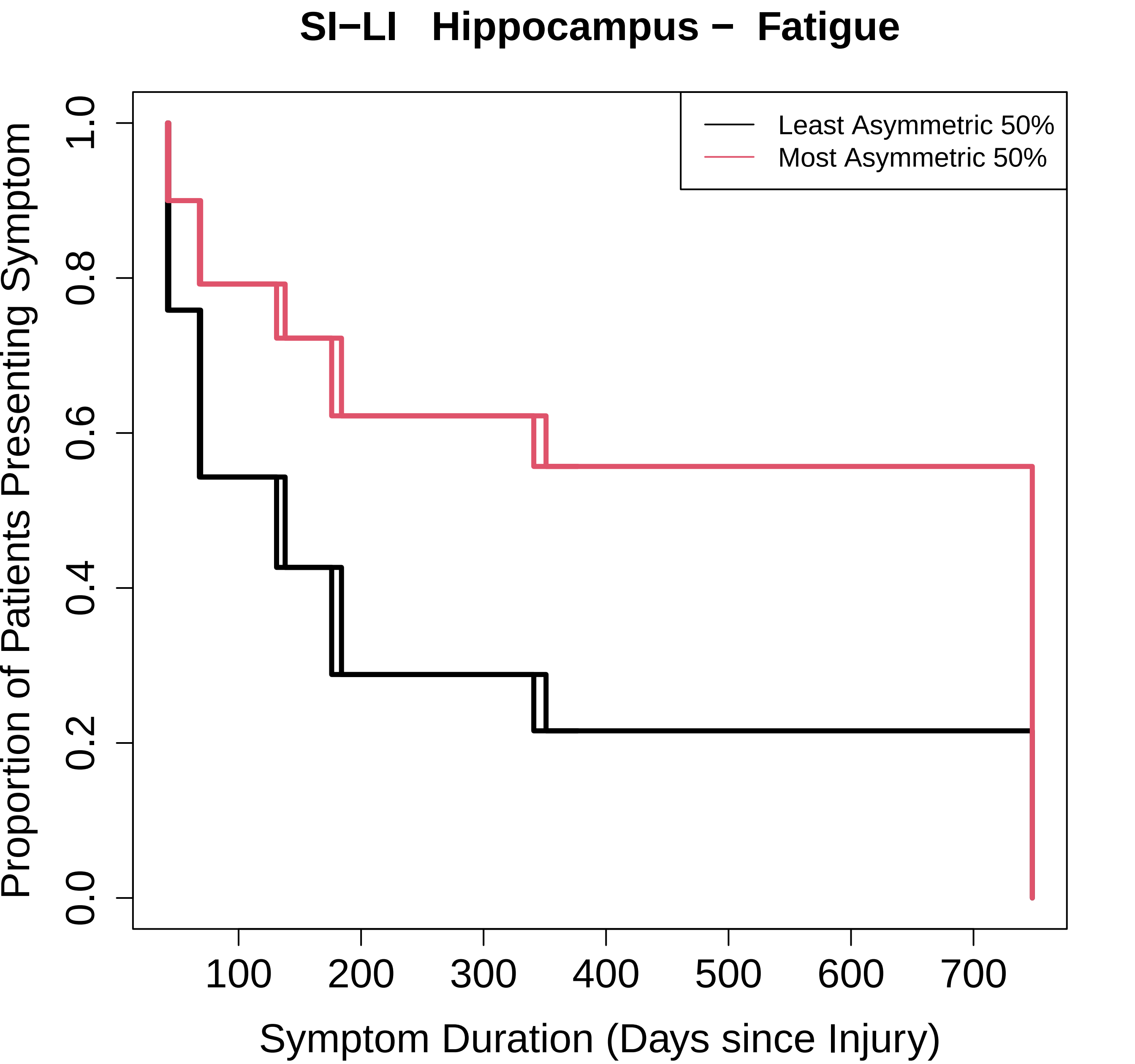

SI−LI Hippocampus − Fatigue
Least Asymmetric 50%
Most Asymmetric 50%
Proportion of Patients Presenting Symptom
0.0
0.2
0.4
0.6
0.8
1.0
100
200
300
400
500
600
700
Symptom Duration (Days since Injury)

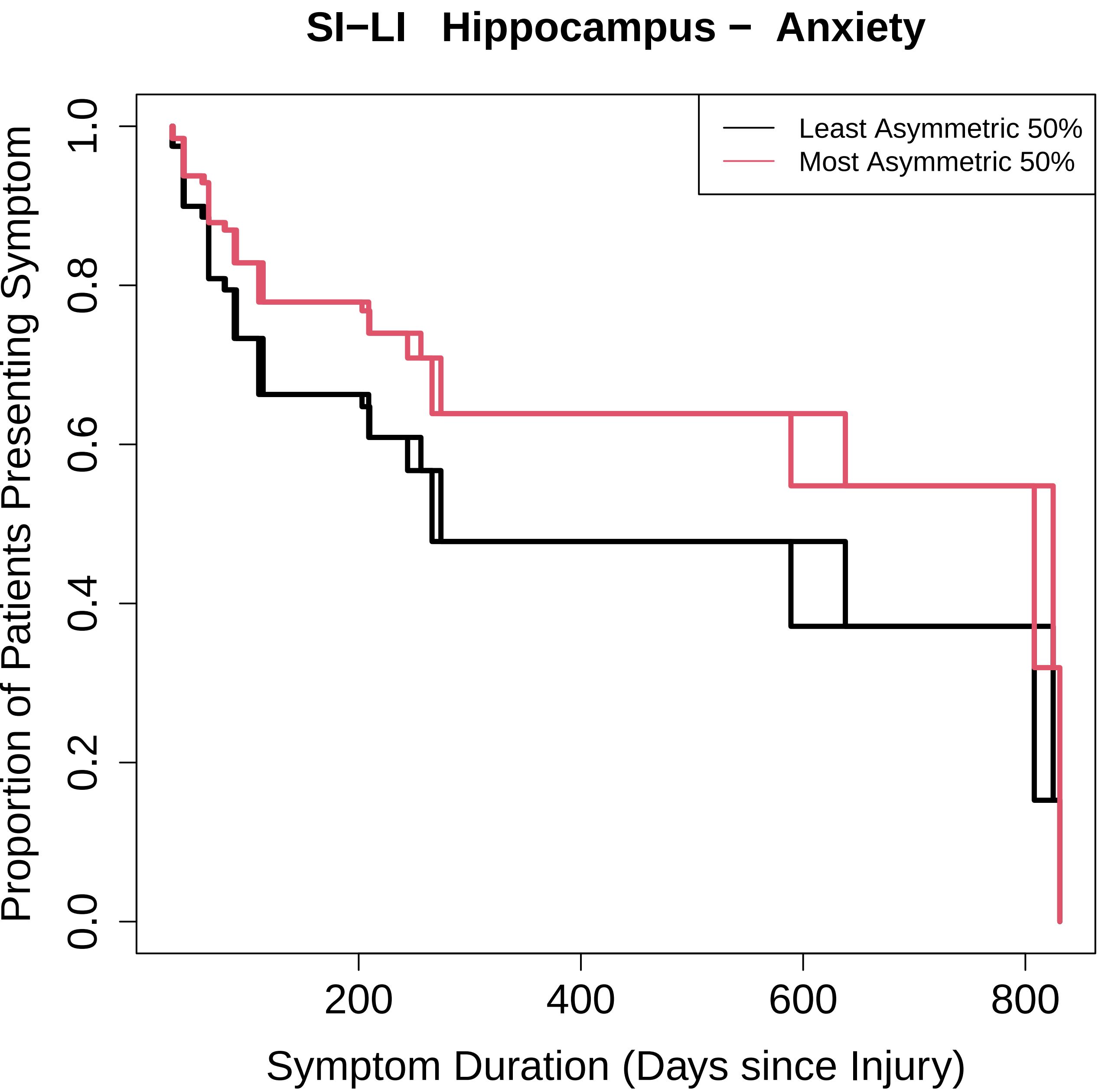

SI−LI Hippocampus − Anxiety
Least Asymmetric 50%
Most Asymmetric 50%
Proportion of Patients Presenting Symptom
0.0
0.2
0.4
0.6
0.8
1.0
200
400
600
800
Symptom Duration (Days since Injury)

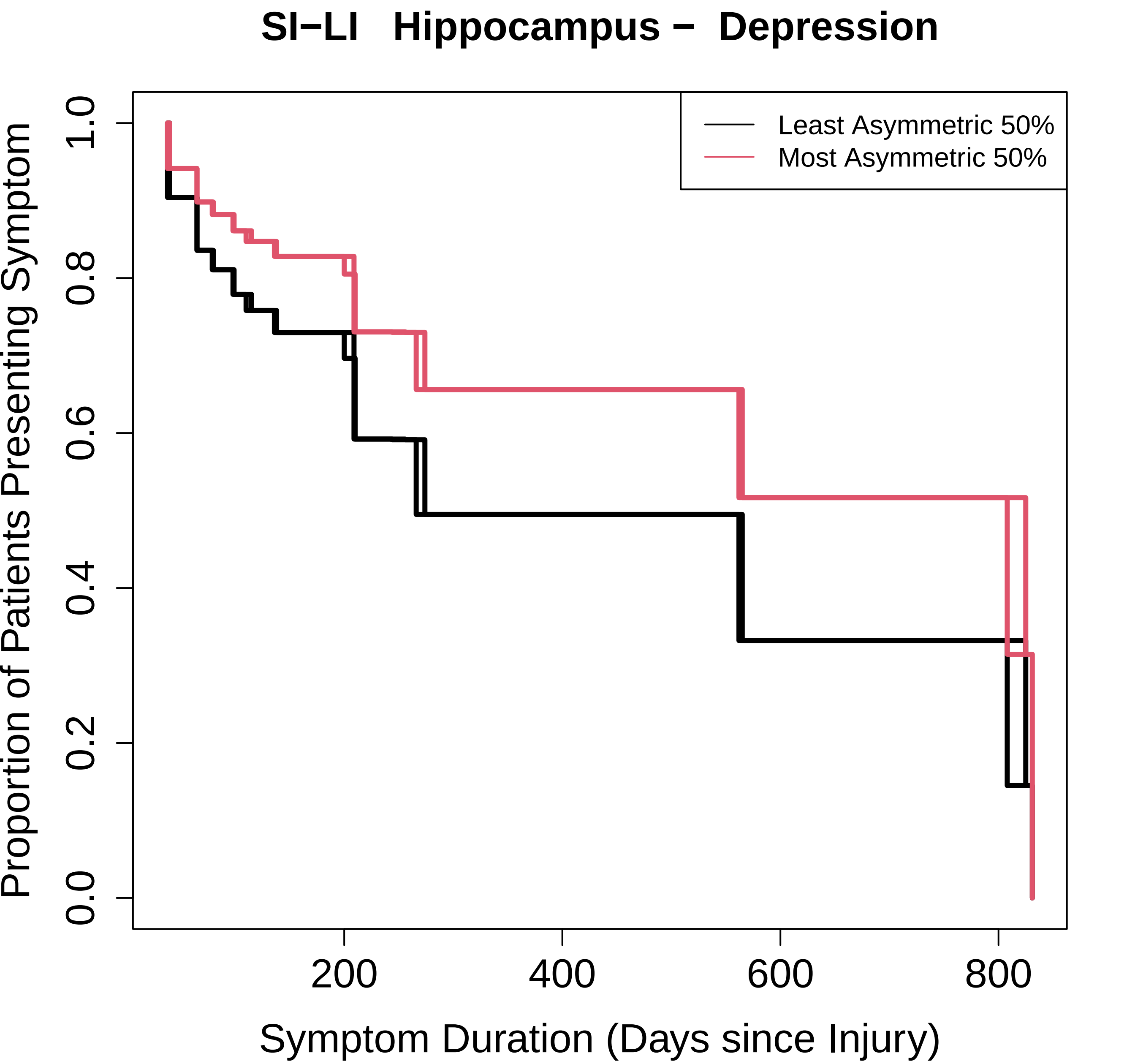
SI−LI Hippocampus − Depression
Least Asymmetric 50%
Most Asymmetric 50%
Proportion of Patients Presenting Symptom
0.0
0.2
0.4
0.6
0.8
1.0
200
400
600
800
Symptom Duration (Days since Injury)

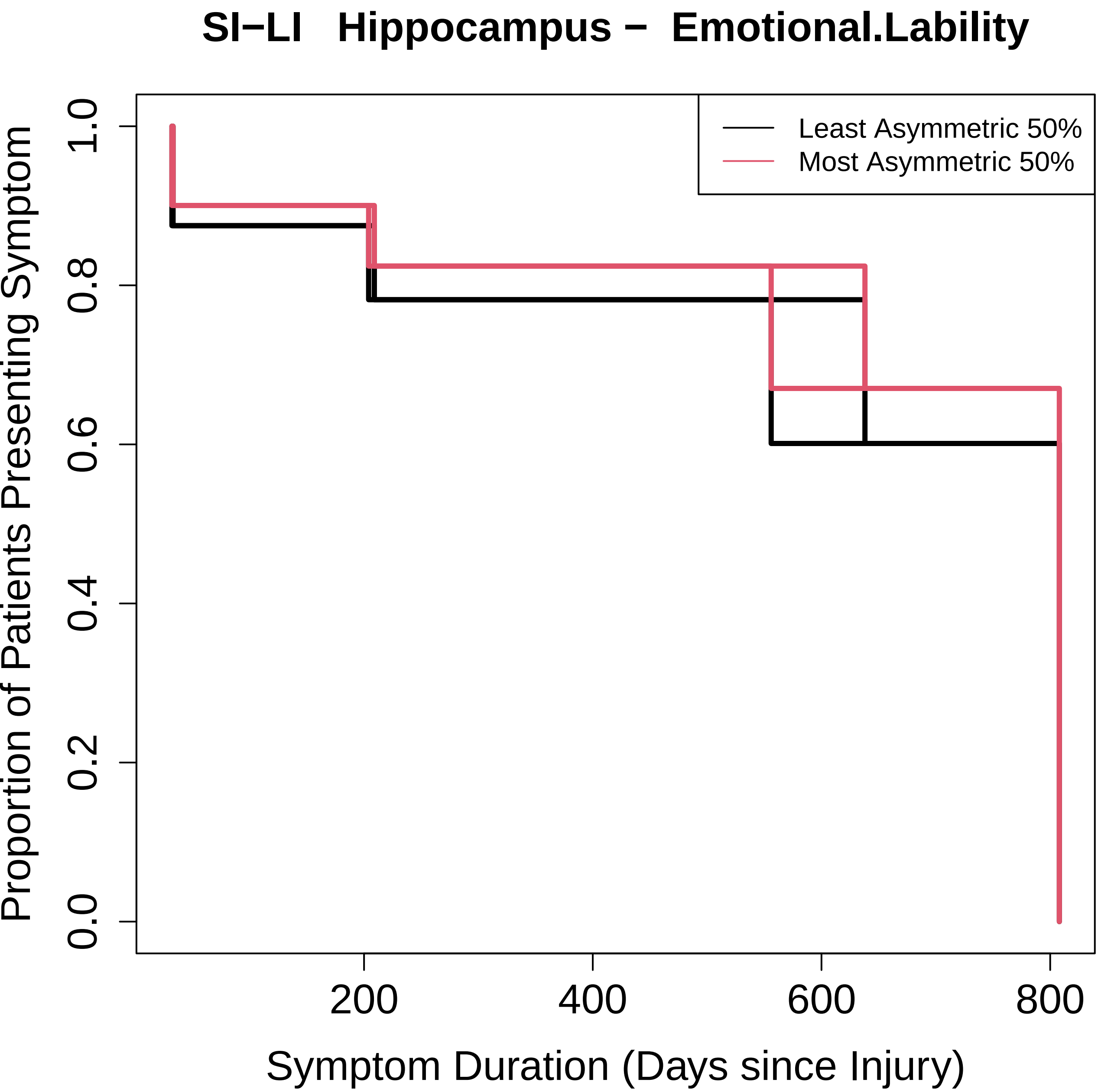
SI−LI Hippocampus − Emotional.Lability
Least Asymmetric 50%
Most Asymmetric 50%
Proportion of Patients Presenting Symptom
0.0
0.2
0.4
0.6
0.8
1.0
200
400
600
800
Symptom Duration (Days since Injury)

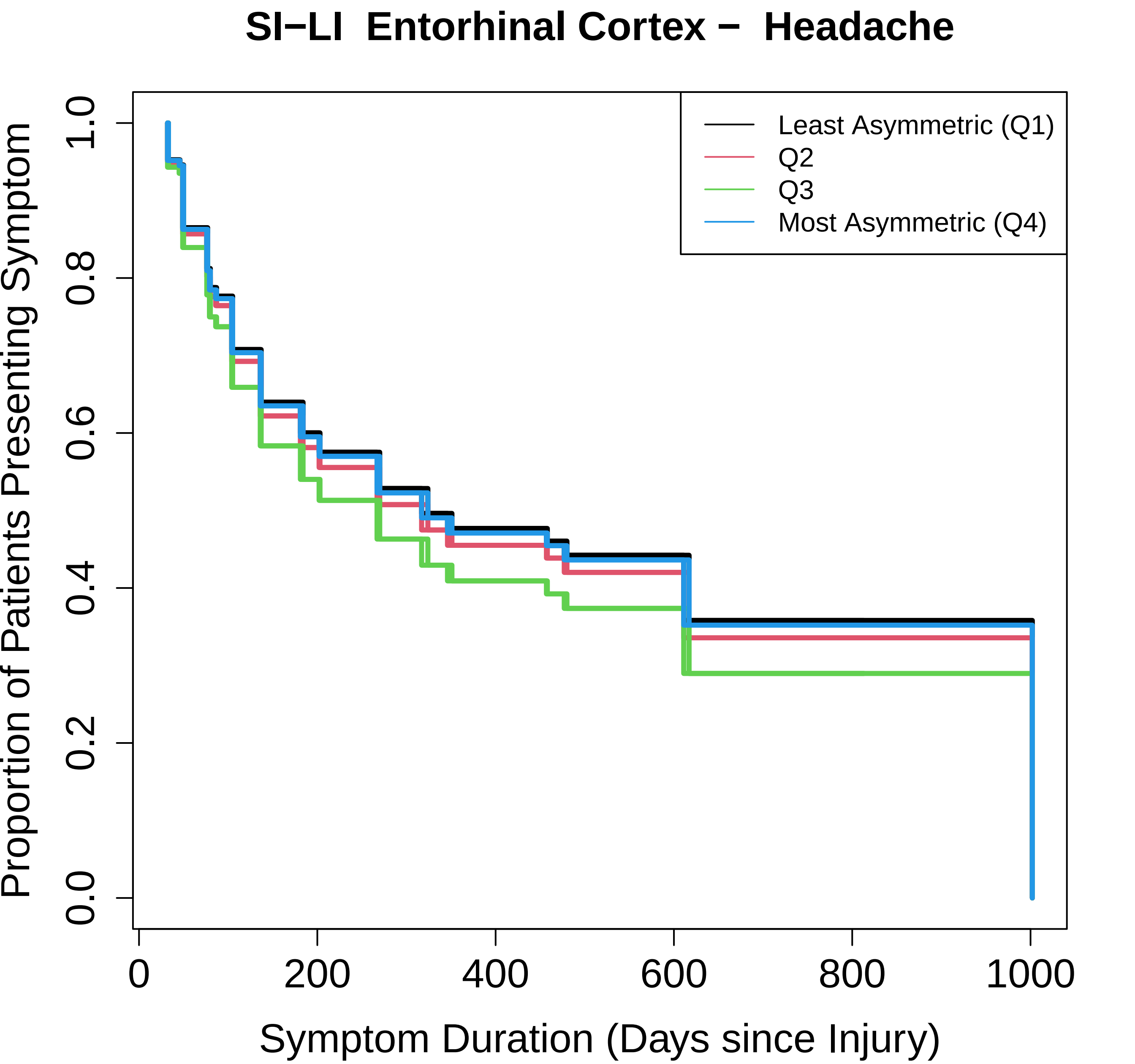
SI−LI Entorhinal Cortex − Headache
Least Asymmetric (Q1)
Q2
Q3
Most Asymmetric (Q4)
Proportion of Patients Presenting Symptom
0.0
0.2
0.4
0.6
0.8
1.0
0
200
400
600
800
1000
Symptom Duration (Days since Injury)

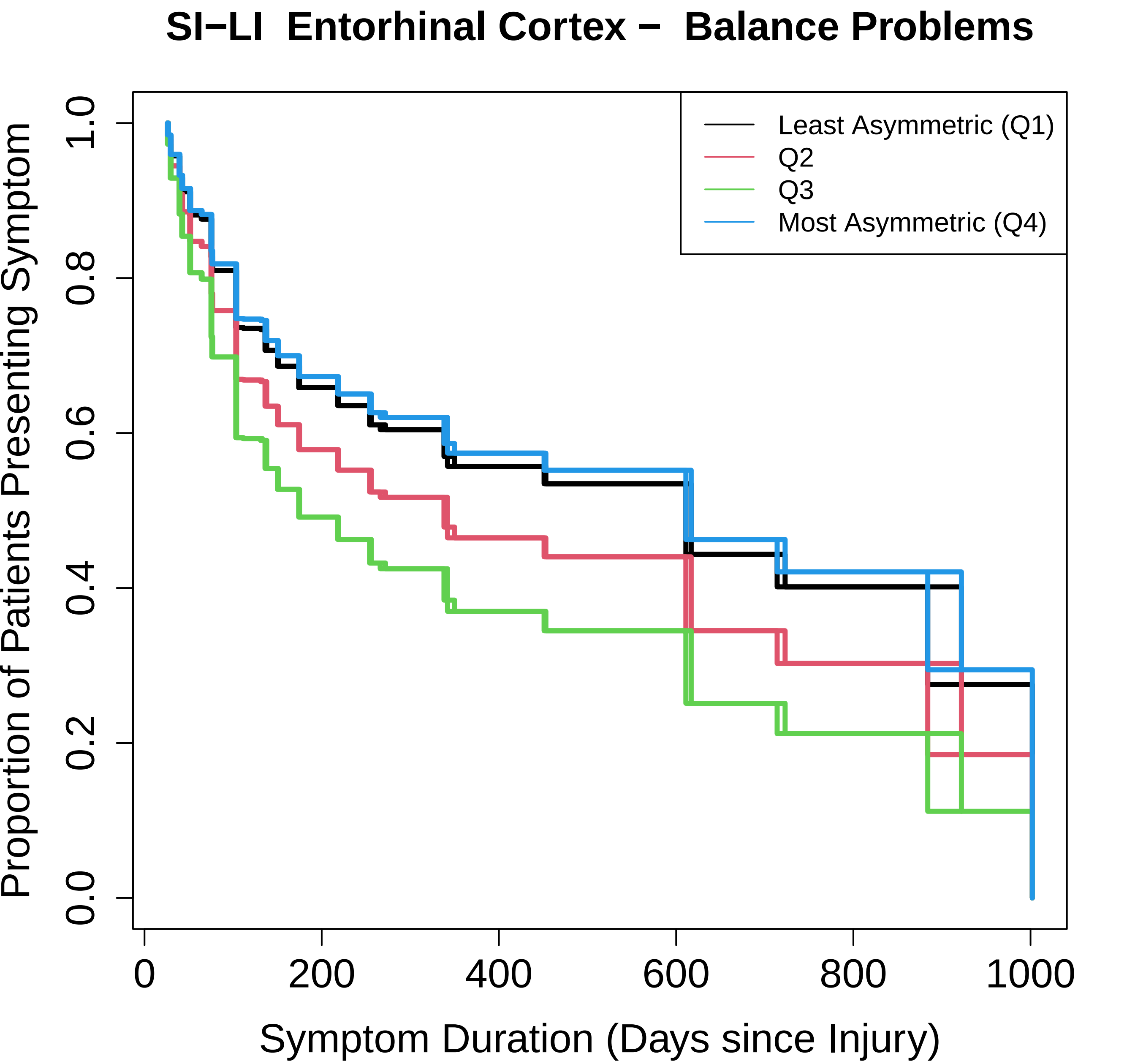
SI−LI Entorhinal Cortex − Balance Problems
Least Asymmetric (Q1)
Q2
Q3
Most Asymmetric (Q4)
Proportion of Patients Presenting Symptom
0.0
0.2
0.4
0.6
0.8
1.0
0
200
400
600
800
1000
Symptom Duration (Days since Injury)

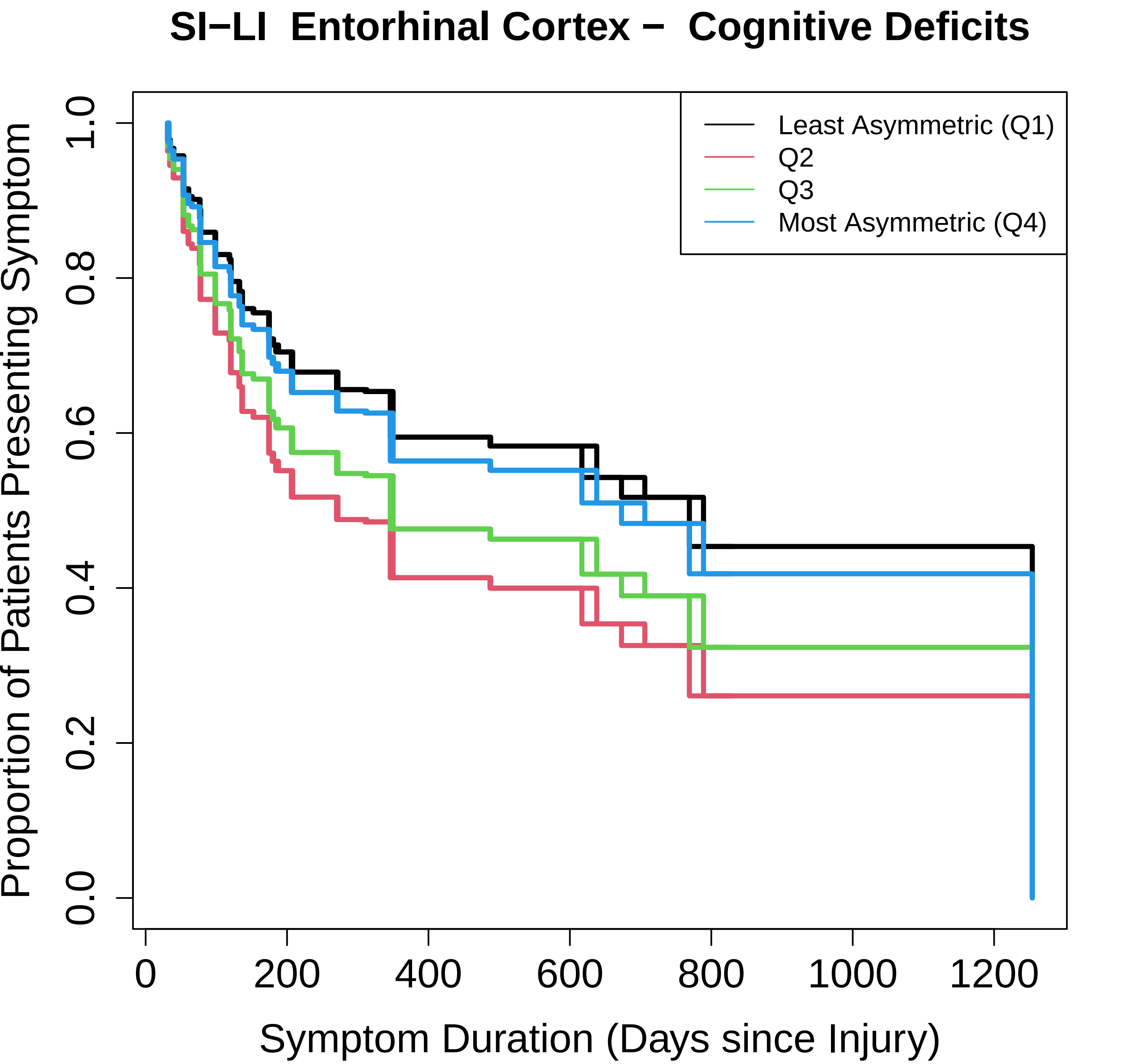
SI−LI Entorhinal Cortex − Cognitive Deficits
Least Asymmetric (Q1)
Q2
Q3
Most Asymmetric (Q4)
Proportion of Patients Presenting Symptom
0.0
0.2
0.4
0.6
0.8
1.0
0
200
400
600
800
1000
1200
Symptom Duration (Days since Injury)

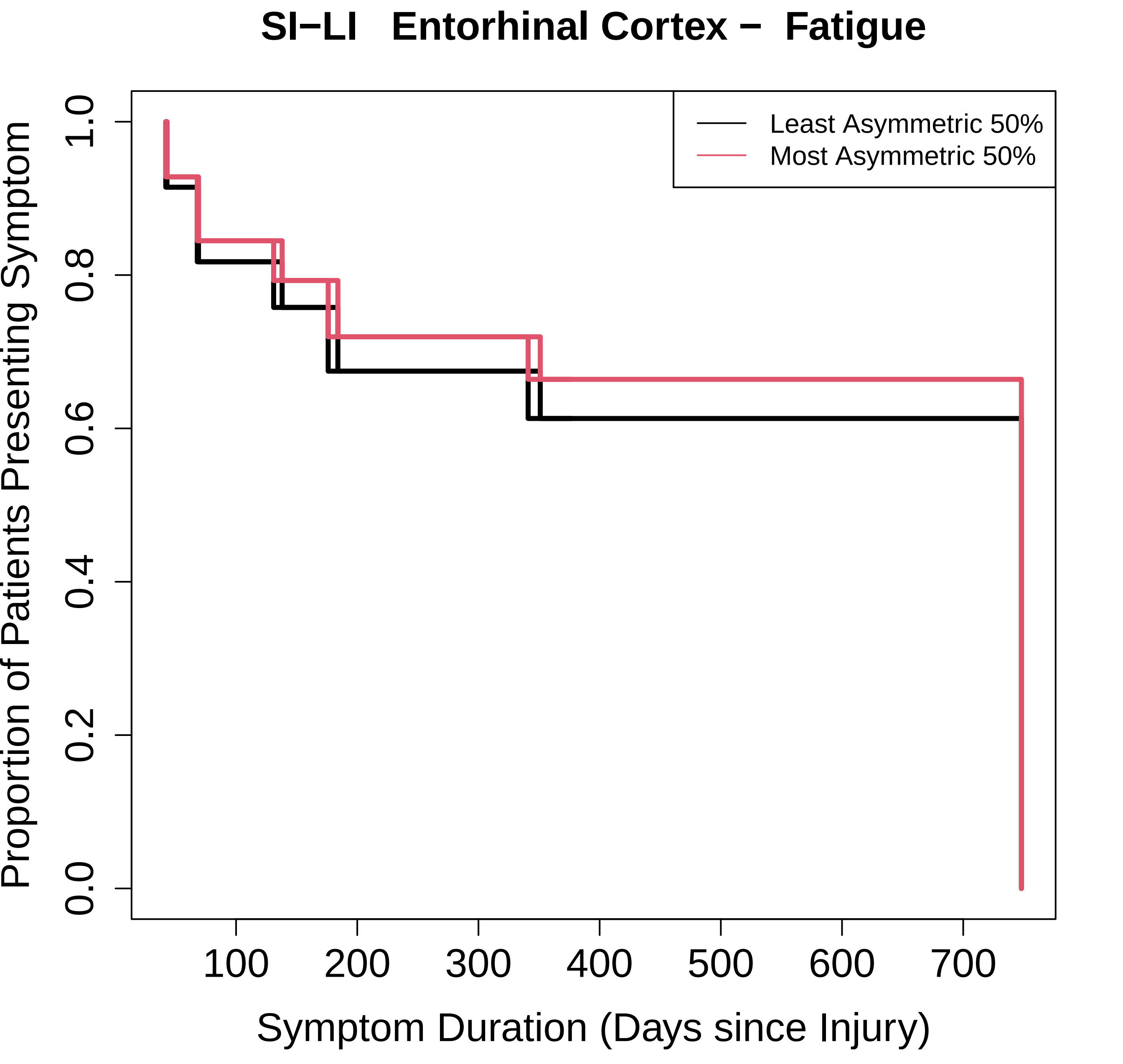

SI−LI Entorhinal Cortex − Fatigue
Least Asymmetric 50%
Most Asymmetric 50%
Proportion of Patients Presenting Symptom
1.0
0.8
0.6
0.4
0.2
0.0
100
200
300
400
500
600
700
Symptom Duration (Days since Injury)

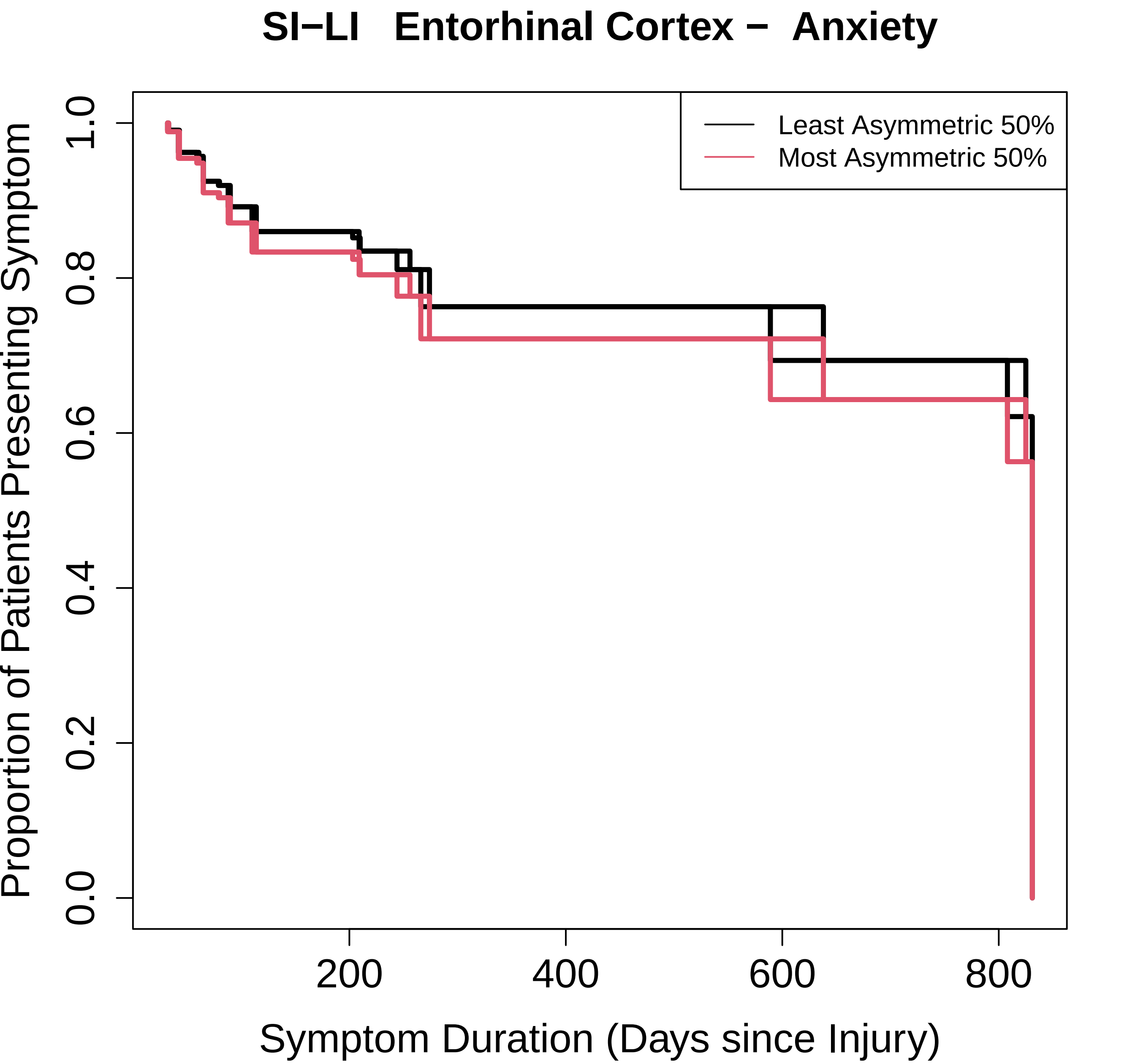

SI−LI Entorhinal Cortex − Anxiety
Least Asymmetric 50%
Most Asymmetric 50%
Proportion of Patients Presenting Symptom
0.0
0.2
0.4
0.6
0.8
1.0
200
400
600
800
Symptom Duration (Days since Injury)

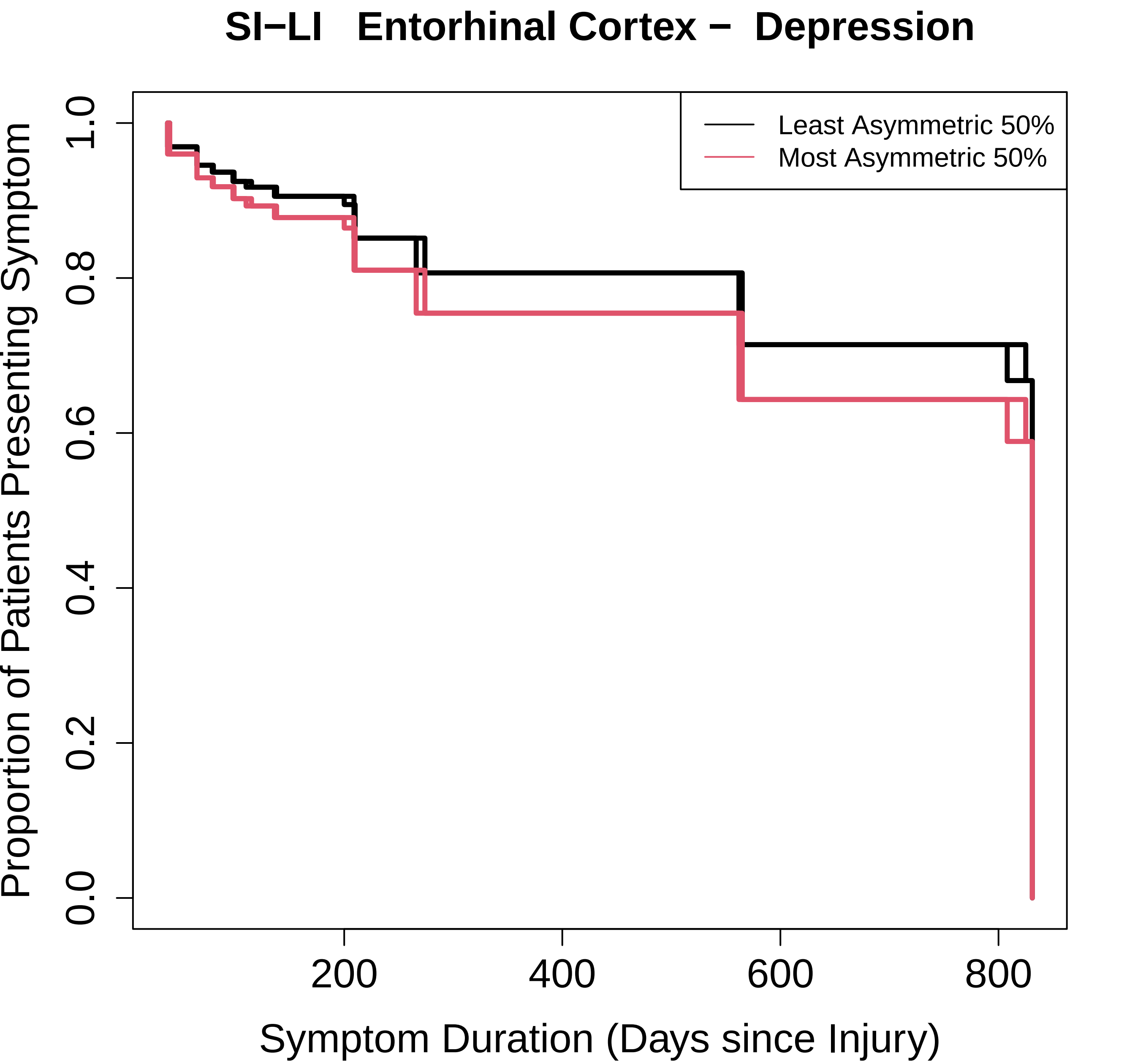
SI−LI Entorhinal Cortex − Depression
Least Asymmetric 50%
Most Asymmetric 50%
1.0
0.8
0.6
0.4
0.2
0.0
Proportion of Patients Presenting Symptom
200
400
600
800
Symptom Duration (Days since Injury)

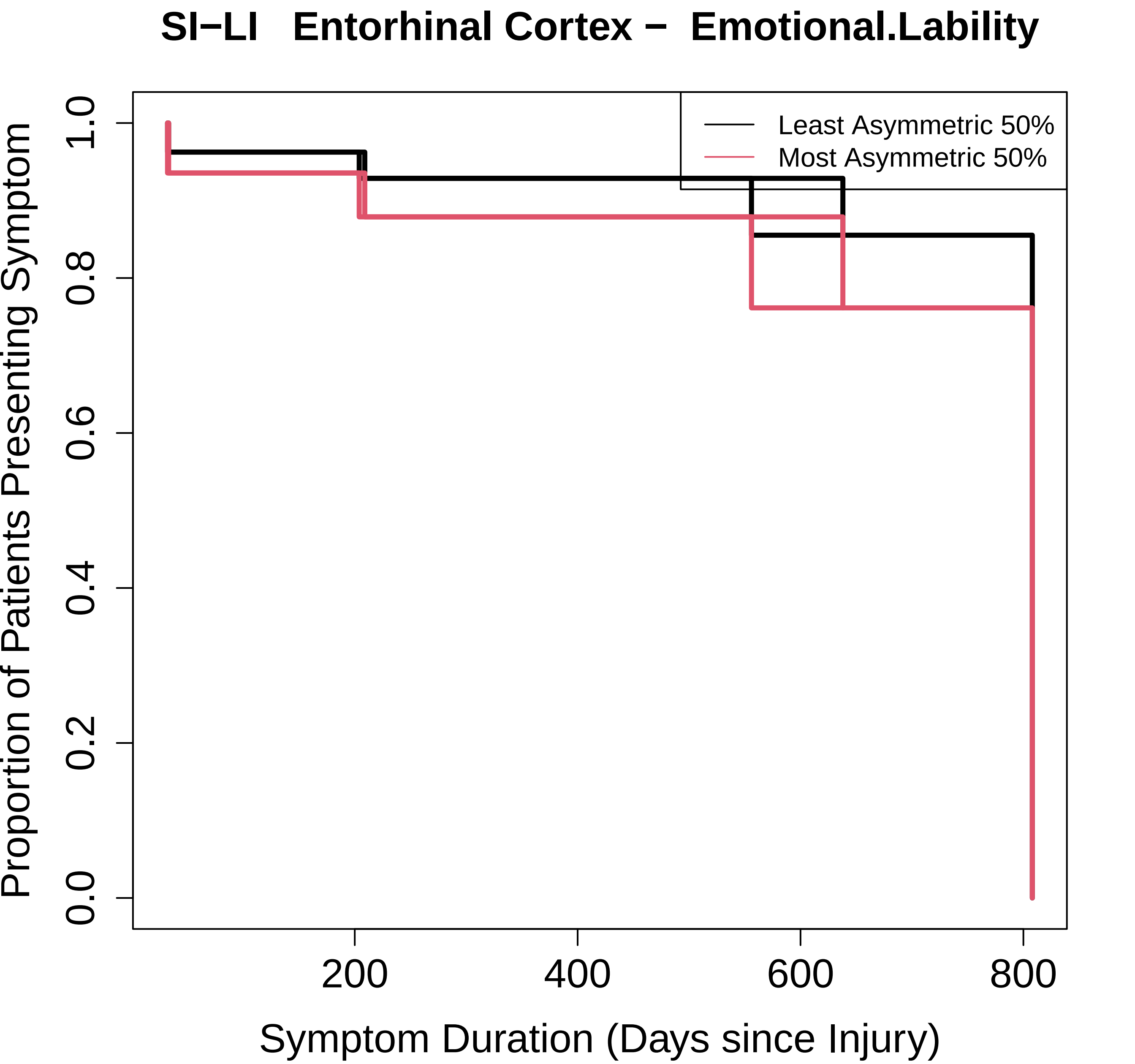
SI−LI Entorhinal Cortex − Emotional.Lability
Least Asymmetric 50%
Most Asymmetric 50%
Proportion of Patients Presenting Symptom
0.0
0.2
0.4
0.6
0.8
1.0
200
400
600
800
Symptom Duration (Days since Injury)

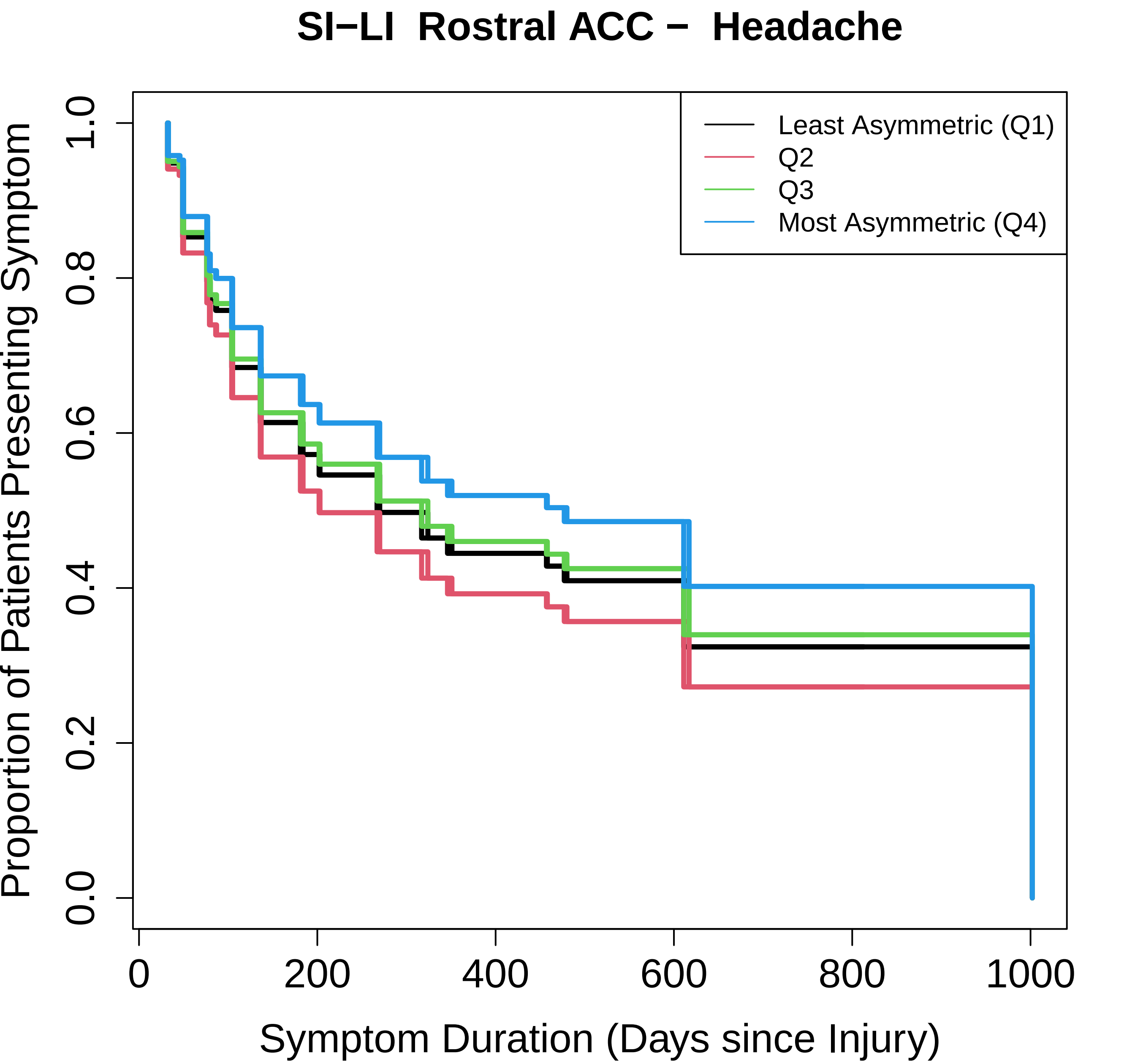
SI−LI Rostral ACC − Headache
Least Asymmetric (Q1)
Q2
Q3
Most Asymmetric (Q4)
Proportion of Patients Presenting Symptom
0.0
0.2
0.4
0.6
0.8
1.0
0
200
400
600
800
1000
Symptom Duration (Days since Injury)

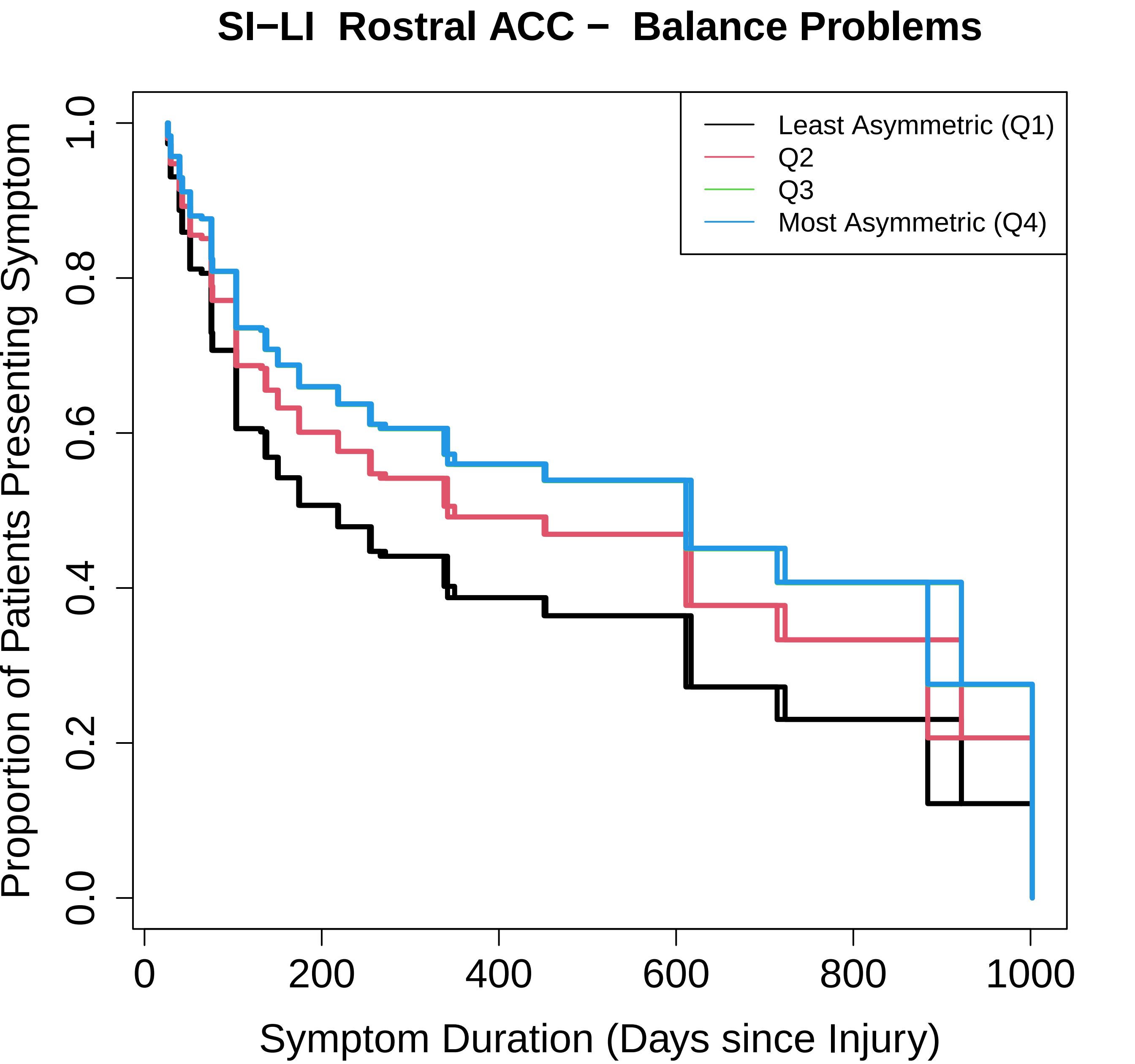
SI−LI Rostral ACC − Balance Problems
Least Asymmetric (Q1)
Q2
Q3
Most Asymmetric (Q4)
Proportion of Patients Presenting Symptom
0.0
0.2
0.4
0.6
0.8
1.0
0
200
400
600
800
1000
Symptom Duration (Days since Injury)

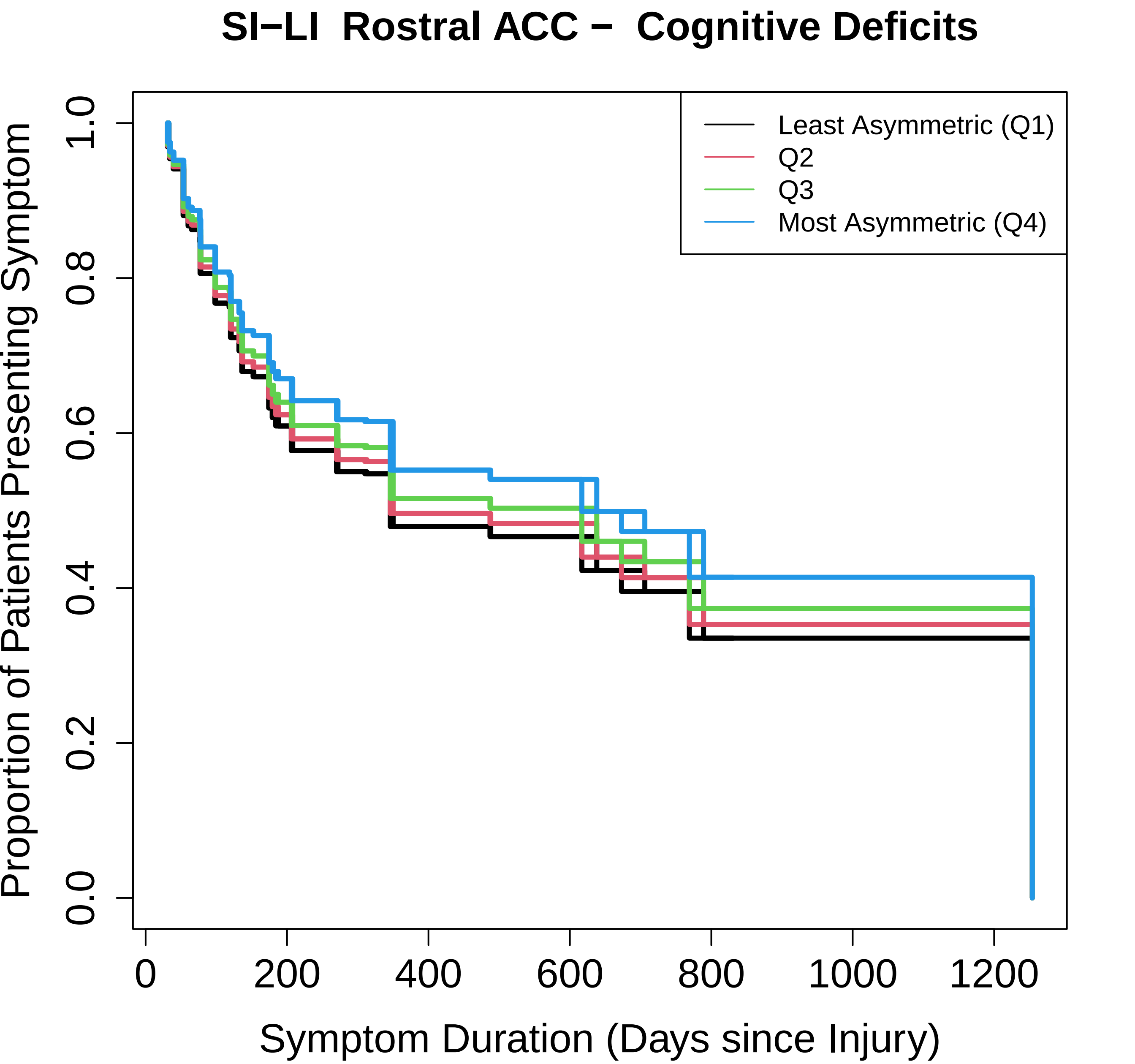

SI−LI Rostral ACC − Cognitive Deficits
Least Asymmetric (Q1)
Q2
Q3
Most Asymmetric (Q4)
Proportion of Patients Presenting Symptom
0.0
0.2
0.4
0.6
0.8
1.0
0
200
400
600
800
1000
1200
Symptom Duration (Days since Injury)

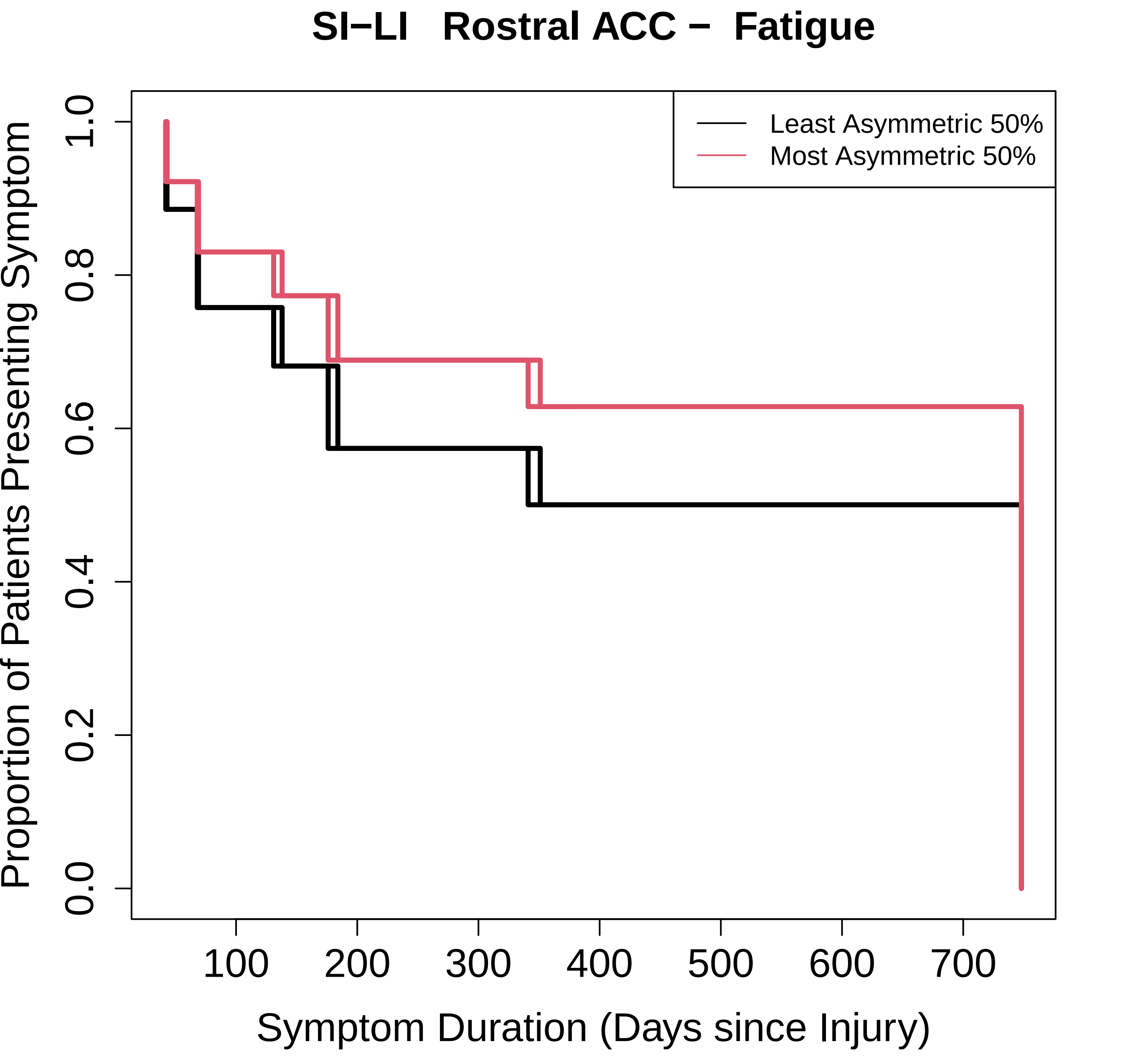
SI−LI Rostral ACC − Fatigue
Least Asymmetric 50%
Most Asymmetric 50%
Proportion of Patients Presenting Symptom
0.0
0.2
0.4
0.6
0.8
1.0
100
200
300
400
500
600
700
Symptom Duration (Days since Injury)

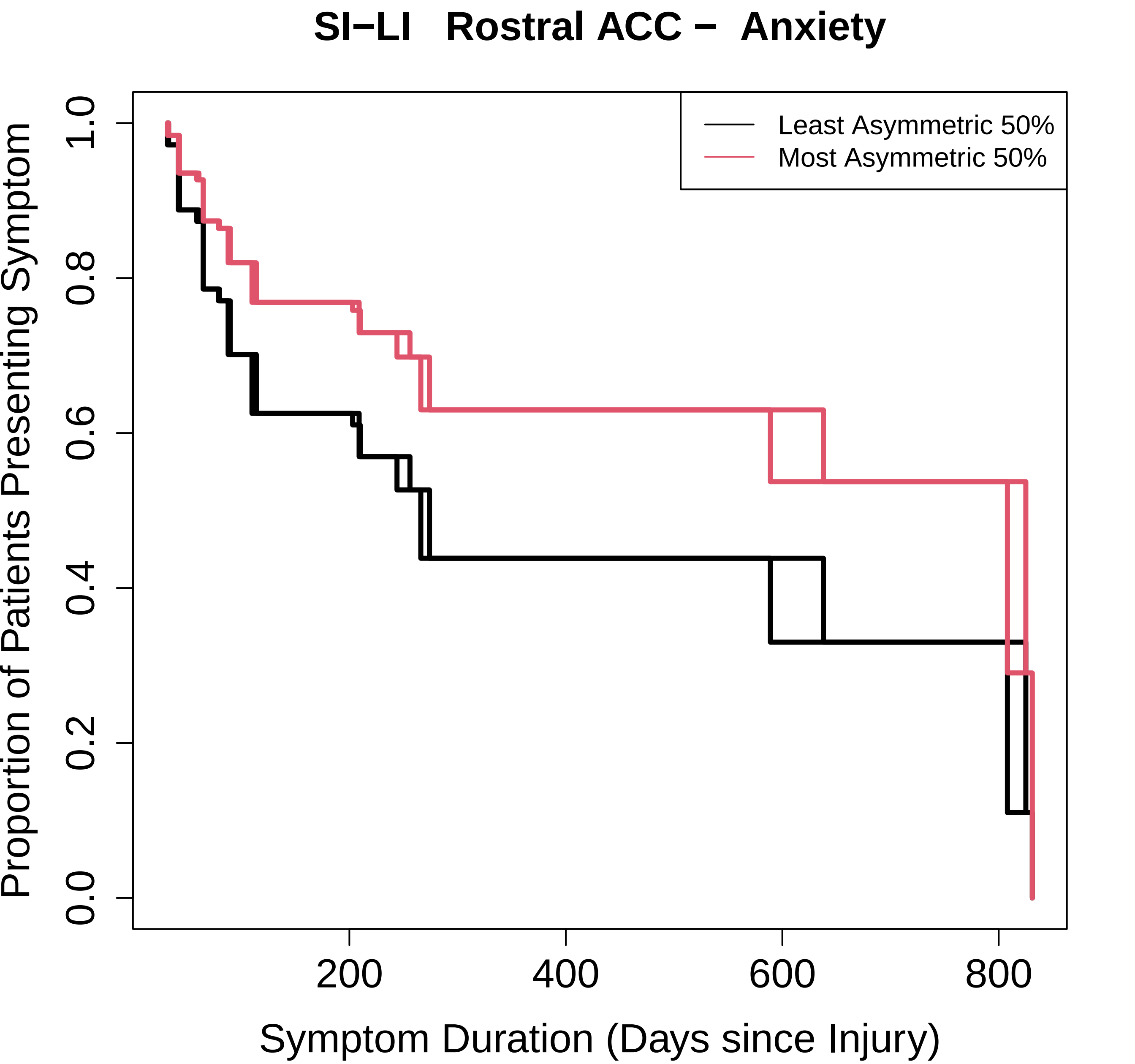

SI−LI Rostral ACC − Anxiety
Least Asymmetric 50%
Most Asymmetric 50%
Proportion of Patients Presenting Symptom
0.0
0.2
0.4
0.6
0.8
1.0
200
400
600
800
Symptom Duration (Days since Injury)

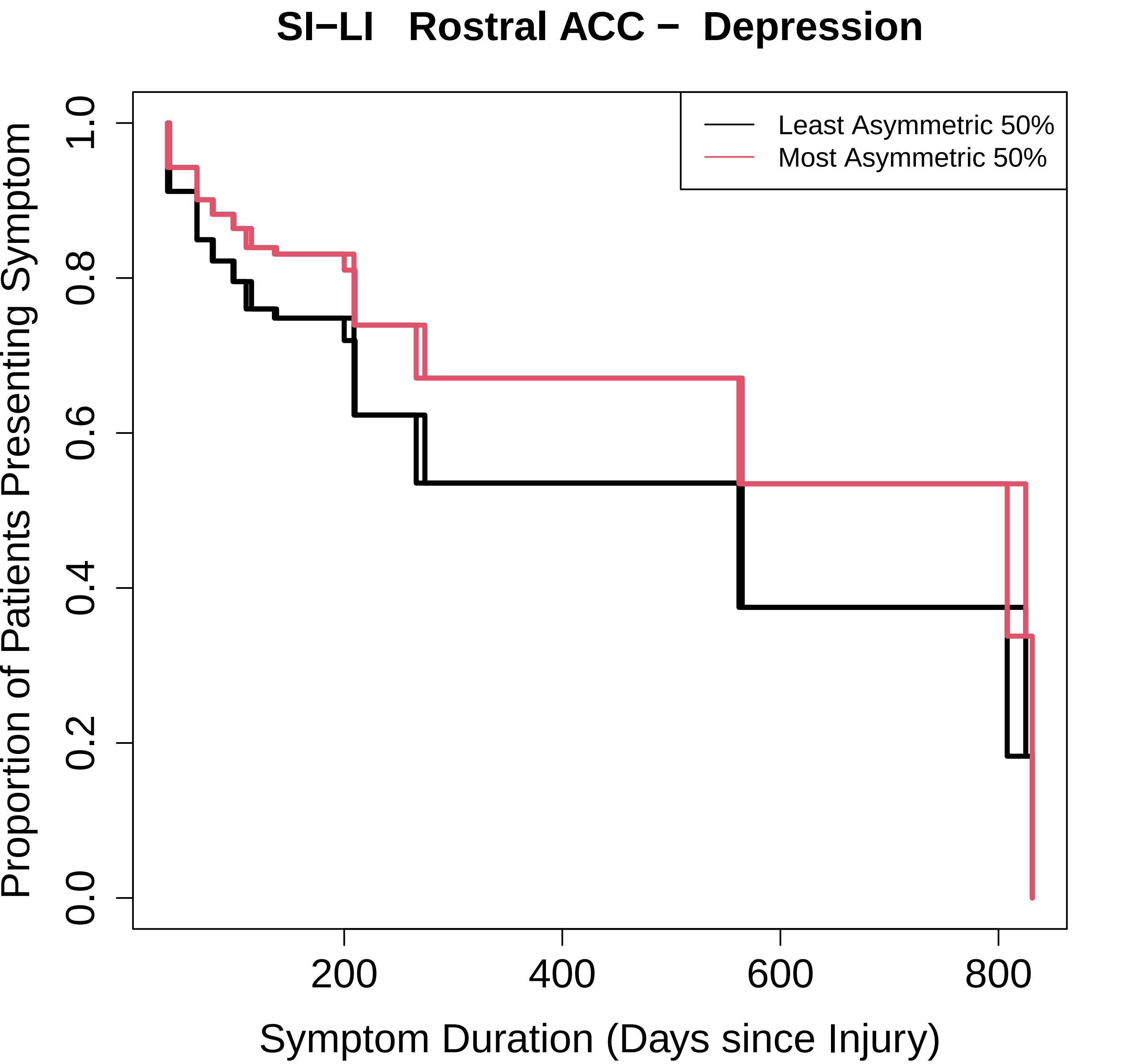

SI−LI Rostral ACC − Depression
Least Asymmetric 50%
Most Asymmetric 50%
Proportion of Patients Presenting Symptom
0.0
0.2
0.4
0.6
0.8
1.0
200
400
600
800
Symptom Duration (Days since Injury)

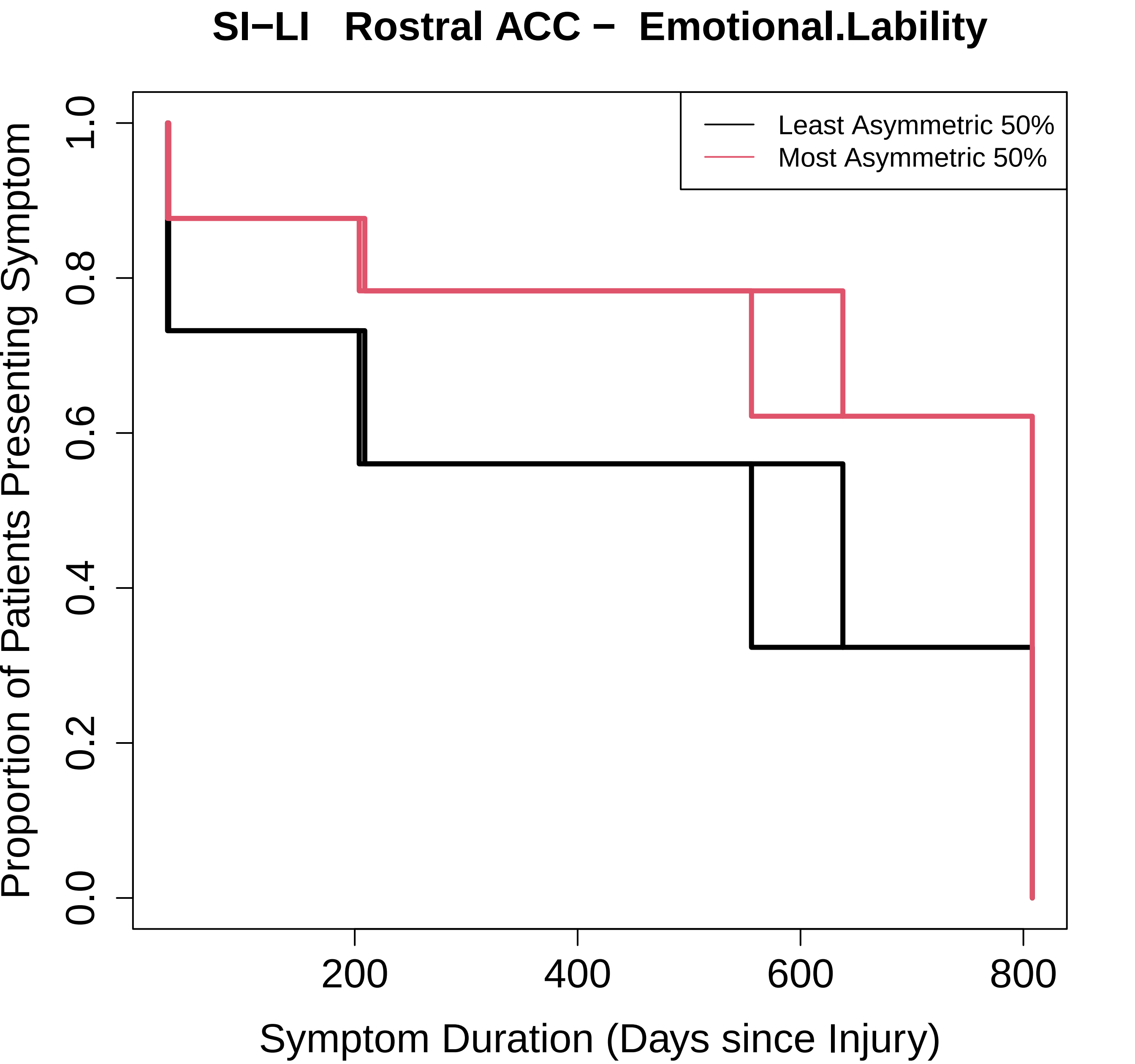
SI−LI Rostral ACC − Emotional.Lability
Least Asymmetric 50%
Most Asymmetric 50%
Proportion of Patients Presenting Symptom
0.0
0.2
0.4
0.6
0.8
1.0
200
400
600
800
Symptom Duration (Days since Injury)

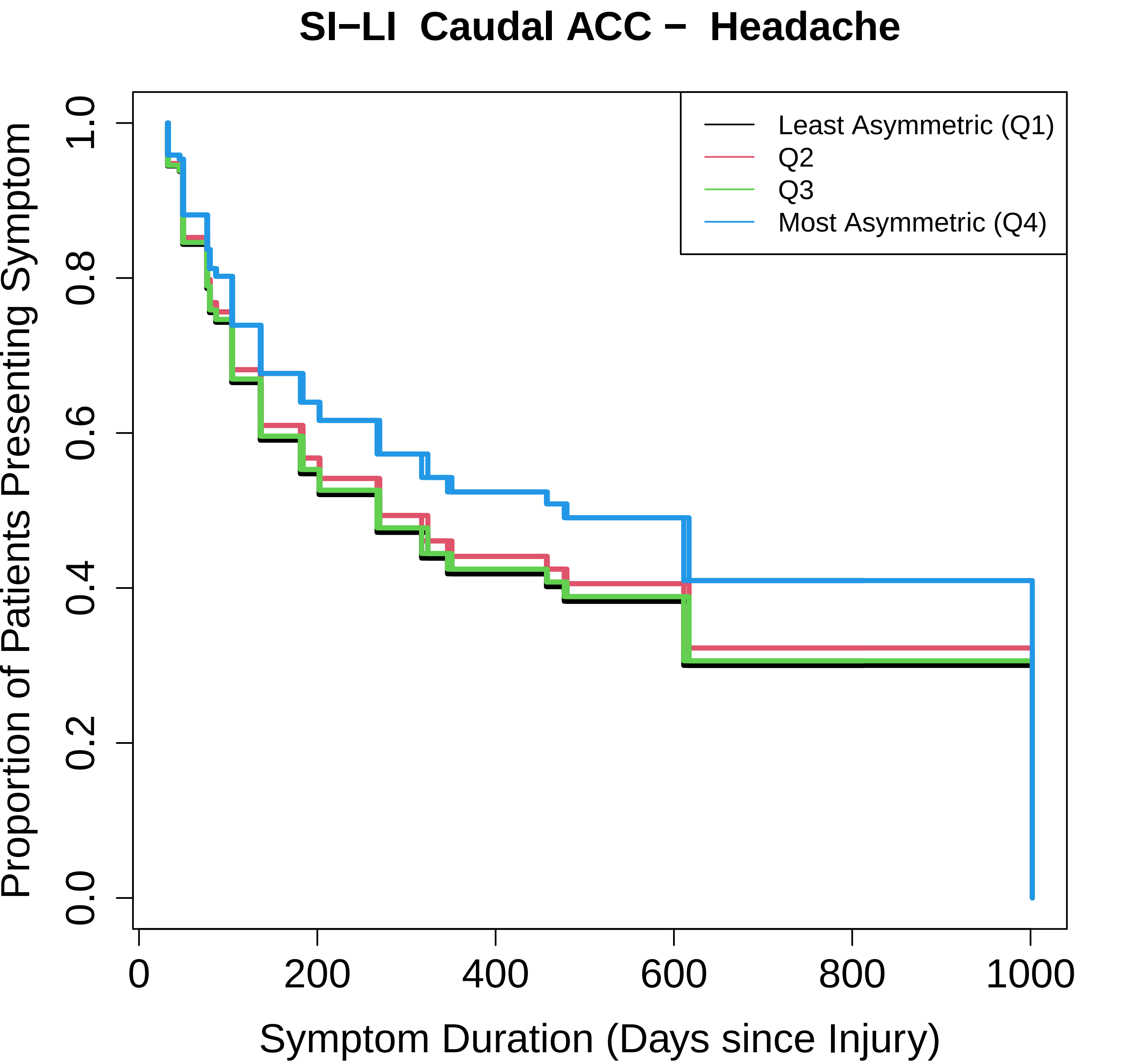

SI−LI Caudal ACC − Headache
Least Asymmetric (Q1)
Q2
Q3
Most Asymmetric (Q4)
Proportion of Patients Presenting Symptom
0.0
0.2
0.4
0.6
0.8
1.0
0
200
400
600
800
1000
Symptom Duration (Days since Injury)

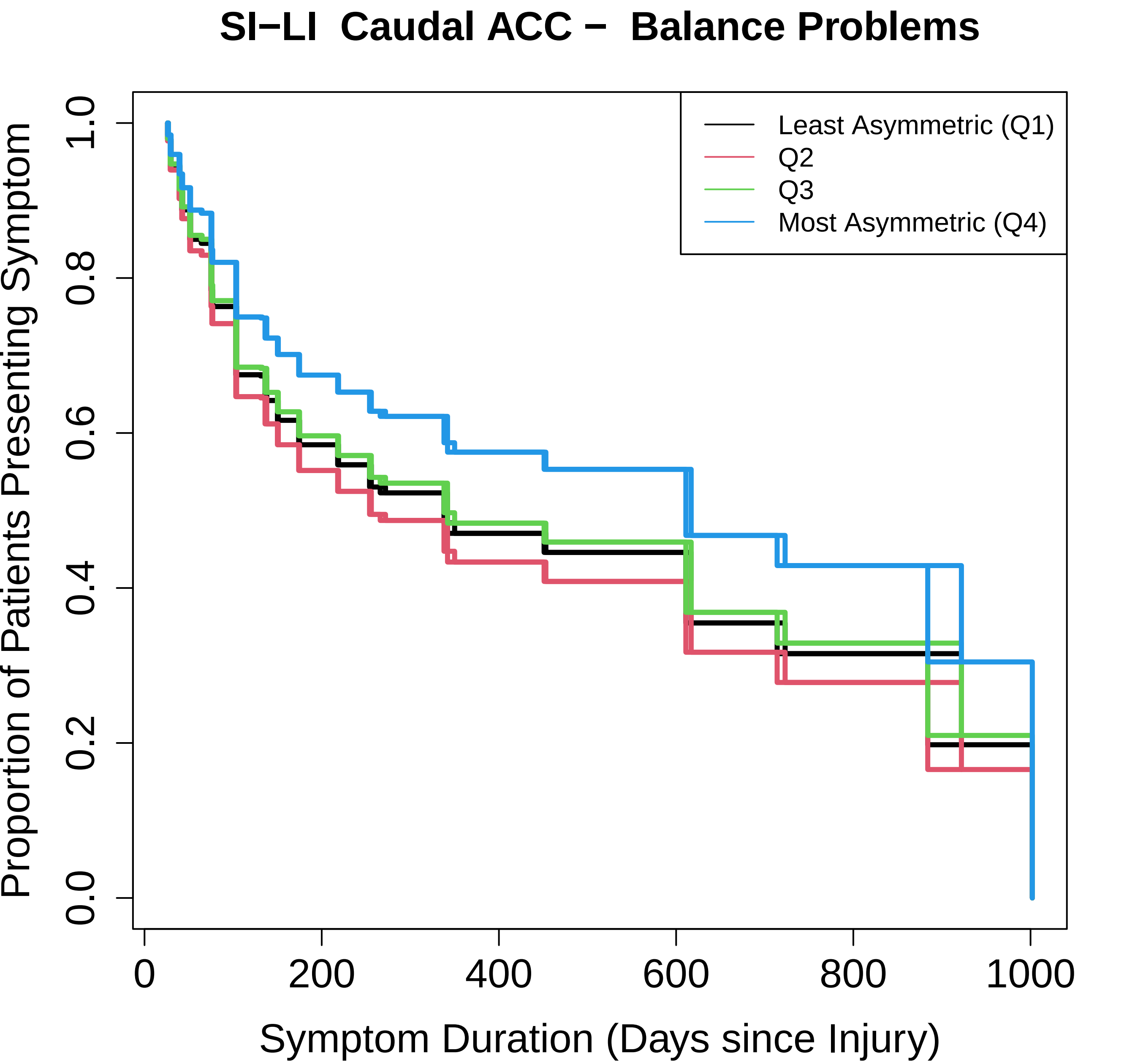
SI−LI Caudal ACC − Balance Problems
Least Asymmetric (Q1)
Q2
Q3
Most Asymmetric (Q4)
Proportion of Patients Presenting Symptom
0.0
0.2
0.4
0.6
0.8
1.0
0
200
400
600
800
1000
Symptom Duration (Days since Injury)

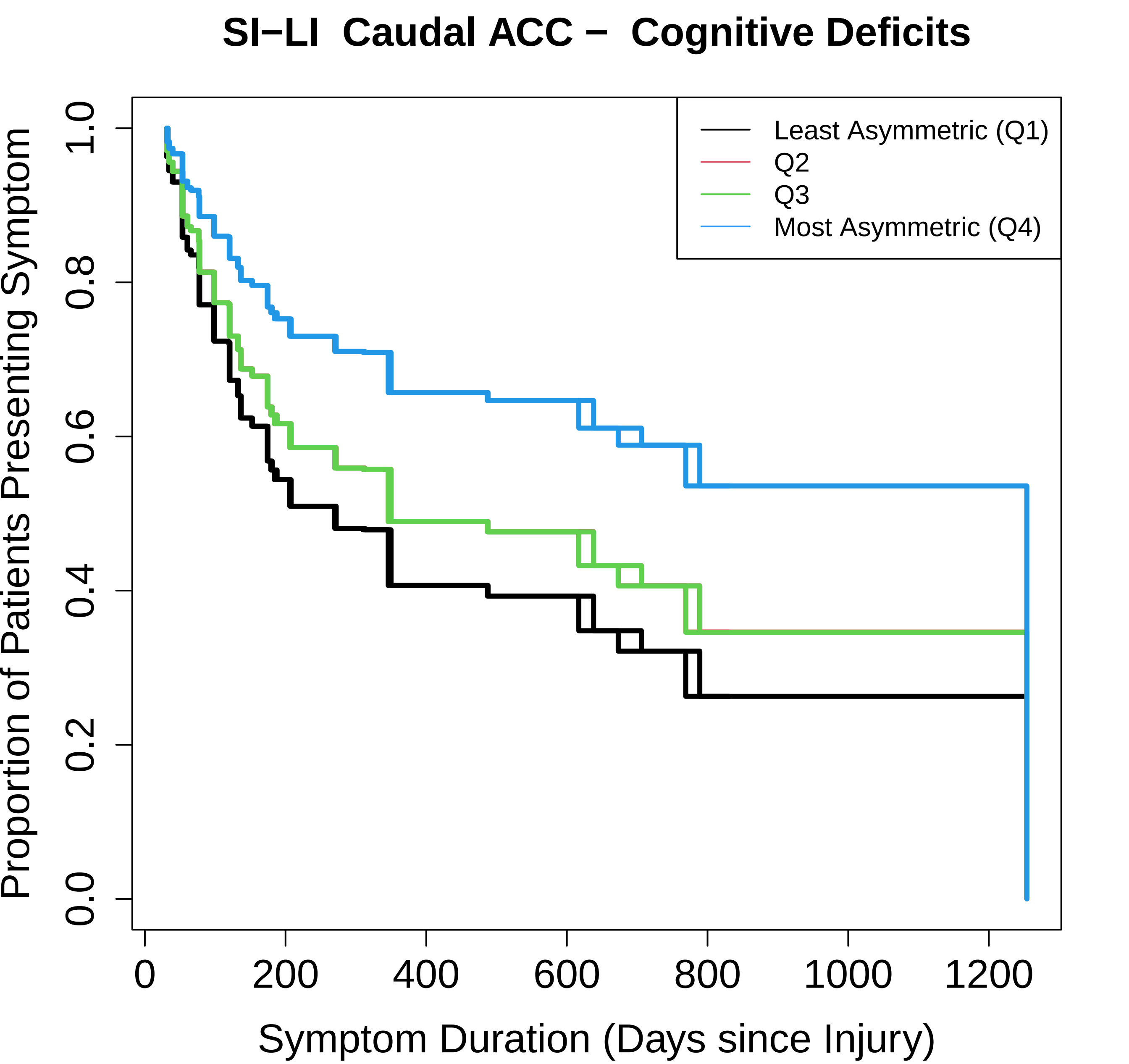
SI−LI Caudal ACC − Cognitive Deficits
Least Asymmetric (Q1)
Q2
Q3
Most Asymmetric (Q4)
Proportion of Patients Presenting Symptom
0.0
0.2
0.4
0.6
0.8
1.0
0
200
400
600
800
1000
1200
Symptom Duration (Days since Injury)

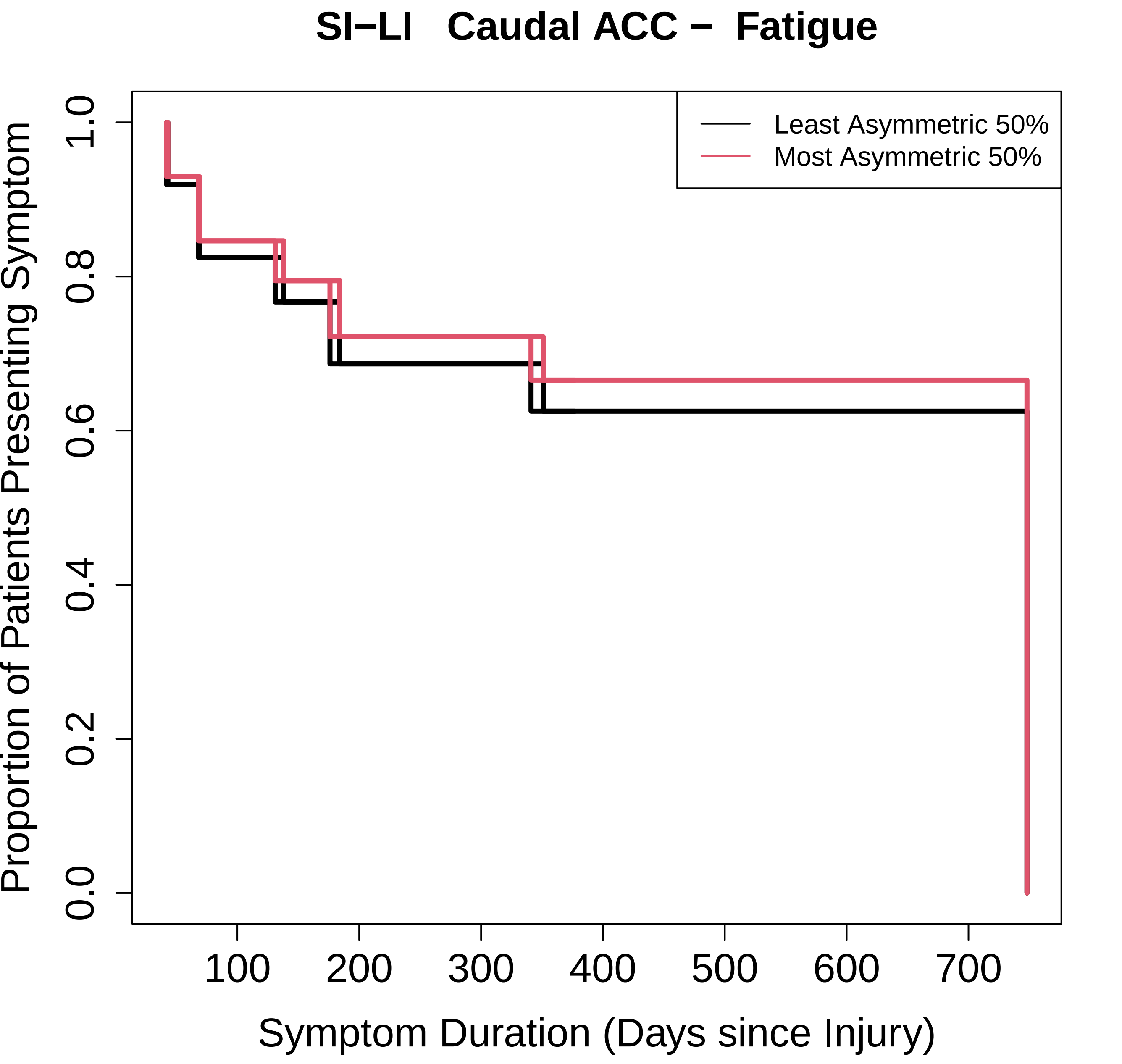

SI−LI Caudal ACC − Fatigue
Least Asymmetric 50%
Most Asymmetric 50%
Proportion of Patients Presenting Symptom
0.0
0.2
0.4
0.6
0.8
1.0
100
200
300
400
500
600
700
Symptom Duration (Days since Injury)

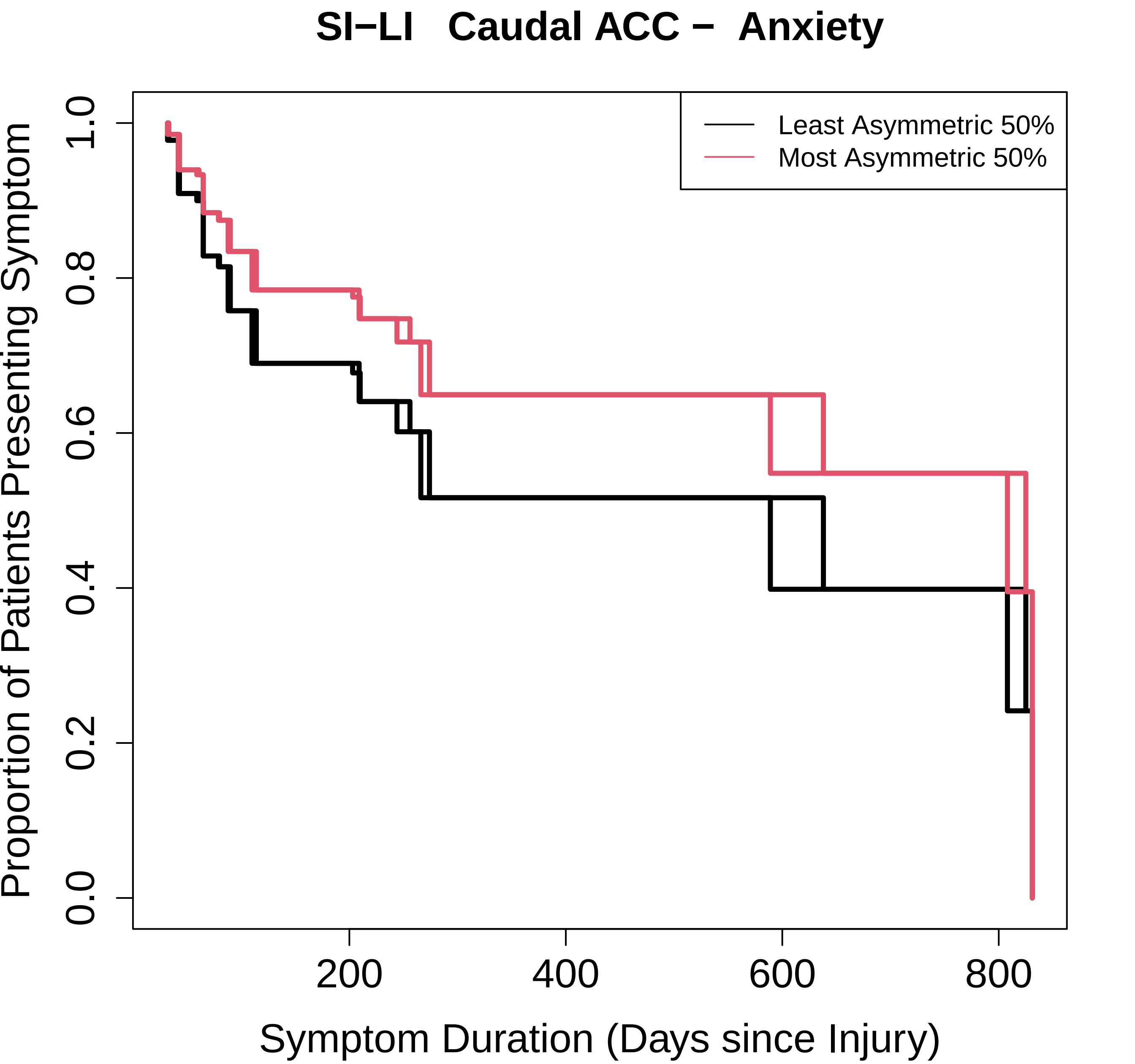

SI−LI Caudal ACC − Anxiety
Least Asymmetric 50%
Most Asymmetric 50%
Proportion of Patients Presenting Symptom
0.0
0.2
0.4
0.6
0.8
1.0
200
400
600
800
Symptom Duration (Days since Injury)

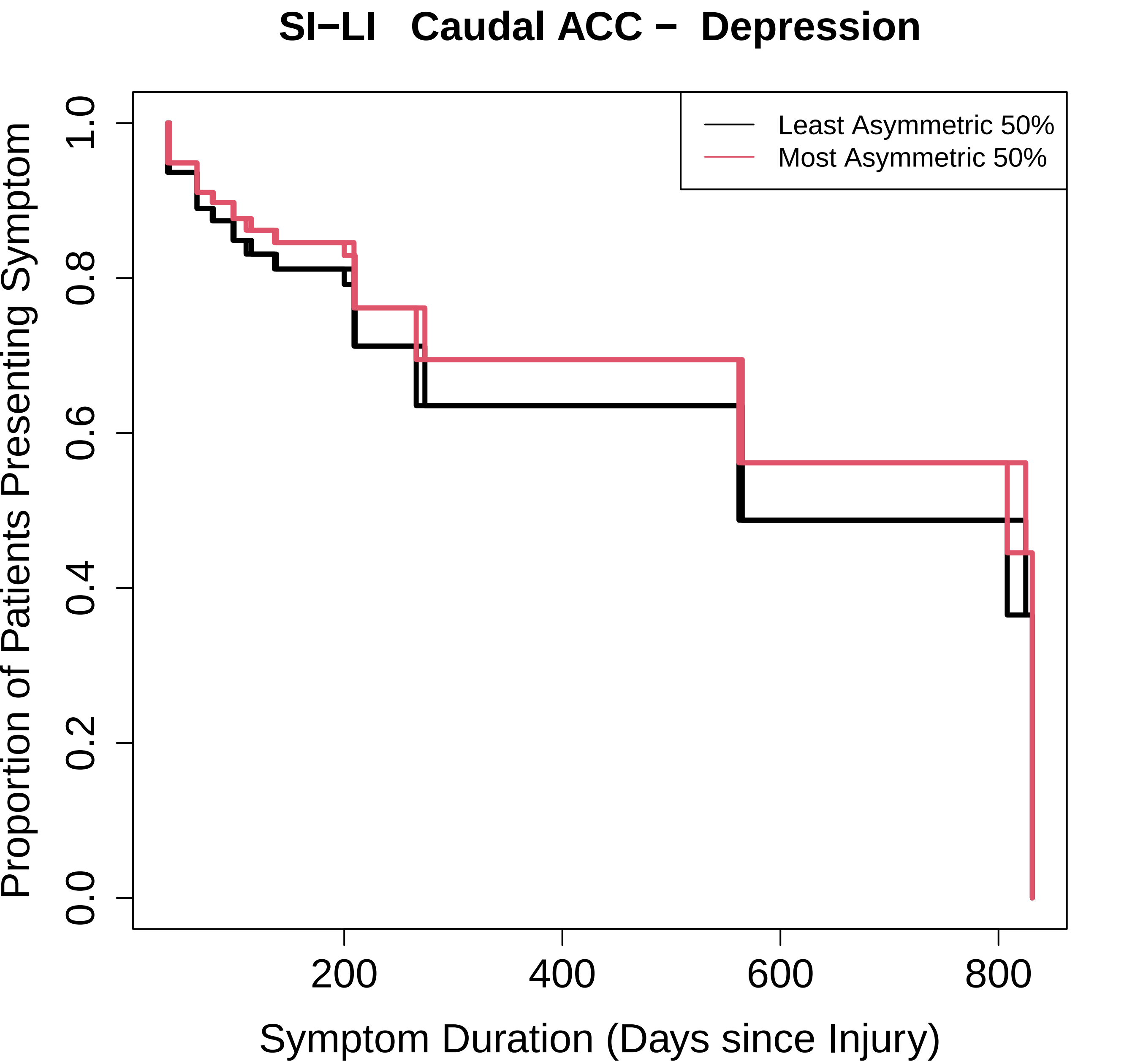
SI−LI Caudal ACC − Depression
Least Asymmetric 50%
Most Asymmetric 50%
Proportion of Patients Presenting Symptom
0.0
0.2
0.4
0.6
0.8
1.0
200
400
600
800
Symptom Duration (Days since Injury)

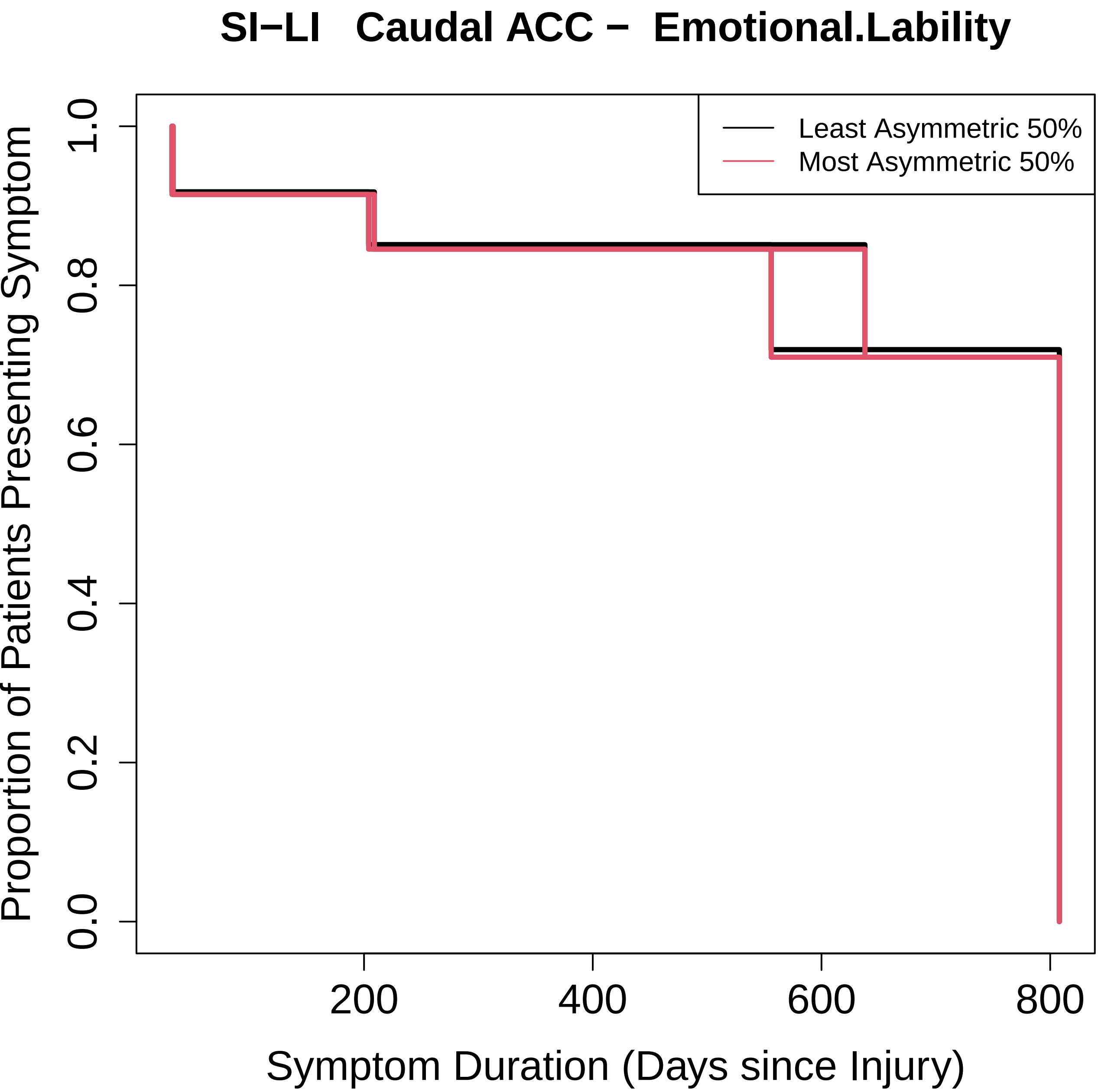

SI−LI Caudal ACC − Emotional.Lability
Least Asymmetric 50%
Most Asymmetric 50%
Proportion of Patients Presenting Symptom
0.0
0.2
0.4
0.6
0.8
1.0
200
400
600
800
Symptom Duration (Days since Injury)

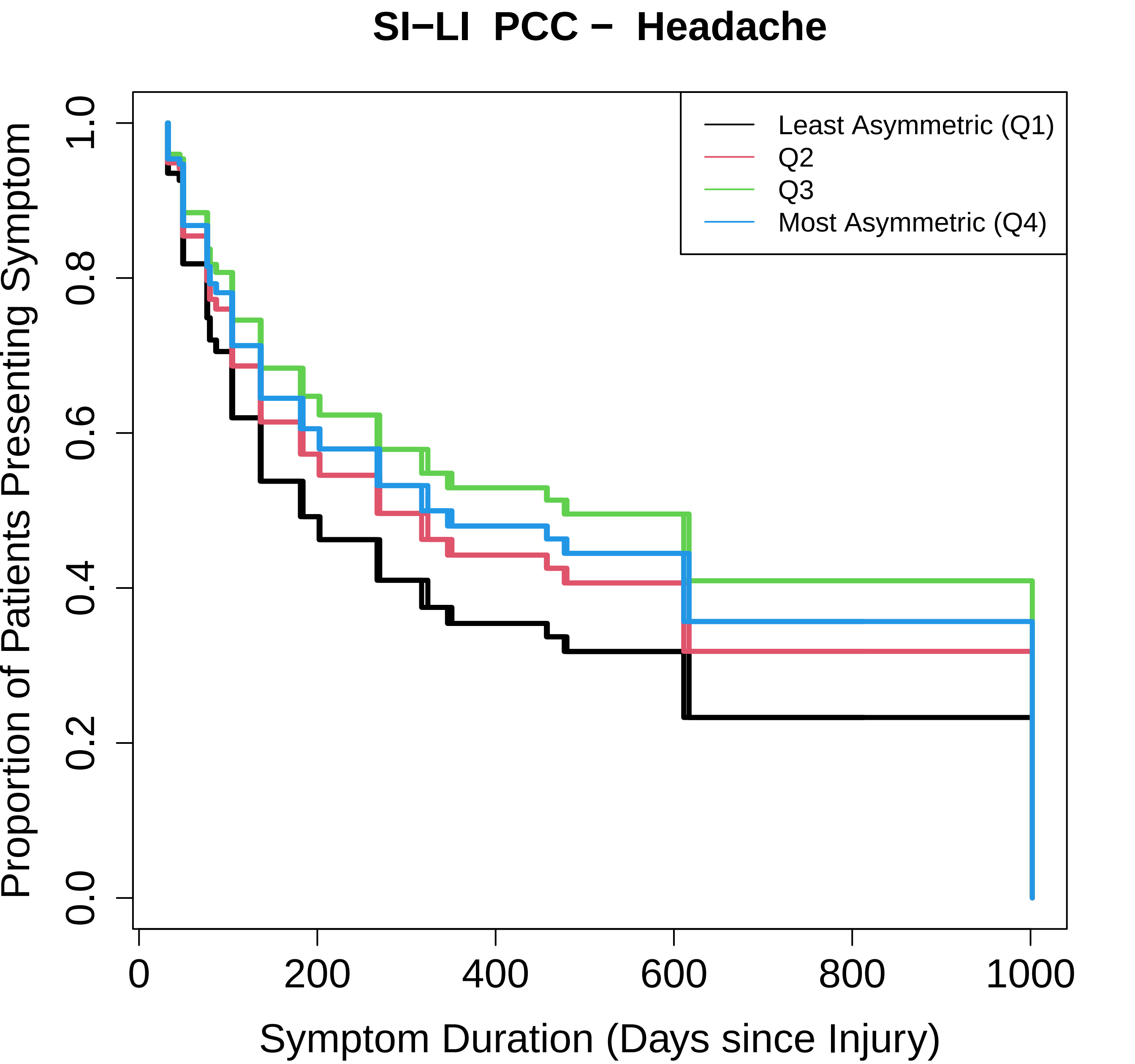
SI−LI PCC − Headache
Least Asymmetric (Q1)
Q2
Q3
Most Asymmetric (Q4)
Proportion of Patients Presenting Symptom
0.0
0.2
0.4
0.6
0.8
1.0
0
200
400
600
800
1000
Symptom Duration (Days since Injury)

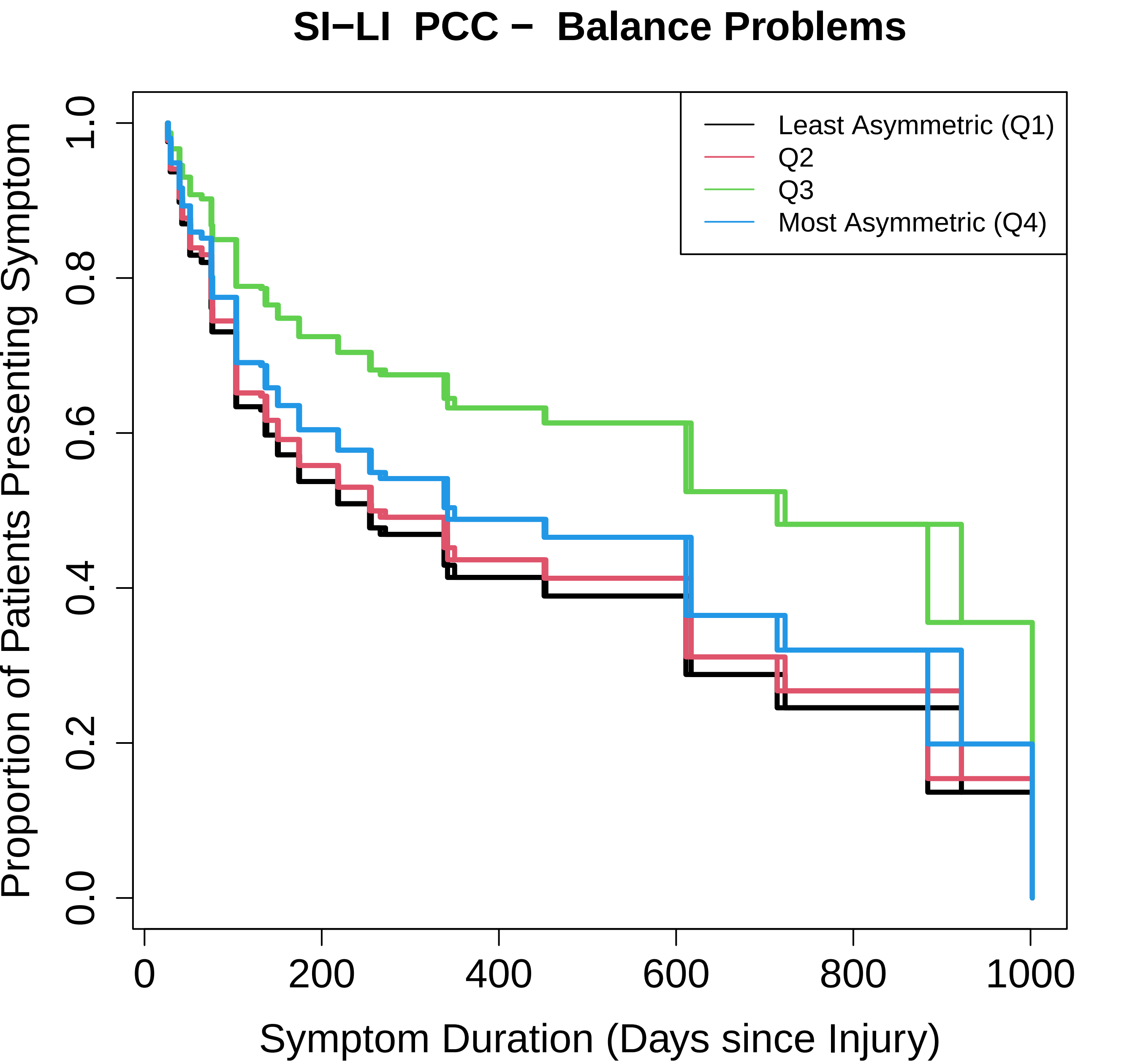
SI−LI PCC − Balance Problems
Least Asymmetric (Q1)
Q2
Q3
Most Asymmetric (Q4)
Proportion of Patients Presenting Symptom
0.0
0.2
0.4
0.6
0.8
1.0
0
200
400
600
800
1000
Symptom Duration (Days since Injury)

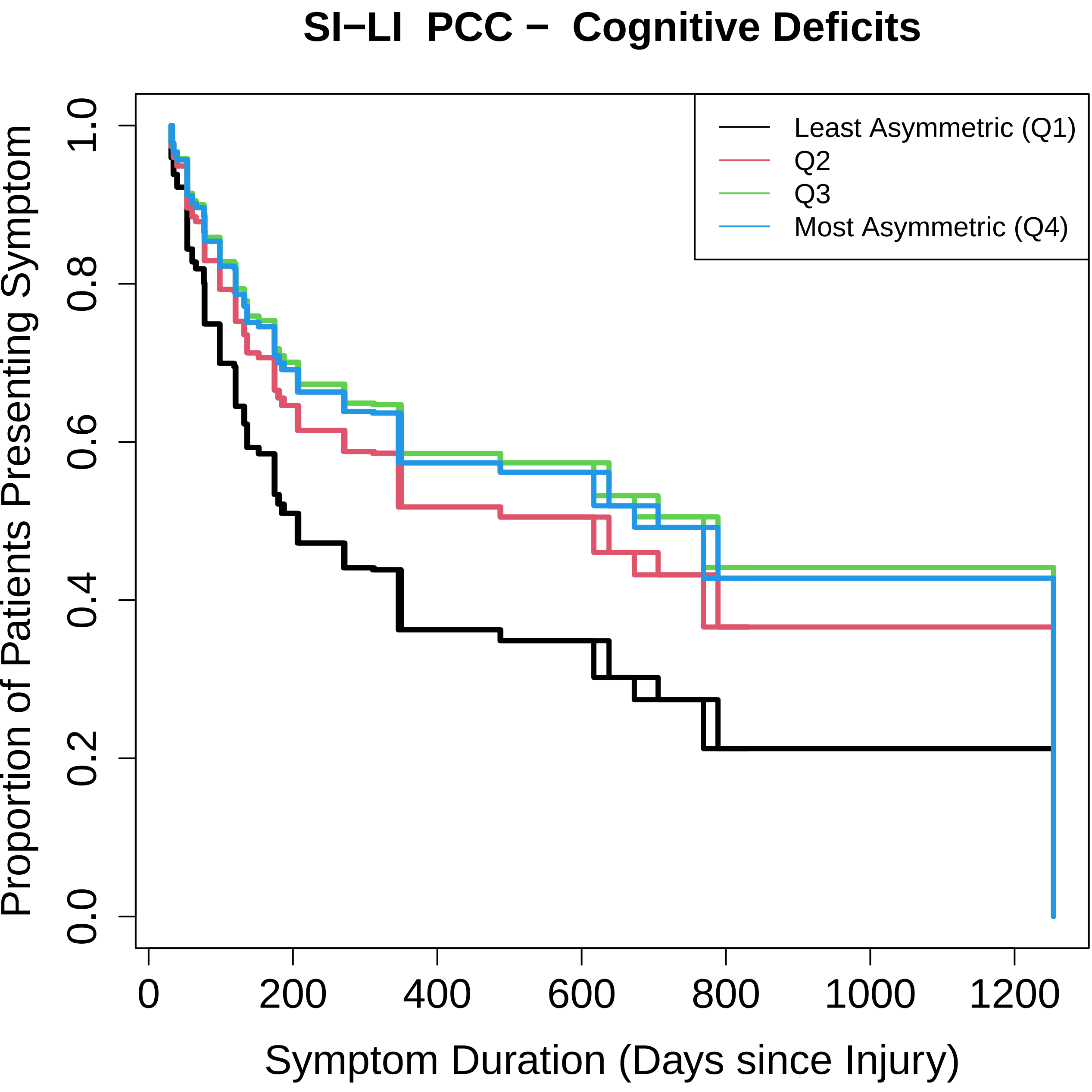

SI−LI PCC − Cognitive Deficits
Proportion of Patients Presenting Symptom
0.0
0.2
0.4
0.6
0.8
1.0
0
200
400
600
800
1000
1200
Symptom Duration (Days since Injury)
Least Asymmetric (Q1)
Q2
Q3
Most Asymmetric (Q4)

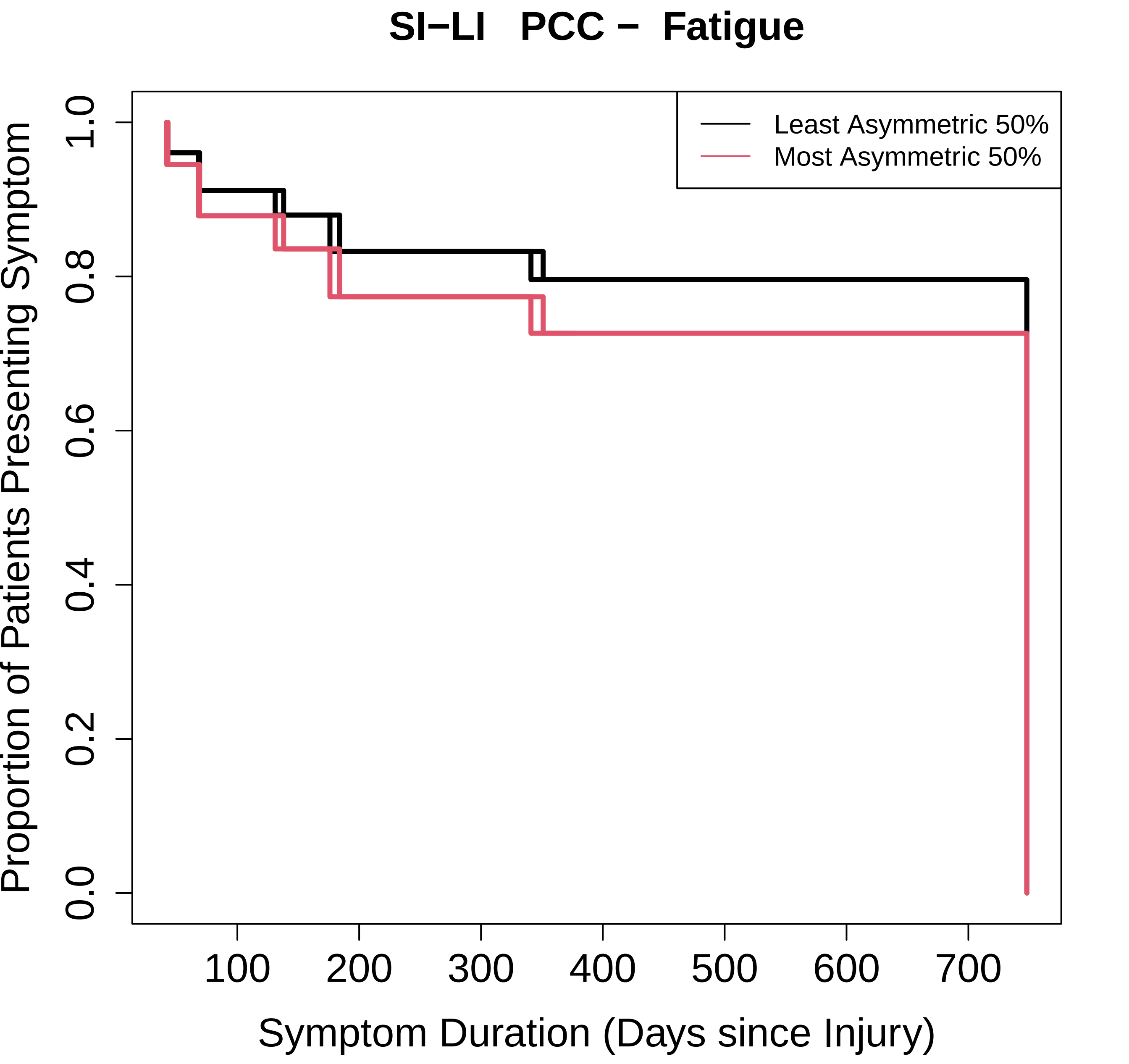
SI−LI PCC − Fatigue
Least Asymmetric 50%
Most Asymmetric 50%
Proportion of Patients Presenting Symptom
0.0
0.2
0.4
0.6
0.8
1.0
100
200
300
400
500
600
700
Symptom Duration (Days since Injury)

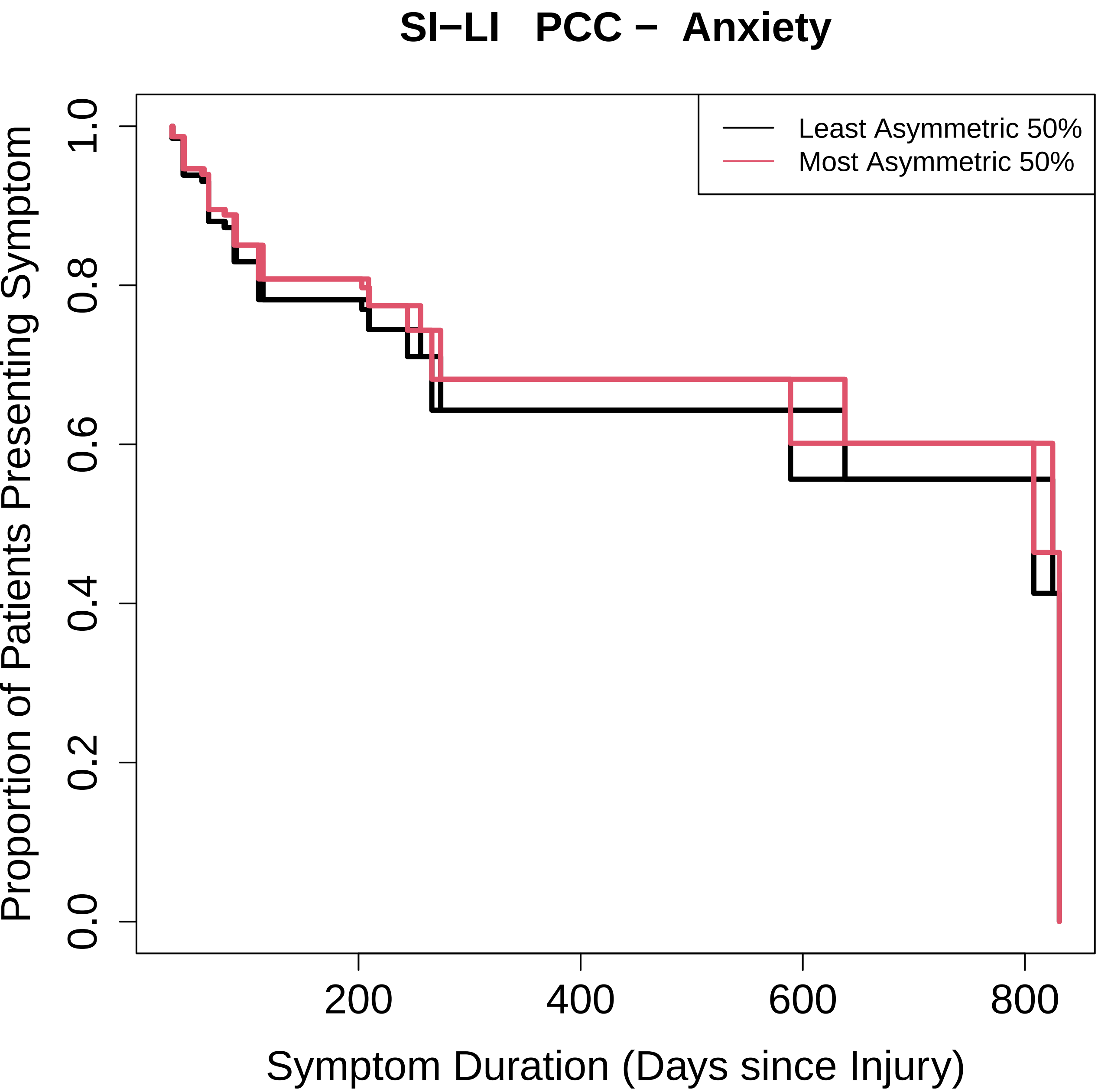

SI−LI PCC − Anxiety
Least Asymmetric 50%
Most Asymmetric 50%
Proportion of Patients Presenting Symptom
0.0
0.2
0.4
0.6
0.8
1.0
200
400
600
800
Symptom Duration (Days since Injury)

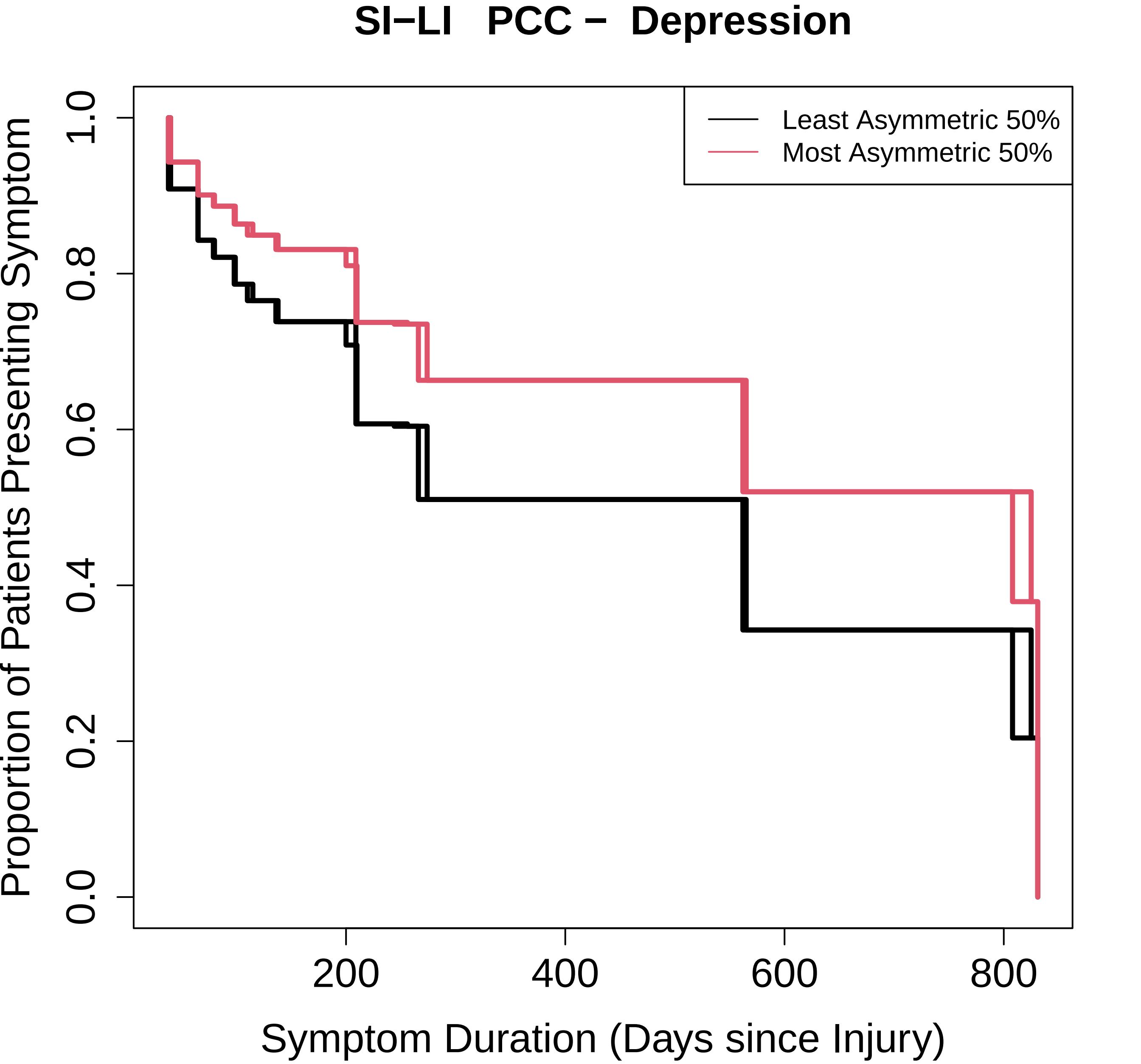
SI−LI PCC − Depression
Least Asymmetric 50%
Most Asymmetric 50%
Proportion of Patients Presenting Symptom
0.0
0.2
0.4
0.6
0.8
1.0
200
400
600
800
Symptom Duration (Days since Injury)

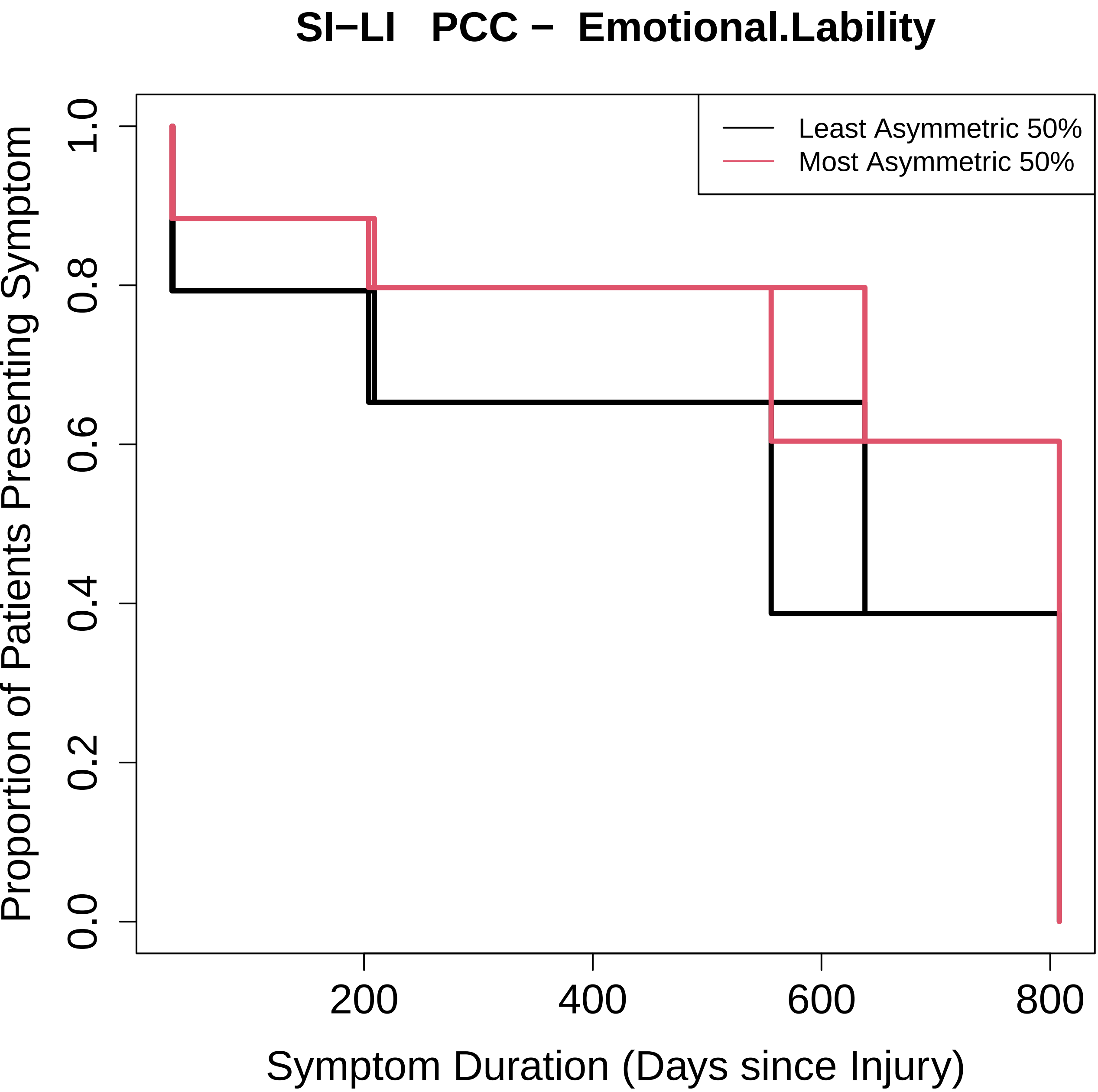
SI−LI PCC − Emotional.Lability
Least Asymmetric 50%
Most Asymmetric 50%
Proportion of Patients Presenting Symptom
0.0
0.2
0.4
0.6
0.8
1.0
200
400
600
800
Symptom Duration (Days since Injury)

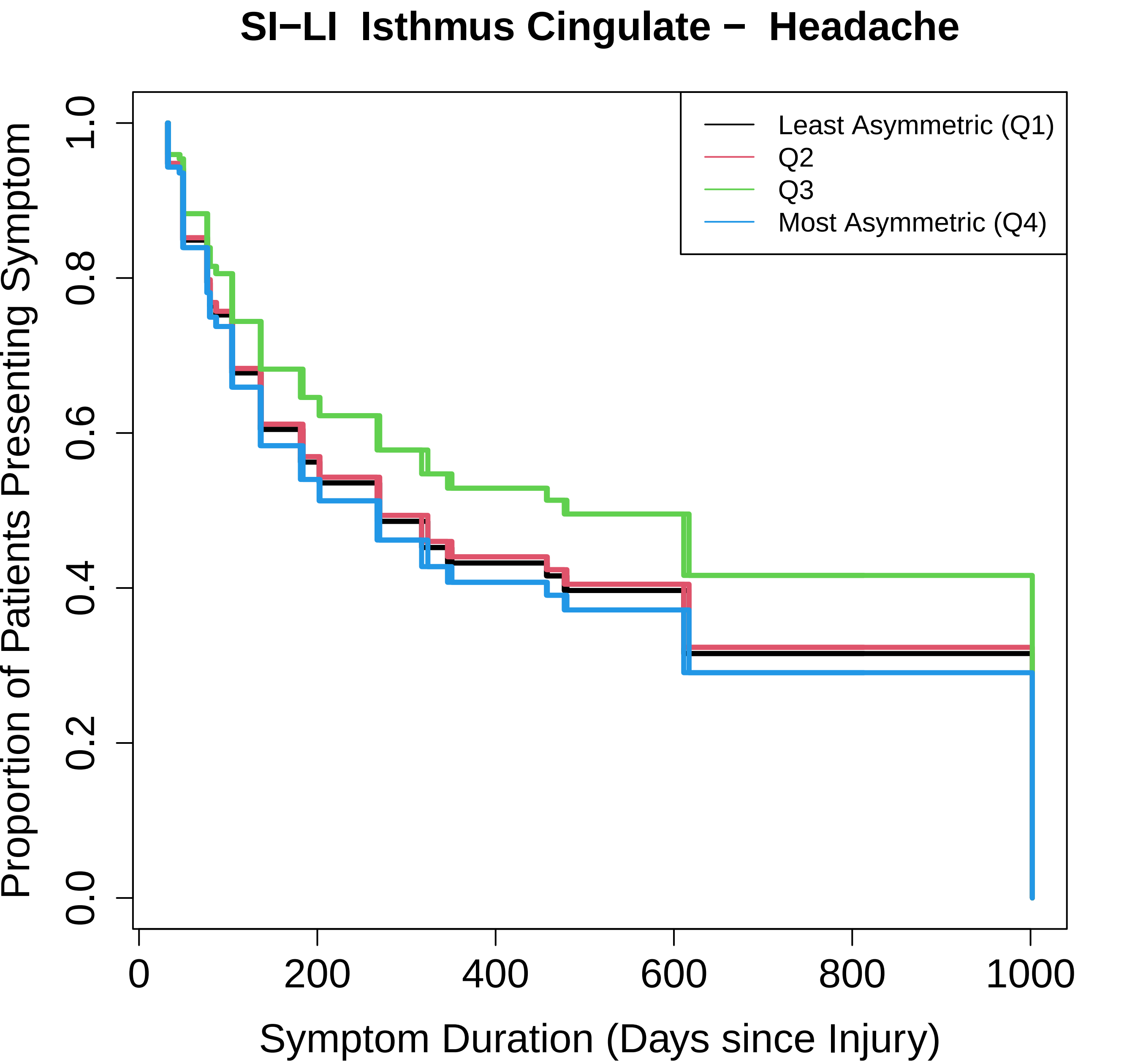

SI−LI Isthmus Cingulate − Headache
Least Asymmetric (Q1)
Q2
Q3
Most Asymmetric (Q4)
Proportion of Patients Presenting Symptom
0.0
0.2
0.4
0.6
0.8
1.0
0
200
400
600
800
1000
Symptom Duration (Days since Injury)

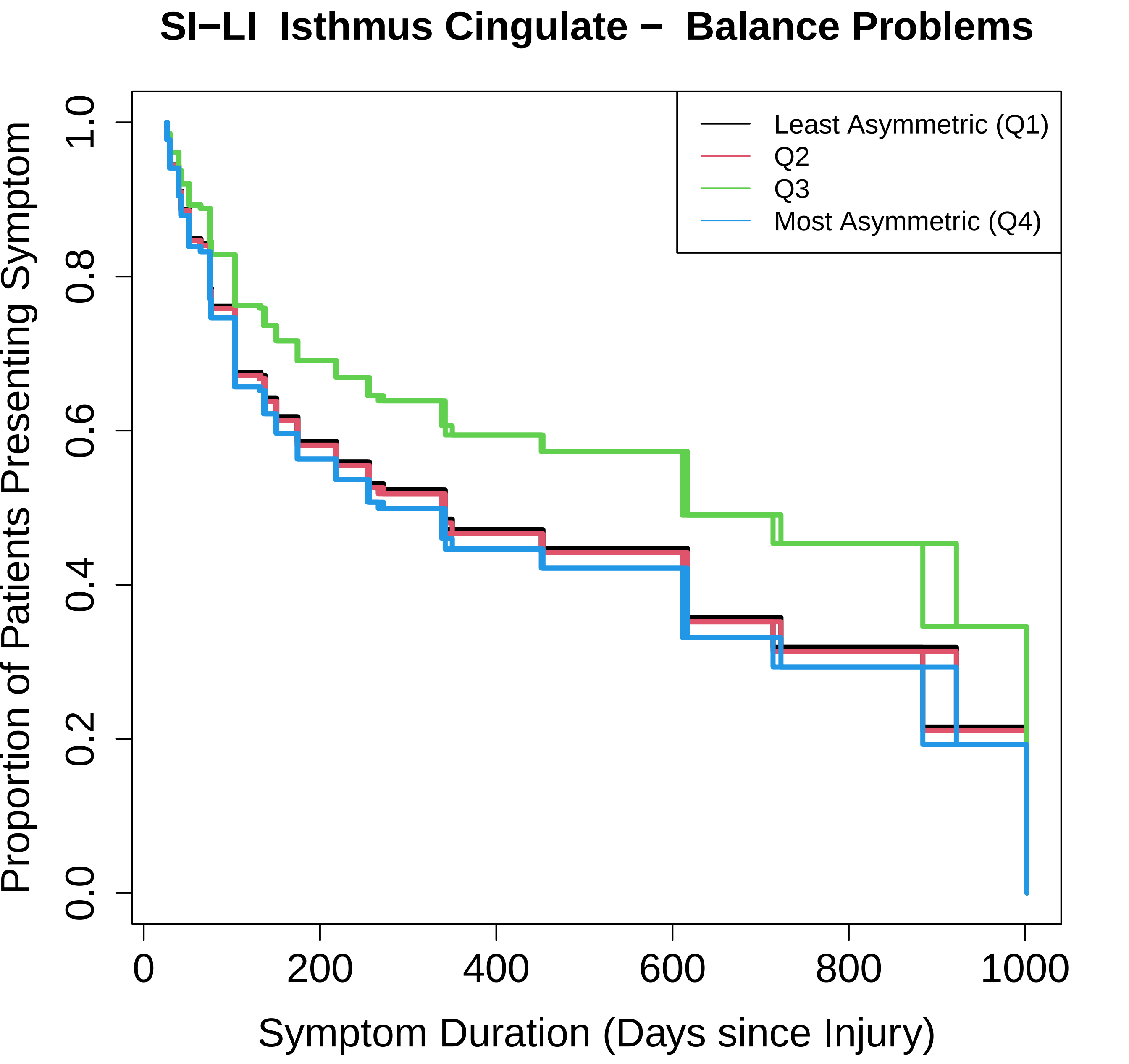
SI−LI Isthmus Cingulate − Balance Problems
Least Asymmetric (Q1)
Q2
Q3
Most Asymmetric (Q4)
Proportion of Patients Presenting Symptom
0.0
0.2
0.4
0.6
0.8
1.0
0
200
400
600
800
1000
Symptom Duration (Days since Injury)

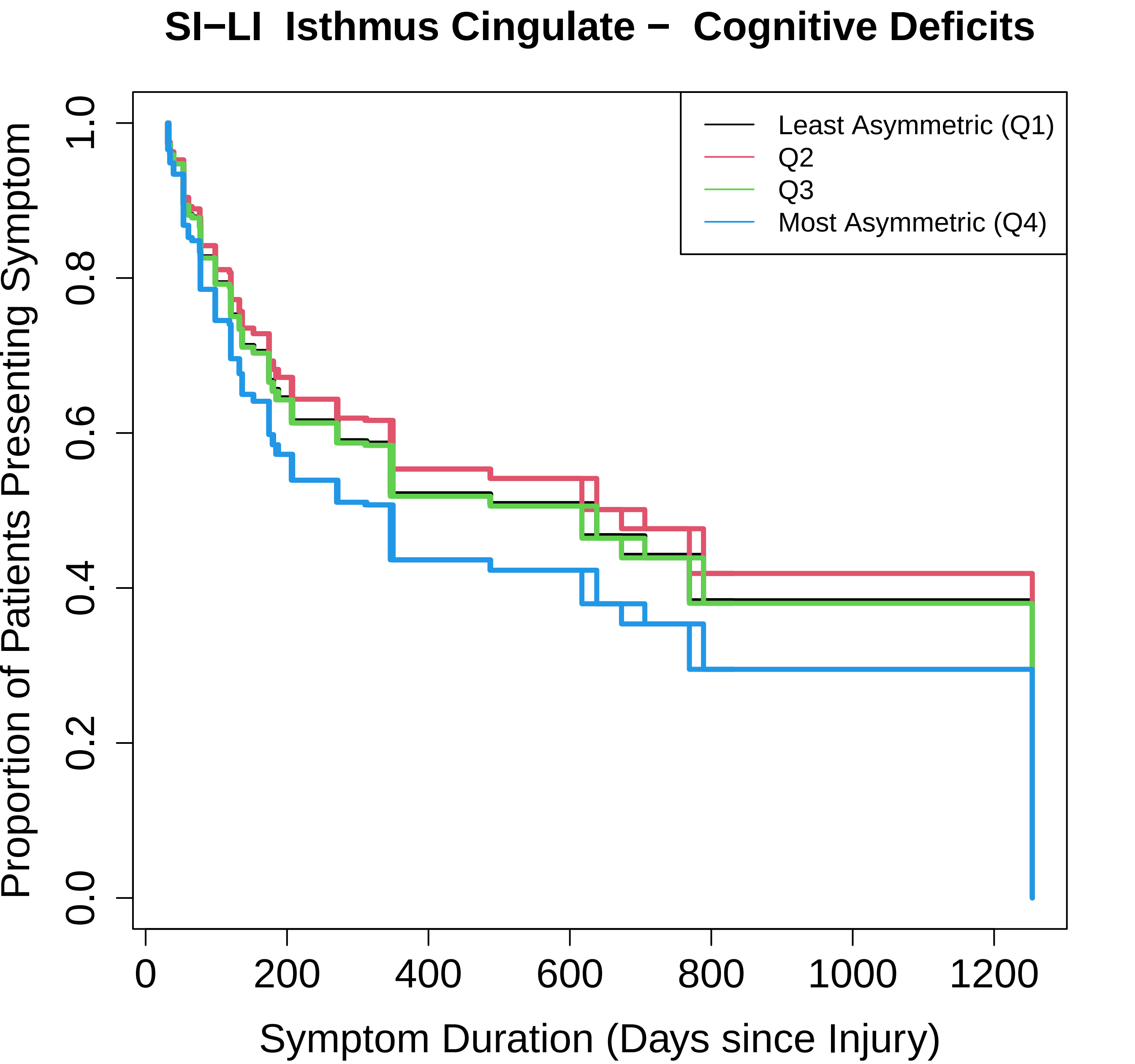
SI−LI Isthmus Cingulate − Cognitive Deficits
Least Asymmetric (Q1)
Q2
Q3
Most Asymmetric (Q4)
Proportion of Patients Presenting Symptom
0.0
0.2
0.4
0.6
0.8
1.0
0
200
400
600
800
1000
1200
Symptom Duration (Days since Injury)

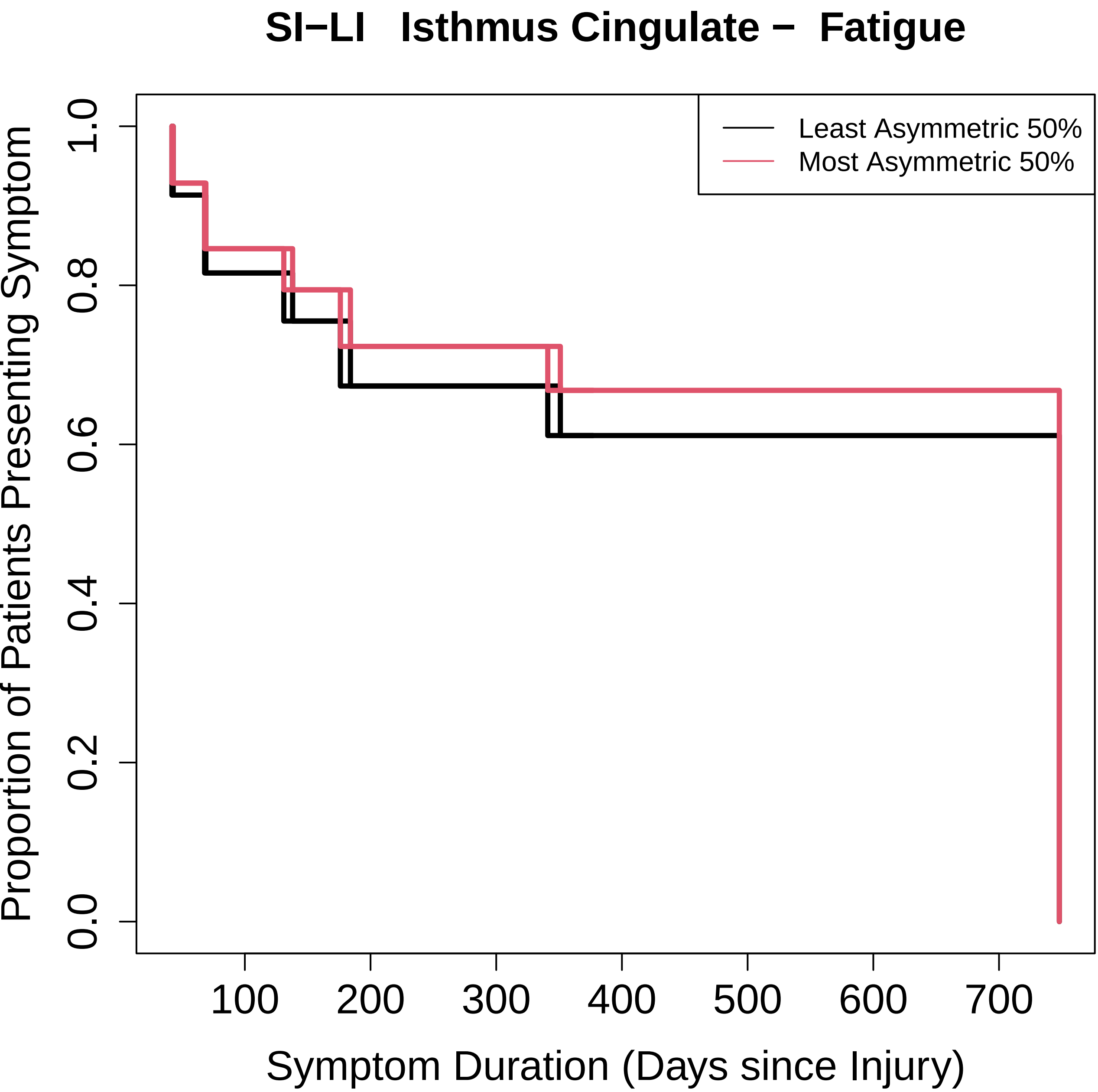

SI−LI Isthmus Cingulate − Fatigue
Least Asymmetric 50%
Most Asymmetric 50%
Proportion of Patients Presenting Symptom
0.0
0.2
0.4
0.6
0.8
1.0
100
200
300
400
500
600
700
Symptom Duration (Days since Injury)

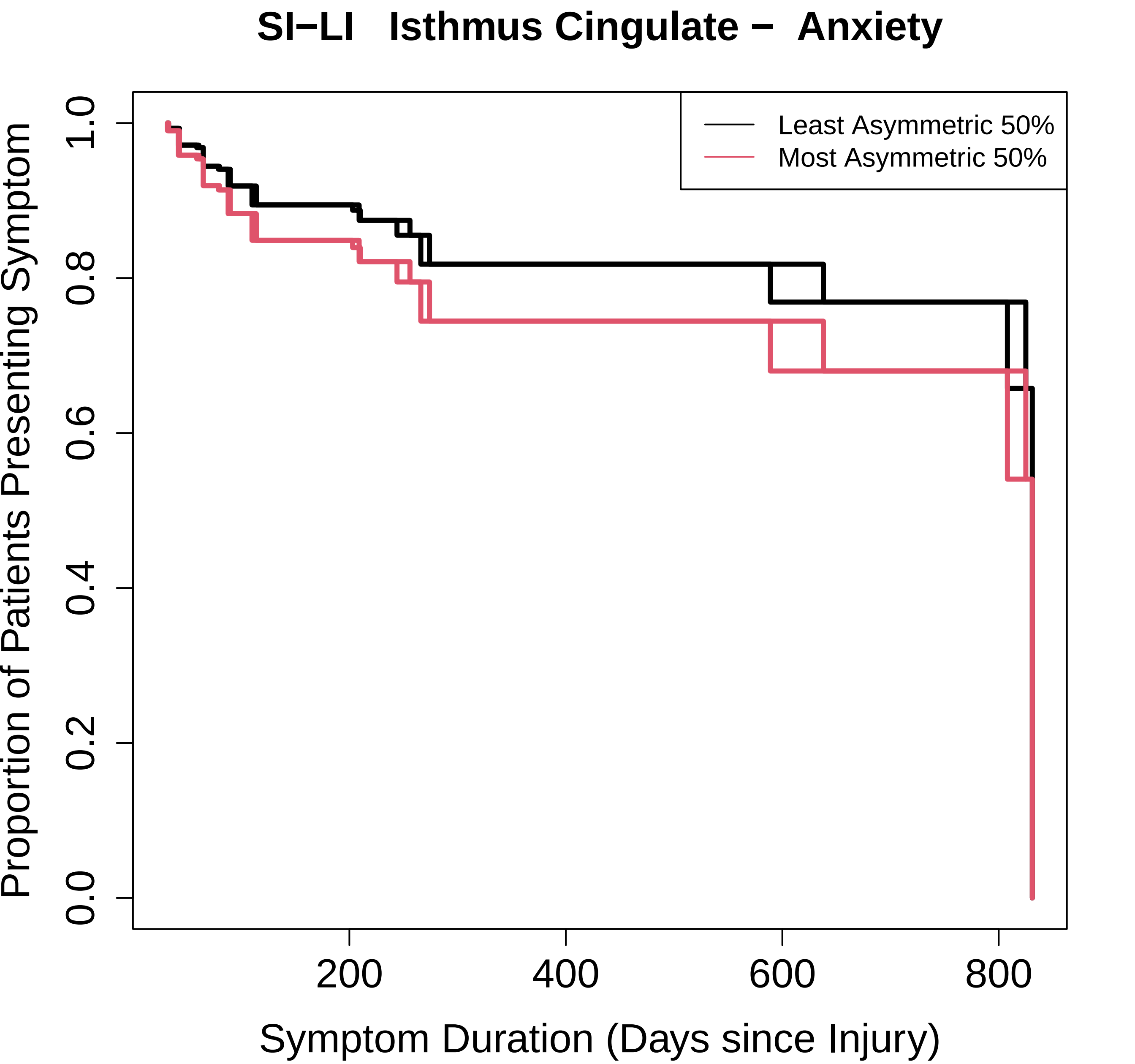
SI−LI Isthmus Cingulate − Anxiety
Least Asymmetric 50%
Most Asymmetric 50%
Proportion of Patients Presenting Symptom
0.0
0.2
0.4
0.6
0.8
1.0
200
400
600
800
Symptom Duration (Days since Injury)

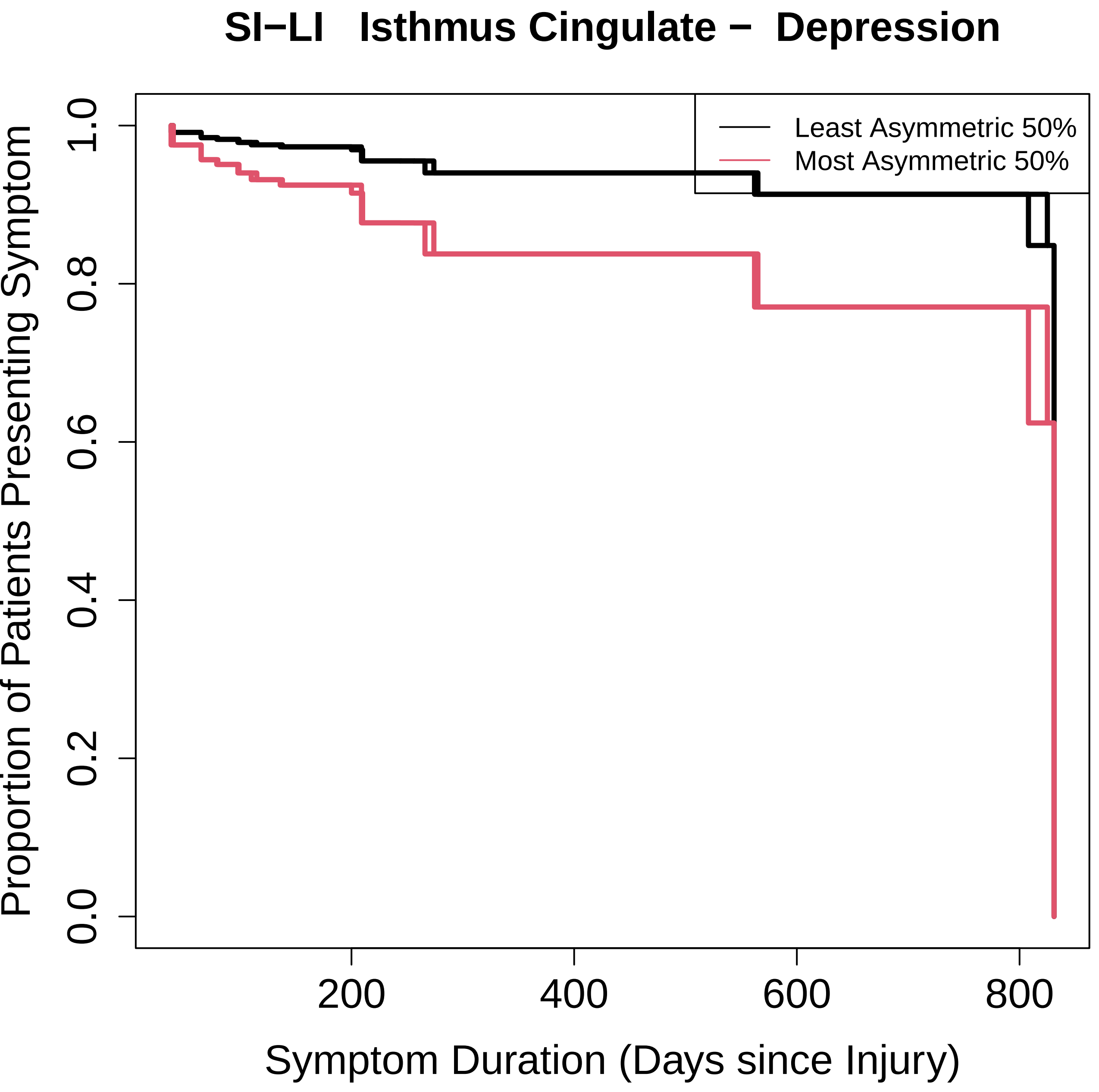
SI−LI Isthmus Cingulate − Depression
Least Asymmetric 50%
Most Asymmetric 50%
Proportion of Patients Presenting Symptom
0.0
0.2
0.4
0.6
0.8
1.0
200
400
600
800
Symptom Duration (Days since Injury)

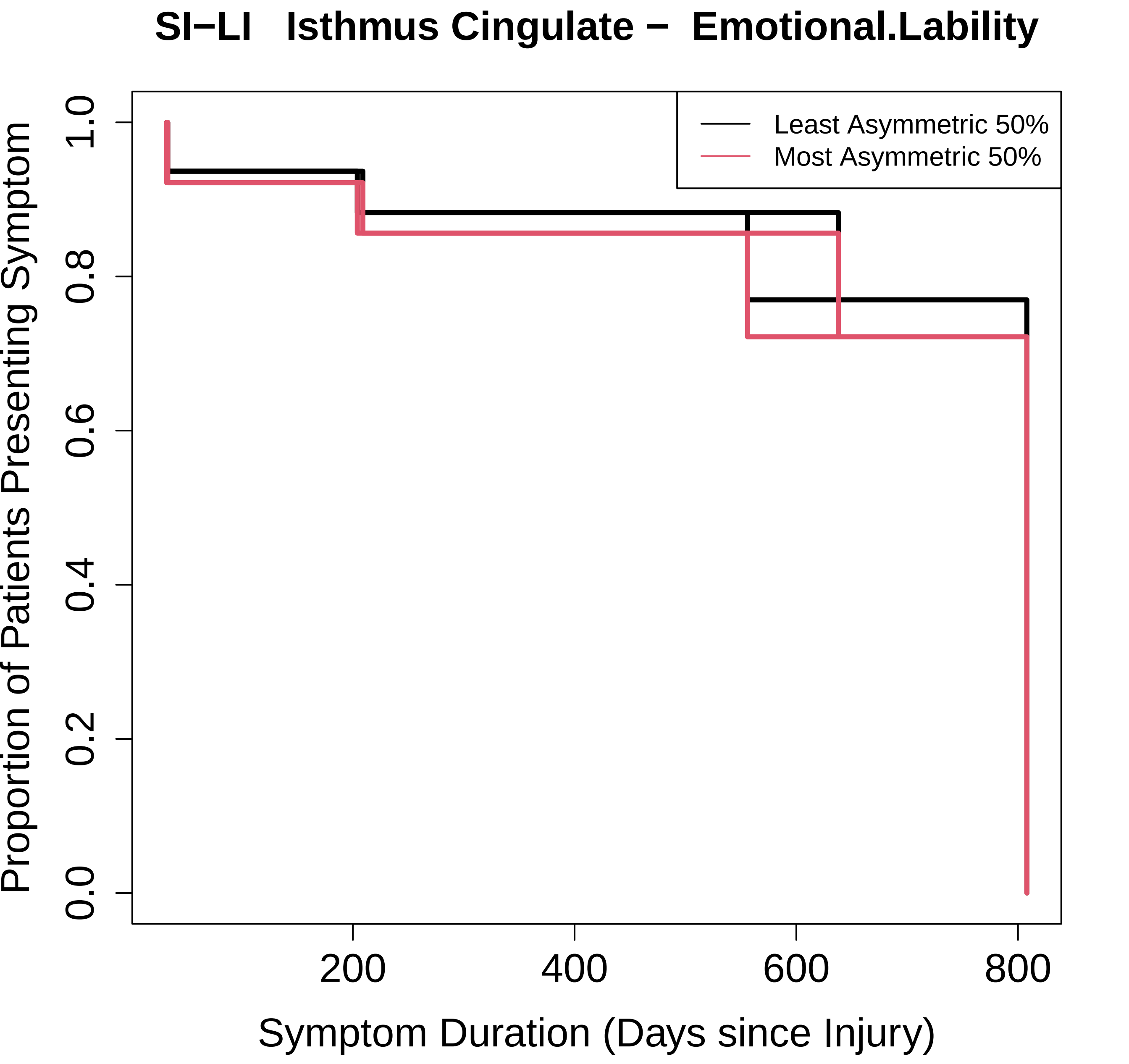

SI−LI Isthmus Cingulate − Emotional.Lability
Least Asymmetric 50%
Most Asymmetric 50%
Proportion of Patients Presenting Symptom
0.0
0.2
0.4
0.6
0.8
1.0
200
400
600
800
Symptom Duration (Days since Injury)

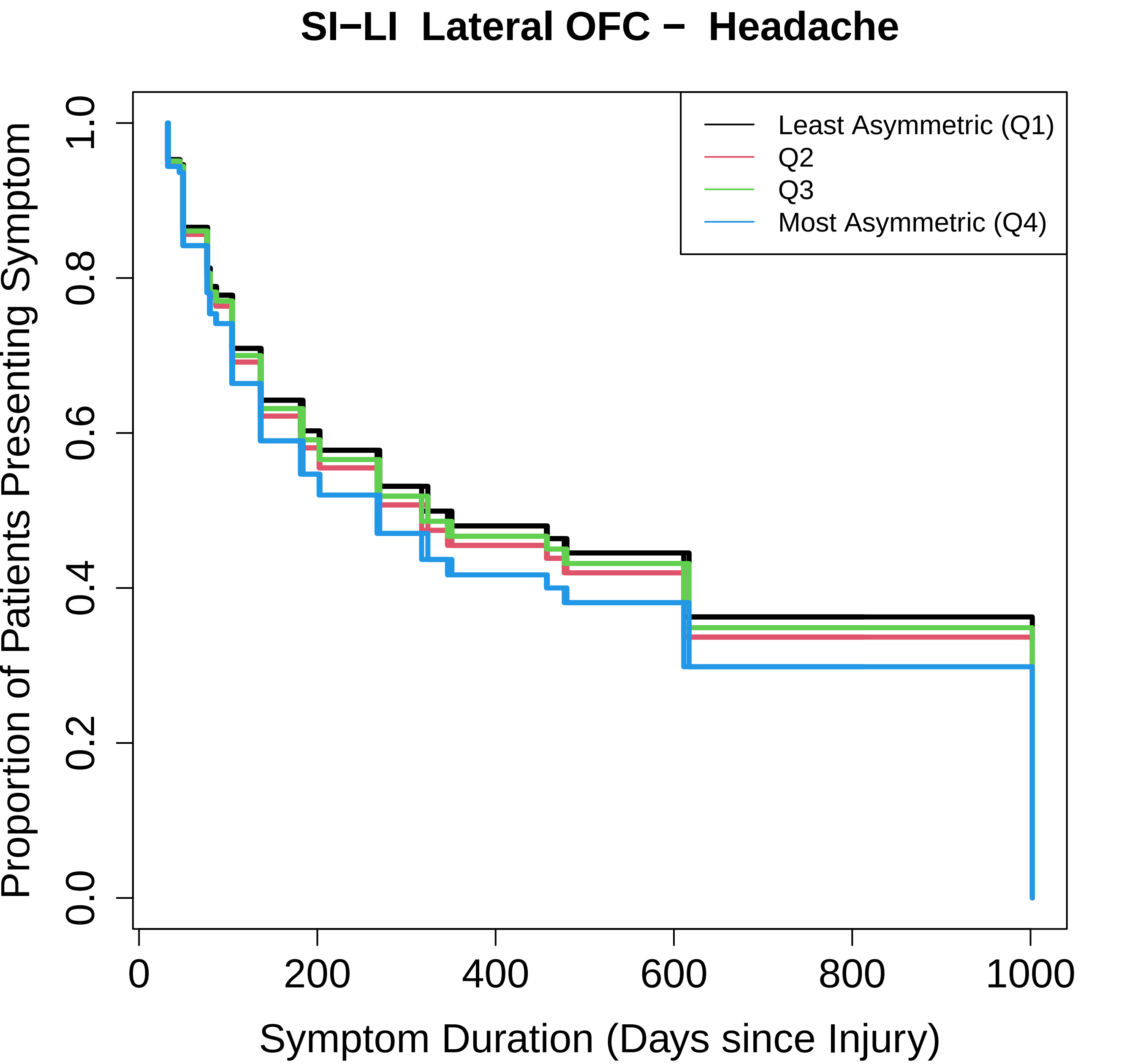
SI−LI Lateral OFC − Headache
Least Asymmetric (Q1)
Q2
Q3
Most Asymmetric (Q4)
Proportion of Patients Presenting Symptom
0.0
0.2
0.4
0.6
0.8
1.0
0
200
400
600
800
1000
Symptom Duration (Days since Injury)

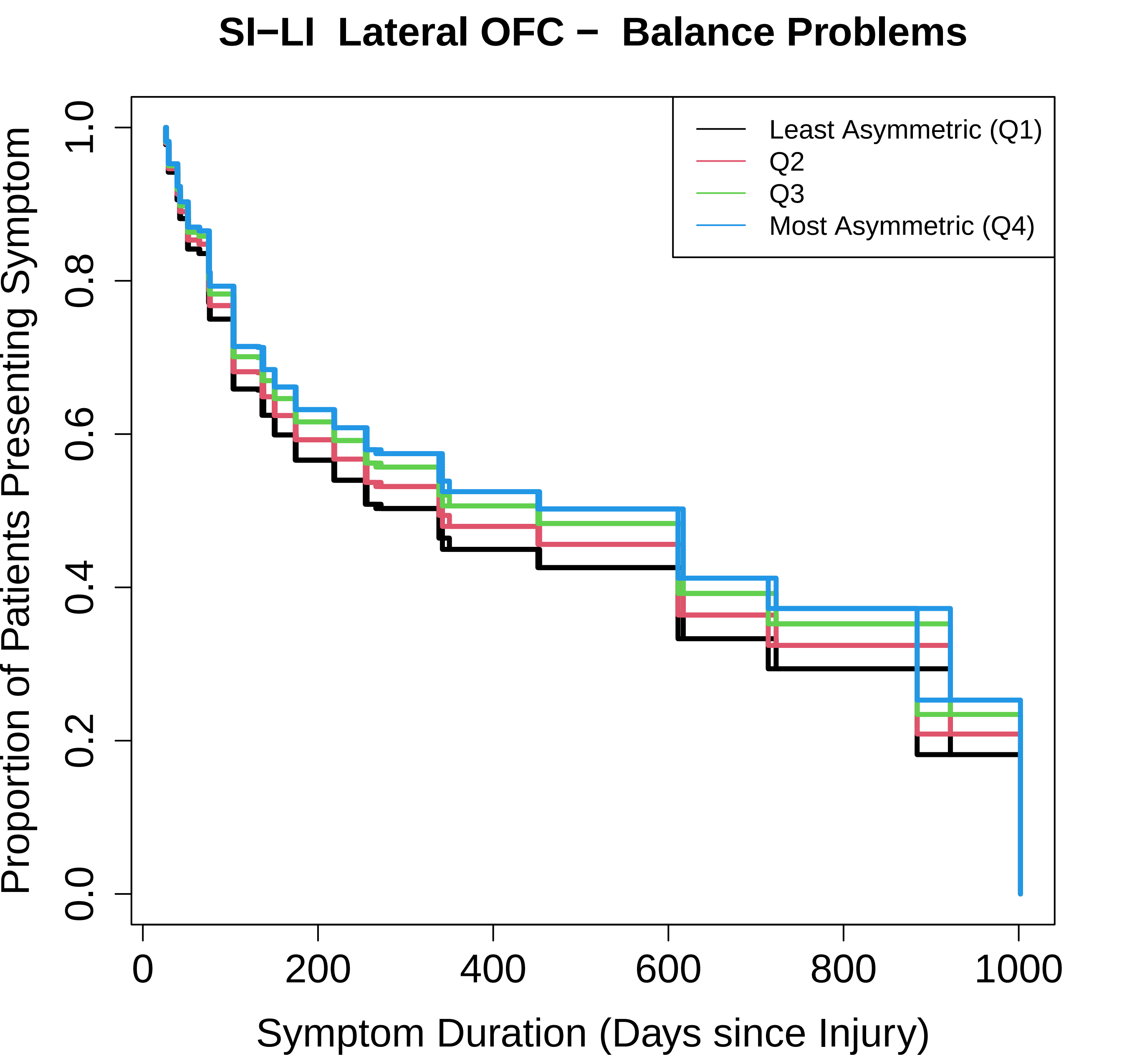

SI−LI Lateral OFC − Balance Problems
Least Asymmetric (Q1)
Q2
Q3
Most Asymmetric (Q4)
Proportion of Patients Presenting Symptom
1.0
0.8
0.6
0.4
0.2
0.0
0
200
400
600
800
1000
Symptom Duration (Days since Injury)

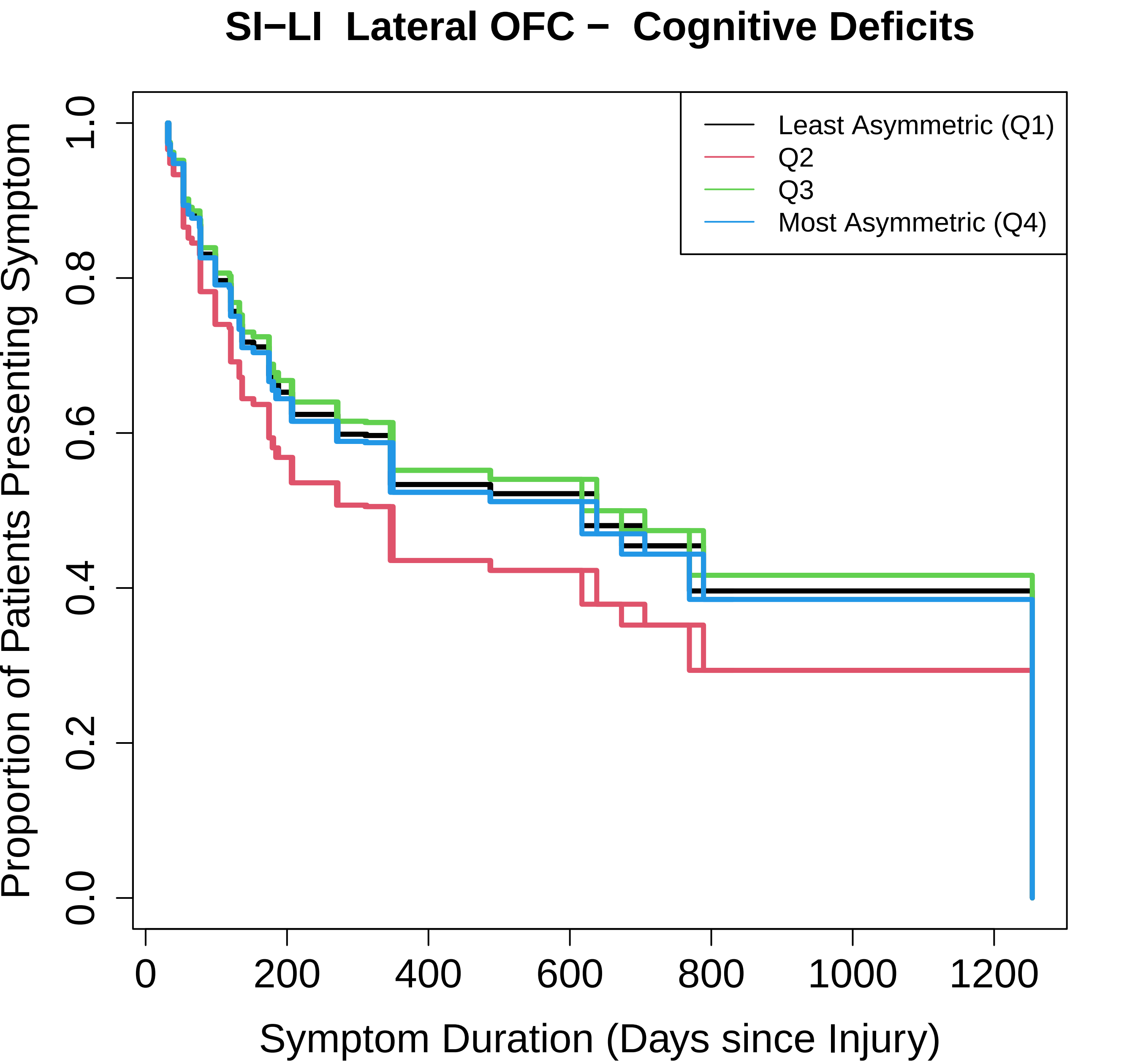
SI−LI Lateral OFC − Cognitive Deficits
Least Asymmetric (Q1)
Q2
Q3
Most Asymmetric (Q4)
Proportion of Patients Presenting Symptom
0.0
0.2
0.4
0.6
0.8
1.0
0
200
400
600
800
1000
1200
Symptom Duration (Days since Injury)

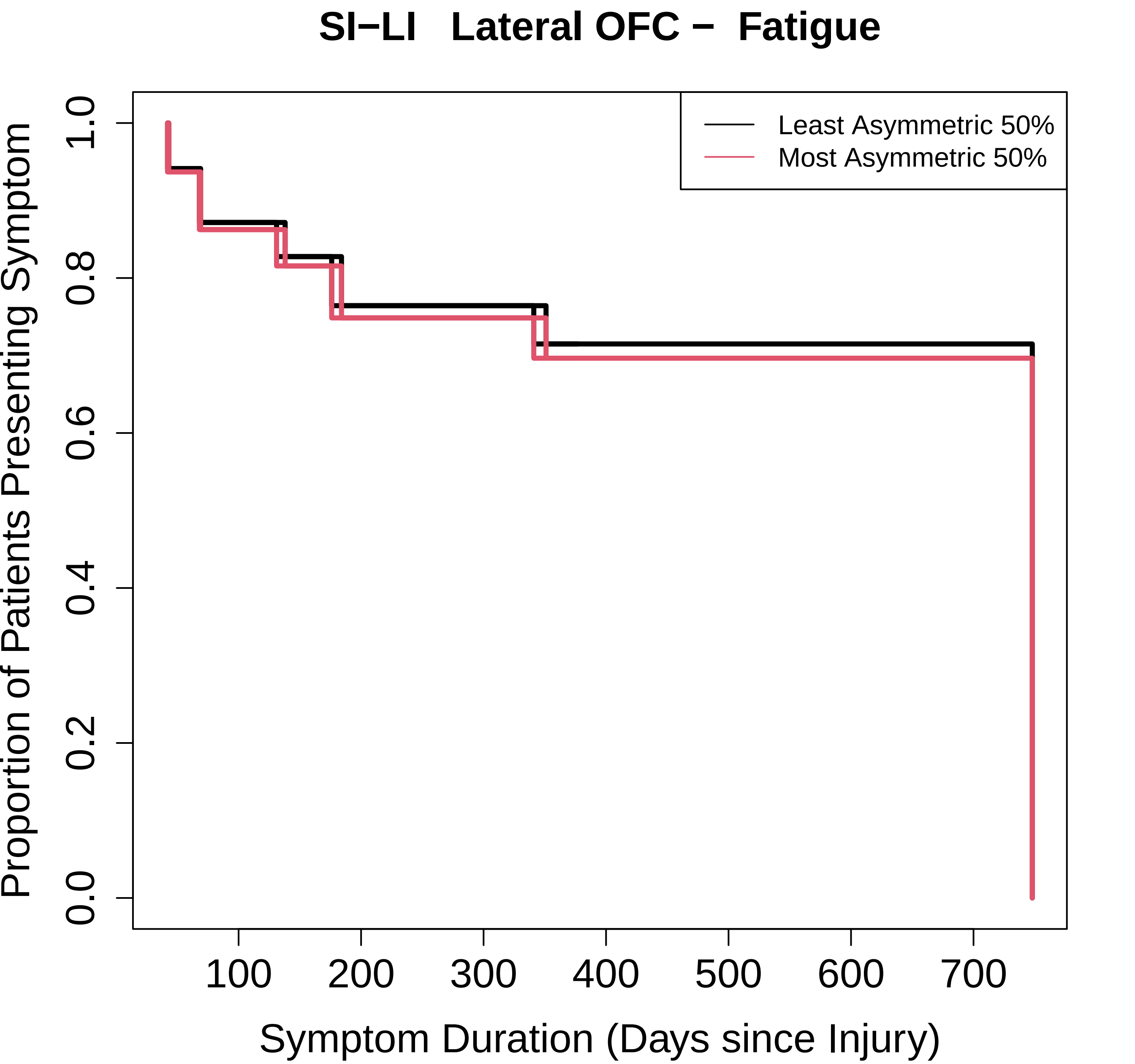
SI−LI Lateral OFC − Fatigue
Least Asymmetric 50%
Most Asymmetric 50%
Proportion of Patients Presenting Symptom
0.0
0.2
0.4
0.6
0.8
1.0
100
200
300
400
500
600
700
Symptom Duration (Days since Injury)

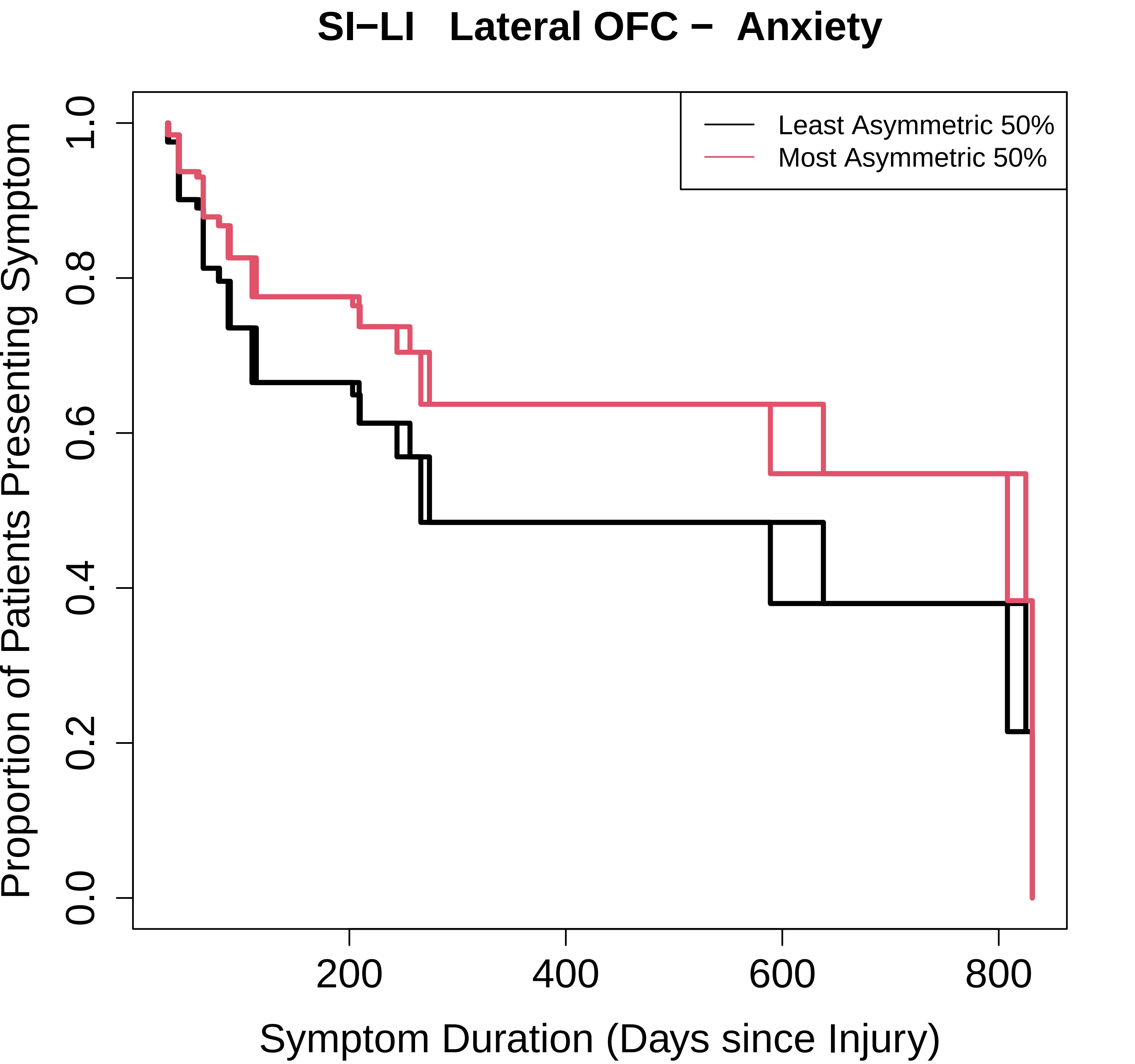

SI−LI Lateral OFC − Anxiety
Least Asymmetric 50%
Most Asymmetric 50%
Proportion of Patients Presenting Symptom
0.0
0.2
0.4
0.6
0.8
1.0
200
400
600
800
Symptom Duration (Days since Injury)

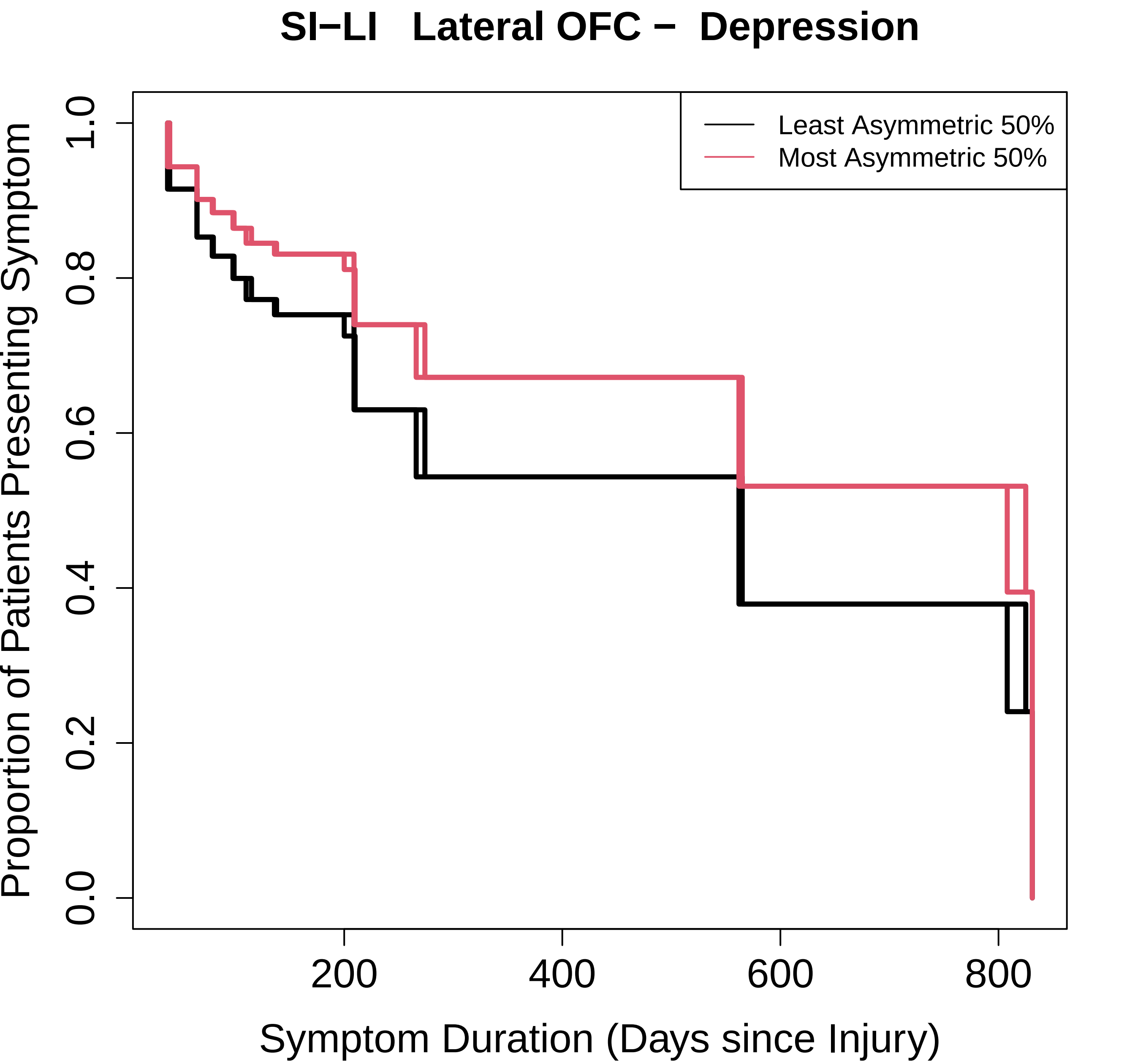
SI−LI Lateral OFC − Depression
Least Asymmetric 50%
Most Asymmetric 50%
Proportion of Patients Presenting Symptom
0.0
0.2
0.4
0.6
0.8
1.0
200
400
600
800
Symptom Duration (Days since Injury)

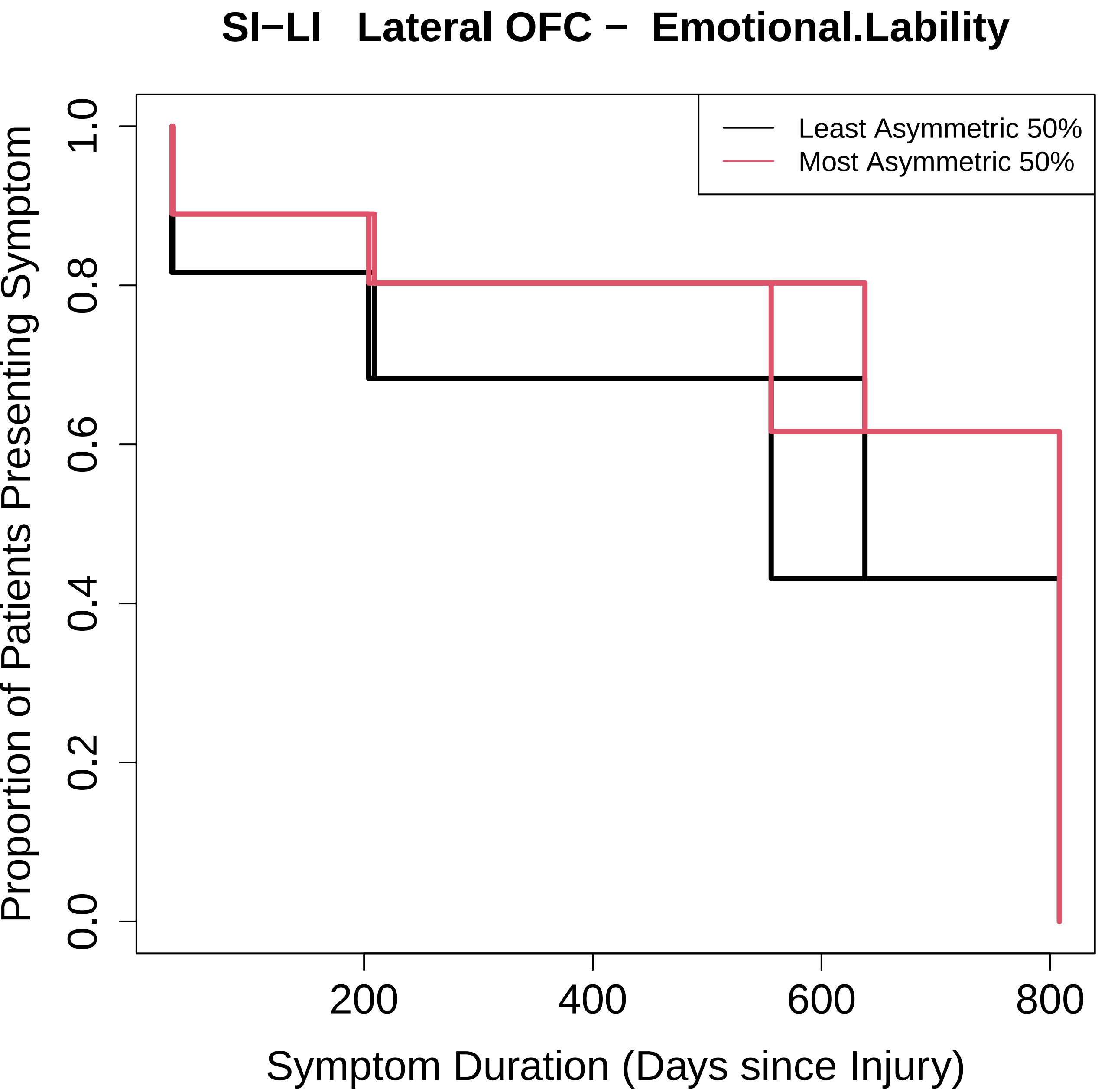
SI−LI Lateral OFC − Emotional.Lability
Least Asymmetric 50%
Most Asymmetric 50%
Proportion of Patients Presenting Symptom
0.0
0.2
0.4
0.6
0.8
1.0
200
400
600
800
Symptom Duration (Days since Injury)

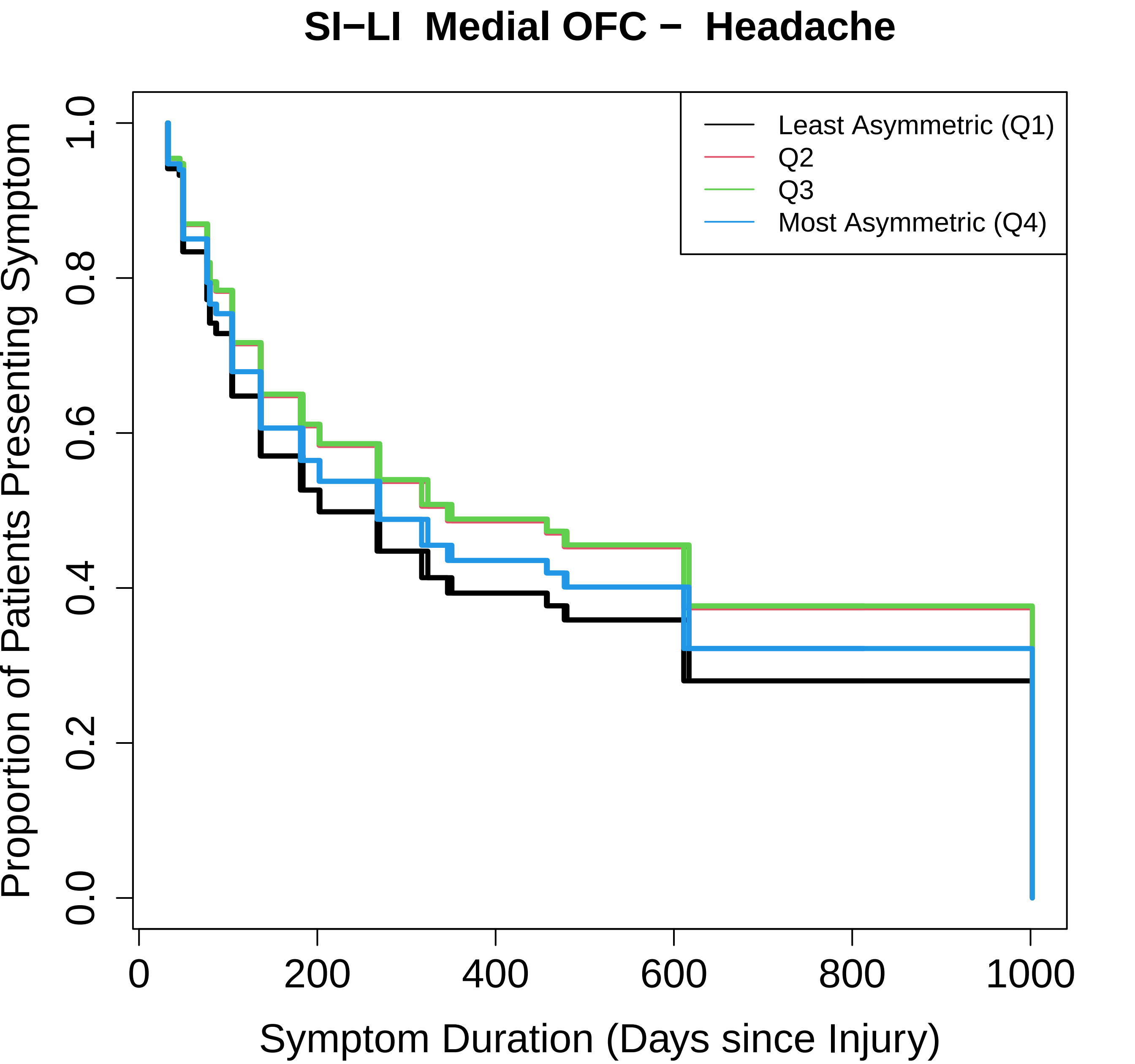

SI−LI Medial OFC − Headache
Least Asymmetric (Q1)
Q2
Q3
Most Asymmetric (Q4)
Proportion of Patients Presenting Symptom
0.0
0.2
0.4
0.6
0.8
1.0
0
200
400
600
800
1000
Symptom Duration (Days since Injury)

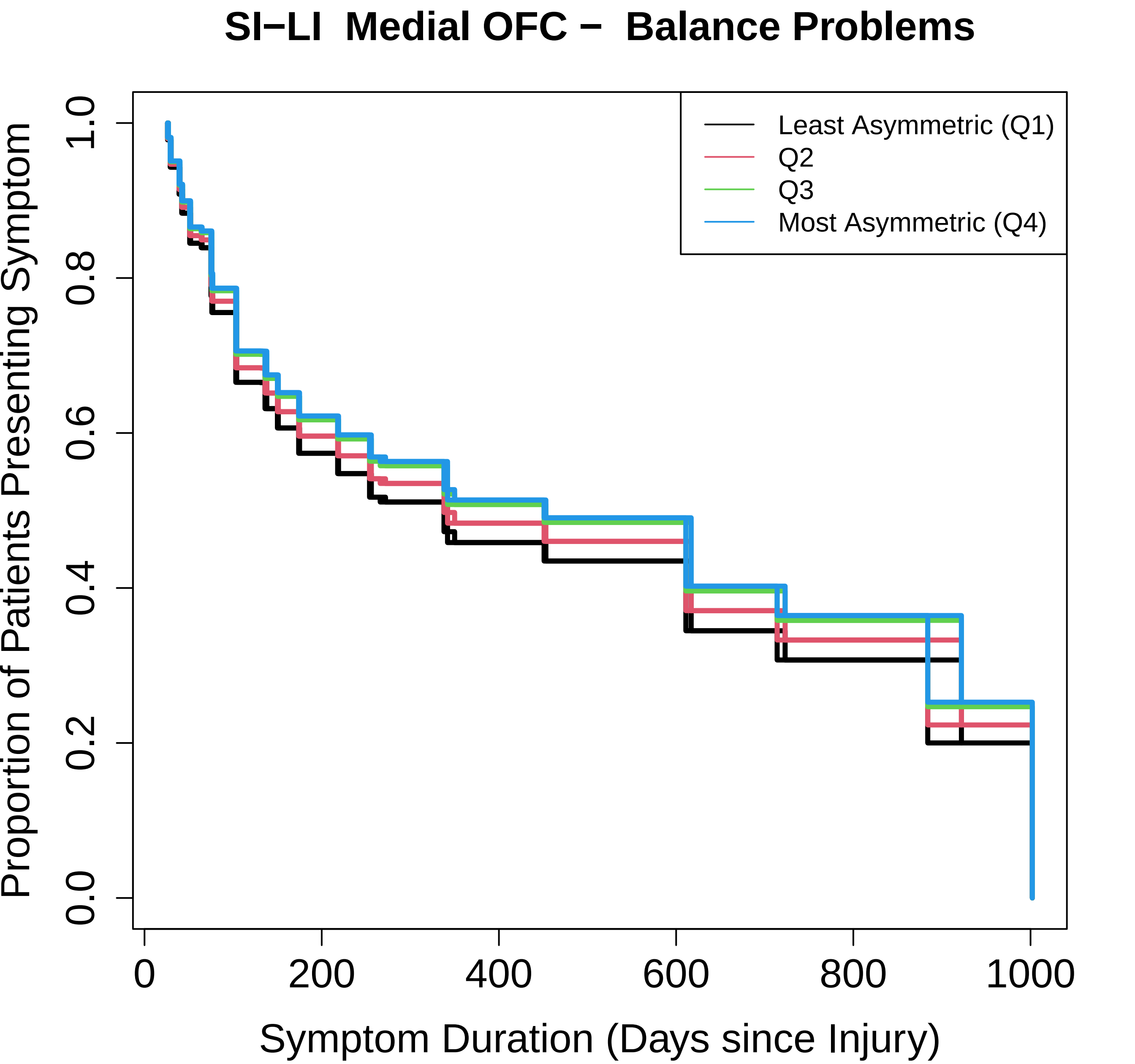
SI−LI Medial OFC − Balance Problems
Least Asymmetric (Q1)
Q2
Q3
Most Asymmetric (Q4)
Proportion of Patients Presenting Symptom
0.0
0.2
0.4
0.6
0.8
1.0
0
200
400
600
800
1000
Symptom Duration (Days since Injury)

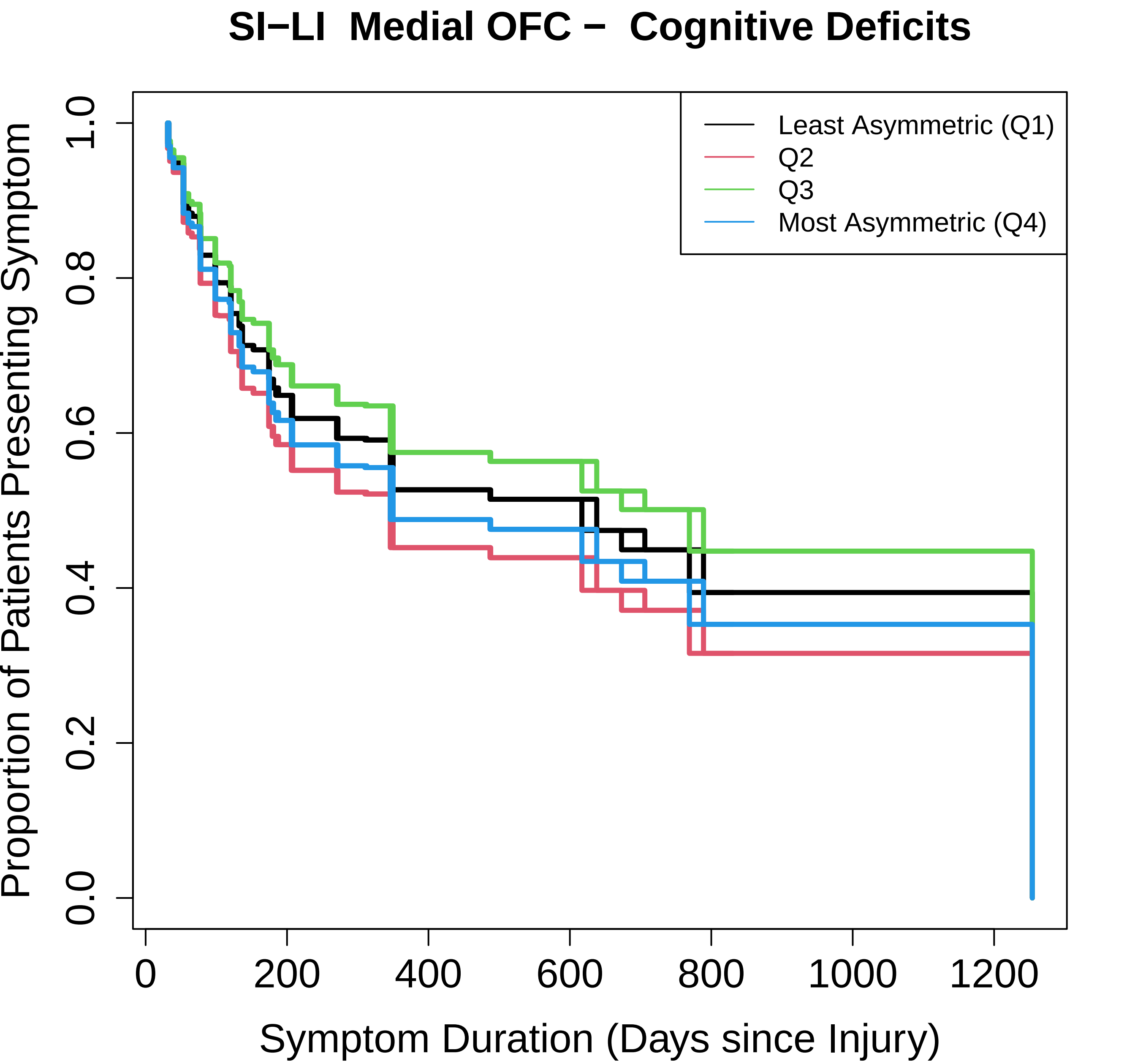
SI−LI Medial OFC − Cognitive Deficits
Least Asymmetric (Q1)
Q2
Q3
Most Asymmetric (Q4)
Proportion of Patients Presenting Symptom
0.0
0.2
0.4
0.6
0.8
1.0
0
200
400
600
800
1000
1200
Symptom Duration (Days since Injury)

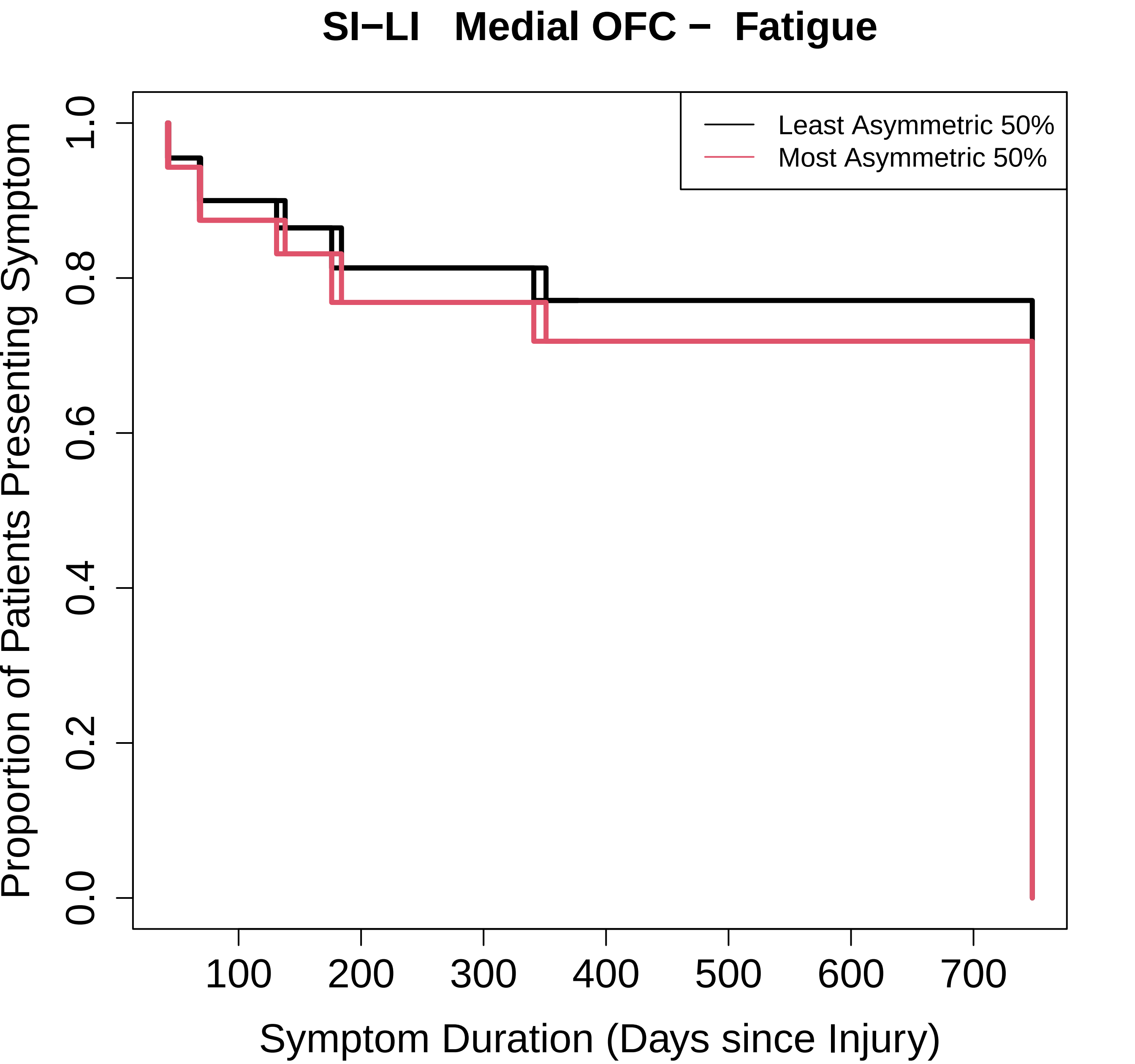
SI−LI Medial OFC − Fatigue
Least Asymmetric 50%
Most Asymmetric 50%
Proportion of Patients Presenting Symptom
0.0
0.2
0.4
0.6
0.8
1.0
100
200
300
400
500
600
700
Symptom Duration (Days since Injury)

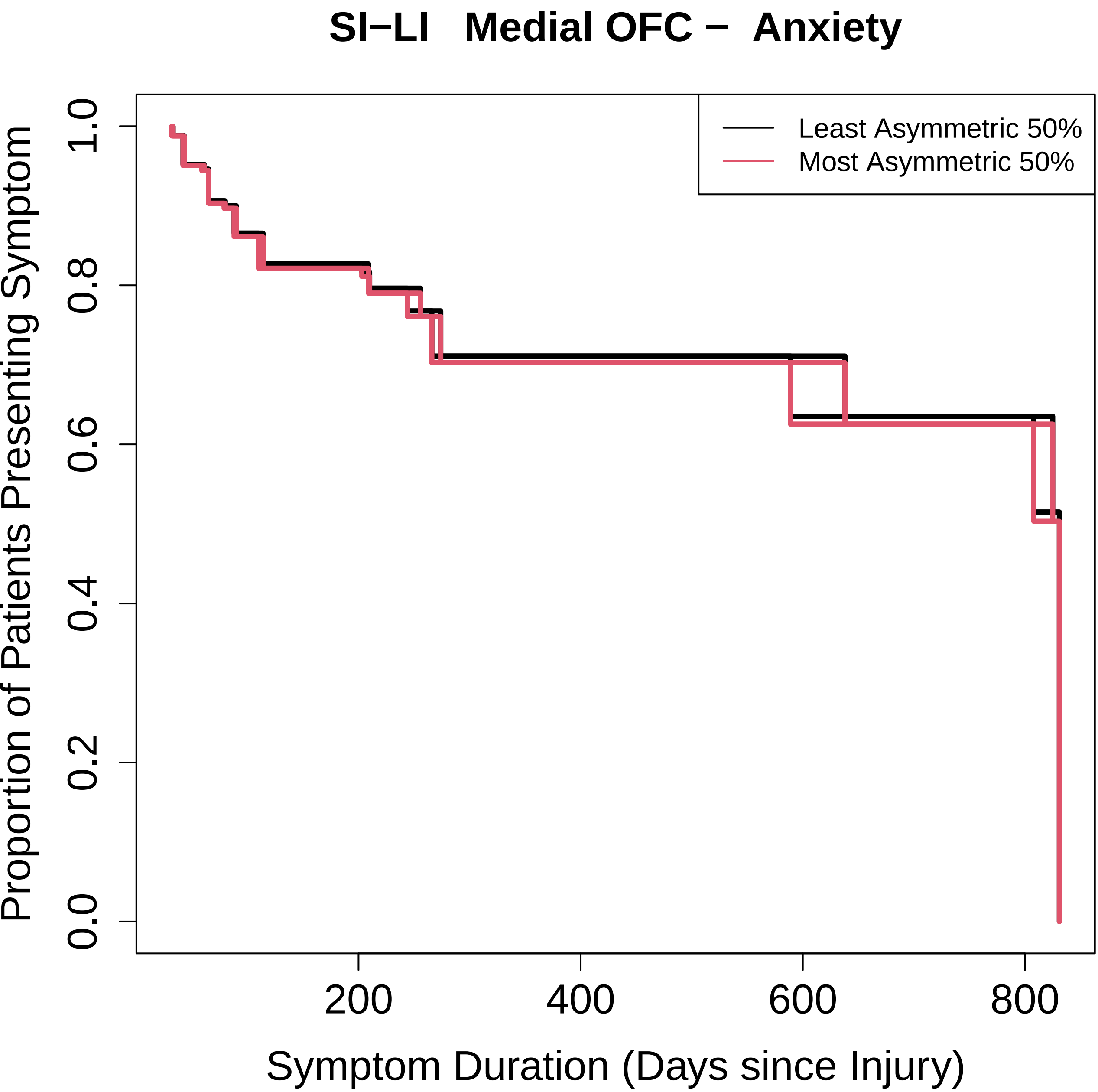

SI−LI Medial OFC − Anxiety
Least Asymmetric 50%
Most Asymmetric 50%
Proportion of Patients Presenting Symptom
0.0
0.2
0.4
0.6
0.8
1.0
200
400
600
800
Symptom Duration (Days since Injury)

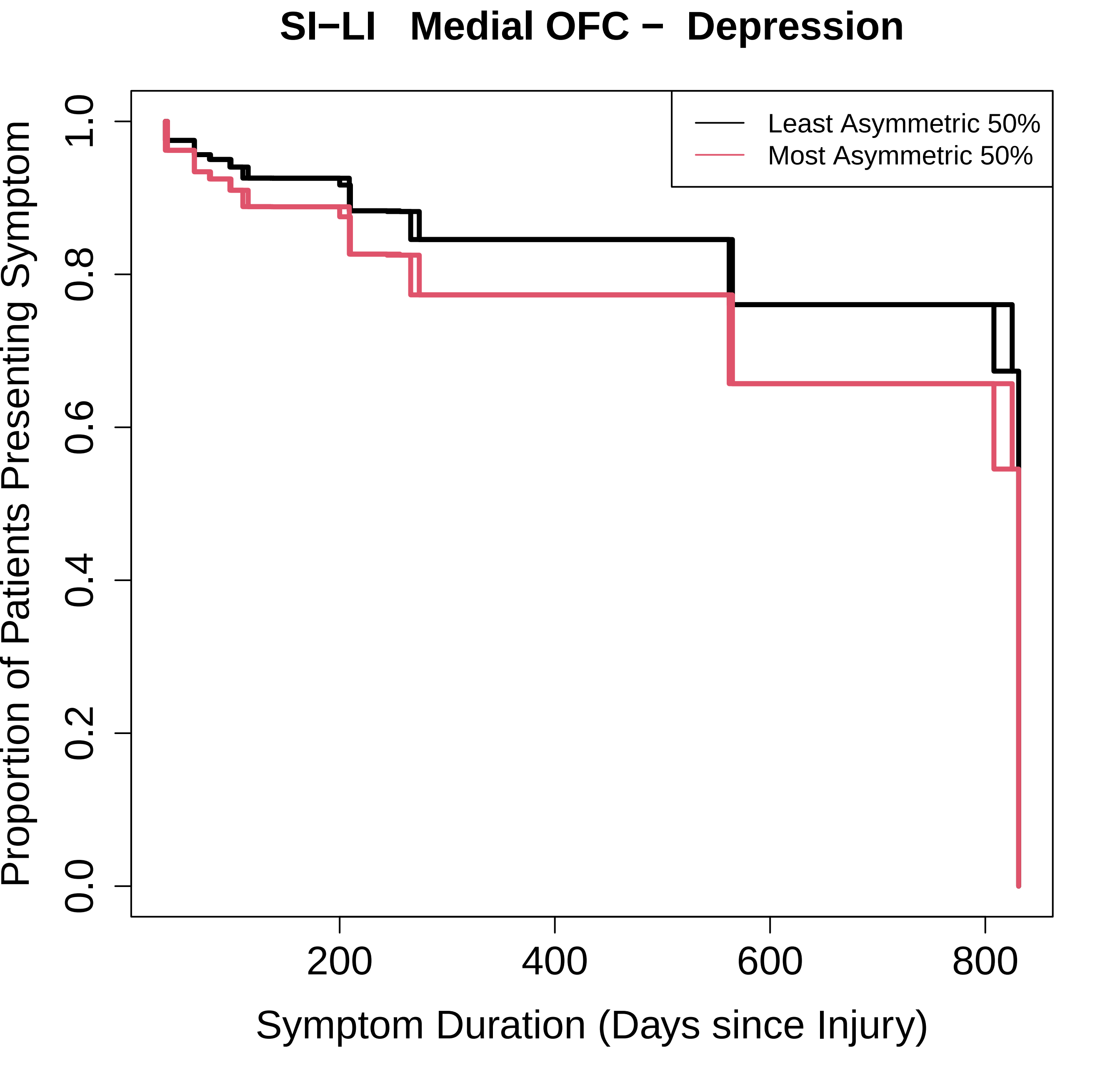

SI−LI Medial OFC − Depression
Least Asymmetric 50%
Most Asymmetric 50%
Proportion of Patients Presenting Symptom
0.0
0.2
0.4
0.6
0.8
1.0
200
400
600
800
Symptom Duration (Days since Injury)

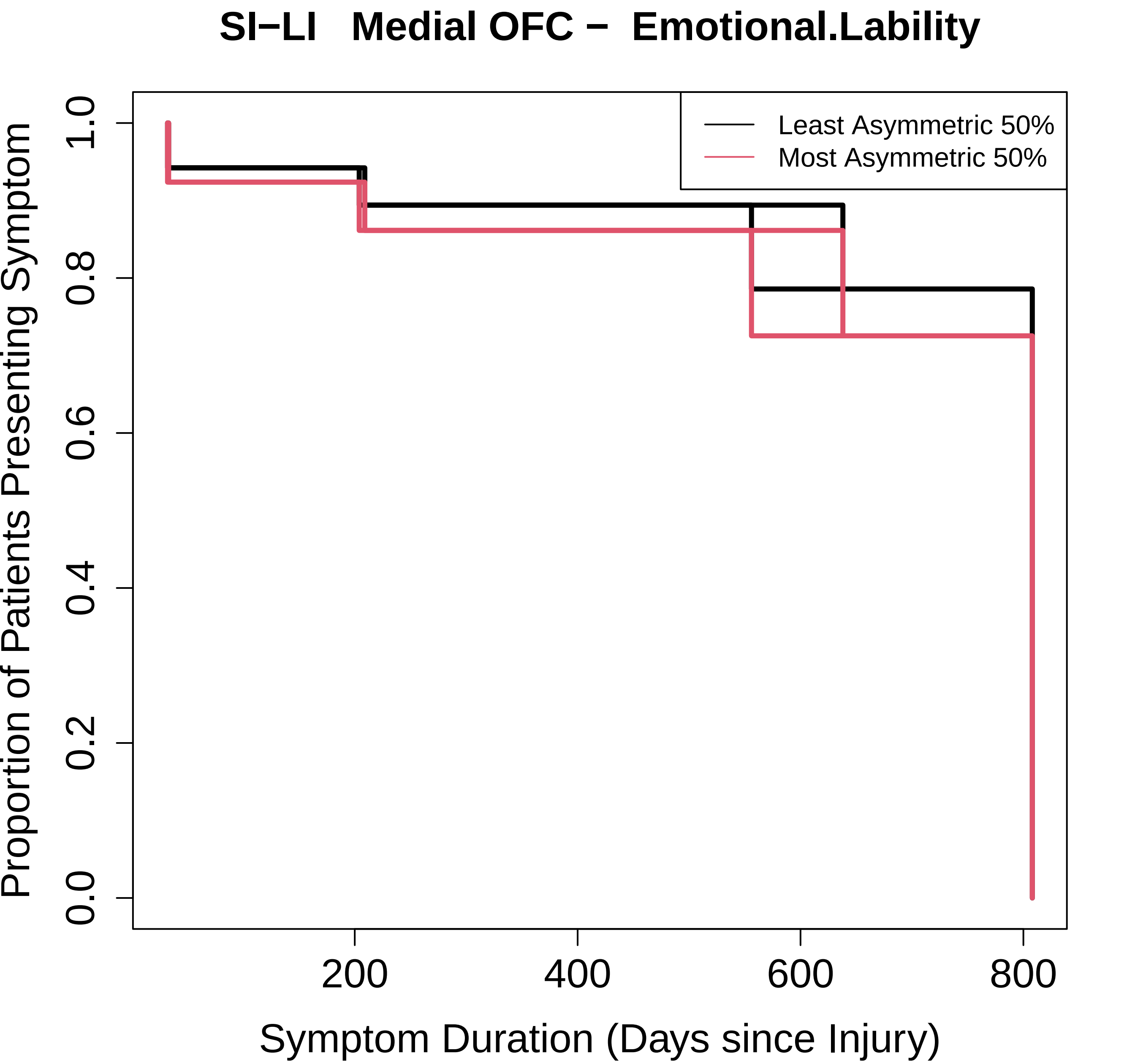
SI−LI Medial OFC − Emotional.Lability
Least Asymmetric 50%
Most Asymmetric 50%
Proportion of Patients Presenting Symptom
0.0
0.2
0.4
0.6
0.8
1.0
200
400
600
800
Symptom Duration (Days since Injury)

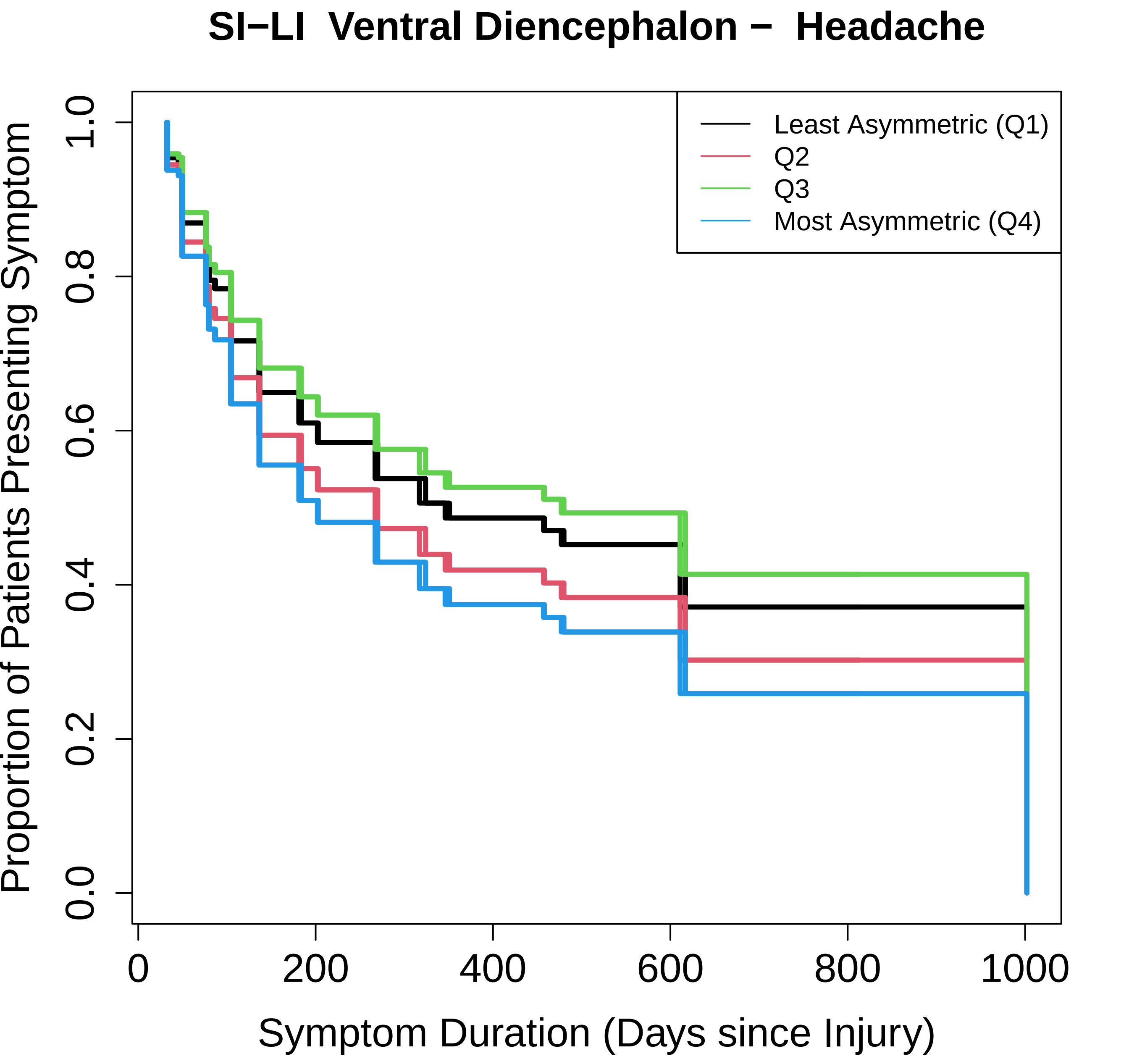
SI−LI Ventral Diencephalon − Headache
Least Asymmetric (Q1)
Q2
Q3
Most Asymmetric (Q4)
Proportion of Patients Presenting Symptom
0.0
0.2
0.4
0.6
0.8
1.0
0
200
400
600
800
1000
Symptom Duration (Days since Injury)

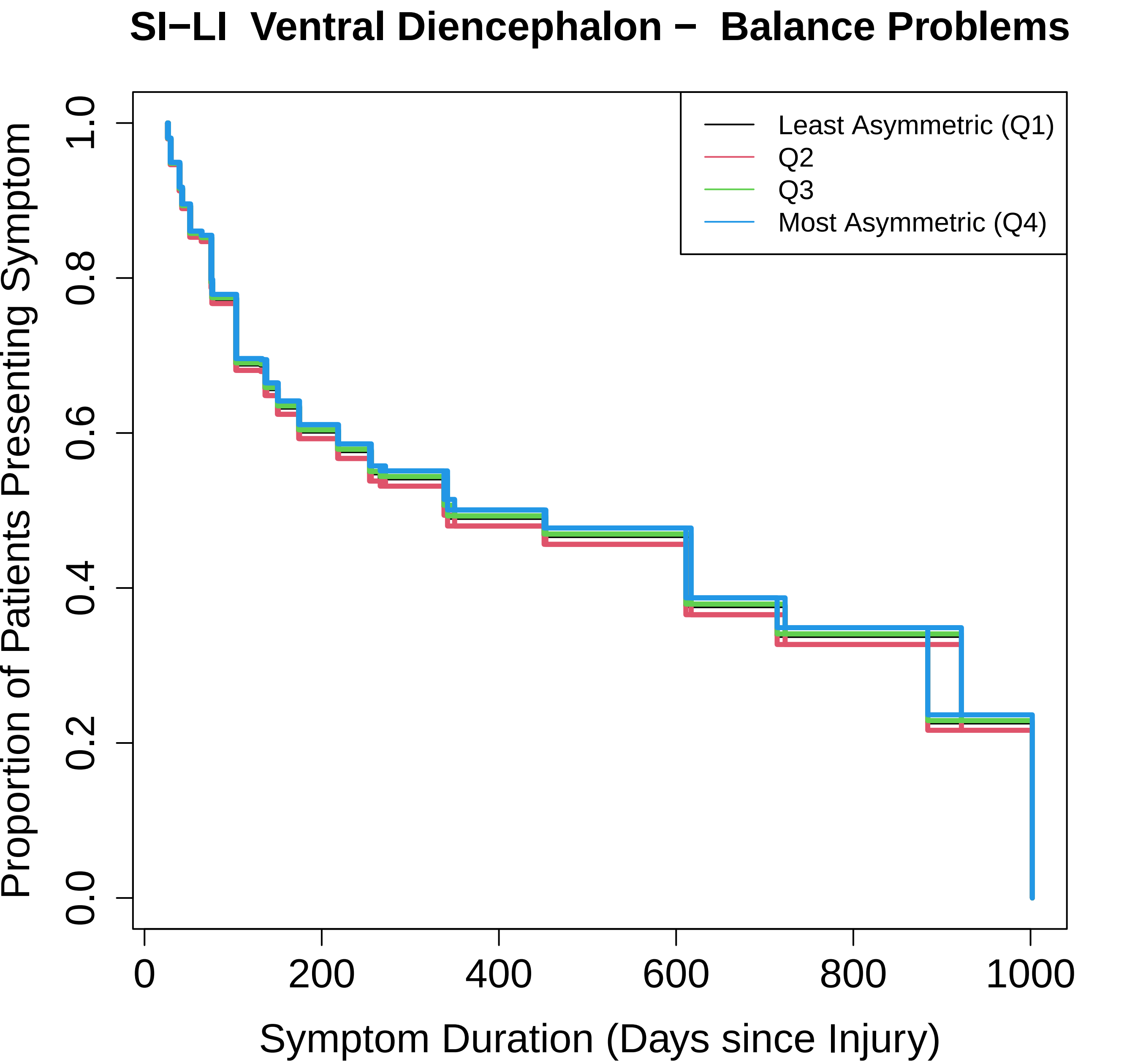
SI−LI Ventral Diencephalon − Balance Problems
Least Asymmetric (Q1)
Q2
Q3
Most Asymmetric (Q4)
Proportion of Patients Presenting Symptom
0.0
0.2
0.4
0.6
0.8
1.0
0
200
400
600
800
1000
Symptom Duration (Days since Injury)

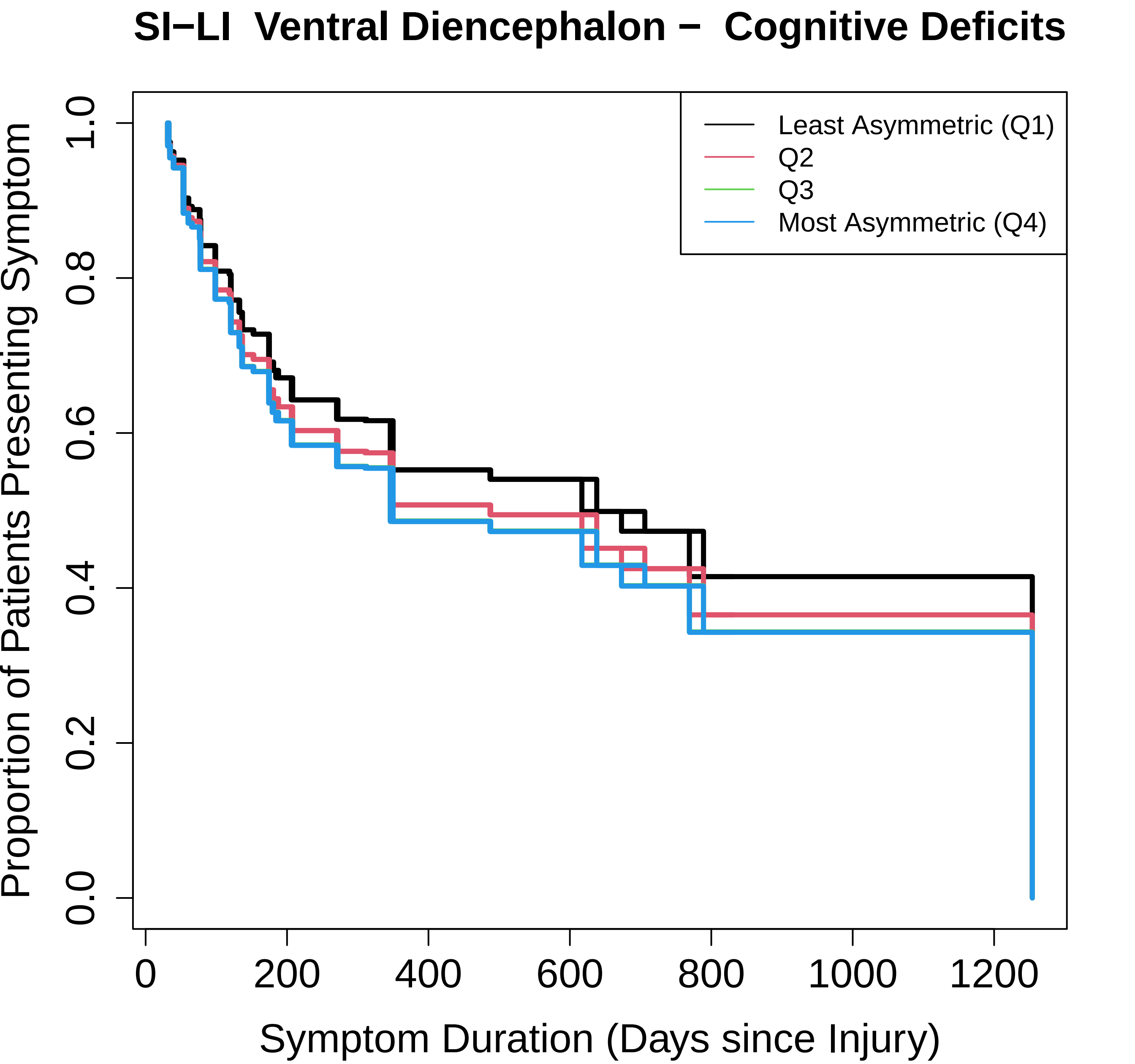

SI−LI Ventral Diencephalon − Cognitive Deficits
Least Asymmetric (Q1)
Q2
Q3
Most Asymmetric (Q4)
Proportion of Patients Presenting Symptom
0.0
0.2
0.4
0.6
0.8
1.0
0
200
400
600
800
1000
1200
Symptom Duration (Days since Injury)

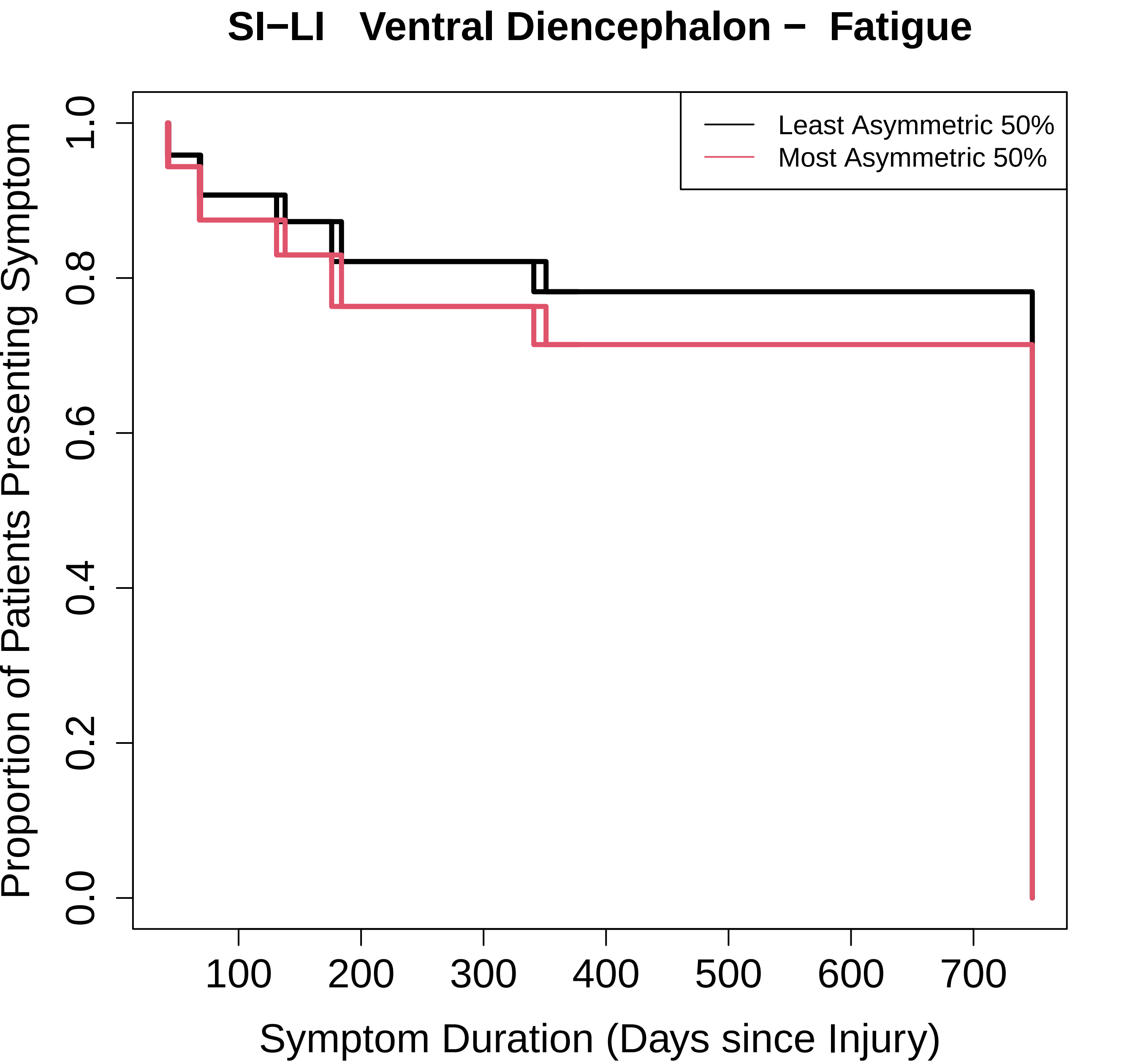
SI−LI Ventral Diencephalon − Fatigue
Least Asymmetric 50%
Most Asymmetric 50%
Proportion of Patients Presenting Symptom
0.0
0.2
0.4
0.6
0.8
1.0
100
200
300
400
500
600
700
Symptom Duration (Days since Injury)

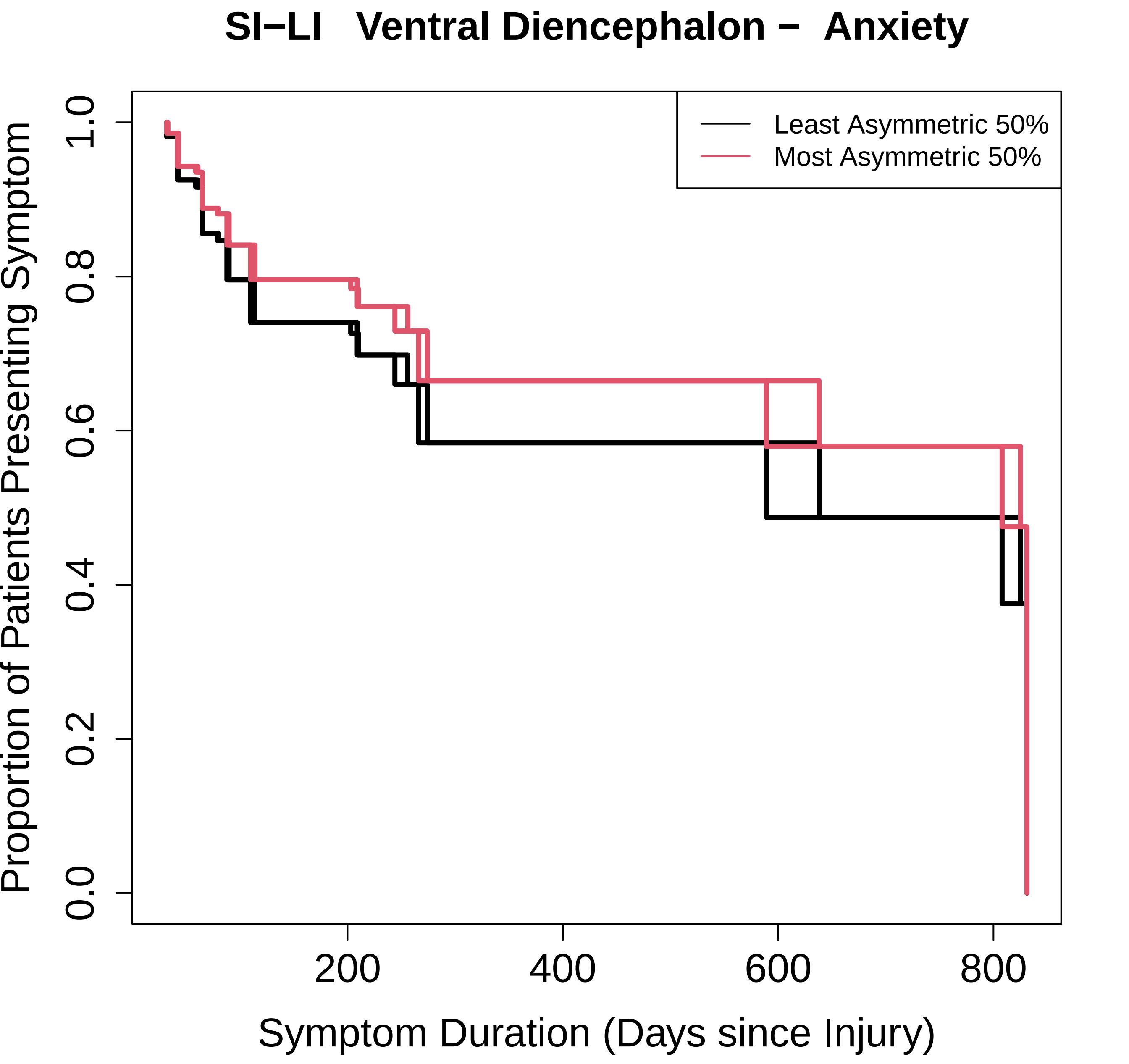
SI−LI Ventral Diencephalon − Anxiety
Least Asymmetric 50%
Most Asymmetric 50%
Proportion of Patients Presenting Symptom
0.0
0.2
0.4
0.6
0.8
1.0
200
400
600
800
Symptom Duration (Days since Injury)

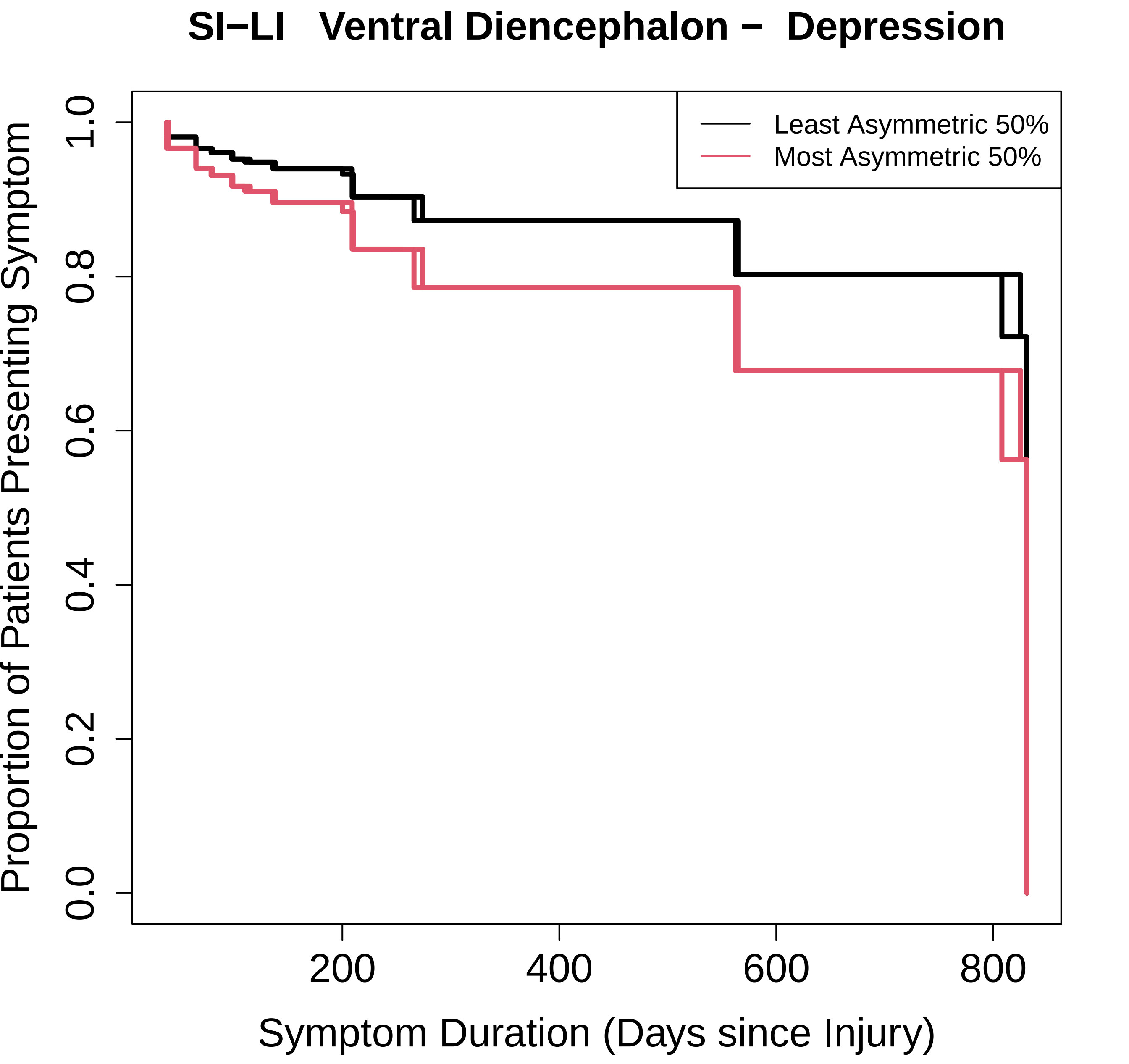
SI−LI Ventral Diencephalon − Depression
Least Asymmetric 50%
Most Asymmetric 50%
Proportion of Patients Presenting Symptom
0.0
0.2
0.4
0.6
0.8
1.0
200
400
600
800
Symptom Duration (Days since Injury)

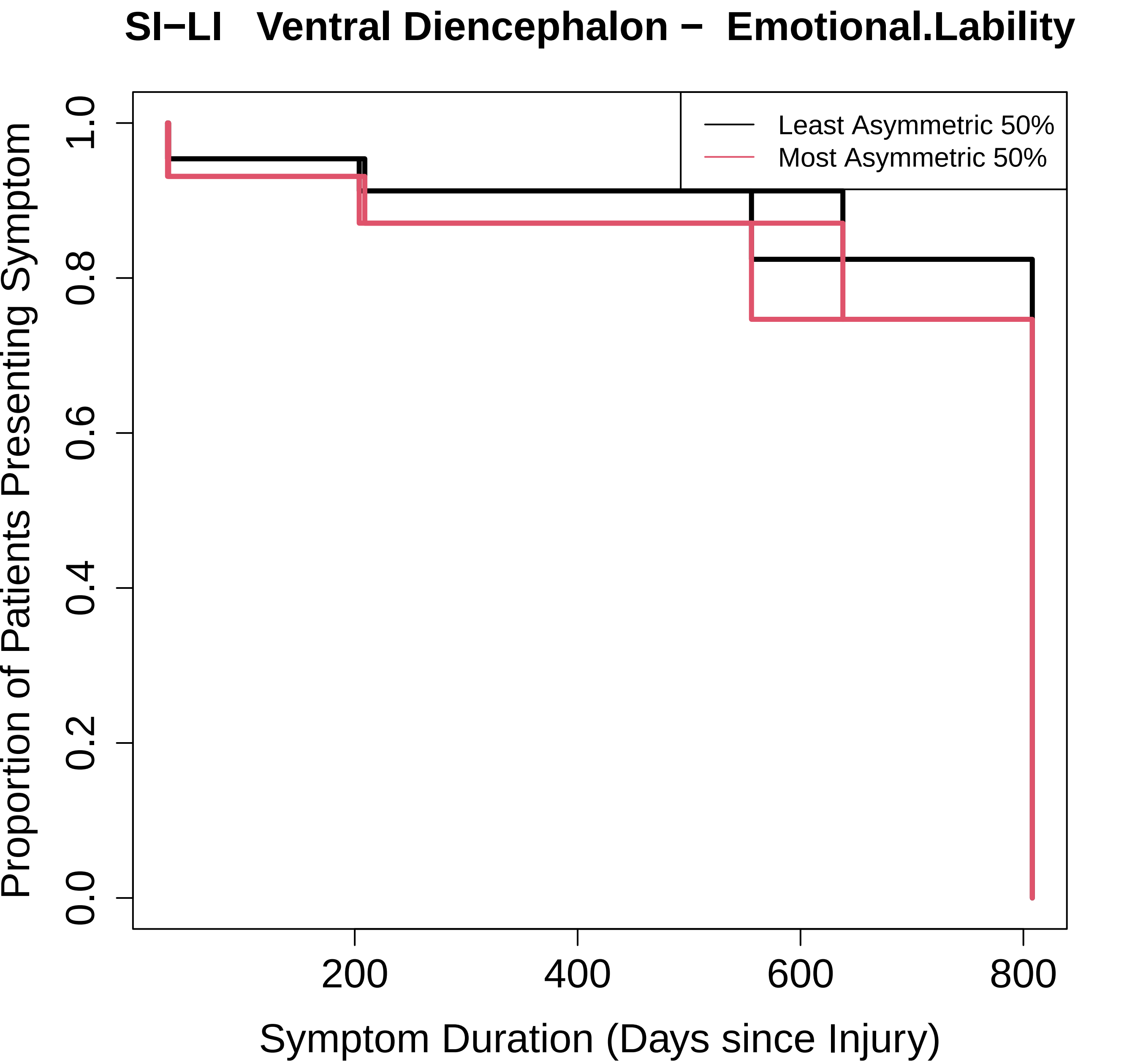

SI−LI Ventral Diencephalon − Emotional.Lability
Least Asymmetric 50%
Most Asymmetric 50%
Proportion of Patients Presenting Symptom
0.0
0.2
0.4
0.6
0.8
1.0
200
400
600
800
Symptom Duration (Days since Injury)

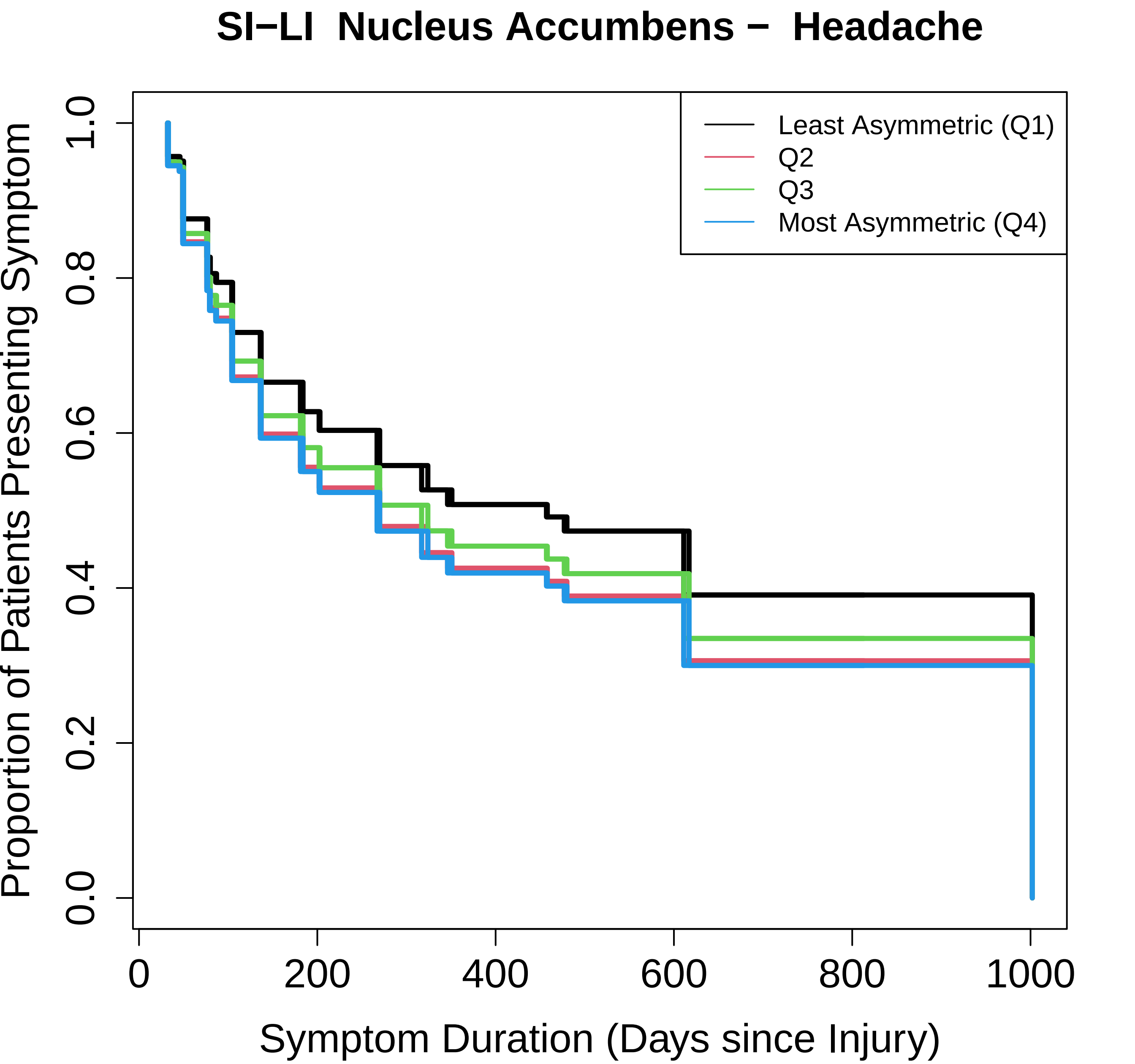
SI−LI Nucleus Accumbens − Headache
Least Asymmetric (Q1)
Q2
Q3
Most Asymmetric (Q4)
Proportion of Patients Presenting Symptom
0.0
0.2
0.4
0.6
0.8
1.0
0
200
400
600
800
1000
Symptom Duration (Days since Injury)

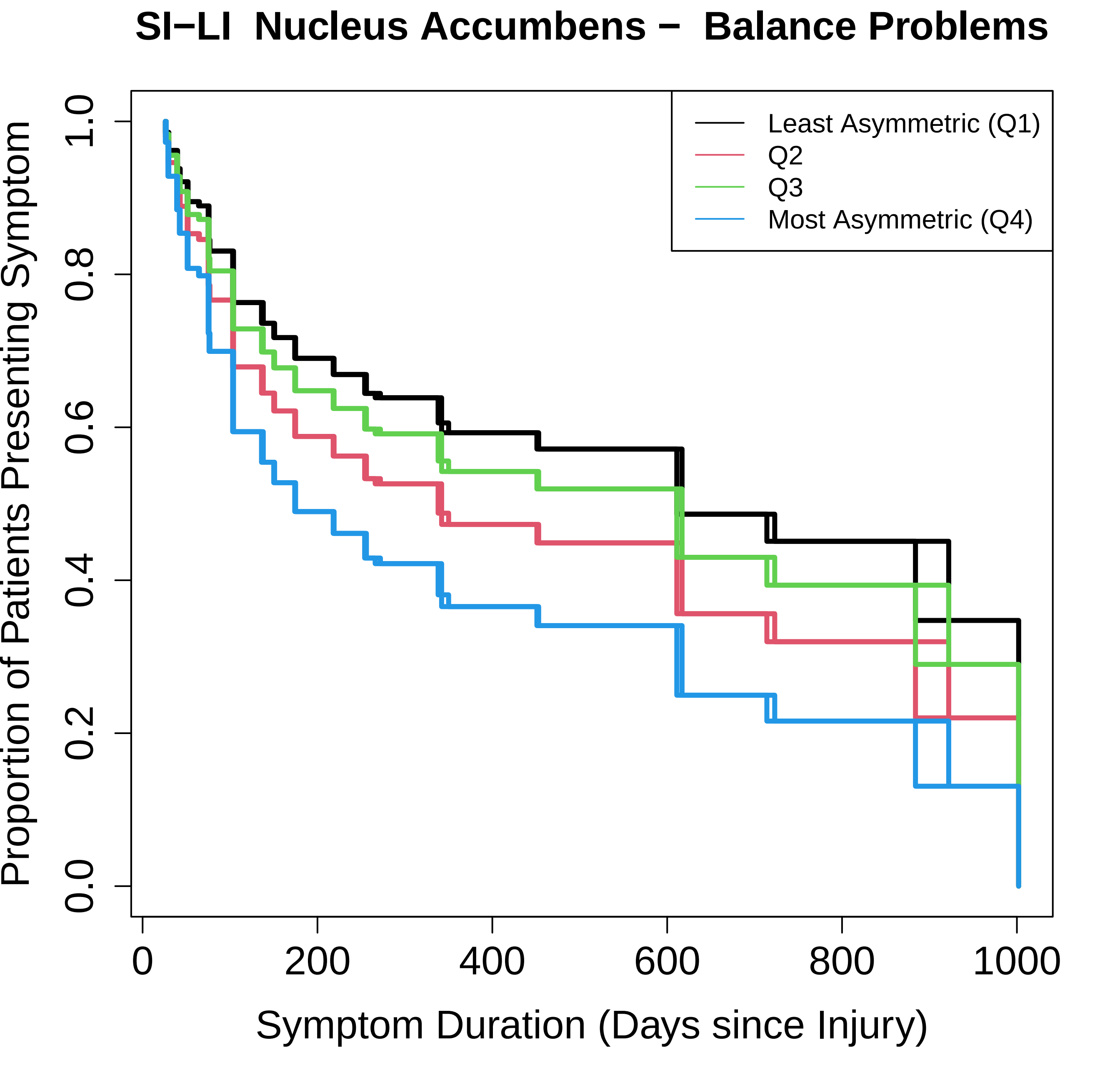

SI−LI Nucleus Accumbens − Balance Problems
Least Asymmetric (Q1)
Q2
Q3
Most Asymmetric (Q4)
Proportion of Patients Presenting Symptom
0.0
0.2
0.4
0.6
0.8
1.0
0
200
400
600
800
1000
Symptom Duration (Days since Injury)

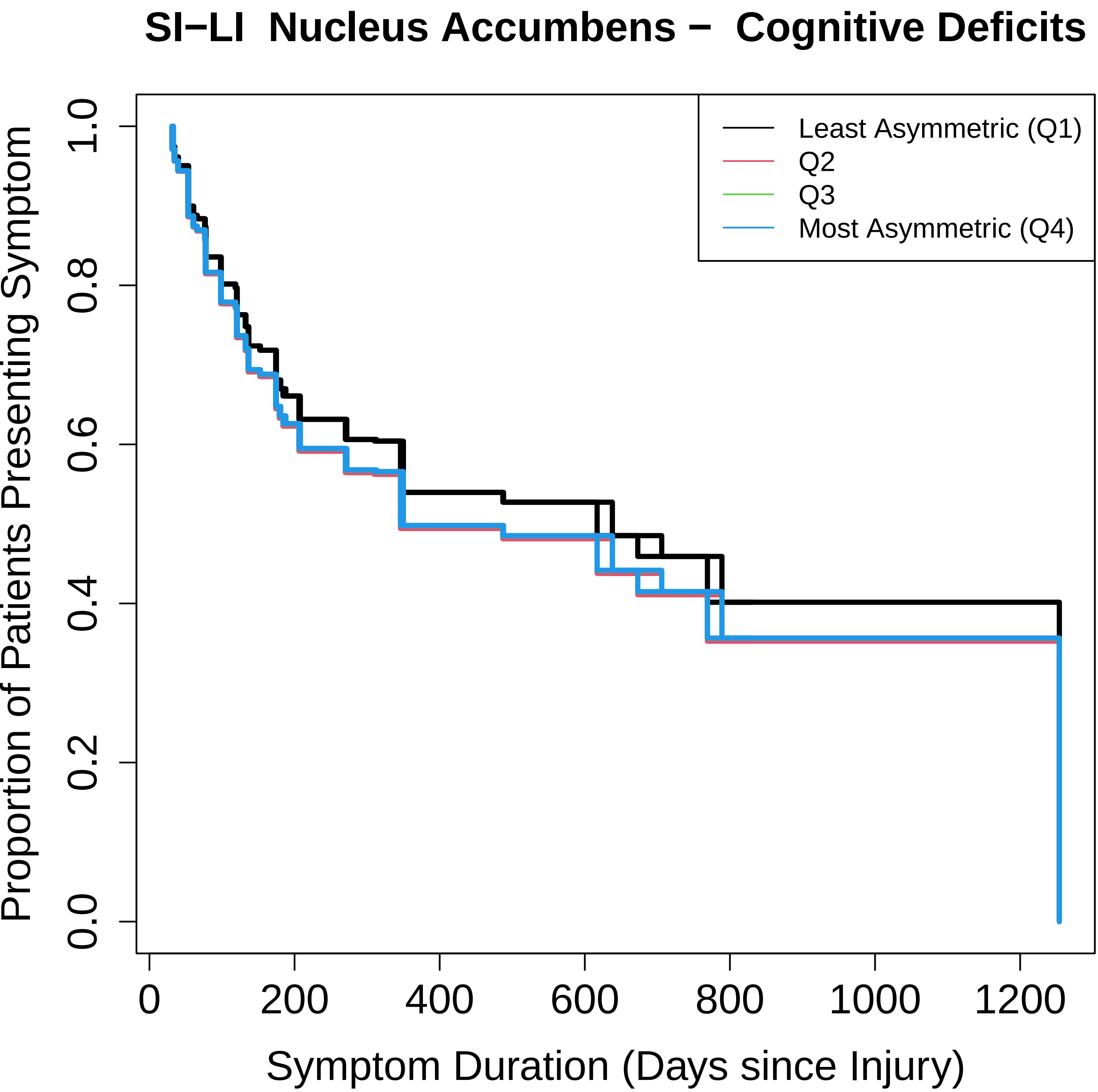
SI−LI Nucleus Accumbens − Cognitive Deficits
Least Asymmetric (Q1)
Q2
Q3
Most Asymmetric (Q4)
Proportion of Patients Presenting Symptom
0.0
0.2
0.4
0.6
0.8
1.0
0
200
400
600
800
1000
1200
Symptom Duration (Days since Injury)

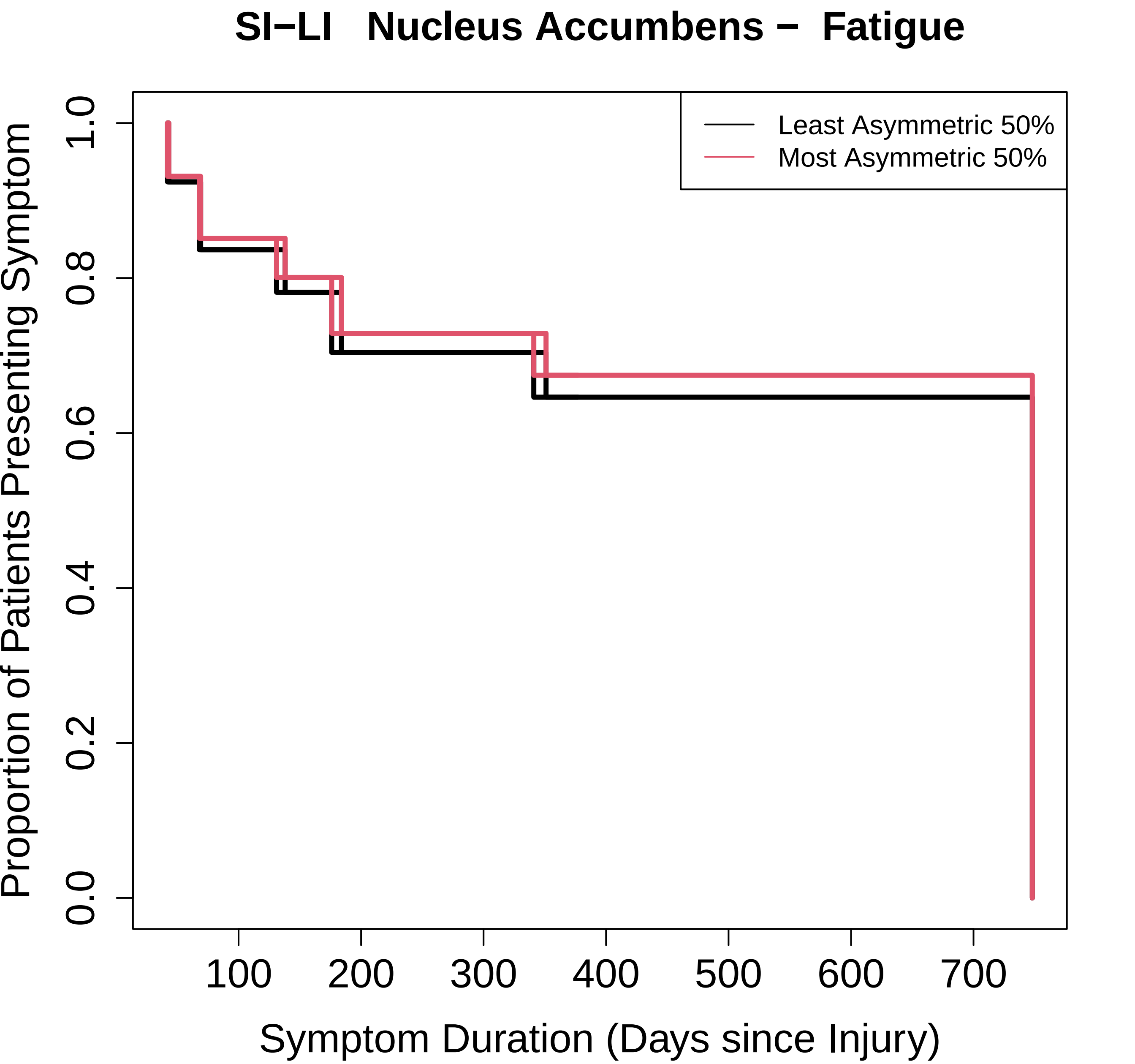
SI−LI Nucleus Accumbens − Fatigue
Least Asymmetric 50%
Most Asymmetric 50%
Proportion of Patients Presenting Symptom
0.0
0.2
0.4
0.6
0.8
1.0
100
200
300
400
500
600
700
Symptom Duration (Days since Injury)

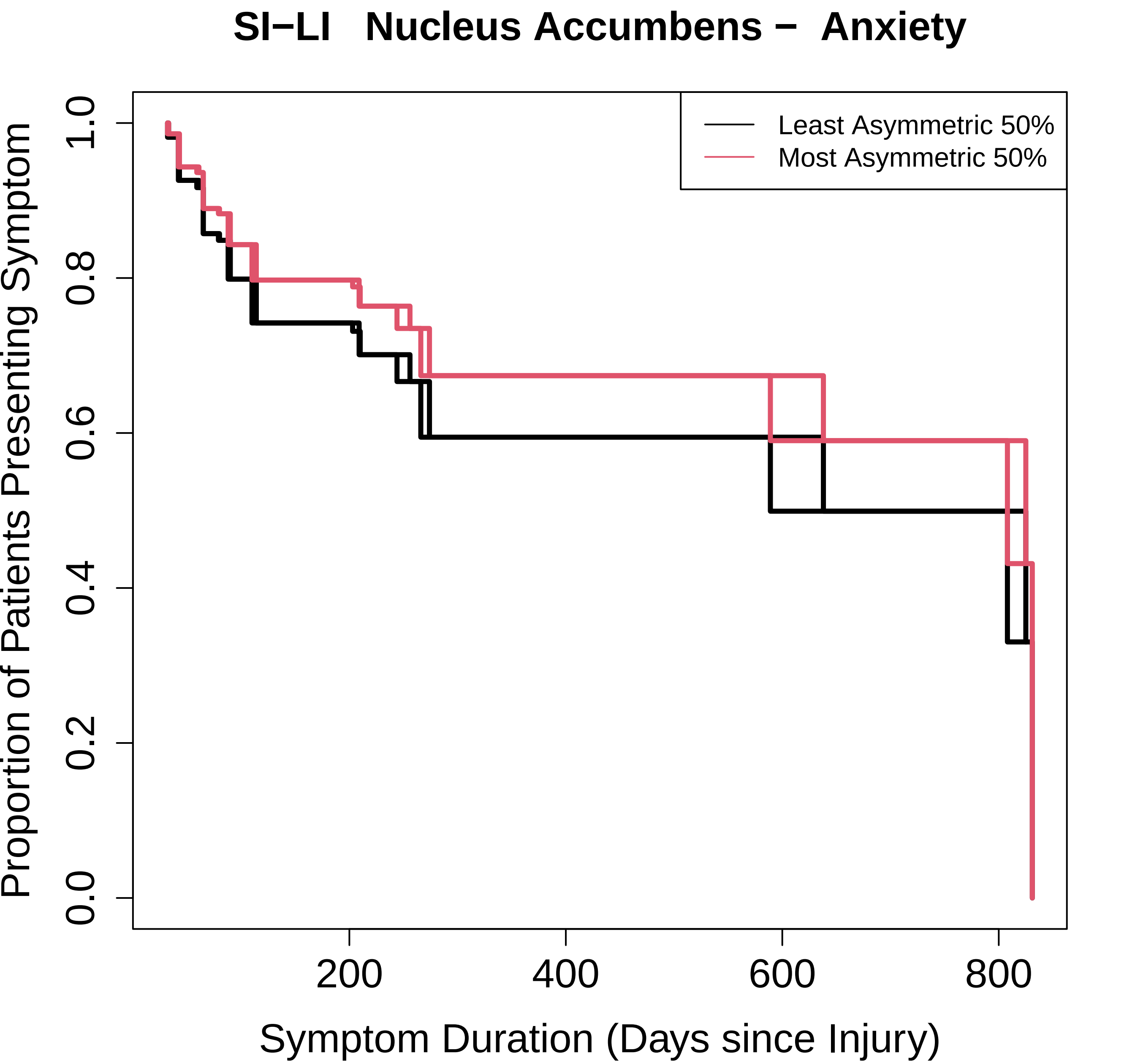

SI−LI Nucleus Accumbens − Anxiety
Least Asymmetric 50%
Most Asymmetric 50%
Proportion of Patients Presenting Symptom
0.0
0.2
0.4
0.6
0.8
1.0
200
400
600
800
Symptom Duration (Days since Injury)

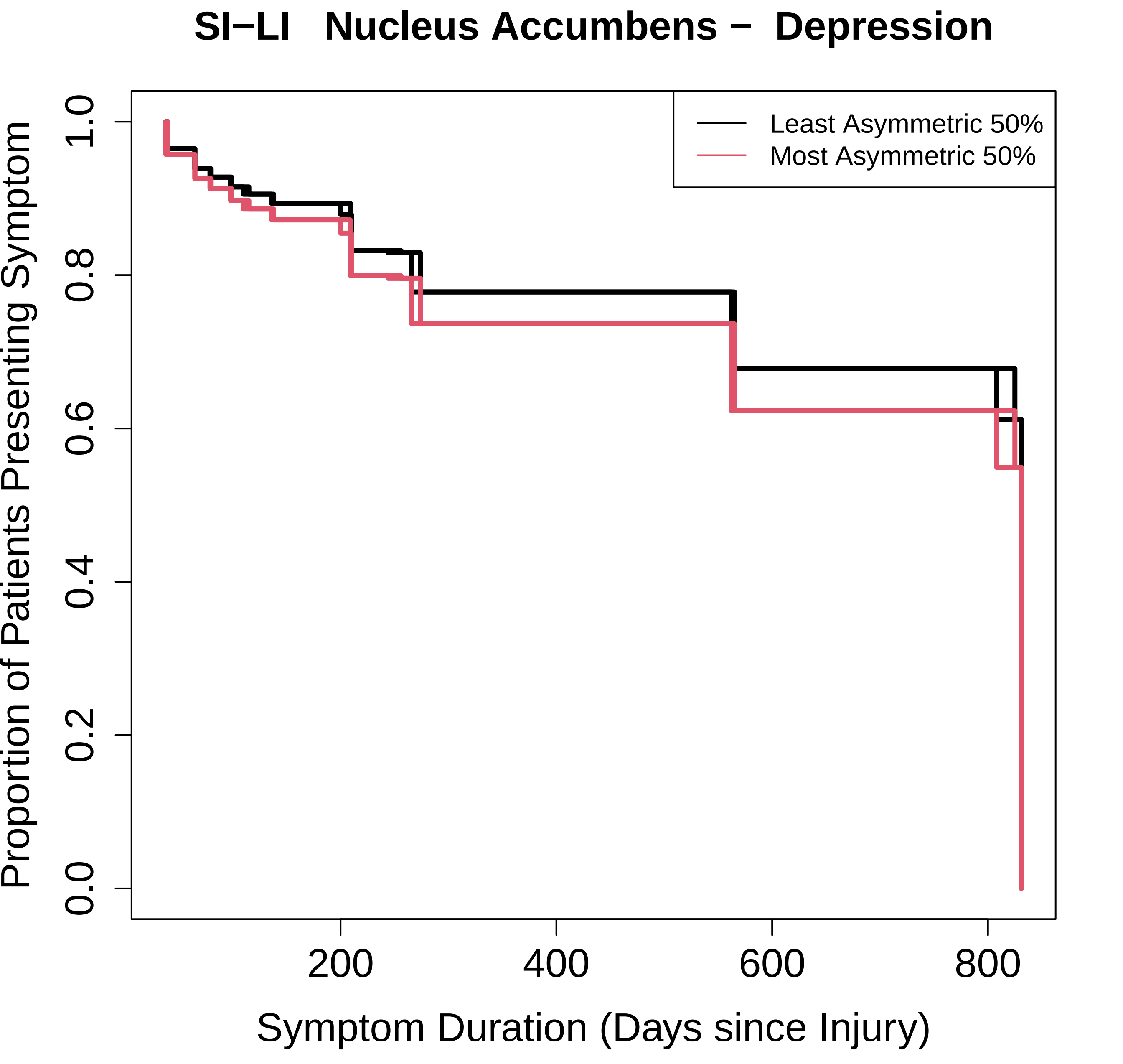
SI−LI Nucleus Accumbens − Depression
Least Asymmetric 50%
Most Asymmetric 50%
Proportion of Patients Presenting Symptom
0.0
0.2
0.4
0.6
0.8
1.0
200
400
600
800
Symptom Duration (Days since Injury)

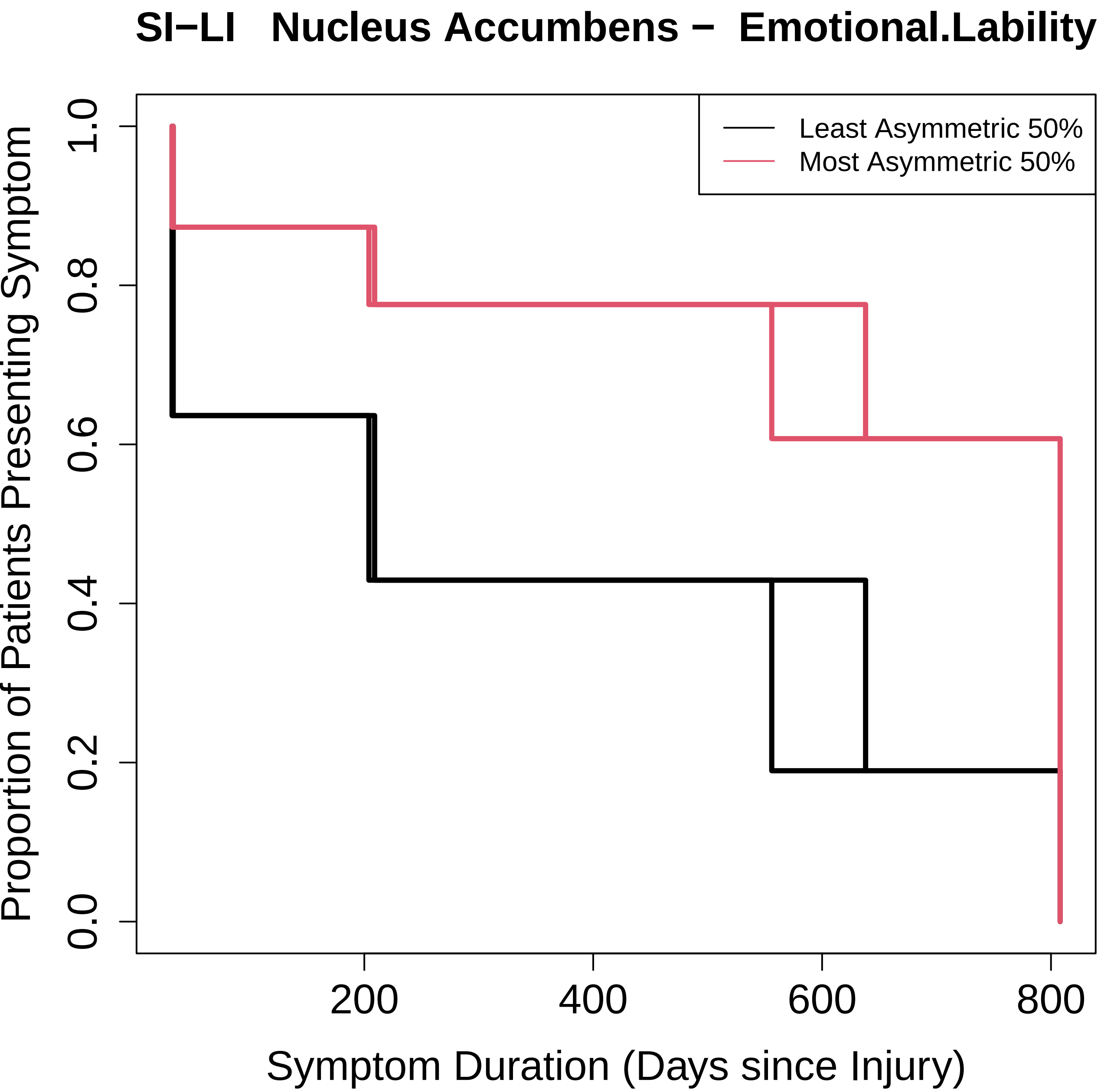
SI−LI Nucleus Accumbens − Emotional.Lability
Least Asymmetric 50%
Most Asymmetric 50%
Proportion of Patients Presenting Symptom
0.0
0.2
0.4
0.6
0.8
1.0
200
400
600
800
Symptom Duration (Days since Injury)

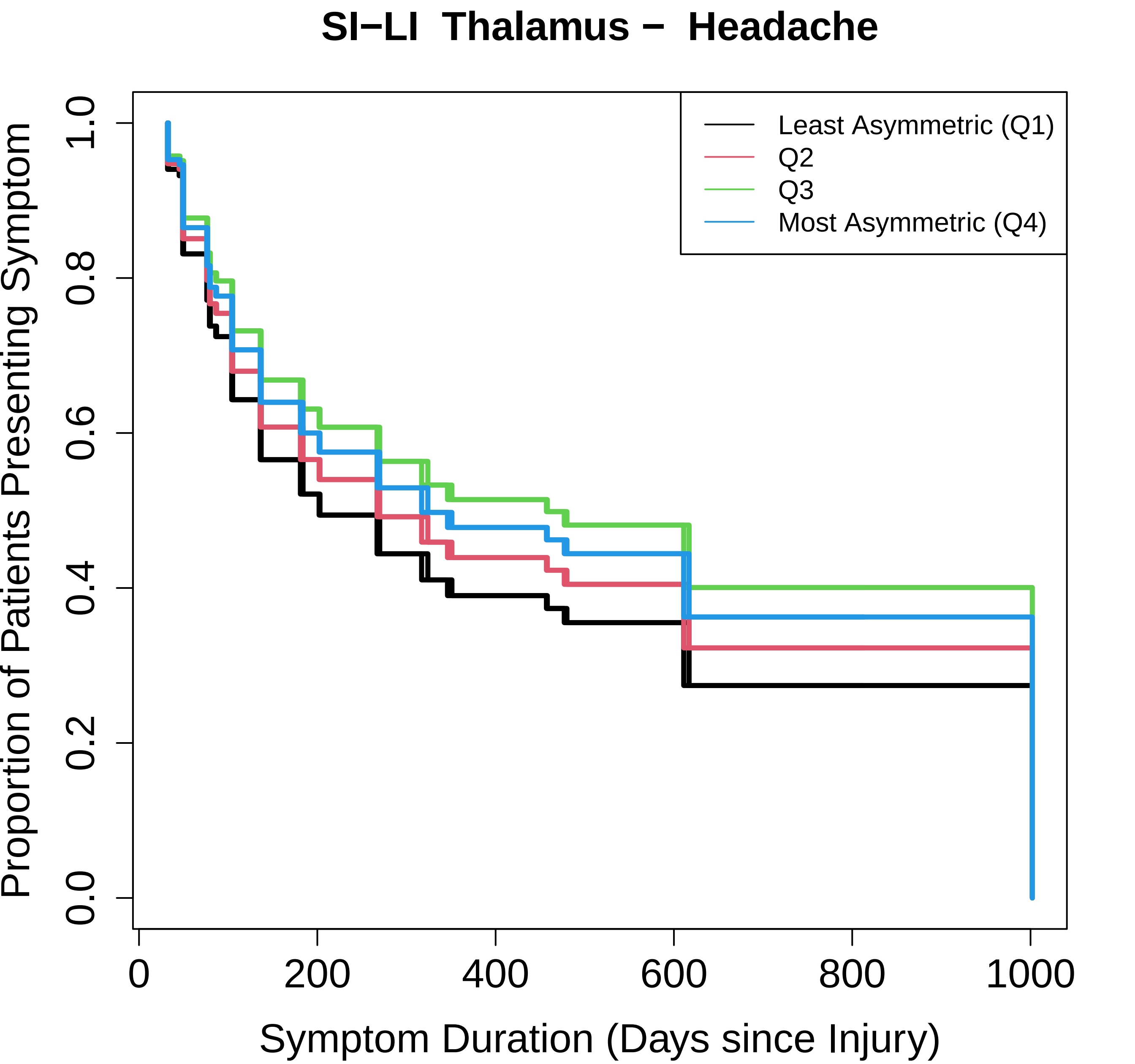

SI−LI Thalamus − Headache
Least Asymmetric (Q1)
Q2
Q3
Most Asymmetric (Q4)
Proportion of Patients Presenting Symptom
0.0
0.2
0.4
0.6
0.8
1.0
0
200
400
600
800
1000
Symptom Duration (Days since Injury)

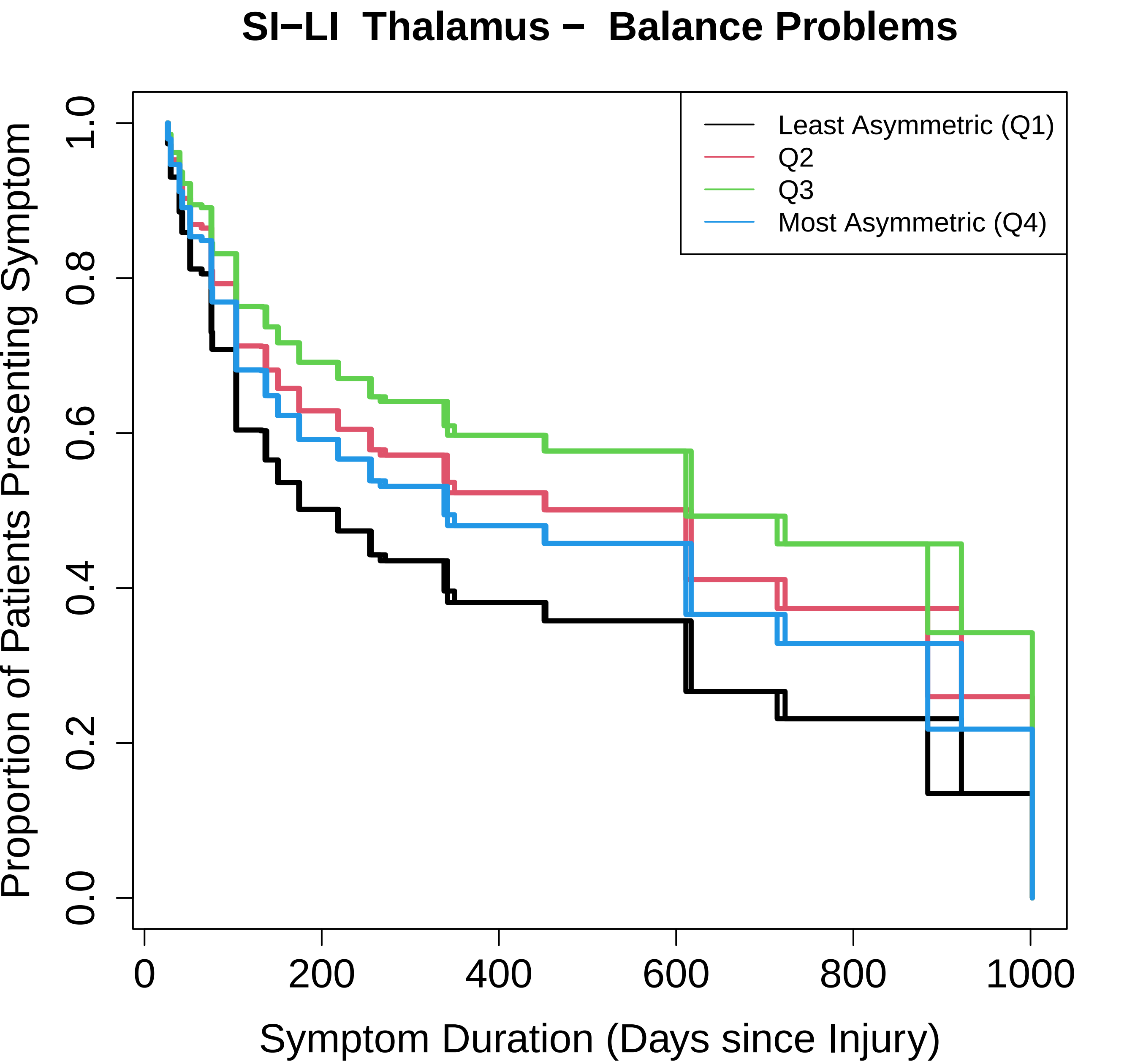
SI−LI Thalamus − Balance Problems
Least Asymmetric (Q1)
Q2
Q3
Most Asymmetric (Q4)
Proportion of Patients Presenting Symptom
0.0
0.2
0.4
0.6
0.8
1.0
0
200
400
600
800
1000
Symptom Duration (Days since Injury)

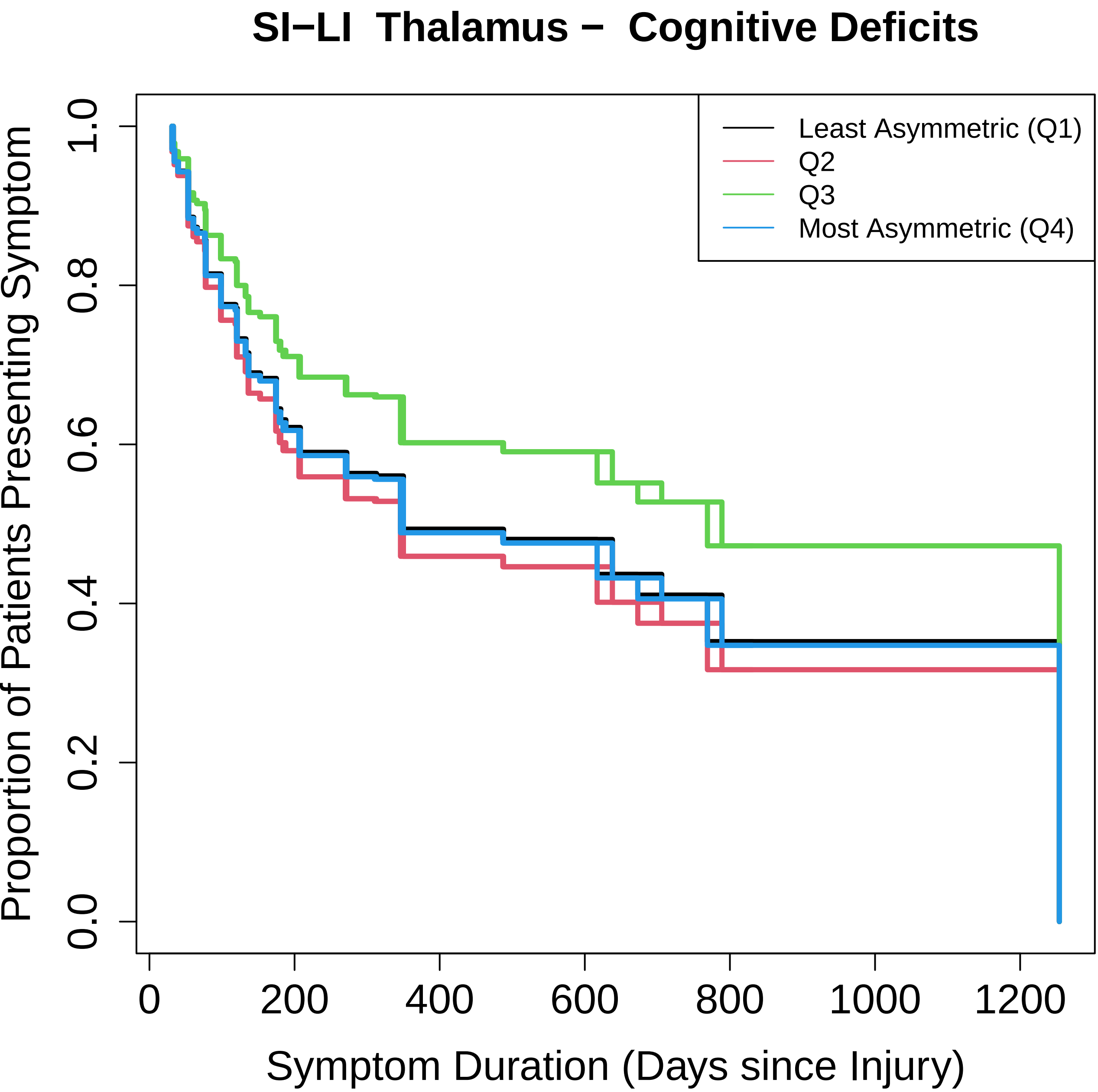
SI−LI Thalamus − Cognitive Deficits
Least Asymmetric (Q1)
Q2
Q3
Most Asymmetric (Q4)
Proportion of Patients Presenting Symptom
0.0
0.2
0.4
0.6
0.8
1.0
0
200
400
600
800
1000
1200
Symptom Duration (Days since Injury)

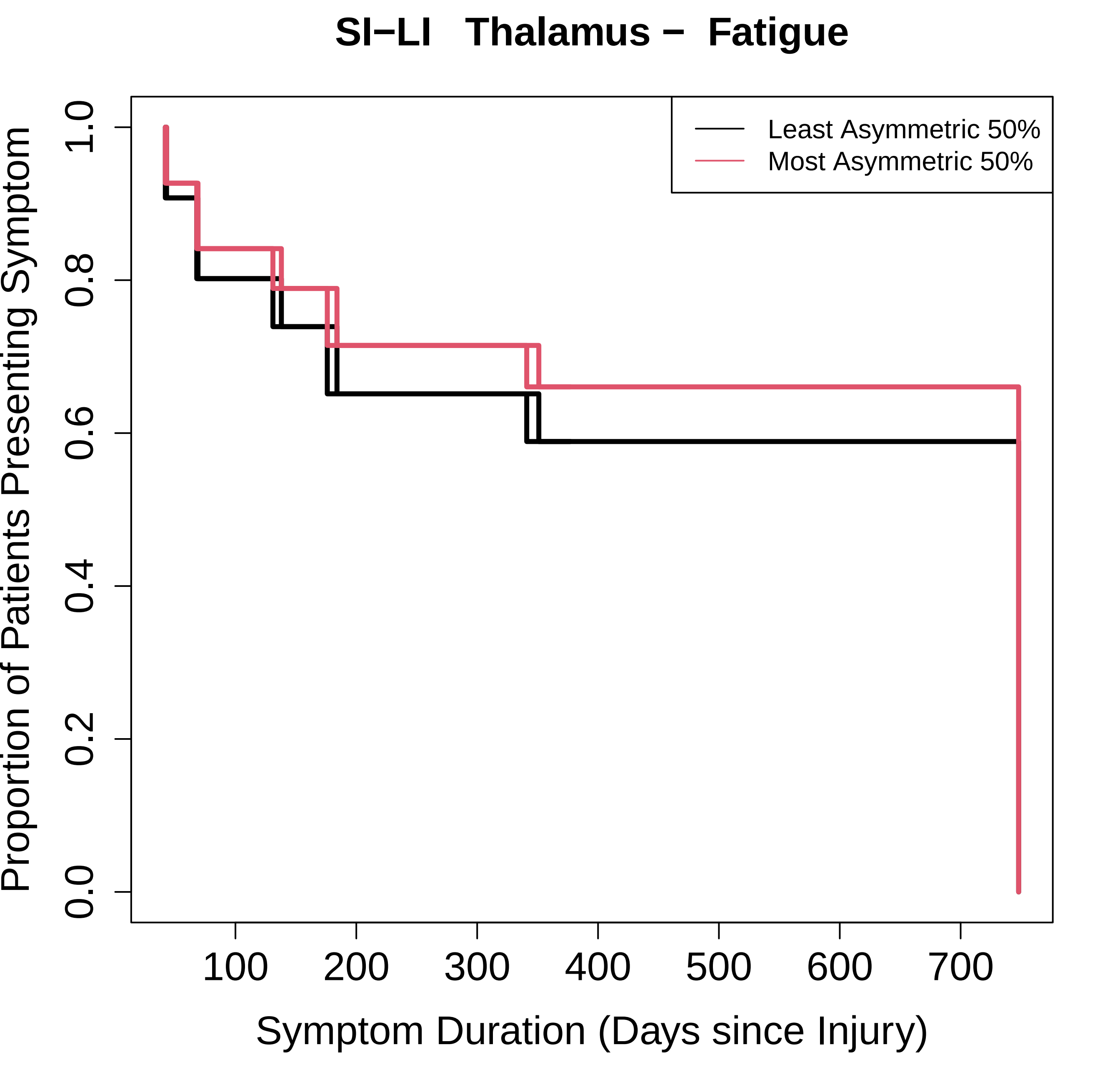
SI−LI Thalamus − Fatigue
Least Asymmetric 50%
Most Asymmetric 50%
Proportion of Patients Presenting Symptom
0.0
0.2
0.4
0.6
0.8
1.0
100
200
300
400
500
600
700
Symptom Duration (Days since Injury)

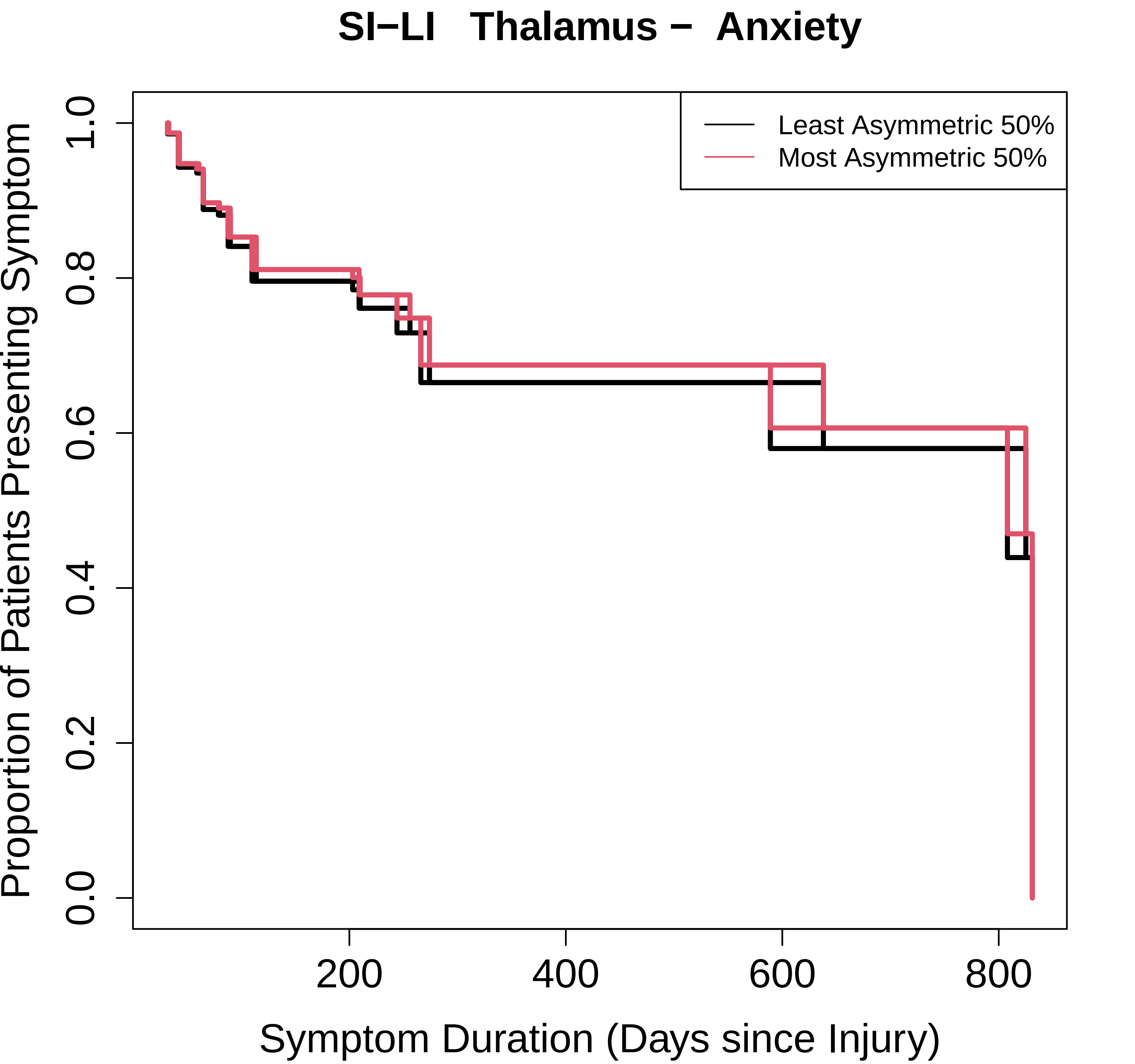

SI−LI Thalamus − Anxiety
Least Asymmetric 50%
Most Asymmetric 50%
Proportion of Patients Presenting Symptom
0.0
0.2
0.4
0.6
0.8
1.0
200
400
600
800
Symptom Duration (Days since Injury)

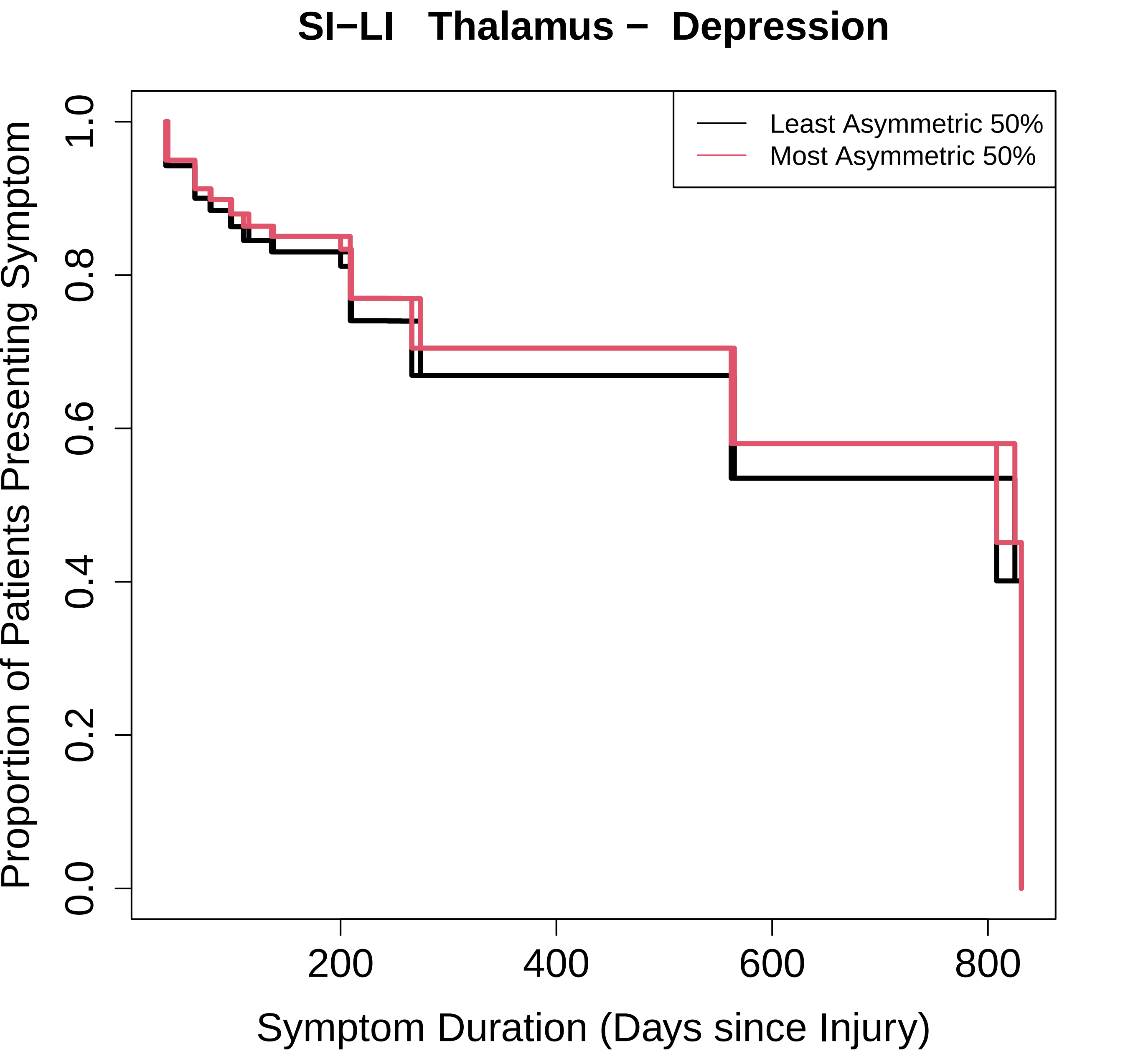
SI−LI Thalamus − Depression
Least Asymmetric 50%
Most Asymmetric 50%
Proportion of Patients Presenting Symptom
0.0
0.2
0.4
0.6
0.8
1.0
200
400
600
800
Symptom Duration (Days since Injury)

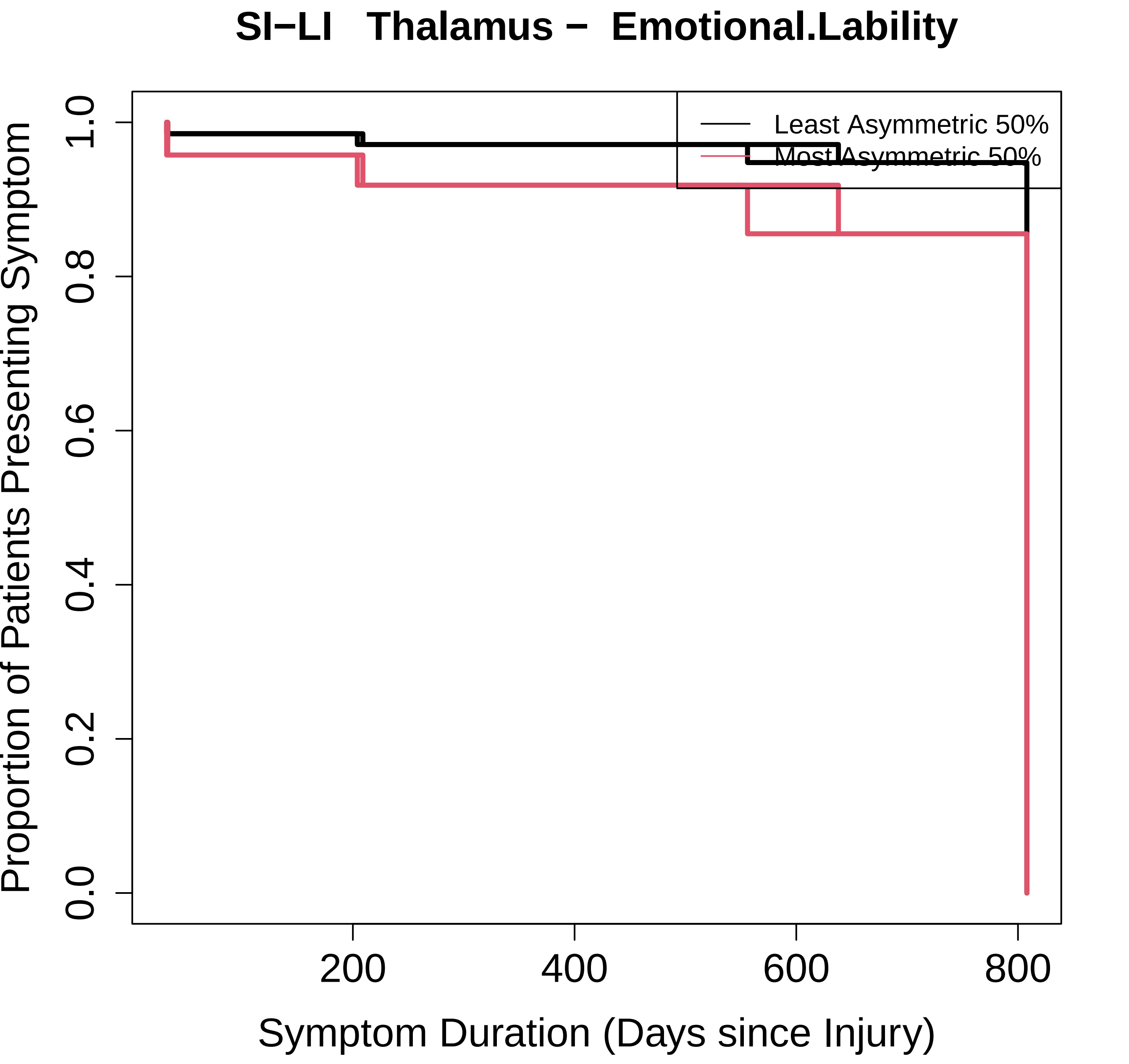
SI−LI Thalamus − Emotional.Lability
Least Asymmetric 50%
Most Asymmetric 50%
Proportion of Patients Presenting Symptom
0.0
0.2
0.4
0.6
0.8
1.0
200
400
600
800
Symptom Duration (Days since Injury)
