# Supplementary material for: Symptom Persistence Relates to Volume and Asymmetry of the Limbic System after Mild Traumatic Brain Injury
Source: J Clin Med. 2024 Aug 30;13(17):5154. doi: 10.3390/jcm13175154 (PMC11396354; doi:10.3390/jcm13175154)
Supplement: Supplementary file 1 [file jcm-13-05154-s001.zip › jcm-3116055-Supplemental Tables.docx]

*Supplementary materials*

**Symptom persistence relates to volume and asymmetry of the limbic system after mild traumatic brain injury**

**Cheryl Vanier^1,2¶*^, Priya Santhanam^1¶^, Nicholas Rochester^1,3^, Lauren Carter^1^, Mike Lim^4^, Amir Kilani^4^, Shivani Venkatesh^2^, Sherwin Azad^4^, Thomas Knoblauch^1,5^, Tapasya Surti^6^, Colin Brown^2^, Justin Sanchez^2^, Leon Ma^7^, Shaunaq Parikh^8^, Leo Germin^9^, Enrico Fazzini^2^ and Travis Snyder^1,2,4,10,11^**

¶Authors contributed equally to this manuscript

1: Imgen Research Group, Las Vegas NV, USA

2: Touro University Nevada College of Osteopathic Medicine, Henderson NV, USA

3: Central Michigan University College of Medicine, Saginaw MI, USA

4: Sunrise Health Graduate Medical Education Consortium, Department of Radiology, Las Vegas NV, USA

5: Department of Interdisciplinary Health Sciences, University of Nevada, Las Vegas, Las Vegas NV, USA

6: University of Texas Health Science Center, Houston TX, USA

7: Loyola University Medical Center, Department of Anesthesiology, Maywood IL, USA

8: University of Pittsburgh, Department of Family Medicine, Pittsburgh PA, USA

9: Clinical Neurology Specialists, Las Vegas NV, USA

10: HCA Healthcare (Nashville, TN), Mountain View Hospital, Las Vegas NV. USA

11: SimonMed Imaging, Las Vegas NV, USA

***** Correspondence: cheryl.vanier@imgen-research.com

**Supplemental Tables**

**Table S1.** Most common combinations of symptoms (Balance problems=Bal, Cognitive deficits=Cog, Fatigue=Fat, Anxiety=Anx, Depression=Dep, Emotional lability=EL). Headache was not included in combination with other groups because 96% of participants reported having headaches. The expected values are the calculated number of participants in a symptom group based on the probability of symptoms co-occurring or not. The 95% confidence interval (CI) is based on the observed values for each symptom group. Expected values that lie outside the 95% CI were evidence for incongruence between observed and expected values. The larger value between E and O is in bold.

| **#** | **Symptoms** | **Expected** | **Observed** | **95% CI** | |
| --- | --- | --- | --- | --- | --- |
| 1 | Headache | 10 | **53** | 46 | 58 |
| 1 | Cog | 34 | **55** | 45 | 64 |
| 1 | Bal | 25 | **44** | 35 | 52 |
| 2 | Bal+Cog | 86 | **125** | 110 | 139 |
| 2 | Cog+Dep | **14** | 2 | 0 | 6 |
| 2 | Cog+Anx | **18** | 4 | 1 | 9 |
| 2 | Cog+Fat | **10** | 4 | 1 | 8 |
| 2 | Bal+Anx | **13** | 4 | 1 | 8 |
| 3 | Bal+Cog+EL | **18** | 11 | 6 | 17 |
| 3 | Bal+Cog+Dep | **36** | 3 | 1 | 8 |
| 3 | Bal+Cog+Anx | **45** | 10 | 5 | 17 |
| 3 | Bal+Cog+Fat | **25** | 11 | 6 | 17 |
| 4 | Bal+Cog+Anx+Dep | 19 | **49** | 41 | 56 |
| 4 | Bal+Cog+Fat+EL | 5 | **12** | 7 | 15 |
| 4 | Bal+Cog+Fat+Dep | **10** | 2 | 0 | 6 |
| 4 | Bal+Cog+Fat+Anx | **13** | 6 | 2 | 11 |
| 5 | Bal+Cog+Anx+Dep+EL | 4 | **19** | 14 | 22 |
| 5 | Bal+Cog+Fat+Anx+Dep | 1 | **17** | 13 | 18 |
| 6 | Bal+Cog+Fat+Anx+Dep+EL | 5 | **36** | 30 | 39 |

**Table S2.** Right and left side limbic structure volumes (cm^3^), adjusted for TIV using regression, shown as mean and standard deviation (SD). Derived values used in the analysis (total volume, laterality indices) are in Table S3.

|  | **Total** | | **No LOC** | | **LOC** | |
| --- | --- | --- | --- | --- | --- | --- |
|  | **Left** | **Right** | **Left** | **Right** | **Left** | **Right** |
| Amygdala | 1.86 (0.29) | 1.73 (0.26) | 1.86 (0.30) | 1.73 (0.27) | 1.86 (0.28) | 1.74 (0.26) |
| Parahippocampus | 2.33 (0.27) | 2.38 (0.30) | 2.33 (0.26) | 2.39 (0.30) | 2.33 (0.29) | 2.36 (0.31) |
| Hippocampus | 4.07 (0.40) | 4.19 (0.41) | 4.07 (0.40) | 4.19 (0.42) | 4.08 (0.38) | 4.19 (0.37) |
| Entorhinal cortex | 2.70 (0.53) | 2.52 (0.54) | 2.71 (0.53) | 2.55 (0.53) | 2.65 (0.53) | 2.45 (0.55) |
| Rostral ACC | 1.26 (0.21) | 2.05 (0.37) | 1.26 (0.20) | 2.05 (0.36) | 1.27 (0.23) | 2.08 (0.40) |
| Caudal ACC | 0.80 (0.22) | 1.59 (0.34) | 0.81 (0.23) | 1.60 (0.34) | 0.78 (0.21) | 1.56 (0.36) |
| PCC | 1.52 (0.34) | 1.43 (0.31) | 1.53 (0.35) | 1.44 (0.31) | 1.49 (0.34) | 1.40 (0.31) |
| Isthmus Cingulate | 2.52 (0.37) | 2.13 (0.29) | 2.53 (0.38) | 2.15 (0.30) | 2.50 (0.36) | 2.11 (0.28) |
| Lateral OFC | 10.20 (1.12) | 9.20 (0.98) | 10.15 (1.12) | 9.17 (0.94) | 10.30 (1.13) | 9.30 (1.07) |
| Medial OFC | 4.60 (0.52) | 7.76 (0.96) | 4.59 (0.49) | 7.74 (0.92) | 4.64 (0.58) | 7.81 (1.08) |
| Ventral Diencephalon | 3.57 (0.43) | 3.55 (0.44) | 3.58 (0.44) | 3.55 (0.46) | 3.54 (0.40) | 3.54 (0.41) |
| Nucleus Accumbens | 0.64 (0.11) | 0.60 (0.11) | 0.65 (0.11) | 0.60 (0.11) | 0.64 (0.12) | 0.60 (0.11) |
| Thalamus | 7.90 (0.75) | 7.92 (0.79) | 7.86 (0.76) | 7.87 (0.78) | 8.01 (0.71) | 8.03 (0.80) |

**Table S3**. Mean and standard deviation (SD) for total regional volume (cm^3^), and median (interquartile range) for laterality index and side-independent laterality index for limbic structures. Adjusted right and left values that were used to compute total volume and indices are in Table S2. P-values are from t-tests or Mann-Whitney tests comparing LOC groups.

|  |  | **Total** | **no LOC** | **LOC** | **P-value** |
| --- | --- | --- | --- | --- | --- |
|  | TIV | 1493 (151) | 1475 (146) | 1537 (155) | **<0.001** |
| Total Volume | Amygdala | 3.59 (0.53) | 3.59 (0.53) | 3.6 (0.51) | 0.759 |
|  | Parahippocampal gyrus | 4.71 (0.51) | 4.72 (0.50) | 4.70 (0.53) | 0.721 |
|  | Hippocampus | 8.26 (0.75) | 8.26 (0.78) | 8.27 (0.69) | 0.866 |
|  | Entorhinal cortex | 5.22 (0.93) | 5.26 (0.93) | 5.10 (0.94) | 0.067 |
|  | Rostral ACC | 3.31 (0.53) | 3.3 (0.51) | 3.35 (0.59) | 0.364 |
|  | Caudal ACC | 2.39 (0.50) | 2.41 (0.49) | 2.35 (0.51) | 0.187 |
|  | PCC | 2.94 (0.63) | 2.96 (0.63) | 2.88 (0.62) | 0.181 |
|  | Isthmus Cingulate | 4.65 (0.62) | 4.67 (0.64) | 4.60 (0.59) | 0.252 |
|  | Lateral OFC | 19.40 (1.96) | 19.32 (1.92) | 19.60 (2.07) | 0.141 |
|  | Medial OFC | 12.37 (1.41) | 12.34 (1.33) | 12.45 (1.61) | 0.420 |
|  | Ventral Diencephalon | 7.12 (0.81) | 7.13 (0.83) | 7.09 (0.76) | 0.596 |
|  | Nucleus Accumbens | 1.24 (0.20) | 1.24 (0.20) | 1.23 (0.21) | 0.656 |
|  | Thalamus | 15.82 (1.44) | 15.73 (1.44) | 16.04 (1.42) | **0.028** |
| LI | Amygdala | -0.03  (-0.06, -0.00) | -0.03  (-0.07, -0.00) | -0.03  (-0.06, -0.00) | 0.440 |
|  | Parahippocampal gyrus | 0.01  (-0.02, 0.04) | 0.01  (-0.02, 0.05) | 0.01  (-0.03, 0.04) | 0.173 |
|  | Hippocampus | 0.02  (-0.00, 0.04) | 0.02  (-0.00, 0.03) | 0.02  (-0.00, 0.04) | 0.848 |
|  | Entorhinal cortex | -0.03  (-0.09, 0.02) | -0.03  (-0.09, 0.02) | -0.04  (-0.09, 0.01) | 0.328 |
|  | Rostral ACC | 0.24  (0.20, 0.28) | 0.24  (0.20, 0.28) | 0.23  (0.19, 0.28) | 0.971 |
|  | Caudal ACC | 0.33  (0.27, 0.40) | 0.33  (0.26, 0.40) | 0.33  (0.28, 0.40) | 0.939 |
|  | PCC | -0.03  (-0.07, 0.01) | -0.03  (-0.07, 0.01) | -0.03  (-0.07, 0.01) | 0.968 |
|  | Isthmus Cingulate | -0.08  (-0.12, -0.05) | -0.08  (-0.11, -0.05) | -0.09  (-0.12, -0.05) | 0.488 |
|  | Lateral OFC | -0.05  (-0.08, -0.02) | -0.05  (-0.08, -0.02) | -0.05  (-0.07, -0.03) | 0.869 |
|  | Medial OFC | 0.26  (0.23, 0.28) | 0.26  (0.23, 0.28) | 0.25  (0.23, 0.28) | 0.858 |
|  | Ventral Diencephalon | 0.00  (-0.03, 0.02) | 0.00  (-0.03, 0.02) | 0.00  (-0.03, 0.02) | 0.496 |
|  | Nucleus Accumbens | -0.04  (-0.08, 0.01) | -0.04  (-0.08, 0.00) | -0.03  (-0.08, 0.02) | 0.240 |
|  | Thalamus | 0.00  (-0.02, 0.02) | 0.00  (-0.02, 0.02) | 0.00  (-0.02, 0.02) | 0.862 |
| siLI | Amygdala | 0.03  (0.02, 0.06) | 0.03  (0.02, 0.06) | 0.03  (0.01, 0.06) | 0.661 |
|  | Parahippocampal gyrus | 0.03  (0.02, 0.06) | 0.03  (0.01, 0.06) | 0.04  (0.02, 0.06) | 0.172 |
|  | Hippocampus | 0.02  (0.01, 0.03) | 0.02  (0.01, 0.03) | 0.02  (0.01, 0.04) | 0.221 |
|  | Entorhinal cortex | 0.06  (0.02, 0.11) | 0.06  (0.03, 0.11) | 0.06  (0.02, 0.11) | 0.610 |
|  | Rostral ACC | 0.04  (0.02, 0.08) | 0.04  (0.02, 0.08) | 0.04  (0.02, 0.08) | 0.857 |
|  | Caudal ACC | 0.07  (0.03, 0.12) | 0.07  (0.03, 0.12) | 0.06  (0.03, 0.10) | 0.314 |
|  | PCC | 0.04  (0.02, 0.06) | 0.04  (0.02, 0.07) | 0.04  (0.02, 0.06) | 0.927 |
|  | Isthmus Cingulate | 0.03  (0.02, 0.06) | 0.03  (0.01, 0.06) | 0.04  (0.02, 0.06) | 0.216 |
|  | Lateral OFC | 0.03  (0.01, 0.04) | 0.03  (0.01, 0.05) | 0.02  (0.01, 0.04) | 0.389 |
|  | Medial OFC | 0.02  (0.01, 0.04) | 0.02  (0.01, 0.04) | 0.02  (0.01, 0.04) | 0.796 |
|  | Ventral Diencephalon | 0.03  (0.01, 0.05) | 0.03  (0.01, 0.05) | 0.03  (0.01, 0.05) | 0.185 |
|  | Nucleus Accumbens | 0.04  (0.02, 0.08) | 0.04  (0.02, 0.07) | 0.05  (0.02, 0.09) | 0.053 |
|  | Thalamus | 0.02  (0.01, 0.03) | 0.02  (0.01, 0.03) | 0.02  (0.01, 0.03) | 0.620 |

**Table S4.** Correlations among volumetric structures based on total volume (upper matrix), laterality index (center matrix), or side-independent laterality (lower matrix). For volume, the Pearson correlations of the z-scores computed from the TIV-adjusted values are reported. For LI and Si-LI, the Kendall’s tai correlations of the indices based on TIV-adjusted volumes are reported. Coefficients >|0.3| are bolded to draw attention to the relationships that were moderate or large.

|  | Amygdala | Parahippo | Hippoc | Entorh  Cortex | Rostral ACC | Caudal ACC | PCC | Isthmus Cing | Lateral OFC | Medial OFC | Ventral Dienceph | Nucleus Accumb | Thalamus |
| --- | --- | --- | --- | --- | --- | --- | --- | --- | --- | --- | --- | --- | --- |
| **Volume** |  |  |  |  |  |  |  |  |  |  |  |  |  |
| Parahipp. Gyrus | 0.08 |  |  |  |  |  |  |  |  |  |  |  |  |
| Hippocampus | **0.47** | **0.41** |  |  |  |  |  |  |  |  |  |  |  |
| Entorhinal Cortex | 0.05 | 0.23 | 0.07 |  |  |  |  |  |  |  |  |  |  |
| Rostral ACC | 0.20 | -0.07 | 0.04 | -0.03 |  |  |  |  |  |  |  |  |  |
| Caudal ACC | -0.15 | 0.02 | -0.08 | -0.02 | 0.15 |  |  |  |  |  |  |  |  |
| PCC | **-0.37** | -0.04 | -0.25 | 0.05 | 0.05 | 0.38 |  |  |  |  |  |  |  |
| Isthmus Cingulate | -0.03 | 0.15 | -0.09 | 0.14 | -0.04 | 0.17 | **0.34** |  |  |  |  |  |  |
| Lateral OFC | **0.37** | 0.11 | 0.23 | 0.06 | 0.31 | -0.05 | -0.17 | 0.05 |  |  |  |  |  |
| Medial OFC | 0.26 | 0.10 | 0.27 | 0.16 | 0.25 | -0.02 | -0.15 | 0.02 | **0.60** |  |  |  |  |
| Ventral Diencephalon | **0.43** | 0.15 | **0.47** | 0.02 | 0.03 | -0.16 | -0.43 | -0.11 | 0.14 | 0.14 |  |  |  |
| Nucleus Accumbens | **0.54** | 0.08 | 0.23 | -0.01 | 0.28 | -0.12 | -0.32 | -0.03 | **0.46** | **0.41** | 0.27 |  |  |
| Thalamus | 0.17 | 0.16 | 0.31 | 0.08 | -0.02 | -0.05 | -0.09 | 0.06 | 0.26 | **0.31** | 0.17 | 0.15 |  |
| TIV | 0.00 | 0.00 | 0.00 | 0.00 | 0.00 | 0.00 | 0.00 | 0.00 | 0.00 | 0.00 | 0.00 | 0.00 | 0.00 |
| **LI** |  |  |  |  |  |  |  |  |  |  |  |  |  |
| Parahipp. Gyrus | -0.01 |  |  |  |  |  |  |  |  |  |  |  |  |
| Hippocampus | 0.09 | 0.22 |  |  |  |  |  |  |  |  |  |  |  |
| Entorhinal Cortex | 0.12 | 0.02 | 0.08 |  |  |  |  |  |  |  |  |  |  |
| Rostral ACC | -0.01 | 0.00 | 0.04 | 0.04 |  |  |  |  |  |  |  |  |  |
| Caudal ACC | -0.04 | 0.04 | 0.03 | 0.01 | 0.19 |  |  |  |  |  |  |  |  |
| PCC | 0.00 | 0.02 | 0.00 | -0.02 | -0.03 | 0.08 |  |  |  |  |  |  |  |
| Isthmus Cingulate | 0.05 | 0.03 | 0.09 | -0.01 | -0.01 | 0.03 | 0.14 |  |  |  |  |  |  |
| Lateral OFC | -0.05 | -0.02 | -0.01 | -0.01 | -0.05 | -0.06 | 0.00 | -0.02 |  |  |  |  |  |
| Medial OFC | -0.01 | 0.01 | -0.01 | 0.05 | 0.13 | 0.17 | -0.04 | -0.06 | 0.02 |  |  |  |  |
| Ventral Diencephalon | 0.00 | 0.09 | 0.14 | 0.02 | 0.06 | 0.04 | -0.05 | -0.01 | -0.03 | 0.10 |  |  |  |
| Nucleus Accumbens | -0.02 | 0.00 | 0.02 | 0.00 | 0.02 | 0.07 | 0.05 | -0.01 | 0.02 | 0.06 | -0.01 |  |  |
| Thalamus | 0.01 | 0.00 | 0.01 | -0.01 | 0.05 | 0.08 | -0.02 | -0.04 | 0.00 | 0.06 | 0.09 | 0.05 |  |
| TIV | 0.00 | 0.00 | 0.04 | 0.02 | -0.01 | 0.00 | -0.01 | 0.00 | 0.00 | 0.00 | 0.01 | 0.00 | 0.00 |
| **siLI** |  |  |  |  |  |  |  |  |  |  |  |  |  |
| Parahipp. Gyrus | -0.02 |  |  |  |  |  |  |  |  |  |  |  |  |
| Hippocampus | 0.01 | 0.06 |  |  |  |  |  |  |  |  |  |  |  |
| Entorhinal Cortex | 0.05 | 0.02 | 0.01 |  |  |  |  |  |  |  |  |  |  |
| Rostral ACC | 0.02 | -0.01 | 0.01 | 0.01 |  |  |  |  |  |  |  |  |  |
| Caudal ACC | 0.03 | 0.00 | 0.03 | -0.01 | 0.05 |  |  |  |  |  |  |  |  |
| PCC | -0.01 | 0.00 | 0.03 | 0.06 | 0.01 | 0.01 |  |  |  |  |  |  |  |
| Isthmus Cingulate | -0.02 | -0.07 | 0.06 | 0.03 | 0.05 | 0.04 | 0.04 |  |  |  |  |  |  |
| Lateral OFC | 0.02 | -0.04 | 0.00 | 0.04 | 0.06 | 0.03 | 0.05 | -0.01 |  |  |  |  |  |
| Medial OFC | 0.04 | 0.06 | 0.04 | 0.01 | 0.04 | 0.08 | 0.03 | 0.00 | 0.01 |  |  |  |  |
| Ventral Diencephalon | 0.01 | 0.01 | 0.04 | -0.02 | -0.01 | -0.02 | 0.06 | 0.08 | 0.00 | 0.04 |  |  |  |
| Nucleus Accumbens | 0.02 | -0.01 | 0.04 | -0.04 | 0.03 | 0.02 | -0.03 | 0.04 | 0.02 | 0.05 | 0.06 |  |  |
| Thalamus | 0.03 | -0.02 | 0.07 | -0.02 | -0.02 | -0.01 | 0.00 | 0.06 | -0.03 | -0.01 | 0.08 | 0.06 |  |
| TIV | 0.07 | 0.08 | 0.09 | 0.14 | 0.09 | 0.06 | 0.00 | 0.10 | 0.08 | 0.11 | 0.02 | 0.02 | 0.04 |

**Table S5**. Mean (SE) z-scores for volume, and median (interquartile range) for laterality index (LI) and side-independent laterality (siLI), reported separately for the groups that did not (-) / did (+) have each symptom. For age, sex, and LOC covariates, the percent of participants who had a characteristic (e.g.,<40, F) for those who did not (-) and those who had (+) a symptom are reported. For DOI to DOS, the median and interquartile range endpoints are reported. Values corresponding to odds ratios having p<0.05 from Table 4 are in bold. Reference condition for age was <40, for sex was female, and for LOC none.

|  |  | **Headache** | **Balance** | **Cognitive** | **Fatigue** | **Anxiety** | **Depression** | **Emotional Lability** |
| --- | --- | --- | --- | --- | --- | --- | --- | --- |
| **Covariates** |  |  |  |  |  |  |  |  |
| Age | >40  <40 | 97%  95% | 67%  75% | 74%  80% | 22%  23% | 33%  35% | 28%  30% | 15%  19% |
| Sex | M  F | 97%  94% | 74%  68% | 77%  77% | 21%  25% | 36%  32% | 29%  30% | 15%  20% |
| LOC | No LOC  LOC | 96%  97% | **69%**  **79%** | **74%**  **85%** | **20%**  **29%** | 32%  40% | **24%**  **42%** | **14%**  **26%** |
| DOI to DOS | -Symptom | 141 (93,286) | 103 (64,190) | 100 (58,159) | 98 (58,173) | 97 (57,176) | 96 (57,168) | 99 (57,173) |
|  | +Symptom | 98 (56,173) | 94 (56,168) | 98 (56,181) | 101 (50,189) | 101 (56,172) | 105 (55,210) | 90 (56,184) |
| **Volume z-scores (mean, SE)** | |  |  |  |  |  |  |  |
| Amygdala | -Symptom | -0.05 (0.19) | 0.16 (0.08) | 0.20 (0.10) | 0.04 (0.05) | 0.03 (0.05) | 0.03 (0.05) | 0.03 (0.05) |
|  | +Symptom | 0.00 (0.04) | -0.06 (0.05) | -0.06 (0.05) | -0.13 (0.09) | -0.06 (0.08) | -0.07 (0.08) | -0.12 (0.10) |
| Parahipp Gyrus | -Symptom | 0.17 (0.20) | 0.05 (0.08) | 0.13 (0.09) | 0.02 (0.05) | 0.01 (0.05) | 0.00 (0.05) | 0.04 (0.05) |
|  | +Symptom | -0.01 (0.04) | -0.02 (0.05) | -0.04 (0.05) | -0.06 (0.09) | -0.03 (0.07) | 0.00 (0.08) | -0.19 (0.11) |
| Hippocampus | -Symptom | 0.11 (0.21) | 0.12 (0.08) | 0.09 (0.09) | 0.05 (0.05) | 0.00 (0.05) | 0.01 (0.05) | 0.04 (0.05) |
|  | +Symptom | 0.00 (0.04) | -0.05 (0.05) | -0.03 (0.05) | -0.18 (0.10) | 0.00 (0.08) | -0.04 (0.08) | -0.20 (0.10) |
| Entorhinal Cortex | -Symptom | 0.09 (0.23) | 0.11 (0.09) | 0.18 (0.09) | 0.00 (0.05) | 0.04 (0.05) | 0.03 (0.05) | 0.00 (0.05) |
|  | +Symptom | 0.00 (0.04) | -0.04 (0.05) | -0.05 (0.05) | 0.01 (0.10) | -0.08 (0.07) | -0.08 (0.08) | -0.02 (0.11) |
| Rostral ACC | -Symptom | -0.29 (0.20) | 0.06 (0.08) | 0.02 (0.09) | 0.03 (0.05) | -0.04 (0.06) | -0.02 (0.05) | -0.02 (0.05) |
|  | +Symptom | 0.01 (0.04) | -0.02 (0.05) | 0.00 (0.05) | -0.09 (0.09) | 0.07 (0.07) | 0.05 (0.07) | 0.12 (0.12) |
| Caudal ACC | -Symptom | 0.39 (0.25) | 0.01 (0.08) | 0 (0.10) | 0.04 (0.05) | 0 (0.05) | 0.02 (0.05) | 0.01 (0.05) |
|  | +Symptom | -0.02 (0.04) | 0.00 (0.05) | 0 (0.05) | -0.15 (0.09) | 0 (0.08) | -0.05 (0.08) | -0.05 (0.10) |
| PCC | -Symptom | 0.08 (0.23) | -0.17 (0.08) | -0.19 (0.09) | -0.01 (0.05) | -0.08 (0.05) | -0.04 (0.05) | -0.01 (0.05) |
|  | +Symptom | 0.00 (0.04) | 0.07 (0.05) | 0.05 (0.05) | 0.03 (0.09) | 0.15 (0.08) | 0.10 (0.08) | 0.06 (0.10) |
| Isthmus Cingulate | -Symptom | 0.20 (0.25) | 0 (0.08) | 0.11 (0.08) | -0.01 (0.05) | -0.03 (0.05) | 0.03 (0.05) | 0.05 (0.05) |
|  | +Symptom | -0.01 (0.04) | 0 (0.05) | -0.03 (0.05) | 0.03 (0.10) | 0.06 (0.08) | -0.07 (0.08) | -0.22 (0.10) |
| Lateral OFC | -Symptom | -0.15 (0.23) | 0.20 (0.08) | 0.20 (0.09) | 0.02 (0.05) | 0 (0.05) | -0.01 (0.05) | 0.02 (0.05) |
|  | +Symptom | 0.01 (0.04) | -0.08 (0.05) | -0.06 (0.05) | -0.07 (0.01) | -0.01 (0.08) | 0.02 (0.08) | -0.09 (0.11) |
| Medial OFC | -Symptom | -0.31 (0.20) | 0.06 (0.07) | 0.15 (0.08) | 0.02 (0.05) | -0.01 (0.05) | -0.03 (0.05) | 0.01 (0.05) |
|  | +Symptom | 0.01 (0.04) | -0.03 (0.05) | -0.04 (0.05) | -0.08 (0.11) | 0.02 (0.08) | 0.06 (0.08) | -0.06 (0.11) |
| Ventral Diencephalon | -Symptom | -0.22 (0.21) | 0.12 (0.08) | 0.03 (0.09) | 0.04 (0.05) | 0.07 (0.05) | 0.06 (0.05) | 0.03 (0.05) |
|  | +Symptom | 0.01 (0.04) | -0.05 (0.05) | -0.01 (0.05) | -0.14 (0.09) | -0.13 (0.07) | -0.13 (0.08) | -0.16 (0.10) |
| Nucleus Accumbens | -Symptom | -0.08 (0.23) | 0.18 (0.08) | 0.33 (0.10) | 0.06 (0.05) | 0.08 (0.05) | 0.07 (0.05) | 0.04 (0.05) |
|  | +Symptom | 0.00 (0.04) | -0.07 (0.05) | -0.10 (0.05) | -0.21 (0.09) | -0.15 (0.07) | -0.18 (0.08) | -0.18 (0.09) |
| Thalamus | -Symptom | **0.59 (0.25)** | 0.02 (0.08) | 0.07 (0.10) | -0.03 (0.05) | -0.06 (0.06) | -0.05 (0.05) | 0.01 (0.05) |
|  | +Symptom | **-0.02 (0.04)** | -0.01 (0.05) | -0.02 (0.05) | 0.12 (0.09) | 0.11 (0.07) | 0.13 (0.07) | -0.05 (0.09) |
| **Laterality index (Median, IQR)** | | |  |  |  |  |  |  |
| Amygdala | -Symptom | -0.05 (-0.09, -0.03) | -0.04 (-0.07, -0.01) | -0.04 (-0.08, -0.00) | -0.03 (-0.06, -0.00) | -0.03 (-0.07, -0.00) | -0.03 (-0.07, -0.00) | -0.03 (-0.06, -0.00) |
|  | +Symptom | -0.03 (-0.06, -0.00) | -0.03 (-0.06, 0.00) | -0.03 (-0.06, -0.00) | -0.04 (-0.07, -0.00) | -0.03 (-0.06, 0.00) | -0.03 (-0.06, 0.00) | -0.03 (-0.07, -0.00) |
| Parahipp Gyrus | -Symptom | 0.01 (-0.04, 0.06) | 0.02 (-0.02, 0.05) | 0.02 (-0.01, 0.05) | 0.01 (-0.02, 0.05) | 0.01 (-0.02, 0.05) | 0.01 (-0.02, 0.05) | 0.01 (-0.02, 0.05) |
|  | +Symptom | 0.01 (-0.02, 0.04) | 0.01 (-0.02, 0.04) | 0.01 (-0.03, 0.04) | 0.01 (-0.02, 0.03) | 0.01 (-0.03, 0.04) | 0 (-0.03, 0.04) | 0.01 (-0.03, 0.04) |
| Hippocampus | -Symptom | 0.01 (0.00, 0.05) | 0.02 (0.00, 0.03) | 0.02 (0.00, 0.04) | 0.02 (0.00, 0.04) | 0.02 (0.00, 0.04) | 0.02 (-0.00, 0.03) | 0.02 (-0.00, 0.03) |
|  | +Symptom | 0.02 (-0.00, 0.04) | 0.02 (-0.00, 0.04) | 0.01 (-0.00, 0.03) | 0.01 (-0.01, 0.04) | 0.01 (-0.01, 0.03) | 0.02 (-0.00, 0.04) | 0.01 (-0.00, 0.04) |
| Entorhinal Cortex | -Symptom | -0.02 (-0.09, 0.04) | -0.02 (-0.10, 0.05) | -0.04 (-0.09, 0.01) | -0.03 (-0.09, 0.02) | -0.04 (-0.10, 0.02) | -0.04 (-0.09, 0.01) | -0.04 (-0.10, 0.01) |
|  | +Symptom | -0.04 (-0.09, 0.02) | -0.04 (-0.09, 0.01) | -0.03 (-0.09, 0.03) | -0.04 (-0.09, 0.03) | -0.03 (-0.08, 0.03) | -0.03 (-0.09, 0.03) | -0.02 (-0.08, 0.04) |
| Rostral ACC | -Symptom | 0.23 (0.18, 0.27) | 0.25 (0.21, 0.29) | 0.24 (0.20, 0.29) | 0.24 (0.20, 0.28) | 0.24 (0.20, 0.28) | 0.24 (0.20, 0.28) | 0.24 (0.20, 0.28) |
|  | +Symptom | 0.24 (0.20, 0.28) | 0.24 (0.19, 0.28) | 0.24 (0.20, 0.28) | 0.24 (0.18, 0.28) | 0.24 (0.19, 0.28) | 0.24 (0.20, 0.28) | 0.24 (0.19, 0.27) |
| Caudal ACC | -Symptom | 0.39 (0.29, 0.41) | 0.36 (0.29, 0.42) | 0.34 (0.28, 0.41) | 0.33 (0.27, 0.40) | 0.33 (0.27, 0.41) | 0.33 (0.27, 0.40) | 0.34 (0.27, 0.40) |
|  | +Symptom | 0.33 (0.27, 0.40) | 0.32 (0.26, 0.39) | 0.33 (0.26, 0.40) | 0.33 (0.25, 0.39) | 0.33 (0.27, 0.39) | 0.34 (0.27, 0.39) | 0.32 (0.24, 0.40) |
| PCC | -Symptom | -0.03 (-0.05, 0.01) | -0.03 (-0.07, 0.02) | -0.03 (-0.07, 0.02) | -0.03 (-0.07, 0.01) | -0.03 (-0.07, 0.01) | -0.03 (-0.07, 0.01) | -0.03 (-0.07, 0.01) |
|  | +Symptom | -0.03 (-0.07, 0.01) | -0.03 (-0.07, 0.01) | -0.03 (-0.07, 0.01) | -0.03 (-0.07, 0.01) | -0.03 (-0.07, 0.01) | -0.04 (-0.07, 0.00) | -0.03 (-0.08, 0.01) |
| Isthmus Cingulate | -Symptom | -0.07 (-0.10, -0.05) | -0.08 (-0.11, -0.04) | -0.08 (-0.10, -0.04) | -0.08 (-0.12, -0.05) | -0.08 (-0.11, -0.05) | -0.08 (-0.11, -0.05) | -0.08 (-0.11, -0.05) |
|  | +Symptom | -0.08 (-0.12, -0.05) | -0.08 (-0.12, -0.05) | -0.08 (-0.12, -0.05) | -0.08 (-0.11, -0.05) | -0.08 (-0.12, -0.05) | -0.08 (-0.12, -0.05) | -0.09 (-0.12, -0.05) |
| Lateral OFC | -Symptom | -0.06 (-0.09, -0.03) | -0.06 (-0.08, -0.03) | -0.06 (-0.08, -0.03) | -0.05 (-0.08, -0.03) | -0.05 (-0.08, -0.03) | -0.05 (-0.08, -0.03) | -0.05 (-0.08, -0.02) |
|  | +Symptom | -0.05 (-0.08, -0.02) | -0.05 (-0.07, -0.02) | -0.05 (-0.08, -0.02) | -0.04 (-0.07, -0.02) | -0.05 (-0.08, -0.02) | -0.05 (-0.08, -0.02) | -0.06 (-0.08, -0.02) |
| Medial OFC | -Symptom | 0.26 (0.22, 0.28) | 0.26 (0.23, 0.28) | 0.25 (0.23, 0.27) | 0.26 (0.23, 0.28) | 0.26 (0.23, 0.28) | 0.26 (0.23, 0.28) | 0.26 (0.23, 0.28) |
|  | +Symptom | 0.26 (0.23, 0.28) | 0.25 (0.23, 0.28) | 0.26 (0.23, 0.28) | 0.25 (0.23, 0.28) | 0.25 (0.23, 0.27) | 0.25 (0.23, 0.27) | 0.25 (0.24, 0.28) |
| Ventral Diencephalon | -Symptom | 0.01 (-0.01, 0.03) | 0 (-0.03, 0.02) | -0.01 (-0.02, 0.02) | -0.01 (-0.03, 0.02) | -0.01 (-0.03, 0.02) | 0 (-0.03, 0.02) | 0 (-0.03, 0.02) |
|  | +Symptom | 0 (-0.03, 0.02) | 0 (-0.03, 0.02) | 0 (-0.03, 0.02) | 0.01 (-0.02, 0.03) | 0 (-0.03, 0.03) | 0 (-0.03, 0.03) | -0.01 (-0.03, 0.03) |
| Nucleus Accumbens | -Symptom | -0.04 (-0.08, 0.05) | -0.04 (-0.08, 0.00) | -0.04 (-0.09, 0.00) | -0.04 (-0.09, 0.01) | -0.04 (-0.08, 0.01) | -0.04 (-0.08, 0.01) | -0.04 (-0.08, 0.01) |
|  | +Symptom | -0.04 (-0.08, 0.01) | -0.04 (-0.08, 0.01) | -0.04 (-0.08, 0.01) | -0.03 (-0.07, 0.01) | -0.03 (-0.08, 0.01) | -0.03 (-0.08, 0.00) | -0.02 (-0.07, 0.01) |
| Thalamus | -Symptom | -0.01 (-0.02, 0.03) | 0 (-0.02, 0.02) | 0 (-0.02, 0.02) | 0 (-0.02, 0.02) | 0 (-0.02, 0.02) | 0 (-0.02, 0.02) | 0 (-0.02, 0.02) |
|  | +Symptom | 0 (-0.02, 0.02) | 0 (-0.02, 0.02) | 0 (-0.02, 0.02) | 0 (-0.02, 0.02) | 0 (-0.02, 0.02) | 0 (-0.02, 0.02) | 0 (-0.02, 0.02) |
| **siLI (Median, IQR)** | |  |  |  |  |  |  |  |
| Amygdala | -Symptom | 0.04 (0.02, 0.08) | 0.03 (0.01, 0.06) | 0.04 (0.02, 0.06) | 0.03 (0.01, 0.06) | 0.03 (0.02, 0.06) | 0.03 (0.02, 0.06) | 0.03 (0.02, 0.06) |
|  | +Symptom | 0.03 (0.02, 0.06) | 0.03 (0.02, 0.06) | 0.03 (0.02, 0.06) | 0.03 (0.02, 0.06) | 0.03 (0.02, 0.06) | 0.03 (0.02, 0.05) | 0.03 (0.01, 0.06) |
| Parahipp Gyrus | -Symptom | 0.05 (0.01, 0.10) | 0.04 (0.02, 0.06) | 0.03 (0.02, 0.06) | 0.04 (0.02, 0.06) | 0.03 (0.01, 0.06) | 0.03 (0.01, 0.06) | 0.03 (0.02, 0.06) |
|  | +Symptom | 0.03 (0.02, 0.06) | 0.03 (0.01, 0.06) | 0.03 (0.02, 0.06) | 0.03 (0.01, 0.06) | 0.03 (0.02, 0.06) | 0.03 (0.02, 0.06) | 0.03 (0.02, 0.06) |
| Hippocampus | -Symptom | 0.02 (0.01, 0.05) | 0.01 (0.01, 0.03) | 0.02 (0.01, 0.03) | 0.02 (0.01, 0.03) | 0.02 (0.01, 0.03) | 0.02 (0.01, 0.03) | 0.02 (0.01, 0.03) |
|  | +Symptom | 0.02 (0.01, 0.03) | 0.02 (0.01, 0.03) | 0.02 (0.01, 0.04) | 0.02 (0.01, 0.03) | 0.02 (0.01, 0.03) | 0.02 (0.01, 0.03) | 0.02 (0.01, 0.04) |
| Entorhinal Cort | -Symptom | 0.06 (0.05, 0.10) | 0.06 (0.03, 0.11) | 0.05 (0.02, 0.10) | 0.05 (0.02, 0.11) | 0.06 (0.03, 0.11) | 0.05 (0.02, 0.11) | 0.06 (0.03, 0.11) |
|  | +Symptom | 0.06 (0.02, 0.11) | 0.05 (0.02, 0.11) | 0.06 (0.03, 0.11) | 0.06 (0.02, 0.10) | 0.06 (0.02, 0.11) | 0.06 (0.02, 0.11) | 0.06 (0.02, 0.11) |
| Rostral ACC | -Symptom | 0.04 (0.03, 0.09) | 0.04 (0.02, 0.08) | 0.04 (0.02, 0.08) | 0.04 (0.02, 0.08) | 0.04 (0.02, 0.08) | 0.04 (0.02, 0.08) | 0.04 (0.02, 0.08) |
|  | +Symptom | 0.04 (0.02, 0.08) | 0.04 (0.02, 0.08) | 0.04 (0.02, 0.08) | 0.05 (0.02, 0.08) | 0.04 (0.02, 0.07) | 0.04 (0.02, 0.07) | 0.05 (0.02, 0.07) |
| Caudal ACC | -Symptom | 0.06 (0.05, 0.10) | 0.07 (0.04, 0.12) | 0.06 (0.03, 0.13) | 0.07 (0.03, 0.12) | 0.07 (0.03, 0.12) | 0.07 (0.03, 0.12) | 0.06 (0.03, 0.11) |
|  | +Symptom | 0.07 (0.03, 0.12) | 0.07 (0.03, 0.11) | 0.07 (0.03, 0.11) | 0.07 (0.03, 0.12) | 0.06 (0.03, 0.12) | 0.06 (0.03, 0.12) | 0.07 (0.03, 0.13) |
| PCC | -Symptom | 0.03 (0.01, 0.05) | 0.04 (0.02, 0.06) | 0.04 (0.02, 0.07) | 0.04 (0.02, 0.06) | 0.04 (0.02, 0.06) | 0.04 (0.02, 0.06) | 0.04 (0.02, 0.06) |
|  | +Symptom | 0.04 (0.02, 0.06) | 0.04 (0.02, 0.07) | 0.04 (0.02, 0.06) | 0.04 (0.02, 0.07) | 0.04 (0.02, 0.06) | 0.04 (0.02, 0.07) | 0.05 (0.02, 0.07) |
| Isthmus Cing | -Symptom | 0.03 (0.02, 0.04) | 0.04 (0.02, 0.06) | 0.03 (0.01, 0.06) | 0.03 (0.02, 0.06) | 0.03 (0.02, 0.06) | 0.03 (0.02, 0.06) | 0.03 (0.02, 0.06) |
|  | +Symptom | 0.03 (0.02, 0.06) | 0.03 (0.02, 0.06) | 0.03 (0.02, 0.06) | 0.03 (0.01, 0.06) | 0.03 (0.02, 0.06) | 0.03 (0.02, 0.06) | 0.03 (0.01, 0.05) |
| Lateral OFC | -Symptom | 0.03 (0.02, 0.05) | 0.02 (0.01, 0.05) | 0.03 (0.01, 0.05) | 0.02 (0.01, 0.04) | 0.02 (0.01, 0.05) | 0.03 (0.01, 0.05) | 0.03 (0.01, 0.05) |
|  | +Symptom | 0.03 (0.01, 0.04) | 0.03 (0.01, 0.04) | 0.03 (0.01, 0.04) | 0.03 (0.01, 0.04) | 0.03 (0.01, 0.04) | 0.03 (0.01, 0.04) | 0.03 (0.01, 0.04) |
| Medial OFC | -Symptom | 0.03 (0.02, 0.04) | 0.02 (0.01, 0.05) | 0.02 (0.01, 0.03) | 0.02 (0.01, 0.04) | 0.02 (0.01, 0.04) | 0.02 (0.01, 0.04) | 0.02 (0.01, 0.04) |
|  | +Symptom | 0.02 (0.01, 0.04) | 0.02 (0.01, 0.04) | 0.02 (0.01, 0.04) | 0.02 (0.01, 0.04) | 0.02 (0.01, 0.04) | 0.02 (0.01, 0.03) | 0.02 (0.01, 0.04) |
| Ventral Dienceph | -Symptom | 0.02 (0.02, 0.03) | 0.03 (0.01, 0.05) | 0.02 (0.01, 0.05) | 0.03 (0.01, 0.05) | 0.03 (0.01, 0.05) | 0.03 (0.01, 0.05) | 0.03 (0.01, 0.05) |
|  | +Symptom | 0.03 (0.01, 0.05) | 0.03 (0.01, 0.05) | 0.03 (0.01, 0.05) | 0.02 (0.01, 0.05) | 0.03 (0.01, 0.05) | 0.03 (0.01, 0.05) | 0.03 (0.01, 0.06) |
| Nucleus Accumbens | -Symptom | 0.05 (0.01, 0.09) | 0.04 (0.02, 0.07) | 0.05 (0.03, 0.08) | 0.05 (0.02, 0.08) | 0.05 (0.02, 0.08) | 0.05 (0.02, 0.08) | 0.05 (0.02, 0.08) |
|  | +Symptom | 0.04 (0.02, 0.08) | 0.05 (0.02, 0.08) | 0.04 (0.02, 0.08) | 0.04 (0.02, 0.07) | 0.04 (0.02, 0.07) | 0.04 (0.02, 0.06) | 0.04 (0.02, 0.07) |
| Thalamus | -Symptom | 0.03 (0.01, 0.05) | 0.02 (0.01, 0.03) | 0.02 (0.01, 0.03) | 0.02 (0.01, 0.03) | 0.02 (0.01, 0.03) | 0.02 (0.01, 0.03) | 0.02 (0.01, 0.03) |
|  | +Symptom | 0.02 (0.01, 0.03) | 0.02 (0.01, 0.03) | 0.02 (0.01, 0.04) | 0.02 (0.01, 0.03) | 0.02 (0.01, 0.04) | 0.02 (0.01, 0.04) | 0.02 (0.01, 0.03) |

**Table S6.** Adjusted odds of symptom presentation for laterality index (LI) and side-independent laterality (siLI) for limbic brain regions. Details follow Table 4. No p-values were<0.05 after the Benjamini-Hochberg adjustment.

|  |  | **Headache** | **Balance** | **Cognitive** | **Fatigue** | **Anxiety** | **Depression** | **Emotional Lability** |
| --- | --- | --- | --- | --- | --- | --- | --- | --- |
|  |  |  |  |  |  |  |  |  |
| **LI** |  |  |  |  |  |  |  |  |
|  | Amygdala | 1.23  0.50,1.88  ASLD | 1.16  0.87,1.41  ASLD | 1.12  0.75,1.50  ASLD | 0.94  0.77,1.89  ASL | 1.17  0.91,1.44  ASLD | 1.06  0.56,1.29  ASLD | 1.08  0.54,1.35  ASLD |
|  | Parahipp gyrus | 1.19  0.40,1.81  ASLD | 0.82  0.68,1.05  ASLD | 0.74  0.28,0.92  ASLD | 0.83  0.71,4.73  ASLD | 0.84  0.70,1.07  ASLD | 0.87  0.65,1.17  ASLD | 0.94  0.76,2.49  ASLD |
|  | Hippocampus | 0.83  0.53,2.48  ASLD | 0.97  0.83,3.75  ASLD | 0.80  0.64,1.04  ASLD | 0.87  0.71,1.20  ASL | 0.85  0.71,1.09  ASLD | 1.04  0.37,1.23  ASLD | 0.97  0.83,9.18  ASLD |
|  | Entorhinal cortex | 0.91  0.62,6.88  ASLD | 0.99  0.94,>100  ASLD | 1.11  0.72,1.37  ASLD | 1.05  0.42,1.27  ASL | 1.17  0.90,1.41  ASLD | 1.20  0.93,1.46  ASLD | 1.31  1.00,1.66  ASLD |
|  | Rostral ACC | 0.98  0.85,15.70  ASLD | 0.86  0.71,1.16  ASLD | 0.98  0.85,15.70  ASLD | 0.91  0.62,1.47  ASL | 0.98  0.85,8.21  ASLD | 1.11  0.75,1.35  ASLD | 0.93  0.74,1.91  ASLD |
|  | Caudal ACC | 0.66  0.40,1.34  ASLD | 0.76  0.57,0.97  ASLD | 0.98  0.83,12.47  ASLD | 0.82  0.66,1.75  ASLD | 0.91  0.76,1.32  SLD | 0.99  0.73,>100  ASLD | 0.86  0.50,1.35  ASLD |
|  | PCC | 0.83  0.54,2.53  ASLD | 0.94  0.78,1.70  ASLD | 0.90  0.74,1.40  ASLD | 1.04  0.03,2.15  ASLS*L* | 0.95  0.80,1.82  ASLD | 0.86  0.71,1.15  ASLD | 1.02  0.01,1.14  ASLD |
|  | Isthmus Cingulate | 0.82  0.53,2.09  ASLD | 0.85  0.70,1.12  ASLD | 0.83  0.68,1.11  ASLD | 0.94  0.77,1.93  ASL | 0.96  0.81,2.27  ASLD | 0.95  0.80,2.16  ASLD | 0.91  0.73,1.68  ASL |
|  | Lateral OFC | 1.24  0.51,1.90  ASLD | 1.26  1.01,1.53  ASLD | 1.11  0.71,1.36  ASLD | 1.16  0.23,1.37  ASLD | 1.07  0.62,1.27  ASLD | 1.08  0.65,1.30  ASLD | 0.95  0.47,3.15  ASLD |
|  | Medial OFC | 1.16  0.32,2.91  ASLD | 0.96  0.81,2.94  ASLD | 1.08  0.55,1.32  ASLD | 1.03  0.13,1.20  ASL | 1.01  0.01,1.10  ASLD | 1.03  0.14,1.19  ASLD | 1.13 0.73,11.13 ASLD |
|  | Ventral Diencephalon | 0.72  0.46,1.43  ASLD | 1.06  0.54,1.28  ASLD | 1.09  0.63,1.34  ASLD | 1.32  1.04,1.64  ASL | 1.06  0.56,1.27  ASLD | 1.07  0.57,1.29  ASLD | 1.15  0.72,1.45  ASLD |
|  | Nucleus Accumbens | 0.77  0.49,1.76  ASLD | 0.95  0.80,2.18  ASLD | 1.13  0.76,1.39  ASLD | 1.27  1.00,1.58  ASL | 0.98  0.88,14.87  ASLD | 0.97  0.83,3.60  ASLD | 1.17  0.79,1.80  ASLD |
|  | Thalamus | 1.09  0.14,1.58  ASLD | 1.02  0.09,1.15  ASLD | 0.96  0.81,3.29  ASLD | 0.96  0.80,2.61  ASL | 0.96  0.82,2.39  ASLD | 1.01  0.03,1.13  ASLD | 1.1  0.61,1.39  ASLD |
| **siLI** |  |  |  |  |  |  |  |  |
|  | Amygdala | 0.82  0.54,2.08  ASLD | 1.09  0.68,1.32  ASLD | 0.95  0.77,2.30  ASLD | 1.18  0.88,1.45  ASL | 1.06  0.59,1.26  ASLD | 1.03  0.23,1.20  ASLD | 0.99  0.96,>100  ASLD |
|  | Parahippocampus | 0.80  0.52,1.87  ASLD | 0.91  0.76,1.41  ASLD | 0.96  0.81,3.61  ASLD | 0.85  0.70,1.63  ASLD | 1.01  0.03,1.11  ASLD | 1.03  0.31,1.21  ASLD | 1.03  0.16,1.23  ASLD |
|  | Hippocampus | 0.87  0.59,3.25  ASLD | 1.16  0.86,1.41  ASLD | 1.25  0.96,1.56  ASLD | 1.11  0.74,1.36  ASL | 1.13  0.83,1.35  ASLD | 1.05  0.47,1.25  ASLD | 1.21  0.89,1.52  ASLD |
|  | Entorhinal cortex | 0.87  0.57,3.81  ASLD | 0.89  0.73,1.28  ASLD | 1.24  0.95,1.54  ASLD | 0.97  0.81,4.64  ASL | 0.96  0.82,2.54  ASLD | 1.06  0.54,1.28  ASLD | 0.99  0.00,1.34  ASLD |
|  | Rostral ACC | 0.86  0.56,2.92  ASLD | 0.98  0.87,12.67  ASLD | 0.91  0.75,1.51  ASLD | 1.01  0.02,1.13  ASL | 0.99  0.89,23.9  ASLD | 0.93  0.77,1.70  ASLD | 0.99  0.85,>100  ASLD |
|  | Caudal ACC | 0.94  0.66,21.37  ASLD | 0.98  0.84,5.90  ASLD | 0.97  0.81,4.39  ASLD | 1.03  0.19,1.21  ASL | 0.97  0.83,2.71  ASLD | 0.96  0.80,2.87  ASLD | 1.13  0.71,1.42  ASLD |
|  | PCC | 1.03  0.00,1.37  ASLD | 1.02  0.06,1.15  ASLD | 0.92  0.76,1.60  ASLD | 1.12  0.74,1.37  ASL | 1.00  0.54,>100  ASLD | 1.08  0.65,1.30  ASLD | 1.02  0.03,1.44  ASLD |
|  | Isthmus Cingulate | 1.18  0.37,1.82  ASLD | 0.95  0.79,1.93  ASLD | 1.07  0.55,1.31  ASLD | 0.91  0.74,1.52  ASL | 1.02  0.15,1.17  ASLD | 1.10  0.71,1.33  ASLD | 0.96  0.80,5.18  ASLD |
|  | Lateral OFC | 0.64  0.29,2.96  ASLDS*D* | 1.05  0.46,1.26  ASLD | 0.96  0.80,2.98  ASLD | 1.07  0.08,1.23  ASLD | 1.04  0.43,1.23  ASLD | 0.96  0.81,2.63  ASLD | 0.96  0.00,1.83  ASLD |
|  | Medial OFC | 0.49  0.24,1.20  ASLDS*D* | 0.92  0.76,1.46  ASLD | 1.16  0.83,1.43  ASLD | 1.05  0.43,1.27  ASL | 0.94  0.79,1.71  ASLD | 0.83  0.22,1.03  ASLD | 0.98  0.86,45.96  ASLD |
|  | Ventral Diencephalon | 1.29  0.54,2.05  ASLD | 1.05  0.44,1.25  ASLD | 1.09  0.64,1.33  ASLD | 1.02  0.03,1.14  ASL | 1.07  0.63,1.27  ASLD | 1.03  0.22,1.20  ASLD | 1.28  0.97,1.61  ASL |
|  | Nucleus Accumbens | 0.94  0.67,24.1  ASLD | 1.14  0.82,1.38  ASLD | 0.89  0.72,1.34  ASLD | 0.87  0.71,1.26  ASL | 0.91  0.76,1.32  ASLD | 0.83  0.68,1.06  ASLD | 0.87  0.69,1.34  ASLD |
|  | Thalamus | 0.71  0.47,1.23  ASLD | 1.08  0.62,1.30  ASLD | 1.22  0.93,1.52  ASLD | 0.89  0.71,1.38  ASL | 1.15  0.87,1.38  ASLD | 1.17  0.89,1.42  ASLD | 1.09  0.57,1.36  ASLD |

**Table S7**. Cutpoints for intervals in the TIV-adjusted scale for quartiles, used in Kaplan-Meier plots.

|  |  | **Minimum** | **Q1 to Q2** | **Q2 to Q3** | **Q3 to Q4** | **Maximum** |
| --- | --- | --- | --- | --- | --- | --- |
| LI |  |  |  |  |  |  |
|  | Amygdala | -0.21891 | -0.06435 | -0.03138 | -0.00203 | 0.13433 |
|  | Parahippocampus | -0.1564 | -0.0232 | 0.0112 | 0.045 | 0.1877 |
|  | Hippocampus | -0.162321 | -0.000272 | 0.016062 | 0.035643 | 0.134935 |
|  | Entorhinal cortex | -0.4064 | -0.0935 | -0.0348 | 0.0181 | 0.2692 |
|  | Rostral ACC | -0.0358 | 0.197 | 0.2387 | 0.281 | 0.427 |
|  | Caudal ACC | -0.0684 | 0.268 | 0.3325 | 0.403 | 0.631 |
|  | PCC | -0.1963 | -0.0696 | -0.0317 | 0.0115 | 0.1875 |
|  | Isthmus Cingulate | -0.2404 | -0.1153 | -0.0814 | -0.0494 | 0.0795 |
|  | Lateral OFC | -0.1881 | -0.076 | -0.052 | -0.0243 | 0.0655 |
|  | Medial OFC | 0.125 | 0.232 | 0.255 | 0.277 | 0.373 |
|  | Ventral Diencephalon | -0.14028 | -0.03047 | -0.00403 | 0.02251 | 0.15969 |
|  | Nucleus Accumbens | -0.3049 | -0.0812 | -0.03769 | 0.00793 | 0.15021 |
|  | Thalamus | -0.181956 | -0.016955 | -0.000288 | 0.019433 | 0.113342 |
| siLI |  |  |  |  |  |  |
|  | Amygdala | 5.74E-05 | 0.0158 | 3.13E-02 | 0.0576 | 0.1875 |
|  | Parahippocampus | 0.000218 | 0.0157 | 0.034184 | 0.0574 | 0.1767 |
|  | Hippocampus | 0.000038 | 0.00888 | 0.018371 | 0.03386 | 0.17835 |
|  | Entorhinal cortex | 0.00015 | 0.0246 | 0.05738 | 0.1084 | 0.3714 |
|  | Rostral ACC | 0.000133 | 0.019 | 0.042153 | 0.077 | 0.2744 |
|  | Caudal ACC | 0.00116 | 0.0336 | 0.06662 | 0.1162 | 0.3997 |
|  | PCC | 0.000193 | 0.0178 | 0.040384 | 0.0643 | 0.2194 |
|  | Isthmus Cingulate | 0.000146 | 0.0154 | 0.032841 | 0.0575 | 0.1611 |
|  | Lateral OFC | 1.09E-05 | 0.0121 | 2.56E-02 | 0.0447 | 0.1361 |
|  | Medial OFC | 2.36E-05 | 0.0096 | 2.24E-02 | 0.0389 | 0.1305 |
|  | Ventral Diencephalon | 2.86E-05 | 0.0118 | 2.66E-02 | 0.0486 | 0.1637 |
|  | Nucleus Accumbens | 0.000272 | 0.0209 | 0.04439 | 0.0778 | 0.2669 |
|  | Thalamus | 6.73E-05 | 0.00911 | 1.75E-02 | 0.03456 | 0.1816 |
